# Status and trends of orthophosphate concentrations in groundwater used for public supply in California

**DOI:** 10.1007/s10661-020-08504-x

**Published:** 2020-07-29

**Authors:** Robert Kent, Tyler D. Johnson, Michael R. Rosen

**Affiliations:** 1 U.S. Geological Survey California Water Science Center, 4165 Spruance Road, Suite 200, San Diego, CA 95101-0812 USA; 2 U.S. Geological Survey California Water Science Center, 2730 Deer Run Road, Carson City, NV 89701 USA

**Keywords:** California groundwater, Orthophosphate, Trend analysis, Water-quality monitoring, Public supply wells

## Abstract

Phosphorus is a necessary nutrient for all organisms. However excessive phosphorus can cause eutrophication in surface water. Groundwater can be an important nonpoint contributor of phosphorus to surface water bodies. Most groundwater phosphorus is in the form of orthophosphate and orthophosphate concentrations in California groundwater vary temporally and geographically. This study quantifies orthophosphate concentrations in water samples from public supply wells in California, evaluates temporal trends (both step and monotonic trends) in orthophosphate concentration for different areas of the state, and explores potential explanatory factors for the trends observed. Orthophosphate concentrations are low in 42 percent of the groundwater used for public supply in California, moderate in 43 percent, and high in 15 percent of this groundwater relative to reference conditions and a goal expressed by the USEPA for streams overlying the aquifers. The findings also suggest that orthophosphate concentrations increased in approximately one-third of this groundwater during the study period (2000 to 2018). The timing of orthophosphate increases observed in time-series evaluations coincided approximately with the timing of increases observed in step-trend evaluations, with both suggesting that the increasing trend occurred mostly before 2011. Principal component analysis (PCA) of the statewide dataset indicates that orthophosphate concentrations are antithetically related to dissolved oxygen (DO), and weakly associated with boron, arsenic, and fluoride. Step trend and time-series trend analyses using PCA were inconclusive.

**Electronic supplementary material:**

The online version of this article (10.1007/s10661-020-08504-x) contains supplementary material, which is available to authorized users.

## Introduction

Phosphorus is a necessary nutrient for all organisms, and is common in soils, rocks, and sediments (Hem 1992). However excessive phosphorus in surface water bodies can lead to eutrophication, because phosphate is often the limiting nutrient for the growth of aquatic plants in fresh water (Drever 1997; Litke 1999). Groundwater can be an important nonpoint contributor of phosphorus to surface water bodies (Litke 1999; U.S. Geological Survey 1999). Phosphorus occurs in natural water primarily as dissolved ortho-, pyro-, and polyphosphates (Hem 1992). Most groundwater phosphate is in the form of the orthophosphate (PO_4_^-^) ion (Domagalski and Johnson 2012), because it is more thermodynamically stable than the other common P^5+^ ions likely to occur in natural waters (Hem 1992). Orthophosphate is naturally present in rivers, streams, and lakes that recharge aquifers, as well as in the aquifer materials themselves from the erosion of rocks, and the recycling of animal waste and plant and animal tissue (Hem 1992).

In addition to naturally occurring orthophosphate, human activity also contributes orthophosphate to surface water that subsequently recharges aquifers. Agriculture contributes orthophosphate through use of chemical phosphorus fertilizers, manure, and composted materials (Domagalski and Johnson 2011, 2012). From about 1940 to 1970, orthophosphate, used as a calcium and magnesium-chelating agent in laundry detergent (Kogawa et al. 2017), was a major source of orthophosphate to the environment (U.S. Geological Survey 1999). From the 1970s to the 1990s the use of phosphate detergents declined due to mandated bans and voluntary cessation of its use (Litke 1999; U.S. Geological Survey 1999). The amount of phosphate discharged to the environment has also decreased, starting in the 1990s, as a result of upgraded wastewater treatment plants (U.S. Geological Survey 1999).

The US Environmental Protection Agency (USEPA) recommends nutrient concentration criteria that estimate reference conditions for rivers, streams, and lakes, by ecoregion, based on the 25th percentiles of all available nutrient data (USEPA 2000a, 2000b, 2001). Reference condition concentrations for total phosphorus in the twelve ecoregions located at least partially in California range from 0.009 to 0.077 milligrams per liter (mg/L) as P. The USEPA has also expressed desired phosphorus limits for the prevention of surface water eutrophication (USEPA 1986). The desired limit for total phosphates in streams flowing into a lake or reservoir is 0.050 mg/L as phosphorus (as P). The desired limit for total phosphorus in other flowing waters not directly discharging to lakes or reservoirs is 0.100 mg/L as P (Macenthun 1973). These other flowing waters that transport phosphorus to streams may include groundwater discharge (Domagalski and Johnson 2011). Dissolved phosphate occurs in small concentrations in natural water because it has low mobility, is readily taken up by biota, and adsorbs to metal oxides in soils (Hem 1992; Litke 1999). However, anthropogenic inputs can cause orthophosphate concentrations in natural waters, including groundwater, to be greater than reference conditions or the desired limits to prevent eutrophication (Holman et al. 2008).

It is imperative to understand how nutrients such as phosphorus change in concentration over time to better manage areas vulnerable to eutrophication. Groundwater quality changes over time are termed “temporal trends” in this study. Temporal trends of groundwater quality are difficult to assess due to the long time scales involved with groundwater movement and the resulting changes in quality, although relatively short-term studies are useful for monitoring the progress of local or statewide remediation efforts (McHugh et al. 2014; Saraceno et al. 2018; Stoline et al. 1993) or to observe short-period variability in groundwater quality (Granato and Smith 1999; MacDonald et al. 2017; Opsahl et al. 2017; Saraceno et al. 2018). In contrast to these studies are the long-term or continuing groundwater quality trend assessments conducted on regional or national spatial scales (e.g., Rosen 1999, 2001; Rosen and Lapham 2008), and these may reach century temporal scales (e.g., Hansen et al. 2018). Groundwater quality trend studies may focus on only one or two water-quality constituents (Hantzsche and Finnemore 1992; Rosen 2003; Batlle Aguilar et al. 2007; Burow et al. 2007; Landon et al. 2011; Kent and Landon 2013; Naranjo et al. 2013; Hansen et al. 2018) or several water-quality constituents (Stoline et al. 1993; Rosen 1999; Barlow et al. 2012; Lindsey and Rupert 2012; Kent and Landon 2016; Kent 2018). An understanding of how and why concentrations of water-quality constituents are changing over time is helpful to water resource managers as they plan for the future.

There have been several studies describing water-quality trend analysis methods (Hirsch et al. 1991; Loftis 1996; Grath et al. 2001; Rosen et al. 2008; Wahlin and Grimvall 2010; Lopez et al. 2014; Kent 2018), as well as studies that estimate the ability of these methods to assess and predict future groundwater quality (Hantzsche and Finnemore 1992; Stuart et al. 2007; Visser et al. 2009; Naranjo et al. 2013). Some trend evaluation studies are purely descriptive, involving neither formal hypothesis testing nor quantification, but include graphical methods and summary statistics (Bodo 1989; Esterby 1996; Jurgens et al. 2018). However, most studies that evaluate temporal trends in water quality use one of two statistical modes (Hirsch et al. 1991). The first mode performs hypothesis tests on the differences between two or more water-quality datasets collected at distinct time periods (Burow et al. 2008; Rupert 2008; Saad 2008; Barlow et al. 2012; Lindsey and Rupert 2012; Kent and Landon 2016; Kent 2018). Changes detected by this mode are sometimes referred to as step trends*.* Step trends, if they exist, are more likely to be detected with a greater number of sample pairs (Anderson 1987), and if there is a relatively long gap between the time periods (Hirsch et al. 1991). The second statistical mode performs correlation tests on data time series where time is the independent variable and some measure of water quality is the dependent variable (Stoline et al. 1993; Rosen 2003; Shipley and Rosen 2005; Batlle Aguilar et al. 2007; Landon et al. 2011; Kent and Landon 2013; Chaudhuri and Ale 2014). Changes detected by this mode are sometimes referred to as monotonic trends (Hirsch et al. 1991; Esterby 1996), and the European Water Framework Directive recommends at least 8 measurements when using this mode of trend analysis (Grath et al. 2001). The present paper quantifies orthophosphate concentrations in water samples from public supply wells in California, evaluates temporal trends (both step trends and monotonic) in orthophosphate concentration for different areas of the state, and explores potential explanatory factors for the trends observed.

### California Groundwater Ambient Monitoring and Assessment Program Priority Basin Project

The California State Water Resources Control Board implemented the Groundwater Ambient Monitoring and Assessment (GAMA) program to assess California groundwater quality (GAMA, http://www.waterboards.ca.gov/gama/). The GAMA Priority Basin Project (GAMA-PBP) is a component of GAMA, conducted in cooperation with the US Geological Survey (USGS) (http://ca.water.usgs.gov/gama/; Kulongoski and Belitz 2004-rev. 2006). GAMA-PBP is conducting three types of water-quality assessments as follows: (1) status of groundwater quality, (2) understanding of factors that affect groundwater quality, and (3) trends in groundwater quality. GAMA-PBP studies span all the major hydrogeologic provinces of California (Belitz et al. 2003), and use consistent methods to collect groundwater quality datasets (Koterba et al. 1995; U.S. Geological Survey variously dated). The statewide status and understanding assessments began in 2004 and were conducted by sequentially sampling 35 defined “study units” ranging in area from less than 80 km^2^ (Santa Barbara study unit) to more than 40,000 km^2^ (Sierra Nevada study unit) (Johnson et al. 2018) (Online Resource 1). The trend assessment began in 2007, and is ongoing (Kent and Landon 2013; Kent et al. 2014; Kent 2015; Kent and Landon 2016; Mathany 2017; Kent 2018).

## Methods

### Well selection

Three different procedures were used to select wells to evaluate status, step trends, and monotonic (time-series) trends of orthophosphate in California groundwater used for public supply.

#### Status well selection

**Fig. 1:**
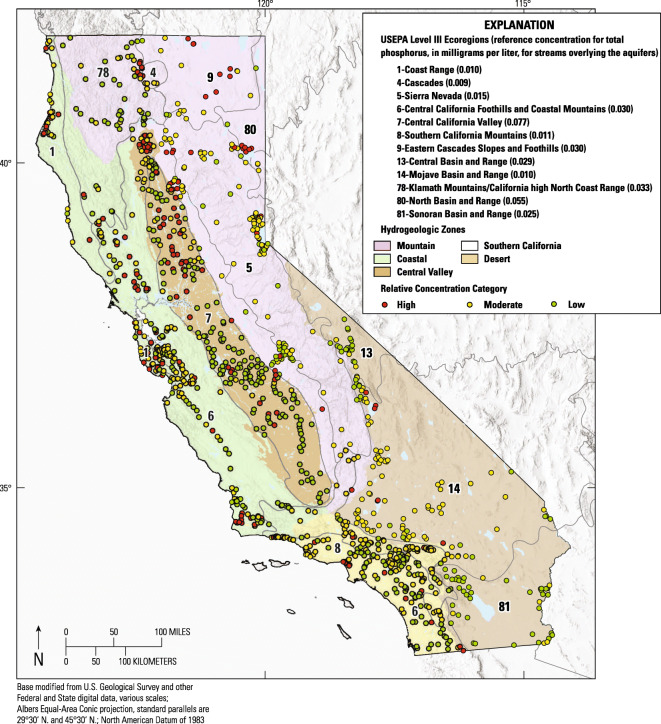
Map of California showing the 1114 public supply wells sampled for the orthophosphate status assessment, the hydrogeologic zones, the numbered USEPA level III ecoregions, and the reference concentrations for total phosphorus in flowing waters overlying the aquifers defined for each ecoregion by the US Environmental Protection Agency. The public supply wells are symbolized by their orthophosphate relative concentration category

The initial sampling of wells for the GAMA-PBP program was designed to provide a spatially unbiased status assessment of the quality of untreated groundwater used for public water supplies in California. Study areas were the fundamental unit of organization for the GAMA-PBP. The GAMA-PBP assessed 87 study areas defined in this manner, which included nearly all the groundwater used statewide for public drinking water supply (Belitz et al. 2015). From 2004 to 2011, the GAMA-PBP collected samples from more than 2000 wells of which 1114 were analyzed for orthophosphate as part of the status assessment (Fig. 1, Online Resources 1 and 2). Details on the selection of wells, grid design, analytical approach, and additional research topics for each study unit can be found in the relevant USGS Reports accessible from the “Publications” link at: http://ca.water.usgs.gov/projects/gama/.

#### Trend well selection for step-trend evaluations

**Fig. 2:**
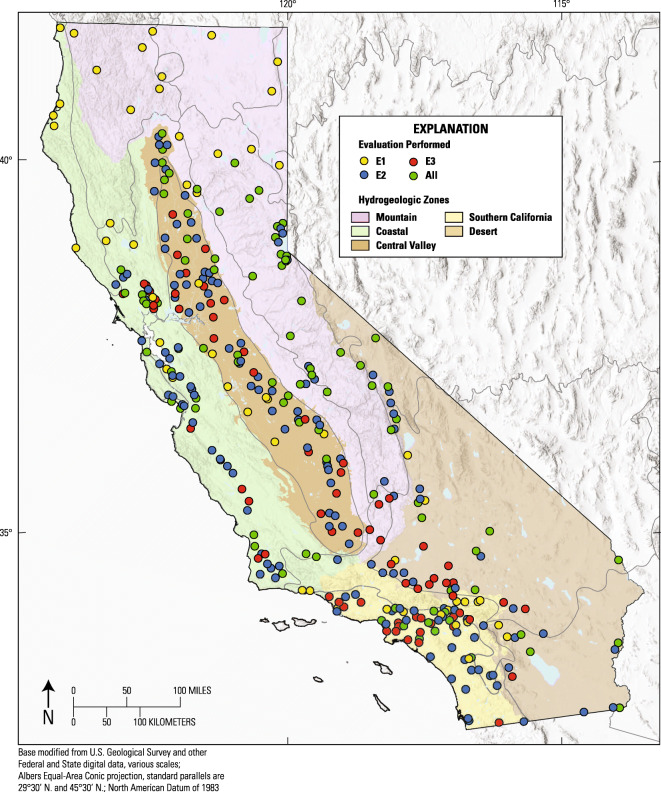
Map of California showing hydrogeologic zones, USEPA level III ecoregions, and the 352 GAMA-PBP wells evaluated for step trends. Yellow symbols are wells evaluated for a step trend between initial and triennial sampling (E1). Blue symbols are wells evaluated between initial and decadal sampling (E2). Red symbols are wells evaluated between triennial and decadal sampling (E3). Green symbols are wells evaluated for step trends between all 3 intervals (E1, E2, and E3)

Approximately 3 and 10 years after their respective initial sampling, a subset of status wells were selected for resampling as “triennial” and “decadal” trend wells (Fig. 2). From 2007 to 2013, the GAMA PBP collected samples from 226 wells to evaluate triennial trends, approximately 10 percent of the status wells in each study area (Online Resource 3). The 226 triennial trend wells represent 34 of the 35 GAMA-PBP study units and 83 of the 87 GAMA-PBP study areas (Kent and Landon 2016). Triennial trend wells were randomly selected from status wells that were still available for sampling in each study area (Kent et al. 2014; Kent 2015; Mathany 2017).

Decadal trend-well sampling began in 2014, continues in 2020, and will be completed in 2021. A revised trend-sampling strategy, which began in 2015, resamples 20 percent of status wells every 5 years. By the end of 2018, 352 wells had been resampled for decadal trends (Online Resource 3). As with triennial trend wells, decadal trend wells are selected randomly from status wells that are still available for sampling in each study area, but with three additional considerations as follows: (1) preference is given to wells that have been sampled as triennial trend wells, (2) preference is given to wells whose initial samples were analyzed for the most complete set of constituents, and (3) decadal trend wells are selected with efforts to provide an approximately even areal distribution of trend wells throughout study areas.

Only wells which have orthophosphate results providing at least one pair-wise comparison among the three sampling intervals (initial, triennial, decadal) could be included in the step-trend evaluations. Therefore, the pair-wise comparisons were approximately 3, 7, or 10 years apart. Note that there were 3 instances in which initially sampled wells had been replaced by new wells in approximately the same location by the time of decadal sampling. Comparisons of major ion and isotope chemistry between samples from the initially sampled and the replacement wells indicated that, in all 3 cases, groundwater quality in the replacement wells was representative of the groundwater quality in the initially sampled wells. Figure 2 shows the locations of the 352 step-trend wells and identifies which pair-wise comparisons (step-trend evaluations) were done on each of them. Site identifications and attributes for these wells are provided in Online Resource 3.

#### Trend well selection for time-series evaluations

The USGS maintains a database of over 75,000 wells and water-quality results in California. These wells include the ones sampled by GAMA-PBP as well as many other projects dating back several decades. Wells for time-series trend evaluation (monotonic trends) were selected from this database (National Water Information System-NWIS-U.S. Geological Survey 2018) based on the availability of at least 8 orthophosphate results for each well, spread over at least 8 years from the year 2000 to 2018. The minimum requirement of 8 results was imposed because the European Water Framework Directive recommends at least 8 measurements when using this mode of trend analysis (Grath et al. 2001). The time period from 2000 to 2018 was selected to approximately coincide with the time period evaluated for step trends (2004 to 2018). Because censored results (those expressed simply as a concentration less than the reporting level) cannot be analytically distinguished, at least 7 of the results for each well needed to be uncensored (detections) for orthophosphate to provide the required 8 distinct results. All censored results were substituted with the value 0.002, which was less than all detected concentrations, so that this value was the lowest ranking result for each time series. When two or, at most, three orthophosphate results per year were available for a well, the mean value for that year was used to give only one result per year. The mean value was used so that each year with orthophosphate data would have equal weight in the time-series evaluations.

An additional requirement was imposed to ensure that all time-series-evaluated wells were of an appropriate depth to represent the groundwater resource used for public supply. Wells that lacked a depth measurement or that were shallower than any of the wells sampled for the GAMA-PBP status assessment of the public-supply resource in the corresponding study unit were excluded from the time-series evaluations. Wells shallower than those sampled for GAMA-PBP status assessment in each area may be unrepresentative of the resource used for public supply. Site identifications and attributes for the 141 wells meeting the requirements for time-series evaluation are provided in Online Resource 4.

### Sample collection and analyses

Groundwater samples for the GAMA-PBP are collected using consistent protocols designed to minimize inadvertent sample contamination (Koterba et al. 1995; U.S. Geological Survey, variously dated). Detailed descriptions of sample collection and analysis methods can be found in USGS GAMA-PBP Data Series Reports accessible from the “Publications” link at: http://ca.water.usgs.gov/projects/gama/. Trend samples were analyzed for a large suite of constituents. Analytical methods for the constituents mentioned in this study, including nutrients (such as orthophosphate), major ions, trace metals, and isotopes are described in Kent et al. (2014), Kent (2015), and Mathany (2017).

### Data evaluation methods

#### Status evaluation of orthophosphate concentrations

The status of orthophosphate in California groundwater used for public supply was evaluated by comparing orthophosphate concentrations in the 1114 status well samples analyzed for orthophosphate during the GAMA-PBP status assessment (Online Resource 2) with two tiers of benchmark concentrations. The benchmarks are based on work done by the US Environmental Protection Agency (USEPA). The first benchmark concentration tier is the level III ecoregion-specific reference concentration for total phosphorus in California flowing waters (U.S. Environmental Protection Agency 2000a, 2000b, 2001) (Fig. 1). California spans twelve level III ecoregions with total phosphorus reference concentrations ranging from 0.009 to 0.077 mg/L. The second benchmark concentration tier is 0.100 mg/L, which is the desired limit for total phosphorus in flowing waters (including groundwater to surface water discharges) not directly discharging to lakes or reservoirs (U.S. Environmental Protection Agency 1986). Note that the use of benchmarks based on total phosphorus includes phosphorus species that may not be readily transported to groundwater.

For the present status assessment, orthophosphate concentrations that are less than the ecoregion-specific reference concentration for flowing water in the ecoregion of the sampled well are considered “low.” Concentrations that are between the ecoregion-specific reference concentration and the second benchmark concentration of 0.100 mg/L as P are considered “moderate.” Concentrations greater than 0.100 mg/L as P are considered “high.” Note that the upper range of ecoregion-specific reference concentration is close to 0.077 mg/L as P, leaving a narrow range (≥ 0.077 and < 0.100) for the designation of moderate conditions to groundwater in the Central Valley, where the upper range is applied. This 3-tiered status assessment method is similar to the method used by the GAMA-PBP to provide context for concentrations of constituents that have health-based thresholds for drinking water (Belitz et al. 2010). Ecological thresholds were used in the present study because there are no health-based thresholds for phosphorus species in drinking water.

#### Cluster analysis

Cluster analysis was performed to identify statistically significant clusters (*p* value ≤ 0.05) of high and low values of orthophosphate using the categories described above. The Hot Spot Analysis tool in Esri’s ArcPro software (Esri Corporation 2019) uses the Getis-Ord Gi* spatial statistic (Ord and Getis 1995) and was run using the 1114 “status” wells with orthophosphate values. The test compares the observed value at a well with its neighbors to determine if the comparison resembles or differs from the mean. The test requires the user to determine a distance at which spatial autocorrelation ceases, indicating that orthophosphate values beyond this distance have no correlation with one another. For this purpose, a semi-variogram plot was created to determine the distance at which the variance plateau occurs. Any well located beyond the determined distance from another well would not be considered part of the same cluster. The False Discovery Rate Correction (FDRC) option was selected when running the tool to mitigate multiple testing and spatial dependency issues (Esri Corporation 2019). The FDRC is a conservative measure, effectively increasing *p* values and reducing the likelihood for the observed values to be statistically significant.

#### Statistical methods for the determination of step trends

Temporal trends in groundwater quality cannot be detected in individual wells by comparing results from just two samples collected over each time interval. In this study, step trends were evaluated by grouping the wells into categories that might be expected to share relatively similar geologic, climatic, and hydrologic characteristics. Belitz et al. (2003) defined 10 hydrogeologic provinces in their framework report which established the design of the GAMA-PBP. For the present study, the study units were grouped into 5 condensed “hydrogeologic zones” as follows: Central Valley, Coastal, Desert, Mountain, and Southern California (Fig. 1). The zones were condensed to increase the number of wells in each group because the ability for a statistical test to detect a difference (when it exists) improves with increasing sample size (Anderson 1987). It should be noted that despite efforts to group the wells into hydrogeologic categories with similar characteristics, variations in geology, climate, and hydrology were large within each hydrogeologic zone.

Hypothesis tests on the grouped differences of paired samples were used to conclude whether step trends in orthophosphate concentrations had occurred in groundwater statewide, and within each of the 5 hydrogeologic zones. Three time intervals were evaluated (Fig. 2). The first evaluation interval (E1) compared initial orthophosphate concentrations with concentrations in samples collected from the same 144 wells approximately 3 years later (triennial sampling). The second evaluation interval (E2) compared initial concentrations in 227 wells with concentrations in samples collected approximately 10 years later (decadal sampling). The third evaluation interval (E3) compared concentrations in samples collected in 159 wells during triennial sampling with concentrations in samples collected during decadal sampling. Therefore, sample pairs for E3 were collected approximately 7 years apart. Comparisons among the evaluation intervals were made on paired samples from the same wells. However, inferences of a step trend are drawn at the statewide scale and for each hydrogeologic zone, and not for individual wells.

Before the hypothesis tests were performed, the data were processed using the “GAMA Replicate Acceptability Criteria” method described by Kent (2018), so that small differences in the paired results, due to analytical limitations, would not support an inference that a step trend had occurred. After processing the data, a Wilcoxon signed-rank test with a modification proposed by Pratt (1959) was performed comparing the paired orthophosphate results (initial and trend sampling results) statewide and within each hydrogeologic zone to determine whether the concentration was increasing or decreasing in a statistically significant way. The Wilcoxon signed-rank test is a nonparametric alternative to a paired *t* test that does not assume that the data have a normal distribution, an assumption often violated with water-quality data (Helsel and Hirsch 2002). The Wilcoxon signed-rank test is used to test whether the median difference between paired observations equals zero (null hypothesis). The absolute values of the differences are ranked, so that the relative magnitudes and the relative number of changes in each direction (increases or decreases) are both taken into consideration. When there is no difference between paired results (ties), the traditional Wilcoxon signed-rank test discards that pair during the ranking step. The Pratt modification ranks the observations, including the tied pair results, and then drops the ties before performing the test. A trend was considered detected at a significance level ≥ 95 percent (*α* = 0.05).

#### Statistical methods for the determination of time-series trends

The nonparametric Mann-Kendall trend test (Mann 1945; Helsel and Hirsch 2002) was used to test for the significance of a Kendall’s τ correlation of orthophosphate concentration and time in the 141 public supply wells that met the requirements described earlier for time-series trend evaluation. The Sen slope estimator was calculated to estimate the trend magnitude (Sen 1968; Hirsch et al. 1991) or rate of change in orthophosphate concentrations (mg/L/year as P). It should be noted that substituting the value of 0.002 for censored data, as described previously, could affect trend slope calculations, especially if the censored result occurred near the beginning or the end of the evaluated period. As with the step-trend evaluations, a time-series trend was considered detected at a significance level ≥ 95 percent (*α* = 0.05). In contrast to the step-trend evaluations, time-series trends were evaluated for individual wells, not for hydrogeologic zones. Based on the results of the trend test, each well was categorized as “decreasing,” “increasing,” or “no trend” in orthophosphate concentrations.

#### Evaluation of potential explanatory factors

Principal component analysis (PCA) was used to look for relationships among groundwater chemistry and attributes of the wells evaluated for this study with orthophosphate concentrations and changes in orthophosphate concentrations in groundwater from those wells. Principal components (PC) show which variables explain the variance in the data. PCs that explain less than 10 percent of the data are generally considered insignificant. Therefore, within these datasets, the first 3 principal components, PC1–PC3, were used to explain the variance in the datasets. Chemical PCA variables included, in addition to orthophosphate concentrations, pH, total dissolved solids, dissolved oxygen, nitrate, magnesium, boron, manganese, fluoride, sulfate, arsenic, uranium, and bicarbonate. Other ions and metals are available and were not included in the PCA analysis because they autocorrelate with those used. For example, sulfate was excluded from the status analysis because it autocorrelates with total dissolved solids, and iron was excluded because it autocorrelates with manganese and there are fewer censored results for manganese than for iron. However, sulfate was included in the step trend analyses because fewer data were available for step trends and changes were observed between steps. Groundwater chemistry data which span the study period are available through the USGS National Water Information System (NWIS) database at https://waterdata.usgs.gov/nwis, by entering the USGS Station IDs provided in Online Resources 2, 3, and 4. Ancillary PCA variables included land use, depth of well below land surface, ranked age of groundwater in the well, septic tank density, and aridity index within a 500-m radius of each well.

Land use data were represented as percentages of the broad categories, agricultural, natural, and urban, in discrete years spanning five decades (1974, 1982, 1992, 2002, and 2012) (Falcone 2015). The land use data from 2002 was used as a median date across the time of orthophosphate sampling and used for the status well PCA. The 19 different “Coding 2012 Land Uses” described by Falcone (2015) were aggregated into the three broad categories used here as follows: codes 43–45 were categorized as agricultural; codes 11, 12, 41, 42, 50, and 60 as natural; and the other ten codes were categorized as urban.  Septic tank density, aridity index, groundwater age, and well depth are static variables in this study. That is, their values were obtained for one moment in time and related to both status and trend data for PCA. Septic tank density was determined from the 1990 Census of Population and Housing (the most recent census that inquired whether a home was on a septic or a sewer system) and expressed as tanks/km^2^ (U.S. Department of Commerce 1992). The aridity index is calculated as the average annual precipitation (PRISM Climate Group 2012) divided by the average annual evapotranspiration (Flint and Flint 2007), and values can range from 0.05 (hyper-arid) to greater than 1.00 (wet). Groundwater ages were static variables based principally on the activities of tritium (Plummer et al. 1993) and carbon-14 (Clark and Fritz 1997) measured during the status assessment, and were presented as the following categories: modern, modern or mixed, mixed, premodern or mixed, and premodern. PCA requires numerical values, so these 5 categories were assigned values of 1 through 5 in the order listed above representing a youngest-to-oldest ranked gradient scale. Springs were included in the status and trends evaluations, and these were assigned a well depth of zero. Ancillary attributes of the wells evaluated in this study are provided in Online Resources 2, 3, and 4.

In total, 20 variables were included in the PCA, 13 chemical variables (see above) and 7 ancillary variables (land use; as percent agricultural, urban, or undeveloped, aridity, depth of well, age rank, and septic tank density). Some data processing was needed before the variables were submitted for PCA analysis. Censored data were processed in a manner similar to what was done before statistical trend evaluations. Censored results in datasets for PCA were set to a single value less than any detected concentration in the dataset. PCA is a nonparametric test and this strategy ensured that censored results shared the lowest ranked value used for each constituent. For PCA trend evaluations (step trends and time series), initial values and rates of change were calculated for each parameter as separate variables. Rates of change were expressed as the average change in milligrams or micrograms per year. For PCA step-trend evaluations, this was simply calculated as

1 $$ \left({P}_{\mathrm{t}}-{P}_{\mathrm{i}}\right)/\mathrm{years} $$

where *P*_i_ is the parameter concentration in the initial sample, *P*_t_ is the parameter concentration in the trend sample, and *years* is the interval length in years. For PCA time-series evaluations, rates of change were calculated by the same Sen slope estimator method used to estimate the magnitude of orthophosphate time-series trends (Sen 1968; Hirsch et al. 1991).

Finally, some wells that were evaluated for status and trends in orthophosphate concentrations lacked some of the additional chemistry and attribute data, and blank entries are not permitted in PCA. For samples lacking field-measured pH or specific conductance, laboratory-measured values were substituted when available. In contrast, most of the alkalinity measurements for GAMA-PBP samples were made at NWQL. But, when these were lacking, field-measured alkalinity measurements were substituted for laboratory measurements when available. However, substituted results were not available for most missing data, and the decision as to how many parameters to include in each PCA was, by necessity, a compromise between including the maximum number of parameters versus including the maximum number of wells. Therefore, PCA was performed for datasets consisting of fewer wells than were evaluated for status and trends in orthophosphate concentrations. PCA was performed for 801 of the 1114 GAMA-PBP status wells that had orthophosphate results (Online Resource 2). PCA variables for the status evaluation consisted of the initial sample measurements for chemical variables, the 2002 land use values, and static values for the other ancillary variables. PCA was performed for 119 of the 144 step-trend wells evaluated in E1, 190 of the 227 wells evaluated for E2, and 139 of the 159 wells evaluated for E3. Chemical and ancillary variables for all PCA trend evaluations were expressed as the slope of their change during the relevant time periods. As with the status PCA evaluation, static values were used for the other ancillary variables for trend-evaluation PCA.

Arsenic, uranium, DO, and groundwater age were not included in PCA for time-series wells because these data were lacking for many of the time-series samples. Aridity was also not included in the time-series analyses because it did not show any predictive value.

Due to the highly variable ranges in concentrations and values among the parameters, all values were normalized using the method of Kramer (1998). Time-series PCA chemical variables were not normalized because all changes over time were analyzed by the slope of the change, and these were all within the same range. However, static explanatory variables (well depth, septic tank density, etc.) were normalized because of the large variations in these parameters. Principal component analyses were conducted using OriginPro 2019b software version 9.6.5.169 (OriginLab® Northampton, MA) add-in module. The add-in uses the same methods as the PCA in the OriginPro software but provides 3D graphical output.

Quality-control samples in the form of blanks and replicates were collected during the three sampling intervals. The results of quality-control samples for orthophosphate and the chemical parameters submitted to PCA were evaluated to determine whether analytical variability or positive bias might have affected the results of trend evaluations or PCA. In addition, GAMA-PBP periodically evaluates field blank results to define study reporting levels that are greater than the laboratory reporting levels (Olsen et al. 2010; Davis et al. 2014). Results from quality-control samples indicate that neither variability nor positive bias had an appreciable effect on trend evaluations or PCA. Field blank samples collected for all three sampling intervals had detection frequencies that were less than 5 percent for all chemical parameters of interest. Replicate results for these parameters were acceptable, by the project criteria described by Kent (2018), with few exceptions.

## Results

### Status of orthophosphate in California groundwater

#### Relative concentrations of orthophosphate

Statewide, orthophosphate was analyzed in samples from 1114 GAMA-PBP status wells (Table 1, Fig. 1, Online Resources 1 and 2). Concentrations in 169 of the initial samples collected from those status wells (15.2 percent) were greater than the 0.100 mg/L as P, defined as “high” by the classification scheme used in this report. Orthophosphate concentrations in samples from 482 wells (43.3 percent) statewide were at levels defined as “moderate” in this report. Orthophosphate concentrations in samples from the remaining 463 status wells (41.6 percent) were at levels defined as “low” in this report (Table 1).

**Table 1:** Area assessed and the relative concentrations (RC) of orthophosphate in samples from status wells evaluated by the GAMA-PBP by hydrogeologic zone

Hydrogeologic zone	Assessed area (km^2^)	Average ecoregion-specific reference concentration for phosphorus in overlying streams (mg/L as P)	Total wells	Median orthophosphate concentration	Number of high RC wells	Percentage of high RC wells	Number of moderate RC wells	Percentage of moderate RC wells	Number of low RC wells	Percentage of low RC wells
Desert	7607	0.017	169	0.017	6	3.6%	93	55.0%	70	41.4%
Mountains	21,707	0.022	257	0.036	44	17.1%	138	53.7%	75	29.2%
Coastal	8952	0.025	260	0.042	53	20.4%	129	49.6%	78	30.0%
Southern California	9803	0.025	177	0.024	12	6.8%	81	45.8%	84	47.5%
Central Valley	39,238	0.069	251	0.048	54	21.5%	41	16.3%	156	62.2%
Statewide	87,307	0.033	1114	0.033	169	15.2%	482	43.3%	463	41.6%

High orthophosphate RC is defined as greater than 0.1 mg/L as P. Moderate RC is defined as between the ecoregion-specific reference concentration for total phosphorus in streams overlying the area where the well is located (USEPA 2000a, b; 2001) and 0.100 mg/L as P, and low RC is defined as less than that ecoregion-specific reference condition

Summary statistics on the relative concentrations of orthophosphate by hydrogeologic zone are presented in Table 1. The zones with the greatest percentages of high relative concentrations were the Central Valley (21.5 percent) and the Coastal (20.4 percent) zones, followed by the Mountain zone (17.1percent). It should be noted, however, that most of the high relative concentrations of orthophosphate in samples from Mountain zone wells were observed in the Cascade Range and Modoc Plateau. In general, moderate relative concentrations were found in groundwater from about half of the wells in each hydrogeologic zone (Table 1, Online Resource 2). The Central Valley zone was the exception. Moderate relative concentrations were found in groundwater from relatively few wells in the Central Valley zone, because the ecoregion-specific reference concentration of 0.077 (Fig. 1), the boundary for moderate relative concentrations, is so close to the high benchmark concentration of 0.100 mg/L as P.

#### Cluster analysis

**Fig. 3:**
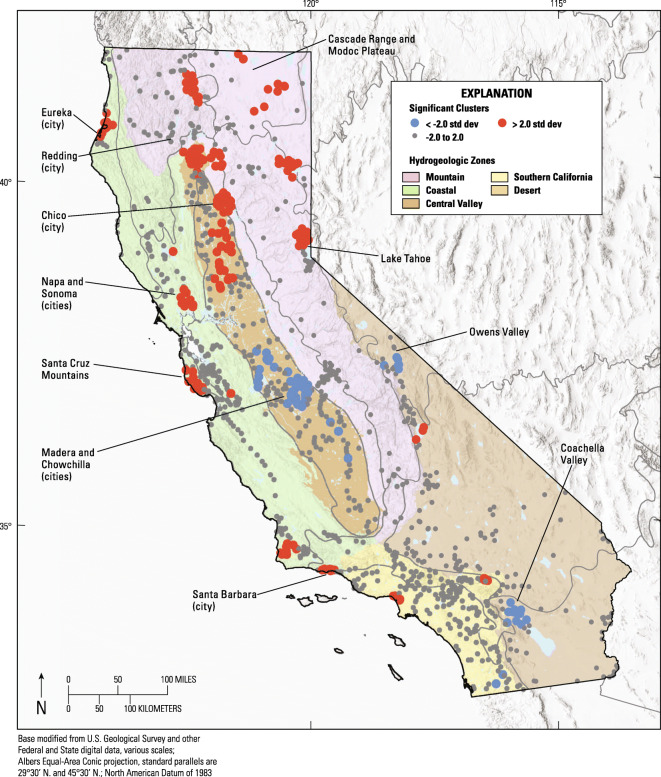
Map of California showing hydrogeologic zones, USEPA level III ecoregions, and significant clusters of status wells with groundwater containing high and low concentrations of orthophosphate. Blue clusters locate significant low orthophosphate concentrations. Red clusters locate significant high orthophosphate concentrations. Gray symbols locate wells and well clusters with groundwater orthophosphate concentrations neither high nor low as defined by cluster analysis. Geographic features identified with leader lines are ponts of reference in the text

Cluster analysis was performed by first creating a semi-variogram of the orthophosphate values, which indicated a plateau of variance at approximately 25 km. Therefore, a search distance of 25 km was used for identifying significant high or low OP clusters. Figure 3 shows the high and low clusters when using *Z*-scores greater than 2 standard deviation (*p* value ≤ 0.046). Significant clusters are seen in the northern half of the Central Valley, portions of the Cascade Range and Modoc Plateau, Santa Cruz mountains, northern Lake Tahoe, as well as in areas near the cities of Eureka, Redding, Chico, Napa/Sonoma, and Santa Barbara. Conversely, clusters of low OP values are seen in the cities of Madera and Chowchilla in the southern half of the Central Valley, as well as portions of the Owens and Coachella Valleys.

**Fig. 4:**
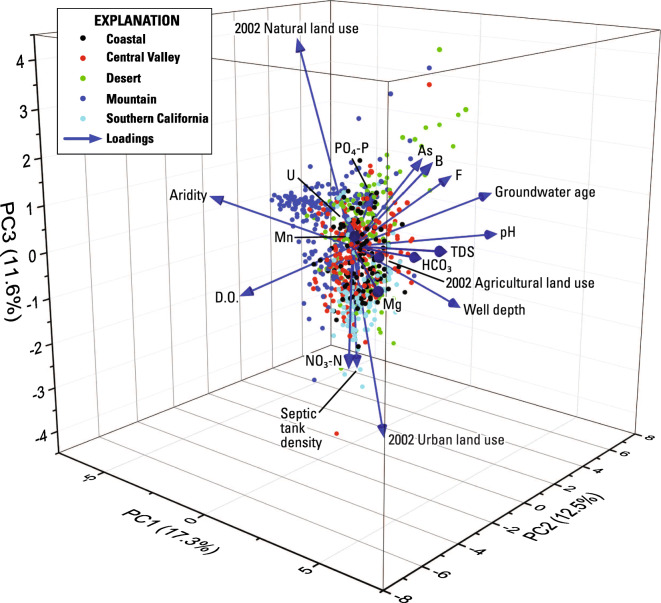
3-dimensional plot of findings from principal component analysis for status wells using normalized values for 12 chemical and 7 non-chemical variables

The first 3 principal components (PC1–3) of 801 status samples explain just over 41 percent of the variance in the data (Fig. 4, Online Resource 5), with PC1 (17.3 percent), PC2 (12.5 percent), and PC3 (11.6 percent) being the only components explaining more than 10 percent of the variance with Eigen values greater than 2. Explaining 41 percent of the variation is somewhat low for the first three PCs, indicating that the dataset is highly variable and that correlations are not well defined by the variables used. However, the loadings shown in Fig. 4 appear to indicate that orthophosphate is most associated with arsenic, boron, and fluoride. No other explanatory factor grouped with orthophosphate, although the DO loading was somewhat antithetical to orthophosphate, suggesting that when DO is low, orthophosphate concentrations are higher. The loading arrows for septic tank density around a well grouped with urban land use and with nitrate concentrations and was also antithetical to orthophosphate (Fig. 4).

**Fig. 5:**
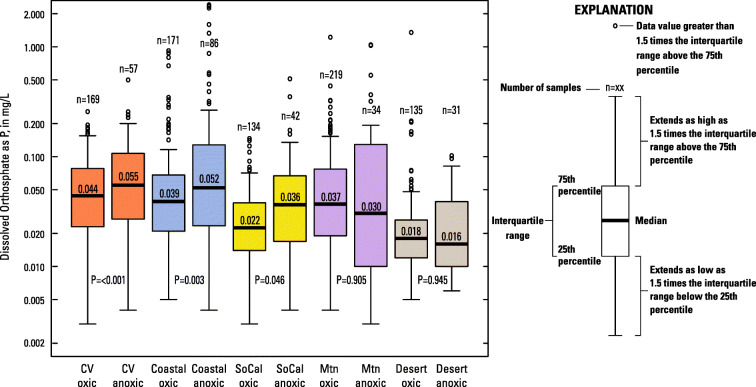
Boxplots of orthophosphate concentrations in status wells by hydrogeologic zone and redox state. Median orthophosphate concentrations, number of samples for each group (*n*), and attained significance of the differences between oxic and anoxic groups (*p*) are labeled. Orthophosphate concentrations were significantly greater in samples from wells with anoxic groundwater than in samples from wells with oxic groundwater (dissolved oxygen > 0.5 mg/L), statewide (*p* = 0.0008), and in 3 of the hydrogeologic zones (Central Valley-CV, Coastal, and Southern California-SoCal). There was no significant difference in orthophosphate concentrations by redox state for the Mountain-Mtn or Desert hydrogeologic zones

Orthophosphate concentrations were related to groundwater redox state. McMahon and Chapelle (2008) defined anoxic groundwater as having a DO concentration less than 0.5 mg/L. Using this criterion, groundwater in 23 percent of the status wells, statewide, was anoxic (Online Resource 2). Orthophosphate concentrations were significantly greater in anoxic groundwater samples compared with oxic samples, statewide, as well as when analysis was done by hydrogeologic zones in the Central Valley, Coastal, and Southern California hydrogeologic zones (Fig. 5). There was no significant difference in orthophosphate concentrations by redox state for the Mountain or Desert hydrogeologic zones.

### Trends in orthophosphate concentrations in California groundwater

Temporal trends in orthophosphate concentrations in California groundwater were evaluated by step-trend and time-series methods. The step-trend method determined trends comparing results among 3 sampling intervals (initial, triennial, and decadal sampling). The time-series method determined trends in groundwater concentrations in individual wells using multiple samples spanning a minimum of 8 years.

#### Step-trend evaluation results

**Fig. 6:**
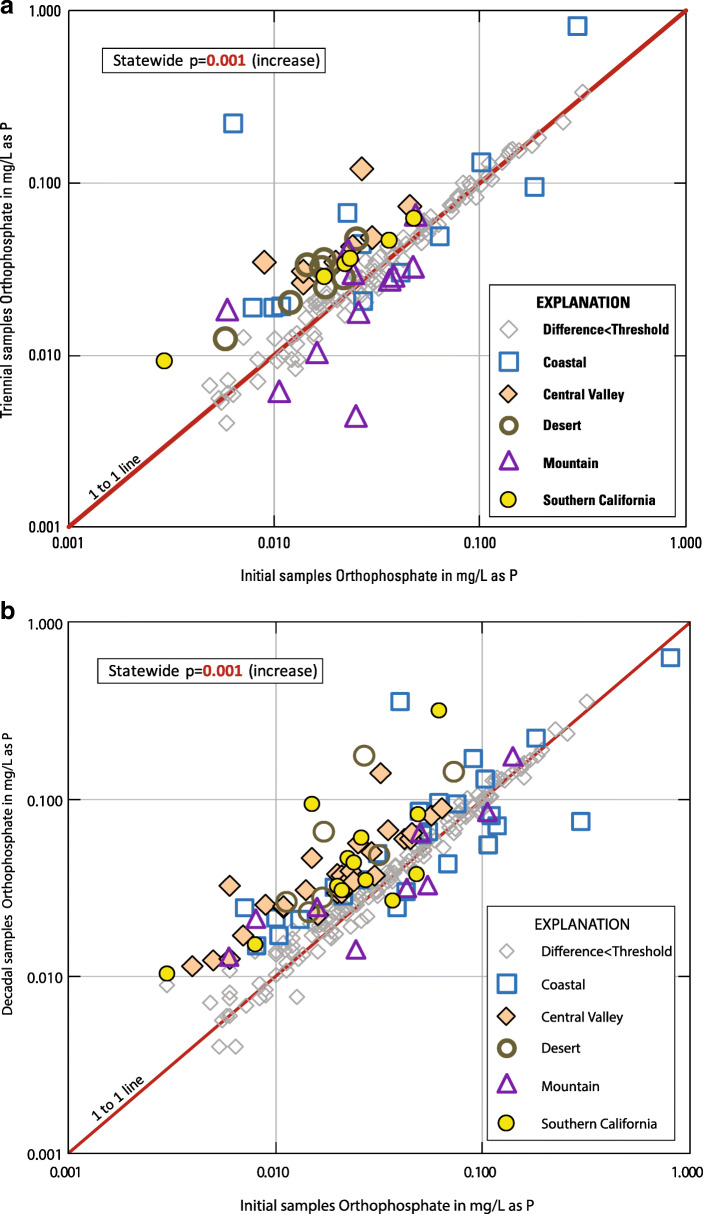

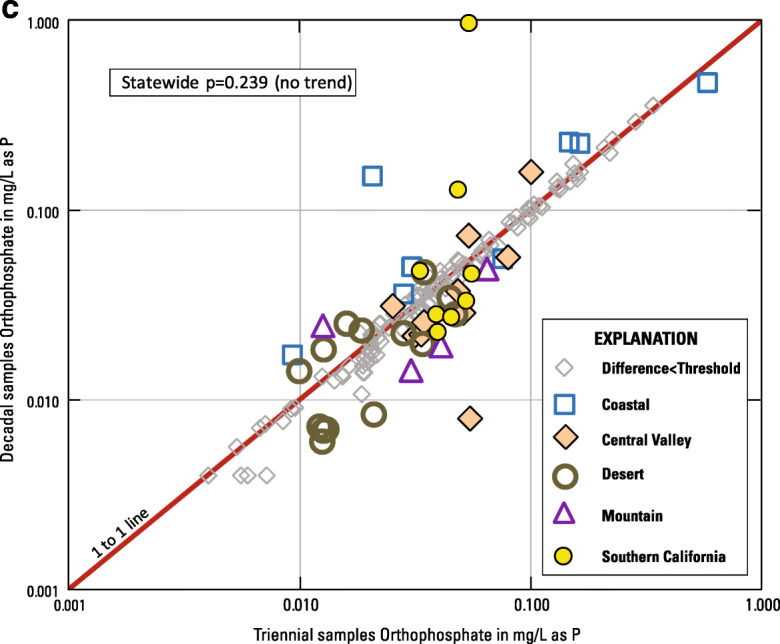
**a** Scatterplot of orthophosphate concentrations, by hydrogeologic zone, measured in initial vs. triennial sampling (E1)**. b** Scatterplot of orthophosphate concentrations, by hydrogeologic zone, measured in initial vs. decadal sampling (E2)**. c** Scatterplot of orthophosphate concentrations, by hydrogeologic zone, measured in triennial vs. decadal sampling (E3)

For E1 (initial compared to triennial sampling results), 144 wells were evaluated for step trends in orthophosphate concentration (Fig. 2, Table 2, Online Resources 1 and 3). Increasing step trends were observed for orthophosphate concentrations statewide and in the Central Valley, Southern California, and Desert hydrogeologic zones (Table 2, Fig. 6a). No step trend was observed in the Mountain nor Coastal zones. These results are similar to those found by Kent and Landon (2016). For E1, the mean rate of increases in orthophosphate concentrations statewide for wells where the changes exceeded the threshold differences was 3.47 × 10^−05^ mg/L/year (Table 2).

**Table 2:** Summary information on Evaluation 1 (E1) step trends in orthophosphate concentrations in California groundwater used for public supply between initial and triennial sampling episodes

Hydrogeologic zone	Wells evaluated for orthophosphate step trend between initial sampling and 3-year sampling (E1)							
	Wells evaluated	Mean step time interval	Wells increasing	Mean rate of increase (mg/L/year as P)	Wells decreasing	Mean rate of decrease (mg/L/year as P)	*p* Value on trend test	Step-trend outcome
Desert	19	3.72	8	9.36E−06	0	na	0.005	Increase
Mountain	46	3.67	4	9.73E−06	8	− 9.68E−06	0.274	No trend
Coastal	32	3.29	8	1.02E−04	4	− 9.68E−06	0.222	No trend
Southern California	20	2.81	6	8.75E−06	0	na	0.015	Increase
Central Valley	27	3.29	8	2.46E−05	0	na	0.005	Increase
Statewide	144	3.40	34	3.47E−05	12	− 1.58E−05	0.001	Increase

Only wells with orthophosphate concentrations that changed by more than the threshold difference were considered “increasing” or “decreasing”

**Fig. 7:**
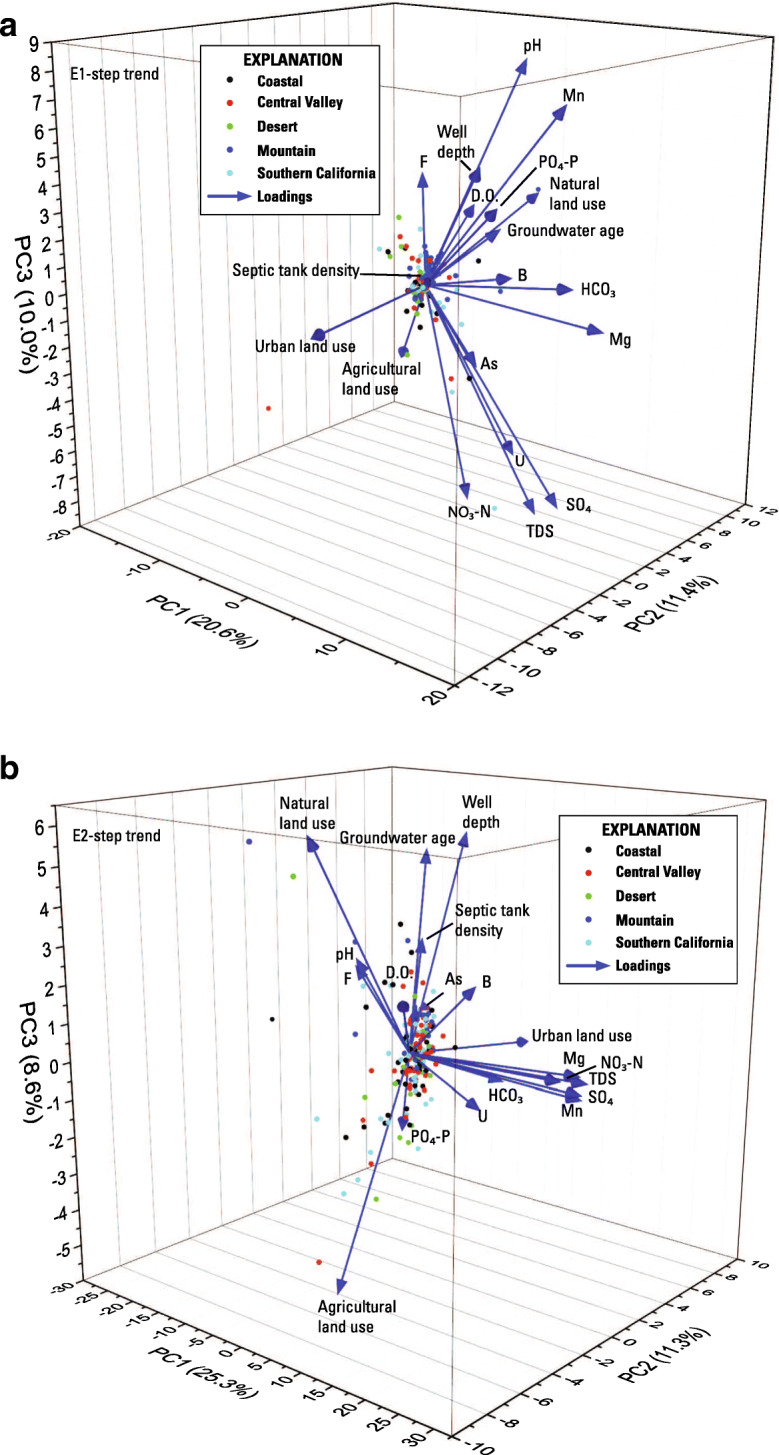

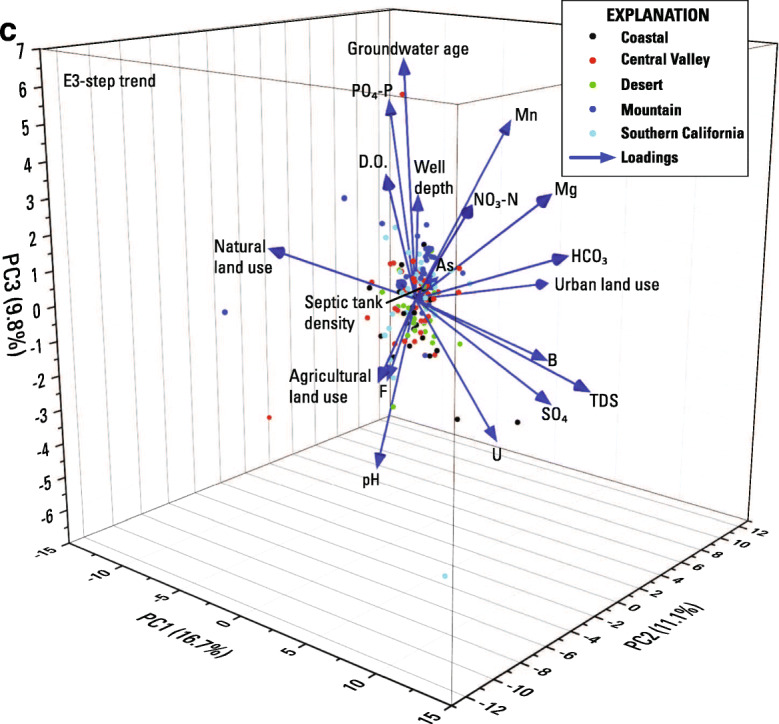
**a** 3-dimensional plot of findings from principal component analysis for E1-initial vs. triennial sampling. All variables were normalized. Chemical and land use variables expressed as the slope of their change. Well depth, aridity, septic tank density, and age rank are static variables**. b** 3-dimensional plot of findings from principal component analysis for E2-initial vs. decadal sampling. All variables were normalized. Chemical and land use variables expressed as the slope of their change. Well depth, aridity, septic tank density, and age rank are static variables**. c** 3-dimensional plot of findings from principal component analysis for E3-triennial vs. decadal sampling. All variables were normalized. Chemical and land use variables expressed as the slope of their change. Well depth, aridity, septic tank density, and age rank are static variables

Principal component analysis was conducted on the entire trend dataset for each step-trend evaluation because there are not enough wells in each hydrologic zone to treat each zone separately. The first 3 principal components (PC1–3) in the E1 PCA evaluation explained 42 percent of the variation (Online Resource 5). PC1 explained 20.6 percent of the variation and PC2 and PC3 explained about 10 percent each. Although well depth, septic tank density, and groundwater age did not vary with time, they were included in all the step analysis PCA to see if a static characteristic might explain a trend in a variable that did vary. Loading scores of PC1 showed that a change in orthophosphate was directly associated with changes in pH, manganese, DO, and in the amount of natural land use near the well. Groundwater age and depth (static variables) of the wells were also associated with this grouping (Fig. 7a). That is, older and deeper groundwater was weakly associated with increasing orthophosphate concentrations. The loading scores for most of these variables were relatively low (< 0.35). In addition, changes occurred in both directions. For example, orthophosphate increased in concentration in 51 wells, and decreased in 67 (one well showed no change), and manganese increased in 68 wells and decreased in 50 wells (one different well showed no change). The same wells increased in both orthophosphate and manganese in 26 wells.

For E2 (initial compared with decadal sampling results), 227 wells were evaluated for step trends (Fig. 2, Table 3, Online Resources 1 and 3). Step-trend patterns for E2 were the same as for E1; increasing step trends were observed statewide and in the Central Valley, Southern California, and Desert hydrogeologic zones (Table 3, Fig. 6b). Again, no step trend was observed in the Mountain nor Coastal zones. For E2, the mean rate of increases in orthophosphate concentrations statewide for wells where the changes exceeded the threshold differences was 8.74 × 10^−06^ mg/L/year.

**Table 3:** Summary information on Evaluation 2 (E2) step trends in orthophosphate concentrations in California groundwater used for public supply between initial and decadal sampling episodes

Hydrogeologic zone	Wells evaluated for orthophosphate step trend between initial sampling and 10-year sampling (E2)							
	Wells evaluated	Mean step time interval	Wells increasing	Mean rate of increase (mg/L/year as P)	Wells decreasing	Mean rate of decrease (mg/L/year as P)	*p* Value on trend test	Step-trend outcome
Desert	37	9.93	7	1.29E−05	0	na	0.008	Increase
Mountain	35	10.02	5	4.40E−06	4	− 4.23E−06	0.772	No trend
Coastal	50	9.77	18	1.14E−05	8	− 1.87E−05	0.114	No trend
Southern California	37	10.02	11	1.23E−05	2	− 2.74E−06	0.011	Increase
Central Valley	68	9.83	29	5.52E−06	0	na	< 0.001	Increase
Statewide	227	9.99	70	8.74E−06	14	− 1.23E−05	< 0.001	Increase

Only wells with orthophosphate concentrations that changed by more than the threshold difference were considered “increasing” or “decreasing”

The first 3 principal components (PC1–3) in the E2 PCA evaluation explained 45.2 percent of the variation. Most of the variation was explained by PC1 (25.3 percent) and PC2 and PC3 both explained less than 12 percent of the variation (Online Resource 5). However, orthophosphate was not significant and had low loading scores in the first three principal components (Fig. 7b).

For E3 (triennial compared to decadal sampling results), 159 wells were evaluated for step trends (Fig. 2, Table 4, Online Resources 1 and 3). In contrast to the findings of the first two step-trend evaluations, no step trends in orthophosphate concentrations were observed for E3 (Table 4, Fig. 6c). The absence of step trends in E3 suggests that the orthophosphate increases observed in E1 and E2 occurred mostly between initial and triennial sampling (2004 to 2013).

**Table 4:** Summary information on Evaluation 3 (E3) step trends in orthophosphate concentrations in California groundwater used for public supply between triennial and decadal sampling episodes

Hydrogeologic zone	Wells evaluated for orthophosphate step trend between 3-year sampling and 10-year sampling (E3)							
	Wells evaluated	Mean step time interval	Wells increasing	Mean rate of increase (mg/L/year as P)	Wells decreasing	Mean rate of decrease (mg/L/year as P)	*p* Value on trend test	Step-trend outcome
Desert	27	6.42	5	3.30E−06	9	− 3.89E−06	0.175	No trend
Mountain	30	5.93	1	6.61E−06	3	− 7.72E−06	0.293	No trend
Coastal	31	6.10	6	2.18E−05	2	− 2.64E−05	0.174	No trend
Southern California	31	7.15	3	1.46E−04	5	− 6.60E−06	0.556	No trend
Central Valley	40	6.11	3	1.27E−05	7	− 8.78713E−06	0.211	No trend
Statewide	159	6.12	18	3.50E−05	26	− 7.91E−06	0.239	No trend

Only wells with orthophosphate concentrations that changed by more than the threshold difference were considered “increasing” or “decreasing”

The first 3 principal components in the E3 PCA evaluation explained 37.6 percent of the variation, which is less than E1 and E2 analyses (Fig. 7c, Online Resource 5). Although orthophosphate has a high loading score for PC3, this principal component explains slightly less than 10 percent of the data. The loading groupings appear to be similar to E1 with groundwater age, well depth, and DO grouping with orthophosphate. However, as mentioned above, PC3 is not particularly significant.

#### Time-series trend evaluation results

**Fig. 8:**
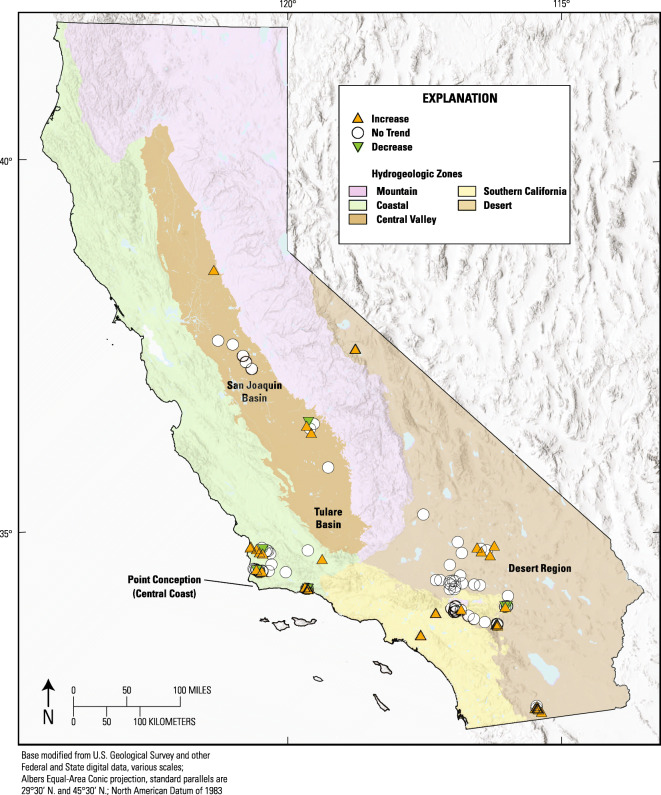
Map of California showing hydrogeologic zones and orthophosphate trend categories (increasing-**∆**, Decreasing-**∇**, or no trend-**○**) of wells evaluated for time-series trends

Time-series evaluations were performed for 141 wells with orthophosphate data meeting the requirements described in the “Methods section” under “Trend well selection for time-series evaluations” (Table 5, Online Resources 1 and 4). Wells in the NWIS database (U.S. Geological Survey 2018) that met these requirements are unevenly distributed in the state and occur in five distinct clusters as follows: the San Joaquin and Tulare Basins (Dubrovsky et al. 1998), the Sacramento River Basin (Domagalski et al. 2001), the Santa Ana Basin (Belitz et al. 2004), the desert region (Dawson and Belitz 2012), and the central coast (Burton et al. 2013; Davis and Kulongoski 2016) (Fig. 8). Time-series evaluations were performed for at least some wells in each of the hydrogeologic zones except for the Mountain zone (Table 5). Some of the wells used in the time-series evaluations are nested, producing groundwater from various depths in the same spatial location.

**Table 5:** Summary information on the results of time-series trend evaluations in orthophosphate concentrations in California groundwater used for public supply

Hydrogeologic zone	Wells evaluated for orthophosphate time-series trends							
	Wells evaluated	Mean time-series length	Wells increasing (number)	Wells increasing (percent)	Mean rate of increase (mg/L/year as P)*	Wells decreasing (number)	Wells decreasing (percent)	Mean rate of decrease (mg/L/year as P)*
Desert	62	15.7	21	34%	1.17E−03	4	6%	− 1.90E−03
Mountain	0	na	na	na	na	na	na	na
Coastal	52	16.1	23	44%	1.92E−03	4	8%	− 4.39E−03
Southern California	15	11.3	3	20%	1.61E−03	0	0%	na
Central Valley	12	12.7	3	25%	2.10E−03	1	8%	− 3.36E−03
Statewide	141	15.1	50	35%	1.60E−03	9	6%	− 3.17E−03

*Change rates calculated only for wells showing a statistically significant increase or decrease

Time-series evaluations found significant increasing trends in orthophosphate concentrations for groundwater from 35 percent of the wells (Table 5). Decreasing trends were found for groundwater from 6 percent of the wells. Wells with increasing trends outnumbered wells with decreasing trends in all hydrogeologic zones where time-series evaluations were performed (Fig. 8, Table 5). However, it should be noted that the mean observed rate at which orthophosphate decreases was about twice the mean observed rate at which it increases (Table 5). It is also interesting to note that rates of change (both increasing and decreasing) found by time-series evaluations (Table 5) were between two and three orders of magnitude greater than rates of change found by step-trend evaluations (Tables 2, 3, and c4). Finally, in most of the wells showing statistically significant increases (30 out of 50), highest orthophosphate concentrations were observed between 2008 and 2011. This period coincides with the interval between initial and triennial sampling during which most of the step-trend increases apparently occurred. Together, these observations suggest that the increasing trend in orthophosphate concentrations occurred early in the study period.

**Fig. 9:**
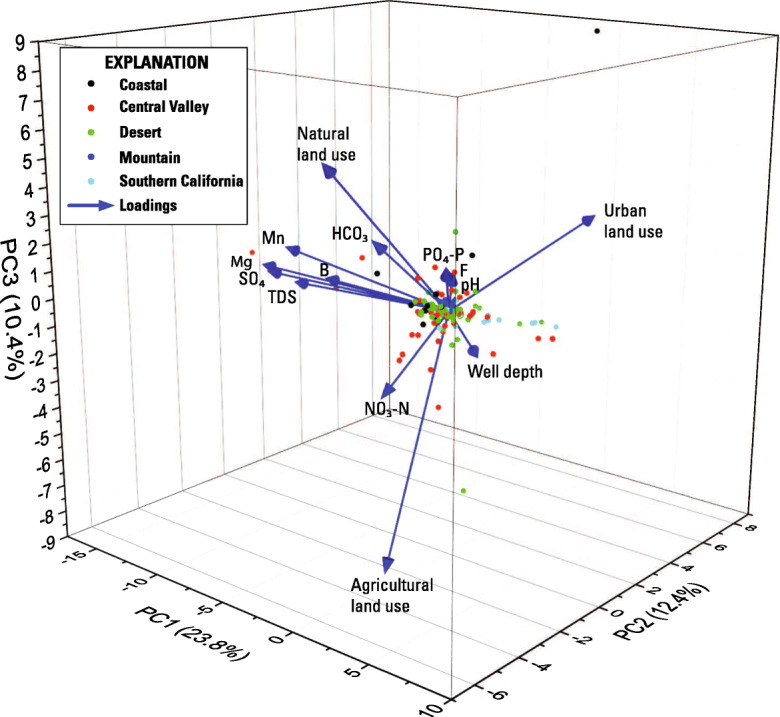
3-dimensional plot of findings from principal component analysis for the time-series evaluation. Variables for time-series PCA were not normalized. Chemical and land use variables are expressed as the slope of their change. Well depth, septic tank density, and age rank are static variables

The first 3 principal components of the time-series data explain 46.6 percent of the data (Fig. 9, Online Resource 5). However, orthophosphate loading is highest for PC3, which explains only about 10 percent of the data. Orthophosphate also does not group with any other variable other than pH, fluoride, and natural land use. Finding direct relationships between changes in orthophosphate and changes in pH and natural land use would be counterintuitive. The solubility of inorganic phosphorus species in water is lower with higher values of pH (Diaz et al. 1994), and it would not be expected that increases in natural land use around a well to be associated with increases in orthophosphate concentrations in groundwater from the well. Fluoride may be introduced into the environment along with phosphate by anthropogenic activities, such as the application of phosphate-containing fertilizers (Saxena and Ahmed 2003), although it is unlikely this would cause a correlation on a statewide basis. However, the loadings are small and the relation between orthophosphate and each of these parameters is weak. It is possible that there are not enough data to fully evaluate time-series trends with PCA. It is also possible that because orthophosphate trends increase and decrease, the PCA may not be able to correlate these changes to other explanatory factors.

## Discussion

The present study found that orthophosphate concentrations in California groundwater used for public supply are at mostly low or moderate concentrations relative to reference conditions for streams overlying the aquifers and the goal expressed by the USEPA for surface water (0.100 mg/L as P) to prevent nutrient enrichment. However, orthophosphate concentrations are high (above that goal) in about 15 percent of the groundwater, statewide, and in more than 20 percent of the groundwater in the Central Valley and Coastal zones of the state.

Cluster analysis indicated that relatively high groundwater orthophosphate concentrations are found specifically in the northern half of the Central Valley, portions of the Cascade Range and Modoc Plateau, Santa Cruz mountains, as well as in areas near the cities of Eureka, Redding, Chico, Napa and Sonoma, Santa Barbara, and Truckee. Relatively low concentrations are found in groundwater near the cities of Madera and Chowchilla in the southern half of the Central Valley, as well as in portions of the Owens and Coachella Valleys.

Present-day groundwater orthophosphate concentrations in the Central Valley hydrogeologic zone may be linked to temporal trends in orthophosphate concentrations found for overlying streams in the recent past. Relatively high groundwater orthophosphate concentrations are prevalent in the northern area of the Central Valley. This is an area where significant upward trends for flow-adjusted orthophosphate concentrations were found for several streams during variable time periods ending in 2004 (Kratzer et al. 2011). In contrast, most groundwater in the southern area of the Central Valley Zone has relatively low concentrations of orthophosphate, where orthophosphate concentrations in streams were mostly decreasing over the same variable time periods (Kratzer et al. 2011). That is, relatively high groundwater orthophosphate in the Central Valley occurs where Kratzer et al. (2011) found surface-water orthophosphate to be increasing before the start of GAMA-PBP, while relatively low groundwater orthophosphate occurs where they found surface-water orthophosphate to be decreasing.

Trend evaluation results suggest that orthophosphate concentrations have increased in approximately one-third of California groundwater used for public supply. Step-trend evaluations comparing grouped-well results from initial sampling with results from triennial sampling were consistent with evaluations comparing results from initial sampling with results from decadal sampling. Those evaluations showed increasing step trends observed statewide and in the Central Valley, Southern California, and Desert hydrogeologic zones. There were no statistically significant step trends for the evaluations comparing results from triennial sampling with results from decadal sampling. This suggests that the orthophosphate concentrations in California groundwater were greatest between initial sampling (2004 to 2011) and triennial sampling (2007 to 2013).

Time-series evaluations and plots show that the timing of increases and the greatest concentrations of orthophosphate are in approximate agreement with step-trend observations. For most wells, the highest concentrations were observed between 2008 and 2011. Also, it appears that groundwater orthophosphate concentrations are still increasing (have not peaked or plateaued) in only 11 of those 50 wells. It should be reiterated that individual wells that met the requirements for time-series evaluation were poorly distributed in California. Nevertheless, time-series evaluation findings mostly confirmed the step-trend evaluation findings that increases in orthophosphate concentrations are more prevalent than decreases, statewide, and for the Central Valley, Southern California, and Desert hydrogeologic zones. In addition, however, and in contrast to the step-trend findings, the time-series evaluation also found many wells showing orthophosphate increases in the Coastal hydrogeologic zone.

It is not clear why the timing of trends in orthophosphate concentrations would be similar throughout California in groundwater with widely varying age distributions and hydrogeologic settings. The state was in a prolonged drought during the study period (Stokstad 2020), but the pattern of relatively wet versus relatively dry years in California between 2000 and 2016 does not explain the timing of the orthophosphate trends.

The relatively high concentrations of orthophosphate prevalent in a few areas of the state are related to low redox conditions as shown by PCA and correlation analysis. A possible explanation for this is that ferric oxides adsorb phosphate, and when these oxides are reduced, phosphate is released (Williams et al. 1976; Drever 1997). The release can be rapid with changing chemical conditions in the aquifer because the phosphate-containing complexes are often adsorbed to sediment surfaces rather than being incorporated in the aquifer material (Kent et al. 2007; Holman et al. 2008). Orthophosphate concentrations were significantly greater in anoxic groundwater samples compared with oxic samples, statewide, as well as in the Central Valley, Coastal, and Southern California hydrogeologic zones. There was no significant difference in orthophosphate concentrations by redox state for the Mountain or Desert hydrogeologic zones.

Explanatory variables other than redox conditions (land use, depth of well, septic tank density, and groundwater age) do not appear to be related to orthophosphate concentrations. PCA analysis shows that orthophosphate loadings are generally low and explain only about 10 percent of the data. Relatively high concentrations of orthophosphate in areas such as the Cascades and Modoc Plateau may be related to the volcanic aquifer materials in the area (Felitsyn 2002; Porder and Ramachandran 2013). However, there is insufficient detailed geologic information for each well to determine if geology is an explanatory factor. Orthophosphate has low mobility because it readily sorbs to aquifer materials (Holman et al. 2008). This low mobility of the orthophosphate ion contrasts with the high mobility of the nitrate ion when it is not being taken up by vegetation (Drever 1997). This may explain why PCA performed for the status evaluation of this study showed that concentrations of nitrate were antithetical to concentrations of orthophosphate.

Groundwater pH has been shown to influence orthophosphate concentrations (Kent et al. 2007; Domagalski and Johnson 2011), because anion sorption is greater at lower pH (Stumm 1992). However, pH did not provide an explanatory factor for OP concentrations or trends in the present study. This is likely because the pH levels observed in the samples collected for this study (5.0 to 9.8 with a median of 7.4) were generally too high to have an effect like the ones observed by the Kent et al. (2007) and Domagalski and Johnson (2011) studies, which included pH values between 4.6 and values slightly above 8.

Given the finding that anoxic groundwater is more likely than oxic groundwater to have higher concentrations of orthophosphate, it might be expected that decreases in DO would be associated with increases in orthophosphate. This study found little evidence of such an inverse correlation between the continuous variables of change in DO and orthophosphate. Confounding expectations, the E1 and E3 PCA evaluations even found a weak direct association between these variables. A possible explanation for this is that release of orthophosphate occurs only when DO concentrations decrease to an anoxic threshold. Manganese may also be released at or below this DO threshold. The strongest direct association found for orthophosphate change by the E1 PCA was with manganese change. The presence of dissolved manganese in groundwater indicates reducing (anoxic) groundwater conditions (Rosecrans et al. 2018).

Principal component analysis of step trends was inconclusive and could not relate available explanatory variables to the binary correlations. This is likely due to several reasons as follows: (1) the changes in orthophosphate concentrations are small compared with other variables. Even with normalization of the data, the small changes are difficult to assess compared with other variables; (2) changes in orthophosphate and other variables are not unidirectional so that complex interactions will reduce the statistical significance of explanatory variables, and (3) the number of trend wells is small for each region, making it difficult to find statistically significant differences by region using multidimensional analysis.

## Conclusions

This study found that orthophosphate concentrations are low in 42 percent of the groundwater used for public supply in California, moderate in 43 percent, and high in 15 percent of this groundwater relative to reference conditions and a goal expressed by the USEPA for streams overlying the aquifers. It should be noted that the California State Water Resources Control Board is currently working on nutrient criteria in water based on biostimulatory response (https://www.waterboards.ca.gov/water_issues/programs/biostimulatory_substances_biointegrity/). The new criteria will likely replace those used here as ecological thresholds in future research. Water managers may use information from the present study to prioritize watersheds for the newly established biostimulatory response monitoring.

The findings also suggest that orthophosphate concentrations increased in about one-third of California groundwater used for public supply during the period from about 2004 to 2011. However, later in the study period, increases were generally not observed. Advancements in wastewater treatment, improvements in agricultural best management practices, and the decline of phosphate detergents in the late twentieth century may have begun to collectively lower the concentrations of orthophosphate in the surface water sources recharging California aquifers.

The baseline conditions and trends described herein for orthophosphate concentrations in California groundwater used for public supply may help to determine whether groundwater discharges to surface water are contributing to eutrophication in surface water bodies in the state. Such information could be more important than ever before due to a recent ruling by the US Supreme Court holding that, under certain circumstances, such discharges may need to be permitted under the Clean Water Act (County of Maui, Hawaii v. Hawaii Wildlife Fund, 2020). Currently (2019) GAMA-PBP is resampling approximately 20 percent of trend wells every 5 years. This sampling strategy will better define temporal trends in California groundwater quality as data accumulate over time.

## Electronic supplementary material

ESM 1 (PDF 169 kb)

ESM 2 (PDF 896 kb)

ESM 3 (PDF 473 kb)

ESM 4 (PDF 286 kb)

ESM 5 (PDF 231 kb)
