# Supplementary material for: Status and trends of orthophosphate concentrations in groundwater used for public supply in California
Source: Environ Monit Assess. 2020 Jul 29;192(8):550. doi: 10.1007/s10661-020-08504-x (PMC7391407; doi:10.1007/s10661-020-08504-x)
Supplement: Supplementary file 1 — (PDF 169 kb) [file 10661_2020_8504_MOESM1_ESM.pdf]

Status and trends of orthophosphate concentrations in groundwater used for public supply in California *Environmental Monitoring and Assessment, Robert Kent, Tyler D. Johnson, and Michael R. Rosen, U.S. Geological Survey California Water Science Center-rhkent@usgs.gov*

Online resource (suppplementary table) 1. Selected attributes of GAMA-PBP (https://ca.water.usgs.gov/gama/) study areas-page 1.

**Hydrogeologic Zone and Study Area-hydrogeologic province (Belitz et al., 2015) in parentheses[1]**	**GAMA-PBP Public Supply Well study unit (chronological sampling sequence number in parentheses)**	**Assessed area (km^2)[2]**	**Number of status (initial) wells**	**Number of status (initial) wells analyzed for orthophosphate**	**Number of triennial wells analyzed for orthophosphate**	**Number of decadal wells analyzed for orthophosphate**
**Desert**						
Coachella Valley (DBR)	Coachella Valley (16)	215	19	19	4	4
Indian Wells Valley (DBR)	Owens and Indian Wells Valleys (14)	666	13	14	2	3
Owens Valley (DBR)		1,486	40	40	4	8
Mojave River Valleys (DBR)	Mojave (23)	1,335	52	19	7	10
Colorado River Valleys (DBR)	Colorado River (21)	487	20	20	3	5
Antelope Valley (DBR)	Antelope Valley (22)	1,379	56	18	6	12
Borrego Valley (DBR)	Borrego Valley, Central Desert, and Low-Use Basins of the Mojave and Sonoran Deserts (28)	176	7	3	1	2
Central Desert basins (DBR)		339	15	9	2	3
Low-use basins of the Mojave and Sonoran Deserts (DBR)		1,523	27	27	3	6
Desert Total		7,607	249	169	32	53
**Mountain**						
Eastside Sacramento Valley (KCM)	Cascade Range and Modoc Plateau (32)	248	15	15	2	0
Honey Lake Valley (KCM)		371	15	15	2	0
Low-use basins of the Cascade Range and Modoc Plateau (KCM)		510	15	15	2	0
Quaternary volcanic areas (KCM)		711	15	15	2	0
Shasta Valley and Shasta Volcanic area (KCM)		320	15	15	2	0
Tertiary volcanic area (KCM)		517	15	15	2	0
Klamath Mountains (KCM)	Klamath Mountains (33)	2,343	38	38	4	0
Southern Sierra Nevada (SNR)	Southern Sierra (11)	1,127	35	7	5	7
Coarse Gold watershed (SNR)	Cental Sierra (10)	458	18	18	2	4
Wishon watershed (SNR)		146	9	9	1	2
Hard Rock Tahoe-Martis (SNR)	Tahoe-Martis (19)	395	13	13	2	3
Martis Valley (SNR)		118	14	14	2	3
Tahoe Valley basins (SNR)		87	14	14	2	4
Sierra Nevada Regional (SNR)[3]	Sierra Nevada (26)	14,116	27	27	16	16
Bear Valley (TSPR)	Bear Valley and Selected Hard Rock Areas (31)	59	14	14	2	0
Hard Rock Lake Arrowhead (TSPR)		183	13	13	2	0
Mountain Total		21,707	285	257	50	39

Status and trends of orthophosphate concentrations in groundwater used for public supply in California *Environmental Monitoring and Assessment, Robert Kent, Tyler D. Johnson, and Michael R. Rosen, U.S. Geological Survey California Water Science Center-rhkent@usgs.gov*

Online resource (suppplementary table) 1. Selected attributes of GAMA-PBP (https://ca.water.usgs.gov/gama/) study areas-page 2.

Hydrogeologic Zone and Study Area-hydrogeologic province (Belitz et al., 2015) in parentheses[1]	Initial sampling period	Triennial sampling period	Decadal sampling period (ongoing in 2019)	Number of wells evaluated for step trend between initial sampling and triennial sampling (E1)	Number of wells evaluated for step trend between initial sampling and decadal sampling (E2)	Number of wells evaluated for step trend between triennial sampling and decadal sampling (E3)	Number of wells evaluated for time-series trends (variable time periods from 2000 to 2018)
**Desert**							
Coachella Valley (DBR)	Feb.-Mar. 2007	Jan. 2011	Feb. 2017	4	4	2	10
Indian Wells Valley (DBR)<br>Owens Valley (DBR)	Sep.-Dec. 2006	Oct. 2010	Dec. 2016	6	11	4	3
Mojave River Valleys (DBR)	Feb.-Apr. 2008	Mar. 2011	Mar. 2018	2	4	7	21
Colorado River Valleys (DBR)	Oct.-Dec. 2007	Jan. 2011	Oct. 2017	3	5	3	0
Antelope Valley (DBR)	Jan.-Mar. 2008	Feb. 2012	Mar.-May 2018	1	5	5	0
Borrego Valley (DBR)<br>Central Desert basins (DBR)<br>Low-use basins of the Mojave and Sonoran Deserts (DBR)	Dec. 2008-Mar. 2010	Oct. 2012	Oct.-Dec 2018	3	8	6	27
Desert Total				19	37	27	61
**Mountain**							
Eastside Sacramento Valley (KCM)<br>Honey Lake Valley (KCM)<br>Low-use basins of the Cascade Range and Modoc Plateau (KCM)<br>Quaternary volcanic areas (KCM)<br>Shasta Valley and Shasta Volcanic area (KCM)<br>Tertiary volcanic area (KCM)	Jul.-Oct. 2010	Jul.-Aug. 2013	planned Summer 2020	12	0	0	0
Klamath Mountains (KCM)	Oct.-Dec. 2010	Oct. 2013	planned Fall 2020	4	0	0	0
Southern Sierra Nevada (SNR)	Jun. 2006	Jun. -Jul. 2008	May. 2016	1	3	5	0
Coarse Gold watershed (SNR)<br>Wishon watershed (SNR)	May 2006	May-Jun. 2010	May. 2016	3	6	3	0
Hard Rock Tahoe-Martis (SNR)<br>Martis Valley (SNR)<br>Tahoe Valley basins (SNR)	Jun.-Sep. 2007	Aug. 2012	Aug.-Sep. 2017	6	10	6	0
Sierra Nevada Regional (SNR)[3]	Jun.-Oct. 2008	Sep.-Oct. 2012	Aug.-Sep. 2018	16	16	16	0
Bear Valley (TSPR)<br>Hard Rock Lake Arrowhead (TSPR)	Apr.-Aug. 2010	Jun. 2013	planned Spring 2020	4	0	0	0
Mountain Total				46	35	30	0

Online resource (suppplementary table) 1. Selected attributes of GAMA-PBP (https://ca.water.usgs.gov/gama/) study areas-page 3.

**Hydrogeologic Zone and Study Area-hydrogeologic province (Belitz et al., 2015) in parentheses[1]**	**GAMA-PBP Public Supply Well study unit (chronological sampling sequence number in parentheses)**	**Assessed area (km^2)[2]**	**Number of status (initial) wells**	**Number of status (initial) wells analyzed for orthophosphate**	**Number of triennial wells analyzed for orthophosphate**	**Number of decadal wells analyzed for orthophosphate**
**Coastal**						
North San Francisco Bay Valley and Plain (NCR)	North San Francisco Bay hydrologic provinces (2)	1,126	50	13	10	15
Sonoma Volcanic Highlands (NCR)		690	20	4	2	6
Wilson Grove Formation Highlands (NCR)		262	14	3	2	4
North Coast coastal basins (NCR)	Northern Coast Ranges (30)	878	30	29	5	0
North Coast inland basins (NCR)		619	28	28	3	0
Monterey Bay area basins (SCR)	Monterey Bay and Salinas Valley Basins (6)	1,185	48	15	5	11
Paso Robles area basin (SCR)		268	11	3	2	3
Salinas Valley basins (SCR)		527	19	7	2	6
Santa Cruz area basins (SCR)		101	13	4	2	5
South San Francisco Bay basins (SCR)	San Francisco Bay (18)	1,012	43	43	5	9
Cuyama Valley (SCR)	South Coast Interior Basins (27)	297	12	12	2	3
Gilroy-Hollister Valley (SCR)		407	17	17	2	4
Livermore Valley (SCR)		157	6	6	1	2
South Coast Coastal alluvial basins (SCR)	South Coast Range (25)	949	39	17	4	8
South Coast Coastal uplands (SCR)		355	16	16	2	4
Santa Barbara area basins (TSPR)	Santa Barbara (34)	119	18	18	2	0
Hard Rock Santa Cruz (SCR)	Santa Cruz, San Gabriel, and Peninsular Ranges Hard Rock Aquifers (35)	1,158	25	25	0	0
Coastal Total		8,952	409	260	51	80

Online resource (suppplementary table) 1. Selected attributes of GAMA-PBP (https://ca.water.usgs.gov/gama/) study areas-page 4.

**Hydrogeologic Zone and Study Area-hydrogeologic province (Belitz et al., 2015) in parentheses[1]**	**Initial sampling period**	**Triennial sampling period**	**Decadal sampling period (ongoing in 2019)**	**Number of wells evaluated for step trend between initial sampling and triennial sampling (E1)**	**Number of wells evaluated for step trend between initial sampling and decadal sampling (E2)**	**Number of wells evaluated for step trend between triennial sampling and decadal sampling (E3)**	**Number of wells evaluated for time-series trends (variable time periods from 2000 to 2018)**
**Coastal**							
North San Francisco Bay Valley and Plain (NCR)<br>Sonoma Volcanic Highlands (NCR)<br>Wilson Grove Formation Highlands (NCR)	Aug.-Nov. 2004	Aug.-Nov. 2007	Sep.-Nov. 2014	7	11	13	0
North Coast coastal basins (NCR)<br>North Coast inland basins (NCR)	Jun.-Oct. 2009	Apr.-May 2012	planned Jun.-Jul. 2019	8	0	0	0
Monterey Bay area basins (SCR)<br>Paso Robles area basin (SCR)<br>Salinas Valley basins (SCR)<br>Santa Cruz area basins (SCR)	Jul.-Sep. 2005	Aug.-Nov. 2008	Aug.-Sep. 2014	2	12	6	0
South San Francisco Bay basins (SCR)	Apr.-Jun. 2007	Mar. 2011	Jun. 2017	5	9	2	0
Cuyama Valley (SCR)<br>Gilroy-Hollister Valley (SCR)<br>Livermore Valley (SCR)	May-Nov. 2008	Jun. 2012	Jul.-Aug. 2018	5	9	5	2
South Coast Coastal alluvial basins (SCR)<br>South Coast Coastal uplands (SCR)	May-Nov. 2008	Jun. 2012	Jun.-Jul. 2018	3	9	5	35
Santa Barbara area basins (TSPR)	Jan.-Feb. 2011	Dec. 2013	planned Winter 2021	2	0	0	16
Hard Rock Santa Cruz (SCR)	Mar.-May. 2011	Not sampled	planned Summer 2021	0	0	0	0
Coastal Total				32	50	31	53

Online resource (suppplementary table) 1. Selected attributes of GAMA-PBP (https://ca.water.usgs.gov/gama/) study areas-page 5.

**Hydrogeologic Zone and Study Area-hydrogeologic province (Belitz et al., 2015) in parentheses[1]**	**GAMA-PBP Public Supply Well study unit (chronological sampling sequence number in parentheses)**	**Assessed area (km^2)[2]**	**Number of status (initial) wells**	**Number of status (initial) wells analyzed for orthophosphate**	**Number of triennial wells analyzed for orthophosphate**	**Number of decadal wells analyzed for orthophosphate**
**Southern California**						
Santa Clara River Valley basins (TSPR)	Santa Clara River Valley (17)	1,093	42	16	6	10
San Fernando Valley (TSPR)	San Fernando-San Gabriel (5)	267	12	4	2	3
San Gabriel Valley (TSPR)		569	23	7	4	5
Central Basin (TSPR)	Coastal Los Angeles Basin (13)	536	21	4	4	4
Orange County Coastal Plain (TSPR)		612	24	3	3	5
Hollywood (TSPR)		41	2	2	0	0
Santa Monica basin (TSPR)		128	4	4	1	1
West Coast basin (TSPR)		252	10	1	1	2
Bunker Hill and Rialto-Colton subbasins (TSPR)	Upper Santa Ana Watershed (15)	483	19	9	5	5
Cucamonga and Chino subbasins (TSPR)		604	25	13	5	5
Riverside-Arlington and Temescal subbasins (TSPR)		285	12	9	2	2
San Jacinto basin (TSPR)		412	21	11	2	4
Yucaipa and San Timoteo subbasins (TSPR)		239	9	3	2	2
Elsinore (TSPR)		104	4	4	0	1
San Diego alluvial basins (SAN)	San Diego Drainages hydrogeologic provinces (1)	380	17	6	0	7
Temecula Valley (SAN)		266	14	8	2	3
Warner Valley (SAN)		68	9	3	1	3
San Diego hard rock (SAN)[4]		3,462	13	4	1	4
Hard Rock Peninsular Ranges (SAN)[4]	Santa Cruz, San Gabriel, and Peninsular Ranges Hard Rock Aquifers (35)	3,462	33	33	0	0
Hard Rock San Gabriel (TSPR)		1,026	33	33	0	0
Southern California Total		9,658	347	177	41	66

Online resource (suppplementary table) 1. Selected attributes of GAMA-PBP (https://ca.water.usgs.gov/gama/) study areas-page 6.

**Hydrogeologic Zone and Study Area-hydrogeologic province (Belitz et al., 2015) in parentheses**[1]	**Initial sampling period**	**Triennial sampling period**	**Decadal sampling period (ongoing in 2019)**	**Number of wells evaluated for step trend between initial sampling and triennial sampling (E1)**	**Number of wells evaluated for step trend between initial sampling and decadal sampling (E2)**	**Number of wells evaluated for step trend between triennial sampling and decadal sampling (E3)**	**Number of wells evaluated for time-series trends (variable time periods from 2000 to 2018)**
**Southern California**							
Santa Clara River Valley basins (TSPR)	Apr.-Jun. 2007	Apr. 2011	Jun.-Aug. 2017	1	3	4	0
San Fernando Valley (TSPR)<br>San Gabriel Valley (TSPR)	May-Jul. 2005	Jun. 2008	Aug. 2015	4	5	4	0
Central Basin (TSPR)<br>Orange County Coastal Plain (TSPR)<br>Hollywood (TSPR)<br>Santa Monica basin (TSPR)<br>West Coast basin (TSPR)	Jun.-Nov. 2006	Aug. 2010	Jul.-Aug. 2016	3	4	10	1
Bunker Hill and Rialto-Colton subbasins (TSPR)<br>Cucamonga and Chino subbasins (TSPR)<br>Riverside-Arlington and Temescal subbasins (TSPR)<br>San Jacinto basin (TSPR)<br>Yucaipa and San Timoteo subbasins (TSPR)<br>Elsinore (TSPR)	Nov. 2006-Mar. 2007	Apr.-May 2009	Jan.-Feb. 2017	10	13	11	14
San Diego alluvial basins (SAN)<br>Temecula Valley (SAN)<br>Warner Valley (SAN)<br>San Diego hard rock (SAN)[4]	May-Jul. 2004	Sept. 2007	Apr.-Aug. 2014	2	12	2	0
Hard Rock Peninsular Ranges (SAN)[4]	Aug.-Nov. 2011	Not sampled	planned Summer 2021	0	0	0	0
Hard Rock San Gabriel (TSPR)	May-Aug. 2011	Not sampled	planned Summer 2021	0	0	0	0
Southern California Total				20	37	31	15

Online resource (suppplementary table) 1. Selected attributes of GAMA-PBP (https://ca.water.usgs.gov/gama/) study areas-page 7.

**Hydrogeologic Zone and Study Area-hydrogeologic province (Belitz et al., 2015) in parentheses[1]**	**GAMA-PBP Public Supply Well study unit (chronological sampling sequence number in parentheses)**	**Assessed area (km^2)[2]**	**Number of status (initial) wells**	**Number of status (initial) wells analyzed for orthophosphate**	**Number of triennial wells analyzed for orthophosphate**	**Number of decadal wells analyzed for orthophosphate**
**Central Valley**						
North American subbasin (SAC)	Southern Sacramento Valley (4)	596	11	5	1	3
Solano subbasin (SAC)		1,417	13	3	1	4
South American subbasin (SAC)		738	12	4	1	3
Southern Sacramento Valley QPc area (SAC)		556	11	6	1	3
Suisun subbasin (SAC)		270	5	2	1	2
Yolo subbasin (SAC)		914	15	8	2	4
Eastern Sacramento Valley subbasins (SAC)	Middle Sacramento Valley (12)	3,702	35	23	3	7
Western Sacramento Valley subbasins (SAC)		3,955	36	22	5	8
Northern Sacramento Valley subbasins (SAC)	Northern Sacramento Valley (20)	489	20	20	2	4
Redding area basin (SAC)		556	23	23	2	5
Cosumnes subbasin (SJV)	Northern San Joaquin Basin (3)	403	10	0	1	2
Eastern San Joaquin subbasin (SJV)		1,962	19	2	2	6
Northern San Joaquin Valley QPc area (SJV)		1,123	11	1	1	3
Tracy subbasin (SJV)		1,270	11	2	1	3
Central-Eastside San Joaquin Valley QPc area (SJV)	Central Eastside San Joaquin Basin (9)	709	9	3	1	2
Merced subbasin (SJV)		1,729	23	8	2	5
Modesto subbasin (SJV)		718	10	3	1	2
Turlock subbasin (SJV)		1,154	16	6	2	4
Kern County subbasin (SJV)	Kern County Subbasin (8)	3,052	47	13	5	10
Kaweah subbasin (SJV)	Southeast San Joaquin Valley (7)	1,713	18	5	2	4
Kings subbasin (SJV)		3,850	39	13	4	8
Tulare Lake subbasin (SJV)		867	9	5	1	2
Tule subbasin (SJV)		1,613	17	5	2	4
Madera and Chowchilla subbasins (SJV)	Madera—Chowchilla (24)	2,161	30	30	4	6
Delta-Mendota subbasin (SJV)	Western San Joaqin Valley (29)	2,719	29	29	3	0
Westside subbasin (SJV)		1,001	10	10	1	0
Central Valley Total		39,238	489	251	52	104
GRAND TOTAL		87,162	1,779	1,114	226	342

Online resource (suppplementary table) 1. Selected attributes of GAMA-PBP (https://ca.water.usgs.gov/gama/) study areas-page 8.

**Hydrogeologic Zone and Study Area-hydrogeologic province (Belitz et al., 2015) in parentheses[1]**	**Initial sampling period**	**Triennial sampling period**	**Decadal sampling period (ongoing in 2019)**	**Number of wells evaluated for step trend between initial sampling and triennial sampling (E1)**	**Number of wells evaluated for step trend between initial sampling and decadal sampling (E2)**	**Number of wells evaluated for step trend between triennial sampling and decadal sampling (E3)**	**Number of wells evaluated for time-series trends (variable time periods from 2000 to 2018)**
**Central Valley**							
North American subbasin (SAC)<br>Solano subbasin (SAC)<br>South American subbasin (SAC)<br>Southern Sacramento Valley QPc area (SAC)<br>Suisun subbasin (SAC)<br>Yolo subbasin (SAC)	Mar.-Jun. 2005	Apr. 2008	Mar.-May. 2014	2	13	5	1
Eastern Sacramento Valley subbasins (SAC)<br>Western Sacramento Valley subbasins (SAC)	Jun.-Sep. 2006	Aug. 2010	Sep.-Oct. 2016	5	12	8	0
Northern Sacramento Valley subbasins (SAC)<br>Redding area basin (SAC)	Oct. 2007-Jan. 2008	Jan. 2011	Oct. 2017-Jan. 2018	4	9	4	0
Cosumnes subbasin (SJV)<br>Eastern San Joaquin subbasin (SJV)<br>Northern San Joaquin Valley QPc area (SJV)<br>Tracy subbasin (SJV)	Dec. 2004-Feb. 2005	Mar.-Apr. 2008	Jan.-Feb. 2014	1	1	5	1
Central-Eastside San Joaquin Valley QPc area (SJV)<br>Merced subbasin (SJV)<br>Modesto subbasin (SJV)<br>Turlock subbasin (SJV)	Mar.-May 2006	Jan. 2010	Feb.-Mar. 2017	3	9	5	4
Kern County subbasin (SJV)	Jan.-Mar. 2006	Feb.-Mar. 2010	Dec. 2015-Apr. 2016	0	5	4	0
Kaweah subbasin (SJV)<br>Kings subbasin (SJV)<br>Tulare Lake subbasin (SJV)<br>Tule subbasin (SJV)	Oct. 2005-Feb. 2006	Nov. 2008	Oct.-Dec. 2015	4	13	7	6
Madera and Chowchilla subbasins (SJV)	Apr.-May 2008	Mar. 2011	Jun. 2018	4	6	2	0
Delta-Mendota subbasin (SJV)<br>Westside subbasin (SJV)	Mar.-Jul. 2010	Apr. 2013	planned Spring 2020	4	0	0	0
Central Valley Total				27	68	40	12
GRAND TOTAL				144	227	159	141

Online resource (suppplementary table) 1. Selected attributes of GAMA-PBP (https://ca.water.usgs.gov/gama/) study areas-page 9.

Footnotes:

[1]Hydrogeologic zones defined for this report. Study areas and hydrogeologic provinces from Belitz et al (2015): KCM, Klamath Mountains--Cascade Range and Modoc Plateau; DBR, Desert--Basin and Range; NCR, Northern Coast Ranges; SAC, Sacramento Valley; SCR, Southern Coast Ranges; SAN, San Diego Drainages; SNR, Sierra Nevada; SJV, San Joaquin Valley; TSRP, Transverse and Selected Peninsular Ranges.

Belitz, K., Fram, M. S., & Johnson T. D. (2015). Metrics for assessing the quality of groundwater used for public supply, CA, USA: Equivalent population and area. *Environmental Science and Technology*, *49* (14), 8330-8338. doi: 10.1021/acs.est.5b00265.

[2]Assessed area for hydrogeologic provinces are a summation of values computed at the scale of study areas.

[3]The Sierra Nevada Regional study unit sampled 30 status wells. However, 3 of these wells had already been sampled to characterize water-quality status in study units located in parts of the Sierra Nevada region. SIERRA-G-02=SOSA-15 in the Southern Sierra study unit, SIERRA-G-07=CWISH-04 in the Central Sierra study unit, SIERRA-V-01=TROCK-05 in the Tahoe-Martis study unit.

[4]San Diego hard rock, as a study area of the San Diego Drainages study unit, and Hard Rock Peninsular Ranges as a study area of the Hard Rock study unit represent the same study area. The Hard Rock study unit had not been sampled trends for trends at the time of preparation of this report.
