# Supplementary material for: Status and trends of orthophosphate concentrations in groundwater used for public supply in California
Source: Environ Monit Assess. 2020 Jul 29;192(8):550. doi: 10.1007/s10661-020-08504-x (PMC7391407; doi:10.1007/s10661-020-08504-x)
Supplement: Supplementary file 2 — (PDF 896 kb) [file 10661_2020_8504_MOESM2_ESM.pdf]

Online resource (suppplementary table) 2. Selected attributes of GAMA-PBP (https://ca.water.usgs.gov/gama/) status wells sampled for orthophosphate concentration-page 1

GAMA-PBP ID	USGS Station ID[1]	GAMA-PBP study unit	GAMA-PBP study area[3]	Hydrogeologic zone
CUY-01	345838119452001	South Coast Interior Basins	Cuyama Valley	Coastal
CUY-02	345603119411901	South Coast Interior Basins	Cuyama Valley	Coastal
CUY-03[2]	345100119290001	South Coast Interior Basins	Cuyama Valley	Coastal
CUY-04	345602119362401	South Coast Interior Basins	Cuyama Valley	Coastal
CUY-05[2]	345600119400001	South Coast Interior Basins	Cuyama Valley	Coastal
CUY-06	345300119310001	South Coast Interior Basins	Cuyama Valley	Coastal
CUY-07	345538119332201	South Coast Interior Basins	Cuyama Valley	Coastal
CUY-08	345405119325101	South Coast Interior Basins	Cuyama Valley	Coastal
CUY-09	345000119020002	South Coast Interior Basins	Cuyama Valley	Coastal
CUY-10	345100119100001	South Coast Interior Basins	Cuyama Valley	Coastal
CUY-11	344143119193001	South Coast Interior Basins	Cuyama Valley	Coastal
CUY-12	345100119290002	South Coast Interior Basins	Cuyama Valley	Coastal
GIL-01	370700121370001	South Coast Interior Basins	Gilroy-Holister Valley	Coastal
GIL-02	370000121330001	South Coast Interior Basins	Gilroy-Holister Valley	Coastal
GIL-03[2]	365200121250001	South Coast Interior Basins	Gilroy-Holister Valley	Coastal
GIL-04	365150121221801	South Coast Interior Basins	Gilroy-Holister Valley	Coastal
GIL-05[2]	365000121310001	South Coast Interior Basins	Gilroy-Holister Valley	Coastal
GIL-06[2]	365600121240001	South Coast Interior Basins	Gilroy-Holister Valley	Coastal
GIL-07[2]	365800121330001	South Coast Interior Basins	Gilroy-Holister Valley	Coastal
GIL-08[2]	370400121350001	South Coast Interior Basins	Gilroy-Holister Valley	Coastal
GIL-09[2]	370300121350001	South Coast Interior Basins	Gilroy-Holister Valley	Coastal
GIL-10	370700121350001	South Coast Interior Basins	Gilroy-Holister Valley	Coastal
GIL-11[2]	370000121310001	South Coast Interior Basins	Gilroy-Holister Valley	Coastal
GIL-12	365200121330001	South Coast Interior Basins	Gilroy-Holister Valley	Coastal
GIL-13	364604121181501	South Coast Interior Basins	Gilroy-Holister Valley	Coastal
GIL-14[2]	365000121250001	South Coast Interior Basins	Gilroy-Holister Valley	Coastal
GIL-15	365500121210001	South Coast Interior Basins	Gilroy-Holister Valley	Coastal
GIL-16	365317121233901	South Coast Interior Basins	Gilroy-Holister Valley	Coastal
GIL-17	365000121230001	South Coast Interior Basins	Gilroy-Holister Valley	Coastal
LIV-01	374000121510001	South Coast Interior Basins	Livermore Valley	Coastal

Online resource (suppplementary table) 2. Selected attributes of GAMA-PBP (https://ca.water.usgs.gov/gama/) status wells sampled for orthophosphate concentration-page 2.

GAMA-PBP ID	USEPA Level III Ecoregions[4]	Level III Ecoregion Reference Concentration[4]	Status Sample Date	Status Sample Orthophosphate Concentration (mg/L as P)	Relative Concentration Category[5]	Redox state[6]	Elevation of LSD (meters above NAVD 88)[7]	Well depth (meters below LSD)[8]	Agricultural land use in 1974[9] (percent)	Natural land use in 1974[9] (percent)	Urban land use in 1974[9] (percent)
CUY-01	Central California Foothills and Coastal Mountains	0.0300	Sep 15 2008	0.023	low	oxic	581	57	0.0%	95.3%	4.7%
CUY-02	Central California Foothills and Coastal Mountains	0.0300	Sep 15 2008	0.018	low	oxic	658	241	0.0%	100.0%	0.0%
CUY-03[2]	Southern California Mountains	0.0109	Sep 16 2008	0.013	moderate	oxic	840	na	64.7%	28.1%	7.2%
CUY-04	Central California Foothills and Coastal Mountains	0.0300	Sep 17 2008	0.013	low	oxic	695	219	46.8%	38.6%	14.5%
CUY-05[2]	Central California Foothills and Coastal Mountains	0.0300	Sep 17 2008	0.010	low	oxic	669	na	29.7%	61.2%	9.1%
CUY-06	Southern California Mountains	0.0109	Sep 17 2008	0.010	low	oxic	778	244	90.1%	9.9%	0.0%
CUY-07	Central California Foothills and Coastal Mountains	0.0300	Sep 18 2008	0.012	low	oxic	724	183	99.1%	0.9%	0.0%
CUY-08	Central California Foothills and Coastal Mountains	0.0300	Sep 18 2008	0.011	low	oxic	751	168	68.9%	31.1%	0.0%
CUY-09	Southern California Mountains	0.0109	Sep 22 2008	0.066	moderate	oxic	1,637	110	6.6%	85.9%	7.5%
CUY-10	Southern California Mountains	0.0109	Sep 23 2008	0.049	moderate	oxic	1,646	91	3.2%	42.3%	54.5%
CUY-11	Southern California Mountains	0.0109	Sep 25 2008	0.018	moderate	oxic	1,122	109	29.7%	67.1%	3.2%
CUY-12	Southern California Mountains	0.0109	Sep 25 2008	0.050	moderate	oxic	836	61	77.6%	15.5%	6.8%
GIL-01	Central California Foothills and Coastal Mountains	0.0300	Aug 11 2008	0.039	moderate	oxic	108	148	35.5%	1.4%	63.1%
GIL-02	Central California Foothills and Coastal Mountains	0.0300	Aug 12 2008	0.037	moderate	oxic	59	143	72.5%	0.0%	27.5%
GIL-03[2]	Central California Foothills and Coastal Mountains	0.0300	Aug 13 2008	0.076	moderate	oxic	83	na	55.8%	30.9%	13.4%
GIL-04	Central California Foothills and Coastal Mountains	0.0300	Aug 13 2008	0.071	moderate	oxic	88	152	58.6%	0.5%	40.9%
GIL-05[2]	Central California Foothills and Coastal Mountains	0.0300	Aug 13 2008	0.049	moderate	oxic	62	na	42.9%	23.0%	34.1%
GIL-06[2]	Central California Foothills and Coastal Mountains	0.0300	Aug 14 2008	0.068	moderate	anoxic	62	na	99.5%	0.0%	0.5%
GIL-07[2]	Central California Foothills and Coastal Mountains	0.0300	Aug 14 2008	0.033	moderate	oxic	56	na	79.1%	10.9%	10.0%
GIL-08[2]	Central California Foothills and Coastal Mountains	0.0300	Aug 14 2008	0.036	moderate	oxic	83	na	61.4%	0.9%	37.7%
GIL-09[2]	Central California Foothills and Coastal Mountains	0.0300	Aug 18 2008	0.036	moderate	oxic	73	na	51.2%	0.9%	47.9%
GIL-10	Central California Foothills and Coastal Mountains	0.0300	Aug 18 2008	0.033	moderate	oxic	110	115	28.9%	14.2%	56.9%
GIL-11[2]	Central California Foothills and Coastal Mountains	0.0300	Aug 19 2008	0.047	moderate	oxic	53	na	72.9%	1.8%	25.3%
GIL-12	Central California Foothills and Coastal Mountains	0.0300	Aug 19 2008	0.064	moderate	anoxic	47	213	40.6%	32.0%	27.4%
GIL-13	Central California Foothills and Coastal Mountains	0.0300	Aug 19 2008	0.027	low	anoxic	153	140	8.2%	80.5%	11.4%
GIL-14[2]	Central California Foothills and Coastal Mountains	0.0300	Aug 20 2008	0.122	high	anoxic	90	na	19.1%	65.9%	15.0%
GIL-15	Central California Foothills and Coastal Mountains	0.0300	Aug 21 2008	0.128	high	anoxic	77	116	94.1%	4.1%	1.8%
GIL-16	Central California Foothills and Coastal Mountains	0.0300	Aug 21 2008	0.056	moderate	oxic	71	103	31.6%	6.5%	61.9%
GIL-17	Central California Foothills and Coastal Mountains	0.0300	Aug 21 2008	0.040	moderate	oxic	97	137	23.0%	2.3%	74.8%
LIV-01	Central California Foothills and Coastal Mountains	0.0300	Aug 25 2008	0.029	low	oxic	109	152	13.2%	10.5%	76.4%

Online resource (suppplementary table) 2. Selected attributes of GAMA-PBP (https://ca.water.usgs.gov/gama/) status wells sampled for orthophosphate concentration-page 3.

GAMA-PBP ID	Agricultural land use in 1982[9] (percent)	Natural land use in 1982[9] (percent)	Urban land use in 1982[9] (percent)	Agricultural land use in 1992[9] (percent)	Natural land use in 1992[9] (percent)	Urban land use in 1992[9] (percent)	Agricultural land use in 2002[9] (percent)	Natural land use in 2002[9] (percent)	Urban land use in 2002[9] (percent)	Agricultural land use in 2012[9] (percent)	Natural land use in 2012[9] (percent)	Urban land use in 2012[9] (percent)	Age Classification[10]	Septic Tanks[11]	Aridity[12]
CUY-01	0.0%	95.3%	4.7%	0.0%	94.8%	5.2%	0.0%	94.8%	5.2%	0.0%	94.8%	5.2%	Mixed	0.08	0.167
CUY-02	0.0%	100.0%	0.0%	0.0%	100.0%	0.0%	0.0%	100.0%	0.0%	0.0%	100.0%	0.0%	Premodern	0.08	0.157
CUY-03[2]	64.3%	28.1%	7.7%	64.3%	28.1%	7.7%	63.8%	28.1%	8.1%	63.8%	28.1%	8.1%	Modern	0.08	0.165
CUY-04	46.8%	38.2%	15.0%	46.8%	38.2%	15.0%	47.3%	37.7%	15.0%	47.3%	37.7%	15.0%	Mixed	0.08	0.138
CUY-05[2]	29.7%	61.2%	9.1%	29.7%	61.2%	9.1%	30.1%	60.7%	9.1%	30.1%	60.7%	9.1%	Premodern	0.08	0.154
CUY-06	98.6%	1.4%	0.0%	98.6%	1.4%	0.0%	93.9%	6.1%	0.0%	93.9%	6.1%	0.0%	Mixed	0.08	0.158
CUY-07	100.0%	0.0%	0.0%	100.0%	0.0%	0.0%	100.0%	0.0%	0.0%	100.0%	0.0%	0.0%	Mixed	0.11	0.148
CUY-08	68.9%	31.1%	0.0%	68.9%	31.1%	0.0%	68.9%	31.1%	0.0%	68.9%	31.1%	0.0%	Mixed	0.08	0.156
CUY-09	15.5%	76.5%	8.0%	6.6%	85.4%	8.0%	22.1%	68.5%	9.4%	22.1%	68.5%	9.4%	Premodern	9.62	0.409
CUY-10	3.2%	32.3%	64.5%	3.2%	26.4%	70.5%	7.7%	21.8%	70.5%	7.7%	21.4%	70.9%	ModernOrMixed	4.84	0.315
CUY-11	32.4%	64.4%	3.2%	31.1%	64.4%	4.6%	30.6%	64.4%	5.0%	30.6%	64.4%	5.0%	Mixed	0.08	0.234
CUY-12	77.6%	15.5%	6.8%	77.6%	15.5%	6.8%	75.8%	16.0%	8.2%	75.8%	16.0%	8.2%	Mixed	0.08	0.164
GIL-01	22.6%	0.9%	76.5%	15.7%	0.9%	83.4%	7.4%	0.5%	92.2%	7.4%	0.5%	92.2%	Mixed	17.20	0.413
GIL-02	70.2%	0.0%	29.8%	66.5%	0.0%	33.5%	65.6%	0.0%	34.4%	46.8%	0.0%	53.2%	Mixed	6.84	0.413
GIL-03[2]	57.6%	29.0%	13.4%	56.7%	30.0%	13.4%	55.8%	30.9%	13.4%	55.8%	30.9%	13.4%	Modern	6.51	0.293
GIL-04	58.6%	0.5%	40.9%	58.6%	0.5%	40.9%	58.2%	0.5%	41.4%	57.7%	0.0%	42.3%	Premodern	13.43	0.306
GIL-05[2]	49.3%	17.1%	33.6%	47.5%	17.5%	35.0%	42.9%	21.2%	35.9%	42.9%	20.3%	36.9%	Modern	7.02	0.367
GIL-06[2]	99.5%	0.0%	0.5%	99.5%	0.0%	0.5%	99.5%	0.0%	0.5%	99.5%	0.0%	0.5%	Modern	2.13	0.399
GIL-07[2]	78.6%	10.0%	11.4%	78.6%	9.1%	12.3%	78.6%	9.1%	12.3%	78.6%	0.5%	20.9%	Modern	3.71	0.411
GIL-08[2]	56.4%	0.9%	42.7%	52.7%	0.9%	46.4%	48.6%	0.9%	50.5%	48.6%	0.5%	50.9%	Modern	35.26	0.409
GIL-09[2]	47.5%	0.9%	51.6%	39.2%	0.9%	59.9%	30.9%	0.9%	68.2%	30.9%	0.0%	69.1%	Modern	18.47	0.412
GIL-10	26.1%	12.8%	61.0%	24.8%	12.4%	62.8%	21.1%	12.4%	66.5%	21.1%	11.0%	67.9%	Mixed	15.25	0.420
GIL-11[2]	72.9%	1.8%	25.3%	72.4%	1.8%	25.8%	72.4%	1.8%	25.8%	72.4%	1.8%	25.8%	Modern	7.93	0.410
GIL-12	40.6%	31.5%	27.9%	40.6%	30.6%	28.8%	38.4%	29.2%	32.4%	38.4%	27.4%	34.2%	Premodern	7.48	0.426
GIL-13	13.2%	75.5%	11.4%	13.2%	75.5%	11.4%	11.4%	77.3%	11.4%	11.4%	77.3%	11.4%	Modern	0.97	0.311
GIL-14[2]	22.7%	62.3%	15.0%	20.5%	64.5%	15.0%	19.1%	63.2%	17.7%	19.1%	60.9%	20.0%	Premodern	10.58	0.288
GIL-15	95.0%	3.2%	1.8%	93.6%	3.2%	3.2%	93.6%	3.2%	3.2%	93.6%	3.2%	3.2%	Premodern	2.02	0.390
GIL-16	17.7%	3.7%	78.6%	10.7%	3.7%	85.6%	9.8%	3.7%	86.5%	9.8%	2.8%	87.4%	Premodern	13.14	0.329
GIL-17	17.1%	2.3%	80.6%	5.9%	2.3%	91.9%	0.0%	2.3%	97.7%	0.0%	0.0%	100.0%	Mixed	31.24	0.287
LIV-01	9.1%	10.5%	80.5%	6.4%	9.5%	84.1%	1.8%	12.3%	85.9%	1.4%	10.9%	87.7%	Modern	2.89	0.340

Online resource (suppplementary table) 2. Selected attributes of GAMA-PBP (https://ca.water.usgs.gov/gama/) status wells sampled for orthophosphate concentration-page 4

GAMA-PBP ID	USGS Station ID[1]	GAMA-PBP study unit	GAMA-PBP study area[3]	Hydrogeologic zone
LIV-02	373840121532901	South Coast Interior Basins	Livermore Valley	Coastal
LIV-03	374000121520001	South Coast Interior Basins	Livermore Valley	Coastal
LIV-05	374200121480001	South Coast Interior Basins	Livermore Valley	Coastal
LIV-06[2]	374000121440001	South Coast Interior Basins	Livermore Valley	Coastal
LIVU-03	374100121520001	South Coast Interior Basins	Livermore Valley	Coastal
SCRC-B05[2]	345417120232101	South Coast Range	South Coast alluvial basins	Coastal
SCRC-B08	343600120110001	South Coast Range	South Coast alluvial basins	Coastal
SCRC-B10	344428120161201	South Coast Range	South Coast alluvial basins	Coastal
SCRC-B11	343939120265302	South Coast Range	South Coast alluvial basins	Coastal
SCRC-B16[2]	345600120250001	South Coast Range	South Coast alluvial basins	Coastal
SCRC-B21	343948120321301	South Coast Range	South Coast alluvial basins	Coastal
SCRC-B22	345712120321701	South Coast Range	South Coast alluvial basins	Coastal
SCRC-B23	350200120320001	South Coast Range	South Coast alluvial basins	Coastal
SCRC-B26	344054120335101	South Coast Range	South Coast alluvial basins	Coastal
SCRC-B27	350400120580001	South Coast Range	South Coast alluvial basins	Coastal
SCRC-B28	350100120290001	South Coast Range	South Coast alluvial basins	Coastal
SCRC-B29[2]	350200120280001	South Coast Range	South Coast alluvial basins	Coastal
SCRC-B30	345900120570001	South Coast Range	South Coast alluvial basins	Coastal
SCRC-B34	344200120270001	South Coast Range	South Coast alluvial basins	Coastal
SCRC-B36	350000120320001	South Coast Range	South Coast alluvial basins	Coastal
SCRC-B37	344021120294502	South Coast Range	South Coast alluvial basins	Coastal
SCRC-B39	345921120381601	South Coast Range	South Coast alluvial basins	Coastal
SCRC-H01	350800120360001	South Coast Range	South Coast Coastal uplands	Coastal
SCRC-H02	344420120041701	South Coast Range	South Coast Coastal uplands	Coastal
SCRC-H03	343800120110001	South Coast Range	South Coast Coastal uplands	Coastal
SCRC-H04	343900120080001	South Coast Range	South Coast Coastal uplands	Coastal
SCRC-H05	344100120030001	South Coast Range	South Coast Coastal uplands	Coastal
SCRC-H06	344800120260001	South Coast Range	South Coast Coastal uplands	Coastal
SCRC-H07	344623120272001	South Coast Range	South Coast Coastal uplands	Coastal
SCRC-H08	343900120040001	South Coast Range	South Coast Coastal uplands	Coastal

Online resource (suppplementary table) 2. Selected attributes of GAMA-PBP (https://ca.water.usgs.gov/gama/) status wells sampled for orthophosphate concentration-page 5.

GAMA-PBP ID	USEPA Level III Ecoregions[4]	Level III Ecoregion Reference Concentration[4]	Status Sample Date	Status Sample Orthophosphate Concentration (mg/L as P)	Relative Concentration Category[5]	Redox state[6]	Elevation of LSD (meters above NAVD 88)[7]	Well depth (meters below LSD)[8]	Agricultural land use in 1974[9] (percent)	Natural land use in 1974[9] (percent)	Urban land use in 1974[9] (percent)
LIV-02	Central California Foothills and Coastal Mountains	0.0300	Aug 25 2008	0.033	moderate	anoxic	96	34	11.0%	19.6%	69.4%
LIV-03	Central California Foothills and Coastal Mountains	0.0300	Aug 25 2008	0.031	moderate	oxic	106	198	2.8%	0.0%	97.2%
LIV-05	Central California Foothills and Coastal Mountains	0.0300	Oct 28 2008	0.050	moderate	oxic	127	91	20.5%	1.4%	78.2%
LIV-06[2]	Central California Foothills and Coastal Mountains	0.0300	Oct 28 2008	0.084	moderate	oxic	175	na	27.1%	0.0%	72.9%
LIVU-03	Central California Foothills and Coastal Mountains	0.0300	Aug 27 2008	0.072	moderate	oxic	103	227	26.5%	0.0%	73.5%
SCRC-B05[2]	Central California Foothills and Coastal Mountains	0.0300	May 22 2008	0.025	low	na	130	198	4.6%	87.5%	7.9%
SCRC-B08	Central California Foothills and Coastal Mountains	0.0300	Jun 4 2008	0.068	moderate	oxic	108	271	7.0%	0.0%	93.0%
SCRC-B10	Central California Foothills and Coastal Mountains	0.0300	Jun 11 2008	0.800	high	oxic	182	149	40.0%	9.5%	50.5%
SCRC-B11	Central California Foothills and Coastal Mountains	0.0300	Jun 12 2008	0.296	high	oxic	25	61	22.7%	0.0%	77.3%
SCRC-B16[2]	Central California Foothills and Coastal Mountains	0.0300	Jun 19 2008	0.019	low	na	70	113	35.6%	0.0%	64.4%
SCRC-B21	Central California Foothills and Coastal Mountains	0.0300	Jun 25 2008	0.177	high	anoxic	12	58	95.5%	2.3%	2.3%
SCRC-B22	Central California Foothills and Coastal Mountains	0.0300	Jun 25 2008	0.023	low	oxic	36	107	100.0%	0.0%	0.0%
SCRC-B23	Central California Foothills and Coastal Mountains	0.0300	Jul 7 2008	0.042	moderate	oxic	80	189	15.5%	82.6%	1.9%
SCRC-B26	Central California Foothills and Coastal Mountains	0.0300	Jul 10 2008	0.391	high	anoxic	8	55	42.1%	57.9%	0.0%
SCRC-B27	Central California Foothills and Coastal Mountains	0.0300	Jul 14 2008	0.050	moderate	oxic	38	128	0.5%	42.4%	57.1%
SCRC-B28	Central California Foothills and Coastal Mountains	0.0300	Jul 15 2008	0.177	high	oxic	113	119	0.5%	7.3%	92.3%
SCRC-B29[2]	Central California Foothills and Coastal Mountains	0.0300	Jul 15 2008	0.028	low	anoxic	94	69	53.2%	4.1%	42.7%
SCRC-B30	Central California Foothills and Coastal Mountains	0.0300	Jul 16 2008	0.025	low	oxic	27	127	91.7%	3.7%	4.6%
SCRC-B34	Central California Foothills and Coastal Mountains	0.0300	Jul 28 2008	0.124	high	anoxic	111	215	0.0%	0.0%	100.0%
SCRC-B36	Central California Foothills and Coastal Mountains	0.0300	Aug 4 2008	0.025	low	oxic	32	61	98.6%	1.4%	0.0%
SCRC-B37	Central California Foothills and Coastal Mountains	0.0300	Aug 7 2008	0.104	high	anoxic	23	71	49.8%	21.0%	29.2%
SCRC-B39	Central California Foothills and Coastal Mountains	0.0300	Nov 20 2008	0.015	low	oxic	22	187	0.0%	100.0%	0.0%
SCRC-H01	Central California Foothills and Coastal Mountains	0.0300	May 19 2008	0.048	moderate	anoxic	38	140	0.0%	66.5%	33.5%
SCRC-H02	Central California Foothills and Coastal Mountains	0.0300	Jun 2 2008	0.193	high	oxic	533	73	0.0%	99.5%	0.5%
SCRC-H03	Central California Foothills and Coastal Mountains	0.0300	Jun 3 2008	0.921	high	oxic	195	168	0.0%	98.2%	1.8%
SCRC-H04	Central California Foothills and Coastal Mountains	0.0300	Jun 3 2008	0.032	moderate	anoxic	323	189	4.2%	93.5%	2.3%
SCRC-H05	Central California Foothills and Coastal Mountains	0.0300	Jun 5 2008	0.023	low	oxic	366	219	0.0%	98.2%	1.8%
SCRC-H06	Central California Foothills and Coastal Mountains	0.0300	Jun 9 2008	0.877	high	oxic	154	105	88.1%	11.4%	0.5%
SCRC-H07	Central California Foothills and Coastal Mountains	0.0300	Jun 9 2008	0.199	high	oxic	105	119	8.2%	91.8%	0.0%
SCRC-H08	Central California Foothills and Coastal Mountains	0.0300	Jun 10 2008	0.025	low	oxic	292	280	0.0%	97.3%	2.7%

Status and trends of orthophosphate concentrations in groundwater used for public supply in California *Environmental Monitoring and Assessment, Robert Kent, Tyler D. Johnson, and Michael R. Rosen, U.S. Geological Survey California Water Science Center-rhkent@usgs.gov*

Online resource (suppplementary table) 2. Selected attributes of GAMA-PBP (https://ca.water.usgs.gov/gama/) status wells sampled for orthophosphate concentration-page 6.

GAMA-PBP ID	Agricultural land use in 1982[9] (percent)	Natural land use in 1982[9] (percent)	Urban land use in 1982[9] (percent)	Agricultural land use in 1992[9] (percent)	Natural land use in 1992[9] (percent)	Urban land use in 1992[9] (percent)	Agricultural land use in 2002[9] (percent)	Natural land use in 2002[9] (percent)	Urban land use in 2002[9] (percent)	Agricultural land use in 2012[9] (percent)	Natural land use in 2012[9] (percent)	Urban land use in 2012[9] (percent)	Age Classification[10]	Septic Tanks[11]	Aridity[12]
LIV-02	11.0%	19.6%	69.4%	10.5%	19.6%	69.9%	4.6%	19.6%	75.8%	4.6%	19.6%	75.8%	Mixed	0.34	0.408
LIV-03	0.5%	0.0%	99.5%	0.0%	0.0%	100.0%	0.0%	0.0%	100.0%	0.0%	0.0%	100.0%	Mixed	1.32	0.361
LIV-05	15.9%	1.4%	82.7%	7.7%	1.4%	90.9%	0.0%	1.4%	98.6%	0.0%	0.9%	99.1%	Mixed	4.05	0.307
LIV-06[2]	27.1%	0.0%	72.9%	26.6%	0.0%	73.4%	25.7%	0.0%	74.3%	18.2%	0.0%	81.8%	Modern	8.05	0.318
LIVU-03	5.9%	0.0%	94.1%	0.0%	0.0%	100.0%	0.0%	0.0%	100.0%	0.0%	0.0%	100.0%	Mixed	0.70	0.370
SCRC-B05[2]	4.6%	87.5%	7.9%	4.6%	87.5%	7.9%	7.9%	84.3%	7.9%	7.9%	83.8%	8.3%	Mixed	4.24	0.307
SCRC-B08	1.9%	0.0%	98.1%	0.0%	0.0%	100.0%	0.0%	0.0%	100.0%	0.0%	0.0%	100.0%	Premodern	9.90	0.319
SCRC-B10	33.2%	9.1%	57.7%	31.8%	8.6%	59.5%	31.4%	8.2%	60.5%	31.4%	7.3%	61.4%	Mixed	0.44	0.322
SCRC-B11	7.3%	0.0%	92.7%	3.6%	0.0%	96.4%	2.3%	0.0%	97.7%	2.3%	0.0%	97.7%	Modern	1.43	0.303
SCRC-B16[2]	21.5%	0.0%	78.5%	5.5%	0.0%	94.5%	5.5%	0.0%	94.5%	5.5%	0.0%	94.5%	Modern	0.43	0.294
SCRC-B21	95.9%	1.8%	2.3%	94.1%	1.8%	4.1%	94.5%	0.5%	5.0%	94.5%	0.5%	5.0%	Mixed	0.57	0.283
SCRC-B22	100.0%	0.0%	0.0%	100.0%	0.0%	0.0%	100.0%	0.0%	0.0%	100.0%	0.0%	0.0%	Modern	0.61	0.303
SCRC-B23	20.7%	77.5%	1.9%	16.4%	80.8%	2.8%	16.0%	79.3%	4.7%	13.6%	80.3%	6.1%	Premodern	5.67	0.334
SCRC-B26	42.1%	57.9%	0.0%	42.1%	57.9%	0.0%	45.4%	53.7%	0.9%	45.4%	53.7%	0.9%	Mixed	0.09	0.284
SCRC-B27	1.4%	41.5%	57.1%	1.4%	41.5%	57.1%	1.4%	41.5%	57.1%	1.4%	41.0%	57.6%	Premodern	5.67	0.333
SCRC-B28	1.4%	7.3%	91.4%	1.4%	6.8%	91.8%	1.4%	6.8%	91.8%	0.0%	6.8%	93.2%	Mixed	70.27	0.334
SCRC-B29[2]	47.2%	0.0%	52.8%	42.2%	0.0%	57.8%	41.7%	0.0%	58.3%	41.7%	0.0%	58.3%	ModernOrMixed	32.12	0.341
SCRC-B30	90.4%	2.8%	6.9%	89.0%	1.4%	9.6%	85.3%	0.5%	14.2%	85.3%	0.0%	14.7%	Modern	0.84	0.323
SCRC-B34	0.0%	0.0%	100.0%	0.0%	0.0%	100.0%	0.0%	0.0%	100.0%	0.0%	0.0%	100.0%	Premodern	0.00	0.319
SCRC-B36	100.0%	0.0%	0.0%	100.0%	0.0%	0.0%	100.0%	0.0%	0.0%	100.0%	0.0%	0.0%	Modern	3.36	0.325
SCRC-B37	51.6%	20.1%	28.3%	51.6%	19.6%	28.8%	56.2%	18.3%	25.6%	56.2%	18.3%	25.6%	Mixed	0.19	0.285
SCRC-B39	0.0%	100.0%	0.0%	0.0%	100.0%	0.0%	0.0%	100.0%	0.0%	0.0%	100.0%	0.0%	Premodern	0.84	0.323
SCRC-H01	0.0%	33.5%	66.5%	0.0%	33.5%	66.5%	0.0%	32.6%	67.4%	0.0%	0.5%	99.5%	Premodern	3.87	0.333
SCRC-H02	0.0%	99.5%	0.5%	0.0%	99.5%	0.5%	0.0%	99.5%	0.5%	0.0%	99.5%	0.5%	Modern	1.39	0.443
SCRC-H03	0.0%	98.2%	1.8%	0.0%	98.2%	1.8%	13.2%	85.0%	1.8%	13.2%	58.6%	28.2%	Mixed	2.03	0.319
SCRC-H04	4.2%	93.5%	2.3%	4.2%	93.5%	2.3%	6.5%	90.3%	3.2%	6.5%	90.3%	3.2%	Premodern	0.53	0.362
SCRC-H05	0.0%	98.2%	1.8%	0.0%	98.2%	1.8%	0.0%	97.7%	2.3%	0.0%	97.3%	2.7%	Premodern	1.39	0.426
SCRC-H06	88.1%	11.4%	0.5%	88.1%	11.4%	0.5%	89.5%	9.6%	0.9%	89.5%	9.6%	0.9%	Mixed	0.63	0.336
SCRC-H07	8.2%	91.8%	0.0%	8.2%	91.8%	0.0%	8.2%	91.8%	0.0%	8.2%	91.8%	0.0%	Mixed	0.17	0.311
SCRC-H08	0.0%	97.3%	2.7%	0.0%	96.8%	3.2%	0.0%	95.4%	4.6%	0.0%	95.4%	4.6%	Premodern	1.39	0.383

GAMA-PBP ID	USGS Station ID[1]	GAMA-PBP study unit	GAMA-PBP study area[3]	Hydrogeologic zone
SCRC-H09	344200120080001	South Coast Range	South Coast Coastal uplands	Coastal
SCRC-H10	344600120080001	South Coast Range	South Coast Coastal uplands	Coastal
SCRC-H11	351100120330001	South Coast Range	South Coast Coastal uplands	Coastal
SCRC-H12	344400120130001	South Coast Range	South Coast Coastal uplands	Coastal
SCRC-H13	344700120190001	South Coast Range	South Coast Coastal uplands	Coastal
SCRC-H14	351200120380001	South Coast Range	South Coast Coastal uplands	Coastal
SCRC-H15	344500120070001	South Coast Range	South Coast Coastal uplands	Coastal
SCRC-H16[2]	343800119550001	South Coast Range	South Coast Coastal uplands	Coastal
NOCO-CO-01[2]	392830123474501	Northern Coast Ranges	North Coast coastal basins	Coastal
NOCO-CO-02[2]	385852123420501	Northern Coast Ranges	North Coast coastal basins	Coastal
NOCO-CO-03[2]	404436124115701	Northern Coast Ranges	North Coast coastal basins	Coastal
NOCO-CO-04[2]	403825124123301	Northern Coast Ranges	North Coast coastal basins	Coastal
NOCO-CO-05[2]	392418123481701	Northern Coast Ranges	North Coast coastal basins	Coastal
NOCO-CO-06[2]	391757123473101	Northern Coast Ranges	North Coast coastal basins	Coastal
NOCO-CO-07[2]	410919124075701	Northern Coast Ranges	North Coast coastal basins	Coastal
NOCO-CO-08[2]	405411124052401	Northern Coast Ranges	North Coast coastal basins	Coastal
NOCO-CO-09[2]	404842124063901	Northern Coast Ranges	North Coast coastal basins	Coastal
NOCO-CO-10[2]	405150123582701	Northern Coast Ranges	North Coast coastal basins	Coastal
NOCO-CO-11[2]	403416124084901	Northern Coast Ranges	North Coast coastal basins	Coastal
NOCO-CO-12[2]	404421124104101	Northern Coast Ranges	North Coast coastal basins	Coastal
NOCO-CO-13[2]	410203124062501	Northern Coast Ranges	North Coast coastal basins	Coastal
NOCO-CO-15[2]	413430124040001	Northern Coast Ranges	North Coast coastal basins	Coastal
NOCO-CO-16[2]	414911124060301	Northern Coast Ranges	North Coast coastal basins	Coastal
NOCO-CO-17[2]	403233124040801	Northern Coast Ranges	North Coast coastal basins	Coastal
NOCO-CO-18[2]	403614124104201	Northern Coast Ranges	North Coast coastal basins	Coastal
NOCO-CO-19[2]	403544124131501	Northern Coast Ranges	North Coast coastal basins	Coastal
NOCO-CO-20[2]	403421124155801	Northern Coast Ranges	North Coast coastal basins	Coastal
NOCO-CO-21[2]	403515124150301	Northern Coast Ranges	North Coast coastal basins	Coastal
NOCO-CO-22[2]	415332124104201	Northern Coast Ranges	North Coast coastal basins	Coastal
NOCO-CO-23[2]	415522124084301	Northern Coast Ranges	North Coast coastal basins	Coastal

Online resource (suppplementary table) 2. Selected attributes of GAMA-PBP (https://ca.water.usgs.gov/gama/) status wells sampled for orthophosphate concentration-page 8.

GAMA-PBP ID	USEPA Level III Ecoregions[4]	Level III Ecoregion Reference Concentration[4]	Status Sample Date	Status Sample Orthophosphate Concentration (mg/L as P)	Relative Concentration Category[5]	Redox state[6]	Elevation of LSD (meters above NAVD 88)[7]	Well depth (meters below LSD)[8]	Agricultural land use in 1974[9] (percent)	Natural land use in 1974[9] (percent)	Urban land use in 1974[9] (percent)
SCRC-H09	Central California Foothills and Coastal Mountains	0.0300	Jun 16 2008	0.021	low	oxic	310	229	76.0%	22.6%	1.4%
SCRC-H10	Central California Foothills and Coastal Mountains	0.0300	Jun 24 2008	0.021	low	oxic	403	183	1.4%	97.3%	1.4%
SCRC-H11	Central California Foothills and Coastal Mountains	0.0300	Jun 26 2008	0.028	low	oxic	98	79	1.4%	96.8%	1.8%
SCRC-H12	Central California Foothills and Coastal Mountains	0.0300	Jul 8 2008	0.030	low	oxic	223	197	0.9%	98.2%	0.9%
SCRC-H13	Central California Foothills and Coastal Mountains	0.0300	Jul 23 2008	0.083	moderate	oxic	255	299	0.0%	99.5%	0.5%
SCRC-H14	Central California Foothills and Coastal Mountains	0.0300	Jul 29 2008	0.046	moderate	oxic	77	91	0.0%	5.0%	95.0%
SCRC-H15	Central California Foothills and Coastal Mountains	0.0300	Aug 5 2008	0.035	moderate	oxic	431	385	23.4%	76.6%	0.0%
SCRC-H16[2]	Central California Foothills and Coastal Mountains	0.0300	Nov 18 2008	0.029	low	na	304	85	0.0%	98.6%	1.4%
NOCO-CO-01[2]	Coast Range	0.0103	Jul 29 2009	0.079	moderate	anoxic	22	5	0.0%	6.5%	93.5%
NOCO-CO-02[2]	Coast Range	0.0103	Jul 30 2009	0.074	moderate	oxic	15	17	0.0%	99.5%	0.5%
NOCO-CO-03[2]	Coast Range	0.0103	Aug 3 2009	0.056	moderate	oxic	12	116	32.9%	2.7%	64.4%
NOCO-CO-04[2]	Coast Range	0.0103	Aug 4 2009	1.596	high	anoxic	74	99	1.4%	79.7%	18.9%
NOCO-CO-05[2]	Coast Range	0.0103	Aug 5 2009	0.009	low	oxic	38	5	0.0%	1.4%	98.6%
NOCO-CO-06[2]	Coast Range	0.0103	Aug 6 2009	0.011	moderate	oxic	29	11	0.0%	88.2%	11.8%
NOCO-CO-07[2]	Coast Range	0.0103	Aug 10 2009	0.049	moderate	oxic	38	40	0.0%	83.0%	17.0%
NOCO-CO-08[2]	Coast Range	0.0103	Aug 11 2009	0.027	moderate	anoxic	10	66	38.2%	0.0%	61.8%
NOCO-CO-09[2]	Coast Range	0.0103	Aug 12 2009	0.579	high	anoxic	2	121	45.7%	19.2%	35.2%
NOCO-CO-10[2]	Coast Range	0.0103	Aug 13 2009	0.192	high	oxic	35	69	22.9%	75.8%	1.3%
NOCO-CO-11[2]	Coast Range	0.0103	Aug 17 2009	0.013	moderate	oxic	15	35	32.4%	10.3%	57.3%
NOCO-CO-12[2]	Coast Range	0.0103	Aug 18 2009	0.071	moderate	oxic	17	19	55.3%	2.8%	41.9%
NOCO-CO-13[2]	Coast Range	0.0103	Aug 19 2009	0.013	moderate	oxic	97	30	0.0%	5.5%	94.5%
NOCO-CO-15[2]	Coast Range	0.0103	Aug 25 2009	0.012	moderate	oxic	8	26	0.0%	83.2%	16.8%
NOCO-CO-16[2]	Coast Range	0.0103	Aug 26 2009	0.017	moderate	oxic	17	23	0.0%	96.8%	3.2%
NOCO-CO-17[2]	Coast Range	0.0103	Aug 27 2009	0.006	low	oxic	45	15	69.4%	25.9%	4.6%
NOCO-CO-18[2]	Coast Range	0.0103	Aug 31 2009	0.006	low	anoxic	12	20	4.6%	64.4%	31.1%
NOCO-CO-19[2]	Coast Range	0.0103	Sep 1 2009	0.008	low	anoxic	11	15	96.3%	2.3%	1.4%
NOCO-CO-20[2]	Coast Range	0.0103	Sep 2 2009	0.037	moderate	oxic	28	0	0.5%	32.3%	67.3%
NOCO-CO-21[2]	Coast Range	0.0103	Sep 3 2009	0.870	high	anoxic	19	51	86.8%	0.0%	13.2%
NOCO-CO-22[2]	Coast Range	0.0103	Sep 14 2009	0.044	moderate	oxic	4	11	90.5%	8.6%	0.9%
NOCO-CO-23[2]	Coast Range	0.0103	Sep 15 2009	0.013	moderate	oxic	12	14	54.3%	5.9%	39.7%

Status and trends of orthophosphate concentrations in groundwater used for public supply in California *Environmental Monitoring and Assessment, Robert Kent, Tyler D. Johnson, and Michael R. Rosen, U.S. Geological Survey California Water Science Center-rhkent@usgs.gov*

Online resource (suppplementary table) 2. Selected attributes of GAMA-PBP (https://ca.water.usgs.gov/gama/) status wells sampled for orthophosphate concentration-page 9.

GAMA-PBP ID	Agricultural land use in 1982[9] (percent)	Natural land use in 1982[9] (percent)	Urban land use in 1982[9] (percent)	Agricultural land use in 1992[9] (percent)	Natural land use in 1992[9] (percent)	Urban land use in 1992[9] (percent)	Agricultural land use in 2002[9] (percent)	Natural land use in 2002[9] (percent)	Urban land use in 2002[9] (percent)	Agricultural land use in 2012[9] (percent)	Natural land use in 2012[9] (percent)	Urban land use in 2012[9] (percent)	Age Classification[10]	Septic Tanks[11]	Aridity[12]
SCRC-H09	76.0%	22.6%	1.4%	76.5%	22.1%	1.4%	77.0%	21.7%	1.4%	77.0%	21.7%	1.4%	Mixed	0.49	0.380
SCRC-H10	1.4%	97.3%	1.4%	1.4%	97.3%	1.4%	1.4%	97.3%	1.4%	1.4%	97.3%	1.4%	Premodern	0.49	0.421
SCRC-H11	21.3%	76.9%	1.8%	21.3%	54.8%	24.0%	21.3%	53.8%	24.9%	22.6%	53.8%	23.5%	Mixed	7.35	0.370
SCRC-H12	0.9%	98.2%	0.9%	0.9%	98.2%	0.9%	8.2%	90.9%	0.9%	8.2%	90.9%	0.9%	Premodern	0.49	0.324
SCRC-H13	0.0%	99.5%	0.5%	0.0%	99.5%	0.5%	25.8%	73.7%	0.5%	38.7%	60.8%	0.5%	Premodern	0.63	0.353
SCRC-H14	0.0%	5.0%	95.0%	0.0%	5.0%	95.0%	0.0%	5.0%	95.0%	0.0%	5.0%	95.0%	Premodern	6.35	0.366
SCRC-H15	23.4%	76.6%	0.0%	23.4%	76.6%	0.0%	23.4%	76.6%	0.0%	23.4%	76.6%	0.0%	Premodern	0.49	0.425
SCRC-H16[2]	0.0%	98.6%	1.4%	0.0%	98.6%	1.4%	0.0%	98.6%	1.4%	0.0%	98.6%	1.4%	Mixed	0.08	0.465
NOCO-CO-01[2]	0.0%	6.5%	93.5%	0.0%	6.5%	93.5%	0.0%	6.5%	93.5%	0.0%	6.5%	93.5%	na	21.42	0.929
NOCO-CO-02[2]	0.0%	99.5%	0.5%	0.0%	99.5%	0.5%	0.0%	99.5%	0.5%	0.0%	99.5%	0.5%	Modern	0.36	0.855
NOCO-CO-03[2]	33.3%	2.7%	63.9%	31.5%	2.7%	65.8%	31.5%	2.7%	65.8%	31.5%	2.7%	65.8%	Premodern	7.60	1.193
NOCO-CO-04[2]	24.0%	57.1%	18.9%	24.0%	56.7%	19.4%	24.0%	56.7%	19.4%	24.0%	56.2%	19.8%	Premodern	3.20	1.275
NOCO-CO-05[2]	0.0%	1.4%	98.6%	0.0%	1.4%	98.6%	0.0%	1.4%	98.6%	0.0%	1.4%	98.6%	Modern	39.27	0.991
NOCO-CO-06[2]	0.0%	87.7%	12.3%	0.0%	87.7%	12.3%	0.0%	87.7%	12.3%	0.0%	87.3%	12.7%	Modern	7.72	1.011
NOCO-CO-07[2]	0.0%	83.0%	17.0%	0.0%	80.7%	19.3%	0.0%	80.3%	19.7%	0.0%	80.3%	19.7%	Modern	1.23	1.542
NOCO-CO-08[2]	37.3%	0.0%	62.7%	35.9%	0.0%	64.1%	34.5%	0.0%	65.5%	34.5%	0.0%	65.5%	Mixed	31.08	1.290
NOCO-CO-09[2]	42.0%	19.2%	38.8%	41.1%	19.2%	39.7%	41.1%	19.2%	39.7%	41.1%	17.4%	41.6%	Premodern	7.83	na
NOCO-CO-10[2]	24.2%	60.1%	15.7%	24.2%	60.1%	15.7%	24.2%	60.1%	15.7%	24.2%	60.1%	15.7%	Mixed	3.47	1.487
NOCO-CO-11[2]	27.7%	9.4%	62.9%	25.4%	9.4%	65.3%	23.9%	9.4%	66.7%	23.9%	8.9%	67.1%	Modern	8.14	1.231
NOCO-CO-12[2]	60.0%	0.0%	40.0%	60.0%	0.0%	40.0%	60.5%	0.0%	39.5%	60.5%	0.0%	39.5%	Mixed	20.26	1.249
NOCO-CO-13[2]	0.0%	5.5%	94.5%	0.0%	5.5%	94.5%	0.0%	5.5%	94.5%	0.0%	5.5%	94.5%	Modern	23.12	1.477
NOCO-CO-15[2]	0.0%	83.2%	16.8%	0.0%	83.2%	16.8%	0.0%	83.2%	16.8%	0.0%	83.2%	16.8%	Modern	0.63	2.150
NOCO-CO-16[2]	0.0%	96.8%	3.2%	0.0%	96.8%	3.2%	19.6%	75.8%	4.6%	19.6%	75.8%	4.6%	Modern	0.40	2.173
NOCO-CO-17[2]	72.7%	19.4%	7.9%	68.1%	22.7%	9.3%	66.2%	23.6%	10.2%	66.2%	22.7%	11.1%	Modern	17.32	1.282
NOCO-CO-18[2]	4.6%	64.4%	31.1%	1.8%	67.1%	31.1%	1.8%	67.1%	31.1%	1.8%	67.1%	31.1%	Mixed	9.55	1.260
NOCO-CO-19[2]	96.3%	2.3%	1.4%	96.3%	2.3%	1.4%	95.4%	2.3%	2.3%	95.4%	2.3%	2.3%	Modern	2.12	1.172
NOCO-CO-20[2]	0.5%	32.3%	67.3%	0.5%	31.8%	67.7%	0.0%	31.8%	68.2%	0.0%	31.8%	68.2%	Mixed	1.73	1.239
NOCO-CO-21[2]	86.8%	0.0%	13.2%	86.8%	0.0%	13.2%	86.8%	0.0%	13.2%	86.8%	0.0%	13.2%	Mixed	1.98	1.176
NOCO-CO-22[2]	90.5%	8.6%	0.9%	90.5%	8.6%	0.9%	91.8%	7.3%	0.9%	90.9%	7.3%	1.8%	Modern	5.85	1.866
NOCO-CO-23[2]	54.3%	5.9%	39.7%	54.3%	5.9%	39.7%	54.3%	5.9%	39.7%	54.3%	5.9%	39.7%	Modern	24.90	1.996

Online resource (suppplementary table) 2. Selected attributes of GAMA-PBP (https://ca.water.usgs.gov/gama/) status wells sampled for orthophosphate concentration-page 10

GAMA-PBP ID	USGS Station ID[1]	GAMA-PBP study unit	GAMA-PBP study area[3]	Hydrogeologic zone
NOCO-CO-24[2]	414911124124701	Northern Coast Ranges	North Coast coastal basins	Coastal
NOCO-CO-25[2]	404943124050502	Northern Coast Ranges	North Coast coastal basins	Coastal
NOCO-CO-26[2]	410420124083301	Northern Coast Ranges	North Coast coastal basins	Coastal
NOCO-CO-27[2]	404120124122601	Northern Coast Ranges	North Coast coastal basins	Coastal
NOCO-CO-28[2]	405330124082501	Northern Coast Ranges	North Coast coastal basins	Coastal
NOCO-CO-29[2]	405352124070901	Northern Coast Ranges	North Coast coastal basins	Coastal
NOCO-CO-30[2]	403358124063701	Northern Coast Ranges	North Coast coastal basins	Coastal
NOCO-IN-01[2]	391544123133601	Northern Coast Ranges	North Coast inland basins	Coastal
NOCO-IN-02[2]	390048123222701	Northern Coast Ranges	North Coast inland basins	Coastal
NOCO-IN-03[2]	393918123283301	Northern Coast Ranges	North Coast inland basins	Coastal
NOCO-IN-04[2]	392412123191701	Northern Coast Ranges	North Coast inland basins	Coastal
NOCO-IN-05[2]	394117123291001	Northern Coast Ranges	North Coast inland basins	Coastal
NOCO-IN-06[2]	394700123125301	Northern Coast Ranges	North Coast inland basins	Coastal
NOCO-IN-07[2]	390329123083301	Northern Coast Ranges	North Coast inland basins	Coastal
NOCO-IN-08[2]	385817123061801	Northern Coast Ranges	North Coast inland basins	Coastal
NOCO-IN-09[2]	391451123114501	Northern Coast Ranges	North Coast inland basins	Coastal
NOCO-IN-10[2]	390528123103802	Northern Coast Ranges	North Coast inland basins	Coastal
NOCO-IN-11	391943123070001	Northern Coast Ranges	North Coast inland basins	Coastal
NOCO-IN-12	390425123091001	Northern Coast Ranges	North Coast inland basins	Coastal
NOCO-IN-13	390909123111701	Northern Coast Ranges	North Coast inland basins	Coastal
NOCO-IN-14	390717123093101	Northern Coast Ranges	North Coast inland basins	Coastal
NOCO-IN-15[2]	392605122561301	Northern Coast Ranges	North Coast inland basins	Coastal
NOCO-IN-16[2]	392637122570901	Northern Coast Ranges	North Coast inland basins	Coastal
NOCO-IN-17[2]	390756122523901	Northern Coast Ranges	North Coast inland basins	Coastal
NOCO-IN-18[2]	385951122523301	Northern Coast Ranges	North Coast inland basins	Coastal
NOCO-IN-19[2]	391717123114601	Northern Coast Ranges	North Coast inland basins	Coastal
NOCO-IN-20[2]	385637122505401	Northern Coast Ranges	North Coast inland basins	Coastal
NOCO-IN-21[2]	385935122520401	Northern Coast Ranges	North Coast inland basins	Coastal
NOCO-IN-22[2]	383947122285501	Northern Coast Ranges	North Coast inland basins	Coastal
NOCO-IN-23[2]	394855123144801	Northern Coast Ranges	North Coast inland basins	Coastal

Online resource (suppplementary table) 2. Selected attributes of GAMA-PBP (https://ca.water.usgs.gov/gama/) status wells sampled for orthophosphate concentration-page 11.

GAMA-PBP ID	USEPA Level III Ecoregions[4]	Level III Ecoregion Reference Concentration[4]	Status Sample Date	Status Sample Orthophosphate Concentration (mg/L as P)	Relative Concentration Category[5]	Redox state[6]	Elevation of LSD (meters above NAVD 88)[7]	Well depth (meters below LSD)[8]	Agricultural land use in 1974[9] (percent)	Natural land use in 1974[9] (percent)	Urban land use in 1974[9] (percent)
NOCO-CO-24[2]	Coast Range	0.0103	Sep 16 2009	0.281	high	oxic	7	5	0.0%	97.7%	2.3%
NOCO-CO-25[2]	Coast Range	0.0103	Sep 17 2009	0.092	moderate	anoxic	14	59	1.8%	62.3%	35.9%
NOCO-CO-26[2]	Coast Range	0.0103	Oct 5 2009	0.007	low	oxic	81	5	0.0%	64.8%	35.2%
NOCO-CO-27[2]	Coast Range	0.0103	Oct 6 2009	0.111	high	anoxic	2	122	79.1%	15.0%	5.9%
NOCO-CO-28[2]	Coast Range	0.0103	Oct 6 2009	2.251	high	anoxic	1	37	0.0%	98.2%	1.8%
NOCO-CO-29[2]	Coast Range	0.0103	Oct 7 2009	0.051	moderate	anoxic	3	61	93.0%	6.1%	0.9%
NOCO-CO-30[2]	Coast Range	0.0103	Oct 8 2009	0.016	moderate	oxic	119	60	0.0%	78.2%	21.8%
NOCO-IN-01[2]	Central California Foothills and Coastal Mountains	0.0300	Jun 1 2009	0.096	moderate	oxic	228	50	0.9%	42.5%	56.6%
NOCO-IN-02[2]	Coast Range	0.0103	Jun 2 2009	0.142	high	oxic	111	37	35.6%	43.8%	20.5%
NOCO-IN-03[2]	Klamath Mountains/California High North Coast Range	0.0325	Jun 3 2009	0.011	low	oxic	510	18	0.5%	66.7%	32.9%
NOCO-IN-04[2]	Klamath Mountains/California High North Coast Range	0.0325	Jun 4 2009	0.017	low	oxic	422	17	69.1%	21.8%	9.1%
NOCO-IN-05[2]	Klamath Mountains/California High North Coast Range	0.0325	Jun 8 2009	1.329	high	anoxic	498	18	20.9%	27.7%	51.4%
NOCO-IN-06[2]	Klamath Mountains/California High North Coast Range	0.0325	Jun 10 2009	0.241	high	anoxic	409	41	50.7%	39.2%	10.1%
NOCO-IN-07[2]	Central California Foothills and Coastal Mountains	0.0300	Jun 15 2009	0.014	low	oxic	159	12	8.9%	80.4%	10.7%
NOCO-IN-08[2]	Central California Foothills and Coastal Mountains	0.0300	Jun 16 2009	0.021	low	oxic	147	12	65.0%	23.8%	11.2%
NOCO-IN-09[2]	Central California Foothills and Coastal Mountains	0.0300	Jun 17 2009	0.128	high	anoxic	217	117	22.1%	45.5%	32.4%
NOCO-IN-10[2]	Central California Foothills and Coastal Mountains	0.0300	Jun 18 2009	0.012	low	oxic	169	31	85.0%	12.7%	2.3%
NOCO-IN-11	Central California Foothills and Coastal Mountains	0.0300	Jun 22 2009	0.187	high	anoxic	292	30	86.7%	1.4%	11.9%
NOCO-IN-12	Central California Foothills and Coastal Mountains	0.0300	Jun 23 2009	0.036	moderate	oxic	170	66	59.6%	38.0%	2.3%
NOCO-IN-13	Central California Foothills and Coastal Mountains	0.0300	Jun 24 2009	0.017	low	oxic	183	11	67.9%	0.0%	32.1%
NOCO-IN-14	Central California Foothills and Coastal Mountains	0.0300	Jun 25 2009	0.042	moderate	anoxic	201	41	73.5%	17.8%	8.7%
NOCO-IN-15[2]	Klamath Mountains/California High North Coast Range	0.0325	Jul 6 2009	2.409	high	anoxic	566	91	0.0%	97.7%	2.3%
NOCO-IN-16[2]	Klamath Mountains/California High North Coast Range	0.0325	Jul 7 2009	0.016	low	oxic	562	46	0.0%	99.1%	0.9%
NOCO-IN-17[2]	Central California Foothills and Coastal Mountains	0.0300	Jul 8 2009	0.067	moderate	oxic	417	23	8.2%	80.5%	11.4%
NOCO-IN-18[2]	Central California Foothills and Coastal Mountains	0.0300	Jul 9 2009	0.047	moderate	oxic	415	52	27.3%	60.0%	12.7%
NOCO-IN-19[2]	Central California Foothills and Coastal Mountains	0.0300	Jul 13 2009	0.034	moderate	oxic	251	24	61.9%	33.0%	5.0%
NOCO-IN-20[2]	Central California Foothills and Coastal Mountains	0.0300	Jul 14 2009	2.341	high	anoxic	448	23	0.0%	98.6%	1.4%
NOCO-IN-21[2]	Central California Foothills and Coastal Mountains	0.0300	Jul 15 2009	0.031	moderate	anoxic	110	16	16.8%	67.3%	15.9%
NOCO-IN-22[2]	Central California Foothills and Coastal Mountains	0.0300	Jul 16 2009	0.045	moderate	anoxic	244	55	26.7%	73.3%	0.0%
NOCO-IN-23[2]	Klamath Mountains/California High North Coast Range	0.0325	Jul 20 2009	0.011	low	oxic	426	43	22.4%	70.8%	6.8%

Status and trends of orthophosphate concentrations in groundwater used for public supply in California *Environmental Monitoring and Assessment, Robert Kent, Tyler D. Johnson, and Michael R. Rosen, U.S. Geological Survey California Water Science Center-rhkent@usgs.gov*

Online resource (suppplementary table) 2. Selected attributes of GAMA-PBP (https://ca.water.usgs.gov/gama/) status wells sampled for orthophosphate concentration-page 12.

GAMA-PBP ID	Agricultural land use in 1982[9] (percent)	Natural land use in 1982[9] (percent)	Urban land use in 1982[9] (percent)	Agricultural land use in 1992[9] (percent)	Natural land use in 1992[9] (percent)	Urban land use in 1992[9] (percent)	Agricultural land use in 2002[9] (percent)	Natural land use in 2002[9] (percent)	Urban land use in 2002[9] (percent)	Agricultural land use in 2012[9] (percent)	Natural land use in 2012[9] (percent)	Urban land use in 2012[9] (percent)	Age Classification[10]	Septic Tanks[11]	Aridity[12]
NOCO-CO-24[2]	0.0%	97.7%	2.3%	0.0%	97.2%	2.8%	0.0%	96.2%	3.8%	9.4%	86.9%	3.8%	Modern	12.40	1.703
NOCO-CO-25[2]	3.2%	60.5%	36.4%	3.2%	60.5%	36.4%	3.2%	60.5%	36.4%	3.2%	60.0%	36.8%	Premodern	7.34	1.198
NOCO-CO-26[2]	0.0%	64.8%	35.2%	0.0%	64.8%	35.2%	0.0%	61.1%	38.9%	0.0%	61.1%	38.9%	Modern	1.27	1.548
NOCO-CO-27[2]	79.1%	15.0%	5.9%	76.4%	17.3%	6.4%	76.8%	16.8%	6.4%	76.4%	16.8%	6.8%	Premodern	1.12	1.202
NOCO-CO-28[2]	0.0%	98.2%	1.8%	0.0%	98.2%	1.8%	0.0%	98.2%	1.8%	0.0%	98.2%	1.8%	Premodern	1.37	1.210
NOCO-CO-29[2]	93.5%	5.6%	0.9%	93.5%	5.6%	0.9%	93.5%	4.7%	1.9%	93.5%	4.2%	2.3%	Mixed	3.36	1.258
NOCO-CO-30[2]	0.0%	76.8%	23.2%	0.0%	75.0%	25.0%	0.0%	75.0%	25.0%	0.0%	75.0%	25.0%	Mixed	4.80	1.377
NOCO-IN-01[2]	1.8%	41.1%	57.1%	0.5%	9.6%	90.0%	0.5%	9.6%	90.0%	0.5%	9.1%	90.4%	Premodern	30.77	0.846
NOCO-IN-02[2]	47.5%	32.0%	20.5%	35.2%	44.3%	20.5%	37.4%	42.0%	20.5%	37.4%	42.0%	20.5%	Mixed	7.95	0.877
NOCO-IN-03[2]	0.5%	66.7%	32.9%	0.5%	66.2%	33.3%	0.5%	66.2%	33.3%	0.5%	66.2%	33.3%	Modern	12.51	1.709
NOCO-IN-04[2]	69.1%	21.8%	9.1%	69.1%	21.8%	9.1%	69.1%	21.8%	9.1%	69.1%	21.8%	9.1%	Modern	10.69	1.163
NOCO-IN-05[2]	20.9%	27.7%	51.4%	20.9%	27.7%	51.4%	20.9%	27.7%	51.4%	20.9%	27.7%	51.4%	Mixed	21.61	1.726
NOCO-IN-06[2]	50.2%	39.2%	10.6%	50.2%	39.2%	10.6%	62.2%	29.5%	8.3%	62.2%	29.5%	8.3%	Premodern	4.15	0.974
NOCO-IN-07[2]	8.9%	74.3%	16.8%	8.9%	71.0%	20.1%	12.6%	62.1%	25.2%	12.6%	62.1%	25.2%	Modern	3.51	0.826
NOCO-IN-08[2]	65.0%	23.8%	11.2%	65.0%	23.8%	11.2%	69.2%	18.7%	12.1%	69.2%	18.7%	12.1%	Mixed	2.34	0.820
NOCO-IN-09[2]	21.6%	9.4%	69.0%	21.6%	9.4%	69.0%	27.7%	8.0%	64.3%	27.7%	7.5%	64.8%	Premodern	24.92	0.842
NOCO-IN-10[2]	85.0%	12.7%	2.3%	84.5%	12.7%	2.7%	86.8%	9.5%	3.6%	86.8%	9.5%	3.6%	Modern	4.33	0.797
NOCO-IN-11	86.2%	1.4%	12.4%	86.2%	1.4%	12.4%	86.7%	0.9%	12.4%	86.7%	0.9%	12.4%	Mixed	14.67	0.920
NOCO-IN-12	61.0%	36.6%	2.3%	60.6%	37.1%	2.3%	66.2%	31.0%	2.8%	66.7%	30.5%	2.8%	Mixed	3.76	0.820
NOCO-IN-13	64.7%	0.0%	35.3%	63.8%	0.0%	36.2%	63.8%	0.0%	36.2%	63.8%	0.0%	36.2%	Modern	21.85	0.820
NOCO-IN-14	73.5%	17.8%	8.7%	73.5%	17.8%	8.7%	76.7%	14.6%	8.7%	76.7%	14.6%	8.7%	Mixed	4.66	0.808
NOCO-IN-15[2]	0.0%	97.7%	2.3%	0.0%	97.7%	2.3%	0.0%	97.7%	2.3%	0.0%	97.7%	2.3%	Mixed	0.12	1.069
NOCO-IN-16[2]	0.0%	99.1%	0.9%	0.0%	99.1%	0.9%	0.0%	99.1%	0.9%	0.0%	99.1%	0.9%	Mixed	0.12	1.034
NOCO-IN-17[2]	8.2%	24.1%	67.7%	8.2%	23.6%	68.2%	5.9%	23.6%	70.5%	5.9%	22.7%	71.4%	Mixed	4.93	0.780
NOCO-IN-18[2]	27.3%	60.0%	12.7%	26.8%	60.0%	13.2%	26.8%	10.5%	62.7%	44.1%	5.5%	50.5%	Mixed	12.07	0.652
NOCO-IN-19[2]	63.3%	31.7%	5.0%	61.9%	33.0%	5.0%	62.4%	32.6%	5.0%	63.8%	31.2%	5.0%	Modern	5.37	0.876
NOCO-IN-20[2]	0.0%	98.6%	1.4%	0.0%	98.6%	1.4%	0.0%	97.3%	2.7%	0.0%	97.3%	2.7%	Mixed	3.32	0.747
NOCO-IN-21[2]	16.8%	67.3%	15.9%	16.8%	67.3%	15.9%	16.8%	11.7%	71.5%	32.7%	7.0%	60.3%	Modern	11.38	0.636
NOCO-IN-22[2]	26.7%	73.3%	0.0%	26.7%	73.3%	0.0%	26.7%	73.3%	0.0%	30.3%	69.7%	0.0%	Mixed	1.72	0.801
NOCO-IN-23[2]	40.6%	5.5%	53.9%	21.9%	8.7%	69.4%	41.6%	8.7%	49.8%	41.6%	8.7%	49.8%	Modern	12.42	0.984

Online resource (suppplementary table) 2. Selected attributes of GAMA-PBP (https://ca.water.usgs.gov/gama/) status wells sampled for orthophosphate concentration-page 13

GAMA-PBP ID	USGS Station ID[1]	GAMA-PBP study unit	GAMA-PBP study area[3]	Hydrogeologic zone
NOCO-IN-24[2]	385852122503901	Northern Coast Ranges	North Coast inland basins	Coastal
NOCO-IN-25[2]	384545122362601	Northern Coast Ranges	North Coast inland basins	Coastal
NOCO-IN-26[2]	392305122332301	Northern Coast Ranges	North Coast inland basins	Coastal
NOCO-IN-27[2]	390309122413901	Northern Coast Ranges	North Coast inland basins	Coastal
NOCO-IN-28[2]	394604123123001	Northern Coast Ranges	North Coast inland basins	Coastal
MSMB-04	365218121490301	Monterey Bay and Salinas Valley Basins	Monterey Bay area basins	Coastal
MSMB-09	365500121470001	Monterey Bay and Salinas Valley Basins	Monterey Bay area basins	Coastal
MSMB-12	364100121470001	Monterey Bay and Salinas Valley Basins	Monterey Bay area basins	Coastal
MSMB-18	364600121430001	Monterey Bay and Salinas Valley Basins	Monterey Bay area basins	Coastal
MSMB-20	365425121452201	Monterey Bay and Salinas Valley Basins	Monterey Bay area basins	Coastal
MSMB-22	365429121411301	Monterey Bay and Salinas Valley Basins	Monterey Bay area basins	Coastal
MSMB-29	363800121390001	Monterey Bay and Salinas Valley Basins	Monterey Bay area basins	Coastal
MSMB-30	364155121384701	Monterey Bay and Salinas Valley Basins	Monterey Bay area basins	Coastal
MSMB-33	364800121370001	Monterey Bay and Salinas Valley Basins	Monterey Bay area basins	Coastal
MSMB-35[2]	365438121403901	Monterey Bay and Salinas Valley Basins	Monterey Bay area basins	Coastal
MSMB-37	364100121360001	Monterey Bay and Salinas Valley Basins	Monterey Bay area basins	Coastal
MSMB-40[2]	363700121340001	Monterey Bay and Salinas Valley Basins	Monterey Bay area basins	Coastal
MSMB-44	363100121270001	Monterey Bay and Salinas Valley Basins	Monterey Bay area basins	Coastal
MSMB-45	363200121520001	Monterey Bay and Salinas Valley Basins	Monterey Bay area basins	Coastal
MSMB-47	363400121300001	Monterey Bay and Salinas Valley Basins	Monterey Bay area basins	Coastal
MSPR-01	353041120394501	Monterey Bay and Salinas Valley Basins	Paso Robles area basin	Coastal
MSPR-08	354501120413301	Monterey Bay and Salinas Valley Basins	Paso Robles area basin	Coastal
MSPR-10	354911120452701	Monterey Bay and Salinas Valley Basins	Paso Robles area basin	Coastal
MSSC-04	370304122014201	Monterey Bay and Salinas Valley Basins	Santa Cruz area basins	Coastal
MSSC-06	365700121580001	Monterey Bay and Salinas Valley Basins	Santa Cruz area basins	Coastal
MSSC-07	365900121570001	Monterey Bay and Salinas Valley Basins	Santa Cruz area basins	Coastal
MSSC-08	370150121565301	Monterey Bay and Salinas Valley Basins	Santa Cruz area basins	Coastal
MSSV-01[2]	355700120510001	Monterey Bay and Salinas Valley Basins	Salinas Valley basins	Coastal
MSSV-02	360000120540001	Monterey Bay and Salinas Valley Basins	Salinas Valley basins	Coastal
MSSV-03	360600121000001	Monterey Bay and Salinas Valley Basins	Salinas Valley basins	Coastal

Online resource (suppplementary table) 2. Selected attributes of GAMA-PBP (https://ca.water.usgs.gov/gama/) status wells sampled for orthophosphate concentration-page 14.

GAMA-PBP ID	USEPA Level III Ecoregions[4]	Level III Ecoregion Reference Concentration[4]	Status Sample Date	Status Sample Orthophosphate Concentration (mg/L as P)	Relative Concentration Category[5]	Redox state[6]	Elevation of LSD (meters above NAVD 88)[7]	Well depth (meters below LSD)[8]	Agricultural land use in 1974[9] (percent)	Natural land use in 1974[9] (percent)	Urban land use in 1974[9] (percent)
NOCO-IN-24[2]	Central California Foothills and Coastal Mountains	0.0300	Jul 21 2009	0.033	moderate	oxic	421	35	2.3%	47.9%	49.8%
NOCO-IN-25[2]	Central California Foothills and Coastal Mountains	0.0300	Jul 22 2009	0.346	high	oxic	327	86	34.4%	45.0%	20.6%
NOCO-IN-26[2]	Central California Foothills and Coastal Mountains	0.0300	Jul 23 2009	0.043	moderate	oxic	352	38	0.0%	99.5%	0.5%
NOCO-IN-27[2]	Central California Foothills and Coastal Mountains	0.0300	Jul 27 2009	0.304	high	anoxic	541	47	39.3%	60.7%	0.0%
NOCO-IN-28[2]	Klamath Mountains/California High North Coast Range	0.0325	Jul 28 2009	0.057	moderate	anoxic	410	12	90.0%	9.6%	0.5%
MSMB-04	Central California Foothills and Coastal Mountains	0.0300	Aug 17 2005	0.011	low	anoxic	2	244	40.7%	43.1%	16.2%
MSMB-09	Central California Foothills and Coastal Mountains	0.0300	Aug 15 2005	0.079	moderate	oxic	31	142	11.4%	0.0%	88.6%
MSMB-12	Central California Foothills and Coastal Mountains	0.0300	Sep 14 2005	0.013	low	anoxic	31	594	0.0%	7.3%	92.7%
MSMB-18	Central California Foothills and Coastal Mountains	0.0300	Aug 11 2005	0.057	moderate	oxic	23	195	5.0%	26.1%	68.8%
MSMB-20	Central California Foothills and Coastal Mountains	0.0300	Aug 16 2005	0.068	moderate	oxic	8	54	0.9%	0.0%	99.1%
MSMB-22	Central California Foothills and Coastal Mountains	0.0300	Sep 19 2005	0.014	low	anoxic	17	128	92.7%	7.3%	0.0%
MSMB-29	Central California Foothills and Coastal Mountains	0.0300	Aug 30 2005	0.066	moderate	oxic	16	84	96.8%	2.3%	0.9%
MSMB-30	Central California Foothills and Coastal Mountains	0.0300	Aug 8 2005	0.060	moderate	oxic	10	204	0.0%	0.0%	100.0%
MSMB-33	Central California Foothills and Coastal Mountains	0.0300	Sep 13 2005	0.039	moderate	oxic	123	152	0.0%	0.0%	100.0%
MSMB-35[2]	Central California Foothills and Coastal Mountains	0.0300	Sep 20 2005	0.048	moderate	anoxic	18	na	90.0%	8.2%	1.8%
MSMB-37	Central California Foothills and Coastal Mountains	0.0300	Sep 1 2005	0.054	moderate	oxic	35	247	15.8%	1.4%	82.8%
MSMB-40[2]	Central California Foothills and Coastal Mountains	0.0300	Aug 29 2005	0.034	moderate	oxic	23	na	70.2%	9.2%	20.6%
MSMB-44	Central California Foothills and Coastal Mountains	0.0300	Sep 22 2005	0.025	low	oxic	37	119	73.5%	2.3%	24.2%
MSMB-45	Central California Foothills and Coastal Mountains	0.0300	Aug 10 2005	0.075	moderate	oxic	22	45	3.7%	17.1%	79.3%
MSMB-47	Central California Foothills and Coastal Mountains	0.0300	Aug 3 2005	0.006	low	oxic	34	21	51.1%	0.9%	47.9%
MSPR-01	Central California Foothills and Coastal Mountains	0.0300	Jul 19 2005	0.010	low	oxic	253	152	44.8%	49.3%	5.9%
MSPR-08	Central California Foothills and Coastal Mountains	0.0300	Jul 20 2005	0.005	low	oxic	184	91	2.8%	10.6%	86.7%
MSPR-10	Central California Foothills and Coastal Mountains	0.0300	Jul 18 2005	0.005	low	anoxic	171	61	0.0%	72.3%	27.7%
MSSC-04	Coast Range	0.0103	Aug 25 2005	0.007	low	anoxic	183	110	0.0%	0.0%	100.0%
MSSC-06	Central California Foothills and Coastal Mountains	0.0300	Aug 24 2005	0.103	high	anoxic	13	70	0.0%	0.0%	100.0%
MSSC-07	Central California Foothills and Coastal Mountains	0.0300	Aug 23 2005	0.018	low	anoxic	17	200	0.0%	0.0%	100.0%
MSSC-08	Coast Range	0.0103	Sep 15 2005	0.075	moderate	anoxic	199	73	0.5%	56.2%	43.4%
MSSV-01[2]	Central California Foothills and Coastal Mountains	0.0300	Jul 26 2005	0.005	low	anoxic	147	na	0.0%	50.5%	49.5%
MSSV-02	Central California Foothills and Coastal Mountains	0.0300	Aug 4 2005	0.051	moderate	anoxic	144	40	57.3%	39.1%	3.6%
MSSV-03	Central California Foothills and Coastal Mountains	0.0300	Sep 12 2005	0.106	high	oxic	111	43	93.6%	6.4%	0.0%

Online resource (suppplementary table) 2. Selected attributes of GAMA-PBP (https://ca.water.usgs.gov/gama/) status wells sampled for orthophosphate concentration-page 15.

GAMA-PBP ID	Agricultural land use in 1982[9] (percent)	Natural land use in 1982[9] (percent)	Urban land use in 1982[9] (percent)	Agricultural land use in 1992[9] (percent)	Natural land use in 1992[9] (percent)	Urban land use in 1992[9] (percent)	Agricultural land use in 2002[9] (percent)	Natural land use in 2002[9] (percent)	Urban land use in 2002[9] (percent)	Agricultural land use in 2012[9] (percent)	Natural land use in 2012[9] (percent)	Urban land use in 2012[9] (percent)	Age Classification[10]	Septic Tanks[11]	Aridity[12]
NOCO-IN-24[2]	8.3%	29.0%	62.7%	2.3%	31.3%	66.4%	1.4%	16.1%	82.5%	7.4%	12.0%	80.6%	Mixed	7.16	0.613
NOCO-IN-25[2]	34.4%	45.0%	20.6%	34.4%	44.5%	21.1%	34.4%	44.5%	21.1%	34.4%	24.3%	41.3%	Mixed	4.77	0.826
NOCO-IN-26[2]	0.0%	99.5%	0.5%	0.0%	99.5%	0.5%	0.0%	99.5%	0.5%	23.2%	76.4%	0.5%	Modern	0.28	0.483
NOCO-IN-27[2]	39.3%	60.7%	0.0%	39.3%	60.7%	0.0%	39.3%	60.7%	0.0%	39.3%	60.7%	0.0%	Mixed	1.31	0.706
NOCO-IN-28[2]	89.0%	9.6%	1.4%	89.0%	9.6%	1.4%	96.3%	2.3%	1.4%	96.3%	2.3%	1.4%	Mixed	4.15	0.978
MSMB-04	39.4%	27.3%	33.3%	37.5%	27.3%	35.2%	40.3%	25.9%	33.8%	40.3%	25.9%	33.8%	Mixed	6.27	0.425
MSMB-09	3.2%	0.0%	96.8%	0.0%	0.0%	100.0%	0.0%	0.0%	100.0%	0.0%	0.0%	100.0%	Mixed	6.61	0.481
MSMB-12	0.0%	6.4%	93.6%	0.0%	6.4%	93.6%	2.3%	6.4%	91.4%	2.3%	6.4%	91.4%	Premodern	0.00	0.325
MSMB-18	4.6%	25.2%	70.2%	4.6%	25.2%	70.2%	9.6%	19.7%	70.6%	9.6%	19.3%	71.1%	Premodern	10.48	0.342
MSMB-20	0.0%	0.0%	100.0%	0.0%	0.0%	100.0%	0.0%	0.0%	100.0%	0.0%	0.0%	100.0%	Mixed	0.22	0.475
MSMB-22	93.2%	6.8%	0.0%	93.2%	0.0%	6.8%	93.2%	0.0%	6.8%	93.2%	0.0%	6.8%	Mixed	3.97	0.444
MSMB-29	96.8%	2.3%	0.9%	96.8%	2.3%	0.9%	96.8%	2.3%	0.9%	96.8%	2.3%	0.9%	Modern	1.24	0.306
MSMB-30	0.0%	0.0%	100.0%	0.0%	0.0%	100.0%	0.0%	0.0%	100.0%	0.0%	0.0%	100.0%	Premodern	2.18	0.313
MSMB-33	0.0%	0.0%	100.0%	0.0%	0.0%	100.0%	0.0%	0.0%	100.0%	0.0%	0.0%	100.0%	Mixed	61.55	0.421
MSMB-35[2]	89.1%	7.7%	3.2%	88.6%	7.3%	4.1%	87.7%	5.9%	6.4%	87.7%	5.0%	7.3%	Modern	1.26	0.439
MSMB-37	14.9%	1.4%	83.7%	7.7%	0.0%	92.3%	3.6%	0.0%	96.4%	0.0%	0.0%	100.0%	Premodern	4.79	0.287
MSMB-40[2]	69.3%	8.7%	22.0%	68.3%	8.7%	22.9%	66.5%	8.7%	24.8%	66.5%	5.5%	28.0%	Mixed	1.29	0.287
MSMB-44	73.5%	2.3%	24.2%	73.1%	2.3%	24.7%	73.5%	1.8%	24.7%	73.5%	0.5%	26.0%	ModernOrMixed	0.86	0.236
MSMB-45	3.2%	16.6%	80.2%	3.2%	16.6%	80.2%	3.2%	16.6%	80.2%	3.2%	15.7%	81.1%	Modern	16.65	0.368
MSMB-47	50.7%	0.9%	48.4%	50.2%	0.9%	48.9%	32.4%	0.5%	67.1%	32.4%	0.5%	67.1%	Premodern	0.80	0.281
MSPR-01	45.7%	48.4%	5.9%	45.7%	26.7%	27.6%	45.2%	25.8%	29.0%	31.7%	24.4%	43.9%	Modern	44.32	0.347
MSPR-08	5.0%	8.3%	86.7%	4.6%	5.5%	89.9%	0.0%	5.0%	95.0%	0.0%	4.1%	95.9%	Premodern	12.83	0.233
MSPR-10	0.0%	72.3%	27.7%	0.0%	71.8%	28.2%	0.0%	70.5%	29.5%	0.0%	67.7%	32.3%	Premodern	0.16	0.228
MSSC-04	0.0%	0.0%	100.0%	0.0%	0.0%	100.0%	0.0%	0.0%	100.0%	0.0%	0.0%	100.0%	Mixed	11.09	0.837
MSSC-06	0.0%	0.0%	100.0%	0.0%	0.0%	100.0%	0.0%	0.0%	100.0%	0.0%	0.0%	100.0%	Mixed	6.84	0.569
MSSC-07	0.0%	0.0%	100.0%	0.0%	0.0%	100.0%	0.0%	0.0%	100.0%	0.0%	0.0%	100.0%	Mixed	52.52	0.614
MSSC-08	0.5%	0.0%	99.5%	0.5%	0.0%	99.5%	0.5%	0.0%	99.5%	0.5%	0.0%	99.5%	Premodern	24.92	0.678
MSSV-01[2]	0.0%	50.5%	49.5%	0.0%	50.5%	49.5%	0.0%	50.5%	49.5%	0.0%	44.4%	55.6%	Premodern	0.13	0.232
MSSV-02	57.3%	39.1%	3.6%	56.4%	40.0%	3.6%	59.5%	36.4%	4.1%	59.5%	36.4%	4.1%	Modern	0.50	0.233
MSSV-03	93.6%	6.4%	0.0%	93.6%	6.4%	0.0%	93.6%	6.4%	0.0%	93.6%	6.4%	0.0%	ModernOrMixed	0.13	0.229

Online resource (suppplementary table) 2. Selected attributes of GAMA-PBP (https://ca.water.usgs.gov/gama/) status wells sampled for orthophosphate concentration-page 16

GAMA-PBP ID	USGS Station ID[1]	GAMA-PBP study unit	GAMA-PBP study area[3]	Hydrogeologic zone
MSSV-07	361207121075501	Monterey Bay and Salinas Valley Basins	Salinas Valley basins	Coastal
MSSV-11	361900121160001	Monterey Bay and Salinas Valley Basins	Salinas Valley basins	Coastal
MSSV-18[2]	362500121180001	Monterey Bay and Salinas Valley Basins	Salinas Valley basins	Coastal
MSSV-19	362800121220001	Monterey Bay and Salinas Valley Basins	Salinas Valley basins	Coastal
SB-01	342500119520001	Santa Barbara	Santa Barbara area basins	Coastal
SB-02	342603119500501	Santa Barbara	Santa Barbara area basins	Coastal
SB-03	342640119475402	Santa Barbara	Santa Barbara area basins	Coastal
SB-04	342553119474201	Santa Barbara	Santa Barbara area basins	Coastal
SB-05	342708119465401	Santa Barbara	Santa Barbara area basins	Coastal
SB-06	342612119462301	Santa Barbara	Santa Barbara area basins	Coastal
SB-07	342647119451701	Santa Barbara	Santa Barbara area basins	Coastal
SB-08	342610119444801	Santa Barbara	Santa Barbara area basins	Coastal
SB-09	342600119390001	Santa Barbara	Santa Barbara area basins	Coastal
SB-10[2]	342508119383101	Santa Barbara	Santa Barbara area basins	Coastal
SB-11	342500119380001	Santa Barbara	Santa Barbara area basins	Coastal
SB-12	342623119373201	Santa Barbara	Santa Barbara area basins	Coastal
SB-13	342500119370001	Santa Barbara	Santa Barbara area basins	Coastal
SB-14	342500119340001	Santa Barbara	Santa Barbara area basins	Coastal
SB-15	342400119310001	Santa Barbara	Santa Barbara area basins	Coastal
SB-16	342409119302101	Santa Barbara	Santa Barbara area basins	Coastal
SB-17	342318119275401	Santa Barbara	Santa Barbara area basins	Coastal
SB-18	342300119270001	Santa Barbara	Santa Barbara area basins	Coastal
SF-01[2]	374500122300001	San Francisco Bay	South San Francisco Bay basins	Coastal
SF-02[2]	374605122284801	San Francisco Bay	South San Francisco Bay basins	Coastal
SF-03[2]	374100122290001	San Francisco Bay	South San Francisco Bay basins	Coastal
SF-04[2]	374100122280001	San Francisco Bay	South San Francisco Bay basins	Coastal
SF-05[2]	373700122240001	San Francisco Bay	South San Francisco Bay basins	Coastal
SF-06[2]	373338122191301	San Francisco Bay	South San Francisco Bay basins	Coastal
SF-07[2]	372722122113101	San Francisco Bay	South San Francisco Bay basins	Coastal
SF-08[2]	372750122112201	San Francisco Bay	South San Francisco Bay basins	Coastal

Online resource (suppplementary table) 2. Selected attributes of GAMA-PBP (https://ca.water.usgs.gov/gama/) status wells sampled for orthophosphate concentration-page 17.

GAMA-PBP ID	USEPA Level III Ecoregions[4]	Level III Ecoregion Reference Concentration[4]	Status Sample Date	Status Sample Orthophosphate Concentration (mg/L as P)	Relative Concentration Category[5]	Redox state[6]	Elevation of LSD (meters above NAVD 88)[7]	Well depth (meters below LSD)[8]	Agricultural land use in 1974[9] (percent)	Natural land use in 1974[9] (percent)	Urban land use in 1974[9] (percent)
MSSV-07	Central California Foothills and Coastal Mountains	0.0300	Aug 2 2005	0.040	moderate	anoxic	93	65	5.0%	32.7%	62.3%
MSSV-11	Central California Foothills and Coastal Mountains	0.0300	Jul 25 2005	0.013	low	oxic	99	269	90.9%	0.5%	8.6%
MSSV-18[2]	Central California Foothills and Coastal Mountains	0.0300	Aug 1 2005	0.007	low	anoxic	62	na	70.5%	0.0%	29.5%
MSSV-19	Central California Foothills and Coastal Mountains	0.0300	Jul 27 2005	0.011	low	oxic	72	253	63.6%	4.1%	32.3%
SB-01	Southern California Mountains	0.0109	Feb 3 2011	0.052	moderate	anoxic	14	46	0.0%	0.0%	100.0%
SB-02	Southern California Mountains	0.0109	Feb 1 2011	0.123	high	anoxic	6	256	0.0%	0.0%	100.0%
SB-03	Southern California Mountains	0.0109	Feb 1 2011	0.018	moderate	anoxic	28	137	0.0%	0.0%	100.0%
SB-04	Southern California Mountains	0.0109	Jan 31 2011	0.088	moderate	oxic	14	213	0.0%	0.0%	100.0%
SB-05	Southern California Mountains	0.0109	Feb 14 2011	0.035	moderate	oxic	111	128	0.0%	2.8%	97.2%
SB-06	Southern California Mountains	0.0109	Feb 2 2011	0.068	moderate	anoxic	25	192	0.0%	1.4%	98.6%
SB-07	Southern California Mountains	0.0109	Jan 26 2011	0.063	moderate	oxic	60	130	0.0%	0.0%	100.0%
SB-08	Southern California Mountains	0.0109	Jan 26 2011	0.093	moderate	anoxic	54	178	0.0%	0.0%	100.0%
SB-09	Southern California Mountains	0.0109	Feb 8 2011	0.070	moderate	oxic	166	107	0.0%	1.4%	98.6%
SB-10[2]	Southern California Mountains	0.0109	Feb 8 2011	0.157	high	anoxic	9	44	0.0%	22.8%	77.2%
SB-11	Southern California Mountains	0.0109	Feb 9 2011	0.028	moderate	oxic	8	63	0.0%	15.4%	84.6%
SB-12	Southern California Mountains	0.0109	Feb 7 2011	0.025	moderate	oxic	81	85	0.0%	0.0%	100.0%
SB-13	Southern California Mountains	0.0109	Feb 7 2011	0.026	moderate	oxic	25	140	5.9%	0.0%	94.1%
SB-14	Southern California Mountains	0.0109	Feb 9 2011	0.052	moderate	anoxic	109	297	0.0%	31.8%	68.2%
SB-15	Central California Foothills and Coastal Mountains	0.0300	Feb 2 2011	0.050	moderate	anoxic	13	292	2.8%	0.0%	97.2%
SB-16	Central California Foothills and Coastal Mountains	0.0300	Feb 3 2011	0.051	moderate	oxic	27	379	37.3%	1.4%	61.3%
SB-17	Central California Foothills and Coastal Mountains	0.0300	Feb 16 2011	0.030	moderate	oxic	47	76	48.9%	48.4%	2.7%
SB-18	Central California Foothills and Coastal Mountains	0.0300	Feb 10 2011	0.031	moderate	oxic	60	90	25.6%	72.6%	1.8%
SF-01[2]	Central California Foothills and Coastal Mountains	0.0300	Jun 20 2007	0.105	high	oxic	8	na	0.0%	12.2%	87.8%
SF-02[2]	Central California Foothills and Coastal Mountains	0.0300	Jun 20 2007	0.060	moderate	oxic	48	110	0.0%	4.2%	95.8%
SF-03[2]	Central California Foothills and Coastal Mountains	0.0300	May 24 2007	0.086	moderate	oxic	40	125	0.0%	0.0%	100.0%
SF-04[2]	Central California Foothills and Coastal Mountains	0.0300	May 24 2007	0.075	moderate	oxic	67	na	0.0%	0.0%	100.0%
SF-05[2]	Central California Foothills and Coastal Mountains	0.0300	May 23 2007	0.103	high	oxic	16	146	0.0%	0.0%	100.0%
SF-06[2]	Central California Foothills and Coastal Mountains	0.0300	May 23 2007	0.042	moderate	oxic	9	55	0.0%	0.0%	100.0%
SF-07[2]	Central California Foothills and Coastal Mountains	0.0300	May 21 2007	0.054	moderate	oxic	21	67	0.0%	0.0%	100.0%
SF-08[2]	Central California Foothills and Coastal Mountains	0.0300	May 21 2007	0.109	high	oxic	15	84	0.0%	0.0%	100.0%

Status and trends of orthophosphate concentrations in groundwater used for public supply in California *Environmental Monitoring and Assessment, Robert Kent, Tyler D. Johnson, and Michael R. Rosen, U.S. Geological Survey California Water Science Center-rhkent@usgs.gov*

Online resource (suppplementary table) 2. Selected attributes of GAMA-PBP (https://ca.water.usgs.gov/gama/) status wells sampled for orthophosphate concentration-page 18.

GAMA-PBP ID	Agricultural land use in 1982[9] (percent)	Natural land use in 1982[9] (percent)	Urban land use in 1982[9] (percent)	Agricultural land use in 1992[9] (percent)	Natural land use in 1992[9] (percent)	Urban land use in 1992[9] (percent)	Agricultural land use in 2002[9] (percent)	Natural land use in 2002[9] (percent)	Urban land use in 2002[9] (percent)	Agricultural land use in 2012[9] (percent)	Natural land use in 2012[9] (percent)	Urban land use in 2012[9] (percent)	Age Classification[10]	Septic Tanks[11]	Aridity[12]
MSSV-07	5.0%	32.3%	62.7%	5.0%	29.1%	65.9%	7.3%	20.9%	71.8%	7.3%	14.5%	78.2%	Modern	0.06	0.236
MSSV-11	90.9%	0.5%	8.6%	90.9%	0.5%	8.6%	91.4%	0.0%	8.6%	91.4%	0.0%	8.6%	Modern	5.10	0.238
MSSV-18[2]	63.6%	0.0%	36.4%	63.2%	0.0%	36.8%	62.3%	0.0%	37.7%	62.3%	0.0%	37.7%	Premodern	6.25	0.218
MSSV-19	64.5%	3.2%	32.3%	59.0%	8.8%	32.3%	57.6%	8.3%	34.1%	4.1%	1.4%	94.5%	Premodern	1.31	0.223
SB-01	0.0%	0.0%	100.0%	0.0%	0.0%	100.0%	0.0%	0.0%	100.0%	0.0%	0.0%	100.0%	Premodern	4.97	0.313
SB-02	0.0%	0.0%	100.0%	0.0%	0.0%	100.0%	0.0%	0.0%	100.0%	0.0%	0.0%	100.0%	Premodern	0.00	0.308
SB-03	0.0%	0.0%	100.0%	0.0%	0.0%	100.0%	0.0%	0.0%	100.0%	0.0%	0.0%	100.0%	Mixed	4.93	0.317
SB-04	0.0%	0.0%	100.0%	0.0%	0.0%	100.0%	0.0%	0.0%	100.0%	0.0%	0.0%	100.0%	Mixed	1.61	0.312
SB-05	0.0%	0.9%	99.1%	0.0%	0.9%	99.1%	0.0%	0.0%	100.0%	0.0%	0.0%	100.0%	Mixed	18.01	0.319
SB-06	0.0%	1.4%	98.6%	0.0%	1.4%	98.6%	0.0%	1.4%	98.6%	0.0%	1.4%	98.6%	Premodern	14.82	0.341
SB-07	0.0%	0.0%	100.0%	0.0%	0.0%	100.0%	0.0%	0.0%	100.0%	0.0%	0.0%	100.0%	Mixed	27.96	0.339
SB-08	0.0%	0.0%	100.0%	0.0%	0.0%	100.0%	0.0%	0.0%	100.0%	0.0%	0.0%	100.0%	Mixed	19.82	0.335
SB-09	0.0%	1.4%	98.6%	0.0%	0.5%	99.5%	0.0%	0.0%	100.0%	0.0%	0.0%	100.0%	Mixed	9.23	0.375
SB-10[2]	0.0%	22.8%	77.2%	0.0%	22.8%	77.2%	0.0%	22.8%	77.2%	0.0%	22.3%	77.7%	Mixed	6.59	na
SB-11	0.0%	15.4%	84.6%	0.0%	15.4%	84.6%	0.0%	15.4%	84.6%	0.0%	15.4%	84.6%	Modern	15.99	0.313
SB-12	0.0%	0.0%	100.0%	0.0%	0.0%	100.0%	0.0%	0.0%	100.0%	0.0%	0.0%	100.0%	Modern	10.02	0.362
SB-13	5.9%	0.0%	94.1%	1.4%	0.0%	98.6%	3.2%	0.0%	96.8%	3.2%	0.0%	96.8%	Mixed	13.92	0.309
SB-14	0.0%	30.0%	70.0%	0.0%	1.4%	98.6%	0.0%	1.4%	98.6%	0.0%	1.4%	98.6%	Premodern	19.91	0.372
SB-15	1.9%	0.0%	98.1%	1.9%	0.0%	98.1%	1.4%	0.0%	98.6%	1.4%	0.0%	98.6%	Premodern	15.52	0.292
SB-16	33.6%	1.4%	65.0%	32.3%	1.4%	66.4%	32.3%	0.9%	66.8%	32.3%	0.0%	67.7%	Mixed	19.08	0.297
SB-17	55.3%	41.1%	3.7%	54.8%	40.6%	4.6%	53.4%	37.4%	9.1%	53.4%	35.6%	11.0%	Modern	3.96	0.314
SB-18	27.4%	69.9%	2.7%	26.9%	69.4%	3.7%	31.1%	61.6%	7.3%	31.1%	61.6%	7.3%	Mixed	3.20	0.356
SF-01[2]	0.0%	12.2%	87.8%	0.0%	12.2%	87.8%	0.0%	12.2%	87.8%	0.0%	10.8%	89.2%	Mixed	0.00	0.481
SF-02[2]	0.0%	4.2%	95.8%	0.0%	4.2%	95.8%	0.0%	4.2%	95.8%	0.0%	4.2%	95.8%	Mixed	0.00	0.537
SF-03[2]	0.0%	0.0%	100.0%	0.0%	0.0%	100.0%	0.0%	0.0%	100.0%	0.0%	0.0%	100.0%	ModernOrMixed	0.00	0.509
SF-04[2]	0.0%	0.0%	100.0%	0.0%	0.0%	100.0%	0.0%	0.0%	100.0%	0.0%	0.0%	100.0%	Mixed	0.65	0.545
SF-05[2]	0.0%	0.0%	100.0%	0.0%	0.0%	100.0%	0.0%	0.0%	100.0%	0.0%	0.0%	100.0%	Premodern	0.00	0.496
SF-06[2]	0.0%	0.0%	100.0%	0.0%	0.0%	100.0%	0.0%	0.0%	100.0%	0.0%	0.0%	100.0%	Modern	1.07	0.448
SF-07[2]	0.0%	0.0%	100.0%	0.0%	0.0%	100.0%	0.0%	0.0%	100.0%	0.0%	0.0%	100.0%	Modern	0.00	0.369
SF-08[2]	0.0%	0.0%	100.0%	0.0%	0.0%	100.0%	0.0%	0.0%	100.0%	0.0%	0.0%	100.0%	Mixed	0.00	0.367

Online resource (suppplementary table) 2. Selected attributes of GAMA-PBP (https://ca.water.usgs.gov/gama/) status wells sampled for orthophosphate concentration-page 19

GAMA-PBP ID	USGS Station ID[1]	GAMA-PBP study unit	GAMA-PBP study area[3]	Hydrogeologic zone
SF-09[2]	372224122051501	San Francisco Bay	South San Francisco Bay basins	Coastal
SF-10[2]	372347122040301	San Francisco Bay	South San Francisco Bay basins	Coastal
SF-11[2]	372119122033201	San Francisco Bay	South San Francisco Bay basins	Coastal
SF-12[2]	372015122025101	San Francisco Bay	South San Francisco Bay basins	Coastal
SF-13[2]	372029122012401	San Francisco Bay	South San Francisco Bay basins	Coastal
SF-14[2]	372141121591101	San Francisco Bay	South San Francisco Bay basins	Coastal
SF-15[2]	372121121571001	San Francisco Bay	South San Francisco Bay basins	Coastal
SF-16[2]	372315121565201	San Francisco Bay	South San Francisco Bay basins	Coastal
SF-17[2]	372400121550001	San Francisco Bay	South San Francisco Bay basins	Coastal
SF-18[2]	372400121510001	San Francisco Bay	South San Francisco Bay basins	Coastal
SF-19[2]	371600122000001	San Francisco Bay	South San Francisco Bay basins	Coastal
SF-20[2]	371658121573802	San Francisco Bay	South San Francisco Bay basins	Coastal
SF-21[2]	371837121574710	San Francisco Bay	South San Francisco Bay basins	Coastal
SF-22[2]	371809121555601	San Francisco Bay	South San Francisco Bay basins	Coastal
SF-23[2]	372100121560001	San Francisco Bay	South San Francisco Bay basins	Coastal
SF-24	371940121525901	San Francisco Bay	South San Francisco Bay basins	Coastal
SF-25[2]	372210121520501	San Francisco Bay	South San Francisco Bay basins	Coastal
SF-26[2]	371500121520001	San Francisco Bay	South San Francisco Bay basins	Coastal
SF-27[2]	371659121525202	San Francisco Bay	South San Francisco Bay basins	Coastal
SF-28	371801121501401	San Francisco Bay	South San Francisco Bay basins	Coastal
SF-29[2]	371529121490101	San Francisco Bay	South San Francisco Bay basins	Coastal
SF-30[2]	371900121450001	San Francisco Bay	South San Francisco Bay basins	Coastal
SF-31[2]	371357121454601	San Francisco Bay	South San Francisco Bay basins	Coastal
SF-32[2]	371300121440001	San Francisco Bay	South San Francisco Bay basins	Coastal
SF-33[2]	371000121390001	San Francisco Bay	South San Francisco Bay basins	Coastal
SF-34[2]	373303121575001	San Francisco Bay	South San Francisco Bay basins	Coastal
SF-35	373200122010001	San Francisco Bay	South San Francisco Bay basins	Coastal
SF-36[2]	373300121580001	San Francisco Bay	South San Francisco Bay basins	Coastal
SF-37[2]	373356121583701	San Francisco Bay	South San Francisco Bay basins	Coastal
SF-38[2]	373700122050001	San Francisco Bay	South San Francisco Bay basins	Coastal

Online resource (suppplementary table) 2. Selected attributes of GAMA-PBP (https://ca.water.usgs.gov/gama/) status wells sampled for orthophosphate concentration-page 20.

GAMA-PBP ID	USEPA Level III Ecoregions[4]	Level III Ecoregion Reference Concentration[4]	Status Sample Date	Status Sample Orthophosphate Concentration (mg/L as P)	Relative Concentration Category[5]	Redox state[6]	Elevation of LSD (meters above NAVD 88)[7]	Well depth (meters below LSD)[8]	Agricultural land use in 1974[9] (percent)	Natural land use in 1974[9] (percent)	Urban land use in 1974[9] (percent)
SF-09[2]	Central California Foothills and Coastal Mountains	0.0300	May 3 2007	0.053	moderate	oxic	44	341	0.0%	0.0%	100.0%
SF-10[2]	Central California Foothills and Coastal Mountains	0.0300	May 3 2007	0.050	moderate	oxic	22	207	0.0%	0.0%	100.0%
SF-11[2]	Central California Foothills and Coastal Mountains	0.0300	May 22 2007	0.031	moderate	oxic	62	182	0.0%	0.0%	100.0%
SF-12[2]	Central California Foothills and Coastal Mountains	0.0300	May 22 2007	0.032	moderate	oxic	77	na	0.0%	0.0%	100.0%
SF-13[2]	Central California Foothills and Coastal Mountains	0.0300	Apr 23 2007	0.068	moderate	oxic	54	232	0.0%	0.0%	100.0%
SF-14[2]	Central California Foothills and Coastal Mountains	0.0300	Apr 25 2007	0.052	moderate	oxic	19	161	0.0%	0.0%	100.0%
SF-15[2]	Central California Foothills and Coastal Mountains	0.0300	Apr 26 2007	0.034	moderate	anoxic	21	247	0.0%	0.0%	100.0%
SF-16[2]	Central California Foothills and Coastal Mountains	0.0300	Apr 30 2007	0.059	moderate	anoxic	13	203	3.6%	0.0%	96.4%
SF-17[2]	Central California Foothills and Coastal Mountains	0.0300	Jun 5 2007	0.063	moderate	oxic	9	na	54.6%	8.8%	36.6%
SF-18[2]	Central California Foothills and Coastal Mountains	0.0300	May 2 2007	0.042	moderate	oxic	37	249	0.0%	0.0%	100.0%
SF-19[2]	Central California Foothills and Coastal Mountains	0.0300	Jun 18 2007	0.066	moderate	anoxic	113	165	0.0%	0.0%	100.0%
SF-20[2]	Central California Foothills and Coastal Mountains	0.0300	May 2 2007	0.026	low	oxic	62	256	0.0%	0.0%	100.0%
SF-21[2]	Central California Foothills and Coastal Mountains	0.0300	May 2 2007	0.025	low	oxic	49	248	0.0%	0.0%	100.0%
SF-22[2]	Central California Foothills and Coastal Mountains	0.0300	May 3 2007	0.024	low	oxic	53	252	0.0%	0.0%	100.0%
SF-23[2]	Central California Foothills and Coastal Mountains	0.0300	Apr 25 2007	0.045	moderate	oxic	22	271	0.0%	0.0%	100.0%
SF-24	Central California Foothills and Coastal Mountains	0.0300	Apr 24 2007	0.030	low	oxic	31	238	0.0%	0.0%	100.0%
SF-25[2]	Central California Foothills and Coastal Mountains	0.0300	May 1 2007	0.040	moderate	oxic	30	187	39.7%	0.0%	60.3%
SF-26[2]	Central California Foothills and Coastal Mountains	0.0300	May 1 2007	0.024	low	oxic	52	130	1.8%	0.0%	98.2%
SF-27[2]	Central California Foothills and Coastal Mountains	0.0300	May 1 2007	0.023	low	oxic	49	133	1.4%	0.0%	98.6%
SF-28	Central California Foothills and Coastal Mountains	0.0300	Apr 23 2007	0.045	moderate	oxic	37	158	8.2%	0.5%	91.3%
SF-29[2]	Central California Foothills and Coastal Mountains	0.0300	Apr 23 2007	0.033	moderate	oxic	54	84	0.0%	0.0%	100.0%
SF-30[2]	Central California Foothills and Coastal Mountains	0.0300	Jun 6 2007	0.021	low	oxic	291	24	12.1%	64.7%	23.3%
SF-31[2]	Central California Foothills and Coastal Mountains	0.0300	Apr 23 2007	0.039	moderate	oxic	71	87	9.1%	14.6%	76.3%
SF-32[2]	Central California Foothills and Coastal Mountains	0.0300	Apr 24 2007	0.070	moderate	oxic	76	na	4.6%	79.0%	16.4%
SF-33[2]	Central California Foothills and Coastal Mountains	0.0300	Apr 30 2007	0.116	high	oxic	116	112	19.1%	7.7%	73.2%
SF-34[2]	Central California Foothills and Coastal Mountains	0.0300	Jun 5 2007	0.030	low	anoxic	16	47	4.1%	18.3%	77.6%
SF-35	Central California Foothills and Coastal Mountains	0.0300	Jun 12 2007	0.095	moderate	anoxic	10	76	0.0%	0.0%	100.0%
SF-36[2]	Central California Foothills and Coastal Mountains	0.0300	Jun 4 2007	0.022	low	oxic	20	58	7.3%	1.8%	90.9%
SF-37[2]	Central California Foothills and Coastal Mountains	0.0300	Jun 5 2007	0.022	low	anoxic	21	61	4.6%	0.0%	95.4%
SF-38[2]	Central California Foothills and Coastal Mountains	0.0300	Jun 21 2007	0.117	high	anoxic	4	163	1.4%	1.4%	97.3%

Status and trends of orthophosphate concentrations in groundwater used for public supply in California *Environmental Monitoring and Assessment, Robert Kent, Tyler D. Johnson, and Michael R. Rosen, U.S. Geological Survey California Water Science Center-rhkent@usgs.gov*

Online resource (suppplementary table) 2. Selected attributes of GAMA-PBP (https://ca.water.usgs.gov/gama/) status wells sampled for orthophosphate concentration-page 21.

GAMA-PBP ID	Agricultural land use in 1982[9] (percent)	Natural land use in 1982[9] (percent)	Urban land use in 1982[9] (percent)	Agricultural land use in 1992[9] (percent)	Natural land use in 1992[9] (percent)	Urban land use in 1992[9] (percent)	Agricultural land use in 2002[9] (percent)	Natural land use in 2002[9] (percent)	Urban land use in 2002[9] (percent)	Agricultural land use in 2012[9] (percent)	Natural land use in 2012[9] (percent)	Urban land use in 2012[9] (percent)	Age Classification[10]	Septic Tanks[11]	Aridity[12]
SF-09[2]	0.0%	0.0%	100.0%	0.0%	0.0%	100.0%	0.0%	0.0%	100.0%	0.0%	0.0%	100.0%	Mixed	15.70	0.370
SF-10[2]	0.0%	0.0%	100.0%	0.0%	0.0%	100.0%	0.0%	0.0%	100.0%	0.0%	0.0%	100.0%	Mixed	22.44	0.310
SF-11[2]	0.0%	0.0%	100.0%	0.0%	0.0%	100.0%	0.0%	0.0%	100.0%	0.0%	0.0%	100.0%	Modern	5.55	0.355
SF-12[2]	0.0%	0.0%	100.0%	0.0%	0.0%	100.0%	0.0%	0.0%	100.0%	0.0%	0.0%	100.0%	Modern	8.06	0.372
SF-13[2]	0.0%	0.0%	100.0%	0.0%	0.0%	100.0%	0.0%	0.0%	100.0%	0.0%	0.0%	100.0%	Mixed	6.03	0.346
SF-14[2]	0.0%	0.0%	100.0%	0.0%	0.0%	100.0%	0.0%	0.0%	100.0%	0.0%	0.0%	100.0%	Mixed	4.76	0.321
SF-15[2]	0.0%	0.0%	100.0%	0.0%	0.0%	100.0%	0.0%	0.0%	100.0%	0.0%	0.0%	100.0%	Premodern	0.01	0.314
SF-16[2]	0.9%	0.0%	99.1%	0.0%	0.0%	100.0%	0.0%	0.0%	100.0%	0.0%	0.0%	100.0%	Mixed	0.00	0.312
SF-17[2]	14.4%	6.0%	79.6%	0.0%	2.8%	97.2%	0.0%	2.3%	97.7%	0.0%	2.3%	97.7%	Mixed	0.77	0.315
SF-18[2]	0.0%	0.0%	100.0%	0.0%	0.0%	100.0%	0.0%	0.0%	100.0%	0.0%	0.0%	100.0%	Mixed	0.11	0.336
SF-19[2]	0.0%	0.0%	100.0%	0.0%	0.0%	100.0%	0.0%	0.0%	100.0%	0.0%	0.0%	100.0%	Premodern	16.26	0.460
SF-20[2]	0.0%	0.0%	100.0%	0.0%	0.0%	100.0%	0.0%	0.0%	100.0%	0.0%	0.0%	100.0%	Modern	34.22	0.378
SF-21[2]	0.0%	0.0%	100.0%	0.0%	0.0%	100.0%	0.0%	0.0%	100.0%	0.0%	0.0%	100.0%	Modern	3.21	0.347
SF-22[2]	0.0%	0.0%	100.0%	0.0%	0.0%	100.0%	0.0%	0.0%	100.0%	0.0%	0.0%	100.0%	Modern	9.85	0.348
SF-23[2]	0.0%	0.0%	100.0%	0.0%	0.0%	100.0%	0.0%	0.0%	100.0%	0.0%	0.0%	100.0%	Mixed	0.00	0.313
SF-24	0.0%	0.0%	100.0%	0.0%	0.0%	100.0%	0.0%	0.0%	100.0%	0.0%	0.0%	100.0%	Mixed	2.51	0.319
SF-25[2]	11.4%	0.0%	88.6%	0.0%	0.0%	100.0%	0.0%	0.0%	100.0%	0.0%	0.0%	100.0%	Mixed	4.76	0.318
SF-26[2]	1.4%	0.0%	98.6%	0.0%	0.0%	100.0%	0.0%	0.0%	100.0%	0.0%	0.0%	100.0%	Modern	0.13	0.370
SF-27[2]	0.0%	0.0%	100.0%	0.0%	0.0%	100.0%	0.0%	0.0%	100.0%	0.0%	0.0%	100.0%	Modern	8.61	0.346
SF-28	8.2%	0.5%	91.3%	0.0%	0.5%	99.5%	0.0%	0.5%	99.5%	0.0%	0.5%	99.5%	Modern	0.95	0.334
SF-29[2]	0.0%	0.0%	100.0%	0.0%	0.0%	100.0%	0.0%	0.0%	100.0%	0.0%	0.0%	100.0%	Modern	0.78	0.370
SF-30[2]	11.6%	65.1%	23.3%	8.8%	43.7%	47.4%	4.2%	46.5%	49.3%	4.2%	45.1%	50.7%	Mixed	5.05	0.469
SF-31[2]	2.7%	14.6%	82.6%	0.0%	14.6%	85.4%	0.0%	14.6%	85.4%	0.0%	14.6%	85.4%	Modern	0.00	0.407
SF-32[2]	4.6%	51.6%	43.8%	4.1%	40.6%	55.3%	4.1%	34.2%	61.6%	4.1%	29.7%	66.2%	Modern	3.04	0.419
SF-33[2]	15.5%	7.3%	77.3%	14.1%	6.8%	79.1%	11.8%	6.8%	81.4%	11.8%	5.5%	82.7%	Mixed	11.06	0.433
SF-34[2]	3.2%	18.3%	78.5%	0.0%	18.3%	81.7%	0.0%	17.8%	82.2%	0.0%	17.8%	82.2%	Mixed	0.15	0.354
SF-35	0.0%	0.0%	100.0%	0.0%	0.0%	100.0%	0.0%	0.0%	100.0%	0.0%	0.0%	100.0%	Mixed	0.00	0.340
SF-36[2]	0.0%	1.8%	98.2%	0.0%	1.8%	98.2%	0.0%	1.8%	98.2%	0.0%	1.8%	98.2%	Modern	9.67	0.369
SF-37[2]	0.0%	0.0%	100.0%	0.0%	0.0%	100.0%	0.0%	0.0%	100.0%	0.0%	0.0%	100.0%	Modern	13.52	0.374
SF-38[2]	0.0%	1.4%	98.6%	0.0%	1.4%	98.6%	0.0%	1.4%	98.6%	0.0%	1.4%	98.6%	Premodern	0.36	0.374

Online resource (suppplementary table) 2. Selected attributes of GAMA-PBP (https://ca.water.usgs.gov/gama/) status wells sampled for orthophosphate concentration-page 22

GAMA-PBP ID	USGS Station ID[1]	GAMA-PBP study unit	GAMA-PBP study area[3]	Hydrogeologic zone
SF-39[2]	373832122064801	San Francisco Bay	South San Francisco Bay basins	Coastal
SF-40[2]	373930122054701	San Francisco Bay	South San Francisco Bay basins	Coastal
SF-41[2]	374008122072001	San Francisco Bay	South San Francisco Bay basins	Coastal
SF-42[2]	374504122112201	San Francisco Bay	South San Francisco Bay basins	Coastal
SF-43[2]	374716122175701	San Francisco Bay	South San Francisco Bay basins	Coastal
NSFVOL-01[2]	382849122420501	North San Francisco Bay hydrologic provinces	Sonoma Volcanic Highlands	Coastal
NSFVOL-14	381639122150801	North San Francisco Bay hydrologic provinces	Sonoma Volcanic Highlands	Coastal
NSFVOL-19	383241122282101	North San Francisco Bay hydrologic provinces	Sonoma Volcanic Highlands	Coastal
NSFVOL-20	381906122274901	North San Francisco Bay hydrologic provinces	Sonoma Volcanic Highlands	Coastal
NSFVP-10[2]	383034122590701	North San Francisco Bay hydrologic provinces	North San Francisco Bay Valley and Plain	Coastal
NSFVP-19[2]	383630122512601	North San Francisco Bay hydrologic provinces	North San Francisco Bay Valley and Plain	Coastal
NSFVP-26	383916122473501	North San Francisco Bay hydrologic provinces	North San Francisco Bay Valley and Plain	Coastal
NSFVP-29	384238122541201	North San Francisco Bay hydrologic provinces	North San Francisco Bay Valley and Plain	Coastal
NSFVP-30[2]	384628122592701	North San Francisco Bay hydrologic provinces	North San Francisco Bay Valley and Plain	Coastal
NSFVP-34	382307122311301	North San Francisco Bay hydrologic provinces	North San Francisco Bay Valley and Plain	Coastal
NSFVP-37	381808122293801	North San Francisco Bay hydrologic provinces	North San Francisco Bay Valley and Plain	Coastal
NSFVP-38	381544122263801	North San Francisco Bay hydrologic provinces	North San Francisco Bay Valley and Plain	Coastal
NSFVP-40[2]	381516122170001	North San Francisco Bay hydrologic provinces	North San Francisco Bay Valley and Plain	Coastal
NSFVP-45[2]	382109122201001	North San Francisco Bay hydrologic provinces	North San Francisco Bay Valley and Plain	Coastal
NSFVP-46	382720122245701	North San Francisco Bay hydrologic provinces	North San Francisco Bay Valley and Plain	Coastal
NSFVP-49	381657122165301	North San Francisco Bay hydrologic provinces	North San Francisco Bay Valley and Plain	Coastal
NSFVP-50	383008122275201	North San Francisco Bay hydrologic provinces	North San Francisco Bay Valley and Plain	Coastal
NSFWG-08	382352122493301	North San Francisco Bay hydrologic provinces	Wilson Grove Formation Highlands	Coastal
NSFWG-10	381455122534001	North San Francisco Bay hydrologic provinces	Wilson Grove Formation Highlands	Coastal
NSFWGFP-01	382345122490701	North San Francisco Bay hydrologic provinces	Wilson Grove Formation Highlands	Coastal
HR-SC-01[2]	373300122290001	Santa Cruz, San Gabriel, and Peninsular Ranges Hard Rock Aquifers	Hard Rock Santa Cruz	Coastal
HR-SC-02[2]	371700122220001	Santa Cruz, San Gabriel, and Peninsular Ranges Hard Rock Aquifers	Hard Rock Santa Cruz	Coastal
HR-SC-03[2]	370900122190001	Santa Cruz, San Gabriel, and Peninsular Ranges Hard Rock Aquifers	Hard Rock Santa Cruz	Coastal
HR-SC-04[2]	372200122150001	Santa Cruz, San Gabriel, and Peninsular Ranges Hard Rock Aquifers	Hard Rock Santa Cruz	Coastal
HR-SC-05[2]	370700122100001	Santa Cruz, San Gabriel, and Peninsular Ranges Hard Rock Aquifers	Hard Rock Santa Cruz	Coastal

Online resource (suppplementary table) 2. Selected attributes of GAMA-PBP (https://ca.water.usgs.gov/gama/) status wells sampled for orthophosphate concentration-page 23.

GAMA-PBP ID	USEPA Level III Ecoregions[4]	Level III Ecoregion Reference Concentration[4]	Status Sample Date	Status Sample Orthophosphate Concentration (mg/L as P)	Relative Concentration Category[5]	Redox state[6]	Elevation of LSD (meters above NAVD 88)[7]	Well depth (meters below LSD)[8]	Agricultural land use in 1974[9] (percent)	Natural land use in 1974[9] (percent)	Urban land use in 1974[9] (percent)
SF-39[2]	Central California Foothills and Coastal Mountains	0.0300	Jun 19 2007	0.094	moderate	anoxic	13	183	0.0%	0.0%	100.0%
SF-40[2]	Central California Foothills and Coastal Mountains	0.0300	Jun 21 2007	0.022	low	anoxic	21	168	0.0%	0.0%	100.0%
SF-41[2]	Central California Foothills and Coastal Mountains	0.0300	Jun 21 2007	0.006	low	oxic	12	47	0.0%	0.0%	100.0%
SF-42[2]	Central California Foothills and Coastal Mountains	0.0300	Jun 19 2007	0.027	low	oxic	4	151	0.0%	0.0%	100.0%
SF-43[2]	Central California Foothills and Coastal Mountains	0.0300	Jun 20 2007	0.092	moderate	oxic	4	108	0.0%	26.5%	73.5%
NSFVOL-01[2]	Central California Foothills and Coastal Mountains	0.0300	Sep 15 2004	0.191	high	oxic	100	98	0.0%	14.6%	85.4%
NSFVOL-14	Central California Foothills and Coastal Mountains	0.0300	Oct 7 2004	0.181	high	oxic	43	127	9.1%	10.5%	80.4%
NSFVOL-19	Central California Foothills and Coastal Mountains	0.0300	Nov 2 2004	0.059	moderate	oxic	239	215	0.5%	29.2%	70.3%
NSFVOL-20	Central California Foothills and Coastal Mountains	0.0300	Nov 4 2004	0.061	moderate	oxic	92	76	0.0%	57.4%	42.6%
NSFVP-10[2]	Coast Range	0.0103	Sep 13 2004	0.019	moderate	anoxic	12	30	0.0%	10.1%	89.9%
NSFVP-19[2]	Central California Foothills and Coastal Mountains	0.0300	Sep 16 2004	0.021	low	anoxic	26	30	4.7%	27.4%	67.9%
NSFVP-26	Central California Foothills and Coastal Mountains	0.0300	Sep 27 2004	0.090	moderate	anoxic	59	183	77.2%	11.9%	11.0%
NSFVP-29	Central California Foothills and Coastal Mountains	0.0300	Sep 28 2004	0.010	low	oxic	64	37	58.6%	6.4%	35.0%
NSFVP-30[2]	Central California Foothills and Coastal Mountains	0.0300	Sep 29 2004	0.034	moderate	oxic	127	na	45.7%	43.4%	11.0%
NSFVP-34	Central California Foothills and Coastal Mountains	0.0300	Oct 18 2004	0.318	high	anoxic	98	79	35.0%	35.5%	29.5%
NSFVP-37	Central California Foothills and Coastal Mountains	0.0300	Oct 19 2004	0.112	high	oxic	37	110	0.9%	0.9%	98.2%
NSFVP-38	Central California Foothills and Coastal Mountains	0.0300	Oct 20 2004	0.146	high	anoxic	10	235	30.0%	11.8%	58.2%
NSFVP-40[2]	Central California Foothills and Coastal Mountains	0.0300	Oct 21 2004	0.024	low	anoxic	5	na	0.9%	40.7%	58.3%
NSFVP-45[2]	Central California Foothills and Coastal Mountains	0.0300	Nov 2 2004	0.023	low	anoxic	31	na	76.4%	10.0%	13.6%
NSFVP-46	Central California Foothills and Coastal Mountains	0.0300	Nov 3 2004	0.012	low	oxic	47	55	81.9%	7.2%	10.9%
NSFVP-49	Central California Foothills and Coastal Mountains	0.0300	Nov 17 2004	0.559	high	anoxic	4	67	0.0%	6.8%	93.2%
NSFVP-50	Central California Foothills and Coastal Mountains	0.0300	Nov 18 2004	0.329	high	oxic	70	61	36.2%	0.0%	63.8%
NSFWG-08	Central California Foothills and Coastal Mountains	0.0300	Oct 4 2004	0.053	moderate	oxic	35	183	0.0%	0.0%	100.0%
NSFWG-10	Central California Foothills and Coastal Mountains	0.0300	Oct 6 2004	0.027	low	anoxic	39	58	0.0%	91.8%	8.2%
NSFWGFP-01	Central California Foothills and Coastal Mountains	0.0300	Oct 5 2004	0.008	low	anoxic	25	161	9.4%	10.3%	80.3%
HR-SC-01[2]	Coast Range	0.0103	Mar 28 2011	0.006	low	oxic	147	237	1.4%	15.9%	82.7%
HR-SC-02[2]	Coast Range	0.0103	Mar 29 2011	0.096	moderate	oxic	147	0	0.0%	100.0%	0.0%
HR-SC-03[2]	Coast Range	0.0103	Mar 29 2011	0.457	high	anoxic	110	43	20.7%	79.3%	0.0%
HR-SC-04[2]	Coast Range	0.0103	Mar 30 2011	0.007	low	oxic	544	91	0.0%	1.4%	98.6%
HR-SC-05[2]	Coast Range	0.0103	Mar 31 2011	0.004	low	anoxic	747	94	0.0%	95.9%	4.1%

Status and trends of orthophosphate concentrations in groundwater used for public supply in California *Environmental Monitoring and Assessment, Robert Kent, Tyler D. Johnson, and Michael R. Rosen, U.S. Geological Survey California Water Science Center-rhkent@usgs.gov*

Online resource (suppplementary table) 2. Selected attributes of GAMA-PBP (https://ca.water.usgs.gov/gama/) status wells sampled for orthophosphate concentration-page 24.

GAMA-PBP ID	Agricultural land use in 1982[9] (percent)	Natural land use in 1982[9] (percent)	Urban land use in 1982[9] (percent)	Agricultural land use in 1992[9] (percent)	Natural land use in 1992[9] (percent)	Urban land use in 1992[9] (percent)	Agricultural land use in 2002[9] (percent)	Natural land use in 2002[9] (percent)	Urban land use in 2002[9] (percent)	Agricultural land use in 2012[9] (percent)	Natural land use in 2012[9] (percent)	Urban land use in 2012[9] (percent)	Age Classification[10]	Septic Tanks[11]	Aridity[12]
SF-39[2]	0.0%	0.0%	100.0%	0.0%	0.0%	100.0%	0.0%	0.0%	100.0%	0.0%	0.0%	100.0%	Premodern	69.38	0.399
SF-40[2]	0.0%	0.0%	100.0%	0.0%	0.0%	100.0%	0.0%	0.0%	100.0%	0.0%	0.0%	100.0%	Mixed	2.44	0.428
SF-41[2]	0.0%	0.0%	100.0%	0.0%	0.0%	100.0%	0.0%	0.0%	100.0%	0.0%	0.0%	100.0%	ModernOrMixed	5.17	0.421
SF-42[2]	0.0%	0.0%	100.0%	0.0%	0.0%	100.0%	0.0%	0.0%	100.0%	0.0%	0.0%	100.0%	Premodern	0.00	0.450
SF-43[2]	0.0%	26.5%	73.5%	0.0%	26.5%	73.5%	0.0%	26.5%	73.5%	0.0%	26.5%	73.5%	Premodern	0.41	0.443
NSFVOL-01[2]	0.0%	0.0%	100.0%	0.0%	0.0%	100.0%	0.0%	0.0%	100.0%	0.0%	0.0%	100.0%	Premodern	14.73	0.821
NSFVOL-14	7.3%	10.5%	82.2%	7.8%	9.6%	82.6%	6.8%	9.6%	83.6%	6.8%	9.6%	83.6%	Modern	2.58	0.611
NSFVOL-19	0.5%	0.9%	98.6%	0.0%	0.9%	99.1%	0.0%	0.9%	99.1%	0.0%	0.5%	99.5%	Premodern	28.43	0.735
NSFVOL-20	0.0%	56.9%	43.1%	0.0%	56.9%	43.1%	0.0%	56.9%	43.1%	0.0%	56.9%	43.1%	Mixed	6.42	0.672
NSFVP-10[2]	0.0%	10.1%	89.9%	0.0%	10.1%	89.9%	0.0%	10.1%	89.9%	0.0%	10.1%	89.9%	Modern	13.75	1.085
NSFVP-19[2]	0.5%	13.2%	86.3%	0.0%	8.5%	91.5%	0.0%	8.5%	91.5%	0.0%	8.5%	91.5%	Modern	19.62	0.853
NSFVP-26	68.9%	11.0%	20.1%	58.4%	10.5%	31.1%	48.9%	10.0%	41.1%	48.9%	6.8%	44.3%	Premodern	4.27	0.846
NSFVP-29	59.1%	5.5%	35.5%	59.1%	5.5%	35.5%	59.1%	5.5%	35.5%	59.5%	5.0%	35.5%	Modern	5.71	0.873
NSFVP-30[2]	62.1%	26.9%	11.0%	42.5%	45.7%	11.9%	52.5%	36.1%	11.4%	52.5%	36.1%	11.4%	Mixed	2.22	0.882
NSFVP-34	35.0%	30.9%	34.1%	26.8%	36.8%	36.4%	30.0%	25.5%	44.5%	30.0%	25.5%	44.5%	Premodern	11.79	0.778
NSFVP-37	1.8%	0.5%	97.7%	0.0%	0.5%	99.5%	0.0%	0.5%	99.5%	0.0%	0.5%	99.5%	Modern	62.54	0.604
NSFVP-38	28.6%	11.4%	60.0%	27.3%	11.4%	61.4%	26.8%	11.4%	61.8%	26.8%	8.2%	65.0%	Premodern	20.98	0.564
NSFVP-40[2]	0.9%	40.7%	58.3%	0.9%	40.7%	58.3%	0.9%	40.7%	58.3%	0.9%	36.6%	62.5%	Premodern	0.42	0.526
NSFVP-45[2]	75.5%	9.5%	15.0%	75.5%	9.1%	15.5%	75.5%	9.1%	15.5%	75.5%	9.1%	15.5%	Mixed	7.81	0.585
NSFVP-46	81.0%	7.2%	11.8%	81.4%	6.3%	12.2%	81.0%	6.3%	12.7%	81.0%	6.3%	12.7%	ModernOrMixed	8.74	0.703
NSFVP-49	0.0%	6.8%	93.2%	0.0%	6.8%	93.2%	0.0%	6.8%	93.2%	0.0%	6.8%	93.2%	Premodern	0.00	0.548
NSFVP-50	14.0%	0.0%	86.0%	10.9%	0.0%	89.1%	10.9%	0.0%	89.1%	10.9%	0.0%	89.1%	Premodern	22.37	0.720
NSFWG-08	0.0%	0.0%	100.0%	0.0%	0.0%	100.0%	0.0%	0.0%	100.0%	0.0%	0.0%	100.0%	Mixed	8.37	0.829
NSFWG-10	0.0%	91.8%	8.2%	0.0%	91.3%	8.7%	0.0%	91.3%	8.7%	0.0%	91.3%	8.7%	Premodern	1.79	0.811
NSFWGFP-01	9.9%	9.9%	80.3%	9.9%	9.9%	80.3%	9.9%	9.9%	80.3%	9.9%	9.4%	80.8%	Mixed	33.67	0.829
HR-SC-01[2]	1.4%	14.5%	84.1%	1.4%	14.5%	84.1%	1.4%	14.5%	84.1%	1.4%	14.5%	84.1%	na	0.85	0.672
HR-SC-02[2]	0.0%	100.0%	0.0%	0.0%	100.0%	0.0%	0.0%	100.0%	0.0%	0.0%	100.0%	0.0%	na	2.03	0.613
HR-SC-03[2]	20.7%	79.3%	0.0%	20.7%	79.3%	0.0%	7.4%	92.6%	0.0%	7.4%	92.6%	0.0%	na	2.59	0.650
HR-SC-04[2]	0.0%	1.4%	98.6%	0.0%	1.4%	98.6%	0.0%	1.4%	98.6%	0.0%	1.4%	98.6%	na	68.27	0.839
HR-SC-05[2]	0.0%	95.9%	4.1%	0.0%	95.4%	4.6%	0.0%	95.4%	4.6%	0.0%	95.4%	4.6%	na	3.44	1.426

Online resource (suppplementary table) 2. Selected attributes of GAMA-PBP (https://ca.water.usgs.gov/gama/) status wells sampled for orthophosphate concentration-page 25

GAMA-PBP ID	USGS Station ID[1]	GAMA-PBP study unit	GAMA-PBP study area[3]	Hydrogeologic zone
HR-SC-06[2]	371300122140001	Santa Cruz, San Gabriel, and Peninsular Ranges Hard Rock Aquifers	Hard Rock Santa Cruz	Coastal
HR-SC-07[2]	372200122230001	Santa Cruz, San Gabriel, and Peninsular Ranges Hard Rock Aquifers	Hard Rock Santa Cruz	Coastal
HR-SC-08[2]	371100122010001	Santa Cruz, San Gabriel, and Peninsular Ranges Hard Rock Aquifers	Hard Rock Santa Cruz	Coastal
HR-SC-09[2]	371000121580001	Santa Cruz, San Gabriel, and Peninsular Ranges Hard Rock Aquifers	Hard Rock Santa Cruz	Coastal
HR-SC-10[2]	371400122060001	Santa Cruz, San Gabriel, and Peninsular Ranges Hard Rock Aquifers	Hard Rock Santa Cruz	Coastal
HR-SC-11[2]	371700122060001	Santa Cruz, San Gabriel, and Peninsular Ranges Hard Rock Aquifers	Hard Rock Santa Cruz	Coastal
HR-SC-12[2]	371000121460001	Santa Cruz, San Gabriel, and Peninsular Ranges Hard Rock Aquifers	Hard Rock Santa Cruz	Coastal
HR-SC-13[2]	370500121470002	Santa Cruz, San Gabriel, and Peninsular Ranges Hard Rock Aquifers	Hard Rock Santa Cruz	Coastal
HR-SC-14[2]	370800121580001	Santa Cruz, San Gabriel, and Peninsular Ranges Hard Rock Aquifers	Hard Rock Santa Cruz	Coastal
HR-SC-15[2]	370100122050001	Santa Cruz, San Gabriel, and Peninsular Ranges Hard Rock Aquifers	Hard Rock Santa Cruz	Coastal
HR-SC-16[2]	370800122090001	Santa Cruz, San Gabriel, and Peninsular Ranges Hard Rock Aquifers	Hard Rock Santa Cruz	Coastal
HR-SC-17[2]	370545122033101	Santa Cruz, San Gabriel, and Peninsular Ranges Hard Rock Aquifers	Hard Rock Santa Cruz	Coastal
HR-SC-18[2]	370200122020001	Santa Cruz, San Gabriel, and Peninsular Ranges Hard Rock Aquifers	Hard Rock Santa Cruz	Coastal
HR-SC-19[2]	370325122132201	Santa Cruz, San Gabriel, and Peninsular Ranges Hard Rock Aquifers	Hard Rock Santa Cruz	Coastal
HR-SC-20[2]	371900122230001	Santa Cruz, San Gabriel, and Peninsular Ranges Hard Rock Aquifers	Hard Rock Santa Cruz	Coastal
HR-SC-21[2]	371800122150001	Santa Cruz, San Gabriel, and Peninsular Ranges Hard Rock Aquifers	Hard Rock Santa Cruz	Coastal
HR-SC-22[2]	370200121450001	Santa Cruz, San Gabriel, and Peninsular Ranges Hard Rock Aquifers	Hard Rock Santa Cruz	Coastal
HR-SC-23[2]	370000121420001	Santa Cruz, San Gabriel, and Peninsular Ranges Hard Rock Aquifers	Hard Rock Santa Cruz	Coastal
HR-SC-24[2]	365400121350001	Santa Cruz, San Gabriel, and Peninsular Ranges Hard Rock Aquifers	Hard Rock Santa Cruz	Coastal
HR-SC-25[2]	370400122040001	Santa Cruz, San Gabriel, and Peninsular Ranges Hard Rock Aquifers	Hard Rock Santa Cruz	Coastal
CE-QPC-01	374526120475501	Central Eastside San Joaquin Basin	Central-Eastside San Joaquin Valley QPc area	Central Valley
CE-QPC-07	373200120270001	Central Eastside San Joaquin Basin	Central-Eastside San Joaquin Valley QPc area	Central Valley
CE-QPC-09	374801120384001	Central Eastside San Joaquin Basin	Central-Eastside San Joaquin Valley QPc area	Central Valley
MER-02[2]	372300120420001	Central Eastside San Joaquin Basin	Merced subbasin	Central Valley
MER-03	372100120360001	Central Eastside San Joaquin Basin	Merced subbasin	Central Valley
MER-09	371928120263302	Central Eastside San Joaquin Basin	Merced subbasin	Central Valley
MER-10	371829120300801	Central Eastside San Joaquin Basin	Merced subbasin	Central Valley
MER-11	371300120150001	Central Eastside San Joaquin Basin	Merced subbasin	Central Valley
MER-12	371100120370001	Central Eastside San Joaquin Basin	Merced subbasin	Central Valley
MER-20[2]	371100120080001	Central Eastside San Joaquin Basin	Merced subbasin	Central Valley

Online resource (suppplementary table) 2. Selected attributes of GAMA-PBP (https://ca.water.usgs.gov/gama/) status wells sampled for orthophosphate concentration-page 26.

GAMA-PBP ID	USEPA Level III Ecoregions[4]	Level III Ecoregion Reference Concentration[4]	Status Sample Date	Status Sample Orthophosphate Concentration (mg/L as P)	Relative Concentration Category[5]	Redox state[6]	Elevation of LSD (meters above NAVD 88)[7]	Well depth (meters below LSD)[8]	Agricultural land use in 1974[9] (percent)	Natural land use in 1974[9] (percent)	Urban land use in 1974[9] (percent)
HR-SC-06[2]	Coast Range	0.0103	Apr 4 2011	0.012	moderate	oxic	635	79	0.0%	99.5%	0.5%
HR-SC-07[2]	Coast Range	0.0103	Apr 4 2011	0.025	moderate	anoxic	136	91	0.0%	100.0%	0.0%
HR-SC-08[2]	Coast Range	0.0103	Apr 5 2011	0.036	moderate	oxic	426	9	3.8%	85.9%	10.3%
HR-SC-09[2]	Central California Foothills and Coastal Mountains	0.0300	Apr 5 2011	0.007	low	anoxic	222	128	0.0%	81.7%	18.3%
HR-SC-10[2]	Coast Range	0.0103	Apr 6 2011	0.166	high	oxic	897	61	0.9%	91.7%	7.4%
HR-SC-11[2]	Central California Foothills and Coastal Mountains	0.0300	Apr 6 2011	0.008	low	oxic	512	34	0.0%	97.2%	2.8%
HR-SC-12[2]	Central California Foothills and Coastal Mountains	0.0300	Apr 7 2011	0.013	low	anoxic	252	69	0.0%	100.0%	0.0%
HR-SC-13[2]	Central California Foothills and Coastal Mountains	0.0300	Apr 18 2011	0.013	low	oxic	305	49	0.0%	96.8%	3.2%
HR-SC-14[2]	Coast Range	0.0103	Apr 19 2011	0.172	high	anoxic	542	55	0.0%	4.1%	95.9%
HR-SC-15[2]	Coast Range	0.0103	Apr 19 2011	0.012	moderate	anoxic	190	98	0.0%	4.5%	95.5%
HR-SC-16[2]	Coast Range	0.0103	Apr 20 2011	0.013	moderate	oxic	348	89	0.0%	7.8%	92.2%
HR-SC-17[2]	Coast Range	0.0103	Apr 20 2011	0.219	high	oxic	204	0	0.0%	1.9%	98.1%
HR-SC-18[2]	Coast Range	0.0103	Apr 21 2011	0.672	high	oxic	234	241	0.0%	18.3%	81.7%
HR-SC-19[2]	Coast Range	0.0103	Apr 21 2011	0.265	high	anoxic	60	55	0.0%	99.1%	0.9%
HR-SC-20[2]	Coast Range	0.0103	May 2 2011	0.048	moderate	oxic	23	20	17.3%	70.5%	12.3%
HR-SC-21[2]	Coast Range	0.0103	May 2 2011	0.143	high	anoxic	271	71	0.0%	81.3%	18.7%
HR-SC-22[2]	Central California Foothills and Coastal Mountains	0.0300	May 3 2011	0.024	low	anoxic	626	140	0.0%	100.0%	0.0%
HR-SC-23[2]	Central California Foothills and Coastal Mountains	0.0300	May 3 2011	0.031	moderate	anoxic	513	146	0.5%	88.6%	11.0%
HR-SC-24[2]	Central California Foothills and Coastal Mountains	0.0300	May 4 2011	0.063	moderate	oxic	41	27	0.0%	71.2%	28.8%
HR-SC-25[2]	Coast Range	0.0103	May 5 2011	0.878	high	oxic	160	53	0.0%	0.9%	99.1%
CE-QPC-01	Central California Valley	0.0770	Mar 20 2006	0.022	low	oxic	80	85	49.3%	28.2%	22.5%
CE-QPC-07	Central California Valley	0.0770	Apr 20 2006	0.069	low	oxic	97	116	18.3%	81.7%	0.0%
CE-QPC-09	Central California Valley	0.0770	May 3 2006	0.110	high	oxic	106	30	11.9%	88.1%	0.0%
MER-02[2]	Central California Valley	0.0770	Mar 29 2006	0.018	low	oxic	43	na	84.1%	7.0%	8.9%
MER-03	Central California Valley	0.0770	Mar 30 2006	0.018	low	oxic	48	160	12.0%	0.0%	88.0%
MER-09	Central California Valley	0.0770	Apr 10 2006	0.040	low	oxic	57	81	42.1%	3.2%	54.6%
MER-10	Central California Valley	0.0770	Apr 11 2006	0.023	low	oxic	51	90	12.3%	0.0%	87.7%
MER-11	Central California Valley	0.0770	Apr 12 2006	0.030	low	oxic	78	192	55.0%	1.8%	43.2%
MER-12	Central California Valley	0.0770	Apr 13 2006	0.029	low	anoxic	33	64	56.2%	43.3%	0.5%
MER-20[2]	Central California Valley	0.0770	May 1 2006	0.016	low	anoxic	99	281	41.4%	58.6%	0.0%

Online resource (suppplementary table) 2. Selected attributes of GAMA-PBP (https://ca.water.usgs.gov/gama/) status wells sampled for orthophosphate concentration-page 27.

GAMA-PBP ID	Agricultural land use in 1982[9] (percent)	Natural land use in 1982[9] (percent)	Urban land use in 1982[9] (percent)	Agricultural land use in 1992[9] (percent)	Natural land use in 1992[9] (percent)	Urban land use in 1992[9] (percent)	Agricultural land use in 2002[9] (percent)	Natural land use in 2002[9] (percent)	Urban land use in 2002[9] (percent)	Agricultural land use in 2012[9] (percent)	Natural land use in 2012[9] (percent)	Urban land use in 2012[9] (percent)	Age Classification[10]	Septic Tanks[11]	Aridity[12]
HR-SC-06[2]	0.0%	99.5%	0.5%	0.0%	99.5%	0.5%	0.0%	99.5%	0.5%	0.0%	99.5%	0.5%	na	1.81	0.956
HR-SC-07[2]	0.0%	100.0%	0.0%	0.0%	100.0%	0.0%	0.0%	100.0%	0.0%	0.0%	100.0%	0.0%	na	1.93	0.632
HR-SC-08[2]	3.8%	85.9%	10.3%	3.8%	26.8%	69.5%	3.8%	26.8%	69.5%	3.8%	26.8%	69.5%	na	23.23	0.867
HR-SC-09[2]	0.0%	81.7%	18.3%	0.0%	81.7%	18.3%	0.0%	81.7%	18.3%	0.0%	81.7%	18.3%	na	2.73	0.830
HR-SC-10[2]	15.2%	76.5%	8.3%	0.5%	91.2%	8.3%	2.3%	88.9%	8.8%	2.3%	88.9%	8.8%	na	3.38	1.071
HR-SC-11[2]	0.0%	97.2%	2.8%	0.0%	97.2%	2.8%	0.0%	97.2%	2.8%	0.0%	97.2%	2.8%	na	1.42	0.709
HR-SC-12[2]	0.0%	100.0%	0.0%	0.0%	100.0%	0.0%	0.0%	100.0%	0.0%	0.0%	100.0%	0.0%	na	3.29	0.582
HR-SC-13[2]	0.0%	96.8%	3.2%	0.0%	96.8%	3.2%	0.0%	96.8%	3.2%	0.0%	96.8%	3.2%	na	2.71	0.876
HR-SC-14[2]	0.0%	1.4%	98.6%	0.0%	1.4%	98.6%	0.0%	1.4%	98.6%	0.0%	1.4%	98.6%	na	57.83	0.906
HR-SC-15[2]	0.0%	4.5%	95.5%	0.0%	4.5%	95.5%	0.0%	4.5%	95.5%	0.0%	4.5%	95.5%	na	121.84	1.006
HR-SC-16[2]	0.0%	0.0%	100.0%	0.0%	0.0%	100.0%	0.0%	0.0%	100.0%	0.0%	0.0%	100.0%	na	23.24	1.389
HR-SC-17[2]	0.0%	1.9%	98.1%	0.0%	1.9%	98.1%	0.0%	1.9%	98.1%	0.0%	1.9%	98.1%	na	54.10	0.973
HR-SC-18[2]	0.0%	17.8%	82.2%	0.0%	17.8%	82.2%	0.0%	17.8%	82.2%	0.0%	17.8%	82.2%	na	61.70	0.909
HR-SC-19[2]	0.0%	99.1%	0.9%	0.0%	99.1%	0.9%	5.4%	93.7%	0.9%	5.4%	93.7%	0.9%	na	0.83	0.726
HR-SC-20[2]	21.4%	65.9%	12.7%	17.3%	69.5%	13.2%	20.5%	63.6%	15.9%	20.5%	63.6%	15.9%	na	1.93	0.632
HR-SC-21[2]	0.0%	81.3%	18.7%	0.0%	81.3%	18.7%	0.0%	81.3%	18.7%	0.0%	80.8%	19.2%	na	9.70	0.737
HR-SC-22[2]	0.0%	100.0%	0.0%	0.0%	100.0%	0.0%	0.0%	100.0%	0.0%	0.0%	100.0%	0.0%	na	3.63	0.839
HR-SC-23[2]	0.9%	87.2%	11.9%	0.9%	86.3%	12.8%	2.3%	84.0%	13.7%	2.3%	83.6%	14.2%	na	2.55	0.702
HR-SC-24[2]	0.0%	69.9%	30.1%	0.0%	68.9%	31.1%	0.0%	67.6%	32.4%	0.0%	67.1%	32.9%	na	7.80	0.466
HR-SC-25[2]	0.0%	0.9%	99.1%	0.0%	0.9%	99.1%	0.0%	0.9%	99.1%	0.0%	0.9%	99.1%	na	108.84	0.955
CE-QPC-01	54.5%	24.4%	21.1%	51.6%	23.9%	24.4%	51.6%	23.9%	24.4%	51.6%	23.9%	24.4%	Modern	15.97	0.291
CE-QPC-07	18.3%	81.7%	0.0%	18.3%	81.7%	0.0%	18.3%	81.7%	0.0%	28.8%	71.2%	0.0%	Premodern	0.51	0.279
CE-QPC-09	11.9%	88.1%	0.0%	11.9%	88.1%	0.0%	11.9%	88.1%	0.0%	11.9%	88.1%	0.0%	Modern	0.53	0.381
MER-02[2]	76.6%	3.3%	20.1%	74.3%	2.8%	22.9%	69.2%	1.9%	29.0%	62.1%	1.4%	36.4%	Mixed	4.15	0.239
MER-03	3.2%	0.0%	96.8%	1.4%	0.0%	98.6%	0.0%	0.0%	100.0%	0.0%	0.0%	100.0%	Mixed	20.87	0.228
MER-09	29.6%	0.9%	69.4%	28.7%	0.9%	70.4%	25.5%	0.9%	73.6%	4.6%	0.0%	95.4%	Modern	55.83	0.237
MER-10	7.3%	0.0%	92.7%	5.5%	0.0%	94.5%	0.0%	0.0%	100.0%	0.0%	0.0%	100.0%	Modern	5.04	0.238
MER-11	41.8%	1.4%	56.8%	40.9%	0.0%	59.1%	40.5%	0.0%	59.5%	38.6%	0.5%	60.9%	Modern	9.79	0.249
MER-12	58.1%	41.5%	0.5%	62.2%	37.3%	0.5%	65.0%	34.6%	0.5%	65.0%	34.1%	0.9%	Modern	0.62	0.229
MER-20[2]	47.7%	52.3%	0.0%	55.9%	44.1%	0.0%	59.5%	40.5%	0.0%	59.5%	40.5%	0.0%	Mixed	1.12	0.236

Online resource (suppplementary table) 2. Selected attributes of GAMA-PBP (https://ca.water.usgs.gov/gama/) status wells sampled for orthophosphate concentration-page 28

GAMA-PBP ID	USGS Station ID[1]	GAMA-PBP study unit	GAMA-PBP study area[3]	Hydrogeologic zone
MER-21	371107120232201	Central Eastside San Joaquin Basin	Merced subbasin	Central Valley
MOD-01	373718121023501	Central Eastside San Joaquin Basin	Modesto subbasin	Central Valley
MOD-02	374109121000101	Central Eastside San Joaquin Basin	Modesto subbasin	Central Valley
MOD-09	374600120480001	Central Eastside San Joaquin Basin	Modesto subbasin	Central Valley
TRLK-01	372900120500001	Central Eastside San Joaquin Basin	Turlock subbasin	Central Valley
TRLK-02	372900120490001	Central Eastside San Joaquin Basin	Turlock subbasin	Central Valley
TRLK-03	373500120510001	Central Eastside San Joaquin Basin	Turlock subbasin	Central Valley
TRLK-05	373000120500001	Central Eastside San Joaquin Basin	Turlock subbasin	Central Valley
TRLK-10	373347121082201	Central Eastside San Joaquin Basin	Turlock subbasin	Central Valley
TRLK-11	373100120470001	Central Eastside San Joaquin Basin	Turlock subbasin	Central Valley
KERN-34	351800119180001	Kern County Subbasin	Kern County subbasin	Central Valley
KERN-35	351900119100001	Kern County Subbasin	Kern County subbasin	Central Valley
KERN-36	352100119060001	Kern County Subbasin	Kern County subbasin	Central Valley
KERN-37	352600118560001	Kern County Subbasin	Kern County subbasin	Central Valley
KERN-38[2]	350900118480001	Kern County Subbasin	Kern County subbasin	Central Valley
KERN-40	345900118560001	Kern County Subbasin	Kern County subbasin	Central Valley
KERN-41	354700119180001	Kern County Subbasin	Kern County subbasin	Central Valley
KERN-42	351800119040001	Kern County Subbasin	Kern County subbasin	Central Valley
KERN-43	350300118580001	Kern County Subbasin	Kern County subbasin	Central Valley
KERN-44	353400119190001	Kern County Subbasin	Kern County subbasin	Central Valley
KERN-45	352800119170001	Kern County Subbasin	Kern County subbasin	Central Valley
KERN-46	350700118560001	Kern County Subbasin	Kern County subbasin	Central Valley
KERN-47	352600119120001	Kern County Subbasin	Kern County subbasin	Central Valley
KING-04	363600120020001	Southeast San Joaquin Valley	Kings subbasin	Central Valley
KING-09	363500119380001	Southeast San Joaquin Valley	Kings subbasin	Central Valley
KING-10	363339119363501	Southeast San Joaquin Valley	Kings subbasin	Central Valley
KING-11	363200119220001	Southeast San Joaquin Valley	Kings subbasin	Central Valley
KING-12	363600119260001	Southeast San Joaquin Valley	Kings subbasin	Central Valley
KING-13	363500119280001	Southeast San Joaquin Valley	Kings subbasin	Central Valley
KING-15	364355119484601	Southeast San Joaquin Valley	Kings subbasin	Central Valley

Online resource (suppplementary table) 2. Selected attributes of GAMA-PBP (https://ca.water.usgs.gov/gama/) status wells sampled for orthophosphate concentration-page 29.

GAMA-PBP ID	USEPA Level III Ecoregions[4]	Level III Ecoregion Reference Concentration[4]	Status Sample Date	Status Sample Orthophosphate Concentration (mg/L as P)	Relative Concentration Category[5]	Redox state[6]	Elevation of LSD (meters above NAVD 88)[7]	Well depth (meters below LSD)[8]	Agricultural land use in 1974[9] (percent)	Natural land use in 1974[9] (percent)	Urban land use in 1974[9] (percent)
MER-21	Central California Valley	0.0770	Apr 5 2006	0.009	low	anoxic	57	105	15.5%	84.0%	0.5%
MOD-01	Central California Valley	0.0770	Mar 13 2006	0.006	low	oxic	21	25	88.0%	0.0%	12.0%
MOD-02	Central California Valley	0.0770	Mar 14 2006	0.021	low	oxic	27	120	0.0%	0.0%	100.0%
MOD-09	Central California Valley	0.0770	Mar 23 2006	0.017	low	oxic	62	103	44.5%	7.3%	48.2%
TRLK-01	Central California Valley	0.0770	Mar 15 2006	0.009	low	oxic	32	106	0.0%	0.0%	100.0%
TRLK-02	Central California Valley	0.0770	Mar 16 2006	0.011	low	oxic	33	83	77.6%	0.0%	22.4%
TRLK-03	Central California Valley	0.0770	Mar 21 2006	0.046	low	oxic	39	151	80.3%	6.9%	12.8%
TRLK-05	Central California Valley	0.0770	Mar 22 2006	0.009	low	oxic	33	144	0.0%	0.0%	100.0%
TRLK-10	Central California Valley	0.0770	Mar 27 2006	0.031	low	oxic	9	35	50.7%	42.0%	7.3%
TRLK-11	Central California Valley	0.0770	Mar 28 2006	0.011	low	oxic	6	125	47.0%	0.0%	53.0%
KERN-34	Central California Valley	0.0770	Jan 10 2006	0.006	low	oxic	94	233	0.9%	99.1%	0.0%
KERN-35	Central California Valley	0.0770	Jan 25 2006	0.006	low	oxic	105	207	18.7%	81.3%	0.0%
KERN-36	Central California Valley	0.0770	Jan 26 2006	0.003	low	oxic	115	219	47.0%	42.0%	11.0%
KERN-37	Central California Valley	0.0770	Feb 15 2006	0.008	low	oxic	161	152	0.0%	90.4%	9.6%
KERN-38[2]	Central California Valley	0.0770	Jan 11 2006	0.004	low	oxic	127	na	97.7%	2.3%	0.0%
KERN-40	Central California Valley	0.0770	Jan 30 2006	0.004	low	anoxic	336	456	43.8%	23.7%	32.4%
KERN-41	Central California Valley	0.0770	Jan 31 2006	0.004	low	oxic	81	424	80.2%	19.8%	0.0%
KERN-42	Central California Valley	0.0770	Feb 1 2006	0.012	low	oxic	109	219	24.5%	5.9%	69.5%
KERN-43	Central California Valley	0.0770	Feb 2 2006	0.004	low	anoxic	163	268	72.1%	2.3%	25.6%
KERN-44	Central California Valley	0.0770	Feb 7 2006	0.004	low	oxic	103	216	45.9%	1.4%	52.8%
KERN-45	Central California Valley	0.0770	Feb 8 2006	0.009	low	oxic	100	183	93.6%	3.2%	3.2%
KERN-46	Central California Valley	0.0770	Feb 28 2006	0.008	low	anoxic	113	313	94.1%	4.6%	1.4%
KERN-47	Central California Valley	0.0770	Mar 2 2006	0.006	low	oxic	105	247	84.0%	5.9%	10.0%
KING-04	Central California Valley	0.0770	Oct 17 2005	0.015	low	oxic	61	152	72.6%	25.1%	2.3%
KING-09	Central California Valley	0.0770	Oct 19 2005	0.006	low	oxic	96	177	46.3%	0.0%	53.7%
KING-10	Central California Valley	0.0770	Oct 19 2005	0.006	low	oxic	94	69	3.2%	1.4%	95.4%
KING-11	Central California Valley	0.0770	Oct 20 2005	0.014	low	oxic	104	165	7.3%	0.0%	92.7%
KING-12	Central California Valley	0.0770	Oct 20 2005	0.030	low	oxic	108	75	5.5%	0.0%	94.5%
KING-13	Central California Valley	0.0770	Oct 20 2005	0.020	low	oxic	96	128	34.9%	34.9%	30.2%
KING-15	Central California Valley	0.0770	Oct 25 2005	0.035	low	oxic	86	134	0.0%	0.0%	100.0%

Status and trends of orthophosphate concentrations in groundwater used for public supply in California *Environmental Monitoring and Assessment, Robert Kent, Tyler D. Johnson, and Michael R. Rosen, U.S. Geological Survey California Water Science Center-rhkent@usgs.gov*

Online resource (suppplementary table) 2. Selected attributes of GAMA-PBP (https://ca.water.usgs.gov/gama/) status wells sampled for orthophosphate concentration-page 30.

GAMA-PBP ID	Agricultural land use in 1982[9] (percent)	Natural land use in 1982[9] (percent)	Urban land use in 1982[9] (percent)	Agricultural land use in 1992[9] (percent)	Natural land use in 1992[9] (percent)	Urban land use in 1992[9] (percent)	Agricultural land use in 2002[9] (percent)	Natural land use in 2002[9] (percent)	Urban land use in 2002[9] (percent)	Agricultural land use in 2012[9] (percent)	Natural land use in 2012[9] (percent)	Urban land use in 2012[9] (percent)	Age Classification[10]	Septic Tanks[11]	Aridity[12]
MER-21	16.0%	83.6%	0.5%	17.8%	81.7%	0.5%	18.7%	80.8%	0.5%	18.7%	80.8%	0.5%	Premodern	1.10	0.233
MOD-01	88.5%	0.0%	11.5%	88.5%	0.0%	11.5%	88.5%	0.0%	11.5%	88.5%	0.0%	11.5%	Modern	58.98	0.255
MOD-02	0.0%	0.0%	100.0%	0.0%	0.0%	100.0%	0.0%	0.0%	100.0%	0.0%	0.0%	100.0%	Modern	7.04	0.257
MOD-09	46.3%	5.5%	48.2%	41.7%	8.7%	49.5%	42.2%	6.9%	50.9%	33.0%	5.5%	61.5%	Modern	15.97	0.286
TRLK-01	0.0%	0.0%	100.0%	0.0%	0.0%	100.0%	0.0%	0.0%	100.0%	0.0%	0.0%	100.0%	Premodern	59.32	0.245
TRLK-02	77.6%	0.0%	22.4%	77.6%	0.0%	22.4%	77.6%	0.0%	22.4%	77.6%	0.0%	22.4%	Mixed	39.46	0.245
TRLK-03	81.2%	6.0%	12.8%	81.2%	6.0%	12.8%	81.2%	6.0%	12.8%	58.7%	2.8%	38.5%	Premodern	15.92	0.261
TRLK-05	0.0%	0.0%	100.0%	0.0%	0.0%	100.0%	0.0%	0.0%	100.0%	0.0%	0.0%	100.0%	Mixed	20.87	0.246
TRLK-10	53.4%	39.3%	7.3%	53.4%	39.3%	7.3%	53.9%	38.8%	7.3%	53.9%	38.8%	7.3%	Modern	2.95	0.239
TRLK-11	47.0%	0.0%	53.0%	47.0%	0.0%	53.0%	47.0%	0.0%	53.0%	13.5%	0.0%	86.5%	Mixed	13.20	0.252
KERN-34	0.9%	99.1%	0.0%	0.9%	99.1%	0.0%	2.8%	97.2%	0.0%	24.1%	75.9%	0.0%	Modern	0.74	0.114
KERN-35	18.7%	81.3%	0.0%	18.7%	81.3%	0.0%	18.7%	81.3%	0.0%	30.6%	69.4%	0.0%	Modern	1.13	0.113
KERN-36	47.5%	21.0%	31.5%	16.4%	17.8%	65.8%	18.7%	16.0%	65.3%	12.8%	12.3%	74.9%	Mixed	36.86	0.113
KERN-37	0.0%	87.7%	12.3%	0.0%	85.4%	14.6%	0.0%	73.5%	26.5%	0.0%	66.7%	33.3%	Modern	1.74	0.128
KERN-38[2]	97.2%	2.3%	0.5%	94.0%	2.3%	3.7%	94.0%	2.3%	3.7%	94.0%	2.3%	3.7%	Mixed	0.31	0.121
KERN-40	44.7%	21.5%	33.8%	41.6%	20.5%	37.9%	41.6%	13.2%	45.2%	41.6%	4.6%	53.9%	PremodernOrMixed	0.42	0.150
KERN-41	83.4%	16.6%	0.0%	82.9%	16.6%	0.5%	85.7%	13.4%	0.9%	85.7%	11.5%	2.8%	Premodern	1.68	0.132
KERN-42	25.9%	3.6%	70.5%	0.9%	0.0%	99.1%	0.0%	0.0%	100.0%	0.0%	0.0%	100.0%	Premodern	0.00	0.111
KERN-43	69.4%	2.3%	28.3%	68.9%	2.3%	28.8%	68.5%	1.8%	29.7%	68.5%	0.0%	31.5%	PremodernOrMixed	0.39	0.121
KERN-44	46.3%	0.9%	52.8%	45.0%	0.9%	54.1%	45.9%	0.0%	54.1%	45.4%	0.5%	54.1%	Modern	10.69	0.126
KERN-45	96.4%	0.9%	2.7%	96.4%	0.9%	2.7%	95.9%	0.9%	3.2%	95.9%	0.9%	3.2%	Mixed	11.46	0.120
KERN-46	95.9%	2.7%	1.4%	95.9%	2.7%	1.4%	97.7%	0.0%	2.3%	97.7%	0.0%	2.3%	PremodernOrMixed	0.59	0.110
KERN-47	84.5%	5.5%	10.0%	83.6%	5.5%	11.0%	84.5%	4.6%	11.0%	84.5%	4.1%	11.4%	ModernOrMixed	2.31	0.118
KING-04	84.5%	12.8%	2.7%	78.5%	15.5%	5.9%	90.9%	1.4%	7.8%	90.9%	1.4%	7.8%	Mixed	1.25	0.159
KING-09	52.8%	0.0%	47.2%	45.3%	0.0%	54.7%	44.9%	0.0%	55.1%	38.3%	0.0%	61.7%	ModernOrMixed	18.26	0.206
KING-10	2.3%	1.4%	96.3%	2.3%	1.4%	96.3%	2.3%	1.4%	96.3%	2.3%	0.9%	96.8%	ModernOrMixed	9.62	0.198
KING-11	6.8%	0.0%	93.2%	6.8%	0.0%	93.2%	6.4%	0.0%	93.6%	0.0%	0.0%	100.0%	PremodernOrMixed	4.33	0.211
KING-12	2.3%	0.0%	97.7%	0.0%	0.0%	100.0%	0.0%	0.0%	100.0%	0.0%	0.0%	100.0%	ModernOrMixed	32.75	0.211
KING-13	34.9%	34.9%	30.2%	33.5%	34.9%	31.6%	53.8%	18.4%	27.8%	53.8%	8.5%	37.7%	PremodernOrMixed	3.00	0.210
KING-15	0.0%	0.0%	100.0%	0.0%	0.0%	100.0%	0.0%	0.0%	100.0%	0.0%	0.0%	100.0%	ModernOrMixed	15.81	0.208

Online resource (suppplementary table) 2. Selected attributes of GAMA-PBP (https://ca.water.usgs.gov/gama/) status wells sampled for orthophosphate concentration-page 31

GAMA-PBP ID	USGS Station ID[1]	GAMA-PBP study unit	GAMA-PBP study area[3]	Hydrogeologic zone
KING-16[2]	364700119500001	Southeast San Joaquin Valley	Kings subbasin	Central Valley
KING-17	364156119475201	Southeast San Joaquin Valley	Kings subbasin	Central Valley
KING-20	364359119293601	Southeast San Joaquin Valley	Kings subbasin	Central Valley
KING-25	362700119290001	Southeast San Joaquin Valley	Kings subbasin	Central Valley
KING-30	363900119260001	Southeast San Joaquin Valley	Kings subbasin	Central Valley
KING-38	364015119420001	Southeast San Joaquin Valley	Kings subbasin	Central Valley
KWH-03	361615119184001	Southeast San Joaquin Valley	Kaweah subbasin	Central Valley
KWH-06	361224119060001	Southeast San Joaquin Valley	Kaweah subbasin	Central Valley
KWH-11	360945119200001	Southeast San Joaquin Valley	Kaweah subbasin	Central Valley
KWH-12	361228119202101	Southeast San Joaquin Valley	Kaweah subbasin	Central Valley
KWH-14	361929119230001	Southeast San Joaquin Valley	Kaweah subbasin	Central Valley
TLR-02	361906119420001	Southeast San Joaquin Valley	Tulare Lake subbasin	Central Valley
TLR-04	362128119390001	Southeast San Joaquin Valley	Tulare Lake subbasin	Central Valley
TLR-05	362305119460001	Southeast San Joaquin Valley	Tulare Lake subbasin	Central Valley
TLR-06[2]	361134119490001	Southeast San Joaquin Valley	Tulare Lake subbasin	Central Valley
TLR-08[2]	361934119360001	Southeast San Joaquin Valley	Tulare Lake subbasin	Central Valley
TULE-01	355259119160701	Southeast San Joaquin Valley	Tule subbasin	Central Valley
TULE-03	360631119190001	Southeast San Joaquin Valley	Tule subbasin	Central Valley
TULE-07	360331119180001	Southeast San Joaquin Valley	Tule subbasin	Central Valley
TULE-08	355810119170001	Southeast San Joaquin Valley	Tule subbasin	Central Valley
TULE-17	355053119195201	Southeast San Joaquin Valley	Tule subbasin	Central Valley
MADCHOW-01	365500120060001	Madera—Chowchilla	Madera and Chowchilla subbasins	Central Valley
MADCHOW-02	365936120062901	Madera—Chowchilla	Madera and Chowchilla subbasins	Central Valley
MADCHOW-03	365747120034901	Madera—Chowchilla	Madera and Chowchilla subbasins	Central Valley
MADCHOW-04	365042119493001	Madera—Chowchilla	Madera and Chowchilla subbasins	Central Valley
MADCHOW-05[2]	370300119590001	Madera—Chowchilla	Madera and Chowchilla subbasins	Central Valley
MADCHOW-06	365800119430001	Madera—Chowchilla	Madera and Chowchilla subbasins	Central Valley
MADCHOW-07[2]	370700120030001	Madera—Chowchilla	Madera and Chowchilla subbasins	Central Valley
MADCHOW-08	370600120090001	Madera—Chowchilla	Madera and Chowchilla subbasins	Central Valley
MADCHOW-09	370500120170001	Madera—Chowchilla	Madera and Chowchilla subbasins	Central Valley

Online resource (suppplementary table) 2. Selected attributes of GAMA-PBP (https://ca.water.usgs.gov/gama/) status wells sampled for orthophosphate concentration-page 32.

GAMA-PBP ID	USEPA Level III Ecoregions[4]	Level III Ecoregion Reference Concentration[4]	Status Sample Date	Status Sample Orthophosphate Concentration (mg/L as P)	Relative Concentration Category[5]	Redox state[6]	Elevation of LSD (meters above NAVD 88)[7]	Well depth (meters below LSD)[8]	Agricultural land use in 1974[9] (percent)	Natural land use in 1974[9] (percent)	Urban land use in 1974[9] (percent)
KING-16[2]	Central California Valley	0.0770	Oct 25 2005	0.048	low	oxic	93	na	0.0%	0.9%	99.1%
KING-17	Central California Valley	0.0770	Oct 26 2005	0.014	low	oxic	85	198	21.0%	0.0%	79.0%
KING-20	Central California Valley	0.0770	Oct 27 2005	0.078	moderate	oxic	120	38	72.4%	14.3%	13.4%
KING-25	Central California Valley	0.0770	Nov 2 2005	0.006	low	oxic	88	117	56.0%	7.9%	36.1%
KING-30	Central California Valley	0.0770	Nov 3 2005	0.024	low	oxic	117	149	81.4%	15.0%	3.6%
KING-38	Central California Valley	0.0770	Dec 15 2005	0.011	low	oxic	93	213	99.5%	0.0%	0.5%
KWH-03	Central California Valley	0.0770	Oct 31 2005	0.006	low	oxic	97	94	69.9%	1.8%	28.3%
KWH-06	Central California Valley	0.0770	Nov 15 2005	0.007	low	oxic	113	177	18.7%	2.8%	78.5%
KWH-11	Central California Valley	0.0770	Nov 17 2005	0.042	low	anoxic	83	213	50.5%	4.6%	45.0%
KWH-12	Central California Valley	0.0770	Nov 28 2005	0.006	low	oxic	89	123	0.0%	0.0%	100.0%
KWH-14	Central California Valley	0.0770	Nov 30 2005	0.006	low	oxic	90	122	4.1%	0.0%	95.9%
TLR-02	Central California Valley	0.0770	Nov 29 2005	0.054	low	oxic	73	405	60.7%	1.8%	37.4%
TLR-04	Central California Valley	0.0770	Dec 1 2005	0.021	low	anoxic	77	402	69.5%	1.4%	29.1%
TLR-05	Central California Valley	0.0770	Dec 5 2005	0.105	high	anoxic	73	171	91.7%	8.3%	0.0%
TLR-06[2]	Central California Valley	0.0770	Dec 6 2005	0.154	high	oxic	62	na	58.9%	1.4%	39.7%
TLR-08[2]	Central California Valley	0.0770	Dec 12 2005	0.032	low	oxic	76	na	50.7%	1.4%	47.9%
TULE-01	Central California Valley	0.0770	Nov 29 2005	0.006	low	oxic	87	244	11.4%	1.4%	87.3%
TULE-03	Central California Valley	0.0770	Nov 30 2005	0.020	low	oxic	81	85	60.9%	29.1%	10.0%
TULE-07	Central California Valley	0.0770	Dec 6 2005	0.005	low	oxic	84	183	23.6%	0.0%	76.4%
TULE-08	Central California Valley	0.0770	Dec 7 2005	0.028	low	oxic	83	247	35.6%	3.7%	60.7%
TULE-17	Central California Valley	0.0770	Feb 28 2006	0.006	low	oxic	74	75	6.4%	93.6%	0.0%
MADCHOW-01	Central California Valley	0.0770	Apr 14 2008	0.048	low	oxic	76	180	32.3%	7.4%	60.4%
MADCHOW-02	Central California Valley	0.0770	Apr 15 2008	0.057	low	oxic	77	183	10.2%	32.4%	57.4%
MADCHOW-03	Central California Valley	0.0770	Apr 15 2008	0.058	low	oxic	84	165	0.0%	0.0%	100.0%
MADCHOW-04	Central California Valley	0.0770	Apr 16 2008	0.094	moderate	oxic	103	146	17.3%	5.0%	77.7%
MADCHOW-05[2]	Central California Valley	0.0770	Apr 16 2008	0.033	low	na	109	107	57.3%	32.7%	10.0%
MADCHOW-06	Central California Foothills and Coastal Mountains	0.0300	Apr 17 2008	0.018	low	oxic	99	94	12.2%	71.8%	16.0%
MADCHOW-07[2]	Central California Valley	0.0770	Apr 21 2008	0.024	low	oxic	105	na	72.2%	27.8%	0.0%
MADCHOW-08	Central California Valley	0.0770	Apr 22 2008	0.044	low	oxic	87	250	92.2%	3.7%	4.1%
MADCHOW-09	Central California Valley	0.0770	Apr 22 2008	0.028	low	oxic	67	71	74.4%	7.3%	18.3%

Status and trends of orthophosphate concentrations in groundwater used for public supply in California *Environmental Monitoring and Assessment, Robert Kent, Tyler D. Johnson, and Michael R. Rosen, U.S. Geological Survey California Water Science Center-rhkent@usgs.gov*

Online resource (suppplementary table) 2. Selected attributes of GAMA-PBP (https://ca.water.usgs.gov/gama/) status wells sampled for orthophosphate concentration-page 33.

GAMA-PBP ID	Agricultural land use in 1982[9] (percent)	Natural land use in 1982[9] (percent)	Urban land use in 1982[9] (percent)	Agricultural land use in 1992[9] (percent)	Natural land use in 1992[9] (percent)	Urban land use in 1992[9] (percent)	Agricultural land use in 2002[9] (percent)	Natural land use in 2002[9] (percent)	Urban land use in 2002[9] (percent)	Agricultural land use in 2012[9] (percent)	Natural land use in 2012[9] (percent)	Urban land use in 2012[9] (percent)	Age Classification[10]	Septic Tanks[11]	Aridity[12]
KING-16[2]	0.0%	0.9%	99.1%	0.0%	0.9%	99.1%	0.0%	0.9%	99.1%	0.0%	0.9%	99.1%	ModernOrMixed	0.00	0.212
KING-17	17.8%	0.0%	82.2%	17.4%	0.0%	82.6%	17.4%	0.0%	82.6%	16.4%	0.0%	83.6%	Premodern	15.63	0.206
KING-20	72.8%	13.4%	13.8%	72.4%	13.4%	14.3%	72.8%	12.9%	14.3%	72.8%	12.9%	14.3%	Modern	7.31	0.219
KING-25	56.0%	7.9%	36.1%	56.0%	7.9%	36.1%	57.4%	6.5%	36.1%	57.4%	6.5%	36.1%	ModernOrMixed	2.74	0.183
KING-30	87.7%	8.2%	4.1%	85.0%	10.9%	4.1%	86.4%	8.6%	5.0%	86.4%	8.6%	5.0%	PremodernOrMixed	4.75	0.215
KING-38	99.5%	0.0%	0.5%	99.5%	0.0%	0.5%	99.1%	0.0%	0.9%	89.7%	0.0%	10.3%	PremodernOrMixed	7.13	0.209
KWH-03	68.0%	1.8%	30.1%	68.0%	1.8%	30.1%	63.5%	1.4%	35.2%	63.5%	0.5%	36.1%	ModernOrMixed	9.69	0.188
KWH-06	16.4%	0.9%	82.7%	15.0%	0.9%	84.1%	13.6%	0.5%	86.0%	13.6%	0.0%	86.4%	ModernOrMixed	8.72	0.199
KWH-11	51.4%	3.2%	45.4%	50.5%	3.2%	46.3%	49.5%	3.2%	47.2%	46.8%	0.0%	53.2%	ModernOrMixed	9.53	0.164
KWH-12	0.0%	0.0%	100.0%	0.0%	0.0%	100.0%	0.0%	0.0%	100.0%	0.0%	0.0%	100.0%	Mixed	1.30	0.170
KWH-14	12.0%	0.0%	88.0%	9.2%	0.0%	90.8%	9.7%	0.0%	90.3%	6.0%	0.0%	94.0%	Modern	4.28	0.180
TLR-02	61.6%	0.9%	37.4%	61.6%	0.9%	37.4%	61.6%	0.9%	37.4%	61.6%	0.9%	37.4%	PremodernOrMixed	4.89	0.150
TLR-04	64.1%	1.4%	34.5%	23.2%	0.0%	76.8%	9.5%	0.0%	90.5%	6.8%	0.0%	93.2%	Premodern	4.21	0.153
TLR-05	92.2%	7.8%	0.0%	92.2%	7.8%	0.0%	92.2%	7.8%	0.0%	92.2%	7.8%	0.0%	Mixed	3.05	0.146
TLR-06[2]	60.7%	0.0%	39.3%	59.4%	0.0%	40.6%	58.9%	0.0%	41.1%	58.9%	0.0%	41.1%	PremodernOrMixed	5.78	0.140
TLR-08[2]	51.1%	0.9%	47.9%	49.8%	1.4%	48.9%	49.3%	1.4%	49.3%	46.1%	0.0%	53.9%	PremodernOrMixed	6.80	0.153
TULE-01	11.8%	0.9%	87.3%	11.4%	0.9%	87.7%	11.4%	0.9%	87.7%	5.9%	0.0%	94.1%	Mixed	3.06	0.137
TULE-03	72.7%	16.4%	10.9%	60.9%	26.4%	12.7%	63.6%	23.2%	13.2%	63.6%	23.2%	13.2%	ModernOrMixed	1.97	0.155
TULE-07	24.1%	0.0%	75.9%	24.1%	0.0%	75.9%	24.1%	0.0%	75.9%	24.1%	0.0%	75.9%	ModernOrMixed	0.15	0.152
TULE-08	35.6%	3.7%	60.7%	32.0%	3.7%	64.4%	32.0%	3.7%	64.4%	32.0%	0.9%	67.1%	Premodern	6.18	0.146
TULE-17	21.0%	79.0%	0.0%	9.6%	90.4%	0.0%	15.1%	84.9%	0.0%	15.1%	84.9%	0.0%	ModernOrMixed	0.94	0.135
MADCHOW-01	32.7%	6.9%	60.4%	30.0%	6.9%	63.1%	30.4%	6.5%	63.1%	30.4%	3.7%	65.9%	Modern	6.94	0.214
MADCHOW-02	14.4%	25.0%	60.6%	11.6%	21.3%	67.1%	11.6%	18.5%	69.9%	10.6%	0.0%	89.4%	Premodern	6.94	0.218
MADCHOW-03	0.0%	0.0%	100.0%	0.0%	0.0%	100.0%	0.0%	0.0%	100.0%	0.0%	0.0%	100.0%	Mixed	4.05	0.218
MADCHOW-04	17.3%	4.5%	78.2%	1.4%	0.9%	97.7%	1.4%	0.9%	97.7%	1.4%	0.9%	97.7%	Modern	13.78	0.214
MADCHOW-05[2]	60.5%	29.5%	10.0%	60.0%	30.0%	10.0%	60.0%	30.0%	10.0%	57.7%	31.8%	10.5%	Mixed	1.73	0.227
MADCHOW-06	20.7%	65.3%	14.1%	11.7%	72.3%	16.0%	17.4%	65.3%	17.4%	16.9%	65.7%	17.4%	Modern	4.54	0.246
MADCHOW-07[2]	88.4%	11.6%	0.0%	77.8%	22.2%	0.0%	79.2%	20.8%	0.0%	79.2%	20.8%	0.0%	Mixed	0.65	0.232
MADCHOW-08	89.9%	3.7%	6.5%	81.1%	3.7%	15.2%	55.8%	3.7%	40.6%	55.3%	0.9%	43.8%	Mixed	1.92	0.229
MADCHOW-09	74.9%	6.8%	18.3%	74.9%	6.8%	18.3%	74.9%	6.8%	18.3%	74.9%	6.8%	18.3%	Modern	3.16	0.224

Online resource (suppplementary table) 2. Selected attributes of GAMA-PBP (https://ca.water.usgs.gov/gama/) status wells sampled for orthophosphate concentration-page 34.

GAMA-PBP ID	USGS Station ID[1]	GAMA-PBP study unit	GAMA-PBP study area[3]	Hydrogeologic zone
MADCHOW-10	370700120150001	Madera—Chowchilla	Madera and Chowchilla subbasins	Central Valley
MADCHOW-11	370500120080001	Madera—Chowchilla	Madera and Chowchilla subbasins	Central Valley
MADCHOW-12	370700120290001	Madera—Chowchilla	Madera and Chowchilla subbasins	Central Valley
MADCHOW-13	365200120070001	Madera—Chowchilla	Madera and Chowchilla subbasins	Central Valley
MADCHOW-14	365100120040001	Madera—Chowchilla	Madera and Chowchilla subbasins	Central Valley
MADCHOW-15	365300119480001	Madera—Chowchilla	Madera and Chowchilla subbasins	Central Valley
MADCHOW-16	370100120030001	Madera—Chowchilla	Madera and Chowchilla subbasins	Central Valley
MADCHOW-17	370200119480001	Madera—Chowchilla	Madera and Chowchilla subbasins	Central Valley
MADCHOW-18	365500120020001	Madera—Chowchilla	Madera and Chowchilla subbasins	Central Valley
MADCHOW-19	365500119450001	Madera—Chowchilla	Madera and Chowchilla subbasins	Central Valley
MADCHOW-20	365300120170001	Madera—Chowchilla	Madera and Chowchilla subbasins	Central Valley
MADCHOW-21	365700119470001	Madera—Chowchilla	Madera and Chowchilla subbasins	Central Valley
MADCHOW-22	370400120280001	Madera—Chowchilla	Madera and Chowchilla subbasins	Central Valley
MADCHOW-23	370400120310001	Madera—Chowchilla	Madera and Chowchilla subbasins	Central Valley
MADCHOW-24	370056120200601	Madera—Chowchilla	Madera and Chowchilla subbasins	Central Valley
MADCHOW-25	365600120160001	Madera—Chowchilla	Madera and Chowchilla subbasins	Central Valley
MADCHOW-26	365500120220001	Madera—Chowchilla	Madera and Chowchilla subbasins	Central Valley
MADCHOW-27	364900120100001	Madera—Chowchilla	Madera and Chowchilla subbasins	Central Valley
MADCHOW-28	370100120210001	Madera—Chowchilla	Madera and Chowchilla subbasins	Central Valley
MADCHOW-29	365045120151801	Madera—Chowchilla	Madera and Chowchilla subbasins	Central Valley
MADCHOW-30	365900120230001	Madera—Chowchilla	Madera and Chowchilla subbasins	Central Valley
ESAC-03	393000121350001	Middle Sacramento Valley	Eastern Sacramento Valley subbasins	Central Valley
ESAC-05	391600121390001	Middle Sacramento Valley	Eastern Sacramento Valley subbasins	Central Valley
ESAC-06	393800121430001	Middle Sacramento Valley	Eastern Sacramento Valley subbasins	Central Valley
ESAC-10	390358121331001	Middle Sacramento Valley	Eastern Sacramento Valley subbasins	Central Valley
ESAC-11	391900121480001	Middle Sacramento Valley	Eastern Sacramento Valley subbasins	Central Valley
ESAC-12[2]	393500121580001	Middle Sacramento Valley	Eastern Sacramento Valley subbasins	Central Valley
ESAC-15	394531121510301	Middle Sacramento Valley	Eastern Sacramento Valley subbasins	Central Valley
ESAC-16	394300121500001	Middle Sacramento Valley	Eastern Sacramento Valley subbasins	Central Valley
ESAC-17	390600121380001	Middle Sacramento Valley	Eastern Sacramento Valley subbasins	Central Valley

Online resource (suppplementary table) 2. Selected attributes of GAMA-PBP (https://ca.water.usgs.gov/gama/) status wells sampled for orthophosphate concentration-page 35.

GAMA-PBP ID	USEPA Level III Ecoregions[4]	Level III Ecoregion Reference Concentration[4]	Status Sample Date	Status Sample Orthophosphate Concentration (mg/L as P)	Relative Concentration Category[5]	Redox state[6]	Elevation of LSD (meters above NAVD 88)[7]	Well depth (meters below LSD)[8]	Agricultural land use in 1974[9] (percent)	Natural land use in 1974[9] (percent)	Urban land use in 1974[9] (percent)
MADCHOW-10	Central California Valley	0.0770	Apr 24 2008	0.038	low	oxic	72	253	0.0%	0.0%	100.0%
MADCHOW-11	Central California Valley	0.0770	Apr 24 2008	0.041	low	oxic	87	238	92.5%	3.3%	4.2%
MADCHOW-12	Central California Valley	0.0770	Apr 28 2008	0.017	low	anoxic	44	91	84.4%	3.2%	12.4%
MADCHOW-13	Central California Valley	0.0770	Apr 29 2008	0.048	low	oxic	68	204	96.8%	1.4%	1.8%
MADCHOW-14	Central California Valley	0.0770	Apr 30 2008	0.032	low	oxic	73	118	94.9%	1.9%	3.2%
MADCHOW-15	Central California Valley	0.0770	Apr 30 2008	0.104	high	oxic	111	137	70.5%	29.1%	0.5%
MADCHOW-16	Central California Valley	0.0770	May 1 2008	0.040	low	oxic	89	226	6.4%	0.0%	93.6%
MADCHOW-17	Central California Foothills and Coastal Mountains	0.0300	May 1 2008	0.148	high	oxic	148	43	0.0%	100.0%	0.0%
MADCHOW-18	Central California Valley	0.0770	May 6 2008	0.082	moderate	oxic	82	101	88.7%	2.3%	8.9%
MADCHOW-19	Central California Valley	0.0770	May 6 2008	0.047	low	oxic	90	61	10.0%	89.6%	0.5%
MADCHOW-20	Central California Valley	0.0770	May 7 2008	0.044	low	oxic	53	107	99.5%	0.0%	0.5%
MADCHOW-21	Central California Valley	0.0770	May 7 2008	0.153	high	oxic	130	98	19.9%	71.0%	9.0%
MADCHOW-22	Central California Valley	0.0770	May 8 2008	0.029	low	oxic	44	99	74.0%	19.6%	6.4%
MADCHOW-23	Central California Valley	0.0770	May 12 2008	0.035	low	oxic	37	200	71.6%	22.9%	5.5%
MADCHOW-24	Central California Valley	0.0770	May 13 2008	0.024	low	oxic	53	90	97.7%	1.8%	0.5%
MADCHOW-25	Central California Valley	0.0770	May 13 2008	0.056	low	oxic	55	61	100.0%	0.0%	0.0%
MADCHOW-26	Central California Valley	0.0770	May 14 2008	0.051	low	oxic	47	155	80.6%	19.0%	0.5%
MADCHOW-27	Central California Valley	0.0770	May 14 2008	0.064	low	oxic	62	146	99.5%	0.0%	0.5%
MADCHOW-28	Central California Valley	0.0770	May 19 2008	0.053	low	oxic	52	66	98.6%	0.0%	1.4%
MADCHOW-29	Central California Valley	0.0770	May 20 2008	0.041	low	oxic	54	104	72.4%	25.8%	1.8%
MADCHOW-30	Central California Valley	0.0770	May 21 2008	0.017	low	oxic	47	118	99.1%	0.9%	0.0%
ESAC-03	Central California Valley	0.0770	Jul 10 2006	0.075	low	oxic	54	83	0.0%	0.0%	100.0%
ESAC-05	Central California Valley	0.0770	Jul 10 2006	0.168	high	oxic	23	125	10.9%	0.0%	89.1%
ESAC-06	Central California Valley	0.0770	Jul 12 2006	0.118	high	oxic	55	79	0.0%	76.0%	24.0%
ESAC-10	Central California Valley	0.0770	Jul 13 2006	0.095	moderate	anoxic	16	96	15.5%	23.2%	61.4%
ESAC-11	Central California Valley	0.0770	Jul 13 2006	0.100	moderate	anoxic	21	158	0.0%	100.0%	0.0%
ESAC-12[2]	Central California Valley	0.0770	Jul 17 2006	0.058	low	na	33	114	99.5%	0.5%	0.0%
ESAC-15	Central California Foothills and Coastal Mountains	0.0300	Jul 20 2006	0.003	low	oxic	60	152	0.0%	0.0%	100.0%
ESAC-16	Central California Foothills and Coastal Mountains	0.0300	Jul 20 2006	0.092	moderate	oxic	84	171	0.0%	28.8%	71.2%
ESAC-17	Central California Valley	0.0770	Jul 20 2006	0.167	high	anoxic	15	37	18.6%	3.7%	77.7%

Status and trends of orthophosphate concentrations in groundwater used for public supply in California *Environmental Monitoring and Assessment, Robert Kent, Tyler D. Johnson, and Michael R. Rosen, U.S. Geological Survey California Water Science Center-rhkent@usgs.gov*

Online resource (suppplementary table) 2. Selected attributes of GAMA-PBP (https://ca.water.usgs.gov/gama/) status wells sampled for orthophosphate concentration-page 36.

GAMA-PBP ID	Agricultural land use in 1982[9] (percent)	Natural land use in 1982[9] (percent)	Urban land use in 1982[9] (percent)	Agricultural land use in 1992[9] (percent)	Natural land use in 1992[9] (percent)	Urban land use in 1992[9] (percent)	Agricultural land use in 2002[9] (percent)	Natural land use in 2002[9] (percent)	Urban land use in 2002[9] (percent)	Agricultural land use in 2012[9] (percent)	Natural land use in 2012[9] (percent)	Urban land use in 2012[9] (percent)	Age Classification[10]	Septic Tanks[11]	Aridity[12]
MADCHOW-10	0.0%	0.0%	100.0%	0.0%	0.0%	100.0%	0.0%	0.0%	100.0%	0.0%	0.0%	100.0%	Premodern	1.87	0.230
MADCHOW-11	89.7%	1.9%	8.5%	74.6%	2.3%	23.0%	74.6%	2.3%	23.0%	74.6%	0.0%	25.4%	Premodern	1.92	0.229
MADCHOW-12	83.9%	3.2%	12.8%	83.9%	3.2%	12.8%	83.9%	3.2%	12.8%	83.9%	3.2%	12.8%	Mixed	1.06	0.224
MADCHOW-13	96.8%	1.4%	1.8%	96.8%	1.4%	1.8%	98.2%	0.0%	1.8%	98.2%	0.0%	1.8%	Mixed	2.62	0.200
MADCHOW-14	95.8%	0.9%	3.2%	95.4%	0.9%	3.7%	96.3%	0.0%	3.7%	96.3%	0.0%	3.7%	Mixed	2.75	0.205
MADCHOW-15	71.4%	25.9%	2.7%	70.9%	24.1%	5.0%	71.4%	22.3%	6.4%	68.6%	21.4%	10.0%	Premodern	6.73	0.224
MADCHOW-16	15.9%	0.0%	84.1%	3.6%	0.0%	96.4%	21.4%	0.0%	78.6%	21.4%	0.0%	78.6%	Premodern	22.62	0.222
MADCHOW-17	0.0%	100.0%	0.0%	0.0%	100.0%	0.0%	0.0%	100.0%	0.0%	0.0%	100.0%	0.0%	Modern	0.35	0.255
MADCHOW-18	86.9%	1.9%	11.3%	83.6%	2.3%	14.1%	83.6%	1.9%	14.6%	83.6%	1.9%	14.6%	Modern	5.72	0.216
MADCHOW-19	25.8%	70.6%	3.6%	7.2%	85.5%	7.2%	56.1%	36.7%	7.2%	50.7%	42.1%	7.2%	Mixed	6.99	0.226
MADCHOW-20	99.5%	0.0%	0.5%	99.5%	0.0%	0.5%	99.5%	0.0%	0.5%	99.5%	0.0%	0.5%	Modern	0.38	0.171
MADCHOW-21	26.2%	63.3%	10.4%	25.8%	63.3%	10.9%	25.8%	61.1%	13.1%	24.4%	60.6%	14.9%	Premodern	5.60	0.242
MADCHOW-22	74.4%	19.2%	6.4%	69.9%	22.8%	7.3%	62.1%	30.6%	7.3%	62.1%	30.1%	7.8%	Premodern	0.63	0.214
MADCHOW-23	71.6%	22.9%	5.5%	70.2%	22.9%	6.9%	69.7%	22.9%	7.3%	69.7%	22.9%	7.3%	Premodern	0.62	0.212
MADCHOW-24	97.7%	1.8%	0.5%	97.7%	1.8%	0.5%	97.3%	1.8%	0.9%	97.3%	1.8%	0.9%	Mixed	2.79	0.198
MADCHOW-25	99.5%	0.0%	0.5%	99.5%	0.0%	0.5%	99.5%	0.0%	0.5%	99.5%	0.0%	0.5%	Modern	0.38	0.184
MADCHOW-26	97.7%	1.9%	0.5%	97.7%	1.9%	0.5%	99.5%	0.0%	0.5%	99.5%	0.0%	0.5%	Modern	0.38	0.173
MADCHOW-27	99.5%	0.0%	0.5%	99.1%	0.0%	0.9%	98.6%	0.0%	1.4%	98.6%	0.0%	1.4%	Modern	2.50	0.177
MADCHOW-28	98.6%	0.0%	1.4%	98.6%	0.0%	1.4%	98.6%	0.0%	1.4%	98.6%	0.0%	1.4%	Modern	2.79	0.204
MADCHOW-29	76.0%	19.8%	4.1%	71.0%	23.0%	6.0%	69.1%	22.1%	8.8%	65.4%	22.1%	12.4%	Modern	0.38	0.168
MADCHOW-30	99.5%	0.5%	0.0%	99.5%	0.5%	0.0%	99.5%	0.5%	0.0%	99.5%	0.5%	0.0%	Mixed	0.54	0.190
ESAC-03	0.0%	0.0%	100.0%	0.0%	0.0%	100.0%	0.0%	0.0%	100.0%	0.0%	0.0%	100.0%	Modern	1.69	0.543
ESAC-05	9.5%	0.0%	90.5%	8.6%	0.0%	91.4%	7.3%	0.0%	92.7%	7.3%	0.0%	92.7%	Modern	7.89	0.456
ESAC-06	0.0%	72.4%	27.6%	0.0%	68.2%	31.8%	0.0%	68.2%	31.8%	0.0%	66.8%	33.2%	Modern	8.06	0.557
ESAC-10	41.8%	14.1%	44.1%	27.7%	18.2%	54.1%	25.9%	1.4%	72.7%	25.9%	1.4%	72.7%	Modern	25.67	0.438
ESAC-11	0.0%	100.0%	0.0%	0.0%	100.0%	0.0%	9.5%	90.5%	0.0%	9.5%	90.5%	0.0%	Premodern	1.29	0.438
ESAC-12[2]	100.0%	0.0%	0.0%	100.0%	0.0%	0.0%	100.0%	0.0%	0.0%	100.0%	0.0%	0.0%	Mixed	1.08	0.446
ESAC-15	0.0%	0.0%	100.0%	0.0%	0.0%	100.0%	0.0%	0.0%	100.0%	0.0%	0.0%	100.0%	Modern	385.26	0.534
ESAC-16	0.5%	28.3%	71.2%	0.5%	28.3%	71.2%	4.6%	24.2%	71.2%	4.6%	23.7%	71.7%	Mixed	8.08	0.577
ESAC-17	17.2%	3.3%	79.5%	14.4%	3.3%	82.3%	10.2%	3.3%	86.5%	10.2%	0.0%	89.8%	Mixed	237.45	0.431

Online resource (suppplementary table) 2. Selected attributes of GAMA-PBP (https://ca.water.usgs.gov/gama/) status wells sampled for orthophosphate concentration-page 37.

GAMA-PBP ID	USGS Station ID[1]	GAMA-PBP study unit	GAMA-PBP study area[3]	Hydrogeologic zone
ESAC-18	394300121480001	Middle Sacramento Valley	Eastern Sacramento Valley subbasins	Central Valley
ESAC-19	390800121450001	Middle Sacramento Valley	Eastern Sacramento Valley subbasins	Central Valley
ESAC-21[2]	391444121571401	Middle Sacramento Valley	Eastern Sacramento Valley subbasins	Central Valley
ESAC-22	392400121420001	Middle Sacramento Valley	Eastern Sacramento Valley subbasins	Central Valley
ESAC-25	394500121480001	Middle Sacramento Valley	Eastern Sacramento Valley subbasins	Central Valley
ESAC-26	385429121472901	Middle Sacramento Valley	Eastern Sacramento Valley subbasins	Central Valley
ESAC-27	390900121350001	Middle Sacramento Valley	Eastern Sacramento Valley subbasins	Central Valley
ESAC-28	392200121413201	Middle Sacramento Valley	Eastern Sacramento Valley subbasins	Central Valley
ESAC-29	384736121411501	Middle Sacramento Valley	Eastern Sacramento Valley subbasins	Central Valley
ESAC-30	390347121501001	Middle Sacramento Valley	Eastern Sacramento Valley subbasins	Central Valley
ESAC-31	392000121560001	Middle Sacramento Valley	Eastern Sacramento Valley subbasins	Central Valley
ESAC-32	390946121341101	Middle Sacramento Valley	Eastern Sacramento Valley subbasins	Central Valley
ESAC-34	392910121451101	Middle Sacramento Valley	Eastern Sacramento Valley subbasins	Central Valley
ESAC-35[2]	393600121530001	Middle Sacramento Valley	Eastern Sacramento Valley subbasins	Central Valley
WSAC-03	395500122100001	Middle Sacramento Valley	Western Sacramento Valley subbasins	Central Valley
WSAC-04	395200122200001	Middle Sacramento Valley	Western Sacramento Valley subbasins	Central Valley
WSAC-06[2]	394600122210001	Middle Sacramento Valley	Western Sacramento Valley subbasins	Central Valley
WSAC-08	394435122110801	Middle Sacramento Valley	Western Sacramento Valley subbasins	Central Valley
WSAC-10[2]	394200122060001	Middle Sacramento Valley	Western Sacramento Valley subbasins	Central Valley
WSAC-11	390045122031701	Middle Sacramento Valley	Western Sacramento Valley subbasins	Central Valley
WSAC-12	391200122010001	Middle Sacramento Valley	Western Sacramento Valley subbasins	Central Valley
WSAC-14	391744122071301	Middle Sacramento Valley	Western Sacramento Valley subbasins	Central Valley
WSAC-15	384214121561101	Middle Sacramento Valley	Western Sacramento Valley subbasins	Central Valley
WSAC-17	385123121470801	Middle Sacramento Valley	Western Sacramento Valley subbasins	Central Valley
WSAC-18	390900122030001	Middle Sacramento Valley	Western Sacramento Valley subbasins	Central Valley
WSAC-21	385600122010001	Middle Sacramento Valley	Western Sacramento Valley subbasins	Central Valley
WSAC-22	390000122080001	Middle Sacramento Valley	Western Sacramento Valley subbasins	Central Valley
WSAC-25[2]	384700121450001	Middle Sacramento Valley	Western Sacramento Valley subbasins	Central Valley
WSAC-26	395400122190001	Middle Sacramento Valley	Western Sacramento Valley subbasins	Central Valley
WSAC-28	394500122140001	Middle Sacramento Valley	Western Sacramento Valley subbasins	Central Valley

Online resource (suppplementary table) 2. Selected attributes of GAMA-PBP (https://ca.water.usgs.gov/gama/) status wells sampled for orthophosphate concentration-page 38.

GAMA-PBP ID	USEPA Level III Ecoregions[4]	Level III Ecoregion Reference Concentration[4]	Status Sample Date	Status Sample Orthophosphate Concentration (mg/L as P)	Relative Concentration Category[5]	Redox state[6]	Elevation of LSD (meters above NAVD 88)[7]	Well depth (meters below LSD)[8]	Agricultural land use in 1974[9] (percent)	Natural land use in 1974[9] (percent)	Urban land use in 1974[9] (percent)
ESAC-18	Central California Foothills and Coastal Mountains	0.0300	Jul 20 2006	0.086	moderate	oxic	66	171	23.2%	0.0%	76.8%
ESAC-19	Central California Valley	0.0770	Jul 20 2006	0.090	moderate	oxic	14	81	84.1%	0.0%	15.9%
ESAC-21[2]	Central California Valley	0.0770	Jul 25 2006	0.120	high	anoxic	16	na	1.4%	97.7%	0.9%
ESAC-22	Central California Valley	0.0770	Jul 26 2006	0.053	low	oxic	32	27	68.3%	12.4%	19.3%
ESAC-25	Central California Foothills and Coastal Mountains	0.0300	Jul 27 2006	0.112	high	oxic	80	174	0.0%	0.0%	100.0%
ESAC-26	Central California Valley	0.0770	Jul 31 2006	0.499	high	anoxic	11	61	86.9%	12.1%	0.9%
ESAC-27	Central California Valley	0.0770	Aug 2 2006	0.100	high	oxic	20	41	0.5%	19.7%	79.8%
ESAC-28	Central California Valley	0.0770	Aug 3 2006	0.194	high	oxic	29	110	4.6%	0.0%	95.4%
ESAC-29	Central California Valley	0.0770	Aug 3 2006	0.240	high	anoxic	9	66	56.6%	42.9%	0.5%
ESAC-30	Central California Valley	0.0770	Aug 7 2006	0.256	high	anoxic	11	51	84.8%	14.3%	0.9%
ESAC-31	Central California Valley	0.0770	Aug 7 2006	0.101	high	anoxic	19	72	99.1%	0.9%	0.0%
ESAC-32	Central California Valley	0.0770	Aug 17 2006	0.090	moderate	oxic	20	43	6.8%	0.0%	93.2%
ESAC-34	Central California Valley	0.0770	Aug 17 2006	0.108	high	oxic	30	18	96.2%	2.8%	0.9%
ESAC-35[2]	Central California Valley	0.0770	Aug 24 2006	0.066	low	na	34	170	100.0%	0.0%	0.0%
WSAC-03	Central California Valley	0.0770	Jul 11 2006	0.036	low	oxic	84	72	5.0%	3.2%	91.8%
WSAC-04	Central California Foothills and Coastal Mountains	0.0300	Jul 11 2006	0.040	moderate	anoxic	138	268	0.0%	100.0%	0.0%
WSAC-06[2]	Central California Foothills and Coastal Mountains	0.0300	Jul 12 2006	0.028	low	oxic	148	na	0.0%	100.0%	0.0%
WSAC-08	Central California Valley	0.0770	Jul 18 2006	0.031	low	oxic	75	55	1.8%	0.0%	98.2%
WSAC-10[2]	Central California Valley	0.0770	Aug 10 2006	0.027	low	oxic	56	69	93.2%	3.6%	3.2%
WSAC-11	Central California Valley	0.0770	Jul 19 2006	0.065	low	oxic	43	174	21.4%	7.3%	71.4%
WSAC-12	Central California Valley	0.0770	Jul 19 2006	0.200	high	anoxic	15	139	47.9%	18.9%	33.2%
WSAC-14	Central California Valley	0.0770	Jul 24 2006	0.077	moderate	anoxic	18	48	90.3%	9.7%	0.0%
WSAC-15	Central California Valley	0.0770	Jul 31 2006	0.047	low	oxic	44	18	100.0%	0.0%	0.0%
WSAC-17	Central California Valley	0.0770	Aug 1 2006	0.257	high	oxic	8	79	79.1%	15.5%	5.5%
WSAC-18	Central California Valley	0.0770	Aug 1 2006	0.070	low	anoxic	26	123	3.2%	4.5%	92.3%
WSAC-21	Central California Valley	0.0770	Aug 2 2006	0.081	moderate	oxic	51	79	94.4%	5.6%	0.0%
WSAC-22	Central California Valley	0.0770	Aug 8 2006	0.047	low	oxic	108	265	61.2%	38.8%	0.0%
WSAC-25[2]	Central California Valley	0.0770	Aug 9 2006	0.101	high	anoxic	12	na	95.0%	0.0%	5.0%
WSAC-26	Central California Foothills and Coastal Mountains	0.0300	Aug 14 2006	0.049	moderate	oxic	125	101	45.5%	54.5%	0.0%
WSAC-28	Central California Valley	0.0770	Aug 15 2006	0.097	moderate	oxic	88	50	90.0%	7.3%	2.7%

Status and trends of orthophosphate concentrations in groundwater used for public supply in California *Environmental Monitoring and Assessment, Robert Kent, Tyler D. Johnson, and Michael R. Rosen, U.S. Geological Survey California Water Science Center-rhkent@usgs.gov*

Online resource (suppplementary table) 2. Selected attributes of GAMA-PBP (https://ca.water.usgs.gov/gama/) status wells sampled for orthophosphate concentration-page 39.

GAMA-PBP ID	Agricultural land use in 1982[9] (percent)	Natural land use in 1982[9] (percent)	Urban land use in 1982[9] (percent)	Agricultural land use in 1992[9] (percent)	Natural land use in 1992[9] (percent)	Urban land use in 1992[9] (percent)	Agricultural land use in 2002[9] (percent)	Natural land use in 2002[9] (percent)	Urban land use in 2002[9] (percent)	Agricultural land use in 2012[9] (percent)	Natural land use in 2012[9] (percent)	Urban land use in 2012[9] (percent)	Age Classification[10]	Septic Tanks[11]	Aridity[12]
ESAC-18	23.2%	0.0%	76.8%	0.0%	0.0%	100.0%	0.0%	0.0%	100.0%	0.0%	0.0%	100.0%	Premodern	108.17	0.556
ESAC-19	83.6%	0.0%	16.4%	83.6%	0.0%	16.4%	82.7%	0.0%	17.3%	82.7%	0.0%	17.3%	Premodern	23.04	0.424
ESAC-21[2]	16.8%	82.3%	0.9%	1.4%	97.3%	1.4%	23.2%	75.5%	1.4%	23.2%	75.5%	1.4%	Premodern	0.55	0.374
ESAC-22	73.4%	7.3%	19.3%	70.2%	10.6%	19.3%	73.4%	7.3%	19.3%	73.4%	7.3%	19.3%	Modern	4.50	0.493
ESAC-25	0.0%	0.0%	100.0%	0.0%	0.0%	100.0%	1.8%	0.0%	98.2%	1.8%	0.0%	98.2%	Premodern	137.68	0.579
ESAC-26	86.9%	12.1%	0.9%	86.0%	13.1%	0.9%	85.5%	13.6%	0.9%	85.5%	13.1%	1.4%	Premodern	0.45	0.390
ESAC-27	1.4%	18.8%	79.8%	1.4%	18.8%	79.8%	1.4%	18.8%	79.8%	1.4%	18.8%	79.8%	Modern	10.01	0.438
ESAC-28	4.2%	0.0%	95.8%	2.3%	0.0%	97.7%	2.3%	0.0%	97.7%	2.3%	0.0%	97.7%	Modern	16.87	0.454
ESAC-29	56.6%	42.9%	0.5%	51.1%	48.4%	0.5%	51.1%	48.4%	0.5%	51.1%	48.4%	0.5%	Mixed	0.97	0.378
ESAC-30	85.7%	13.4%	0.9%	85.7%	13.4%	0.9%	84.8%	13.4%	1.8%	84.8%	13.4%	1.8%	Premodern	0.81	0.397
ESAC-31	99.5%	0.5%	0.0%	99.5%	0.5%	0.0%	100.0%	0.0%	0.0%	100.0%	0.0%	0.0%	Mixed	0.55	0.401
ESAC-32	6.8%	0.0%	93.2%	6.8%	0.0%	93.2%	6.8%	0.0%	93.2%	6.8%	0.0%	93.2%	Modern	0.59	0.443
ESAC-34	96.2%	2.8%	0.9%	96.2%	2.8%	0.9%	95.8%	2.8%	1.4%	95.8%	2.8%	1.4%	Modern	0.26	0.490
ESAC-35[2]	100.0%	0.0%	0.0%	100.0%	0.0%	0.0%	99.1%	0.0%	0.9%	99.1%	0.0%	0.9%	ModernOrMixed	1.08	0.481
WSAC-03	5.0%	3.2%	91.8%	2.3%	3.2%	94.5%	2.3%	3.2%	94.5%	0.0%	2.7%	97.3%	Modern	1.83	0.479
WSAC-04	0.0%	100.0%	0.0%	0.0%	100.0%	0.0%	0.0%	100.0%	0.0%	0.0%	100.0%	0.0%	Premodern	0.50	0.458
WSAC-06[2]	0.0%	100.0%	0.0%	0.0%	100.0%	0.0%	0.0%	100.0%	0.0%	0.0%	100.0%	0.0%	Modern	0.25	0.456
WSAC-08	1.8%	0.0%	98.2%	1.8%	0.0%	98.2%	1.8%	0.0%	98.2%	1.8%	0.0%	98.2%	Mixed	35.50	0.452
WSAC-10[2]	92.8%	3.6%	3.6%	92.8%	3.6%	3.6%	92.3%	3.6%	4.1%	92.3%	3.2%	4.5%	Modern	5.06	0.451
WSAC-11	33.2%	7.3%	59.5%	20.5%	7.3%	72.3%	19.1%	7.3%	73.6%	19.1%	5.9%	75.0%	Premodern	0.96	0.375
WSAC-12	49.3%	16.1%	34.6%	41.9%	16.1%	41.9%	39.2%	13.4%	47.5%	39.2%	4.6%	56.2%	PremodernOrMixed	2.41	0.362
WSAC-14	90.3%	9.7%	0.0%	90.3%	9.7%	0.0%	91.7%	8.3%	0.0%	91.7%	7.8%	0.5%	Premodern	0.55	0.371
WSAC-15	100.0%	0.0%	0.0%	100.0%	0.0%	0.0%	100.0%	0.0%	0.0%	100.0%	0.0%	0.0%	Modern	0.76	0.421
WSAC-17	79.1%	15.5%	5.5%	77.7%	16.8%	5.5%	79.5%	15.0%	5.5%	79.5%	15.0%	5.5%	Mixed	0.42	0.385
WSAC-18	4.1%	4.5%	91.4%	3.6%	4.1%	92.3%	3.6%	4.1%	92.3%	3.6%	0.0%	96.4%	Mixed	1.08	0.342
WSAC-21	94.4%	5.6%	0.0%	94.4%	5.6%	0.0%	94.4%	5.6%	0.0%	94.4%	5.6%	0.0%	Mixed	1.40	0.395
WSAC-22	61.6%	38.4%	0.0%	61.6%	38.4%	0.0%	63.0%	37.0%	0.0%	63.0%	37.0%	0.0%	Mixed	0.69	0.388
WSAC-25[2]	95.0%	0.0%	5.0%	95.0%	0.0%	5.0%	95.0%	0.0%	5.0%	95.0%	0.0%	5.0%	Premodern	1.36	0.381
WSAC-26	45.5%	54.5%	0.0%	45.5%	54.5%	0.0%	45.5%	54.5%	0.0%	45.5%	54.5%	0.0%	Mixed	0.50	0.459
WSAC-28	90.0%	0.0%	10.0%	88.1%	0.0%	11.9%	94.1%	0.0%	5.9%	94.1%	0.0%	5.9%	Modern	24.42	0.452

Online resource (suppplementary table) 2. Selected attributes of GAMA-PBP (https://ca.water.usgs.gov/gama/) status wells sampled for orthophosphate concentration-page 40.

GAMA-PBP ID	USGS Station ID[1]	GAMA-PBP study unit	GAMA-PBP study area[3]	Hydrogeologic zone
WSAC-29	393149122122101	Middle Sacramento Valley	Western Sacramento Valley subbasins	Central Valley
WSAC-30[2]	391000122130001	Middle Sacramento Valley	Western Sacramento Valley subbasins	Central Valley
WSAC-31	391900122040001	Middle Sacramento Valley	Western Sacramento Valley subbasins	Central Valley
WSAC-34	393800122040001	Middle Sacramento Valley	Western Sacramento Valley subbasins	Central Valley
WSAC-35	394100122000001	Middle Sacramento Valley	Western Sacramento Valley subbasins	Central Valley
WSAC-36[2]	392700122020001	Middle Sacramento Valley	Western Sacramento Valley subbasins	Central Valley
ESJ-12	380113121062801	Northern San Joaquin Basin	Eastern San Joaquin subbasin	Central Valley
ESJ-19	375919121182401	Northern San Joaquin Basin	Eastern San Joaquin subbasin	Central Valley
NSJ-QPC-01[2]	380419120555101	Northern San Joaquin Basin	Northern San Joaquin Valley QPc area	Central Valley
TRCY-03	374100121260001	Northern San Joaquin Basin	Tracy subbasin	Central Valley
TRCY-07	375000121260001	Northern San Joaquin Basin	Tracy subbasin	Central Valley
NSAC-01	400800122070001	Northern Sacramento Valley	Northern Sacramento Valley subbasins	Central Valley
NSAC-02	400600122060001	Northern Sacramento Valley	Northern Sacramento Valley subbasins	Central Valley
NSAC-03[2]	400100122230001	Northern Sacramento Valley	Northern Sacramento Valley subbasins	Central Valley
NSAC-04	400800122120001	Northern Sacramento Valley	Northern Sacramento Valley subbasins	Central Valley
NSAC-05[2]	395800122100001	Northern Sacramento Valley	Northern Sacramento Valley subbasins	Central Valley
NSAC-06	400700122110001	Northern Sacramento Valley	Northern Sacramento Valley subbasins	Central Valley
NSAC-07	400800122180001	Northern Sacramento Valley	Northern Sacramento Valley subbasins	Central Valley
NSAC-08	401300122150001	Northern Sacramento Valley	Northern Sacramento Valley subbasins	Central Valley
NSAC-09	400900122130001	Northern Sacramento Valley	Northern Sacramento Valley subbasins	Central Valley
NSAC-10	401057122141501	Northern Sacramento Valley	Northern Sacramento Valley subbasins	Central Valley
NSAC-11	401100122110001	Northern Sacramento Valley	Northern Sacramento Valley subbasins	Central Valley
NSAC-12	400400122090001	Northern Sacramento Valley	Northern Sacramento Valley subbasins	Central Valley
NSAC-13	400000122050001	Northern Sacramento Valley	Northern Sacramento Valley subbasins	Central Valley
NSAC-14	400300122080001	Northern Sacramento Valley	Northern Sacramento Valley subbasins	Central Valley
NSAC-15	400400122050001	Northern Sacramento Valley	Northern Sacramento Valley subbasins	Central Valley
NSAC-16	400118122054501	Northern Sacramento Valley	Northern Sacramento Valley subbasins	Central Valley
NSAC-17[2]	395500122260001	Northern Sacramento Valley	Northern Sacramento Valley subbasins	Central Valley
NSAC-18	401200122180001	Northern Sacramento Valley	Northern Sacramento Valley subbasins	Central Valley
NSAC-19	400900122210001	Northern Sacramento Valley	Northern Sacramento Valley subbasins	Central Valley

Status and trends of orthophosphate concentrations in groundwater used for public supply in California *Environmental Monitoring and Assessment, Robert Kent, Tyler D. Johnson, and Michael R. Rosen, U.S. Geological Survey California Water Science Center-rhkent@usgs.gov*

Online resource (suppplementary table) 2. Selected attributes of GAMA-PBP (https://ca.water.usgs.gov/gama/) status wells sampled for orthophosphate concentration-page 41.

GAMA-PBP ID	USEPA Level III Ecoregions[4]	Level III Ecoregion Reference Concentration[4]	Status Sample Date	Status Sample Orthophosphate Concentration (mg/L as P)	Relative Concentration Category[5]	Redox state[6]	Elevation of LSD (meters above NAVD 88)[7]	Well depth (meters below LSD)[8]	Agricultural land use in 1974[9] (percent)	Natural land use in 1974[9] (percent)	Urban land use in 1974[9] (percent)
WSAC-29	Central California Valley	0.0770	Aug 16 2006	0.043	low	oxic	43	231	20.3%	3.7%	76.0%
WSAC-30[2]	Central California Valley	0.0770	Aug 16 2006	0.037	low	oxic	37	na	98.6%	0.9%	0.5%
WSAC-31	Central California Valley	0.0770	Aug 21 2006	0.077	low	oxic	18	75	92.2%	7.8%	0.0%
WSAC-34	Central California Valley	0.0770	Aug 22 2006	0.032	low	oxic	43	55	98.6%	0.9%	0.5%
WSAC-35	Central California Valley	0.0770	Aug 23 2006	0.036	low	oxic	43	125	94.5%	5.5%	0.0%
WSAC-36[2]	Central California Valley	0.0770	Aug 23 2006	0.103	high	na	24	79	100.0%	0.0%	0.0%
ESJ-12	Central California Valley	0.0770	Jan 13 2005	0.046	low	oxic	26	82	96.8%	0.0%	3.2%
ESJ-19	Central California Valley	0.0770	Feb 18 2005	0.054	low	oxic	5	158	0.0%	0.0%	100.0%
NSJ-QPC-01[2]	Central California Valley	0.0770	Jan 11 2005	0.044	low	oxic	55	na	56.7%	42.9%	0.5%
TRCY-03	Central California Valley	0.0770	Jan 6 2005	0.016	low	oxic	63	274	11.0%	49.8%	39.3%
TRCY-07	Central California Valley	0.0770	Feb 8 2005	0.227	high	anoxic	1	25	95.4%	0.0%	4.6%
NSAC-01	Central California Valley	0.0770	Oct 4 2007	0.058	low	oxic	77	56	77.6%	8.2%	14.2%
NSAC-02	Central California Valley	0.0770	Oct 24 2007	0.067	low	oxic	73	26	84.9%	6.4%	8.7%
NSAC-03[2]	Central California Foothills and Coastal Mountains	0.0300	Oct 29 2007	0.032	moderate	oxic	157	na	0.0%	85.3%	14.7%
NSAC-04	Central California Valley	0.0770	Oct 30 2007	0.105	high	oxic	90	65	12.8%	22.8%	64.4%
NSAC-05[2]	Central California Valley	0.0770	Oct 31 2007	0.040	low	oxic	78	na	7.0%	78.5%	14.5%
NSAC-06	Central California Valley	0.0770	Oct 31 2007	0.076	low	oxic	87	48	42.6%	48.6%	8.8%
NSAC-07	Central California Foothills and Coastal Mountains	0.0300	Nov 1 2007	0.039	moderate	oxic	147	137	3.2%	88.1%	8.7%
NSAC-08	Central California Foothills and Coastal Mountains	0.0300	Nov 5 2007	0.055	moderate	anoxic	128	88	0.0%	48.6%	51.4%
NSAC-09	Central California Valley	0.0770	Nov 6 2007	0.052	low	oxic	96	155	0.0%	12.0%	88.0%
NSAC-10	Central California Valley	0.0770	Nov 6 2007	0.078	moderate	oxic	83	91	0.0%	10.3%	89.7%
NSAC-11	Central California Valley	0.0770	Nov 26 2007	0.042	low	oxic	81	24	59.4%	2.3%	38.4%
NSAC-12	Central California Valley	0.0770	Nov 27 2007	0.057	low	oxic	77	73	49.8%	29.7%	20.5%
NSAC-13	Central California Valley	0.0770	Nov 27 2007	0.107	high	oxic	63	67	86.4%	9.3%	4.2%
NSAC-14	Central California Valley	0.0770	Nov 28 2007	0.074	low	oxic	69	131	46.6%	0.5%	53.0%
NSAC-15	Central California Valley	0.0770	Nov 28 2007	0.032	low	oxic	72	79	93.2%	1.4%	5.5%
NSAC-16	Central California Valley	0.0770	Dec 6 2007	0.139	high	oxic	66	94	39.4%	0.0%	60.6%
NSAC-17[2]	Central California Foothills and Coastal Mountains	0.0300	Dec 13 2007	0.086	moderate	anoxic	181	na	66.5%	33.0%	0.5%
NSAC-18	Central California Foothills and Coastal Mountains	0.0300	Dec 18 2007	0.080	moderate	oxic	149	73	0.0%	99.1%	0.9%
NSAC-19	Central California Foothills and Coastal Mountains	0.0300	Jan 8 2008	0.035	moderate	oxic	132	103	23.4%	75.2%	1.4%

Status and trends of orthophosphate concentrations in groundwater used for public supply in California *Environmental Monitoring and Assessment, Robert Kent, Tyler D. Johnson, and Michael R. Rosen, U.S. Geological Survey California Water Science Center-rhkent@usgs.gov*

Online resource (suppplementary table) 2. Selected attributes of GAMA-PBP (https://ca.water.usgs.gov/gama/) status wells sampled for orthophosphate concentration-page 42.

GAMA-PBP ID	Agricultural land use in 1982[9] (percent)	Natural land use in 1982[9] (percent)	Urban land use in 1982[9] (percent)	Agricultural land use in 1992[9] (percent)	Natural land use in 1992[9] (percent)	Urban land use in 1992[9] (percent)	Agricultural land use in 2002[9] (percent)	Natural land use in 2002[9] (percent)	Urban land use in 2002[9] (percent)	Agricultural land use in 2012[9] (percent)	Natural land use in 2012[9] (percent)	Urban land use in 2012[9] (percent)	Age Classification[10]	Septic Tanks[11]	Aridity[12]
WSAC-29	14.7%	1.4%	83.9%	10.6%	1.4%	88.0%	12.9%	0.5%	86.6%	12.9%	0.5%	86.6%	Premodern	39.01	0.386
WSAC-30[2]	98.6%	0.9%	0.5%	98.6%	0.9%	0.5%	98.6%	0.9%	0.5%	98.6%	0.9%	0.5%	Mixed	0.88	0.356
WSAC-31	95.4%	4.6%	0.0%	95.4%	4.6%	0.0%	96.3%	3.7%	0.0%	96.3%	3.7%	0.0%	Premodern	0.75	0.384
WSAC-34	98.6%	0.9%	0.5%	98.6%	0.9%	0.5%	98.6%	0.9%	0.5%	98.6%	0.9%	0.5%	Modern	2.13	0.431
WSAC-35	94.5%	5.5%	0.0%	94.5%	5.5%	0.0%	94.5%	5.5%	0.0%	94.5%	5.1%	0.5%	Modern	1.85	0.459
WSAC-36[2]	100.0%	0.0%	0.0%	100.0%	0.0%	0.0%	100.0%	0.0%	0.0%	100.0%	0.0%	0.0%	Mixed	0.76	0.409
ESJ-12	96.8%	0.0%	3.2%	96.8%	0.0%	3.2%	96.8%	0.0%	3.2%	96.8%	0.0%	3.2%	Premodern	4.61	0.311
ESJ-19	0.0%	0.0%	100.0%	0.0%	0.0%	100.0%	0.0%	0.0%	100.0%	0.0%	0.0%	100.0%	ModernOrMixed	1.18	0.317
NSJ-QPC-01[2]	56.7%	42.9%	0.5%	55.8%	43.8%	0.5%	56.7%	42.9%	0.5%	56.7%	42.9%	0.5%	Modern	1.27	0.355
TRCY-03	17.8%	35.2%	47.0%	9.1%	40.2%	50.7%	8.7%	33.8%	57.5%	7.3%	25.6%	67.1%	Premodern	0.56	0.192
TRCY-07	95.0%	0.0%	5.0%	93.2%	0.0%	6.8%	92.7%	0.0%	7.3%	92.7%	0.0%	7.3%	Modern	0.92	0.269
NSAC-01	77.6%	8.2%	14.2%	77.6%	8.2%	14.2%	77.2%	8.2%	14.6%	77.2%	6.4%	16.4%	Modern	12.47	0.520
NSAC-02	83.5%	6.4%	10.1%	82.6%	6.4%	11.0%	81.2%	6.4%	12.4%	81.2%	6.4%	12.4%	Modern	7.44	0.509
NSAC-03[2]	0.0%	85.3%	14.7%	0.0%	85.3%	14.7%	0.0%	85.3%	14.7%	0.0%	85.3%	14.7%	Mixed	0.61	0.540
NSAC-04	12.8%	22.8%	64.4%	12.3%	22.8%	64.8%	12.3%	22.8%	64.8%	12.3%	22.4%	65.3%	Modern	5.09	0.535
NSAC-05[2]	6.5%	78.5%	15.0%	6.5%	78.0%	15.4%	5.6%	78.0%	16.4%	5.6%	78.0%	16.4%	Mixed	11.24	0.493
NSAC-06	42.6%	48.1%	9.3%	41.7%	47.7%	10.6%	41.7%	47.7%	10.6%	41.7%	47.7%	10.6%	Modern	4.82	0.523
NSAC-07	3.2%	88.1%	8.7%	3.2%	88.1%	8.7%	3.2%	88.1%	8.7%	3.2%	88.1%	8.7%	Mixed	0.80	0.581
NSAC-08	0.0%	48.6%	51.4%	0.0%	47.2%	52.8%	0.0%	47.2%	52.8%	0.0%	46.8%	53.2%	Premodern	2.33	0.588
NSAC-09	0.0%	12.0%	88.0%	0.0%	12.0%	88.0%	0.0%	12.0%	88.0%	0.0%	12.0%	88.0%	Mixed	5.71	0.552
NSAC-10	0.0%	10.3%	89.7%	0.0%	10.3%	89.7%	0.0%	10.3%	89.7%	0.0%	9.8%	90.2%	Mixed	18.78	0.551
NSAC-11	56.2%	1.4%	42.5%	52.5%	4.6%	42.9%	52.1%	4.6%	43.4%	52.1%	4.6%	43.4%	Modern	30.44	0.533
NSAC-12	48.4%	29.7%	21.9%	48.4%	29.7%	21.9%	49.3%	28.8%	21.9%	52.5%	28.3%	19.2%	Premodern	19.89	0.501
NSAC-13	86.4%	9.3%	4.2%	84.1%	11.2%	4.7%	83.2%	11.2%	5.6%	83.2%	11.2%	5.6%	Premodern	3.83	0.500
NSAC-14	42.9%	0.5%	56.6%	42.0%	0.5%	57.5%	42.0%	0.5%	57.5%	42.0%	0.5%	57.5%	Mixed	26.11	0.500
NSAC-15	92.7%	1.4%	5.9%	92.2%	1.4%	6.4%	88.1%	0.9%	11.0%	86.3%	2.7%	11.0%	Modern	7.44	0.506
NSAC-16	36.7%	0.0%	63.3%	32.6%	0.0%	67.4%	31.2%	0.0%	68.8%	31.2%	0.0%	68.8%	Mixed	52.10	0.499
NSAC-17[2]	66.5%	33.0%	0.5%	66.5%	33.0%	0.5%	67.0%	32.6%	0.5%	67.0%	32.6%	0.5%	Premodern	0.50	0.497
NSAC-18	0.0%	99.1%	0.9%	0.0%	99.1%	0.9%	0.0%	97.7%	2.3%	0.0%	97.7%	2.3%	Mixed	3.31	0.603
NSAC-19	23.4%	75.2%	1.4%	17.4%	81.2%	1.4%	16.5%	82.1%	1.4%	16.5%	82.1%	1.4%	Mixed	1.54	0.573

Online resource (suppplementary table) 2. Selected attributes of GAMA-PBP (https://ca.water.usgs.gov/gama/) status wells sampled for orthophosphate concentration-page 43.

GAMA-PBP ID	USGS Station ID[1]	GAMA-PBP study unit	GAMA-PBP study area[3]	Hydrogeologic zone
NSAC-20	400500122130001	Northern Sacramento Valley	Northern Sacramento Valley subbasins	Central Valley
RED-01	402000122130001	Northern Sacramento Valley	Redding area basin	Central Valley
RED-02	402200122120001	Northern Sacramento Valley	Redding area basin	Central Valley
RED-03	403200122190001	Northern Sacramento Valley	Redding area basin	Central Valley
RED-04	403000122180001	Northern Sacramento Valley	Redding area basin	Central Valley
RED-05	402300122170001	Northern Sacramento Valley	Redding area basin	Central Valley
RED-06	403000122220001	Northern Sacramento Valley	Redding area basin	Central Valley
RED-07	402800122160001	Northern Sacramento Valley	Redding area basin	Central Valley
RED-08	403300122180001	Northern Sacramento Valley	Redding area basin	Central Valley
RED-09	402100122190001	Northern Sacramento Valley	Redding area basin	Central Valley
RED-10	402400122160001	Northern Sacramento Valley	Redding area basin	Central Valley
RED-11	402700122200001	Northern Sacramento Valley	Redding area basin	Central Valley
RED-12	403300122140001	Northern Sacramento Valley	Redding area basin	Central Valley
RED-13	402300122200001	Northern Sacramento Valley	Redding area basin	Central Valley
RED-14	402300122240001	Northern Sacramento Valley	Redding area basin	Central Valley
RED-15	402500122250001	Northern Sacramento Valley	Redding area basin	Central Valley
RED-16	401900122220001	Northern Sacramento Valley	Redding area basin	Central Valley
RED-17	402500122110001	Northern Sacramento Valley	Redding area basin	Central Valley
RED-18[2]	402900122160001	Northern Sacramento Valley	Redding area basin	Central Valley
RED-19	400900122084301	Northern Sacramento Valley	Redding area basin	Central Valley
RED-20	401900122160001	Northern Sacramento Valley	Redding area basin	Central Valley
RED-21[2]	402500122130001	Northern Sacramento Valley	Redding area basin	Central Valley
RED-22	402300122150002	Northern Sacramento Valley	Redding area basin	Central Valley
RED-23	403300122120001	Northern Sacramento Valley	Redding area basin	Central Valley
NAM-01[2]	384100121350001	Southern Sacramento Valley	North American subbasin	Central Valley
NAM-02[2]	383703121280701	Southern Sacramento Valley	North American subbasin	Central Valley
NAM-05[2]	384224121261401	Southern Sacramento Valley	North American subbasin	Central Valley
NAM-06	384100121310003	Southern Sacramento Valley	North American subbasin	Central Valley
NAM-08[2]	385340121255701	Southern Sacramento Valley	North American subbasin	Central Valley
SAM-02[2]	382501121222201	Southern Sacramento Valley	South American subbasin	Central Valley

Status and trends of orthophosphate concentrations in groundwater used for public supply in California *Environmental Monitoring and Assessment, Robert Kent, Tyler D. Johnson, and Michael R. Rosen, U.S. Geological Survey California Water Science Center-rhkent@usgs.gov*

Online resource (suppplementary table) 2. Selected attributes of GAMA-PBP (https://ca.water.usgs.gov/gama/) status wells sampled for orthophosphate concentration-page 44.

GAMA-PBP ID	USEPA Level III Ecoregions[4]	Level III Ecoregion Reference Concentration[4]	Status Sample Date	Status Sample Orthophosphate Concentration (mg/L as P)	Relative Concentration Category[5]	Redox state[6]	Elevation of LSD (meters above NAVD 88)[7]	Well depth (meters below LSD)[8]	Agricultural land use in 1974[9] (percent)	Natural land use in 1974[9] (percent)	Urban land use in 1974[9] (percent)
NSAC-20	Central California Valley	0.0770	Jan 15 2008	0.087	moderate	oxic	94	62	48.6%	49.1%	2.3%
RED-01	Central California Foothills and Coastal Mountains	0.0300	Oct 1 2007	0.128	high	oxic	215	127	0.0%	98.6%	1.4%
RED-02	Central California Foothills and Coastal Mountains	0.0300	Oct 1 2007	0.106	high	oxic	166	145	0.0%	98.2%	1.8%
RED-03	Central California Foothills and Coastal Mountains	0.0300	Oct 2 2007	0.065	moderate	oxic	161	155	0.0%	0.0%	100.0%
RED-04	Central California Foothills and Coastal Mountains	0.0300	Oct 3 2007	0.044	moderate	anoxic	150	120	5.0%	0.5%	94.5%
RED-05	Central California Foothills and Coastal Mountains	0.0300	Oct 3 2007	0.184	high	oxic	146	150	1.4%	1.4%	97.2%
RED-06	Central California Foothills and Coastal Mountains	0.0300	Oct 22 2007	0.045	moderate	oxic	140	9	26.5%	1.8%	71.7%
RED-07	Central California Foothills and Coastal Mountains	0.0300	Oct 23 2007	0.131	high	oxic	145	61	26.8%	55.5%	17.7%
RED-08	Central California Foothills and Coastal Mountains	0.0300	Oct 23 2007	0.161	high	oxic	174	71	0.0%	3.7%	96.3%
RED-09	Central California Foothills and Coastal Mountains	0.0300	Oct 25 2007	0.103	high	oxic	154	61	35.6%	58.9%	5.5%
RED-10	Central California Foothills and Coastal Mountains	0.0300	Oct 25 2007	0.075	moderate	oxic	192	162	0.0%	85.3%	14.7%
RED-11	Central California Foothills and Coastal Mountains	0.0300	Nov 7 2007	0.178	high	oxic	146	108	7.3%	1.4%	91.4%
RED-12	Central California Foothills and Coastal Mountains	0.0300	Nov 8 2007	0.156	high	anoxic	139	110	20.5%	7.8%	71.7%
RED-13	Central California Foothills and Coastal Mountains	0.0300	Nov 20 2007	0.116	high	oxic	145	131	22.4%	11.0%	66.7%
RED-14	Central California Foothills and Coastal Mountains	0.0300	Nov 29 2007	0.064	moderate	anoxic	159	137	90.0%	3.2%	6.8%
RED-15	Central California Foothills and Coastal Mountains	0.0300	Dec 3 2007	0.151	high	oxic	230	112	0.0%	100.0%	0.0%
RED-16	Central California Foothills and Coastal Mountains	0.0300	Dec 3 2007	0.057	moderate	oxic	158	32	0.0%	96.3%	3.7%
RED-17	Central California Foothills and Coastal Mountains	0.0300	Dec 5 2007	0.092	moderate	oxic	115	43	57.7%	39.1%	3.3%
RED-18[2]	Central California Foothills and Coastal Mountains	0.0300	Dec 5 2007	0.092	moderate	oxic	142	na	20.3%	49.8%	30.0%
RED-19	Central California Foothills and Coastal Mountains	0.0300	Dec 11 2007	0.056	moderate	oxic	187	91	14.0%	85.1%	0.9%
RED-20	Central California Foothills and Coastal Mountains	0.0300	Dec 12 2007	0.155	high	oxic	176	108	0.5%	92.7%	6.8%
RED-21[2]	Central California Foothills and Coastal Mountains	0.0300	Dec 12 2007	0.128	high	oxic	129	na	89.6%	4.1%	6.3%
RED-22	Central California Foothills and Coastal Mountains	0.0300	Jan 15 2008	0.107	high	oxic	125	36	64.4%	32.9%	2.7%
RED-23	Central California Foothills and Coastal Mountains	0.0300	Jan 16 2008	0.096	moderate	oxic	142	41	47.9%	51.1%	0.9%
NAM-01[2]	Central California Valley	0.0770	Mar 28 2005	0.251	high	na	6	143	19.2%	0.9%	79.9%
NAM-02[2]	Central California Valley	0.0770	Mar 29 2005	0.032	low	na	10	114	0.0%	0.0%	100.0%
NAM-05[2]	Central California Valley	0.0770	Apr 7 2005	0.025	low	na	20	158	8.3%	1.8%	89.9%
NAM-06	Central California Valley	0.0770	Apr 14 2005	0.125	high	anoxic	9	152	91.8%	0.9%	7.3%
NAM-08[2]	Central California Valley	0.0770	May 4 2005	0.094	moderate	na	24	168	26.0%	73.5%	0.5%
SAM-02[2]	Central California Valley	0.0770	Mar 15 2005	0.064	low	na	15	156	3.2%	0.0%	96.8%

Online resource (suppplementary table) 2. Selected attributes of GAMA-PBP (https://ca.water.usgs.gov/gama/) status wells sampled for orthophosphate concentration-page 45.

GAMA-PBP ID	Agricultural land use in 1982[9] (percent)	Natural land use in 1982[9] (percent)	Urban land use in 1982[9] (percent)	Agricultural land use in 1992[9] (percent)	Natural land use in 1992[9] (percent)	Urban land use in 1992[9] (percent)	Agricultural land use in 2002[9] (percent)	Natural land use in 2002[9] (percent)	Urban land use in 2002[9] (percent)	Agricultural land use in 2012[9] (percent)	Natural land use in 2012[9] (percent)	Urban land use in 2012[9] (percent)	Age Classification[10]	Septic Tanks[11]	Aridity[12]
NSAC-20	48.6%	49.1%	2.3%	48.6%	49.1%	2.3%	55.6%	40.3%	4.2%	55.6%	39.8%	4.6%	Premodern	4.82	0.523
RED-01	0.0%	98.6%	1.4%	0.0%	98.6%	1.4%	0.0%	97.7%	2.3%	0.0%	97.2%	2.8%	Premodern	2.39	0.646
RED-02	0.0%	97.7%	2.3%	0.0%	97.2%	2.8%	0.0%	96.3%	3.7%	0.0%	96.3%	3.7%	Premodern	2.39	0.599
RED-03	0.0%	0.0%	100.0%	0.0%	0.0%	100.0%	0.0%	0.0%	100.0%	0.0%	0.0%	100.0%	Modern	39.15	0.803
RED-04	5.0%	0.5%	94.5%	2.7%	0.5%	96.8%	6.8%	0.0%	93.2%	6.8%	0.0%	93.2%	Modern	23.56	0.769
RED-05	0.5%	0.0%	99.5%	0.5%	0.0%	99.5%	0.0%	0.0%	100.0%	0.0%	0.0%	100.0%	Premodern	7.64	0.655
RED-06	19.2%	1.8%	79.0%	17.8%	1.8%	80.4%	22.8%	1.8%	75.3%	22.8%	1.8%	75.3%	Modern	31.90	0.784
RED-07	24.5%	21.4%	54.1%	24.1%	21.4%	54.5%	25.5%	20.5%	54.1%	25.5%	20.5%	54.1%	Modern	23.32	0.730
RED-08	0.0%	1.4%	98.6%	0.0%	1.4%	98.6%	0.0%	1.4%	98.6%	0.0%	1.4%	98.6%	Modern	39.02	0.805
RED-09	35.2%	58.9%	5.9%	35.2%	58.9%	5.9%	38.8%	55.3%	5.9%	38.8%	55.3%	5.9%	Modern	7.37	0.658
RED-10	0.0%	85.3%	14.7%	0.0%	84.8%	15.2%	0.0%	84.8%	15.2%	0.0%	84.8%	15.2%	Premodern	10.18	0.703
RED-11	7.3%	1.4%	91.4%	7.3%	1.4%	91.4%	7.3%	1.4%	91.4%	7.3%	1.4%	91.4%	Modern	71.14	0.739
RED-12	10.5%	8.2%	81.3%	10.5%	8.2%	81.3%	23.7%	7.3%	68.9%	23.3%	7.3%	69.4%	Mixed	17.29	0.765
RED-13	19.6%	8.7%	71.7%	19.6%	8.7%	71.7%	19.6%	8.7%	71.7%	19.6%	8.7%	71.7%	Mixed	14.31	0.664
RED-14	89.5%	3.2%	7.3%	89.5%	3.2%	7.3%	90.4%	1.4%	8.2%	90.4%	1.4%	8.2%	Premodern	12.34	0.668
RED-15	0.0%	100.0%	0.0%	0.0%	100.0%	0.0%	0.0%	99.5%	0.5%	0.0%	99.5%	0.5%	Mixed	8.13	0.785
RED-16	0.0%	96.3%	3.7%	0.0%	96.3%	3.7%	0.0%	96.3%	3.7%	0.0%	96.3%	3.7%	Modern	3.59	0.656
RED-17	57.2%	39.1%	3.7%	57.2%	34.4%	8.4%	57.7%	33.5%	8.8%	59.5%	30.2%	10.2%	Premodern	7.75	0.593
RED-18[2]	16.6%	29.0%	54.4%	16.6%	29.0%	54.4%	18.9%	29.0%	52.1%	17.5%	29.5%	53.0%	Modern	19.17	0.750
RED-19	14.0%	85.1%	0.9%	14.0%	85.1%	0.9%	19.1%	79.5%	1.4%	19.1%	79.5%	1.4%	Modern	1.46	0.615
RED-20	0.5%	92.7%	6.8%	0.5%	92.7%	6.8%	7.8%	85.4%	6.8%	7.8%	85.4%	6.8%	Premodern	2.58	0.697
RED-21[2]	86.9%	5.4%	7.7%	86.4%	2.3%	11.3%	86.9%	0.5%	12.7%	86.9%	0.0%	13.1%	Modern	11.11	0.599
RED-22	64.4%	32.9%	2.7%	63.9%	11.4%	24.7%	63.5%	11.4%	25.1%	62.6%	12.3%	25.1%	Modern	9.94	0.607
RED-23	47.9%	51.1%	0.9%	47.9%	51.1%	0.9%	55.7%	43.4%	0.9%	55.7%	43.4%	0.9%	Modern	1.39	0.738
NAM-01[2]	10.0%	0.5%	89.5%	6.4%	0.0%	93.6%	2.7%	1.4%	95.9%	2.7%	0.0%	97.3%	ModernOrMixed	1.76	0.395
NAM-02[2]	0.0%	0.0%	100.0%	0.0%	0.0%	100.0%	0.0%	0.0%	100.0%	0.0%	0.0%	100.0%	ModernOrMixed	3.09	0.396
NAM-05[2]	7.8%	0.9%	91.2%	0.5%	1.8%	97.7%	0.0%	0.0%	100.0%	0.0%	0.0%	100.0%	PremodernOrMixed	69.88	0.437
NAM-06	91.8%	0.9%	7.3%	91.8%	0.5%	7.8%	91.8%	0.0%	8.2%	63.9%	0.0%	36.1%	Premodern	2.64	0.405
NAM-08[2]	26.0%	73.5%	0.5%	26.0%	73.5%	0.5%	26.0%	73.5%	0.5%	26.0%	73.5%	0.5%	ModernOrMixed	1.73	0.422
SAM-02[2]	0.5%	0.0%	99.5%	0.0%	0.0%	100.0%	0.0%	0.0%	100.0%	0.0%	0.0%	100.0%	ModernOrMixed	1.38	0.372

Online resource (suppplementary table) 2. Selected attributes of GAMA-PBP (https://ca.water.usgs.gov/gama/) status wells sampled for orthophosphate concentration-page 46.

GAMA-PBP ID	USGS Station ID[1]	GAMA-PBP study unit	GAMA-PBP study area[3]	Hydrogeologic zone
SAM-03[2]	382843121322901	Southern Sacramento Valley	South American subbasin	Central Valley
SAM-07[2]	383514121184001	Southern Sacramento Valley	South American subbasin	Central Valley
SAM-11[2]	382952121234401	Southern Sacramento Valley	South American subbasin	Central Valley
SOL-01[2]	380935121421601	Southern Sacramento Valley	Solano subbasin	Central Valley
SOL-03[2]	382100121560001	Southern Sacramento Valley	Solano subbasin	Central Valley
SOL-06[2]	381426121304001	Southern Sacramento Valley	Solano subbasin	Central Valley
SSV-QPC-02[2]	384112121130601	Southern Sacramento Valley	Southern Sacramento Valley QPc area	Central Valley
SSV-QPC-05[2]	383602121212701	Southern Sacramento Valley	Southern Sacramento Valley QPc area	Central Valley
SSV-QPC-06	384100121210001	Southern Sacramento Valley	Southern Sacramento Valley QPc area	Central Valley
SSV-QPC-07	384413121163801	Southern Sacramento Valley	Southern Sacramento Valley QPc area	Central Valley
SSV-QPC-08[2]	385856121222501	Southern Sacramento Valley	Southern Sacramento Valley QPc area	Central Valley
SSV-QPC-09[2]	383300121120001	Southern Sacramento Valley	Southern Sacramento Valley QPc area	Central Valley
SUI-01[2]	380800121490001	Southern Sacramento Valley	Suisun subbasin	Central Valley
SUI-02[2]	381400122020001	Southern Sacramento Valley	Suisun subbasin	Central Valley
YOL-02[2]	383400121460001	Southern Sacramento Valley	Yolo subbasin	Central Valley
YOL-03	383155121452001	Southern Sacramento Valley	Yolo subbasin	Central Valley
YOL-04	383121121575301	Southern Sacramento Valley	Yolo subbasin	Central Valley
YOL-06[2]	384000121520001	Southern Sacramento Valley	Yolo subbasin	Central Valley
YOL-08	383425121341801	Southern Sacramento Valley	Yolo subbasin	Central Valley
YOL-09[2]	383900121360001	Southern Sacramento Valley	Yolo subbasin	Central Valley
YOL-13[2]	384000121590001	Southern Sacramento Valley	Yolo subbasin	Central Valley
YOL-14	383308121322801	Southern Sacramento Valley	Yolo subbasin	Central Valley
DM-01	363907120144401	Western San Joaquin Valley	Delta-Mendota subbasin	Central Valley
DM-02	364632120223403	Western San Joaquin Valley	Delta-Mendota subbasin	Central Valley
DM-03	370310120510801	Western San Joaquin Valley	Delta-Mendota subbasin	Central Valley
DM-04	370200120380001	Western San Joaquin Valley	Delta-Mendota subbasin	Central Valley
DM-05	365100120270001	Western San Joaquin Valley	Delta-Mendota subbasin	Central Valley
DM-06	372800121070001	Western San Joaquin Valley	Delta-Mendota subbasin	Central Valley
DM-07	370800120570001	Western San Joaquin Valley	Delta-Mendota subbasin	Central Valley
DM-08	370602121002301	Western San Joaquin Valley	Delta-Mendota subbasin	Central Valley

Online resource (suppplementary table) 2. Selected attributes of GAMA-PBP (https://ca.water.usgs.gov/gama/) status wells sampled for orthophosphate concentration-page 47.

GAMA-PBP ID	USEPA Level III Ecoregions[4]	Level III Ecoregion Reference Concentration[4]	Status Sample Date	Status Sample Orthophosphate Concentration (mg/L as P)	Relative Concentration Category[5]	Redox state[6]	Elevation of LSD (meters above NAVD 88)[7]	Well depth (meters below LSD)[8]	Agricultural land use in 1974[9] (percent)	Natural land use in 1974[9] (percent)	Urban land use in 1974[9] (percent)
SAM-03[2]	Central California Valley	0.0770	Mar 22 2005	0.218	high	na	3	67	46.6%	40.2%	13.2%
SAM-07[2]	Central California Valley	0.0770	Apr 5 2005	0.025	low	na	26	94	0.0%	0.0%	100.0%
SAM-11[2]	Central California Valley	0.0770	Apr 21 2005	0.050	low	na	13	82	8.3%	0.0%	91.7%
SOL-01[2]	Central California Valley	0.0770	Mar 16 2005	0.123	high	na	4	244	62.3%	0.5%	37.3%
SOL-03[2]	Central California Valley	0.0770	Mar 23 2005	0.023	low	na	33	287	70.4%	0.0%	29.6%
SOL-06[2]	Central California Valley	0.0770	Mar 30 2005	0.225	high	na	0	74	58.9%	10.0%	31.1%
SSV-QPC-02[2]	Central California Valley	0.0770	Mar 16 2005	0.058	low	na	77	63	2.7%	0.0%	97.3%
SSV-QPC-05[2]	Central California Valley	0.0770	Mar 22 2005	0.052	low	na	32	91	0.0%	0.0%	100.0%
SSV-QPC-06	Central California Valley	0.0770	Mar 22 2005	0.097	moderate	oxic	37	165	14.6%	0.0%	85.4%
SSV-QPC-07	Central California Valley	0.0770	Apr 4 2005	0.084	moderate	oxic	52	92	8.4%	0.0%	91.6%
SSV-QPC-08[2]	Central California Valley	0.0770	Apr 6 2005	0.091	moderate	na	33	48	12.8%	32.4%	54.8%
SSV-QPC-09[2]	Central California Valley	0.0770	Apr 7 2005	0.118	high	na	5	90	0.0%	80.3%	19.7%
SUI-01[2]	Central California Valley	0.0770	Mar 31 2005	0.043	low	na	47	na	41.9%	55.8%	2.3%
SUI-02[2]	Central California Valley	0.0770	Apr 20 2005	0.084	moderate	na	7	na	16.9%	56.8%	26.3%
YOL-02[2]	Central California Valley	0.0770	Apr 18 2005	0.108	high	na	16	34	69.5%	0.9%	29.5%
YOL-03	Central California Valley	0.0770	Apr 19 2005	0.052	low	anoxic	18	442	31.1%	4.1%	64.8%
YOL-04	Central California Valley	0.0770	Apr 26 2005	0.045	low	oxic	40	82	49.5%	8.6%	41.8%
YOL-06[2]	Central California Valley	0.0770	Apr 27 2005	0.169	high	na	41	120	38.6%	16.4%	45.0%
YOL-08	Central California Valley	0.0770	May 10 2005	0.127	high	anoxic	4	120	13.7%	7.3%	79.0%
YOL-09[2]	Central California Valley	0.0770	May 17 2005	0.219	high	na	8	na	67.4%	26.7%	5.9%
YOL-13[2]	Central California Valley	0.0770	May 24 2005	0.055	low	na	57	57	98.1%	0.0%	1.9%
YOL-14	Central California Valley	0.0770	May 25 2005	0.027	low	anoxic	5	411	31.3%	10.1%	58.5%
DM-01	Central California Valley	0.0770	Mar 1 2010	0.083	moderate	anoxic	51	278	58.2%	1.4%	40.5%
DM-02	Central California Valley	0.0770	Mar 1 2010	0.061	low	anoxic	50	76	52.3%	43.6%	4.1%
DM-03	Central California Valley	0.0770	Mar 2 2010	0.050	low	oxic	38	94	19.6%	0.0%	80.4%
DM-04	Central California Valley	0.0770	Mar 2 2010	0.120	high	anoxic	34	55	87.2%	1.8%	11.0%
DM-05	Central California Valley	0.0770	Mar 3 2010	0.026	low	anoxic	45	70	65.7%	5.6%	28.6%
DM-06	Central California Valley	0.0770	Mar 4 2010	0.026	low	oxic	27	182	51.6%	0.0%	48.4%
DM-07	Central California Valley	0.0770	Mar 8 2010	0.044	low	oxic	31	91	43.4%	54.8%	1.8%
DM-08	Central California Valley	0.0770	Mar 8 2010	0.055	low	oxic	42	79	28.3%	57.5%	14.2%

Status and trends of orthophosphate concentrations in groundwater used for public supply in California *Environmental Monitoring and Assessment, Robert Kent, Tyler D. Johnson, and Michael R. Rosen, U.S. Geological Survey California Water Science Center-rhkent@usgs.gov*

Online resource (suppplementary table) 2. Selected attributes of GAMA-PBP (https://ca.water.usgs.gov/gama/) status wells sampled for orthophosphate concentration-page 48.

GAMA-PBP ID	Agricultural land use in 1982[9] (percent)	Natural land use in 1982[9] (percent)	Urban land use in 1982[9] (percent)	Agricultural land use in 1992[9] (percent)	Natural land use in 1992[9] (percent)	Urban land use in 1992[9] (percent)	Agricultural land use in 2002[9] (percent)	Natural land use in 2002[9] (percent)	Urban land use in 2002[9] (percent)	Agricultural land use in 2012[9] (percent)	Natural land use in 2012[9] (percent)	Urban land use in 2012[9] (percent)	Age Classification[10]	Septic Tanks[11]	Aridity[12]
SAM-03[2]	39.7%	36.1%	24.2%	22.8%	36.1%	41.1%	23.7%	34.2%	42.0%	23.7%	33.8%	42.5%	ModernOrMixed	1.24	0.362
SAM-07[2]	0.0%	0.0%	100.0%	0.0%	0.0%	100.0%	0.0%	0.0%	100.0%	0.0%	0.0%	100.0%	ModernOrMixed	2.64	0.398
SAM-11[2]	2.8%	0.0%	97.2%	0.0%	0.0%	100.0%	0.0%	0.0%	100.0%	0.0%	0.0%	100.0%	PremodernOrMixed	9.70	0.384
SOL-01[2]	52.7%	0.5%	46.8%	50.9%	0.9%	48.2%	50.9%	0.9%	48.2%	50.9%	0.9%	48.2%	PremodernOrMixed	0.36	0.346
SOL-03[2]	62.9%	0.0%	37.1%	0.0%	0.0%	100.0%	0.0%	0.0%	100.0%	0.0%	0.0%	100.0%	PremodernOrMixed	0.19	0.473
SOL-06[2]	58.9%	10.0%	31.1%	58.9%	9.6%	31.5%	58.9%	9.6%	31.5%	58.9%	9.1%	32.0%	ModernOrMixed	4.99	0.346
SSV-QPC-02[2]	1.8%	0.0%	98.2%	0.0%	0.0%	100.0%	0.0%	0.0%	100.0%	0.0%	0.0%	100.0%	ModernOrMixed	8.09	0.496
SSV-QPC-05[2]	0.0%	0.0%	100.0%	0.0%	0.0%	100.0%	0.0%	0.0%	100.0%	0.0%	0.0%	100.0%	ModernOrMixed	4.65	0.410
SSV-QPC-06	1.4%	0.0%	98.6%	0.0%	0.0%	100.0%	0.0%	0.0%	100.0%	0.0%	0.0%	100.0%	Modern	0.00	0.453
SSV-QPC-07	0.9%	0.0%	99.1%	0.0%	0.0%	100.0%	0.0%	0.0%	100.0%	0.0%	0.0%	100.0%	Mixed	3.19	0.469
SSV-QPC-08[2]	12.8%	32.4%	54.8%	7.8%	37.4%	54.8%	7.8%	37.4%	54.8%	7.8%	37.4%	54.8%	ModernOrMixed	2.37	0.426
SSV-QPC-09[2]	0.0%	80.3%	19.7%	0.0%	80.3%	19.7%	0.0%	80.3%	19.7%	0.0%	60.6%	39.4%	PremodernOrMixed	0.38	0.433
SUI-01[2]	41.9%	55.8%	2.3%	41.9%	55.8%	2.3%	45.2%	52.5%	2.3%	45.2%	52.5%	2.3%	ModernOrMixed	0.50	0.381
SUI-02[2]	16.9%	56.8%	26.3%	7.0%	64.8%	28.2%	16.9%	53.5%	29.6%	16.9%	50.7%	32.4%	PremodernOrMixed	0.13	0.392
YOL-02[2]	68.6%	0.9%	30.5%	68.2%	0.9%	30.9%	67.7%	0.9%	31.4%	67.7%	0.9%	31.4%	ModernOrMixed	4.81	0.385
YOL-03	32.0%	4.1%	63.9%	31.5%	4.1%	64.4%	34.7%	3.7%	61.6%	26.5%	0.9%	72.6%	Premodern	0.54	0.381
YOL-04	42.7%	9.1%	48.2%	39.1%	9.5%	51.4%	39.5%	9.1%	51.4%	39.5%	6.8%	53.6%	Modern	3.54	0.459
YOL-06[2]	38.6%	15.9%	45.5%	33.6%	20.5%	45.9%	33.6%	20.5%	45.9%	26.4%	18.2%	55.5%	ModernOrMixed	4.47	0.414
YOL-08	5.5%	7.3%	87.2%	0.0%	7.3%	92.7%	0.0%	7.3%	92.7%	0.0%	6.8%	93.2%	Premodern	0.00	0.376
YOL-09[2]	67.9%	25.8%	6.3%	58.8%	34.4%	6.8%	54.3%	38.0%	7.7%	54.3%	36.7%	9.0%	ModernOrMixed	1.16	0.391
YOL-13[2]	98.1%	0.0%	1.9%	98.1%	0.0%	1.9%	98.1%	0.0%	1.9%	98.1%	0.0%	1.9%	ModernOrMixed	0.83	0.440
YOL-14	21.7%	10.1%	68.2%	12.4%	18.0%	69.6%	12.0%	16.1%	71.9%	9.7%	16.1%	74.2%	Premodern	40.34	0.368
DM-01	59.1%	0.9%	40.0%	59.1%	0.9%	40.0%	58.6%	0.5%	40.9%	58.6%	0.0%	41.4%	Premodern	0.59	0.156
DM-02	64.5%	31.4%	4.1%	46.4%	49.5%	4.1%	46.4%	48.2%	5.5%	34.1%	60.0%	5.9%	Modern	0.53	0.158
DM-03	16.9%	0.0%	83.1%	14.6%	0.0%	85.4%	13.7%	0.0%	86.3%	9.1%	0.0%	90.9%	Modern	3.43	0.189
DM-04	87.2%	1.8%	11.0%	87.2%	1.4%	11.4%	87.2%	1.4%	11.4%	87.2%	1.4%	11.4%	Modern	5.00	0.208
DM-05	69.0%	3.3%	27.7%	68.1%	3.8%	28.2%	67.1%	3.3%	29.6%	67.1%	3.3%	29.6%	Modern	0.36	0.164
DM-06	40.8%	0.0%	59.2%	35.2%	0.0%	64.8%	35.2%	0.0%	64.8%	35.2%	0.0%	64.8%	Mixed	13.23	0.231
DM-07	45.2%	53.0%	1.8%	45.7%	52.1%	2.3%	54.8%	42.5%	2.7%	54.8%	42.5%	2.7%	Mixed	1.41	0.200
DM-08	28.8%	57.1%	14.2%	28.8%	56.2%	15.1%	32.9%	51.1%	16.0%	32.9%	51.1%	16.0%	Modern	2.18	0.193

Online resource (suppplementary table) 2. Selected attributes of GAMA-PBP (https://ca.water.usgs.gov/gama/) status wells sampled for orthophosphate concentration-page 49.

GAMA-PBP ID	USGS Station ID[1]	GAMA-PBP study unit	GAMA-PBP study area[3]	Hydrogeologic zone
DM-09	371800121010001	Western San Joaquin Valley	Delta-Mendota subbasin	Central Valley
DM-10	371500120590001	Western San Joaquin Valley	Delta-Mendota subbasin	Central Valley
DM-11	373345121103201	Western San Joaquin Valley	Delta-Mendota subbasin	Central Valley
DM-12	373600121170001	Western San Joaquin Valley	Delta-Mendota subbasin	Central Valley
DM-13	365800120490001	Western San Joaquin Valley	Delta-Mendota subbasin	Central Valley
DM-14	372100120590001	Western San Joaquin Valley	Delta-Mendota subbasin	Central Valley
DM-15	365400120390001	Western San Joaquin Valley	Delta-Mendota subbasin	Central Valley
DM-16	370000120390001	Western San Joaquin Valley	Delta-Mendota subbasin	Central Valley
DM-17	364200120510001	Western San Joaquin Valley	Delta-Mendota subbasin	Central Valley
DM-18	365000120450001	Western San Joaquin Valley	Delta-Mendota subbasin	Central Valley
DM-19	365000120400001	Western San Joaquin Valley	Delta-Mendota subbasin	Central Valley
DM-20	372200121050001	Western San Joaquin Valley	Delta-Mendota subbasin	Central Valley
DM-21	373200121150001	Western San Joaquin Valley	Delta-Mendota subbasin	Central Valley
DM-22	365300120330001	Western San Joaquin Valley	Delta-Mendota subbasin	Central Valley
DM-23	370200120480001	Western San Joaquin Valley	Delta-Mendota subbasin	Central Valley
DM-24	365900120340001	Western San Joaquin Valley	Delta-Mendota subbasin	Central Valley
DM-25	370500120420001	Western San Joaquin Valley	Delta-Mendota subbasin	Central Valley
DM-26	371000121010001	Western San Joaquin Valley	Delta-Mendota subbasin	Central Valley
DM-27	370600120490001	Western San Joaquin Valley	Delta-Mendota subbasin	Central Valley
DM-28	365800120510001	Western San Joaquin Valley	Delta-Mendota subbasin	Central Valley
DM-29	370900120450001	Western San Joaquin Valley	Delta-Mendota subbasin	Central Valley
WS-01	360800120090001	Western San Joaquin Valley	Westside subbasin	Central Valley
WS-02	362100119550001	Western San Joaquin Valley	Westside subbasin	Central Valley
WS-03	360851120014602	Western San Joaquin Valley	Westside subbasin	Central Valley
WS-04	361706119562201	Western San Joaquin Valley	Westside subbasin	Central Valley
WS-05	362300120130001	Western San Joaquin Valley	Westside subbasin	Central Valley
WS-06[2]	361300119540001	Western San Joaquin Valley	Westside subbasin	Central Valley
WS-07	362600120120001	Western San Joaquin Valley	Westside subbasin	Central Valley
WS-08	360237120004201	Western San Joaquin Valley	Westside subbasin	Central Valley
WS-09	363100120050001	Western San Joaquin Valley	Westside subbasin	Central Valley

Online resource (suppplementary table) 2. Selected attributes of GAMA-PBP (https://ca.water.usgs.gov/gama/) status wells sampled for orthophosphate concentration-page 50.

GAMA-PBP ID	USEPA Level III Ecoregions[4]	Level III Ecoregion Reference Concentration[4]	Status Sample Date	Status Sample Orthophosphate Concentration (mg/L as P)	Relative Concentration Category[5]	Redox state[6]	Elevation of LSD (meters above NAVD 88)[7]	Well depth (meters below LSD)[8]	Agricultural land use in 1974[9] (percent)	Natural land use in 1974[9] (percent)	Urban land use in 1974[9] (percent)
DM-09	Central California Valley	0.0770	Mar 9 2010	0.030	low	oxic	27	192	0.0%	0.5%	99.5%
DM-10	Central California Valley	0.0770	Mar 9 2010	0.037	low	oxic	30	137	49.8%	1.4%	48.8%
DM-11	Central California Valley	0.0770	Mar 10 2010	0.041	low	oxic	16	51	53.7%	16.5%	29.8%
DM-12	Central California Valley	0.0770	Mar 11 2010	0.027	low	oxic	40	107	98.2%	0.9%	0.9%
DM-13	Central California Valley	0.0770	Mar 17 2010	0.055	low	anoxic	47	152	88.6%	10.0%	1.4%
DM-14	Central California Valley	0.0770	Mar 18 2010	0.042	low	anoxic	19	32	6.4%	92.7%	0.9%
DM-15	Central California Valley	0.0770	Apr 12 2010	0.050	low	anoxic	44	66	60.7%	32.2%	7.0%
DM-16	Central California Valley	0.0770	Apr 12 2010	0.107	high	anoxic	35	46	94.1%	3.7%	2.3%
DM-17	Central California Foothills and Coastal Mountains	0.0300	Apr 13 2010	0.050	moderate	anoxic	352	85	0.0%	99.5%	0.5%
DM-18	Central California Valley	0.0770	Apr 13 2010	0.009	low	anoxic	106	169	70.8%	26.0%	3.2%
DM-19	Central California Valley	0.0770	Apr 14 2010	0.022	low	anoxic	69	107	98.6%	0.5%	0.9%
DM-20	Central California Valley	0.0770	Apr 15 2010	0.027	low	oxic	43	64	99.5%	0.5%	0.0%
DM-21	Central California Foothills and Coastal Mountains	0.0300	Apr 15 2010	0.036	moderate	oxic	69	152	37.6%	59.6%	2.8%
DM-22	Central California Valley	0.0770	Jun 15 2010	0.088	moderate	anoxic	46	108	85.0%	14.1%	0.9%
DM-23	Central California Valley	0.0770	Jun 15 2010	0.050	low	oxic	34	61	96.8%	3.2%	0.0%
DM-24	Central California Valley	0.0770	Jun 16 2010	0.076	low	anoxic	37	46	99.5%	0.5%	0.0%
DM-25	Central California Valley	0.0770	Jun 16 2010	0.151	high	anoxic	31	46	96.4%	3.6%	0.0%
DM-26	Central California Valley	0.0770	Jun 17 2010	0.029	low	oxic	44	85	95.4%	0.5%	4.1%
DM-27	Central California Valley	0.0770	Jun 24 2010	0.051	low	anoxic	30	175	44.0%	53.7%	2.3%
DM-28	Central California Valley	0.0770	Jun 29 2010	0.031	low	oxic	60	54	97.7%	0.9%	1.4%
DM-29	Central California Valley	0.0770	Jun 30 2010	0.040	low	anoxic	29	70	92.7%	7.3%	0.0%
WS-01	Central California Valley	0.0770	Mar 15 2010	0.023	low	oxic	147	305	86.3%	1.4%	12.3%
WS-02	Central California Valley	0.0770	Jun 7 2010	0.149	high	anoxic	66	207	96.4%	3.2%	0.5%
WS-03	Central California Valley	0.0770	Jun 7 2010	0.022	low	anoxic	93	643	100.0%	0.0%	0.0%
WS-04	Central California Valley	0.0770	Jun 8 2010	0.017	low	anoxic	72	189	90.3%	5.1%	4.6%
WS-05	Central California Valley	0.0770	Jun 8 2010	0.017	low	anoxic	84	472	95.9%	4.1%	0.0%
WS-06[2]	Central California Valley	0.0770	Jun 9 2010	0.011	low	anoxic	70	na	99.1%	0.5%	0.5%
WS-07	Central California Valley	0.0770	Jun 10 2010	0.028	low	anoxic	73	311	99.1%	0.9%	0.0%
WS-08	Central California Foothills and Coastal Mountains	0.0300	Jun 22 2010	0.022	low	oxic	113	457	94.1%	0.0%	5.9%
WS-09	Central California Valley	0.0770	Jun 23 2010	0.170	high	anoxic	56	186	70.3%	5.0%	24.7%

Online resource (suppplementary table) 2. Selected attributes of GAMA-PBP (https://ca.water.usgs.gov/gama/) status wells sampled for orthophosphate concentration-page 51.

GAMA-PBP ID	Agricultural land use in 1982[9] (percent)	Natural land use in 1982[9] (percent)	Urban land use in 1982[9] (percent)	Agricultural land use in 1992[9] (percent)	Natural land use in 1992[9] (percent)	Urban land use in 1992[9] (percent)	Agricultural land use in 2002[9] (percent)	Natural land use in 2002[9] (percent)	Urban land use in 2002[9] (percent)	Agricultural land use in 2012[9] (percent)	Natural land use in 2012[9] (percent)	Urban land use in 2012[9] (percent)	Age Classification[10]	Septic Tanks[11]	Aridity[12]
DM-09	0.0%	0.5%	99.5%	0.0%	0.5%	99.5%	0.0%	0.5%	99.5%	0.0%	0.0%	100.0%	Mixed	13.01	0.221
DM-10	44.2%	1.4%	54.4%	44.2%	0.5%	55.3%	42.3%	0.5%	57.2%	42.3%	0.0%	57.7%	Mixed	6.78	0.218
DM-11	54.1%	16.1%	29.8%	54.1%	16.1%	29.8%	56.9%	14.2%	28.9%	56.9%	14.2%	28.9%	Mixed	2.18	0.231
DM-12	98.2%	0.9%	0.9%	98.2%	0.9%	0.9%	98.2%	0.5%	1.4%	98.2%	0.5%	1.4%	Mixed	1.79	0.208
DM-13	88.6%	10.0%	1.4%	88.6%	10.0%	1.4%	88.1%	10.0%	1.8%	88.1%	10.0%	1.8%	Premodern	0.77	0.182
DM-14	13.3%	85.8%	0.9%	2.8%	96.3%	0.9%	7.3%	91.3%	1.4%	7.3%	91.3%	1.4%	Modern	3.15	0.226
DM-15	77.1%	15.0%	7.9%	49.1%	41.6%	9.3%	49.5%	40.7%	9.8%	49.5%	40.7%	9.8%	Premodern	0.34	0.182
DM-16	94.1%	3.7%	2.3%	93.2%	3.2%	3.7%	92.7%	2.7%	4.6%	92.7%	2.7%	4.6%	Premodern	3.14	0.200
DM-17	0.0%	99.5%	0.5%	0.0%	99.5%	0.5%	0.0%	99.5%	0.5%	0.0%	99.1%	0.9%	Mixed	0.30	0.175
DM-18	79.0%	16.9%	4.1%	66.7%	28.8%	4.6%	64.8%	30.6%	4.6%	62.6%	32.9%	4.6%	Premodern	0.34	0.160
DM-19	98.6%	0.5%	0.9%	98.6%	0.0%	1.4%	98.6%	0.0%	1.4%	98.6%	0.0%	1.4%	Premodern	0.34	0.167
DM-20	99.5%	0.5%	0.0%	99.5%	0.5%	0.0%	99.5%	0.5%	0.0%	96.8%	3.2%	0.0%	Mixed	0.97	0.222
DM-21	38.1%	59.2%	2.8%	38.1%	59.2%	2.8%	38.1%	58.3%	3.7%	38.1%	58.3%	3.7%	Modern	1.79	0.204
DM-22	85.0%	14.1%	0.9%	85.0%	14.1%	0.9%	84.5%	14.1%	1.4%	84.5%	14.1%	1.4%	Premodern	1.04	0.179
DM-23	96.8%	3.2%	0.0%	96.8%	3.2%	0.0%	96.8%	3.2%	0.0%	96.8%	3.2%	0.0%	Premodern	0.77	0.189
DM-24	99.5%	0.5%	0.0%	99.5%	0.5%	0.0%	99.5%	0.5%	0.0%	99.5%	0.0%	0.5%	Modern	1.88	0.196
DM-25	96.8%	3.2%	0.0%	96.8%	3.2%	0.0%	96.8%	3.2%	0.0%	75.1%	24.9%	0.0%	Mixed	1.02	0.206
DM-26	95.4%	0.5%	4.1%	95.4%	0.5%	4.1%	95.0%	0.5%	4.6%	95.0%	0.0%	5.0%	Modern	1.53	0.204
DM-27	49.1%	48.6%	2.3%	54.1%	41.3%	4.6%	59.2%	36.2%	4.6%	59.2%	36.2%	4.6%	Premodern	0.81	0.193
DM-28	97.7%	0.9%	1.4%	97.7%	0.9%	1.4%	97.7%	0.5%	1.9%	97.7%	0.5%	1.9%	ModernOrMixed	0.20	0.175
DM-29	94.1%	5.9%	0.0%	99.1%	0.9%	0.0%	99.1%	0.9%	0.0%	90.4%	9.6%	0.0%	Modern	1.01	0.214
WS-01	86.3%	0.9%	12.8%	85.8%	0.9%	13.2%	85.8%	0.9%	13.2%	85.8%	0.0%	14.2%	Premodern	0.20	0.129
WS-02	96.8%	2.7%	0.5%	96.8%	2.7%	0.5%	96.8%	2.7%	0.5%	96.8%	2.7%	0.5%	Mixed	0.56	0.141
WS-03	100.0%	0.0%	0.0%	100.0%	0.0%	0.0%	100.0%	0.0%	0.0%	100.0%	0.0%	0.0%	Premodern	0.20	0.132
WS-04	95.4%	0.0%	4.6%	95.4%	0.0%	4.6%	95.4%	0.0%	4.6%	95.4%	0.0%	4.6%	Premodern	0.56	0.140
WS-05	95.9%	4.1%	0.0%	95.9%	4.1%	0.0%	100.0%	0.0%	0.0%	100.0%	0.0%	0.0%	Premodern	0.44	0.137
WS-06[2]	99.1%	0.5%	0.5%	97.3%	0.5%	2.3%	97.3%	0.0%	2.7%	97.3%	0.0%	2.7%	Premodern	0.07	0.139
WS-07	99.1%	0.9%	0.0%	98.1%	0.9%	0.9%	98.1%	0.9%	0.9%	98.1%	0.9%	0.9%	Premodern	0.31	0.137
WS-08	94.1%	0.0%	5.9%	94.1%	0.0%	5.9%	94.1%	0.0%	5.9%	94.1%	0.0%	5.9%	Premodern	0.38	0.116
WS-09	71.2%	3.7%	25.1%	71.2%	3.7%	25.1%	71.2%	3.7%	25.1%	71.2%	2.3%	26.5%	Premodern	0.73	0.145

Online resource (suppplementary table) 2. Selected attributes of GAMA-PBP (https://ca.water.usgs.gov/gama/) status wells sampled for orthophosphate concentration-page 52.

GAMA-PBP ID	USGS Station ID[1]	GAMA-PBP study unit	GAMA-PBP study area[3]	Hydrogeologic zone
WS-10	361900119560001	Western San Joaquin Valley	Westside subbasin	Central Valley
ANT-02	343331118014901	Antelope Valley	Antelope Valley	Desert
ANT-04	344834118151401	Antelope Valley	Antelope Valley	Desert
ANT-07	345006118125701	Antelope Valley	Antelope Valley	Desert
ANT-09	344000118250001	Antelope Valley	Antelope Valley	Desert
ANT-12	343953118041901	Antelope Valley	Antelope Valley	Desert
ANT-15	344700118360001	Antelope Valley	Antelope Valley	Desert
ANT-17	344120118081301	Antelope Valley	Antelope Valley	Desert
ANT-26	343222117552901	Antelope Valley	Antelope Valley	Desert
ANT-34	350038117420501	Antelope Valley	Antelope Valley	Desert
ANT-40	344849117545801	Antelope Valley	Antelope Valley	Desert
ANT-43	343144117572901	Antelope Valley	Antelope Valley	Desert
ANT-45[2]	350000117500001	Antelope Valley	Antelope Valley	Desert
ANT-46	343932118144001	Antelope Valley	Antelope Valley	Desert
ANT-47	344415118130301	Antelope Valley	Antelope Valley	Desert
ANT-48[2]	345100118120001	Antelope Valley	Antelope Valley	Desert
ANT-49	343717118063601	Antelope Valley	Antelope Valley	Desert
ANT-51	350100117500001	Antelope Valley	Antelope Valley	Desert
ANT-52	343713117485101	Antelope Valley	Antelope Valley	Desert
BV-03	332057116240101	Borrego Valley, Central Desert, and Low-Use Basins of the Mojave and Sonoran Deserts	Borrego Valley	Desert
BV-04	331305116233201	Borrego Valley, Central Desert, and Low-Use Basins of the Mojave and Sonoran Deserts	Borrego Valley	Desert
BV-07	331100116170002	Borrego Valley, Central Desert, and Low-Use Basins of the Mojave and Sonoran Deserts	Borrego Valley	Desert
CD-01	341643116243101	Borrego Valley, Central Desert, and Low-Use Basins of the Mojave and Sonoran Deserts	Central Desert basins	Desert
CD-03[2]	340900116290001	Borrego Valley, Central Desert, and Low-Use Basins of the Mojave and Sonoran Deserts	Central Desert basins	Desert
CD-04	340804116083001	Borrego Valley, Central Desert, and Low-Use Basins of the Mojave and Sonoran Deserts	Central Desert basins	Desert
CD-07	340831116172201	Borrego Valley, Central Desert, and Low-Use Basins of the Mojave and Sonoran Deserts	Central Desert basins	Desert
CD-09	340738116244301	Borrego Valley, Central Desert, and Low-Use Basins of the Mojave and Sonoran Deserts	Central Desert basins	Desert
CD-11[2]	340400116330001	Borrego Valley, Central Desert, and Low-Use Basins of the Mojave and Sonoran Deserts	Central Desert basins	Desert
CD-12	340700116170001	Borrego Valley, Central Desert, and Low-Use Basins of the Mojave and Sonoran Deserts	Central Desert basins	Desert
CD-13	340718116010301	Borrego Valley, Central Desert, and Low-Use Basins of the Mojave and Sonoran Deserts	Central Desert basins	Desert

Online resource (suppplementary table) 2. Selected attributes of GAMA-PBP (https://ca.water.usgs.gov/gama/) status wells sampled for orthophosphate concentration-page 53.

GAMA-PBP ID	USEPA Level III Ecoregions[4]	Level III Ecoregion Reference Concentration[4]	Status Sample Date	Status Sample Orthophosphate Concentration (mg/L as P)	Relative Concentration Category[5]	Redox state[6]	Elevation of LSD (meters above NAVD 88)[7]	Well depth (meters below LSD)[8]	Agricultural land use in 1974[9] (percent)	Natural land use in 1974[9] (percent)	Urban land use in 1974[9] (percent)
WS-10	Central California Valley	0.0770	Jul 8 2010	0.035	low	anoxic	71	183	54.9%	21.6%	23.5%
ANT-02	Mojave Basin and Range	0.0100	Jan 28 2008	0.036	moderate	oxic	833	122	0.0%	0.0%	100.0%
ANT-04	Mojave Basin and Range	0.0100	Jan 29 2008	0.013	moderate	oxic	731	233	5.2%	90.6%	4.2%
ANT-07	Mojave Basin and Range	0.0100	Jan 29 2008	0.014	moderate	oxic	719	161	0.0%	98.2%	1.8%
ANT-09	Mojave Basin and Range	0.0100	Jan 30 2008	0.074	moderate	oxic	1,004	65	18.8%	77.5%	3.7%
ANT-12	Mojave Basin and Range	0.0100	Jan 30 2008	0.011	moderate	oxic	747	366	70.8%	17.4%	11.9%
ANT-15	Mojave Basin and Range	0.0100	Jan 31 2008	0.032	moderate	oxic	908	207	34.1%	65.5%	0.5%
ANT-17	Mojave Basin and Range	0.0100	Jan 31 2008	0.026	moderate	oxic	734	168	0.0%	0.0%	100.0%
ANT-26	Mojave Basin and Range	0.0100	Feb 7 2008	0.015	moderate	oxic	860	98	0.0%	97.2%	2.8%
ANT-34	Mojave Basin and Range	0.0100	Feb 14 2008	0.022	moderate	oxic	727	185	0.0%	84.5%	15.5%
ANT-40	Mojave Basin and Range	0.0100	Feb 28 2008	0.024	moderate	oxic	701	257	0.0%	100.0%	0.0%
ANT-43	Mojave Basin and Range	0.0100	Mar 4 2008	0.024	moderate	oxic	903	168	46.4%	18.6%	35.0%
ANT-45[2]	Mojave Basin and Range	0.0100	Mar 5 2008	0.040	moderate	oxic	698	na	0.0%	5.9%	94.1%
ANT-46	Mojave Basin and Range	0.0100	Mar 6 2008	0.017	moderate	oxic	758	145	72.6%	0.9%	26.5%
ANT-47	Mojave Basin and Range	0.0100	Mar 17 2008	0.015	moderate	oxic	714	335	0.0%	66.7%	33.3%
ANT-48[2]	Mojave Basin and Range	0.0100	Mar 18 2008	0.012	moderate	oxic	730	na	3.2%	55.0%	41.7%
ANT-49	Mojave Basin and Range	0.0100	Mar 19 2008	0.018	moderate	oxic	781	244	0.0%	65.7%	34.3%
ANT-51	Mojave Basin and Range	0.0100	Mar 20 2008	0.206	high	oxic	699	100	0.0%	33.0%	67.0%
ANT-52	Mojave Basin and Range	0.0100	Apr 7 2008	0.014	moderate	anoxic	810	91	0.0%	3.7%	96.3%
BV-03	Sonoran Basin and Range	0.0250	Oct 20 2009	0.019	low	oxic	283	107	0.0%	100.0%	0.0%
BV-04	Sonoran Basin and Range	0.0250	Oct 26 2009	0.033	moderate	oxic	253	192	0.0%	99.1%	0.9%
BV-07	Sonoran Basin and Range	0.0250	Dec 3 2009	0.013	low	oxic	170	115	0.0%	100.0%	0.0%
CD-01	Mojave Basin and Range	0.0100	Dec 15 2008	0.016	moderate	oxic	933	117	0.0%	88.1%	11.9%
CD-03[2]	Mojave Basin and Range	0.0100	Dec 16 2008	0.025	moderate	oxic	1,211	na	0.0%	97.2%	2.8%
CD-04	Mojave Basin and Range	0.0100	Dec 17 2008	0.018	moderate	oxic	766	107	0.0%	63.3%	36.7%
CD-07	Mojave Basin and Range	0.0100	Oct 5 2009	0.027	moderate	oxic	801	226	0.0%	98.6%	1.4%
CD-09	Mojave Basin and Range	0.0100	Oct 6 2009	0.027	moderate	oxic	985	340	0.0%	19.4%	80.6%
CD-11[2]	Mojave Basin and Range	0.0100	Oct 7 2009	0.027	moderate	oxic	871	na	0.0%	27.7%	72.3%
CD-12	Mojave Basin and Range	0.0100	Oct 7 2009	0.014	moderate	oxic	851	168	0.0%	92.7%	7.3%
CD-13	Mojave Basin and Range	0.0100	Nov 16 2009	0.009	low	oxic	635	79	0.0%	87.5%	12.5%

Online resource (suppplementary table) 2. Selected attributes of GAMA-PBP (https://ca.water.usgs.gov/gama/) status wells sampled for orthophosphate concentration-page 54.

GAMA-PBP ID	Agricultural land use in 1982[9] (percent)	Natural land use in 1982[9] (percent)	Urban land use in 1982[9] (percent)	Agricultural land use in 1992[9] (percent)	Natural land use in 1992[9] (percent)	Urban land use in 1992[9] (percent)	Agricultural land use in 2002[9] (percent)	Natural land use in 2002[9] (percent)	Urban land use in 2002[9] (percent)	Agricultural land use in 2012[9] (percent)	Natural land use in 2012[9] (percent)	Urban land use in 2012[9] (percent)	Age Classification[10]	Septic Tanks[11]	Aridity[12]
WS-10	54.9%	21.6%	23.5%	54.9%	21.6%	23.5%	54.9%	21.6%	23.5%	54.9%	21.1%	23.9%	Premodern	0.56	0.140
ANT-02	0.0%	0.0%	100.0%	0.0%	0.0%	100.0%	0.0%	0.0%	100.0%	0.0%	0.0%	100.0%	Modern	2.23	0.138
ANT-04	5.2%	90.6%	4.2%	2.3%	93.4%	4.2%	2.3%	93.4%	4.2%	2.3%	93.4%	4.2%	Premodern	4.39	0.131
ANT-07	0.0%	98.2%	1.8%	0.0%	98.2%	1.8%	0.0%	98.2%	1.8%	0.0%	5.5%	94.5%	Premodern	2.64	0.123
ANT-09	18.8%	77.5%	3.7%	17.4%	16.1%	66.5%	17.0%	16.1%	67.0%	17.0%	16.1%	67.0%	Modern	2.48	0.379
ANT-12	66.7%	16.4%	16.9%	63.5%	16.9%	19.6%	61.6%	16.4%	21.9%	37.0%	7.3%	55.7%	Premodern	4.24	0.113
ANT-15	34.1%	65.5%	0.5%	28.2%	71.4%	0.5%	28.2%	71.4%	0.5%	66.8%	32.7%	0.5%	Mixed	0.59	0.194
ANT-17	0.0%	0.0%	100.0%	0.0%	0.0%	100.0%	0.0%	0.0%	100.0%	0.0%	0.0%	100.0%	Premodern	2.99	0.110
ANT-26	0.0%	97.2%	2.8%	0.0%	97.2%	2.8%	0.0%	96.3%	3.7%	0.0%	95.8%	4.2%	Premodern	7.24	0.118
ANT-34	0.0%	84.5%	15.5%	0.0%	84.5%	15.5%	0.0%	84.5%	15.5%	0.0%	84.5%	15.5%	PremodernOrMixed	0.94	0.088
ANT-40	0.0%	100.0%	0.0%	0.0%	100.0%	0.0%	0.0%	100.0%	0.0%	0.0%	99.5%	0.5%	Premodern	0.00	0.095
ANT-43	45.9%	18.6%	35.5%	45.5%	13.6%	40.9%	45.5%	13.6%	40.9%	45.5%	3.6%	50.9%	Premodern	73.61	0.124
ANT-45[2]	0.0%	5.9%	94.1%	0.0%	5.9%	94.1%	0.0%	5.9%	94.1%	0.0%	5.9%	94.1%	Premodern	14.20	0.088
ANT-46	72.6%	0.9%	26.5%	47.0%	0.0%	53.0%	43.4%	0.0%	56.6%	42.0%	0.0%	58.0%	Mixed	3.12	0.149
ANT-47	0.0%	66.2%	33.8%	0.0%	62.5%	37.5%	0.0%	62.5%	37.5%	0.0%	58.3%	41.7%	Premodern	1.10	0.130
ANT-48[2]	3.2%	55.0%	41.7%	0.9%	55.0%	44.0%	0.5%	54.6%	45.0%	12.8%	1.4%	85.8%	Mixed	2.64	0.119
ANT-49	0.0%	65.7%	34.3%	0.0%	65.7%	34.3%	0.0%	65.7%	34.3%	0.0%	65.7%	34.3%	Premodern	1.28	0.122
ANT-51	0.0%	33.0%	67.0%	0.0%	33.0%	67.0%	0.0%	33.0%	67.0%	0.0%	33.0%	67.0%	Premodern	16.42	0.088
ANT-52	0.0%	3.7%	96.3%	0.0%	3.2%	96.8%	0.0%	3.2%	96.8%	0.0%	3.2%	96.8%	Premodern	63.40	0.110
BV-03	0.0%	100.0%	0.0%	0.0%	100.0%	0.0%	0.0%	100.0%	0.0%	0.0%	100.0%	0.0%	Modern	0.47	0.107
BV-04	0.0%	99.1%	0.9%	0.0%	99.1%	0.9%	0.0%	0.5%	99.5%	0.0%	0.5%	99.5%	Premodern	2.58	0.092
BV-07	0.0%	100.0%	0.0%	0.0%	100.0%	0.0%	0.0%	100.0%	0.0%	0.0%	99.5%	0.5%	PremodernOrMixed	1.97	0.084
CD-01	0.0%	88.1%	11.9%	0.0%	23.3%	76.7%	0.0%	23.3%	76.7%	0.0%	23.3%	76.7%	Premodern	2.53	0.106
CD-03[2]	0.0%	97.2%	2.8%	0.0%	97.2%	2.8%	0.0%	97.2%	2.8%	0.0%	97.2%	2.8%	Mixed	1.45	0.175
CD-04	0.0%	63.3%	36.7%	0.0%	63.3%	36.7%	0.0%	62.3%	37.7%	0.0%	62.3%	37.7%	Mixed	11.34	0.102
CD-07	0.0%	98.6%	1.4%	0.0%	89.0%	11.0%	0.0%	89.0%	11.0%	0.0%	89.0%	11.0%	Premodern	9.36	0.129
CD-09	0.0%	13.4%	86.6%	0.0%	3.7%	96.3%	0.0%	3.7%	96.3%	0.0%	1.4%	98.6%	Modern	71.12	0.156
CD-11[2]	0.0%	27.7%	72.3%	0.0%	27.7%	72.3%	0.0%	27.7%	72.3%	0.0%	0.0%	100.0%	Modern	21.16	0.191
CD-12	0.0%	92.7%	7.3%	0.0%	92.7%	7.3%	0.0%	92.7%	7.3%	0.0%	92.7%	7.3%	Premodern	10.40	0.143
CD-13	0.0%	87.5%	12.5%	0.0%	87.5%	12.5%	0.0%	87.5%	12.5%	0.0%	87.0%	13.0%	Premodern	9.81	0.074

Online resource (suppplementary table) 2. Selected attributes of GAMA-PBP (https://ca.water.usgs.gov/gama/) status wells sampled for orthophosphate concentration-page 55.

GAMA-PBP ID	USGS Station ID[1]	GAMA-PBP study unit	GAMA-PBP study area[3]	Hydrogeologic zone
CD-14	341836116150501	Borrego Valley, Central Desert, and Low-Use Basins of the Mojave and Sonoran Deserts	Central Desert basins	Desert
LUB-01	350206118082401	Borrego Valley, Central Desert, and Low-Use Basins of the Mojave and Sonoran Deserts	Low-use basins of the Mojave and Sonoran Deserts	Desert
LUB-02	350701117590401	Borrego Valley, Central Desert, and Low-Use Basins of the Mojave and Sonoran Deserts	Low-use basins of the Mojave and Sonoran Deserts	Desert
LUB-03	351659117591901	Borrego Valley, Central Desert, and Low-Use Basins of the Mojave and Sonoran Deserts	Low-use basins of the Mojave and Sonoran Deserts	Desert
LUB-04[2]	350700118000001	Borrego Valley, Central Desert, and Low-Use Basins of the Mojave and Sonoran Deserts	Low-use basins of the Mojave and Sonoran Deserts	Desert
LUB-05	342400116540001	Borrego Valley, Central Desert, and Low-Use Basins of the Mojave and Sonoran Deserts	Low-use basins of the Mojave and Sonoran Deserts	Desert
LUB-06	351556116404601	Borrego Valley, Central Desert, and Low-Use Basins of the Mojave and Sonoran Deserts	Low-use basins of the Mojave and Sonoran Deserts	Desert
LUB-07	351125116374901	Borrego Valley, Central Desert, and Low-Use Basins of the Mojave and Sonoran Deserts	Low-use basins of the Mojave and Sonoran Deserts	Desert
LUB-08	351811116361801	Borrego Valley, Central Desert, and Low-Use Basins of the Mojave and Sonoran Deserts	Low-use basins of the Mojave and Sonoran Deserts	Desert
LUB-09	351600116030001	Borrego Valley, Central Desert, and Low-Use Basins of the Mojave and Sonoran Deserts	Low-use basins of the Mojave and Sonoran Deserts	Desert
LUB-10	345703116350801	Borrego Valley, Central Desert, and Low-Use Basins of the Mojave and Sonoran Deserts	Low-use basins of the Mojave and Sonoran Deserts	Desert
LUB-11	352350117451601	Borrego Valley, Central Desert, and Low-Use Basins of the Mojave and Sonoran Deserts	Low-use basins of the Mojave and Sonoran Deserts	Desert
LUB-12	342000116340001	Borrego Valley, Central Desert, and Low-Use Basins of the Mojave and Sonoran Deserts	Low-use basins of the Mojave and Sonoran Deserts	Desert
LUB-13	342637116562001	Borrego Valley, Central Desert, and Low-Use Basins of the Mojave and Sonoran Deserts	Low-use basins of the Mojave and Sonoran Deserts	Desert
LUB-14	330300116250001	Borrego Valley, Central Desert, and Low-Use Basins of the Mojave and Sonoran Deserts	Low-use basins of the Mojave and Sonoran Deserts	Desert
LUB-15	324416115594102	Borrego Valley, Central Desert, and Low-Use Basins of the Mojave and Sonoran Deserts	Low-use basins of the Mojave and Sonoran Deserts	Desert
LUB-16	324222115130201	Borrego Valley, Central Desert, and Low-Use Basins of the Mojave and Sonoran Deserts	Low-use basins of the Mojave and Sonoran Deserts	Desert
LUB-17	325200114510001	Borrego Valley, Central Desert, and Low-Use Basins of the Mojave and Sonoran Deserts	Low-use basins of the Mojave and Sonoran Deserts	Desert
LUB-18	323600116110001	Borrego Valley, Central Desert, and Low-Use Basins of the Mojave and Sonoran Deserts	Low-use basins of the Mojave and Sonoran Deserts	Desert
LUB-19[2]	330900116060001	Borrego Valley, Central Desert, and Low-Use Basins of the Mojave and Sonoran Deserts	Low-use basins of the Mojave and Sonoran Deserts	Desert
LUB-20	324353115574301	Borrego Valley, Central Desert, and Low-Use Basins of the Mojave and Sonoran Deserts	Low-use basins of the Mojave and Sonoran Deserts	Desert
LUB-21	334400115210001	Borrego Valley, Central Desert, and Low-Use Basins of the Mojave and Sonoran Deserts	Low-use basins of the Mojave and Sonoran Deserts	Desert
LUB-22	333333114544101	Borrego Valley, Central Desert, and Low-Use Basins of the Mojave and Sonoran Deserts	Low-use basins of the Mojave and Sonoran Deserts	Desert
LUB-23	334712115485601	Borrego Valley, Central Desert, and Low-Use Basins of the Mojave and Sonoran Deserts	Low-use basins of the Mojave and Sonoran Deserts	Desert
LUB-24	352700115390001	Borrego Valley, Central Desert, and Low-Use Basins of the Mojave and Sonoran Deserts	Low-use basins of the Mojave and Sonoran Deserts	Desert
LUB-25	352800115160001	Borrego Valley, Central Desert, and Low-Use Basins of the Mojave and Sonoran Deserts	Low-use basins of the Mojave and Sonoran Deserts	Desert
LUB-26	350400115220001	Borrego Valley, Central Desert, and Low-Use Basins of the Mojave and Sonoran Deserts	Low-use basins of the Mojave and Sonoran Deserts	Desert
LUB-27	343300115320001	Borrego Valley, Central Desert, and Low-Use Basins of the Mojave and Sonoran Deserts	Low-use basins of the Mojave and Sonoran Deserts	Desert
COA-01	335532116471701	Coachella Valley	Coachella Valley	Desert
COA-02[2]	332700116030001	Coachella Valley	Coachella Valley	Desert

Online resource (suppplementary table) 2. Selected attributes of GAMA-PBP (https://ca.water.usgs.gov/gama/) status wells sampled for orthophosphate concentration-page 56.

GAMA-PBP ID	USEPA Level III Ecoregions[4]	Level III Ecoregion Reference Concentration[4]	Status Sample Date	Status Sample Orthophosphate Concentration (mg/L as P)	Relative Concentration Category[5]	Redox state[6]	Elevation of LSD (meters above NAVD 88)[7]	Well depth (meters below LSD)[8]	Agricultural land use in 1974[9] (percent)	Natural land use in 1974[9] (percent)	Urban land use in 1974[9] (percent)
CD-14	Mojave Basin and Range	0.0100	Nov 17 2009	0.009	low	oxic	769	183	0.0%	100.0%	0.0%
LUB-01	Mojave Basin and Range	0.0100	Dec 2 2008	0.011	moderate	oxic	812	116	0.0%	98.2%	1.8%
LUB-02	Mojave Basin and Range	0.0100	Dec 2 2008	0.022	moderate	oxic	739	166	0.0%	32.3%	67.7%
LUB-03	Mojave Basin and Range	0.0100	Dec 3 2008	0.051	moderate	anoxic	611	139	0.0%	82.6%	17.4%
LUB-04[2]	Mojave Basin and Range	0.0100	Dec 3 2008	0.017	moderate	oxic	745	na	0.5%	45.5%	54.1%
LUB-05	Mojave Basin and Range	0.0100	Dec 4 2008	0.012	moderate	oxic	983	149	0.0%	94.0%	6.0%
LUB-06	Mojave Basin and Range	0.0100	Dec 8 2008	0.015	moderate	oxic	751	149	0.0%	37.9%	62.1%
LUB-07	Mojave Basin and Range	0.0100	Dec 9 2008	0.011	moderate	oxic	665	201	0.0%	100.0%	0.0%
LUB-08	Mojave Basin and Range	0.0100	Dec 9 2008	0.029	moderate	oxic	725	244	0.0%	100.0%	0.0%
LUB-09	Mojave Basin and Range	0.0100	Dec 10 2008	0.018	moderate	oxic	317	68	0.0%	65.3%	34.7%
LUB-10	Mojave Basin and Range	0.0100	Dec 10 2008	0.096	moderate	anoxic	529	91	0.0%	100.0%	0.0%
LUB-11	Mojave Basin and Range	0.0100	Dec 11 2008	0.017	moderate	oxic	700	183	0.0%	100.0%	0.0%
LUB-12	Mojave Basin and Range	0.0100	Dec 16 2008	1.351	high	oxic	999	256	0.0%	97.3%	2.7%
LUB-13	Mojave Basin and Range	0.0100	Oct 8 2009	0.014	moderate	oxic	901	152	0.0%	90.4%	9.6%
LUB-14	Sonoran Basin and Range	0.0250	Oct 21 2009	0.024	low	oxic	724	73	0.0%	97.7%	2.3%
LUB-15	Sonoran Basin and Range	0.0250	Oct 26 2009	0.013	low	oxic	118	95	0.0%	51.6%	48.4%
LUB-16	Sonoran Basin and Range	0.0250	Oct 28 2009	0.009	low	oxic	27	32	0.0%	100.0%	0.0%
LUB-17	Sonoran Basin and Range	0.0250	Oct 28 2009	0.008	low	oxic	151	178	0.0%	100.0%	0.0%
LUB-18	Central California Foothills and Coastal Mountains	0.0300	Nov 2 2009	0.160	high	oxic	869	11	0.0%	76.5%	23.5%
LUB-19[2]	Sonoran Basin and Range	0.0250	Nov 3 2009	0.007	low	anoxic	54	na	0.0%	100.0%	0.0%
LUB-20	Sonoran Basin and Range	0.0250	Nov 4 2009	0.018	low	oxic	86	30	0.5%	88.1%	11.5%
LUB-21	Sonoran Basin and Range	0.0250	Nov 18 2009	0.008	low	oxic	191	122	1.4%	89.5%	9.1%
LUB-22	Sonoran Basin and Range	0.0250	Nov 19 2009	0.010	low	anoxic	140	366	0.0%	99.1%	0.9%
LUB-23	Mojave Basin and Range	0.0100	Dec 7 2009	0.014	moderate	oxic	930	123	0.0%	100.0%	0.0%
LUB-24	Mojave Basin and Range	0.0100	Dec 8 2009	0.011	moderate	oxic	358	240	0.0%	87.2%	12.8%
LUB-25	Mojave Basin and Range	0.0100	Dec 8 2009	0.008	low	oxic	932	264	0.0%	94.5%	5.5%
LUB-26	Mojave Basin and Range	0.0100	Dec 9 2009	0.010	moderate	oxic	1,410	217	0.0%	100.0%	0.0%
LUB-27	Mojave Basin and Range	0.0100	Mar 4 2010	0.021	moderate	oxic	74	122	0.0%	96.8%	3.2%
COA-01	Southern California Mountains	0.0109	Feb 27 2007	0.019	moderate	oxic	607	366	0.5%	97.3%	2.3%
COA-02[2]	Sonoran Basin and Range	0.0250	Feb 28 2007	0.007	low	anoxic	-64	na	31.5%	65.3%	3.2%

Status and trends of orthophosphate concentrations in groundwater used for public supply in California *Environmental Monitoring and Assessment, Robert Kent, Tyler D. Johnson, and Michael R. Rosen, U.S. Geological Survey California Water Science Center-rhkent@usgs.gov*

Online resource (suppplementary table) 2. Selected attributes of GAMA-PBP (https://ca.water.usgs.gov/gama/) status wells sampled for orthophosphate concentration-page 57.

GAMA-PBP ID	Agricultural land use in 1982[9] (percent)	Natural land use in 1982[9] (percent)	Urban land use in 1982[9] (percent)	Agricultural land use in 1992[9] (percent)	Natural land use in 1992[9] (percent)	Urban land use in 1992[9] (percent)	Agricultural land use in 2002[9] (percent)	Natural land use in 2002[9] (percent)	Urban land use in 2002[9] (percent)	Agricultural land use in 2012[9] (percent)	Natural land use in 2012[9] (percent)	Urban land use in 2012[9] (percent)	Age Classification[10]	Septic Tanks[11]	Aridity[12]
CD-14	0.0%	100.0%	0.0%	0.0%	100.0%	0.0%	0.0%	100.0%	0.0%	0.0%	100.0%	0.0%	Premodern	0.05	0.083
LUB-01	0.0%	98.2%	1.8%	0.0%	98.2%	1.8%	0.0%	98.2%	1.8%	0.0%	98.2%	1.8%	Mixed	7.39	0.104
LUB-02	0.0%	32.3%	67.7%	0.0%	29.1%	70.9%	0.0%	29.1%	70.9%	0.0%	0.0%	100.0%	Premodern	59.15	0.096
LUB-03	0.0%	82.6%	17.4%	0.0%	82.6%	17.4%	0.0%	82.6%	17.4%	0.0%	82.6%	17.4%	Mixed	0.41	0.088
LUB-04[2]	0.0%	44.5%	55.5%	0.0%	44.5%	55.5%	0.0%	21.8%	78.2%	0.0%	21.4%	78.6%	Premodern	2.39	0.097
LUB-05	0.0%	94.0%	6.0%	0.0%	93.5%	6.5%	0.0%	93.5%	6.5%	0.0%	93.5%	6.5%	Premodern	7.55	0.120
LUB-06	0.0%	37.9%	62.1%	0.0%	37.9%	62.1%	0.0%	28.3%	71.7%	0.0%	12.8%	87.2%	Premodern	0.06	0.079
LUB-07	0.0%	100.0%	0.0%	0.0%	100.0%	0.0%	0.0%	100.0%	0.0%	0.0%	100.0%	0.0%	Premodern	0.06	0.075
LUB-08	0.0%	100.0%	0.0%	0.0%	100.0%	0.0%	0.0%	100.0%	0.0%	0.0%	100.0%	0.0%	Premodern	0.06	0.077
LUB-09	0.0%	62.6%	37.4%	0.0%	62.1%	37.9%	0.0%	61.6%	38.4%	0.0%	61.6%	38.4%	Premodern	0.02	0.070
LUB-10	0.0%	100.0%	0.0%	0.0%	100.0%	0.0%	0.0%	100.0%	0.0%	0.0%	100.0%	0.0%	Premodern	0.30	0.064
LUB-11	0.0%	100.0%	0.0%	0.0%	100.0%	0.0%	0.0%	100.0%	0.0%	0.0%	100.0%	0.0%	Premodern	0.41	0.106
LUB-12	0.0%	97.3%	2.7%	0.0%	97.3%	2.7%	0.0%	97.3%	2.7%	0.0%	97.3%	2.7%	Premodern	1.45	0.096
LUB-13	0.0%	90.4%	9.6%	0.0%	90.0%	10.0%	0.0%	90.0%	10.0%	0.0%	90.0%	10.0%	Premodern	5.58	0.100
LUB-14	0.0%	96.4%	3.6%	0.0%	96.4%	3.6%	0.0%	96.4%	3.6%	0.0%	95.9%	4.1%	Premodern	0.16	0.188
LUB-15	0.0%	51.1%	48.9%	0.0%	51.1%	48.9%	0.0%	50.7%	49.3%	0.0%	50.2%	49.8%	Premodern	8.76	0.060
LUB-16	0.0%	100.0%	0.0%	0.0%	100.0%	0.0%	0.0%	100.0%	0.0%	0.0%	100.0%	0.0%	Modern	0.05	0.057
LUB-17	0.0%	100.0%	0.0%	0.0%	100.0%	0.0%	0.0%	100.0%	0.0%	0.0%	100.0%	0.0%	Premodern	0.05	0.064
LUB-18	0.0%	76.5%	23.5%	0.0%	76.5%	23.5%	0.0%	76.5%	23.5%	0.0%	76.5%	23.5%	Modern	4.56	0.262
LUB-19[2]	0.0%	100.0%	0.0%	0.0%	100.0%	0.0%	0.0%	100.0%	0.0%	0.0%	100.0%	0.0%	Premodern	0.16	0.067
LUB-20	0.5%	88.1%	11.5%	0.5%	87.2%	12.4%	0.0%	81.2%	18.8%	0.0%	69.3%	30.7%	Premodern	0.12	0.056
LUB-21	24.7%	66.2%	9.1%	0.9%	90.0%	9.1%	22.8%	68.0%	9.1%	20.1%	70.8%	9.1%	Premodern	0.03	0.061
LUB-22	0.0%	98.6%	1.4%	0.0%	78.5%	21.5%	0.0%	75.3%	24.7%	0.0%	74.4%	25.6%	Premodern	0.13	0.068
LUB-23	0.0%	100.0%	0.0%	0.0%	100.0%	0.0%	0.0%	100.0%	0.0%	0.0%	100.0%	0.0%	Premodern	0.05	0.100
LUB-24	0.0%	87.2%	12.8%	0.0%	87.2%	12.8%	0.0%	86.8%	13.2%	0.0%	86.8%	13.2%	Premodern	0.02	0.128
LUB-25	0.0%	94.5%	5.5%	0.0%	94.5%	5.5%	0.0%	94.5%	5.5%	0.0%	94.5%	5.5%	Premodern	0.01	0.113
LUB-26	0.0%	100.0%	0.0%	0.0%	100.0%	0.0%	0.0%	100.0%	0.0%	0.0%	100.0%	0.0%	Modern	0.02	0.150
LUB-27	0.0%	96.8%	3.2%	0.0%	96.3%	3.7%	0.0%	95.9%	4.1%	0.0%	95.9%	4.1%	Premodern	0.02	0.066
COA-01	0.5%	97.3%	2.3%	0.5%	97.3%	2.3%	0.5%	97.3%	2.3%	0.5%	97.3%	2.3%	Modern	13.45	0.266
COA-02[2]	42.5%	53.9%	3.7%	31.5%	64.8%	3.7%	47.9%	47.9%	4.1%	47.9%	47.9%	4.1%	Premodern	5.27	0.055

Online resource (suppplementary table) 2. Selected attributes of GAMA-PBP (https://ca.water.usgs.gov/gama/) status wells sampled for orthophosphate concentration-page 58.

GAMA-PBP ID	USGS Station ID[1]	GAMA-PBP study unit	GAMA-PBP study area[3]	Hydrogeologic zone
COA-03[2]	334700116170001	Coachella Valley	Coachella Valley	Desert
COA-04	334400116160001	Coachella Valley	Coachella Valley	Desert
COA-05	334548116203501	Coachella Valley	Coachella Valley	Desert
COA-06	334449116162201	Coachella Valley	Coachella Valley	Desert
COA-07	333700116160001	Coachella Valley	Coachella Valley	Desert
COA-08	334300116110001	Coachella Valley	Coachella Valley	Desert
COA-09[2]	334800116300001	Coachella Valley	Coachella Valley	Desert
COA-10[2]	333400116110001	Coachella Valley	Coachella Valley	Desert
COA-11[2]	333100116070001	Coachella Valley	Coachella Valley	Desert
COA-12	333300116020001	Coachella Valley	Coachella Valley	Desert
COA-13	335523116484601	Coachella Valley	Coachella Valley	Desert
COA-14	334551116242101	Coachella Valley	Coachella Valley	Desert
COA-15	335500116320001	Coachella Valley	Coachella Valley	Desert
COA-16	334700116100001	Coachella Valley	Coachella Valley	Desert
COA-17[2]	340100116370001	Coachella Valley	Coachella Valley	Desert
COA-18	333800116050001	Coachella Valley	Coachella Valley	Desert
COA-19[2]	335300116210001	Coachella Valley	Coachella Valley	Desert
COLOR-01[2]	325401114295201	Colorado River	Colorado River Valleys	Desert
COLOR-02[2]	325200114280001	Colorado River	Colorado River Valleys	Desert
COLOR-03	324400114380001	Colorado River	Colorado River Valleys	Desert
COLOR-04	332413114433501	Colorado River	Colorado River Valleys	Desert
COLOR-05	333700114340001	Colorado River	Colorado River Valleys	Desert
COLOR-06	333646114360901	Colorado River	Colorado River Valleys	Desert
COLOR-07	333059114334901	Colorado River	Colorado River Valleys	Desert
COLOR-08	333644114421601	Colorado River	Colorado River Valleys	Desert
COLOR-09[2]	333500114410001	Colorado River	Colorado River Valleys	Desert
COLOR-10	333130114394001	Colorado River	Colorado River Valleys	Desert
COLOR-11[2]	331806114440201	Colorado River	Colorado River Valleys	Desert
COLOR-12	344613114354101	Colorado River	Colorado River Valleys	Desert
COLOR-13	345209114383303	Colorado River	Colorado River Valleys	Desert

Online resource (suppplementary table) 2. Selected attributes of GAMA-PBP (https://ca.water.usgs.gov/gama/) status wells sampled for orthophosphate concentration-page 59.

GAMA-PBP ID	USEPA Level III Ecoregions[4]	Level III Ecoregion Reference Concentration[4]	Status Sample Date	Status Sample Orthophosphate Concentration (mg/L as P)	Relative Concentration Category[5]	Redox state[6]	Elevation of LSD (meters above NAVD 88)[7]	Well depth (meters below LSD)[8]	Agricultural land use in 1974[9] (percent)	Natural land use in 1974[9] (percent)	Urban land use in 1974[9] (percent)
COA-03[2]	Sonoran Basin and Range	0.0250	Feb 28 2007	0.010	low	oxic	45	na	60.0%	26.0%	14.0%
COA-04	Sonoran Basin and Range	0.0250	Mar 1 2007	0.011	low	oxic	24	326	0.0%	2.8%	97.2%
COA-05	Sonoran Basin and Range	0.0250	Mar 5 2007	0.011	low	oxic	69	332	0.0%	0.0%	100.0%
COA-06	Sonoran Basin and Range	0.0250	Mar 5 2007	0.015	low	anoxic	16	199	0.0%	1.4%	98.6%
COA-07	Sonoran Basin and Range	0.0250	Mar 6 2007	0.006	low	oxic	1	73	15.5%	73.6%	10.9%
COA-08	Sonoran Basin and Range	0.0250	Mar 6 2007	0.010	low	oxic	-5	104	22.8%	19.6%	57.5%
COA-09[2]	Sonoran Basin and Range	0.0250	Mar 7 2007	0.020	low	oxic	119	na	0.0%	0.0%	100.0%
COA-10[2]	Sonoran Basin and Range	0.0250	Mar 8 2007	0.012	low	anoxic	-32	na	68.0%	18.3%	13.7%
COA-11[2]	Sonoran Basin and Range	0.0250	Mar 8 2007	0.006	low	anoxic	-34	na	57.3%	33.6%	9.1%
COA-12	Sonoran Basin and Range	0.0250	Mar 8 2007	0.020	low	anoxic	-53	160	62.6%	29.7%	7.8%
COA-13	Southern California Mountains	0.0109	Mar 12 2007	0.022	moderate	oxic	586	271	5.0%	56.6%	38.4%
COA-14	Sonoran Basin and Range	0.0250	Mar 12 2007	0.012	low	oxic	71	250	13.7%	0.0%	86.3%
COA-15	Sonoran Basin and Range	0.0250	Mar 14 2007	0.018	low	oxic	266	122	0.0%	32.4%	67.6%
COA-16	Sonoran Basin and Range	0.0250	Mar 15 2007	0.006	low	oxic	146	198	0.0%	98.6%	1.4%
COA-17[2]	Mojave Basin and Range	0.0100	Mar 15 2007	0.008	low	oxic	755	na	0.0%	100.0%	0.0%
COA-18	Sonoran Basin and Range	0.0250	Mar 19 2007	0.005	low	oxic	13	241	23.4%	75.7%	0.9%
COA-19[2]	Sonoran Basin and Range	0.0250	Mar 29 2007	0.006	low	anoxic	324	na	0.0%	54.4%	45.6%
COLOR-01[2]	Sonoran Basin and Range	0.0250	Oct 1 2007	0.007	low	oxic	97	na	1.4%	98.6%	0.0%
COLOR-02[2]	Sonoran Basin and Range	0.0250	Oct 1 2007	0.012	low	oxic	54	na	3.2%	91.7%	5.0%
COLOR-03	Sonoran Basin and Range	0.0250	Oct 2 2007	0.054	moderate	anoxic	40	156	57.8%	1.8%	40.4%
COLOR-04	Sonoran Basin and Range	0.0250	Oct 3 2007	0.055	moderate	anoxic	71	27	42.2%	51.8%	6.0%
COLOR-05	Sonoran Basin and Range	0.0250	Oct 22 2007	0.020	low	anoxic	83	186	58.2%	0.0%	41.8%
COLOR-06	Sonoran Basin and Range	0.0250	Oct 24 2007	0.016	low	anoxic	82	154	0.0%	0.0%	100.0%
COLOR-07	Sonoran Basin and Range	0.0250	Oct 25 2007	0.009	low	anoxic	104	134	45.5%	51.2%	3.3%
COLOR-08	Sonoran Basin and Range	0.0250	Nov 1 2007	0.008	low	oxic	119	152	0.0%	42.1%	57.9%
COLOR-09[2]	Sonoran Basin and Range	0.0250	Nov 5 2007	0.009	low	anoxic	101	na	65.0%	34.6%	0.5%
COLOR-10	Sonoran Basin and Range	0.0250	Nov 6 2007	0.016	low	anoxic	75	305	60.8%	16.1%	23.0%
COLOR-11[2]	Sonoran Basin and Range	0.0250	Nov 7 2007	0.021	low	oxic	66	na	0.0%	97.7%	2.3%
COLOR-12	Mojave Basin and Range	0.0100	Nov 26 2007	0.006	low	oxic	223	128	0.0%	91.7%	8.3%
COLOR-13	Mojave Basin and Range	0.0100	Nov 27 2007	0.010	moderate	oxic	175	62	0.0%	59.1%	40.9%

Status and trends of orthophosphate concentrations in groundwater used for public supply in California *Environmental Monitoring and Assessment, Robert Kent, Tyler D. Johnson, and Michael R. Rosen, U.S. Geological Survey California Water Science Center-rhkent@usgs.gov*

Online resource (suppplementary table) 2. Selected attributes of GAMA-PBP (https://ca.water.usgs.gov/gama/) status wells sampled for orthophosphate concentration-page 60.

GAMA-PBP ID	Agricultural land use in 1982[9] (percent)	Natural land use in 1982[9] (percent)	Urban land use in 1982[9] (percent)	Agricultural land use in 1992[9] (percent)	Natural land use in 1992[9] (percent)	Urban land use in 1992[9] (percent)	Agricultural land use in 2002[9] (percent)	Natural land use in 2002[9] (percent)	Urban land use in 2002[9] (percent)	Agricultural land use in 2012[9] (percent)	Natural land use in 2012[9] (percent)	Urban land use in 2012[9] (percent)	Age Classification[10]	Septic Tanks[11]	Aridity[12]
COA-03[2]	36.3%	12.6%	51.2%	17.7%	7.0%	75.3%	0.0%	6.0%	94.0%	0.0%	0.0%	100.0%	Premodern	4.03	0.060
COA-04	0.0%	0.9%	99.1%	0.0%	0.5%	99.5%	0.0%	0.5%	99.5%	0.0%	0.0%	100.0%	Premodern	108.13	0.059
COA-05	0.0%	0.0%	100.0%	0.0%	0.0%	100.0%	0.0%	0.0%	100.0%	0.0%	0.0%	100.0%	Premodern	7.20	0.073
COA-06	0.5%	0.9%	98.6%	0.0%	0.5%	99.5%	0.0%	0.5%	99.5%	0.0%	0.0%	100.0%	Premodern	90.17	0.057
COA-07	15.5%	73.2%	11.4%	15.5%	69.5%	15.0%	14.1%	57.7%	28.2%	17.3%	36.8%	45.9%	Mixed	2.71	0.060
COA-08	29.2%	12.8%	58.0%	3.7%	3.7%	92.7%	0.9%	1.8%	97.3%	0.0%	1.8%	98.2%	Premodern	4.13	0.054
COA-09[2]	0.0%	0.0%	100.0%	0.0%	0.0%	100.0%	0.0%	0.0%	100.0%	0.0%	0.0%	100.0%	Modern	2.62	0.106
COA-10[2]	69.4%	16.4%	14.2%	67.6%	18.3%	14.2%	68.9%	16.9%	14.2%	69.4%	16.4%	14.2%	Premodern	2.32	0.058
COA-11[2]	56.4%	33.6%	10.0%	55.9%	33.6%	10.5%	55.9%	33.6%	10.5%	55.5%	34.1%	10.5%	Mixed	4.54	0.057
COA-12	80.8%	11.4%	7.8%	62.6%	29.7%	7.8%	62.6%	29.7%	7.8%	62.6%	29.2%	8.2%	Premodern	1.41	0.055
COA-13	1.8%	56.2%	42.0%	0.0%	56.2%	43.8%	0.0%	56.2%	43.8%	0.0%	49.8%	50.2%	Mixed	12.40	0.275
COA-14	2.7%	0.0%	97.3%	0.5%	0.0%	99.5%	0.0%	0.0%	100.0%	0.0%	0.0%	100.0%	Modern	7.88	0.089
COA-15	0.0%	32.4%	67.6%	0.0%	32.4%	67.6%	0.0%	32.4%	67.6%	0.0%	32.0%	68.0%	Premodern	19.93	0.123
COA-16	0.0%	98.6%	1.4%	0.0%	98.6%	1.4%	0.0%	98.6%	1.4%	0.0%	98.6%	1.4%	Premodern	0.68	0.067
COA-17[2]	0.0%	100.0%	0.0%	0.0%	100.0%	0.0%	0.0%	100.0%	0.0%	0.0%	100.0%	0.0%	Mixed	0.39	0.195
COA-18	23.4%	75.7%	0.9%	23.4%	75.7%	0.9%	23.4%	75.2%	1.4%	28.0%	70.6%	1.4%	Mixed	3.22	0.062
COA-19[2]	0.0%	52.1%	47.9%	0.0%	0.9%	99.1%	0.0%	0.9%	99.1%	0.0%	0.0%	100.0%	Premodern	8.60	0.119
COLOR-01[2]	1.4%	98.6%	0.0%	1.4%	98.6%	0.0%	3.7%	96.3%	0.0%	2.3%	97.7%	0.0%	Modern	0.05	0.063
COLOR-02[2]	3.2%	91.7%	5.0%	3.2%	91.7%	5.0%	20.2%	74.8%	5.0%	20.2%	74.8%	5.0%	Modern	2.82	0.062
COLOR-03	57.8%	1.8%	40.4%	56.4%	1.8%	41.7%	57.8%	1.4%	40.8%	57.8%	0.9%	41.3%	Mixed	8.25	0.063
COLOR-04	45.0%	48.6%	6.4%	44.5%	49.1%	6.4%	43.1%	48.2%	8.7%	43.1%	48.2%	8.7%	Modern	0.04	0.066
COLOR-05	56.8%	0.0%	43.2%	42.3%	0.0%	57.7%	40.9%	0.0%	59.1%	36.8%	0.0%	63.2%	Modern	12.43	0.068
COLOR-06	0.0%	0.0%	100.0%	0.0%	0.0%	100.0%	0.0%	0.0%	100.0%	0.0%	0.0%	100.0%	Mixed	18.52	0.067
COLOR-07	45.5%	51.2%	3.3%	45.5%	51.2%	3.3%	46.5%	50.2%	3.3%	46.5%	50.2%	3.3%	Modern	0.70	0.071
COLOR-08	0.0%	42.1%	57.9%	0.0%	42.1%	57.9%	0.0%	42.1%	57.9%	0.0%	42.1%	57.9%	Premodern	0.04	0.067
COLOR-09[2]	67.8%	31.8%	0.5%	64.5%	35.0%	0.5%	66.8%	32.7%	0.5%	66.8%	32.7%	0.5%	Premodern	0.64	0.066
COLOR-10	69.6%	7.4%	23.0%	61.3%	15.7%	23.0%	61.3%	15.7%	23.0%	61.3%	15.7%	23.0%	Modern	0.71	0.068
COLOR-11[2]	0.0%	97.7%	2.3%	0.0%	97.7%	2.3%	0.0%	97.7%	2.3%	0.0%	97.7%	2.3%	Modern	0.04	0.066
COLOR-12	0.0%	91.7%	8.3%	0.0%	91.7%	8.3%	0.0%	91.3%	8.7%	0.0%	91.3%	8.7%	Premodern	0.25	0.078
COLOR-13	0.0%	58.6%	41.4%	0.0%	58.6%	41.4%	0.0%	58.6%	41.4%	0.0%	58.6%	41.4%	Premodern	5.57	0.077

Online resource (suppplementary table) 2. Selected attributes of GAMA-PBP (https://ca.water.usgs.gov/gama/) status wells sampled for orthophosphate concentration-page 61.

GAMA-PBP ID	USGS Station ID[1]	GAMA-PBP study unit	GAMA-PBP study area[3]	Hydrogeologic zone
COLOR-14[2]	324452114443101	Colorado River	Colorado River Valleys	Desert
COLOR-15[2]	324846114325101	Colorado River	Colorado River Valleys	Desert
COLOR-16	334011114320103	Colorado River	Colorado River Valleys	Desert
COLOR-17	344300114310001	Colorado River	Colorado River Valleys	Desert
COLOR-18	333701114411301	Colorado River	Colorado River Valleys	Desert
COLOR-19	324522114344001	Colorado River	Colorado River Valleys	Desert
COLOR-20	344549114370501	Colorado River	Colorado River Valleys	Desert
MOJO-12	344300117200001	Mojave	Mojave River Valleys	Desert
MOJO-13[2]	343713117361601	Mojave	Mojave River Valleys	Desert
MOJO-14	343106117183101	Mojave	Mojave River Valleys	Desert
MOJO-15	342902117195101	Mojave	Mojave River Valleys	Desert
MOJO-16	342438117181401	Mojave	Mojave River Valleys	Desert
MOJO-17	342358117154802	Mojave	Mojave River Valleys	Desert
MOJO-18	342600117140001	Mojave	Mojave River Valleys	Desert
MOJO-19	343156117162701	Mojave	Mojave River Valleys	Desert
MOJO-25[2]	343600117210001	Mojave	Mojave River Valleys	Desert
MOJO-27	342601117205901	Mojave	Mojave River Valleys	Desert
MOJO-29	342800117090001	Mojave	Mojave River Valleys	Desert
MOJO-30	343419117213901	Mojave	Mojave River Valleys	Desert
MOJO-31	342800117310001	Mojave	Mojave River Valleys	Desert
MOJO-35	345350116515302	Mojave	Mojave River Valleys	Desert
MOJO-36	344800116360001	Mojave	Mojave River Valleys	Desert
MOJO-39[2]	345100117070001	Mojave	Mojave River Valleys	Desert
MOJO-41[2]	345400116390001	Mojave	Mojave River Valleys	Desert
MOJO-42	345127116473201	Mojave	Mojave River Valleys	Desert
MOJO-49	345551116520801	Mojave	Mojave River Valleys	Desert
OIW-01	353851117431001	Owens and Indian Wells Valleys	Indian Wells Valley	Desert
OIW-02	353800117490001	Owens and Indian Wells Valleys	Indian Wells Valley	Desert
OIW-03[2]	353251117554201	Owens and Indian Wells Valleys	Indian Wells Valley	Desert
OIW-04[2]	354800117520001	Owens and Indian Wells Valleys	Indian Wells Valley	Desert

Online resource (suppplementary table) 2. Selected attributes of GAMA-PBP (https://ca.water.usgs.gov/gama/) status wells sampled for orthophosphate concentration-page 62.

GAMA-PBP ID	USEPA Level III Ecoregions[4]	Level III Ecoregion Reference Concentration[4]	Status Sample Date	Status Sample Orthophosphate Concentration (mg/L as P)	Relative Concentration Category[5]	Redox state[6]	Elevation of LSD (meters above NAVD 88)[7]	Well depth (meters below LSD)[8]	Agricultural land use in 1974[9] (percent)	Natural land use in 1974[9] (percent)	Urban land use in 1974[9] (percent)
COLOR-14[2]	Sonoran Basin and Range	0.0250	Nov 28 2007	0.010	low	anoxic	75	na	0.0%	86.7%	13.3%
COLOR-15[2]	Sonoran Basin and Range	0.0250	Nov 29 2007	0.010	low	anoxic	42	na	92.3%	5.9%	1.8%
COLOR-16	Sonoran Basin and Range	0.0250	Dec 10 2007	0.026	moderate	anoxic	85	183	57.7%	36.3%	6.0%
COLOR-17	Mojave Basin and Range	0.0100	Dec 11 2007	0.007	low	oxic	162	64	0.0%	96.4%	3.6%
COLOR-18	Sonoran Basin and Range	0.0250	Dec 12 2007	0.009	low	oxic	103	183	8.6%	90.0%	1.4%
COLOR-19	Sonoran Basin and Range	0.0250	Dec 17 2007	0.059	moderate	oxic	42	44	81.7%	15.1%	3.2%
COLOR-20	Mojave Basin and Range	0.0100	Dec 18 2007	0.006	low	oxic	276	213	0.0%	44.0%	56.0%
MOJO-12	Mojave Basin and Range	0.0100	Feb 11 2008	0.020	moderate	oxic	751	110	0.0%	79.1%	20.9%
MOJO-13[2]	Mojave Basin and Range	0.0100	Feb 11 2008	0.010	low	anoxic	874	na	0.9%	62.3%	36.8%
MOJO-14	Mojave Basin and Range	0.0100	Feb 12 2008	0.019	moderate	oxic	898	162	0.0%	0.0%	100.0%
MOJO-15	Mojave Basin and Range	0.0100	Feb 12 2008	0.018	moderate	oxic	931	219	0.0%	0.0%	100.0%
MOJO-16	Mojave Basin and Range	0.0100	Feb 13 2008	0.008	low	oxic	992	305	0.0%	0.0%	100.0%
MOJO-17	Mojave Basin and Range	0.0100	Feb 13 2008	0.038	moderate	oxic	944	247	0.0%	0.0%	100.0%
MOJO-18	Mojave Basin and Range	0.0100	Feb 14 2008	0.036	moderate	oxic	871	140	0.0%	53.9%	46.1%
MOJO-19	Mojave Basin and Range	0.0100	Feb 14 2008	0.007	low	oxic	848	128	0.0%	0.5%	99.5%
MOJO-25[2]	Mojave Basin and Range	0.0100	Feb 27 2008	0.040	moderate	anoxic	799	na	2.3%	89.8%	7.9%
MOJO-27	Mojave Basin and Range	0.0100	Feb 28 2008	0.009	low	oxic	1,027	338	0.0%	0.0%	100.0%
MOJO-29	Mojave Basin and Range	0.0100	Mar 3 2008	0.011	moderate	oxic	918	146	0.0%	45.0%	55.0%
MOJO-30	Mojave Basin and Range	0.0100	Mar 4 2008	0.022	moderate	oxic	884	198	0.0%	0.0%	100.0%
MOJO-31	Mojave Basin and Range	0.0100	Mar 5 2008	0.011	moderate	anoxic	1,092	344	0.0%	98.2%	1.8%
MOJO-35	Mojave Basin and Range	0.0100	Mar 18 2008	0.048	moderate	oxic	598	122	0.9%	68.6%	30.5%
MOJO-36	Mojave Basin and Range	0.0100	Mar 19 2008	0.029	moderate	anoxic	548	43	0.0%	83.6%	16.4%
MOJO-39[2]	Mojave Basin and Range	0.0100	Mar 20 2008	0.012	moderate	oxic	701	na	0.0%	88.3%	11.7%
MOJO-41[2]	Mojave Basin and Range	0.0100	Mar 25 2008	0.011	moderate	oxic	555	na	0.0%	98.6%	1.4%
MOJO-42	Mojave Basin and Range	0.0100	Mar 26 2008	0.016	moderate	oxic	587	118	0.0%	29.0%	71.0%
MOJO-49	Mojave Basin and Range	0.0100	Apr 1 2008	0.015	moderate	oxic	612	122	0.0%	99.5%	0.5%
OIW-01	Mojave Basin and Range	0.0100	Oct 16 2006	0.034	moderate	oxic	717	259	0.0%	51.6%	48.4%
OIW-02	Mojave Basin and Range	0.0100	Oct 17 2006	0.014	moderate	oxic	746	146	0.0%	45.2%	54.8%
OIW-03[2]	Mojave Basin and Range	0.0100	Oct 17 2006	0.017	moderate	oxic	911	na	0.0%	92.5%	7.5%
OIW-04[2]	Mojave Basin and Range	0.0100	Oct 18 2006	0.046	moderate	na	752	122	0.0%	90.9%	9.1%

Status and trends of orthophosphate concentrations in groundwater used for public supply in California *Environmental Monitoring and Assessment, Robert Kent, Tyler D. Johnson, and Michael R. Rosen, U.S. Geological Survey California Water Science Center-rhkent@usgs.gov*

Online resource (suppplementary table) 2. Selected attributes of GAMA-PBP (https://ca.water.usgs.gov/gama/) status wells sampled for orthophosphate concentration-page 63.

GAMA-PBP ID	Agricultural land use in 1982[9] (percent)	Natural land use in 1982[9] (percent)	Urban land use in 1982[9] (percent)	Agricultural land use in 1992[9] (percent)	Natural land use in 1992[9] (percent)	Urban land use in 1992[9] (percent)	Agricultural land use in 2002[9] (percent)	Natural land use in 2002[9] (percent)	Urban land use in 2002[9] (percent)	Agricultural land use in 2012[9] (percent)	Natural land use in 2012[9] (percent)	Urban land use in 2012[9] (percent)	Age Classification[10]	Septic Tanks[11]	Aridity[12]
COLOR-14[2]	0.0%	86.7%	13.3%	0.0%	86.7%	13.3%	0.0%	86.7%	13.3%	0.0%	86.7%	13.3%	Premodern	0.05	0.057
COLOR-15[2]	92.3%	5.9%	1.8%	92.3%	5.9%	1.8%	98.2%	0.0%	1.8%	98.2%	0.0%	1.8%	Modern	4.78	0.063
COLOR-16	61.9%	32.1%	6.0%	57.7%	36.3%	6.0%	58.1%	35.8%	6.0%	58.1%	35.8%	6.0%	Modern	2.10	0.071
COLOR-17	0.0%	95.9%	4.1%	0.0%	95.9%	4.1%	0.0%	95.9%	4.1%	0.0%	95.9%	4.1%	Premodern	0.39	0.083
COLOR-18	10.0%	88.2%	1.8%	5.9%	91.4%	2.7%	5.9%	91.4%	2.7%	5.9%	91.4%	2.7%	Mixed	0.03	0.066
COLOR-19	81.7%	15.1%	3.2%	81.7%	15.1%	3.2%	81.7%	14.2%	4.1%	81.7%	14.2%	4.1%	Modern	4.78	0.065
COLOR-20	0.0%	44.0%	56.0%	0.0%	44.0%	56.0%	0.0%	44.0%	56.0%	0.0%	44.0%	56.0%	Premodern	0.01	0.086
MOJO-12	0.0%	78.2%	21.8%	0.0%	77.3%	22.7%	0.0%	77.3%	22.7%	4.1%	60.0%	35.9%	Modern	0.47	0.075
MOJO-13[2]	0.9%	62.3%	36.8%	0.0%	60.9%	39.1%	0.0%	59.5%	40.5%	0.0%	58.2%	41.8%	Premodern	0.58	0.091
MOJO-14	0.0%	0.0%	100.0%	0.0%	0.0%	100.0%	0.0%	0.0%	100.0%	0.0%	0.0%	100.0%	Premodern	7.51	0.108
MOJO-15	0.0%	0.0%	100.0%	0.0%	0.0%	100.0%	0.0%	0.0%	100.0%	0.0%	0.0%	100.0%	Premodern	58.35	0.133
MOJO-16	0.0%	0.0%	100.0%	0.0%	0.0%	100.0%	0.0%	0.0%	100.0%	0.0%	0.0%	100.0%	Premodern	99.30	0.174
MOJO-17	0.0%	0.0%	100.0%	0.0%	0.0%	100.0%	0.0%	0.0%	100.0%	0.0%	0.0%	100.0%	Modern	153.93	0.145
MOJO-18	0.0%	53.9%	46.1%	0.0%	39.7%	60.3%	0.0%	39.7%	60.3%	0.0%	0.0%	100.0%	Modern	20.40	0.109
MOJO-19	0.0%	0.5%	99.5%	0.0%	0.9%	99.1%	0.0%	0.9%	99.1%	0.0%	0.9%	99.1%	Mixed	65.36	0.096
MOJO-25[2]	2.3%	89.3%	8.4%	1.9%	89.3%	8.8%	0.5%	89.3%	10.2%	0.5%	89.3%	10.2%	Mixed	0.58	0.080
MOJO-27	0.0%	0.0%	100.0%	0.0%	0.0%	100.0%	0.0%	0.0%	100.0%	0.0%	0.0%	100.0%	Premodern	146.13	0.171
MOJO-29	0.0%	45.0%	55.0%	0.0%	45.0%	55.0%	0.0%	45.0%	55.0%	0.0%	45.0%	55.0%	Premodern	7.44	0.109
MOJO-30	0.0%	0.0%	100.0%	0.0%	0.0%	100.0%	0.0%	0.0%	100.0%	0.0%	0.0%	100.0%	Mixed	1.26	0.088
MOJO-31	0.0%	98.2%	1.8%	0.0%	50.7%	49.3%	0.0%	50.7%	49.3%	0.0%	50.2%	49.8%	Premodern	13.14	0.171
MOJO-35	0.9%	68.6%	30.5%	0.0%	69.5%	30.5%	0.0%	69.5%	30.5%	0.0%	69.5%	30.5%	Modern	1.88	0.071
MOJO-36	0.0%	83.6%	16.4%	0.0%	83.6%	16.4%	0.0%	83.6%	16.4%	0.0%	83.6%	16.4%	Premodern	0.30	0.065
MOJO-39[2]	0.0%	88.3%	11.7%	0.0%	87.8%	12.2%	0.0%	87.8%	12.2%	0.0%	87.3%	12.7%	Premodern	8.75	0.088
MOJO-41[2]	0.0%	98.6%	1.4%	0.0%	98.6%	1.4%	0.0%	96.8%	3.2%	0.0%	96.8%	3.2%	Premodern	3.81	0.064
MOJO-42	0.0%	29.0%	71.0%	0.0%	29.0%	71.0%	0.0%	29.0%	71.0%	0.0%	29.0%	71.0%	Premodern	3.82	0.065
MOJO-49	0.0%	99.5%	0.5%	0.0%	99.5%	0.5%	0.0%	99.5%	0.5%	0.0%	99.5%	0.5%	PremodernOrMixed	1.88	0.072
OIW-01	0.0%	51.6%	48.4%	0.0%	51.6%	48.4%	0.0%	51.6%	48.4%	0.0%	51.6%	48.4%	Premodern	16.68	0.079
OIW-02	0.0%	44.7%	55.3%	0.0%	44.3%	55.7%	0.0%	43.8%	56.2%	0.0%	43.8%	56.2%	Premodern	12.56	0.078
OIW-03[2]	0.0%	92.5%	7.5%	0.0%	92.1%	7.9%	0.0%	92.1%	7.9%	0.0%	92.1%	7.9%	PremodernOrMixed	0.46	0.101
OIW-04[2]	0.0%	90.5%	9.5%	0.0%	90.5%	9.5%	0.0%	90.5%	9.5%	0.0%	90.5%	9.5%	PremodernOrMixed	0.18	0.083

Online resource (suppplementary table) 2. Selected attributes of GAMA-PBP (https://ca.water.usgs.gov/gama/) status wells sampled for orthophosphate concentration-page 64.

GAMA-PBP ID	USGS Station ID[1]	GAMA-PBP study unit	GAMA-PBP study area[3]	Hydrogeologic zone
OIW-05	353601117483802	Owens and Indian Wells Valleys	Indian Wells Valley	Desert
OIW-06	353729117400301	Owens and Indian Wells Valleys	Indian Wells Valley	Desert
OIW-07	353740117414301	Owens and Indian Wells Valleys	Indian Wells Valley	Desert
OIW-08	354200117500001	Owens and Indian Wells Valleys	Indian Wells Valley	Desert
OIW-09	354000117520001	Owens and Indian Wells Valleys	Indian Wells Valley	Desert
OIW-10	354100117490001	Owens and Indian Wells Valleys	Indian Wells Valley	Desert
OIW-11	353851117383501	Owens and Indian Wells Valleys	Indian Wells Valley	Desert
OIW-12	354711117464001	Owens and Indian Wells Valleys	Indian Wells Valley	Desert
OIW-13	354900117390001	Owens and Indian Wells Valleys	Indian Wells Valley	Desert
OIWU-01	353900117460001	Owens and Indian Wells Valleys	Indian Wells Valley	Desert
OV-01	372200118250001	Owens and Indian Wells Valleys	Owens Valley	Desert
OV-02	371000118180001	Owens and Indian Wells Valleys	Owens Valley	Desert
OV-03[2]	372900118200001	Owens and Indian Wells Valleys	Owens Valley	Desert
OV-04	373000118380001	Owens and Indian Wells Valleys	Owens Valley	Desert
OV-05[2]	363400118030001	Owens and Indian Wells Valleys	Owens Valley	Desert
OV-06	365600118140001	Owens and Indian Wells Valleys	Owens Valley	Desert
OV-07	364953118144101	Owens and Indian Wells Valleys	Owens Valley	Desert
OV-08	372400118360001	Owens and Indian Wells Valleys	Owens Valley	Desert
OV-09[2]	371700118230001	Owens and Indian Wells Valleys	Owens Valley	Desert
OV-10[2]	372300118330001	Owens and Indian Wells Valleys	Owens Valley	Desert
OV-11	371900118320001	Owens and Indian Wells Valleys	Owens Valley	Desert
OV-12	363500118110001	Owens and Indian Wells Valleys	Owens Valley	Desert
OV-13	364700118170001	Owens and Indian Wells Valleys	Owens Valley	Desert
OV-14	364844118113401	Owens and Indian Wells Valleys	Owens Valley	Desert
OV-15	364600118070001	Owens and Indian Wells Valleys	Owens Valley	Desert
OV-16	370500118150001	Owens and Indian Wells Valleys	Owens Valley	Desert
OV-17	372100118190001	Owens and Indian Wells Valleys	Owens Valley	Desert
OV-18	373942118243401	Owens and Indian Wells Valleys	Owens Valley	Desert
OV-19[2]	363400118050001	Owens and Indian Wells Valleys	Owens Valley	Desert
OV-20[2]	374050118250101	Owens and Indian Wells Valleys	Owens Valley	Desert

Online resource (suppplementary table) 2. Selected attributes of GAMA-PBP (https://ca.water.usgs.gov/gama/) status wells sampled for orthophosphate concentration-page 65.

GAMA-PBP ID	USEPA Level III Ecoregions[4]	Level III Ecoregion Reference Concentration[4]	Status Sample Date	Status Sample Orthophosphate Concentration (mg/L as P)	Relative Concentration Category[5]	Redox state[6]	Elevation of LSD (meters above NAVD 88)[7]	Well depth (meters below LSD)[8]	Agricultural land use in 1974[9] (percent)	Natural land use in 1974[9] (percent)	Urban land use in 1974[9] (percent)
OIW-05	Mojave Basin and Range	0.0100	Oct 18 2006	0.025	moderate	oxic	781	311	0.0%	100.0%	0.0%
OIW-06	Mojave Basin and Range	0.0100	Oct 18 2006	0.013	moderate	oxic	699	71	0.0%	7.8%	92.2%
OIW-07	Mojave Basin and Range	0.0100	Oct 19 2006	0.018	moderate	anoxic	713	189	0.0%	52.5%	47.5%
OIW-08	Mojave Basin and Range	0.0100	Oct 30 2006	0.062	moderate	oxic	703	58	0.9%	96.8%	2.3%
OIW-09	Mojave Basin and Range	0.0100	Oct 31 2006	0.013	moderate	oxic	841	0	0.0%	89.9%	10.1%
OIW-10	Mojave Basin and Range	0.0100	Oct 31 2006	0.016	moderate	anoxic	710	79	0.0%	94.5%	5.5%
OIW-11	Mojave Basin and Range	0.0100	Dec 6 2006	0.038	moderate	anoxic	684	23	0.0%	74.5%	25.5%
OIW-12	Mojave Basin and Range	0.0100	Dec 7 2006	0.029	moderate	oxic	687	61	0.0%	100.0%	0.0%
OIW-13	Mojave Basin and Range	0.0100	Dec 14 2006	0.042	moderate	oxic	682	21	0.0%	100.0%	0.0%
OIWU-01	Mojave Basin and Range	0.0100	Oct 17 2006	0.011	moderate	oxic	736	372	0.0%	90.3%	9.7%
OV-01	Central Basin and Range	0.0288	Sep 11 2006	0.021	low	oxic	1,279	66	35.3%	0.9%	63.8%
OV-02	Central Basin and Range	0.0288	Sep 11 2006	0.024	low	oxic	1,221	38	37.9%	29.2%	32.9%
OV-03[2]	Central Basin and Range	0.0288	Sep 11 2006	0.014	low	na	1,314	76	0.0%	100.0%	0.0%
OV-04	Central Basin and Range	0.0288	Sep 11 2006	0.037	moderate	oxic	1,853	104	0.0%	100.0%	0.0%
OV-05[2]	Mojave Basin and Range	0.0100	Sep 12 2006	0.025	moderate	oxic	1,123	na	0.0%	100.0%	0.0%
OV-06	Central Basin and Range	0.0288	Sep 12 2006	0.082	moderate	anoxic	1,172	60	0.0%	86.4%	13.6%
OV-07	Central Basin and Range	0.0288	Sep 12 2006	0.019	low	oxic	1,311	198	0.0%	81.8%	18.2%
OV-08	Central Basin and Range	0.0288	Sep 13 2006	0.027	low	oxic	1,483	244	1.8%	76.3%	21.9%
OV-09[2]	Central Basin and Range	0.0288	Sep 13 2006	0.059	moderate	oxic	1,295	na	0.0%	61.5%	38.5%
OV-10[2]	Central Basin and Range	0.0288	Sep 13 2006	0.015	low	oxic	1,422	na	1.8%	98.2%	0.0%
OV-11	Central Basin and Range	0.0288	Sep 14 2006	0.013	low	oxic	1,781	49	7.9%	92.1%	0.0%
OV-12	Central Basin and Range	0.0288	Sep 14 2006	0.031	moderate	oxic	1,788	38	0.0%	99.5%	0.5%
OV-13	Central Basin and Range	0.0288	Sep 18 2006	0.008	low	oxic	1,839	35	0.0%	100.0%	0.0%
OV-14	Central Basin and Range	0.0288	Sep 19 2006	0.029	moderate	oxic	1,178	72	65.9%	33.2%	0.9%
OV-15	Mojave Basin and Range	0.0100	Sep 20 2006	0.030	moderate	oxic	1,142	62	0.0%	100.0%	0.0%
OV-16	Central Basin and Range	0.0288	Sep 20 2006	0.075	moderate	oxic	1,186	56	0.0%	100.0%	0.0%
OV-17	Central Basin and Range	0.0288	Sep 21 2006	0.028	low	oxic	1,232	188	0.9%	89.0%	10.1%
OV-18	Central Basin and Range	0.0288	Oct 2 2006	0.016	low	oxic	1,388	196	98.6%	0.9%	0.5%
OV-19[2]	Mojave Basin and Range	0.0100	Oct 2 2006	0.026	moderate	oxic	1,381	na	0.0%	77.4%	22.6%
OV-20[2]	Central Basin and Range	0.0288	Oct 2 2006	0.011	low	na	1,400	na	40.0%	59.1%	0.9%

Online resource (suppplementary table) 2. Selected attributes of GAMA-PBP (https://ca.water.usgs.gov/gama/) status wells sampled for orthophosphate concentration-page 66.

GAMA-PBP ID	Agricultural land use in 1982[9] (percent)	Natural land use in 1982[9] (percent)	Urban land use in 1982[9] (percent)	Agricultural land use in 1992[9] (percent)	Natural land use in 1992[9] (percent)	Urban land use in 1992[9] (percent)	Agricultural land use in 2002[9] (percent)	Natural land use in 2002[9] (percent)	Urban land use in 2002[9] (percent)	Agricultural land use in 2012[9] (percent)	Natural land use in 2012[9] (percent)	Urban land use in 2012[9] (percent)	Age Classification[10]	Septic Tanks[11]	Aridity[12]
OIW-05	0.0%	100.0%	0.0%	0.0%	100.0%	0.0%	0.0%	100.0%	0.0%	0.0%	100.0%	0.0%	Premodern	0.46	0.081
OIW-06	0.0%	7.8%	92.2%	0.0%	7.8%	92.2%	0.0%	7.8%	92.2%	0.0%	6.4%	93.6%	PremodernOrMixed	7.51	0.077
OIW-07	0.0%	47.0%	53.0%	0.0%	46.1%	53.9%	0.0%	42.0%	58.0%	0.0%	41.6%	58.4%	Premodern	11.51	0.079
OIW-08	0.0%	96.8%	3.2%	0.0%	95.4%	4.6%	0.0%	94.5%	5.5%	0.0%	94.5%	5.5%	Premodern	3.65	0.075
OIW-09	0.0%	88.9%	11.1%	0.0%	88.0%	12.0%	0.0%	86.2%	13.8%	0.0%	86.2%	13.8%	Modern	1.09	0.094
OIW-10	0.0%	93.6%	6.4%	0.0%	91.7%	8.3%	0.0%	91.3%	8.7%	0.0%	91.3%	8.7%	PremodernOrMixed	3.65	0.076
OIW-11	0.0%	74.5%	25.5%	0.0%	74.5%	25.5%	0.0%	74.5%	25.5%	0.0%	73.6%	26.4%	Mixed	0.00	0.077
OIW-12	0.0%	100.0%	0.0%	0.0%	100.0%	0.0%	0.0%	100.0%	0.0%	0.0%	100.0%	0.0%	Premodern	0.06	0.076
OIW-13	0.0%	100.0%	0.0%	0.0%	100.0%	0.0%	0.0%	100.0%	0.0%	0.0%	100.0%	0.0%	PremodernOrMixed	0.06	0.077
OIWU-01	0.0%	54.2%	45.8%	0.0%	54.2%	45.8%	0.0%	54.2%	45.8%	0.0%	54.2%	45.8%	Premodern	16.82	0.077
OV-01	31.7%	0.0%	68.3%	30.3%	0.0%	69.7%	29.4%	0.0%	70.6%	27.5%	1.8%	70.6%	Modern	0.01	0.100
OV-02	30.6%	29.2%	40.2%	30.6%	28.8%	40.6%	45.2%	11.9%	42.9%	45.2%	11.9%	42.9%	Modern	16.15	0.130
OV-03[2]	0.0%	100.0%	0.0%	0.0%	100.0%	0.0%	0.0%	100.0%	0.0%	0.0%	100.0%	0.0%	ModernOrMixed	0.11	0.106
OV-04	0.0%	100.0%	0.0%	0.0%	100.0%	0.0%	0.0%	100.0%	0.0%	0.0%	100.0%	0.0%	Modern	0.33	0.252
OV-05[2]	0.0%	100.0%	0.0%	0.0%	100.0%	0.0%	0.0%	100.0%	0.0%	0.0%	100.0%	0.0%	Modern	0.35	0.131
OV-06	0.0%	86.4%	13.6%	0.0%	86.4%	13.6%	0.0%	86.4%	13.6%	0.0%	86.4%	13.6%	Premodern	0.08	0.128
OV-07	0.0%	81.8%	18.2%	0.0%	81.8%	18.2%	0.0%	81.8%	18.2%	0.0%	81.8%	18.2%	Modern	0.19	0.133
OV-08	1.8%	76.3%	21.9%	1.8%	76.3%	21.9%	20.1%	58.0%	21.9%	20.1%	58.0%	21.9%	Modern	0.32	0.150
OV-09[2]	0.0%	58.4%	41.6%	0.0%	58.4%	41.6%	0.0%	58.4%	41.6%	0.0%	57.9%	42.1%	Modern	0.54	0.139
OV-10[2]	1.8%	98.2%	0.0%	1.8%	98.2%	0.0%	3.7%	96.3%	0.0%	3.7%	96.3%	0.0%	Modern	0.32	0.133
OV-11	9.3%	90.7%	0.0%	9.3%	90.7%	0.0%	13.6%	86.4%	0.0%	13.6%	86.4%	0.0%	Mixed	0.32	0.237
OV-12	0.0%	99.5%	0.5%	0.0%	99.5%	0.5%	0.0%	99.5%	0.5%	0.0%	99.5%	0.5%	Modern	0.35	0.205
OV-13	0.0%	100.0%	0.0%	0.0%	100.0%	0.0%	0.0%	100.0%	0.0%	0.0%	99.5%	0.5%	Mixed	0.19	0.225
OV-14	65.5%	33.2%	1.4%	65.5%	33.2%	1.4%	65.5%	33.2%	1.4%	56.8%	41.8%	1.4%	Modern	0.19	0.123
OV-15	0.0%	100.0%	0.0%	0.0%	100.0%	0.0%	0.0%	100.0%	0.0%	0.0%	100.0%	0.0%	Premodern	0.00	0.120
OV-16	0.0%	100.0%	0.0%	0.0%	100.0%	0.0%	0.0%	100.0%	0.0%	0.0%	100.0%	0.0%	Mixed	0.19	0.112
OV-17	0.9%	89.0%	10.1%	0.9%	89.0%	10.1%	20.6%	69.3%	10.1%	25.7%	64.2%	10.1%	Mixed	0.05	0.088
OV-18	98.6%	0.9%	0.5%	98.6%	0.9%	0.5%	98.6%	0.9%	0.5%	97.3%	2.3%	0.5%	Mixed	0.11	0.104
OV-19[2]	0.0%	76.5%	23.5%	0.0%	76.5%	23.5%	0.0%	76.5%	23.5%	0.0%	76.5%	23.5%	Modern	0.35	0.142
OV-20[2]	37.7%	61.4%	0.9%	37.7%	61.4%	0.9%	52.7%	46.4%	0.9%	38.6%	60.5%	0.9%	ModernOrMixed	0.11	0.105

Online resource (suppplementary table) 2. Selected attributes of GAMA-PBP (https://ca.water.usgs.gov/gama/) status wells sampled for orthophosphate concentration-page 67.

GAMA-PBP ID	USGS Station ID[1]	GAMA-PBP study unit	GAMA-PBP study area[3]	Hydrogeologic zone
OV-21	364300118080001	Owens and Indian Wells Valleys	Owens Valley	Desert
OV-22	364800118110001	Owens and Indian Wells Valleys	Owens Valley	Desert
OV-23	365500118140001	Owens and Indian Wells Valleys	Owens Valley	Desert
OV-24	370900118160001	Owens and Indian Wells Valleys	Owens Valley	Desert
OV-25	363617118041301	Owens and Indian Wells Valleys	Owens Valley	Desert
OV-26	372123118261501	Owens and Indian Wells Valleys	Owens Valley	Desert
OV-27	372400118200001	Owens and Indian Wells Valleys	Owens Valley	Desert
OV-28	373100118220001	Owens and Indian Wells Valleys	Owens Valley	Desert
OV-29	374900118280001	Owens and Indian Wells Valleys	Owens Valley	Desert
OV-30	372511118193301	Owens and Indian Wells Valleys	Owens Valley	Desert
OV-31	361900118010001	Owens and Indian Wells Valleys	Owens Valley	Desert
OV-32	362900117520001	Owens and Indian Wells Valleys	Owens Valley	Desert
OV-33	364154118034301	Owens and Indian Wells Valleys	Owens Valley	Desert
OV-34	363800118100001	Owens and Indian Wells Valleys	Owens Valley	Desert
OV-35	365300118150001	Owens and Indian Wells Valleys	Owens Valley	Desert
OV-36[2]	361400117580001	Owens and Indian Wells Valleys	Owens Valley	Desert
OV-37	372100118260001	Owens and Indian Wells Valleys	Owens Valley	Desert
OV-38	361200118010001	Owens and Indian Wells Valleys	Owens Valley	Desert
OV-39	363200118020001	Owens and Indian Wells Valleys	Owens Valley	Desert
OV-40	362700117530001	Owens and Indian Wells Valleys	Owens Valley	Desert
BEAR-G01	341200117040001	Bear Valley and Selected Hard Rock Areas	Hard Rock Lake Arrowhead	Mountain
BEAR-G02	341300117140001	Bear Valley and Selected Hard Rock Areas	Hard Rock Lake Arrowhead	Mountain
BEAR-G03	341500117160001	Bear Valley and Selected Hard Rock Areas	Hard Rock Lake Arrowhead	Mountain
BEAR-G04	341400117070001	Bear Valley and Selected Hard Rock Areas	Hard Rock Lake Arrowhead	Mountain
BEAR-G05	341400117080001	Bear Valley and Selected Hard Rock Areas	Hard Rock Lake Arrowhead	Mountain
BEAR-G06	341600117160001	Bear Valley and Selected Hard Rock Areas	Hard Rock Lake Arrowhead	Mountain
BEAR-G07	341539117130401	Bear Valley and Selected Hard Rock Areas	Hard Rock Lake Arrowhead	Mountain
BEAR-G08	341500117130001	Bear Valley and Selected Hard Rock Areas	Hard Rock Lake Arrowhead	Mountain
BEAR-G09	341600117130001	Bear Valley and Selected Hard Rock Areas	Hard Rock Lake Arrowhead	Mountain
BEAR-G10	341400117040001	Bear Valley and Selected Hard Rock Areas	Hard Rock Lake Arrowhead	Mountain

Online resource (suppplementary table) 2. Selected attributes of GAMA-PBP (https://ca.water.usgs.gov/gama/) status wells sampled for orthophosphate concentration-page 68.

GAMA-PBP ID	USEPA Level III Ecoregions[4]	Level III Ecoregion Reference Concentration[4]	Status Sample Date	Status Sample Orthophosphate Concentration (mg/L as P)	Relative Concentration Category[5]	Redox state[6]	Elevation of LSD (meters above NAVD 88)[7]	Well depth (meters below LSD)[8]	Agricultural land use in 1974[9] (percent)	Natural land use in 1974[9] (percent)	Urban land use in 1974[9] (percent)
OV-21	Central Basin and Range	0.0288	Oct 3 2006	0.050	moderate	oxic	1,169	78	0.0%	89.5%	10.5%
OV-22	Central Basin and Range	0.0288	Oct 3 2006	0.020	low	oxic	1,192	198	22.3%	35.8%	41.9%
OV-23	Central Basin and Range	0.0288	Oct 3 2006	0.051	moderate	oxic	1,167	57	0.9%	99.1%	0.0%
OV-24	Central Basin and Range	0.0288	Oct 4 2006	0.022	low	oxic	1,207	93	1.4%	47.0%	51.6%
OV-25	Mojave Basin and Range	0.0100	Oct 4 2006	0.022	moderate	oxic	1,149	119	0.0%	79.4%	20.6%
OV-26	Central Basin and Range	0.0288	Oct 4 2006	0.012	low	oxic	1,320	39	0.0%	38.4%	61.6%
OV-27	Central Basin and Range	0.0288	Oct 5 2006	0.015	low	oxic	1,256	61	19.5%	55.0%	25.5%
OV-28	Central Basin and Range	0.0288	Oct 5 2006	0.020	low	oxic	1,293	46	3.2%	56.9%	39.9%
OV-29	Central Basin and Range	0.0288	Oct 5 2006	0.017	low	oxic	1,638	61	0.0%	78.8%	21.2%
OV-30	Central Basin and Range	0.0288	Oct 5 2006	0.019	low	oxic	1,272	118	0.0%	98.2%	1.8%
OV-31	Mojave Basin and Range	0.0100	Oct 16 2006	0.023	moderate	oxic	1,105	73	0.0%	74.9%	25.1%
OV-32	Mojave Basin and Range	0.0100	Oct 23 2006	0.102	high	anoxic	1,118	38	0.0%	93.5%	6.5%
OV-33	Mojave Basin and Range	0.0100	Oct 23 2006	0.169	high	oxic	1,129	97	0.0%	100.0%	0.0%
OV-34	Central Basin and Range	0.0288	Oct 24 2006	0.023	low	oxic	1,540	213	0.0%	100.0%	0.0%
OV-35	Central Basin and Range	0.0288	Oct 24 2006	0.012	low	oxic	1,211	88	0.0%	100.0%	0.0%
OV-36[2]	Mojave Basin and Range	0.0100	Oct 25 2006	0.054	moderate	oxic	1,131	na	48.2%	49.1%	2.8%
OV-37	Central Basin and Range	0.0288	Oct 25 2006	0.012	low	oxic	1,334	122	4.7%	57.7%	37.6%
OV-38	Mojave Basin and Range	0.0100	Oct 30 2006	0.036	moderate	oxic	1,428	71	0.0%	100.0%	0.0%
OV-39	Mojave Basin and Range	0.0100	Nov 2 2006	0.046	moderate	anoxic	1,140	83	0.0%	86.4%	13.6%
OV-40	Mojave Basin and Range	0.0100	Dec 11 2006	0.211	high	oxic	1,086	236	0.0%	100.0%	0.0%
BEAR-G01	Southern California Mountains	0.0109	Apr 19 2010	0.018	moderate	oxic	1,952	70	0.0%	38.2%	61.8%
BEAR-G02	Southern California Mountains	0.0109	Apr 19 2010	0.058	moderate	oxic	1,647	0	0.0%	27.4%	72.6%
BEAR-G03	Southern California Mountains	0.0109	Apr 20 2010	0.026	moderate	oxic	1,449	91	0.0%	0.5%	99.5%
BEAR-G04	Southern California Mountains	0.0109	Apr 21 2010	0.021	moderate	oxic	1,629	0	0.0%	99.1%	0.9%
BEAR-G05	Southern California Mountains	0.0109	Apr 21 2010	0.010	low	oxic	1,659	0	0.0%	99.5%	0.5%
BEAR-G06	Southern California Mountains	0.0109	Apr 22 2010	0.019	moderate	oxic	1,171	0	0.0%	99.1%	0.9%
BEAR-G07	Southern California Mountains	0.0109	May 10 2010	0.016	moderate	oxic	1,568	152	0.0%	6.4%	93.6%
BEAR-G08	Southern California Mountains	0.0109	May 10 2010	0.032	moderate	oxic	1,586	214	0.0%	0.0%	100.0%
BEAR-G09	Southern California Mountains	0.0109	Aug 19 2010	0.012	moderate	oxic	1,440	152	0.0%	35.2%	64.8%
BEAR-G10	Southern California Mountains	0.0109	May 12 2010	0.009	low	oxic	2,117	67	0.0%	14.7%	85.3%

Online resource (suppplementary table) 2. Selected attributes of GAMA-PBP (https://ca.water.usgs.gov/gama/) status wells sampled for orthophosphate concentration-page 69.

GAMA-PBP ID	Agricultural land use in 1982[9] (percent)	Natural land use in 1982[9] (percent)	Urban land use in 1982[9] (percent)	Agricultural land use in 1992[9] (percent)	Natural land use in 1992[9] (percent)	Urban land use in 1992[9] (percent)	Agricultural land use in 2002[9] (percent)	Natural land use in 2002[9] (percent)	Urban land use in 2002[9] (percent)	Agricultural land use in 2012[9] (percent)	Natural land use in 2012[9] (percent)	Urban land use in 2012[9] (percent)	Age Classification[10]	Septic Tanks[11]	Aridity[12]
OV-21	0.0%	89.5%	10.5%	0.0%	89.0%	11.0%	0.0%	89.0%	11.0%	0.0%	89.0%	11.0%	ModernOrMixed	0.26	0.125
OV-22	22.3%	34.4%	43.3%	22.3%	34.4%	43.3%	22.3%	34.4%	43.3%	22.3%	34.4%	43.3%	Modern	0.16	0.126
OV-23	0.9%	99.1%	0.0%	0.9%	99.1%	0.0%	3.2%	96.8%	0.0%	3.2%	96.8%	0.0%	ModernOrMixed	0.00	0.125
OV-24	1.4%	44.7%	53.9%	0.0%	44.7%	55.3%	0.0%	44.3%	55.7%	0.0%	44.3%	55.7%	Modern	13.62	0.108
OV-25	0.0%	78.5%	21.5%	0.0%	78.5%	21.5%	0.0%	77.6%	22.4%	0.0%	77.6%	22.4%	Modern	0.35	0.124
OV-26	0.0%	37.9%	62.1%	0.0%	37.9%	62.1%	5.0%	32.9%	62.1%	5.0%	32.9%	62.1%	Modern	2.33	0.107
OV-27	19.5%	55.0%	25.5%	19.5%	55.0%	25.5%	25.9%	48.6%	25.5%	38.6%	35.9%	25.5%	Mixed	0.05	0.090
OV-28	3.2%	56.9%	39.9%	3.2%	56.9%	39.9%	4.1%	56.0%	39.9%	6.9%	53.2%	39.9%	PremodernOrMixed	0.11	0.098
OV-29	0.0%	78.8%	21.2%	0.0%	78.8%	21.2%	6.8%	71.6%	21.6%	6.8%	71.6%	21.6%	PremodernOrMixed	0.11	0.136
OV-30	0.0%	98.2%	1.8%	0.0%	98.2%	1.8%	0.0%	98.2%	1.8%	0.0%	98.2%	1.8%	Mixed	0.05	0.101
OV-31	0.0%	74.9%	25.1%	0.0%	74.4%	25.6%	0.0%	74.4%	25.6%	0.0%	74.4%	25.6%	Premodern	0.06	0.125
OV-32	0.0%	93.5%	6.5%	0.0%	93.5%	6.5%	0.0%	93.5%	6.5%	0.0%	93.5%	6.5%	Premodern	0.03	0.111
OV-33	0.0%	100.0%	0.0%	0.0%	100.0%	0.0%	0.0%	100.0%	0.0%	0.0%	100.0%	0.0%	PremodernOrMixed	0.05	0.129
OV-34	0.0%	100.0%	0.0%	0.0%	100.0%	0.0%	0.0%	100.0%	0.0%	0.0%	100.0%	0.0%	Premodern	0.35	0.164
OV-35	0.0%	100.0%	0.0%	0.0%	100.0%	0.0%	0.0%	100.0%	0.0%	0.0%	100.0%	0.0%	Mixed	0.19	0.130
OV-36[2]	48.2%	49.1%	2.8%	48.2%	49.1%	2.8%	48.2%	49.1%	2.8%	45.4%	51.8%	2.8%	Mixed	0.06	0.127
OV-37	4.7%	57.7%	37.6%	4.7%	57.7%	37.6%	38.5%	23.0%	38.5%	38.5%	23.0%	38.5%	Modern	0.86	0.109
OV-38	0.0%	100.0%	0.0%	0.0%	100.0%	0.0%	0.0%	100.0%	0.0%	0.0%	100.0%	0.0%	ModernOrMixed	0.06	0.149
OV-39	0.0%	86.4%	13.6%	0.0%	86.4%	13.6%	0.0%	85.9%	14.1%	0.0%	85.9%	14.1%	PremodernOrMixed	0.14	0.130
OV-40	0.0%	100.0%	0.0%	0.0%	100.0%	0.0%	0.0%	100.0%	0.0%	0.0%	100.0%	0.0%	Premodern	0.06	0.102
BEAR-G01	0.0%	37.8%	62.2%	0.0%	36.9%	63.1%	0.0%	31.3%	68.7%	0.0%	23.5%	76.5%	Mixed	0.33	0.661
BEAR-G02	0.0%	27.4%	72.6%	0.0%	27.4%	72.6%	0.0%	27.4%	72.6%	0.0%	27.4%	72.6%	Modern	2.06	0.715
BEAR-G03	0.0%	0.5%	99.5%	0.0%	0.5%	99.5%	0.0%	0.5%	99.5%	0.0%	0.5%	99.5%	Modern	11.69	0.667
BEAR-G04	0.0%	99.1%	0.9%	0.0%	98.6%	1.4%	0.0%	81.2%	18.8%	0.0%	75.1%	24.9%	Modern	0.37	0.611
BEAR-G05	0.0%	99.1%	0.9%	0.0%	99.1%	0.9%	0.0%	96.3%	3.7%	0.0%	95.9%	4.1%	Mixed	0.41	0.635
BEAR-G06	0.0%	99.1%	0.9%	0.0%	99.1%	0.9%	0.0%	99.1%	0.9%	0.0%	99.1%	0.9%	Mixed	2.26	0.492
BEAR-G07	0.0%	6.4%	93.6%	0.0%	6.4%	93.6%	0.0%	6.4%	93.6%	0.0%	6.4%	93.6%	Mixed	4.55	0.637
BEAR-G08	0.0%	0.0%	100.0%	0.0%	0.0%	100.0%	0.0%	0.0%	100.0%	0.0%	0.0%	100.0%	ModernOrMixed	4.19	0.680
BEAR-G09	0.0%	31.9%	68.1%	0.0%	31.9%	68.1%	0.0%	31.5%	68.5%	0.0%	31.5%	68.5%	Mixed	15.50	0.463
BEAR-G10	0.0%	14.7%	85.3%	0.0%	14.7%	85.3%	0.0%	14.7%	85.3%	0.0%	14.7%	85.3%	Mixed	0.54	0.642

Online resource (suppplementary table) 2. Selected attributes of GAMA-PBP (https://ca.water.usgs.gov/gama/) status wells sampled for orthophosphate concentration-page 70.

GAMA-PBP ID	USGS Station ID[1]	GAMA-PBP study unit	GAMA-PBP study area[3]	Hydrogeologic zone
BEAR-G11	341400117170001	Bear Valley and Selected Hard Rock Areas	Hard Rock Lake Arrowhead	Mountain
BEAR-G12	341500117040001	Bear Valley and Selected Hard Rock Areas	Hard Rock Lake Arrowhead	Mountain
BEAR-G13	341600117100001	Bear Valley and Selected Hard Rock Areas	Hard Rock Lake Arrowhead	Mountain
BEAR-S01	341400116530001	Bear Valley and Selected Hard Rock Areas	Bear Valley	Mountain
BEAR-S02	341423116534001	Bear Valley and Selected Hard Rock Areas	Bear Valley	Mountain
BEAR-S03	341600116560001	Bear Valley and Selected Hard Rock Areas	Bear Valley	Mountain
BEAR-S04	341500116540001	Bear Valley and Selected Hard Rock Areas	Bear Valley	Mountain
BEAR-S05	341400116510001	Bear Valley and Selected Hard Rock Areas	Bear Valley	Mountain
BEAR-S06	341400116500001	Bear Valley and Selected Hard Rock Areas	Bear Valley	Mountain
BEAR-S07	341300116510001	Bear Valley and Selected Hard Rock Areas	Bear Valley	Mountain
BEAR-S08	341400116490001	Bear Valley and Selected Hard Rock Areas	Bear Valley	Mountain
BEAR-S09	341400116480001	Bear Valley and Selected Hard Rock Areas	Bear Valley	Mountain
BEAR-S10	341300116460002	Bear Valley and Selected Hard Rock Areas	Bear Valley	Mountain
BEAR-S11	341547116503801	Bear Valley and Selected Hard Rock Areas	Bear Valley	Mountain
BEAR-S12	341559116495101	Bear Valley and Selected Hard Rock Areas	Bear Valley	Mountain
BEAR-S13	341500116480001	Bear Valley and Selected Hard Rock Areas	Bear Valley	Mountain
BEAR-S14	341321116483001	Bear Valley and Selected Hard Rock Areas	Bear Valley	Mountain
CAMP-ES-01	394800121340001	Cascade Range and Modoc Plateau	Eastside Sacramento Valley	Mountain
CAMP-ES-02	394400121340001	Cascade Range and Modoc Plateau	Eastside Sacramento Valley	Mountain
CAMP-ES-03	401800121520001	Cascade Range and Modoc Plateau	Eastside Sacramento Valley	Mountain
CAMP-ES-04	393900121370001	Cascade Range and Modoc Plateau	Eastside Sacramento Valley	Mountain
CAMP-ES-05	394500121360001	Cascade Range and Modoc Plateau	Eastside Sacramento Valley	Mountain
CAMP-ES-06	395300121390001	Cascade Range and Modoc Plateau	Eastside Sacramento Valley	Mountain
CAMP-ES-07	402000121450001	Cascade Range and Modoc Plateau	Eastside Sacramento Valley	Mountain
CAMP-ES-08	401800121460001	Cascade Range and Modoc Plateau	Eastside Sacramento Valley	Mountain
CAMP-ES-09	395200121460001	Cascade Range and Modoc Plateau	Eastside Sacramento Valley	Mountain
CAMP-ES-10	394600121410001	Cascade Range and Modoc Plateau	Eastside Sacramento Valley	Mountain
CAMP-ES-11	395200121400001	Cascade Range and Modoc Plateau	Eastside Sacramento Valley	Mountain
CAMP-ES-12	395500121430001	Cascade Range and Modoc Plateau	Eastside Sacramento Valley	Mountain
CAMP-ES-13	395300121440001	Cascade Range and Modoc Plateau	Eastside Sacramento Valley	Mountain

Online resource (suppplementary table) 2. Selected attributes of GAMA-PBP (https://ca.water.usgs.gov/gama/) status wells sampled for orthophosphate concentration-page 71.

GAMA-PBP ID	USEPA Level III Ecoregions[4]	Level III Ecoregion Reference Concentration[4]	Status Sample Date	Status Sample Orthophosphate Concentration (mg/L as P)	Relative Concentration Category[5]	Redox state[6]	Elevation of LSD (meters above NAVD 88)[7]	Well depth (meters below LSD)[8]	Agricultural land use in 1974[9] (percent)	Natural land use in 1974[9] (percent)	Urban land use in 1974[9] (percent)
BEAR-G11	Southern California Mountains	0.0109	May 13 2010	0.030	moderate	oxic	1,452	0	0.0%	21.3%	78.7%
BEAR-G12	Southern California Mountains	0.0109	May 19 2010	0.072	moderate	oxic	1,863	0	0.0%	100.0%	0.0%
BEAR-G13	Southern California Mountains	0.0109	Jun 30 2010	0.049	moderate	oxic	1,612	114	0.0%	24.8%	75.2%
BEAR-S01	Southern California Mountains	0.0109	Apr 26 2010	0.021	moderate	oxic	2,071	113	0.0%	33.9%	66.1%
BEAR-S02	Southern California Mountains	0.0109	Apr 26 2010	0.045	moderate	oxic	2,104	111	0.0%	7.4%	92.6%
BEAR-S03	Southern California Mountains	0.0109	Apr 27 2010	0.103	high	oxic	2,060	15	0.0%	29.1%	70.9%
BEAR-S04	Southern California Mountains	0.0109	Apr 27 2010	0.025	moderate	anoxic	2,063	88	0.0%	80.3%	19.7%
BEAR-S05	Southern California Mountains	0.0109	Apr 28 2010	0.013	moderate	oxic	2,108	216	0.0%	0.0%	100.0%
BEAR-S06	Southern California Mountains	0.0109	Apr 29 2010	0.022	moderate	oxic	2,202	179	0.0%	0.9%	99.1%
BEAR-S07	Southern California Mountains	0.0109	Apr 29 2010	0.055	moderate	oxic	2,151	101	0.0%	2.7%	97.3%
BEAR-S08	Southern California Mountains	0.0109	May 3 2010	0.042	moderate	oxic	2,141	232	0.0%	1.8%	98.2%
BEAR-S09	Southern California Mountains	0.0109	May 3 2010	0.074	moderate	oxic	2,071	122	0.9%	13.2%	85.9%
BEAR-S10	Southern California Mountains	0.0109	May 4 2010	0.028	moderate	oxic	2,217	82	0.0%	93.2%	6.8%
BEAR-S11	Southern California Mountains	0.0109	May 5 2010	0.018	moderate	oxic	2,057	163	0.0%	0.0%	100.0%
BEAR-S12	Southern California Mountains	0.0109	May 5 2010	0.060	moderate	oxic	2,048	53	0.0%	21.8%	78.2%
BEAR-S13	Southern California Mountains	0.0109	May 6 2010	0.092	moderate	oxic	2,062	72	0.0%	27.9%	72.1%
BEAR-S14	Southern California Mountains	0.0109	May 6 2010	0.015	moderate	oxic	2,214	0	0.0%	100.0%	0.0%
CAMP-ES-01	Central California Foothills and Coastal Mountains	0.0300	Sep 13 2010	0.134	high	oxic	717	122	0.0%	44.1%	55.9%
CAMP-ES-02	Central California Foothills and Coastal Mountains	0.0300	Sep 13 2010	0.083	moderate	oxic	514	99	0.0%	9.6%	90.4%
CAMP-ES-03	Central California Foothills and Coastal Mountains	0.0300	Sep 14 2010	0.107	high	oxic	660	0	0.0%	99.5%	0.5%
CAMP-ES-04	Central California Foothills and Coastal Mountains	0.0300	Sep 16 2010	0.072	moderate	oxic	117	145	11.0%	71.7%	17.4%
CAMP-ES-05	Central California Foothills and Coastal Mountains	0.0300	Sep 20 2010	0.097	moderate	oxic	589	168	0.0%	0.0%	100.0%
CAMP-ES-06	Central California Foothills and Coastal Mountains	0.0300	Sep 20 2010	0.103	high	oxic	793	283	0.0%	92.7%	7.3%
CAMP-ES-07	Sierra Nevada	0.0150	Sep 21 2010	0.110	high	oxic	942	24	0.0%	100.0%	0.0%
CAMP-ES-08	Central California Foothills and Coastal Mountains	0.0300	Oct 4 2010	0.112	high	oxic	1,014	118	0.0%	100.0%	0.0%
CAMP-ES-09	Central California Foothills and Coastal Mountains	0.0300	Oct 4 2010	0.116	high	oxic	465	223	0.0%	99.1%	0.9%
CAMP-ES-10	Central California Foothills and Coastal Mountains	0.0300	Oct 5 2010	0.112	high	oxic	463	235	0.0%	100.0%	0.0%
CAMP-ES-11	Central California Foothills and Coastal Mountains	0.0300	Oct 5 2010	0.117	high	oxic	703	146	0.0%	90.5%	9.5%
CAMP-ES-12	Central California Foothills and Coastal Mountains	0.0300	Oct 6 2010	0.018	low	oxic	860	303	0.0%	95.0%	5.0%
CAMP-ES-13	Central California Foothills and Coastal Mountains	0.0300	Oct 6 2010	0.065	moderate	oxic	626	29	0.0%	98.6%	1.4%

Online resource (suppplementary table) 2. Selected attributes of GAMA-PBP (https://ca.water.usgs.gov/gama/) status wells sampled for orthophosphate concentration-page 72.

GAMA-PBP ID	Agricultural land use in 1982[9] (percent)	Natural land use in 1982[9] (percent)	Urban land use in 1982[9] (percent)	Agricultural land use in 1992[9] (percent)	Natural land use in 1992[9] (percent)	Urban land use in 1992[9] (percent)	Agricultural land use in 2002[9] (percent)	Natural land use in 2002[9] (percent)	Urban land use in 2002[9] (percent)	Agricultural land use in 2012[9] (percent)	Natural land use in 2012[9] (percent)	Urban land use in 2012[9] (percent)	Age Classification[10]	Septic Tanks[11]	Aridity[12]
BEAR-G11	0.0%	21.3%	78.7%	0.0%	21.3%	78.7%	0.0%	21.3%	78.7%	0.0%	21.3%	78.7%	Modern	23.78	0.684
BEAR-G12	0.0%	100.0%	0.0%	0.0%	99.5%	0.5%	0.0%	98.2%	1.8%	0.0%	94.5%	5.5%	ModernOrMixed	0.97	0.582
BEAR-G13	0.0%	24.3%	75.7%	0.0%	24.3%	75.7%	0.0%	24.3%	75.7%	0.0%	24.3%	75.7%	Modern	0.00	0.552
BEAR-S01	0.0%	19.5%	80.5%	0.0%	18.6%	81.4%	0.0%	17.6%	82.4%	0.0%	16.7%	83.3%	Mixed	0.00	0.441
BEAR-S02	0.0%	4.7%	95.3%	0.0%	3.3%	96.7%	0.0%	3.3%	96.7%	0.0%	3.3%	96.7%	Mixed	0.02	0.475
BEAR-S03	0.0%	28.2%	71.8%	0.0%	27.7%	72.3%	0.0%	20.5%	79.5%	0.0%	10.5%	89.5%	Modern	1.04	0.560
BEAR-S04	0.0%	78.4%	21.6%	0.0%	77.0%	23.0%	0.0%	73.2%	26.8%	0.0%	64.3%	35.7%	Mixed	3.12	0.447
BEAR-S05	0.0%	0.0%	100.0%	0.0%	0.0%	100.0%	0.0%	0.0%	100.0%	0.0%	0.0%	100.0%	Premodern	0.47	0.452
BEAR-S06	0.0%	0.9%	99.1%	0.0%	0.5%	99.5%	0.0%	0.5%	99.5%	0.0%	0.5%	99.5%	Mixed	0.78	0.490
BEAR-S07	0.0%	0.5%	99.5%	0.0%	0.0%	100.0%	0.0%	0.0%	100.0%	0.0%	0.0%	100.0%	Modern	0.00	0.486
BEAR-S08	0.0%	1.8%	98.2%	0.0%	1.8%	98.2%	0.0%	1.8%	98.2%	0.0%	1.8%	98.2%	Premodern	1.57	0.483
BEAR-S09	0.9%	13.2%	85.9%	0.9%	10.0%	89.1%	0.9%	10.0%	89.1%	0.9%	10.0%	89.1%	Mixed	1.76	0.460
BEAR-S10	0.0%	93.2%	6.8%	0.0%	93.2%	6.8%	0.0%	92.7%	7.3%	0.0%	92.7%	7.3%	ModernOrMixed	2.13	0.494
BEAR-S11	0.0%	0.0%	100.0%	0.0%	0.0%	100.0%	0.0%	0.0%	100.0%	0.0%	0.0%	100.0%	ModernOrMixed	2.09	0.460
BEAR-S12	0.0%	21.8%	78.2%	0.0%	21.8%	78.2%	0.0%	21.8%	78.2%	0.0%	19.1%	80.9%	Premodern	1.27	0.461
BEAR-S13	0.0%	27.9%	72.1%	0.0%	27.9%	72.1%	0.0%	27.9%	72.1%	0.0%	27.4%	72.6%	Mixed	1.16	0.459
BEAR-S14	0.0%	100.0%	0.0%	0.0%	100.0%	0.0%	0.0%	100.0%	0.0%	0.0%	100.0%	0.0%	ModernOrMixed	1.53	0.539
CAMP-ES-01	0.0%	5.6%	94.4%	0.0%	5.6%	94.4%	0.0%	5.6%	94.4%	0.0%	5.6%	94.4%	Modern	111.91	1.353
CAMP-ES-02	0.0%	9.1%	90.9%	0.0%	9.1%	90.9%	0.0%	0.0%	100.0%	0.0%	0.0%	100.0%	Modern	39.83	1.139
CAMP-ES-03	0.0%	99.1%	0.9%	0.0%	98.6%	1.4%	0.0%	97.2%	2.8%	0.0%	97.2%	2.8%	Premodern	0.42	0.748
CAMP-ES-04	12.3%	70.3%	17.4%	10.5%	72.1%	17.4%	10.5%	71.7%	17.8%	10.5%	70.8%	18.7%	Mixed	4.74	0.622
CAMP-ES-05	0.0%	0.0%	100.0%	0.0%	0.0%	100.0%	0.0%	0.0%	100.0%	0.0%	0.0%	100.0%	Modern	255.86	1.251
CAMP-ES-06	0.0%	92.2%	7.8%	0.0%	92.2%	7.8%	0.0%	92.2%	7.8%	0.0%	92.2%	7.8%	Modern	3.13	1.372
CAMP-ES-07	0.0%	100.0%	0.0%	0.0%	100.0%	0.0%	0.0%	100.0%	0.0%	0.0%	100.0%	0.0%	Modern	0.42	0.828
CAMP-ES-08	0.0%	100.0%	0.0%	0.0%	100.0%	0.0%	0.0%	100.0%	0.0%	0.0%	100.0%	0.0%	Modern	0.42	0.869
CAMP-ES-09	0.0%	99.1%	0.9%	0.0%	99.1%	0.9%	0.0%	99.1%	0.9%	0.0%	99.1%	0.9%	Mixed	1.65	0.994
CAMP-ES-10	0.0%	100.0%	0.0%	0.0%	100.0%	0.0%	0.0%	100.0%	0.0%	0.0%	100.0%	0.0%	Premodern	4.04	1.021
CAMP-ES-11	0.0%	90.5%	9.5%	0.0%	90.5%	9.5%	0.0%	90.5%	9.5%	0.0%	90.5%	9.5%	Modern	2.94	1.326
CAMP-ES-12	0.0%	95.0%	5.0%	0.0%	95.0%	5.0%	0.0%	95.0%	5.0%	0.0%	95.0%	5.0%	Modern	1.65	1.284
CAMP-ES-13	0.0%	98.6%	1.4%	0.0%	98.6%	1.4%	0.0%	98.6%	1.4%	0.0%	98.6%	1.4%	Modern	1.65	1.158

Online resource (suppplementary table) 2. Selected attributes of GAMA-PBP (https://ca.water.usgs.gov/gama/) status wells sampled for orthophosphate concentration-page 73.

GAMA-PBP ID	USGS Station ID[1]	GAMA-PBP study unit	GAMA-PBP study area[3]	Hydrogeologic zone
CAMP-ES-14[2]	395000121490001	Cascade Range and Modoc Plateau	Eastside Sacramento Valley	Mountain
CAMP-ES-15	394600121460001	Cascade Range and Modoc Plateau	Eastside Sacramento Valley	Mountain
CAMP-HL-01	402200120150001	Cascade Range and Modoc Plateau	Honey Lake Valley	Mountain
CAMP-HL-02	402100120380001	Cascade Range and Modoc Plateau	Honey Lake Valley	Mountain
CAMP-HL-03[2]	402300120370001	Cascade Range and Modoc Plateau	Honey Lake Valley	Mountain
CAMP-HL-04	402210120314401	Cascade Range and Modoc Plateau	Honey Lake Valley	Mountain
CAMP-HL-05	402300120350001	Cascade Range and Modoc Plateau	Honey Lake Valley	Mountain
CAMP-HL-06	401500120270001	Cascade Range and Modoc Plateau	Honey Lake Valley	Mountain
CAMP-HL-07	401900120220001	Cascade Range and Modoc Plateau	Honey Lake Valley	Mountain
CAMP-HL-08	402100120180001	Cascade Range and Modoc Plateau	Honey Lake Valley	Mountain
CAMP-HL-09	400800120080001	Cascade Range and Modoc Plateau	Honey Lake Valley	Mountain
CAMP-HL-10	400800120080002	Cascade Range and Modoc Plateau	Honey Lake Valley	Mountain
CAMP-HL-11	402100120250001	Cascade Range and Modoc Plateau	Honey Lake Valley	Mountain
CAMP-HL-12	400200120070001	Cascade Range and Modoc Plateau	Honey Lake Valley	Mountain
CAMP-HL-13	400900120220001	Cascade Range and Modoc Plateau	Honey Lake Valley	Mountain
CAMP-HL-14	402400120260001	Cascade Range and Modoc Plateau	Honey Lake Valley	Mountain
CAMP-HL-15	402300120230001	Cascade Range and Modoc Plateau	Honey Lake Valley	Mountain
CAMP-LU-01	415700121550001	Cascade Range and Modoc Plateau	Low-use basins of the Cascade Range and Modoc Plateau	Mountain
CAMP-LU-02	415700121280001	Cascade Range and Modoc Plateau	Low-use basins of the Cascade Range and Modoc Plateau	Mountain
CAMP-LU-03	414900120530001	Cascade Range and Modoc Plateau	Low-use basins of the Cascade Range and Modoc Plateau	Mountain
CAMP-LU-04	413200120100001	Cascade Range and Modoc Plateau	Low-use basins of the Cascade Range and Modoc Plateau	Mountain
CAMP-LU-05	412900120310001	Cascade Range and Modoc Plateau	Low-use basins of the Cascade Range and Modoc Plateau	Mountain
CAMP-LU-06	412600120520001	Cascade Range and Modoc Plateau	Low-use basins of the Cascade Range and Modoc Plateau	Mountain
CAMP-LU-07	414706122001701	Cascade Range and Modoc Plateau	Low-use basins of the Cascade Range and Modoc Plateau	Mountain
CAMP-LU-08	411800122050001	Cascade Range and Modoc Plateau	Low-use basins of the Cascade Range and Modoc Plateau	Mountain
CAMP-LU-09	414100120670001	Cascade Range and Modoc Plateau	Low-use basins of the Cascade Range and Modoc Plateau	Mountain
CAMP-LU-10	415301121222101	Cascade Range and Modoc Plateau	Low-use basins of the Cascade Range and Modoc Plateau	Mountain
CAMP-LU-11	411100120560001	Cascade Range and Modoc Plateau	Low-use basins of the Cascade Range and Modoc Plateau	Mountain
CAMP-LU-12	403300121430001	Cascade Range and Modoc Plateau	Low-use basins of the Cascade Range and Modoc Plateau	Mountain
CAMP-LU-13	410700121080001	Cascade Range and Modoc Plateau	Low-use basins of the Cascade Range and Modoc Plateau	Mountain

Online resource (suppplementary table) 2. Selected attributes of GAMA-PBP (https://ca.water.usgs.gov/gama/) status wells sampled for orthophosphate concentration-page 74.

GAMA-PBP ID	USEPA Level III Ecoregions[4]	Level III Ecoregion Reference Concentration[4]	Status Sample Date	Status Sample Orthophosphate Concentration (mg/L as P)	Relative Concentration Category[5]	Redox state[6]	Elevation of LSD (meters above NAVD 88)[7]	Well depth (meters below LSD)[8]	Agricultural land use in 1974[9] (percent)	Natural land use in 1974[9] (percent)	Urban land use in 1974[9] (percent)
CAMP-ES-14[2]	Central California Foothills and Coastal Mountains	0.0300	Oct 7 2010	0.100	high	oxic	126	na	0.0%	92.3%	7.7%
CAMP-ES-15	Central California Foothills and Coastal Mountains	0.0300	Oct 12 2010	0.107	high	oxic	90	146	0.0%	1.9%	98.1%
CAMP-HL-01	Northern Basin and Range	0.0550	Aug 10 2010	0.213	high	oxic	1,229	91	0.0%	99.5%	0.5%
CAMP-HL-02	Sierra Nevada	0.0150	Aug 11 2010	0.032	moderate	oxic	1,324	73	0.9%	96.3%	2.7%
CAMP-HL-03[2]	Sierra Nevada	0.0150	Aug 11 2010	0.062	moderate	oxic	1,263	na	35.2%	24.7%	40.2%
CAMP-HL-04	Sierra Nevada	0.0150	Aug 12 2010	0.100	moderate	oxic	1,249	183	55.3%	31.3%	13.4%
CAMP-HL-05	Sierra Nevada	0.0150	Aug 18 2010	0.282	high	oxic	1,262	58	28.1%	35.3%	36.7%
CAMP-HL-06	Northern Basin and Range	0.0550	Aug 18 2010	0.041	low	oxic	1,252	17	42.5%	32.4%	25.1%
CAMP-HL-07	Northern Basin and Range	0.0550	Aug 19 2010	0.440	high	oxic	1,223	61	36.2%	62.9%	0.9%
CAMP-HL-08	Northern Basin and Range	0.0550	Aug 19 2010	1.029	high	anoxic	1,221	63	46.8%	52.3%	0.9%
CAMP-HL-09	Central Basin and Range	0.0288	Aug 23 2010	0.077	moderate	oxic	1,260	162	8.6%	50.9%	40.5%
CAMP-HL-10	Central Basin and Range	0.0288	Aug 23 2010	0.086	moderate	oxic	1,257	166	1.9%	75.5%	22.7%
CAMP-HL-11	Northern Basin and Range	0.0550	Aug 24 2010	0.319	high	anoxic	1,234	69	65.6%	17.9%	16.5%
CAMP-HL-12	Central Basin and Range	0.0288	Aug 24 2010	0.091	moderate	oxic	1,287	75	0.0%	56.2%	43.8%
CAMP-HL-13	Sierra Nevada	0.0150	Aug 31 2010	0.082	moderate	oxic	1,364	40	0.0%	100.0%	0.0%
CAMP-HL-14	Northern Basin and Range	0.0550	Aug 31 2010	0.110	high	anoxic	1,248	31	46.1%	53.9%	0.0%
CAMP-HL-15	Northern Basin and Range	0.0550	Sep 1 2010	1.222	high	oxic	1,237	37	0.5%	68.0%	31.5%
CAMP-LU-01	Eastern Cascades Slopes and Foothills	0.0300	Jul 19 2010	0.073	moderate	anoxic	1,294	377	22.4%	4.6%	73.1%
CAMP-LU-02	Eastern Cascades Slopes and Foothills	0.0300	Jul 19 2010	0.366	high	anoxic	1,231	812	55.9%	0.0%	44.1%
CAMP-LU-03	Eastern Cascades Slopes and Foothills	0.0300	Jul 21 2010	0.079	moderate	oxic	1,335	204	9.6%	2.8%	87.6%
CAMP-LU-04	Northern Basin and Range	0.0550	Jul 21 2010	0.048	low	oxic	1,422	108	21.8%	28.6%	49.5%
CAMP-LU-05	Eastern Cascades Slopes and Foothills	0.0300	Jul 22 2010	0.072	moderate	oxic	1,338	152	25.0%	14.1%	60.9%
CAMP-LU-06	Eastern Cascades Slopes and Foothills	0.0300	Jul 22 2010	0.551	high	anoxic	1,316	67	52.8%	12.8%	34.4%
CAMP-LU-07	Eastern Cascades Slopes and Foothills	0.0300	Jul 27 2010	0.087	moderate	oxic	1,299	27	65.6%	17.2%	17.2%
CAMP-LU-08	Cascades	0.0091	Jul 29 2010	0.053	moderate	oxic	1,243	0	0.0%	100.0%	0.0%
CAMP-LU-09	Eastern Cascades Slopes and Foothills	0.0300	Aug 2 2010	0.114	high	oxic	1,341	94	22.3%	69.5%	8.2%
CAMP-LU-10	Eastern Cascades Slopes and Foothills	0.0300	Aug 3 2010	0.176	high	anoxic	1,235	105	0.5%	75.9%	23.6%
CAMP-LU-11	Eastern Cascades Slopes and Foothills	0.0300	Aug 4 2010	0.173	high	oxic	1,288	56	26.8%	55.0%	18.2%
CAMP-LU-12	Sierra Nevada	0.0150	Aug 5 2010	0.061	moderate	oxic	1,258	45	0.0%	100.0%	0.0%
CAMP-LU-13	Eastern Cascades Slopes and Foothills	0.0300	Aug 9 2010	0.149	high	oxic	1,257	105	27.6%	28.1%	44.2%

Online resource (suppplementary table) 2. Selected attributes of GAMA-PBP (https://ca.water.usgs.gov/gama/) status wells sampled for orthophosphate concentration-page 75.

GAMA-PBP ID	Agricultural land use in 1982[9] (percent)	Natural land use in 1982[9] (percent)	Urban land use in 1982[9] (percent)	Agricultural land use in 1992[9] (percent)	Natural land use in 1992[9] (percent)	Urban land use in 1992[9] (percent)	Agricultural land use in 2002[9] (percent)	Natural land use in 2002[9] (percent)	Urban land use in 2002[9] (percent)	Agricultural land use in 2012[9] (percent)	Natural land use in 2012[9] (percent)	Urban land use in 2012[9] (percent)	Age Classification[10]	Septic Tanks[11]	Aridity[12]
CAMP-ES-14[2]	0.0%	90.9%	9.1%	0.0%	89.5%	10.5%	0.0%	89.1%	10.9%	0.0%	89.1%	10.9%	Premodern	1.65	0.668
CAMP-ES-15	0.0%	1.9%	98.1%	0.0%	1.9%	98.1%	0.0%	1.9%	98.1%	0.0%	1.9%	98.1%	Modern	1.65	0.616
CAMP-HL-01	0.0%	99.5%	0.5%	0.0%	99.5%	0.5%	10.6%	88.9%	0.5%	10.6%	88.9%	0.5%	Premodern	0.37	0.194
CAMP-HL-02	0.9%	96.3%	2.7%	0.9%	94.1%	5.0%	0.9%	94.1%	5.0%	0.9%	94.1%	5.0%	Mixed	2.22	0.375
CAMP-HL-03[2]	34.7%	24.7%	40.6%	34.7%	24.2%	41.1%	37.4%	22.4%	40.2%	37.4%	21.0%	41.6%	Mixed	1.70	0.359
CAMP-HL-04	55.3%	31.3%	13.4%	55.3%	31.3%	13.4%	57.1%	29.5%	13.4%	59.4%	26.3%	14.3%	Mixed	4.25	0.332
CAMP-HL-05	28.1%	35.3%	36.7%	27.1%	35.3%	37.6%	27.6%	34.8%	37.6%	27.6%	31.2%	41.2%	Mixed	4.25	0.359
CAMP-HL-06	42.5%	32.0%	25.6%	42.5%	32.0%	25.6%	42.5%	32.0%	25.6%	42.5%	32.0%	25.6%	Modern	4.25	0.320
CAMP-HL-07	36.2%	62.9%	0.9%	36.2%	62.9%	0.9%	39.8%	59.3%	0.9%	39.8%	59.3%	0.9%	Premodern	1.28	0.214
CAMP-HL-08	51.4%	47.7%	0.9%	46.8%	52.3%	0.9%	51.4%	47.7%	0.9%	53.7%	45.4%	0.9%	Premodern	1.09	0.200
CAMP-HL-09	8.6%	50.9%	40.5%	8.6%	50.9%	40.5%	11.4%	48.2%	40.5%	11.4%	48.2%	40.5%	Premodern	0.00	0.217
CAMP-HL-10	1.4%	75.5%	23.1%	1.4%	75.5%	23.1%	2.3%	74.5%	23.1%	2.3%	74.5%	23.1%	Mixed	0.00	0.217
CAMP-HL-11	65.6%	17.9%	16.5%	65.6%	17.9%	16.5%	65.6%	16.1%	18.3%	65.6%	16.1%	18.3%	Mixed	1.28	0.235
CAMP-HL-12	0.0%	55.8%	44.2%	0.0%	55.3%	44.7%	0.0%	54.8%	45.2%	0.0%	53.9%	46.1%	Modern	0.69	0.259
CAMP-HL-13	0.0%	100.0%	0.0%	0.0%	100.0%	0.0%	0.0%	100.0%	0.0%	0.0%	100.0%	0.0%	Modern	1.10	0.407
CAMP-HL-14	46.1%	53.9%	0.0%	46.1%	53.9%	0.0%	48.9%	51.1%	0.0%	45.7%	54.3%	0.0%	Premodern	1.12	0.270
CAMP-HL-15	0.5%	67.6%	32.0%	0.5%	67.6%	32.0%	0.5%	67.6%	32.0%	0.5%	67.6%	32.0%	Mixed	0.53	0.241
CAMP-LU-01	22.4%	4.6%	73.1%	22.4%	4.6%	73.1%	23.3%	4.1%	72.6%	23.3%	4.1%	72.6%	Premodern	0.25	0.387
CAMP-LU-02	52.7%	0.0%	47.3%	52.3%	0.0%	47.7%	51.8%	0.0%	48.2%	51.8%	0.0%	48.2%	Premodern	0.20	0.286
CAMP-LU-03	9.6%	2.8%	87.6%	9.6%	2.8%	87.6%	9.6%	2.8%	87.6%	9.6%	2.3%	88.1%	Mixed	1.24	0.297
CAMP-LU-04	21.8%	28.6%	49.5%	21.8%	28.6%	49.5%	21.8%	28.6%	49.5%	21.8%	28.6%	49.5%	Modern	0.43	0.324
CAMP-LU-05	25.0%	14.1%	60.9%	25.0%	14.1%	60.9%	25.0%	14.1%	60.9%	25.0%	14.1%	60.9%	Mixed	1.21	0.296
CAMP-LU-06	56.0%	9.2%	34.9%	54.6%	9.2%	36.2%	54.6%	9.2%	36.2%	54.6%	9.2%	36.2%	Premodern	0.11	0.404
CAMP-LU-07	71.5%	10.4%	18.1%	63.3%	3.2%	33.5%	63.3%	3.2%	33.5%	59.3%	7.2%	33.5%	Modern	0.25	0.324
CAMP-LU-08	0.0%	100.0%	0.0%	0.0%	100.0%	0.0%	0.0%	100.0%	0.0%	0.0%	100.0%	0.0%	Modern	0.11	1.527
CAMP-LU-09	25.5%	66.4%	8.2%	25.5%	66.4%	8.2%	25.5%	65.9%	8.6%	25.5%	65.9%	8.6%	Premodern	0.22	0.337
CAMP-LU-10	2.3%	74.1%	23.6%	2.3%	73.6%	24.1%	2.3%	73.6%	24.1%	2.3%	73.2%	24.5%	Premodern	0.12	0.291
CAMP-LU-11	26.4%	55.0%	18.6%	26.4%	55.0%	18.6%	26.4%	55.0%	18.6%	26.4%	55.0%	18.6%	Mixed	0.19	0.391
CAMP-LU-12	0.0%	100.0%	0.0%	0.0%	100.0%	0.0%	0.0%	100.0%	0.0%	0.0%	100.0%	0.0%	ModernOrMixed	1.26	1.115
CAMP-LU-13	27.6%	28.1%	44.2%	27.6%	28.1%	44.2%	35.5%	20.3%	44.2%	35.5%	19.8%	44.7%	Modern	0.16	0.451

Online resource (suppplementary table) 2. Selected attributes of GAMA-PBP (https://ca.water.usgs.gov/gama/) status wells sampled for orthophosphate concentration-page 76.

GAMA-PBP ID	USGS Station ID[1]	GAMA-PBP study unit	GAMA-PBP study area[3]	Hydrogeologic zone
CAMP-LU-14	401800121140001	Cascade Range and Modoc Plateau	Low-use basins of the Cascade Range and Modoc Plateau	Mountain
CAMP-LU-15	401700121140001	Cascade Range and Modoc Plateau	Low-use basins of the Cascade Range and Modoc Plateau	Mountain
CAMP-QV-01	413400121360001	Cascade Range and Modoc Plateau	Quaternary volcanic areas	Mountain
CAMP-QV-02	411900122070001	Cascade Range and Modoc Plateau	Quaternary volcanic areas	Mountain
CAMP-QV-03	415200120670001	Cascade Range and Modoc Plateau	Quaternary volcanic areas	Mountain
CAMP-QV-04	415200121220001	Cascade Range and Modoc Plateau	Quaternary volcanic areas	Mountain
CAMP-QV-05	405500121370001	Cascade Range and Modoc Plateau	Quaternary volcanic areas	Mountain
CAMP-QV-06[2]	402500120380001	Cascade Range and Modoc Plateau	Quaternary volcanic areas	Mountain
CAMP-QV-07	405200121390001	Cascade Range and Modoc Plateau	Quaternary volcanic areas	Mountain
CAMP-QV-08	405500121330001	Cascade Range and Modoc Plateau	Quaternary volcanic areas	Mountain
CAMP-QV-09	401700121020001	Cascade Range and Modoc Plateau	Quaternary volcanic areas	Mountain
CAMP-QV-10	403700121540001	Cascade Range and Modoc Plateau	Quaternary volcanic areas	Mountain
CAMP-QV-11	402900121540001	Cascade Range and Modoc Plateau	Quaternary volcanic areas	Mountain
CAMP-QV-12	403100121560001	Cascade Range and Modoc Plateau	Quaternary volcanic areas	Mountain
CAMP-QV-13	402700121480001	Cascade Range and Modoc Plateau	Quaternary volcanic areas	Mountain
CAMP-QV-14	401800121250001	Cascade Range and Modoc Plateau	Quaternary volcanic areas	Mountain
CAMP-QV-15	403000121490001	Cascade Range and Modoc Plateau	Quaternary volcanic areas	Mountain
CAMP-SH-01	414100122380001	Cascade Range and Modoc Plateau	Shasta Valley and Shasta Volcanic area	Mountain
CAMP-SH-02	412800122270001	Cascade Range and Modoc Plateau	Shasta Valley and Shasta Volcanic area	Mountain
CAMP-SH-03	413100122310001	Cascade Range and Modoc Plateau	Shasta Valley and Shasta Volcanic area	Mountain
CAMP-SH-04	412400122220001	Cascade Range and Modoc Plateau	Shasta Valley and Shasta Volcanic area	Mountain
CAMP-SH-05	412500122210001	Cascade Range and Modoc Plateau	Shasta Valley and Shasta Volcanic area	Mountain
CAMP-SH-06	411900122182101	Cascade Range and Modoc Plateau	Shasta Valley and Shasta Volcanic area	Mountain
CAMP-SH-07	411800122120001	Cascade Range and Modoc Plateau	Shasta Valley and Shasta Volcanic area	Mountain
CAMP-SH-08[2]	412500122240001	Cascade Range and Modoc Plateau	Shasta Valley and Shasta Volcanic area	Mountain
CAMP-SH-09	413800122310001	Cascade Range and Modoc Plateau	Shasta Valley and Shasta Volcanic area	Mountain
CAMP-SH-10	413100122220001	Cascade Range and Modoc Plateau	Shasta Valley and Shasta Volcanic area	Mountain
CAMP-SH-11[2]	413200122220001	Cascade Range and Modoc Plateau	Shasta Valley and Shasta Volcanic area	Mountain
CAMP-SH-12	413700122240001	Cascade Range and Modoc Plateau	Shasta Valley and Shasta Volcanic area	Mountain
CAMP-SH-13	413600122240001	Cascade Range and Modoc Plateau	Shasta Valley and Shasta Volcanic area	Mountain

Online resource (suppplementary table) 2. Selected attributes of GAMA-PBP (https://ca.water.usgs.gov/gama/) status wells sampled for orthophosphate concentration-page 77.

GAMA-PBP ID	USEPA Level III Ecoregions[4]	Level III Ecoregion Reference Concentration[4]	Status Sample Date	Status Sample Orthophosphate Concentration (mg/L as P)	Relative Concentration Category[5]	Redox state[6]	Elevation of LSD (meters above NAVD 88)[7]	Well depth (meters below LSD)[8]	Agricultural land use in 1974[9] (percent)	Natural land use in 1974[9] (percent)	Urban land use in 1974[9] (percent)
CAMP-LU-14	Sierra Nevada	0.0150	Aug 30 2010	0.079	moderate	oxic	1,387	72	0.0%	22.6%	77.4%
CAMP-LU-15	Sierra Nevada	0.0150	Aug 30 2010	0.032	moderate	oxic	1,380	113	0.0%	6.4%	93.6%
CAMP-QV-01	Eastern Cascades Slopes and Foothills	0.0300	Jul 20 2010	0.063	moderate	oxic	2,099	0	0.0%	100.0%	0.0%
CAMP-QV-02	Cascades	0.0091	Jul 29 2010	0.049	moderate	oxic	1,380	0	0.0%	100.0%	0.0%
CAMP-QV-03	Eastern Cascades Slopes and Foothills	0.0300	Aug 2 2010	0.182	high	oxic	1,528	226	0.0%	100.0%	0.0%
CAMP-QV-04	Eastern Cascades Slopes and Foothills	0.0300	Aug 3 2010	0.194	high	oxic	1,247	99	0.0%	99.1%	0.9%
CAMP-QV-05	Eastern Cascades Slopes and Foothills	0.0300	Aug 9 2010	0.042	moderate	oxic	975	69	0.0%	24.4%	75.6%
CAMP-QV-06[2]	Sierra Nevada	0.0150	Aug 12 2010	0.046	moderate	oxic	1,279	na	0.5%	50.5%	49.1%
CAMP-QV-07	Sierra Nevada	0.0150	Aug 17 2010	0.036	moderate	oxic	993	91	0.0%	40.7%	59.3%
CAMP-QV-08	Eastern Cascades Slopes and Foothills	0.0300	Aug 17 2010	0.073	moderate	oxic	981	74	0.0%	91.4%	8.6%
CAMP-QV-09	Sierra Nevada	0.0150	Aug 25 2010	0.031	moderate	oxic	1,519	0	0.0%	100.0%	0.0%
CAMP-QV-10	Sierra Nevada	0.0150	Sep 13 2010	0.060	moderate	oxic	689	53	0.0%	100.0%	0.0%
CAMP-QV-11	Central California Foothills and Coastal Mountains	0.0300	Sep 13 2010	0.046	moderate	oxic	1,017	52	0.0%	97.3%	2.7%
CAMP-QV-12	Central California Foothills and Coastal Mountains	0.0300	Sep 14 2010	0.050	moderate	oxic	789	71	0.0%	100.0%	0.0%
CAMP-QV-13	Sierra Nevada	0.0150	Sep 14 2010	0.093	moderate	oxic	901	56	0.0%	100.0%	0.0%
CAMP-QV-14	Sierra Nevada	0.0150	Sep 15 2010	0.164	high	oxic	1,437	142	0.0%	93.4%	6.6%
CAMP-QV-15	Sierra Nevada	0.0150	Sep 20 2010	0.056	moderate	oxic	1,136	46	0.0%	94.5%	5.5%
CAMP-SH-01	Klamath Mountains/California High North Coast Range	0.0325	Jul 12 2010	0.033	moderate	oxic	836	37	0.0%	31.2%	68.8%
CAMP-SH-02	Klamath Mountains/California High North Coast Range	0.0325	Jul 13 2010	0.247	high	oxic	900	71	1.4%	61.5%	37.1%
CAMP-SH-03	Klamath Mountains/California High North Coast Range	0.0325	Jul 13 2010	0.038	moderate	oxic	847	33	74.5%	10.9%	14.5%
CAMP-SH-04	Cascades	0.0091	Jul 14 2010	0.187	high	oxic	1,134	137	0.0%	73.7%	26.3%
CAMP-SH-05	Cascades	0.0091	Jul 14 2010	0.186	high	oxic	1,170	0	0.9%	99.1%	0.0%
CAMP-SH-06	Cascades	0.0091	Jul 15 2010	0.081	moderate	oxic	1,154	100	0.0%	0.0%	100.0%
CAMP-SH-07	Cascades	0.0091	Jul 15 2010	0.037	moderate	oxic	1,361	0	0.0%	100.0%	0.0%
CAMP-SH-08[2]	Klamath Mountains/California High North Coast Range	0.0325	Jul 26 2010	0.120	high	oxic	1,006	na	6.1%	68.1%	25.8%
CAMP-SH-09	Klamath Mountains/California High North Coast Range	0.0325	Jul 27 2010	0.050	moderate	oxic	785	70	42.7%	21.1%	36.2%
CAMP-SH-10	Klamath Mountains/California High North Coast Range	0.0325	Jul 28 2010	0.181	high	oxic	866	94	0.0%	79.0%	21.0%
CAMP-SH-11[2]	Klamath Mountains/California High North Coast Range	0.0325	Jul 28 2010	0.193	high	anoxic	842	na	67.3%	30.4%	2.3%
CAMP-SH-12	Klamath Mountains/California High North Coast Range	0.0325	Sep 22 2010	0.322	high	oxic	805	46	90.4%	6.8%	2.7%
CAMP-SH-13	Klamath Mountains/California High North Coast Range	0.0325	Sep 23 2010	0.167	high	oxic	794	0	73.6%	25.9%	0.5%

Online resource (suppplementary table) 2. Selected attributes of GAMA-PBP (https://ca.water.usgs.gov/gama/) status wells sampled for orthophosphate concentration-page 78.

GAMA-PBP ID	Agricultural land use in 1982[9] (percent)	Natural land use in 1982[9] (percent)	Urban land use in 1982[9] (percent)	Agricultural land use in 1992[9] (percent)	Natural land use in 1992[9] (percent)	Urban land use in 1992[9] (percent)	Agricultural land use in 2002[9] (percent)	Natural land use in 2002[9] (percent)	Urban land use in 2002[9] (percent)	Agricultural land use in 2012[9] (percent)	Natural land use in 2012[9] (percent)	Urban land use in 2012[9] (percent)	Age Classification[10]	Septic Tanks[11]	Aridity[12]
CAMP-LU-14	0.0%	22.6%	77.4%	0.0%	22.6%	77.4%	0.0%	22.6%	77.4%	0.0%	22.6%	77.4%	Mixed	0.71	0.804
CAMP-LU-15	0.0%	6.4%	93.6%	0.0%	6.4%	93.6%	0.0%	6.4%	93.6%	0.0%	6.4%	93.6%	Modern	1.95	0.797
CAMP-QV-01	0.0%	100.0%	0.0%	0.0%	100.0%	0.0%	0.0%	100.0%	0.0%	0.0%	100.0%	0.0%	Modern	0.01	1.384
CAMP-QV-02	0.0%	100.0%	0.0%	0.0%	100.0%	0.0%	0.0%	100.0%	0.0%	0.0%	100.0%	0.0%	Modern	0.11	1.740
CAMP-QV-03	0.0%	100.0%	0.0%	0.0%	100.0%	0.0%	0.0%	100.0%	0.0%	0.0%	100.0%	0.0%	Premodern	0.11	0.321
CAMP-QV-04	4.1%	95.0%	0.9%	16.4%	82.6%	0.9%	16.4%	82.6%	0.9%	16.4%	82.6%	0.9%	Mixed	0.12	0.313
CAMP-QV-05	0.0%	21.7%	78.3%	0.0%	21.7%	78.3%	0.0%	21.7%	78.3%	0.0%	21.7%	78.3%	ModernOrMixed	39.10	0.541
CAMP-QV-06[2]	0.5%	50.5%	49.1%	0.5%	50.5%	49.1%	0.5%	50.5%	49.1%	1.4%	49.5%	49.1%	Mixed	1.17	0.367
CAMP-QV-07	0.0%	40.7%	59.3%	0.0%	40.7%	59.3%	0.0%	40.7%	59.3%	0.0%	40.7%	59.3%	Modern	0.33	0.672
CAMP-QV-08	0.0%	91.4%	8.6%	0.0%	91.4%	8.6%	0.0%	91.4%	8.6%	0.0%	91.4%	8.6%	Mixed	0.49	0.477
CAMP-QV-09	0.0%	100.0%	0.0%	0.0%	100.0%	0.0%	0.0%	100.0%	0.0%	0.0%	100.0%	0.0%	Modern	0.99	0.807
CAMP-QV-10	0.0%	100.0%	0.0%	0.0%	100.0%	0.0%	0.0%	100.0%	0.0%	0.0%	100.0%	0.0%	Modern	1.11	1.008
CAMP-QV-11	0.0%	96.8%	3.2%	0.0%	96.8%	3.2%	0.0%	96.8%	3.2%	0.0%	96.8%	3.2%	Modern	1.83	0.925
CAMP-QV-12	0.0%	100.0%	0.0%	0.0%	100.0%	0.0%	0.0%	100.0%	0.0%	0.0%	100.0%	0.0%	Modern	1.53	0.845
CAMP-QV-13	0.0%	100.0%	0.0%	0.0%	100.0%	0.0%	0.0%	100.0%	0.0%	0.0%	100.0%	0.0%	Mixed	0.74	0.901
CAMP-QV-14	0.0%	92.5%	7.5%	0.0%	92.5%	7.5%	0.0%	92.5%	7.5%	0.0%	92.5%	7.5%	Premodern	0.42	1.310
CAMP-QV-15	0.0%	48.4%	51.6%	0.0%	48.4%	51.6%	0.0%	48.4%	51.6%	0.0%	48.4%	51.6%	Mixed	1.37	1.093
CAMP-SH-01	0.0%	28.1%	71.9%	0.0%	28.1%	71.9%	0.0%	28.1%	71.9%	0.0%	28.1%	71.9%	Mixed	6.28	0.481
CAMP-SH-02	1.4%	61.5%	37.1%	1.4%	61.5%	37.1%	1.4%	61.5%	37.1%	1.4%	61.1%	37.6%	Mixed	1.24	0.818
CAMP-SH-03	75.0%	10.5%	14.5%	74.1%	10.9%	15.0%	75.5%	10.9%	13.6%	74.5%	11.8%	13.6%	Modern	1.62	0.812
CAMP-SH-04	0.0%	10.6%	89.4%	0.0%	10.6%	89.4%	0.0%	8.3%	91.7%	0.0%	7.8%	92.2%	Premodern	2.12	0.898
CAMP-SH-05	0.9%	99.1%	0.0%	0.9%	99.1%	0.0%	8.3%	91.7%	0.0%	8.3%	91.7%	0.0%	Mixed	1.75	0.791
CAMP-SH-06	0.0%	0.0%	100.0%	0.0%	0.0%	100.0%	0.0%	0.0%	100.0%	0.0%	0.0%	100.0%	Mixed	18.76	1.073
CAMP-SH-07	0.0%	100.0%	0.0%	0.0%	100.0%	0.0%	0.0%	100.0%	0.0%	0.0%	100.0%	0.0%	Modern	1.93	1.324
CAMP-SH-08[2]	6.1%	67.6%	26.3%	6.1%	60.1%	33.8%	6.1%	60.1%	33.8%	6.1%	60.1%	33.8%	Mixed	1.13	0.703
CAMP-SH-09	40.8%	21.1%	38.1%	40.8%	21.1%	38.1%	40.8%	21.1%	38.1%	40.8%	21.1%	38.1%	Modern	2.37	0.550
CAMP-SH-10	0.0%	71.7%	28.3%	0.0%	67.6%	32.4%	0.0%	46.6%	53.4%	0.0%	34.2%	65.8%	Mixed	1.30	0.706
CAMP-SH-11[2]	70.0%	27.6%	2.3%	67.3%	30.4%	2.3%	70.0%	27.6%	2.3%	66.4%	31.3%	2.3%	Modern	1.30	0.652
CAMP-SH-12	90.9%	6.4%	2.7%	90.9%	6.4%	2.7%	93.6%	2.3%	4.1%	93.6%	2.3%	4.1%	Modern	0.78	0.470
CAMP-SH-13	73.6%	25.9%	0.5%	73.6%	25.9%	0.5%	75.9%	23.6%	0.5%	74.5%	25.0%	0.5%	Mixed	1.30	0.493

Online resource (suppplementary table) 2. Selected attributes of GAMA-PBP (https://ca.water.usgs.gov/gama/) status wells sampled for orthophosphate concentration-page 79.

GAMA-PBP ID	USGS Station ID[1]	GAMA-PBP study unit	GAMA-PBP study area[3]	Hydrogeologic zone
CAMP-SH-14	412259122235101	Cascade Range and Modoc Plateau	Shasta Valley and Shasta Volcanic area	Mountain
CAMP-SH-15	414200122330001	Cascade Range and Modoc Plateau	Shasta Valley and Shasta Volcanic area	Mountain
CAMP-TV-01	415500122210001	Cascade Range and Modoc Plateau	Tertiary volcanic area	Mountain
CAMP-TV-02[2]	410100121400001	Cascade Range and Modoc Plateau	Tertiary volcanic area	Mountain
CAMP-TV-03	402500121590001	Cascade Range and Modoc Plateau	Tertiary volcanic area	Mountain
CAMP-TV-04	404800121300001	Cascade Range and Modoc Plateau	Tertiary volcanic area	Mountain
CAMP-TV-05	405300121100001	Cascade Range and Modoc Plateau	Tertiary volcanic area	Mountain
CAMP-TV-06	401800121090001	Cascade Range and Modoc Plateau	Tertiary volcanic area	Mountain
CAMP-TV-07	410800120160001	Cascade Range and Modoc Plateau	Tertiary volcanic area	Mountain
CAMP-TV-08	404300120430001	Cascade Range and Modoc Plateau	Tertiary volcanic area	Mountain
CAMP-TV-09	401400121120001	Cascade Range and Modoc Plateau	Tertiary volcanic area	Mountain
CAMP-TV-10[2]	401200121100001	Cascade Range and Modoc Plateau	Tertiary volcanic area	Mountain
CAMP-TV-11	405100121500001	Cascade Range and Modoc Plateau	Tertiary volcanic area	Mountain
CAMP-TV-12	400400121330001	Cascade Range and Modoc Plateau	Tertiary volcanic area	Mountain
CAMP-TV-13	400500121330001	Cascade Range and Modoc Plateau	Tertiary volcanic area	Mountain
CAMP-TV-14	405100121530001	Cascade Range and Modoc Plateau	Tertiary volcanic area	Mountain
CAMP-TV-15	415500122270001	Cascade Range and Modoc Plateau	Tertiary volcanic area	Mountain
CGOLD-01	370900119500001	Central Sierra	Coarse Gold watershed	Mountain
CGOLD-02	371200119400001	Central Sierra	Coarse Gold watershed	Mountain
CGOLD-03	371300119430001	Central Sierra	Coarse Gold watershed	Mountain
CGOLD-04	372100119410001	Central Sierra	Coarse Gold watershed	Mountain
CGOLD-05	372100119430001	Central Sierra	Coarse Gold watershed	Mountain
CGOLD-06	372500119430001	Central Sierra	Coarse Gold watershed	Mountain
CGOLD-07	372700119380001	Central Sierra	Coarse Gold watershed	Mountain
CGOLD-08	371900119380001	Central Sierra	Coarse Gold watershed	Mountain
CGOLD-09	370700119520001	Central Sierra	Coarse Gold watershed	Mountain
CGOLD-10	371600119370001	Central Sierra	Coarse Gold watershed	Mountain
CGOLD-11[2]	371600119420001	Central Sierra	Coarse Gold watershed	Mountain
CGOLD-12	371000119450001	Central Sierra	Coarse Gold watershed	Mountain
CGOLD-13	371200119460001	Central Sierra	Coarse Gold watershed	Mountain

Status and trends of orthophosphate concentrations in groundwater used for public supply in California *Environmental Monitoring and Assessment, Robert Kent, Tyler D. Johnson, and Michael R. Rosen, U.S. Geological Survey California Water Science Center-rhkent@usgs.gov*

Online resource (supplementary table) 2. Selected attributes of GAMA-PBP (https://ca.water.usgs.gov/gama/) status wells sampled for orthophosphate concentration-page 80.

GAMA-PBP ID	USEPA Level III Ecoregions[4]	Level III Ecoregion Reference Concentration[4]	Status Sample Date	Status Sample Orthophosphate Concentration (mg/L as P)	Relative Concentration Category[5]	Redox state[6]	Elevation of LSD (meters above NAVD 88)[7]	Well depth (meters below LSD)[8]	Agricultural land use in 1974[9] (percent)	Natural land use in 1974[9] (percent)	Urban land use in 1974[9] (percent)
CAMP-SH-14	Cascades	0.0091	Oct 13 2010	0.059	moderate	oxic	1,248	43	0.0%	96.4%	3.6%
CAMP-SH-15	Klamath Mountains/California High North Coast Range	0.0325	Oct 14 2010	0.011	low	anoxic	783	37	99.5%	0.0%	0.5%
CAMP-TV-01	Eastern Cascades Slopes and Foothills	0.0300	Jul 12 2010	0.015	low	oxic	820	66	27.4%	72.1%	0.5%
CAMP-TV-02[2]	Eastern Cascades Slopes and Foothills	0.0300	Aug 4 2010	0.086	moderate	oxic	900	na	0.0%	99.1%	0.9%
CAMP-TV-03	Central California Foothills and Coastal Mountains	0.0300	Aug 5 2010	0.083	moderate	oxic	298	0	0.0%	100.0%	0.0%
CAMP-TV-04	Sierra Nevada	0.0150	Aug 16 2010	0.077	moderate	oxic	1,053	88	12.5%	81.0%	6.5%
CAMP-TV-05	Eastern Cascades Slopes and Foothills	0.0300	Aug 16 2010	0.064	moderate	oxic	1,300	91	1.4%	79.5%	19.2%
CAMP-TV-06	Sierra Nevada	0.0150	Aug 25 2010	0.028	moderate	oxic	1,470	116	0.0%	90.8%	9.2%
CAMP-TV-07	Eastern Cascades Slopes and Foothills	0.0300	Aug 26 2010	0.038	moderate	oxic	1,909	70	0.0%	100.0%	0.0%
CAMP-TV-08	Northern Basin and Range	0.0550	Sep 1 2010	0.038	low	oxic	1,577	61	0.0%	99.5%	0.5%
CAMP-TV-09	Sierra Nevada	0.0150	Sep 2 2010	0.055	moderate	oxic	1,391	123	0.0%	89.7%	10.3%
CAMP-TV-10[2]	Sierra Nevada	0.0150	Sep 2 2010	0.022	moderate	oxic	1,387	na	0.0%	72.4%	27.6%
CAMP-TV-11	Eastern Cascades Slopes and Foothills	0.0300	Sep 15 2010	0.019	low	oxic	1,148	62	0.0%	100.0%	0.0%
CAMP-TV-12	Sierra Nevada	0.0150	Sep 15 2010	0.044	moderate	oxic	1,317	64	0.0%	98.6%	1.4%
CAMP-TV-13	Sierra Nevada	0.0150	Sep 16 2010	0.032	moderate	oxic	1,286	41	0.0%	100.0%	0.0%
CAMP-TV-14	Eastern Cascades Slopes and Foothills	0.0300	Sep 21 2010	0.013	low	oxic	1,001	53	0.0%	94.0%	6.0%
CAMP-TV-15	Eastern Cascades Slopes and Foothills	0.0300	Oct 13 2010	0.011	low	oxic	693	84	0.0%	99.5%	0.5%
CGOLD-01	Central California Foothills and Coastal Mountains	0.0300	May 8 2006	0.009	low	oxic	233	307	0.0%	100.0%	0.0%
CGOLD-02	Central California Foothills and Coastal Mountains	0.0300	May 9 2006	0.006	low	anoxic	678	229	0.0%	84.9%	15.1%
CGOLD-03	Central California Foothills and Coastal Mountains	0.0300	May 9 2006	0.052	moderate	anoxic	579	207	0.0%	100.0%	0.0%
CGOLD-04	Central California Foothills and Coastal Mountains	0.0300	May 10 2006	0.013	low	oxic	702	152	0.0%	100.0%	0.0%
CGOLD-05	Central California Foothills and Coastal Mountains	0.0300	May 12 2006	0.006	low	oxic	671	43	0.0%	77.8%	22.2%
CGOLD-06	Sierra Nevada	0.0150	May 15 2006	0.011	low	oxic	988	115	0.0%	87.8%	12.2%
CGOLD-07	Sierra Nevada	0.0150	May 16 2006	0.006	low	anoxic	1,341	141	0.0%	100.0%	0.0%
CGOLD-08	Sierra Nevada	0.0150	May 16 2006	0.020	moderate	oxic	705	69	0.0%	41.6%	58.4%
CGOLD-09	Central California Foothills and Coastal Mountains	0.0300	May 17 2006	0.023	low	oxic	202	313	0.0%	100.0%	0.0%
CGOLD-10	Central California Foothills and Coastal Mountains	0.0300	May 18 2006	0.011	low	oxic	1,123	312	0.0%	100.0%	0.0%
CGOLD-11[2]	Central California Foothills and Coastal Mountains	0.0300	May 18 2006	0.003	low	anoxic	671	na	0.0%	89.9%	10.1%
CGOLD-12	Central California Foothills and Coastal Mountains	0.0300	May 23 2006	0.040	moderate	anoxic	397	268	0.0%	80.9%	19.1%
CGOLD-13	Central California Foothills and Coastal Mountains	0.0300	May 23 2006	0.006	low	oxic	333	92	0.0%	97.2%	2.8%

Online resource (suppplementary table) 2. Selected attributes of GAMA-PBP (https://ca.water.usgs.gov/gama/) status wells sampled for orthophosphate concentration-page 81.

GAMA-PBP ID	Agricultural land use in 1982[9] (percent)	Natural land use in 1982[9] (percent)	Urban land use in 1982[9] (percent)	Agricultural land use in 1992[9] (percent)	Natural land use in 1992[9] (percent)	Urban land use in 1992[9] (percent)	Agricultural land use in 2002[9] (percent)	Natural land use in 2002[9] (percent)	Urban land use in 2002[9] (percent)	Agricultural land use in 2012[9] (percent)	Natural land use in 2012[9] (percent)	Urban land use in 2012[9] (percent)	Age Classification[10]	Septic Tanks[11]	Aridity[12]
CAMP-SH-14	0.0%	95.9%	4.1%	0.0%	95.9%	4.1%	0.0%	95.5%	4.5%	0.0%	95.5%	4.5%	Modern	1.14	1.016
CAMP-SH-15	99.5%	0.0%	0.5%	99.5%	0.0%	0.5%	99.5%	0.0%	0.5%	99.5%	0.0%	0.5%	Modern	3.29	0.492
CAMP-TV-01	27.4%	72.1%	0.5%	27.4%	72.1%	0.5%	32.0%	67.6%	0.5%	32.0%	67.6%	0.5%	Mixed	0.56	0.515
CAMP-TV-02[2]	0.0%	99.1%	0.9%	0.0%	99.1%	0.9%	0.0%	99.1%	0.9%	0.0%	99.1%	0.9%	Mixed	0.27	0.669
CAMP-TV-03	0.0%	100.0%	0.0%	0.0%	100.0%	0.0%	0.0%	99.5%	0.5%	0.0%	99.5%	0.5%	Mixed	1.83	0.678
CAMP-TV-04	12.5%	81.0%	6.5%	12.5%	80.6%	6.9%	12.0%	80.6%	7.4%	12.0%	80.6%	7.4%	Premodern	0.31	0.825
CAMP-TV-05	1.4%	79.5%	19.2%	1.4%	79.5%	19.2%	1.4%	79.5%	19.2%	1.4%	79.5%	19.2%	Premodern	0.09	0.438
CAMP-TV-06	0.0%	90.8%	9.2%	0.0%	90.8%	9.2%	0.0%	90.8%	9.2%	0.0%	90.8%	9.2%	Mixed	10.79	0.895
CAMP-TV-07	0.0%	100.0%	0.0%	0.0%	100.0%	0.0%	0.0%	100.0%	0.0%	0.0%	100.0%	0.0%	Premodern	0.04	0.626
CAMP-TV-08	0.0%	99.5%	0.5%	0.0%	99.5%	0.5%	0.0%	99.5%	0.5%	0.0%	99.5%	0.5%	Mixed	1.35	0.433
CAMP-TV-09	0.0%	15.0%	85.0%	0.0%	14.6%	85.4%	0.0%	14.6%	85.4%	0.0%	14.6%	85.4%	Premodern	18.41	0.887
CAMP-TV-10[2]	0.0%	72.4%	27.6%	0.0%	72.4%	27.6%	0.0%	72.4%	27.6%	0.0%	72.4%	27.6%	Modern	5.66	0.953
CAMP-TV-11	0.0%	100.0%	0.0%	0.0%	100.0%	0.0%	0.0%	100.0%	0.0%	0.0%	100.0%	0.0%	Modern	1.46	1.600
CAMP-TV-12	0.0%	98.6%	1.4%	0.0%	98.6%	1.4%	0.0%	98.6%	1.4%	0.0%	98.6%	1.4%	ModernOrMixed	1.16	1.794
CAMP-TV-13	0.0%	100.0%	0.0%	0.0%	100.0%	0.0%	0.0%	100.0%	0.0%	0.0%	100.0%	0.0%	Modern	1.16	1.726
CAMP-TV-14	0.0%	94.0%	6.0%	0.0%	93.5%	6.5%	0.0%	93.5%	6.5%	0.0%	93.5%	6.5%	Modern	1.25	1.606
CAMP-TV-15	0.0%	99.5%	0.5%	0.0%	99.5%	0.5%	0.0%	99.5%	0.5%	0.0%	99.5%	0.5%	Mixed	0.53	0.434
CGOLD-01	0.0%	100.0%	0.0%	0.0%	100.0%	0.0%	0.0%	100.0%	0.0%	0.0%	100.0%	0.0%	Mixed	0.35	0.286
CGOLD-02	0.0%	84.9%	15.1%	0.0%	84.9%	15.1%	0.0%	84.9%	15.1%	0.0%	84.9%	15.1%	Mixed	4.97	0.540
CGOLD-03	0.0%	100.0%	0.0%	0.0%	100.0%	0.0%	0.0%	100.0%	0.0%	0.0%	100.0%	0.0%	Premodern	3.48	0.480
CGOLD-04	0.0%	100.0%	0.0%	0.0%	25.9%	74.1%	0.0%	25.9%	74.1%	0.0%	25.9%	74.1%	Modern	8.62	0.549
CGOLD-05	0.0%	77.8%	22.2%	0.0%	0.0%	100.0%	0.0%	0.0%	100.0%	0.0%	0.0%	100.0%	Mixed	14.09	0.513
CGOLD-06	0.0%	86.9%	13.1%	0.0%	38.0%	62.0%	0.0%	38.0%	62.0%	0.0%	38.0%	62.0%	Modern	7.86	0.670
CGOLD-07	0.0%	100.0%	0.0%	0.0%	100.0%	0.0%	0.0%	100.0%	0.0%	0.0%	100.0%	0.0%	Premodern	3.99	0.924
CGOLD-08	0.0%	0.0%	100.0%	0.0%	0.0%	100.0%	0.0%	0.0%	100.0%	0.0%	0.0%	100.0%	Modern	26.56	0.555
CGOLD-09	0.0%	100.0%	0.0%	0.0%	100.0%	0.0%	0.0%	100.0%	0.0%	0.0%	100.0%	0.0%	Mixed	0.35	0.257
CGOLD-10	0.0%	100.0%	0.0%	0.0%	100.0%	0.0%	0.0%	100.0%	0.0%	0.0%	100.0%	0.0%	Modern	3.48	0.772
CGOLD-11[2]	0.0%	89.0%	11.0%	0.0%	89.0%	11.0%	0.0%	89.0%	11.0%	0.0%	89.0%	11.0%	Modern	3.48	0.585
CGOLD-12	0.0%	80.0%	20.0%	0.0%	14.4%	85.6%	0.0%	14.4%	85.6%	0.0%	14.4%	85.6%	Premodern	10.88	0.372
CGOLD-13	0.0%	97.2%	2.8%	0.0%	18.1%	81.9%	0.0%	18.1%	81.9%	0.0%	18.1%	81.9%	Modern	9.96	0.375

Online resource (suppplementary table) 2. Selected attributes of GAMA-PBP (https://ca.water.usgs.gov/gama/) status wells sampled for orthophosphate concentration-page 82.

GAMA-PBP ID	USGS Station ID[1]	GAMA-PBP study unit	GAMA-PBP study area[3]	Hydrogeologic zone
CGOLD-14[2]	371900119380002	Central Sierra	Coarse Gold watershed	Mountain
CGOLD-15	371500119430001	Central Sierra	Coarse Gold watershed	Mountain
CGOLD-16	372500119360001	Central Sierra	Coarse Gold watershed	Mountain
CGOLD-17	371500119470001	Central Sierra	Coarse Gold watershed	Mountain
CGOLD-18	372300119370001	Central Sierra	Coarse Gold watershed	Mountain
CWISH-01	371600119320001	Central Sierra	Wishon watershed	Mountain
CWISH-02	371300119300001	Central Sierra	Wishon watershed	Mountain
CWISH-03	371800119320001	Central Sierra	Wishon watershed	Mountain
CWISH-04	371900119340001	Central Sierra	Wishon watershed	Mountain
CWISH-05	371700119310001	Central Sierra	Wishon watershed	Mountain
CWISH-06[2]	371100119270001	Central Sierra	Wishon watershed	Mountain
CWISH-07	371300119280001	Central Sierra	Wishon watershed	Mountain
CWISH-08	372000119290001	Central Sierra	Wishon watershed	Mountain
CWISH-09	372000119280001	Central Sierra	Wishon watershed	Mountain
KLAM-01	415100123530001	Klamath Mountains	Klamath Mountains	Mountain
KLAM-02	413900123040001	Klamath Mountains	Klamath Mountains	Mountain
KLAM-03	414900122390001	Klamath Mountains	Klamath Mountains	Mountain
KLAM-04	414000122470001	Klamath Mountains	Klamath Mountains	Mountain
KLAM-05	414000122490001	Klamath Mountains	Klamath Mountains	Mountain
KLAM-06	415300122330001	Klamath Mountains	Klamath Mountains	Mountain
KLAM-07[2]	411700123070001	Klamath Mountains	Klamath Mountains	Mountain
KLAM-08	415800123440001	Klamath Mountains	Klamath Mountains	Mountain
KLAM-09	405200123320001	Klamath Mountains	Klamath Mountains	Mountain
KLAM-10[2]	413400123320001	Klamath Mountains	Klamath Mountains	Mountain
KLAM-11	412200123270001	Klamath Mountains	Klamath Mountains	Mountain
KLAM-12	405300122450001	Klamath Mountains	Klamath Mountains	Mountain
KLAM-13	404500123050001	Klamath Mountains	Klamath Mountains	Mountain
KLAM-14[2]	404400123150001	Klamath Mountains	Klamath Mountains	Mountain
KLAM-15[2]	404600123200001	Klamath Mountains	Klamath Mountains	Mountain

Online resource (suppplementary table) 2. Selected attributes of GAMA-PBP (https://ca.water.usgs.gov/gama/) status wells sampled for orthophosphate concentration-page 83.

GAMA-PBP ID	USEPA Level III Ecoregions[4]	Level III Ecoregion Reference Concentration[4]	Status Sample Date	Status Sample Orthophosphate Concentration (mg/L as P)	Relative Concentration Category[5]	Redox state[6]	Elevation of LSD (meters above NAVD 88)[7]	Well depth (meters below LSD)[8]	Agricultural land use in 1974[9] (percent)	Natural land use in 1974[9] (percent)	Urban land use in 1974[9] (percent)
CGOLD-14[2]	Sierra Nevada	0.0150	May 23 2006	0.014	low	oxic	696	na	0.0%	2.3%	97.7%
CGOLD-15	Central California Foothills and Coastal Mountains	0.0300	May 23 2006	1.042	high	anoxic	671	108	0.0%	96.8%	3.2%
CGOLD-16	Sierra Nevada	0.0150	May 24 2006	0.044	moderate	oxic	1,464	152	0.0%	100.0%	0.0%
CGOLD-17	Central California Foothills and Coastal Mountains	0.0300	May 25 2006	0.014	low	oxic	367	91	0.0%	100.0%	0.0%
CGOLD-18	Sierra Nevada	0.0150	May 31 2006	0.009	low	oxic	924	192	0.0%	95.0%	5.0%
CWISH-01	Sierra Nevada	0.0150	May 8 2006	0.008	low	anoxic	1,023	185	0.0%	93.6%	6.4%
CWISH-02	Sierra Nevada	0.0150	May 11 2006	0.006	low	anoxic	812	158	0.0%	76.5%	23.5%
CWISH-03	Sierra Nevada	0.0150	May 11 2006	0.003	low	anoxic	1,068	144	0.0%	98.2%	1.8%
CWISH-04	Sierra Nevada	0.0150	May 15 2006	0.023	moderate	oxic	1,037	213	0.0%	91.8%	8.2%
CWISH-05	Sierra Nevada	0.0150	May 17 2006	0.019	moderate	oxic	1,062	62	0.0%	98.6%	1.4%
CWISH-06[2]	Sierra Nevada	0.0150	May 22 2006	0.006	low	na	550	274	0.0%	99.1%	0.9%
CWISH-07	Sierra Nevada	0.0150	May 22 2006	0.006	low	oxic	885	274	0.0%	99.1%	0.9%
CWISH-08	Sierra Nevada	0.0150	May 25 2006	0.019	moderate	oxic	1,647	282	0.0%	100.0%	0.0%
CWISH-09	Sierra Nevada	0.0150	May 25 2006	0.019	moderate	oxic	1,644	213	0.0%	100.0%	0.0%
KLAM-01	Klamath Mountains/California High North Coast Range	0.0325	Oct 18 2010	0.006	low	oxic	220	0	0.0%	91.7%	8.3%
KLAM-02	Klamath Mountains/California High North Coast Range	0.0325	Oct 19 2010	0.011	low	oxic	928	0	0.0%	100.0%	0.0%
KLAM-03	Klamath Mountains/California High North Coast Range	0.0325	Oct 19 2010	0.025	low	oxic	603	12	0.0%	91.3%	8.7%
KLAM-04	Klamath Mountains/California High North Coast Range	0.0325	Oct 20 2010	0.014	low	oxic	1,446	0	0.0%	100.0%	0.0%
KLAM-05	Klamath Mountains/California High North Coast Range	0.0325	Oct 20 2010	0.036	moderate	oxic	911	24	0.0%	98.6%	1.4%
KLAM-06	Klamath Mountains/California High North Coast Range	0.0325	Oct 21 2010	0.010	low	anoxic	708	82	0.0%	79.7%	20.3%
KLAM-07[2]	Klamath Mountains/California High North Coast Range	0.0325	Oct 21 2010	0.018	low	oxic	681	na	0.0%	98.6%	1.4%
KLAM-08	Klamath Mountains/California High North Coast Range	0.0325	Nov 1 2010	0.007	low	oxic	656	122	0.0%	92.2%	7.8%
KLAM-09	Klamath Mountains/California High North Coast Range	0.0325	Nov 1 2010	0.012	low	oxic	217	15	0.0%	99.5%	0.5%
KLAM-10[2]	Klamath Mountains/California High North Coast Range	0.0325	Nov 2 2010	0.035	moderate	na	324	0	0.0%	100.0%	0.0%
KLAM-11	Klamath Mountains/California High North Coast Range	0.0325	Nov 2 2010	0.016	low	oxic	200	27	0.0%	99.1%	0.9%
KLAM-12	Klamath Mountains/California High North Coast Range	0.0325	Nov 3 2010	0.022	low	oxic	809	0	0.0%	98.6%	1.4%
KLAM-13	Klamath Mountains/California High North Coast Range	0.0325	Nov 3 2010	0.018	low	oxic	430	14	0.0%	91.6%	8.4%
KLAM-14[2]	Klamath Mountains/California High North Coast Range	0.0325	Nov 4 2010	0.037	moderate	oxic	387	na	0.0%	89.8%	10.2%
KLAM-15[2]	Klamath Mountains/California High North Coast Range	0.0325	Nov 4 2010	0.015	low	oxic	347	na	0.0%	93.2%	6.8%

Online resource (suppplementary table) 2. Selected attributes of GAMA-PBP (https://ca.water.usgs.gov/gama/) status wells sampled for orthophosphate concentration-page 84.

GAMA-PBP ID	Agricultural land use in 1982[9] (percent)	Natural land use in 1982[9] (percent)	Urban land use in 1982[9] (percent)	Agricultural land use in 1992[9] (percent)	Natural land use in 1992[9] (percent)	Urban land use in 1992[9] (percent)	Agricultural land use in 2002[9] (percent)	Natural land use in 2002[9] (percent)	Urban land use in 2002[9] (percent)	Agricultural land use in 2012[9] (percent)	Natural land use in 2012[9] (percent)	Urban land use in 2012[9] (percent)	Age Classification[10]	Septic Tanks[11]	Aridity[12]
CGOLD-14[2]	0.0%	2.3%	97.7%	0.0%	2.3%	97.7%	0.0%	2.3%	97.7%	0.0%	2.3%	97.7%	Mixed	39.18	0.552
CGOLD-15	0.0%	96.8%	3.2%	0.0%	96.8%	3.2%	0.0%	96.8%	3.2%	0.0%	96.8%	3.2%	Modern	3.48	0.534
CGOLD-16	0.0%	100.0%	0.0%	0.0%	100.0%	0.0%	0.0%	100.0%	0.0%	0.0%	100.0%	0.0%	ModernOrMixed	4.06	0.872
CGOLD-17	0.0%	100.0%	0.0%	0.0%	100.0%	0.0%	0.0%	100.0%	0.0%	0.0%	100.0%	0.0%	Modern	0.65	0.397
CGOLD-18	0.0%	94.5%	5.5%	0.0%	94.5%	5.5%	0.0%	94.5%	5.5%	0.0%	94.5%	5.5%	Mixed	7.28	0.723
CWISH-01	0.0%	93.6%	6.4%	0.0%	93.6%	6.4%	0.0%	93.1%	6.9%	0.0%	93.1%	6.9%	Mixed	7.46	0.731
CWISH-02	0.0%	76.5%	23.5%	0.0%	76.0%	24.0%	0.0%	76.0%	24.0%	0.0%	76.0%	24.0%	Premodern	2.60	0.644
CWISH-03	0.0%	98.2%	1.8%	0.0%	98.2%	1.8%	0.0%	98.2%	1.8%	0.0%	98.2%	1.8%	Premodern	1.05	0.777
CWISH-04	0.0%	85.5%	14.5%	0.0%	80.5%	19.5%	0.0%	79.1%	20.9%	0.0%	79.1%	20.9%	Modern	9.28	0.757
CWISH-05	0.0%	98.6%	1.4%	0.0%	98.6%	1.4%	0.0%	98.6%	1.4%	0.0%	98.6%	1.4%	Modern	2.19	0.722
CWISH-06[2]	0.0%	99.1%	0.9%	0.0%	99.1%	0.9%	0.0%	99.1%	0.9%	0.0%	99.1%	0.9%	Premodern	2.60	0.611
CWISH-07	0.0%	99.1%	0.9%	0.0%	99.1%	0.9%	0.0%	99.1%	0.9%	0.0%	99.1%	0.9%	Mixed	2.60	0.646
CWISH-08	0.0%	100.0%	0.0%	0.0%	100.0%	0.0%	0.0%	100.0%	0.0%	0.0%	100.0%	0.0%	Modern	0.00	1.186
CWISH-09	0.0%	100.0%	0.0%	0.0%	100.0%	0.0%	0.0%	100.0%	0.0%	0.0%	100.0%	0.0%	Modern	0.00	1.238
KLAM-01	0.0%	91.2%	8.8%	0.0%	91.2%	8.8%	0.0%	91.2%	8.8%	0.0%	91.2%	8.8%	Modern	0.27	2.433
KLAM-02	0.0%	100.0%	0.0%	0.0%	100.0%	0.0%	0.0%	100.0%	0.0%	0.0%	100.0%	0.0%	Modern	0.55	0.911
KLAM-03	0.0%	91.3%	8.7%	0.0%	90.4%	9.6%	0.0%	90.4%	9.6%	0.0%	90.0%	10.0%	Mixed	0.54	0.474
KLAM-04	0.0%	100.0%	0.0%	0.0%	100.0%	0.0%	0.0%	99.5%	0.5%	0.0%	99.5%	0.5%	Modern	0.60	0.862
KLAM-05	0.0%	98.6%	1.4%	0.0%	98.6%	1.4%	0.0%	98.6%	1.4%	0.0%	98.6%	1.4%	Modern	0.62	0.597
KLAM-06	0.0%	79.7%	20.3%	0.0%	79.3%	20.7%	0.5%	78.8%	20.7%	0.5%	78.8%	20.7%	Mixed	0.52	0.433
KLAM-07[2]	0.0%	98.6%	1.4%	0.0%	98.6%	1.4%	0.0%	98.6%	1.4%	0.0%	98.6%	1.4%	Modern	0.18	1.068
KLAM-08	0.0%	92.2%	7.8%	0.0%	91.7%	8.3%	0.0%	90.8%	9.2%	0.0%	90.8%	9.2%	Mixed	0.27	2.182
KLAM-09	0.0%	99.5%	0.5%	0.0%	99.5%	0.5%	0.0%	99.5%	0.5%	0.0%	99.5%	0.5%	Mixed	3.83	1.476
KLAM-10[2]	0.0%	100.0%	0.0%	0.0%	100.0%	0.0%	0.0%	100.0%	0.0%	0.0%	100.0%	0.0%	Modern	0.16	2.031
KLAM-11	0.0%	99.1%	0.9%	0.0%	99.1%	0.9%	0.0%	99.1%	0.9%	0.0%	99.1%	0.9%	Modern	0.16	1.490
KLAM-12	0.0%	98.6%	1.4%	0.0%	98.6%	1.4%	0.0%	98.6%	1.4%	0.0%	98.6%	1.4%	Modern	0.34	1.020
KLAM-13	0.0%	91.1%	8.9%	0.0%	90.2%	9.8%	0.0%	90.2%	9.8%	0.0%	90.2%	9.8%	Modern	0.37	0.922
KLAM-14[2]	0.5%	89.4%	10.2%	0.5%	88.9%	10.6%	0.0%	88.9%	11.1%	0.0%	88.9%	11.1%	Modern	0.44	1.060
KLAM-15[2]	0.0%	92.7%	7.3%	0.0%	92.3%	7.7%	0.0%	91.8%	8.2%	0.0%	91.8%	8.2%	Modern	0.44	1.331

Online resource (suppplementary table) 2. Selected attributes of GAMA-PBP (https://ca.water.usgs.gov/gama/) status wells sampled for orthophosphate concentration-page 85.

GAMA-PBP ID	USGS Station ID[1]	GAMA-PBP study unit	GAMA-PBP study area[3]	Hydrogeologic zone
KLAM-16	405300122220001	Klamath Mountains	Klamath Mountains	Mountain
KLAM-17[2]	412100122540001	Klamath Mountains	Klamath Mountains	Mountain
KLAM-18	405400122470001	Klamath Mountains	Klamath Mountains	Mountain
KLAM-19	404400123010001	Klamath Mountains	Klamath Mountains	Mountain
KLAM-20	405100122450001	Klamath Mountains	Klamath Mountains	Mountain
KLAM-21	403900122470001	Klamath Mountains	Klamath Mountains	Mountain
KLAM-22	405600123370001	Klamath Mountains	Klamath Mountains	Mountain
KLAM-23	404200122510001	Klamath Mountains	Klamath Mountains	Mountain
KLAM-24[2]	411700122480001	Klamath Mountains	Klamath Mountains	Mountain
KLAM-25	404600122200001	Klamath Mountains	Klamath Mountains	Mountain
KLAM-26	403000122320001	Klamath Mountains	Klamath Mountains	Mountain
KLAM-27	404700122180001	Klamath Mountains	Klamath Mountains	Mountain
KLAM-28	404900122190001	Klamath Mountains	Klamath Mountains	Mountain
KLAM-29	411400122150001	Klamath Mountains	Klamath Mountains	Mountain
KLAM-30	410800122180001	Klamath Mountains	Klamath Mountains	Mountain
KLAM-31[2]	412500122300001	Klamath Mountains	Klamath Mountains	Mountain
KLAM-32	405200122150001	Klamath Mountains	Klamath Mountains	Mountain
KLAM-33	405500122240001	Klamath Mountains	Klamath Mountains	Mountain
KLAM-34	404257122195401	Klamath Mountains	Klamath Mountains	Mountain
KLAM-35[2]	403800122330001	Klamath Mountains	Klamath Mountains	Mountain
KLAM-36	410500122420001	Klamath Mountains	Klamath Mountains	Mountain
KLAM-37[2]	404400122470001	Klamath Mountains	Klamath Mountains	Mountain
KLAM-38[2]	404700122460001	Klamath Mountains	Klamath Mountains	Mountain
SIERRA-G-01	351500118370001	Sierra Nevada	Sierra Nevada Regional	Mountain
SIERRA-G-03	355200118400001	Sierra Nevada	Sierra Nevada Regional	Mountain
SIERRA-G-04[2]	360700118320001	Sierra Nevada	Sierra Nevada Regional	Mountain
SIERRA-G-05	362600118540001	Sierra Nevada	Sierra Nevada Regional	Mountain
SIERRA-G-06	372700119430001	Sierra Nevada	Sierra Nevada Regional	Mountain
SIERRA-G-08	370600119190001	Sierra Nevada	Sierra Nevada Regional	Mountain
SIERRA-G-09	364100118520001	Sierra Nevada	Sierra Nevada Regional	Mountain

Online resource (suppplementary table) 2. Selected attributes of GAMA-PBP (https://ca.water.usgs.gov/gama/) status wells sampled for orthophosphate concentration-page 86.

GAMA-PBP ID	USEPA Level III Ecoregions[4]	Level III Ecoregion Reference Concentration[4]	Status Sample Date	Status Sample Orthophosphate Concentration (mg/L as P)	Relative Concentration Category[5]	Redox state[6]	Elevation of LSD (meters above NAVD 88)[7]	Well depth (meters below LSD)[8]	Agricultural land use in 1974[9] (percent)	Natural land use in 1974[9] (percent)	Urban land use in 1974[9] (percent)
KLAM-16	Klamath Mountains/California High North Coast Range	0.0325	Nov 15 2010	0.024	low	oxic	341	85	5.0%	94.1%	0.9%
KLAM-17[2]	Klamath Mountains/California High North Coast Range	0.0325	Nov 15 2010	0.024	low	oxic	1,012	na	0.0%	99.1%	0.9%
KLAM-18	Klamath Mountains/California High North Coast Range	0.0325	Nov 16 2010	0.017	low	oxic	1,006	98	0.0%	98.6%	1.4%
KLAM-19	Klamath Mountains/California High North Coast Range	0.0325	Nov 16 2010	0.029	low	oxic	545	24	0.0%	91.6%	8.4%
KLAM-20	Klamath Mountains/California High North Coast Range	0.0325	Nov 17 2010	0.026	low	oxic	751	0	0.0%	98.2%	1.8%
KLAM-21	Klamath Mountains/California High North Coast Range	0.0325	Nov 17 2010	0.022	low	oxic	799	69	0.0%	98.6%	1.4%
KLAM-22	Klamath Mountains/California High North Coast Range	0.0325	Nov 18 2010	0.017	low	oxic	139	13	0.0%	78.1%	21.9%
KLAM-23	Klamath Mountains/California High North Coast Range	0.0325	Nov 18 2010	0.028	low	oxic	547	93	0.0%	85.7%	14.3%
KLAM-24[2]	Klamath Mountains/California High North Coast Range	0.0325	Nov 29 2010	0.004	low	anoxic	1,047	na	0.0%	97.7%	2.3%
KLAM-25	Central California Foothills and Coastal Mountains	0.0300	Nov 30 2010	0.029	low	oxic	331	38	0.0%	100.0%	0.0%
KLAM-26	Central California Foothills and Coastal Mountains	0.0300	Nov 30 2010	0.035	moderate	oxic	333	27	35.3%	59.1%	5.6%
KLAM-27	Central California Foothills and Coastal Mountains	0.0300	Dec 1 2010	0.026	low	oxic	348	98	0.0%	99.5%	0.5%
KLAM-28	Klamath Mountains/California High North Coast Range	0.0325	Dec 1 2010	0.015	low	oxic	333	34	0.0%	96.3%	3.7%
KLAM-29	Klamath Mountains/California High North Coast Range	0.0325	Dec 2 2010	0.075	moderate	oxic	820	0	0.0%	59.8%	40.2%
KLAM-30	Klamath Mountains/California High North Coast Range	0.0325	Dec 2 2010	0.025	low	anoxic	612	37	0.0%	88.0%	12.0%
KLAM-31[2]	Klamath Mountains/California High North Coast Range	0.0325	Dec 6 2010	0.010	low	oxic	1,210	na	0.0%	100.0%	0.0%
KLAM-32	Klamath Mountains/California High North Coast Range	0.0325	Dec 7 2010	0.030	low	oxic	432	69	0.0%	99.5%	0.5%
KLAM-33	Klamath Mountains/California High North Coast Range	0.0325	Dec 7 2010	0.073	moderate	oxic	381	76	0.0%	91.3%	8.7%
KLAM-34	Central California Foothills and Coastal Mountains	0.0300	Dec 8 2010	0.020	low	oxic	257	54	1.9%	63.4%	34.7%
KLAM-35[2]	Central California Foothills and Coastal Mountains	0.0300	Dec 8 2010	0.016	low	oxic	406	na	0.0%	99.5%	0.5%
KLAM-36	Klamath Mountains/California High North Coast Range	0.0325	Dec 9 2010	0.011	low	oxic	755	8	0.0%	85.8%	14.2%
KLAM-37[2]	Klamath Mountains/California High North Coast Range	0.0325	Dec 13 2010	0.039	moderate	oxic	587	na	0.0%	99.1%	0.9%
KLAM-38[2]	Klamath Mountains/California High North Coast Range	0.0325	Dec 14 2010	0.012	low	oxic	600	na	0.0%	98.6%	1.4%
SIERRA-G-01	Southern California Mountains	0.0109	Jun 23 2008	0.014	moderate	anoxic	990	183	0.0%	100.0%	0.0%
SIERRA-G-03	Sierra Nevada	0.0150	Jul 9 2008	0.064	moderate	anoxic	910	91	0.0%	100.0%	0.0%
SIERRA-G-04[2]	Sierra Nevada	0.0150	Jul 10 2008	0.028	moderate	na	2,316	0	0.0%	100.0%	0.0%
SIERRA-G-05	Central California Foothills and Coastal Mountains	0.0300	Jul 15 2008	0.146	high	oxic	251	37	0.0%	92.2%	7.8%
SIERRA-G-06	Sierra Nevada	0.0150	Jul 21 2008	0.015	moderate	anoxic	946	274	0.0%	100.0%	0.0%
SIERRA-G-08	Sierra Nevada	0.0150	Jul 23 2008	0.031	moderate	oxic	1,709	37	0.0%	61.8%	38.2%
SIERRA-G-09	Sierra Nevada	0.0150	Jul 24 2008	0.036	moderate	oxic	2,274	0	0.0%	93.9%	6.1%

Online resource (suppplementary table) 2. Selected attributes of GAMA-PBP (https://ca.water.usgs.gov/gama/) status wells sampled for orthophosphate concentration-page 87.

GAMA-PBP ID	Agricultural land use in 1982[9] (percent)	Natural land use in 1982[9] (percent)	Urban land use in 1982[9] (percent)	Agricultural land use in 1992[9] (percent)	Natural land use in 1992[9] (percent)	Urban land use in 1992[9] (percent)	Agricultural land use in 2002[9] (percent)	Natural land use in 2002[9] (percent)	Urban land use in 2002[9] (percent)	Agricultural land use in 2012[9] (percent)	Natural land use in 2012[9] (percent)	Urban land use in 2012[9] (percent)	Age Classification[10]	Septic Tanks[11]	Aridity[12]
KLAM-16	5.0%	94.1%	0.9%	5.0%	94.1%	0.9%	15.1%	84.0%	0.9%	15.1%	84.0%	0.9%	Modern	1.47	1.558
KLAM-17[2]	0.0%	99.1%	0.9%	0.0%	99.1%	0.9%	0.0%	98.6%	1.4%	0.0%	98.6%	1.4%	Modern	0.18	0.588
KLAM-18	0.0%	98.6%	1.4%	0.0%	98.6%	1.4%	0.0%	97.7%	2.3%	0.0%	97.7%	2.3%	Modern	0.34	1.136
KLAM-19	0.0%	91.6%	8.4%	0.0%	91.2%	8.8%	0.0%	90.7%	9.3%	0.0%	90.2%	9.8%	Modern	0.37	0.901
KLAM-20	0.0%	98.2%	1.8%	0.0%	98.2%	1.8%	0.0%	98.2%	1.8%	0.0%	98.2%	1.8%	Modern	0.34	0.919
KLAM-21	0.0%	98.6%	1.4%	0.0%	98.6%	1.4%	0.0%	98.6%	1.4%	0.0%	98.6%	1.4%	Modern	0.52	1.044
KLAM-22	0.0%	78.1%	21.9%	0.0%	77.7%	22.3%	0.0%	76.3%	23.7%	0.0%	74.4%	25.6%	Modern	0.57	1.482
KLAM-23	0.0%	85.7%	14.3%	0.0%	85.7%	14.3%	0.0%	85.3%	14.7%	0.0%	85.3%	14.7%	Modern	1.86	0.803
KLAM-24[2]	0.0%	97.7%	2.3%	0.0%	97.7%	2.3%	0.0%	96.8%	3.2%	0.0%	96.8%	3.2%	Mixed	0.18	0.710
KLAM-25	0.0%	100.0%	0.0%	0.0%	100.0%	0.0%	0.0%	100.0%	0.0%	0.0%	100.0%	0.0%	Mixed	1.49	1.390
KLAM-26	35.3%	59.1%	5.6%	35.3%	59.1%	5.6%	35.3%	59.1%	5.6%	35.3%	59.1%	5.6%	Modern	1.57	0.959
KLAM-27	0.0%	99.5%	0.5%	0.0%	99.5%	0.5%	0.0%	99.5%	0.5%	0.0%	99.5%	0.5%	Modern	0.70	1.491
KLAM-28	0.0%	96.3%	3.7%	0.0%	95.9%	4.1%	0.0%	95.9%	4.1%	0.0%	95.9%	4.1%	Modern	1.49	1.730
KLAM-29	0.0%	59.8%	40.2%	0.0%	59.8%	40.2%	0.0%	59.8%	40.2%	0.0%	59.8%	40.2%	Mixed	1.07	1.559
KLAM-30	0.0%	88.0%	12.0%	0.0%	88.0%	12.0%	0.0%	88.0%	12.0%	0.0%	88.0%	12.0%	Mixed	0.47	1.594
KLAM-31[2]	0.0%	100.0%	0.0%	0.0%	100.0%	0.0%	0.0%	100.0%	0.0%	0.0%	100.0%	0.0%	Mixed	1.13	1.310
KLAM-32	0.0%	99.5%	0.5%	0.0%	99.5%	0.5%	0.0%	99.5%	0.5%	0.0%	99.5%	0.5%	Mixed	0.69	1.542
KLAM-33	0.0%	91.3%	8.7%	0.0%	90.8%	9.2%	0.0%	90.8%	9.2%	0.0%	90.8%	9.2%	Mixed	1.46	1.672
KLAM-34	1.9%	62.4%	35.7%	1.9%	17.8%	80.3%	11.7%	17.4%	70.9%	11.7%	16.4%	71.8%	Mixed	10.85	1.280
KLAM-35[2]	0.0%	99.5%	0.5%	0.0%	99.5%	0.5%	0.0%	99.1%	0.9%	0.0%	98.2%	1.8%	Modern	1.16	1.432
KLAM-36	0.0%	85.4%	14.6%	0.0%	85.4%	14.6%	0.0%	85.4%	14.6%	0.0%	84.5%	15.5%	Modern	0.09	1.319
KLAM-37[2]	0.0%	99.1%	0.9%	0.0%	99.1%	0.9%	0.0%	99.1%	0.9%	0.0%	99.1%	0.9%	Modern	1.86	0.807
KLAM-38[2]	0.0%	98.6%	1.4%	0.0%	98.6%	1.4%	0.0%	98.6%	1.4%	0.0%	98.2%	1.8%	Modern	1.86	0.901
SIERRA-G-01	0.0%	100.0%	0.0%	0.0%	100.0%	0.0%	0.0%	100.0%	0.0%	0.0%	100.0%	0.0%	Mixed	4.02	0.279
SIERRA-G-03	0.0%	100.0%	0.0%	0.0%	100.0%	0.0%	0.0%	100.0%	0.0%	0.0%	100.0%	0.0%	Mixed	5.54	0.501
SIERRA-G-04[2]	0.0%	100.0%	0.0%	0.0%	100.0%	0.0%	0.0%	100.0%	0.0%	0.0%	100.0%	0.0%	Modern	1.15	0.929
SIERRA-G-05	0.0%	91.7%	8.3%	0.0%	91.2%	8.8%	0.0%	91.2%	8.8%	0.0%	91.2%	8.8%	Modern	3.56	0.380
SIERRA-G-06	0.0%	100.0%	0.0%	0.0%	100.0%	0.0%	0.0%	100.0%	0.0%	0.0%	100.0%	0.0%	Mixed	3.20	0.755
SIERRA-G-08	0.0%	44.5%	55.5%	0.0%	40.0%	60.0%	0.0%	40.0%	60.0%	0.0%	40.0%	60.0%	Modern	8.39	0.784
SIERRA-G-09	0.0%	93.5%	6.5%	0.0%	93.5%	6.5%	0.0%	93.5%	6.5%	0.0%	93.5%	6.5%	Modern	0.83	0.992

Online resource (suppplementary table) 2. Selected attributes of GAMA-PBP (https://ca.water.usgs.gov/gama/) status wells sampled for orthophosphate concentration-page 88.

GAMA-PBP ID	USGS Station ID[1]	GAMA-PBP study unit	GAMA-PBP study area[3]	Hydrogeologic zone
SIERRA-G-10	381900119450001	Sierra Nevada	Sierra Nevada Regional	Mountain
SIERRA-G-11	380500120110001	Sierra Nevada	Sierra Nevada Regional	Mountain
SIERRA-G-12	375100119560001	Sierra Nevada	Sierra Nevada Regional	Mountain
SIERRA-G-13	383900120360001	Sierra Nevada	Sierra Nevada Regional	Mountain
SIERRA-G-14	384800120050001	Sierra Nevada	Sierra Nevada Regional	Mountain
SIERRA-G-15	385300121150001	Sierra Nevada	Sierra Nevada Regional	Mountain
SIERRA-G-16	363500118140001	Sierra Nevada	Sierra Nevada Regional	Mountain
SIERRA-G-17	371100118330001	Sierra Nevada	Sierra Nevada Regional	Mountain
SIERRA-G-18	372700118440001	Sierra Nevada	Sierra Nevada Regional	Mountain
SIERRA-M-01	375500120220001	Sierra Nevada	Sierra Nevada Regional	Mountain
SIERRA-M-02	382800120500001	Sierra Nevada	Sierra Nevada Regional	Mountain
SIERRA-M-03	401000120560001	Sierra Nevada	Sierra Nevada Regional	Mountain
SIERRA-M-04	394100120400001	Sierra Nevada	Sierra Nevada Regional	Mountain
SIERRA-M-05[2]	391600120560001	Sierra Nevada	Sierra Nevada Regional	Mountain
SIERRA-M-06	393100121110001	Sierra Nevada	Sierra Nevada Regional	Mountain
SIERRA-S-01	353900118170001	Sierra Nevada	Sierra Nevada Regional	Mountain
SIERRA-S-02	385131120021601	Sierra Nevada	Sierra Nevada Regional	Mountain
SIERRA-S-03	394800120300001	Sierra Nevada	Sierra Nevada Regional	Mountain
SIERRA-V-02	394800121350001	Sierra Nevada	Sierra Nevada Regional	Mountain
SIERRA-V-03[2]	373700119050001	Sierra Nevada	Sierra Nevada Regional	Mountain
SOSA-03	353800118230001	Southern Sierra	Sierra Nevada Regional	Mountain
SOSA-07	354700118270001	Southern Sierra	Sierra Nevada Regional	Mountain
SOSA-10	354100118130001	Southern Sierra	Sierra Nevada Regional	Mountain
SOSA-13	350700118270001	Southern Sierra	Sierra Nevada Regional	Mountain
SOSA-15	354300118330001	Southern Sierra	Sierra Nevada Regional	Mountain
SOSA-22[2]	355300118220001	Southern Sierra	Sierra Nevada Regional	Mountain
SOSA-35[2]	351000118200001	Southern Sierra	Sierra Nevada Regional	Mountain
TMART-01	392100120080001	Tahoe-Martis	Martis Valley	Mountain
TMART-02	392000120050001	Tahoe-Martis	Martis Valley	Mountain
TMART-03	392200120040001	Tahoe-Martis	Martis Valley	Mountain

Online resource (suppplementary table) 2. Selected attributes of GAMA-PBP (https://ca.water.usgs.gov/gama/) status wells sampled for orthophosphate concentration-page 89.

GAMA-PBP ID	USEPA Level III Ecoregions[4]	Level III Ecoregion Reference Concentration[4]	Status Sample Date	Status Sample Orthophosphate Concentration (mg/L as P)	Relative Concentration Category[5]	Redox state[6]	Elevation of LSD (meters above NAVD 88)[7]	Well depth (meters below LSD)[8]	Agricultural land use in 1974[9] (percent)	Natural land use in 1974[9] (percent)	Urban land use in 1974[9] (percent)
SIERRA-G-10	Sierra Nevada	0.0150	Jul 28 2008	0.013	low	oxic	1,904	32	0.0%	91.8%	8.2%
SIERRA-G-11	Sierra Nevada	0.0150	Jul 29 2008	0.095	moderate	oxic	1,151	198	0.0%	100.0%	0.0%
SIERRA-G-12	Sierra Nevada	0.0150	Aug 14 2008	0.036	moderate	oxic	1,180	206	0.0%	100.0%	0.0%
SIERRA-G-13	Sierra Nevada	0.0150	Aug 21 2008	0.078	moderate	oxic	842	122	0.0%	100.0%	0.0%
SIERRA-G-14	Sierra Nevada	0.0150	Aug 26 2008	0.024	moderate	oxic	2,059	122	0.0%	100.0%	0.0%
SIERRA-G-15	Central California Foothills and Coastal Mountains	0.0300	Sep 8 2008	0.005	low	anoxic	61	146	8.2%	81.3%	10.5%
SIERRA-G-16	Sierra Nevada	0.0150	Sep 22 2008	0.006	low	oxic	2,620	0	0.0%	100.0%	0.0%
SIERRA-G-17	Sierra Nevada	0.0150	Sep 23 2008	0.019	moderate	oxic	2,987	0	0.0%	100.0%	0.0%
SIERRA-G-18	Sierra Nevada	0.0150	Sep 24 2008	0.008	low	oxic	3,082	0	0.0%	100.0%	0.0%
SIERRA-M-01	Central California Foothills and Coastal Mountains	0.0300	Jul 30 2008	0.012	low	anoxic	464	122	0.0%	100.0%	0.0%
SIERRA-M-02	Central California Foothills and Coastal Mountains	0.0300	Aug 6 2008	0.035	moderate	anoxic	336	134	0.0%	66.1%	33.9%
SIERRA-M-03	Sierra Nevada	0.0150	Sep 10 2008	0.010	low	oxic	1,190	0	0.0%	100.0%	0.0%
SIERRA-M-04	Sierra Nevada	0.0150	Sep 17 2008	0.005	low	oxic	1,958	0	0.0%	100.0%	0.0%
SIERRA-M-05[2]	Sierra Nevada	0.0150	Oct 8 2008	0.027	moderate	anoxic	937	na	0.0%	100.0%	0.0%
SIERRA-M-06	Sierra Nevada	0.0150	Oct 8 2008	0.006	low	oxic	1,024	41	0.0%	90.1%	9.9%
SIERRA-S-01	Southern California Mountains	0.0109	Jun 25 2008	0.046	moderate	oxic	809	37	45.5%	49.3%	5.2%
SIERRA-S-02	Sierra Nevada	0.0150	Aug 19 2008	0.031	moderate	oxic	1,921	101	0.0%	36.7%	63.3%
SIERRA-S-03	Sierra Nevada	0.0150	Oct 20 2008	0.049	moderate	oxic	1,477	158	1.4%	55.0%	43.6%
SIERRA-V-02	Central California Foothills and Coastal Mountains	0.0300	Oct 7 2008	0.079	moderate	oxic	721	214	0.0%	8.2%	91.8%
SIERRA-V-03[2]	Sierra Nevada	0.0150	Oct 21 2008	0.090	moderate	na	2,333	67	0.0%	99.5%	0.5%
SOSA-03	Southern California Mountains	0.0109	Jun 6 2006	0.055	moderate	oxic	795	82	29.5%	30.4%	40.1%
SOSA-07	Southern California Mountains	0.0109	Jun 7 2006	0.062	moderate	anoxic	854	16	0.0%	100.0%	0.0%
SOSA-10	Mojave Basin and Range	0.0100	Jun 8 2006	0.054	moderate	oxic	830	53	0.0%	92.5%	7.5%
SOSA-13	Southern California Mountains	0.0109	Jun 12 2006	0.029	moderate	oxic	1,223	158	27.0%	19.5%	53.5%
SOSA-15	Sierra Nevada	0.0150	Jun 12 2006	0.025	moderate	oxic	1,818	0	0.0%	34.1%	65.9%
SOSA-22[2]	Sierra Nevada	0.0150	Jun 15 2006	0.068	moderate	oxic	2,196	na	0.0%	100.0%	0.0%
SOSA-35[2]	Mojave Basin and Range	0.0100	Jun 30 2006	0.129	high	anoxic	1,299	na	0.5%	98.6%	0.9%
TMART-01	Sierra Nevada	0.0150	Jun 25 2007	0.216	high	oxic	1,782	345	0.0%	84.9%	15.1%
TMART-02	Sierra Nevada	0.0150	Jun 26 2007	0.029	moderate	oxic	1,867	210	0.0%	97.3%	2.7%
TMART-03	Sierra Nevada	0.0150	Jun 26 2007	0.140	high	anoxic	1,706	117	0.0%	95.9%	4.1%

Online resource (suppplementary table) 2. Selected attributes of GAMA-PBP (https://ca.water.usgs.gov/gama/) status wells sampled for orthophosphate concentration-page 90.

GAMA-PBP ID	Agricultural land use in 1982[9] (percent)	Natural land use in 1982[9] (percent)	Urban land use in 1982[9] (percent)	Agricultural land use in 1992[9] (percent)	Natural land use in 1992[9] (percent)	Urban land use in 1992[9] (percent)	Agricultural land use in 2002[9] (percent)	Natural land use in 2002[9] (percent)	Urban land use in 2002[9] (percent)	Agricultural land use in 2012[9] (percent)	Natural land use in 2012[9] (percent)	Urban land use in 2012[9] (percent)	Age Classification[10]	Septic Tanks[11]	Aridity[12]
SIERRA-G-10	0.0%	91.8%	8.2%	0.0%	90.9%	9.1%	0.0%	90.9%	9.1%	0.0%	90.9%	9.1%	Modern	0.03	1.731
SIERRA-G-11	0.0%	100.0%	0.0%	0.0%	100.0%	0.0%	0.0%	100.0%	0.0%	0.0%	100.0%	0.0%	Modern	0.88	0.880
SIERRA-G-12	0.0%	100.0%	0.0%	0.0%	100.0%	0.0%	0.0%	100.0%	0.0%	0.0%	100.0%	0.0%	Modern	0.21	0.765
SIERRA-G-13	0.0%	100.0%	0.0%	0.0%	100.0%	0.0%	0.0%	100.0%	0.0%	0.0%	100.0%	0.0%	Modern	0.88	0.996
SIERRA-G-14	0.0%	100.0%	0.0%	0.0%	100.0%	0.0%	0.0%	100.0%	0.0%	0.0%	100.0%	0.0%	Modern	7.41	1.286
SIERRA-G-15	8.2%	77.6%	14.2%	8.2%	77.6%	14.2%	8.2%	77.6%	14.2%	7.8%	33.3%	58.9%	Mixed	3.64	0.470
SIERRA-G-16	0.0%	100.0%	0.0%	0.0%	100.0%	0.0%	0.0%	100.0%	0.0%	0.0%	100.0%	0.0%	Mixed	0.35	0.550
SIERRA-G-17	0.0%	100.0%	0.0%	0.0%	100.0%	0.0%	0.0%	100.0%	0.0%	0.0%	100.0%	0.0%	Modern	0.54	0.663
SIERRA-G-18	0.0%	100.0%	0.0%	0.0%	100.0%	0.0%	0.0%	100.0%	0.0%	0.0%	100.0%	0.0%	Mixed	0.32	0.752
SIERRA-M-01	0.0%	100.0%	0.0%	0.0%	68.3%	31.7%	0.0%	66.5%	33.5%	0.0%	66.5%	33.5%	Mixed	4.17	0.595
SIERRA-M-02	0.0%	66.1%	33.9%	0.0%	66.1%	33.9%	1.4%	65.6%	33.0%	1.4%	65.6%	33.0%	Mixed	2.71	0.588
SIERRA-M-03	0.0%	100.0%	0.0%	0.0%	100.0%	0.0%	0.0%	100.0%	0.0%	0.0%	100.0%	0.0%	ModernOrMixed	1.52	0.978
SIERRA-M-04	0.0%	100.0%	0.0%	0.0%	100.0%	0.0%	0.0%	100.0%	0.0%	0.0%	100.0%	0.0%	Mixed	5.42	1.598
SIERRA-M-05[2]	0.0%	100.0%	0.0%	0.0%	100.0%	0.0%	0.0%	100.0%	0.0%	0.0%	100.0%	0.0%	Mixed	6.03	1.291
SIERRA-M-06	0.0%	90.1%	9.9%	0.0%	90.1%	9.9%	0.0%	90.1%	9.9%	0.0%	89.7%	10.3%	Modern	1.95	1.563
SIERRA-S-01	46.5%	26.8%	26.8%	45.5%	26.8%	27.7%	45.5%	26.8%	27.7%	45.5%	26.8%	27.7%	Modern	0.50	0.202
SIERRA-S-02	0.0%	34.4%	65.6%	0.0%	34.4%	65.6%	0.0%	33.9%	66.1%	0.0%	33.9%	66.1%	Modern	0.00	0.886
SIERRA-S-03	0.0%	55.0%	45.0%	0.0%	12.4%	87.6%	0.0%	12.4%	87.6%	0.0%	12.4%	87.6%	Modern	1.55	0.507
SIERRA-V-02	0.0%	0.9%	99.1%	0.0%	0.0%	100.0%	0.0%	0.0%	100.0%	0.0%	0.0%	100.0%	Modern	117.37	1.343
SIERRA-V-03[2]	0.0%	99.5%	0.5%	0.0%	99.5%	0.5%	0.0%	99.5%	0.5%	0.0%	99.5%	0.5%	Mixed	0.00	0.601
SOSA-03	29.5%	30.4%	40.1%	0.9%	17.5%	81.6%	0.9%	17.5%	81.6%	0.9%	7.8%	91.2%	Mixed	13.15	0.204
SOSA-07	0.0%	100.0%	0.0%	0.0%	100.0%	0.0%	0.0%	100.0%	0.0%	0.0%	100.0%	0.0%	Modern	0.02	0.261
SOSA-10	0.0%	92.5%	7.5%	0.0%	92.5%	7.5%	0.0%	92.5%	7.5%	0.0%	92.5%	7.5%	Mixed	0.66	0.223
SOSA-13	27.0%	19.5%	53.5%	4.7%	9.8%	85.6%	4.7%	9.8%	85.6%	4.7%	1.9%	93.5%	Premodern	1.66	0.221
SOSA-15	0.0%	33.2%	66.8%	0.0%	33.2%	66.8%	0.0%	33.2%	66.8%	0.0%	32.7%	67.3%	Modern	1.11	0.761
SOSA-22[2]	0.0%	100.0%	0.0%	0.0%	100.0%	0.0%	0.0%	100.0%	0.0%	0.0%	100.0%	0.0%	Modern	0.02	0.657
SOSA-35[2]	0.5%	98.6%	0.9%	0.5%	98.6%	0.9%	0.5%	98.6%	0.9%	0.5%	98.2%	1.4%	PremodernOrMixed	0.34	0.223
TMART-01	0.0%	83.5%	16.5%	0.0%	82.6%	17.4%	0.0%	82.6%	17.4%	0.0%	82.6%	17.4%	Mixed	10.08	0.651
TMART-02	0.0%	67.6%	32.4%	0.0%	67.6%	32.4%	0.0%	67.6%	32.4%	0.0%	67.6%	32.4%	Mixed	1.27	0.824
TMART-03	0.0%	95.0%	5.0%	0.0%	95.0%	5.0%	0.0%	95.0%	5.0%	0.0%	95.0%	5.0%	Premodern	1.34	0.587

Online resource (suppplementary table) 2. Selected attributes of GAMA-PBP (https://ca.water.usgs.gov/gama/) status wells sampled for orthophosphate concentration-page 91.

GAMA-PBP ID	USGS Station ID[1]	GAMA-PBP study unit	GAMA-PBP study area[3]	Hydrogeologic zone
TMART-04	391900120080001	Tahoe-Martis	Martis Valley	Mountain
TMART-05	392000120080001	Tahoe-Martis	Martis Valley	Mountain
TMART-06	391900120100001	Tahoe-Martis	Martis Valley	Mountain
TMART-07	391900120120001	Tahoe-Martis	Martis Valley	Mountain
TMART-08	392000120100001	Tahoe-Martis	Martis Valley	Mountain
TMART-09	392200120110001	Tahoe-Martis	Martis Valley	Mountain
TMART-10	392000120150001	Tahoe-Martis	Martis Valley	Mountain
TMART-11	391900120070001	Tahoe-Martis	Martis Valley	Mountain
TMART-12	392400120100002	Tahoe-Martis	Martis Valley	Mountain
TMART-13	391800120120001	Tahoe-Martis	Martis Valley	Mountain
TMART-14	391700120070001	Tahoe-Martis	Martis Valley	Mountain
TROCK-01	391900120170001	Tahoe-Martis	Hard Rock Tahoe-Martis	Mountain
TROCK-02	385700120060001	Tahoe-Martis	Hard Rock Tahoe-Martis	Mountain
TROCK-03	392400120100001	Tahoe-Martis	Hard Rock Tahoe-Martis	Mountain
TROCK-04	390900120140001	Tahoe-Martis	Hard Rock Tahoe-Martis	Mountain
TROCK-05	391000120120001	Tahoe-Martis	Hard Rock Tahoe-Martis	Mountain
TROCK-06	391552120045101	Tahoe-Martis	Hard Rock Tahoe-Martis	Mountain
TROCK-07	390157120070501	Tahoe-Martis	Hard Rock Tahoe-Martis	Mountain
TROCK-08	385000120040001	Tahoe-Martis	Hard Rock Tahoe-Martis	Mountain
TROCK-09	384900120020001	Tahoe-Martis	Hard Rock Tahoe-Martis	Mountain
TROCK-10	391700120120001	Tahoe-Martis	Hard Rock Tahoe-Martis	Mountain
TROCK-11	385500120030001	Tahoe-Martis	Hard Rock Tahoe-Martis	Mountain
TROCK-12[2]	391300120120001	Tahoe-Martis	Hard Rock Tahoe-Martis	Mountain
TROCK-13	391500120120001	Tahoe-Martis	Hard Rock Tahoe-Martis	Mountain
TTAHO-01	390600120090001	Tahoe-Martis	Tahoe Valley basins	Mountain
TTAHO-02	391500120020001	Tahoe-Martis	Tahoe Valley basins	Mountain
TTAHO-03	390200120070001	Tahoe-Martis	Tahoe Valley basins	Mountain
TTAHO-04	390300120090001	Tahoe-Martis	Tahoe Valley basins	Mountain
TTAHO-05	391000120080001	Tahoe-Martis	Tahoe Valley basins	Mountain
TTAHO-06	385500120000001	Tahoe-Martis	Tahoe Valley basins	Mountain

Online resource (suppplementary table) 2. Selected attributes of GAMA-PBP (https://ca.water.usgs.gov/gama/) status wells sampled for orthophosphate concentration-page 92.

GAMA-PBP ID	USEPA Level III Ecoregions[4]	Level III Ecoregion Reference Concentration[4]	Status Sample Date	Status Sample Orthophosphate Concentration (mg/L as P)	Relative Concentration Category[5]	Redox state[6]	Elevation of LSD (meters above NAVD 88)[7]	Well depth (meters below LSD)[8]	Agricultural land use in 1974[9] (percent)	Natural land use in 1974[9] (percent)	Urban land use in 1974[9] (percent)
TMART-04	Sierra Nevada	0.0150	Jun 27 2007	0.070	moderate	oxic	1,795	313	0.0%	24.7%	75.3%
TMART-05	Sierra Nevada	0.0150	Jun 27 2007	0.325	high	oxic	1,819	418	0.0%	100.0%	0.0%
TMART-06	Sierra Nevada	0.0150	Jun 28 2007	0.043	moderate	oxic	1,792	274	0.0%	29.5%	70.5%
TMART-07	Sierra Nevada	0.0150	Jul 10 2007	0.026	moderate	oxic	1,793	32	0.0%	39.1%	60.9%
TMART-08	Sierra Nevada	0.0150	Jul 10 2007	0.023	moderate	oxic	1,821	187	0.0%	58.4%	41.6%
TMART-09	Sierra Nevada	0.0150	Sep 20 2007	0.004	low	oxic	1,811	37	0.0%	95.5%	4.5%
TMART-10	Sierra Nevada	0.0150	Jul 16 2007	0.045	moderate	oxic	2,013	183	0.0%	31.8%	68.2%
TMART-11	Sierra Nevada	0.0150	Jul 18 2007	0.028	moderate	oxic	1,803	40	0.0%	100.0%	0.0%
TMART-12	Sierra Nevada	0.0150	Jul 26 2007	0.041	moderate	oxic	1,789	76	0.0%	98.1%	1.9%
TMART-13	Sierra Nevada	0.0150	Sep 11 2007	0.106	high	oxic	1,785	53	0.0%	94.6%	5.4%
TMART-14	Sierra Nevada	0.0150	Sep 12 2007	0.050	moderate	oxic	1,780	244	0.0%	97.7%	2.3%
TROCK-01	Sierra Nevada	0.0150	Jul 9 2007	0.024	moderate	oxic	1,842	82	0.0%	67.9%	32.1%
TROCK-02	Sierra Nevada	0.0150	Jul 11 2007	0.006	low	oxic	2,007	0	0.0%	96.3%	3.7%
TROCK-03	Sierra Nevada	0.0150	Jul 16 2007	0.049	moderate	oxic	1,848	89	0.0%	100.0%	0.0%
TROCK-04	Sierra Nevada	0.0150	Jul 17 2007	0.048	moderate	oxic	2,197	155	0.0%	100.0%	0.0%
TROCK-05	Sierra Nevada	0.0150	Jul 17 2007	0.038	moderate	oxic	2,060	0	0.0%	99.1%	0.9%
TROCK-06	Sierra Nevada	0.0150	Jul 18 2007	0.029	moderate	oxic	1,906	66	0.0%	43.6%	56.4%
TROCK-07	Sierra Nevada	0.0150	Jul 24 2007	0.099	moderate	oxic	1,898	98	0.0%	52.3%	47.7%
TROCK-08	Sierra Nevada	0.0150	Jul 30 2007	0.010	low	oxic	2,471	0	0.0%	100.0%	0.0%
TROCK-09	Sierra Nevada	0.0150	Jul 30 2007	0.015	moderate	oxic	2,321	0	0.0%	97.7%	2.3%
TROCK-10	Sierra Nevada	0.0150	Jul 31 2007	0.020	moderate	oxic	1,925	186	0.0%	100.0%	0.0%
TROCK-11	Sierra Nevada	0.0150	Aug 1 2007	0.014	low	oxic	1,937	15	0.0%	100.0%	0.0%
TROCK-12[2]	Sierra Nevada	0.0150	Sep 12 2007	0.011	low	anoxic	1,846	na	0.0%	95.5%	4.5%
TROCK-13	Sierra Nevada	0.0150	Sep 13 2007	0.036	moderate	oxic	1,824	31	0.0%	95.4%	4.6%
TTAHO-01	Sierra Nevada	0.0150	Jul 11 2007	0.105	high	oxic	1,900	15	0.0%	86.4%	13.6%
TTAHO-02	Sierra Nevada	0.0150	Jul 19 2007	0.077	moderate	oxic	1,964	268	0.0%	80.6%	19.4%
TTAHO-03	Sierra Nevada	0.0150	Jul 23 2007	0.032	moderate	oxic	1,918	46	0.0%	87.2%	12.8%
TTAHO-04	Sierra Nevada	0.0150	Jul 24 2007	0.031	moderate	oxic	1,952	73	0.0%	38.1%	61.9%
TTAHO-05	Sierra Nevada	0.0150	Jul 25 2007	0.064	moderate	oxic	1,951	147	0.0%	54.5%	45.5%
TTAHO-06	Sierra Nevada	0.0150	Aug 2 2007	0.016	moderate	oxic	1,900	151	0.0%	24.3%	75.7%

Online resource (suppplementary table) 2. Selected attributes of GAMA-PBP (https://ca.water.usgs.gov/gama/) status wells sampled for orthophosphate concentration-page 93.

GAMA-PBP ID	Agricultural land use in 1982[9] (percent)	Natural land use in 1982[9] (percent)	Urban land use in 1982[9] (percent)	Agricultural land use in 1992[9] (percent)	Natural land use in 1992[9] (percent)	Urban land use in 1992[9] (percent)	Agricultural land use in 2002[9] (percent)	Natural land use in 2002[9] (percent)	Urban land use in 2002[9] (percent)	Agricultural land use in 2012[9] (percent)	Natural land use in 2012[9] (percent)	Urban land use in 2012[9] (percent)	Age Classification[10]	Septic Tanks[11]	Aridity[12]
TMART-04	0.0%	24.2%	75.8%	0.0%	22.3%	77.7%	0.0%	21.4%	78.6%	0.0%	20.5%	79.5%	Mixed	2.28	0.701
TMART-05	0.0%	100.0%	0.0%	0.0%	100.0%	0.0%	0.0%	100.0%	0.0%	0.0%	100.0%	0.0%	Mixed	1.27	0.664
TMART-06	0.0%	21.8%	78.2%	0.0%	21.8%	78.2%	0.0%	21.8%	78.2%	0.0%	21.8%	78.2%	Modern	64.35	0.761
TMART-07	0.0%	38.6%	61.4%	0.0%	38.2%	61.8%	0.0%	38.2%	61.8%	0.0%	31.4%	68.6%	Modern	2.02	0.802
TMART-08	0.0%	58.4%	41.6%	0.0%	6.4%	93.6%	0.0%	6.4%	93.6%	0.0%	3.7%	96.3%	Mixed	18.73	0.711
TMART-09	0.0%	95.5%	4.5%	0.0%	95.5%	4.5%	0.0%	95.5%	4.5%	0.0%	95.5%	4.5%	Modern	0.34	0.777
TMART-10	0.0%	0.9%	99.1%	0.0%	0.9%	99.1%	0.0%	0.9%	99.1%	0.0%	0.9%	99.1%	Premodern	0.49	1.019
TMART-11	0.0%	100.0%	0.0%	0.0%	100.0%	0.0%	0.0%	100.0%	0.0%	0.0%	100.0%	0.0%	Mixed	1.41	0.683
TMART-12	0.0%	98.1%	1.9%	0.0%	98.1%	1.9%	0.0%	98.1%	1.9%	0.0%	98.1%	1.9%	Premodern	0.97	0.737
TMART-13	0.0%	94.6%	5.4%	0.0%	94.6%	5.4%	0.0%	94.6%	5.4%	0.0%	94.1%	5.9%	Premodern	5.27	0.874
TMART-14	0.0%	97.7%	2.3%	0.0%	62.5%	37.5%	0.0%	59.3%	40.7%	0.0%	57.4%	42.6%	Premodern	5.27	0.783
TROCK-01	0.0%	13.3%	86.7%	0.0%	11.9%	88.1%	0.0%	11.9%	88.1%	0.0%	11.9%	88.1%	Modern	0.85	0.955
TROCK-02	0.0%	96.3%	3.7%	0.0%	95.9%	4.1%	0.0%	95.4%	4.6%	0.0%	95.4%	4.6%	Modern	0.00	1.128
TROCK-03	0.0%	100.0%	0.0%	0.0%	100.0%	0.0%	0.0%	100.0%	0.0%	0.0%	100.0%	0.0%	Premodern	0.97	0.729
TROCK-04	0.0%	100.0%	0.0%	0.0%	100.0%	0.0%	0.0%	100.0%	0.0%	0.0%	100.0%	0.0%	Modern	1.20	1.578
TROCK-05	0.0%	48.8%	51.2%	0.0%	48.8%	51.2%	0.0%	48.8%	51.2%	0.0%	48.8%	51.2%	Modern	1.20	1.343
TROCK-06	0.0%	42.7%	57.3%	0.0%	42.7%	57.3%	0.0%	42.7%	57.3%	0.0%	42.7%	57.3%	Modern	0.00	0.824
TROCK-07	0.0%	52.3%	47.7%	0.0%	52.3%	47.7%	0.0%	52.3%	47.7%	0.0%	51.8%	48.2%	Modern	0.00	0.816
TROCK-08	0.0%	100.0%	0.0%	0.0%	100.0%	0.0%	0.0%	100.0%	0.0%	0.0%	100.0%	0.0%	ModernOrMixed	0.00	1.287
TROCK-09	0.0%	97.7%	2.3%	0.0%	97.7%	2.3%	0.0%	97.7%	2.3%	0.0%	97.7%	2.3%	Modern	0.00	1.000
TROCK-10	0.0%	100.0%	0.0%	0.0%	100.0%	0.0%	0.0%	100.0%	0.0%	0.0%	100.0%	0.0%	Mixed	5.27	0.886
TROCK-11	0.0%	100.0%	0.0%	0.0%	100.0%	0.0%	0.0%	100.0%	0.0%	0.0%	100.0%	0.0%	Modern	0.00	0.772
TROCK-12[2]	0.0%	56.6%	43.4%	0.0%	56.6%	43.4%	0.0%	56.6%	43.4%	0.0%	56.6%	43.4%	Premodern	2.63	0.986
TROCK-13	0.0%	94.5%	5.5%	0.0%	94.0%	6.0%	0.0%	93.6%	6.4%	0.0%	93.6%	6.4%	Premodern	5.27	0.997
TTAHO-01	0.0%	86.4%	13.6%	0.0%	86.0%	14.0%	0.0%	86.0%	14.0%	0.0%	86.0%	14.0%	Modern	0.00	0.815
TTAHO-02	0.0%	48.8%	51.2%	0.0%	47.5%	52.5%	0.0%	47.5%	52.5%	0.0%	47.5%	52.5%	Premodern	0.00	0.789
TTAHO-03	0.0%	87.2%	12.8%	0.0%	87.2%	12.8%	0.0%	87.2%	12.8%	0.0%	87.2%	12.8%	Modern	0.00	0.784
TTAHO-04	0.0%	38.1%	61.9%	0.0%	38.1%	61.9%	0.0%	38.1%	61.9%	0.0%	38.1%	61.9%	Modern	0.00	0.820
TTAHO-05	0.0%	54.5%	45.5%	0.0%	54.5%	45.5%	0.0%	54.5%	45.5%	0.0%	54.5%	45.5%	Mixed	0.00	0.790
TTAHO-06	0.0%	24.3%	75.7%	0.0%	21.5%	78.5%	0.0%	21.5%	78.5%	0.0%	21.5%	78.5%	Mixed	3.07	0.635

Online resource (suppplementary table) 2. Selected attributes of GAMA-PBP (https://ca.water.usgs.gov/gama/) status wells sampled for orthophosphate concentration-page 94.

GAMA-PBP ID	USGS Station ID[1]	GAMA-PBP study unit	GAMA-PBP study area[3]	Hydrogeologic zone
TTAHO-07	385200120000001	Tahoe-Martis	Tahoe Valley basins	Mountain
TTAHO-08	385532119590001	Tahoe-Martis	Tahoe Valley basins	Mountain
TTAHO-09	385627120034401	Tahoe-Martis	Tahoe Valley basins	Mountain
TTAHO-10	385238120015101	Tahoe-Martis	Tahoe Valley basins	Mountain
TTAHO-11	384800120010001	Tahoe-Martis	Tahoe Valley basins	Mountain
TTAHO-12	385157120005801	Tahoe-Martis	Tahoe Valley basins	Mountain
TTAHO-13	385522119580204	Tahoe-Martis	Tahoe Valley basins	Mountain
TTAHO-14	385729119565101	Tahoe-Martis	Tahoe Valley basins	Mountain
CLABCB-02	335209118082001	Coastal Los Angeles Basin	Central Basin	Southern California
CLABCB-03	335817118051301	Coastal Los Angeles Basin	Central Basin	Southern California
CLABCB-04	334836118061201	Coastal Los Angeles Basin	Central Basin	Southern California
CLABCB-17	335712118054901	Coastal Los Angeles Basin	Central Basin	Southern California
CLABDA-01	340200118270001	Coastal Los Angeles Basin	Santa Monica basin	Southern California
CLABDA-02	340152118273601	Coastal Los Angeles Basin	Santa Monica basin	Southern California
CLABDA-03	340400118240001	Coastal Los Angeles Basin	Hollywood	Southern California
CLABDA-04	340235118295901	Coastal Los Angeles Basin	Santa Monica basin	Southern California
CLABDA-05	340400118230001	Coastal Los Angeles Basin	Hollywood	Southern California
CLABDA-06	335900118220001	Coastal Los Angeles Basin	Santa Monica basin	Southern California
CLABOC-01	334631117504101	Coastal Los Angeles Basin	Orange County Coastal Plain	Southern California
CLABOC-02	334932117532401	Coastal Los Angeles Basin	Orange County Coastal Plain	Southern California
CLABOC-20	335210117422801	Coastal Los Angeles Basin	Orange County Coastal Plain	Southern California
CLABWB-04	335800118230001	Coastal Los Angeles Basin	West Coast basin	Southern California
SDALLV-01	332005117004101	San Diego Drainages hydrogeologic province	San Diego alluvial basins	Southern California
SDALLV-02	333147117401901	San Diego Drainages hydrogeologic province	San Diego alluvial basins	Southern California
SDALLV-03	323925117044001	San Diego Drainages hydrogeologic province	San Diego alluvial basins	Southern California
SDALLV-06	331334117202102	San Diego Drainages hydrogeologic province	San Diego alluvial basins	Southern California
SDALLV-09	324111117052601	San Diego Drainages hydrogeologic province	San Diego alluvial basins	Southern California
SDALLV-13	331707117223601	San Diego Drainages hydrogeologic province	San Diego alluvial basins	Southern California
SDHDRK-04	332011116534301	San Diego Drainages hydrogeologic province	San Diego hard rock	Southern California
SDHDRK-05	330447117065301	San Diego Drainages hydrogeologic province	San Diego hard rock	Southern California

Online resource (suppplementary table) 2. Selected attributes of GAMA-PBP (https://ca.water.usgs.gov/gama/) status wells sampled for orthophosphate concentration-page 95.

GAMA-PBP ID	USEPA Level III Ecoregions[4]	Level III Ecoregion Reference Concentration[4]	Status Sample Date	Status Sample Orthophosphate Concentration (mg/L as P)	Relative Concentration Category[5]	Redox state[6]	Elevation of LSD (meters above NAVD 88)[7]	Well depth (meters below LSD)[8]	Agricultural land use in 1974[9] (percent)	Natural land use in 1974[9] (percent)	Urban land use in 1974[9] (percent)
TTAHO-07	Sierra Nevada	0.0150	Aug 13 2007	0.153	high	oxic	1,923	81	0.0%	16.8%	83.2%
TTAHO-08	Sierra Nevada	0.0150	Aug 14 2007	0.044	moderate	oxic	1,916	48	0.0%	8.9%	91.1%
TTAHO-09	Sierra Nevada	0.0150	Aug 15 2007	0.090	moderate	oxic	1,899	30	0.0%	100.0%	0.0%
TTAHO-10	Sierra Nevada	0.0150	Aug 16 2007	0.035	moderate	oxic	1,952	76	0.0%	64.8%	35.2%
TTAHO-11	Sierra Nevada	0.0150	Aug 21 2007	0.034	moderate	anoxic	1,973	61	0.0%	75.3%	24.7%
TTAHO-12	Sierra Nevada	0.0150	Aug 22 2007	0.014	low	oxic	1,930	99	0.0%	37.0%	63.0%
TTAHO-13	Sierra Nevada	0.0150	Aug 29 2007	0.024	moderate	oxic	1,923	41	0.0%	75.3%	24.7%
TTAHO-14	Sierra Nevada	0.0150	Sep 10 2007	0.009	low	oxic	1,954	76	0.0%	5.5%	94.5%
CLABCB-02	Central California Foothills and Coastal Mountains	0.0300	Aug 15 2006	0.067	moderate	anoxic	20	205	0.0%	0.0%	100.0%
CLABCB-03	Central California Foothills and Coastal Mountains	0.0300	Aug 16 2006	0.027	low	oxic	48	158	0.0%	0.0%	100.0%
CLABCB-04	Central California Foothills and Coastal Mountains	0.0300	Aug 17 2006	0.022	low	anoxic	6	255	0.0%	0.0%	100.0%
CLABCB-17	Central California Foothills and Coastal Mountains	0.0300	Sep 14 2006	0.025	low	oxic	43	191	0.0%	0.0%	100.0%
CLABDA-01	Central California Foothills and Coastal Mountains	0.0300	Aug 7 2006	0.076	moderate	oxic	74	76	0.0%	0.0%	100.0%
CLABDA-02	Central California Foothills and Coastal Mountains	0.0300	Aug 8 2006	0.110	high	oxic	48	168	0.0%	0.0%	100.0%
CLABDA-03	Central California Foothills and Coastal Mountains	0.0300	Aug 9 2006	0.038	moderate	anoxic	82	203	0.0%	0.0%	100.0%
CLABDA-04	Central California Foothills and Coastal Mountains	0.0300	Aug 10 2006	0.125	high	oxic	95	86	0.0%	0.0%	100.0%
CLABDA-05	Central California Foothills and Coastal Mountains	0.0300	Nov 14 2006	0.054	moderate	anoxic	72	226	0.0%	0.0%	100.0%
CLABDA-06	Central California Foothills and Coastal Mountains	0.0300	Nov 15 2006	0.135	high	anoxic	71	134	0.0%	0.0%	100.0%
CLABOC-01	Central California Foothills and Coastal Mountains	0.0300	Jun 5 2006	0.023	low	oxic	62	396	10.5%	0.0%	89.5%
CLABOC-02	Central California Foothills and Coastal Mountains	0.0300	Jun 7 2006	0.030	moderate	oxic	55	472	0.0%	0.0%	100.0%
CLABOC-20	Central California Foothills and Coastal Mountains	0.0300	Sep 13 2006	0.351	high	anoxic	117	30	0.0%	27.1%	72.9%
CLABWB-04	Central California Foothills and Coastal Mountains	0.0300	Sep 12 2006	0.160	high	anoxic	23	189	0.0%	0.0%	100.0%
SDALLV-01	Central California Foothills and Coastal Mountains	0.0300	Jun 30 2004	0.021	low	oxic	214	61	63.3%	34.0%	2.8%
SDALLV-02	Central California Foothills and Coastal Mountains	0.0300	Jul 1 2004	0.016	low	oxic	64	40	9.1%	0.0%	90.9%
SDALLV-03	Central California Foothills and Coastal Mountains	0.0300	Jul 12 2004	0.022	low	anoxic	7	185	0.0%	0.0%	100.0%
SDALLV-06	Central California Foothills and Coastal Mountains	0.0300	Jul 13 2004	0.062	moderate	anoxic	12	61	2.7%	0.0%	97.3%
SDALLV-09	Central California Foothills and Coastal Mountains	0.0300	Jul 14 2004	0.008	low	anoxic	28	247	0.0%	0.0%	100.0%
SDALLV-13	Central California Foothills and Coastal Mountains	0.0300	Jul 15 2004	0.092	moderate	anoxic	13	55	0.0%	71.4%	28.6%
SDHDRK-04	Southern California Mountains	0.0109	Jul 19 2004	0.003	low	oxic	1,450	96	0.0%	100.0%	0.0%
SDHDRK-05	Central California Foothills and Coastal Mountains	0.0300	Jul 20 2004	0.026	low	oxic	104	137	0.0%	0.0%	100.0%

Online resource (suppplementary table) 2. Selected attributes of GAMA-PBP (https://ca.water.usgs.gov/gama/) status wells sampled for orthophosphate concentration-page 96.

GAMA-PBP ID	Agricultural land use in 1982[9] (percent)	Natural land use in 1982[9] (percent)	Urban land use in 1982[9] (percent)	Agricultural land use in 1992[9] (percent)	Natural land use in 1992[9] (percent)	Urban land use in 1992[9] (percent)	Agricultural land use in 2002[9] (percent)	Natural land use in 2002[9] (percent)	Urban land use in 2002[9] (percent)	Agricultural land use in 2012[9] (percent)	Natural land use in 2012[9] (percent)	Urban land use in 2012[9] (percent)	Age Classification[10]	Septic Tanks[11]	Aridity[12]
TTAHO-07	0.0%	16.4%	83.6%	0.0%	16.4%	83.6%	0.0%	16.4%	83.6%	0.0%	16.4%	83.6%	Modern	0.34	0.803
TTAHO-08	0.0%	8.9%	91.1%	0.0%	8.9%	91.1%	0.0%	8.9%	91.1%	0.0%	8.9%	91.1%	Modern	0.09	0.582
TTAHO-09	0.0%	100.0%	0.0%	0.0%	100.0%	0.0%	0.0%	100.0%	0.0%	0.0%	100.0%	0.0%	Premodern	0.00	0.724
TTAHO-10	0.0%	64.3%	35.7%	0.0%	53.1%	46.9%	0.0%	53.1%	46.9%	0.0%	53.1%	46.9%	Premodern	0.00	0.891
TTAHO-11	0.0%	75.3%	24.7%	0.0%	75.3%	24.7%	0.0%	75.3%	24.7%	0.0%	75.3%	24.7%	Mixed	0.00	1.048
TTAHO-12	0.0%	29.2%	70.8%	0.0%	27.4%	72.6%	0.0%	26.9%	73.1%	0.0%	26.5%	73.5%	Modern	0.00	0.849
TTAHO-13	0.0%	74.9%	25.1%	0.0%	68.0%	32.0%	0.0%	68.0%	32.0%	0.0%	68.0%	32.0%	Modern	0.01	0.564
TTAHO-14	0.0%	5.1%	94.9%	0.0%	5.1%	94.9%	0.0%	5.1%	94.9%	0.0%	4.6%	95.4%	Premodern	82.88	0.479
CLABCB-02	0.0%	0.0%	100.0%	0.0%	0.0%	100.0%	0.0%	0.0%	100.0%	0.0%	0.0%	100.0%	Modern	9.62	0.243
CLABCB-03	0.0%	0.0%	100.0%	0.0%	0.0%	100.0%	0.0%	0.0%	100.0%	0.0%	0.0%	100.0%	Modern	0.01	0.262
CLABCB-04	0.0%	0.0%	100.0%	0.0%	0.0%	100.0%	0.0%	0.0%	100.0%	0.0%	0.0%	100.0%	Premodern	0.00	0.222
CLABCB-17	0.0%	0.0%	100.0%	0.0%	0.0%	100.0%	0.0%	0.0%	100.0%	0.0%	0.0%	100.0%	ModernOrMixed	0.40	0.263
CLABDA-01	0.0%	0.0%	100.0%	0.0%	0.0%	100.0%	0.0%	0.0%	100.0%	0.0%	0.0%	100.0%	Modern	3.88	0.287
CLABDA-02	0.0%	0.0%	100.0%	0.0%	0.0%	100.0%	0.0%	0.0%	100.0%	0.0%	0.0%	100.0%	ModernOrMixed	4.46	0.278
CLABDA-03	0.0%	0.0%	100.0%	0.0%	0.0%	100.0%	0.0%	0.0%	100.0%	0.0%	0.0%	100.0%	Premodern	0.09	0.290
CLABDA-04	0.0%	0.0%	100.0%	0.0%	0.0%	100.0%	0.0%	0.0%	100.0%	0.0%	0.0%	100.0%	Modern	0.82	0.298
CLABDA-05	0.0%	0.0%	100.0%	0.0%	0.0%	100.0%	0.0%	0.0%	100.0%	0.0%	0.0%	100.0%	Premodern	0.98	0.300
CLABDA-06	0.0%	0.0%	100.0%	0.0%	0.0%	100.0%	0.0%	0.0%	100.0%	0.0%	0.0%	100.0%	ModernOrMixed	0.00	0.262
CLABOC-01	2.7%	0.0%	97.3%	0.0%	0.0%	100.0%	0.0%	0.0%	100.0%	0.0%	0.0%	100.0%	ModernOrMixed	9.97	0.248
CLABOC-02	0.0%	0.0%	100.0%	0.0%	0.0%	100.0%	0.0%	0.0%	100.0%	0.0%	0.0%	100.0%	Modern	0.00	0.242
CLABOC-20	0.0%	26.6%	73.4%	0.0%	19.7%	80.3%	0.0%	13.3%	86.7%	0.0%	5.5%	94.5%	Modern	0.15	0.292
CLABWB-04	0.0%	0.0%	100.0%	0.0%	0.0%	100.0%	0.0%	0.0%	100.0%	0.0%	0.0%	100.0%	Premodern	0.00	0.259
SDALLV-01	63.3%	34.0%	2.8%	63.3%	33.5%	3.3%	67.4%	29.3%	3.3%	67.4%	29.3%	3.3%	Modern	3.51	0.305
SDALLV-02	8.7%	0.0%	91.3%	0.5%	0.0%	99.5%	0.0%	0.0%	100.0%	0.0%	0.0%	100.0%	Modern	9.70	0.250
SDALLV-03	0.0%	0.0%	100.0%	0.0%	0.0%	100.0%	0.0%	0.0%	100.0%	0.0%	0.0%	100.0%	Premodern	34.04	0.190
SDALLV-06	0.5%	0.0%	99.5%	0.0%	0.0%	100.0%	0.0%	0.0%	100.0%	0.0%	0.0%	100.0%	Modern	0.05	0.228
SDALLV-09	0.0%	0.0%	100.0%	0.0%	0.0%	100.0%	0.0%	0.0%	100.0%	0.0%	0.0%	100.0%	Premodern	0.05	0.195
SDALLV-13	0.0%	71.4%	28.6%	0.0%	70.9%	29.1%	0.0%	67.1%	32.9%	0.0%	62.4%	37.6%	Modern	0.08	0.246
SDHDRK-04	0.0%	100.0%	0.0%	0.0%	100.0%	0.0%	0.0%	100.0%	0.0%	0.0%	100.0%	0.0%	Modern	0.66	0.540
SDHDRK-05	0.0%	0.0%	100.0%	0.0%	0.0%	100.0%	0.0%	0.0%	100.0%	0.0%	0.0%	100.0%	Modern	65.63	0.277

Online resource (suppplementary table) 2. Selected attributes of GAMA-PBP (https://ca.water.usgs.gov/gama/) status wells sampled for orthophosphate concentration-page 97.

GAMA-PBP ID	USGS Station ID[1]	GAMA-PBP study unit	GAMA-PBP study area[3]	Hydrogeologic zone
SDTEM-06[2]	332800117070401	San Diego Drainages hydrogeologic province	Temecula Valley	Southern California
SDTEM-10	333010117003101	San Diego Drainages hydrogeologic province	Temecula Valley	Southern California
SDTEM-12	333159117015201	San Diego Drainages hydrogeologic province	Temecula Valley	Southern California
SDTEM-13	333202117103201	San Diego Drainages hydrogeologic province	Temecula Valley	Southern California
SDTEMFP-01	332845117064801	San Diego Drainages hydrogeologic province	Temecula Valley	Southern California
SDTEMFP-03	332922117025301	San Diego Drainages hydrogeologic province	Temecula Valley	Southern California
SDWARN-04	331508116422901	San Diego Drainages hydrogeologic province	Warner Valley	Southern California
SDWARN-05	331442116390201	San Diego Drainages hydrogeologic province	Warner Valley	Southern California
SDWARN-06	331533116395601	San Diego Drainages hydrogeologic province	Warner Valley	Southern California
ULASF-08	340900118170001	San Fernando-San Gabriel	San Fernando Valley	Southern California
ULASF-09	341100118130001	San Fernando-San Gabriel	San Fernando Valley	Southern California
ULASF-10	341000118220001	San Fernando-San Gabriel	San Fernando Valley	Southern California
ULASF-12	340800118160001	San Fernando-San Gabriel	San Fernando Valley	Southern California
ULASG-05	340321118000501	San Fernando-San Gabriel	San Gabriel Valley	Southern California
ULASG-06	340148118030901	San Fernando-San Gabriel	San Gabriel Valley	Southern California
ULASG-08	340835118055401	San Fernando-San Gabriel	San Gabriel Valley	Southern California
ULASG-11	341100118092001	San Fernando-San Gabriel	San Gabriel Valley	Southern California
ULASG-14	340700117560001	San Fernando-San Gabriel	San Gabriel Valley	Southern California
ULASG-15	340900117550001	San Fernando-San Gabriel	San Gabriel Valley	Southern California
ULASG-16	340652117504101	San Fernando-San Gabriel	San Gabriel Valley	Southern California
USAWB-02	341018117253201	Upper Santa Ana Watershed	Bunker Hill and Rialto-Colton subbasins	Southern California
USAWB-04	340904117221001	Upper Santa Ana Watershed	Bunker Hill and Rialto-Colton subbasins	Southern California
USAWB-07	340437117170301	Upper Santa Ana Watershed	Bunker Hill and Rialto-Colton subbasins	Southern California
USAWB-08	340717117194601	Upper Santa Ana Watershed	Bunker Hill and Rialto-Colton subbasins	Southern California
USAWB-10	340745117170501	Upper Santa Ana Watershed	Bunker Hill and Rialto-Colton subbasins	Southern California
USAWB-11	340858117152002	Upper Santa Ana Watershed	Bunker Hill and Rialto-Colton subbasins	Southern California
USAWB-14	340700117240001	Upper Santa Ana Watershed	Bunker Hill and Rialto-Colton subbasins	Southern California
USAWB-16	340617117163601	Upper Santa Ana Watershed	Bunker Hill and Rialto-Colton subbasins	Southern California
USAWB-18	340510117201901	Upper Santa Ana Watershed	Bunker Hill and Rialto-Colton subbasins	Southern California
USAWC-01	340300117270001	Upper Santa Ana Watershed	Cucamonga and Chino subbasins	Southern California

Online resource (suppplementary table) 2. Selected attributes of GAMA-PBP (https://ca.water.usgs.gov/gama/) status wells sampled for orthophosphate concentration-page 98.

GAMA-PBP ID	USEPA Level III Ecoregions[4]	Level III Ecoregion Reference Concentration[4]	Status Sample Date	Status Sample Orthophosphate Concentration (mg/L as P)	Relative Concentration Category[5]	Redox state[6]	Elevation of LSD (meters above NAVD 88)[7]	Well depth (meters below LSD)[8]	Agricultural land use in 1974[9] (percent)	Natural land use in 1974[9] (percent)	Urban land use in 1974[9] (percent)
SDTEM-06[2]	Central California Foothills and Coastal Mountains	0.0300	May 26 2004	0.080	moderate	oxic	315	na	55.7%	22.8%	21.5%
SDTEM-10	Central California Foothills and Coastal Mountains	0.0300	Jul 26 2004	0.037	moderate	oxic	388	76	1.4%	97.7%	0.9%
SDTEM-12	Central California Foothills and Coastal Mountains	0.0300	Jun 21 2004	0.019	low	oxic	458	166	57.5%	39.7%	2.7%
SDTEM-13	Central California Foothills and Coastal Mountains	0.0300	Jun 22 2004	0.006	low	oxic	321	262	29.6%	46.9%	23.5%
SDTEMFP-01	Central California Foothills and Coastal Mountains	0.0300	May 19 2004	0.015	low	oxic	318	762	11.4%	64.5%	24.1%
SDTEMFP-03	Central California Foothills and Coastal Mountains	0.0300	Jun 14 2004	0.013	low	oxic	359	264	19.9%	74.5%	5.6%
SDWARN-04	Southern California Mountains	0.0109	Jun 24 2004	0.048	moderate	oxic	866	134	4.1%	94.9%	0.9%
SDWARN-05	Southern California Mountains	0.0109	Jun 28 2004	0.012	moderate	oxic	899	226	0.0%	99.1%	0.9%
SDWARN-06	Southern California Mountains	0.0109	Jun 29 2004	0.049	moderate	oxic	903	223	0.0%	99.1%	0.9%
ULASF-08	Central California Foothills and Coastal Mountains	0.0300	Jun 6 2005	0.015	low	oxic	142	122	0.0%	0.9%	99.1%
ULASF-09	Central California Foothills and Coastal Mountains	0.0300	Jun 7 2005	0.024	low	oxic	310	56	0.0%	0.0%	100.0%
ULASF-10	Central California Foothills and Coastal Mountains	0.0300	Jun 8 2005	0.022	low	oxic	202	283	0.0%	0.0%	100.0%
ULASF-12	Central California Foothills and Coastal Mountains	0.0300	Jun 9 2005	0.046	moderate	oxic	137	61	0.0%	0.0%	100.0%
ULASG-05	Central California Foothills and Coastal Mountains	0.0300	Jun 14 2005	0.040	moderate	oxic	92	393	0.0%	0.0%	100.0%
ULASG-06	Central California Foothills and Coastal Mountains	0.0300	Jun 14 2005	0.008	low	oxic	65	217	5.6%	0.0%	94.4%
ULASG-08	Central California Foothills and Coastal Mountains	0.0300	Jun 15 2005	0.018	low	oxic	219	122	0.0%	0.0%	100.0%
ULASG-11	Central California Foothills and Coastal Mountains	0.0300	Jun 16 2005	0.020	low	oxic	351	149	0.0%	0.0%	100.0%
ULASG-14	Central California Foothills and Coastal Mountains	0.0300	Jun 20 2005	0.005	low	oxic	169	305	0.0%	0.0%	100.0%
ULASG-15	Central California Foothills and Coastal Mountains	0.0300	Jun 23 2005	0.003	low	oxic	208	122	0.0%	26.0%	74.0%
ULASG-16	Central California Foothills and Coastal Mountains	0.0300	Jul 11 2005	0.036	moderate	oxic	238	126	0.0%	0.0%	100.0%
USAWB-02	Central California Foothills and Coastal Mountains	0.0300	Nov 27 2006	0.012	low	oxic	566	68	0.0%	77.3%	22.7%
USAWB-04	Central California Foothills and Coastal Mountains	0.0300	Nov 28 2006	0.018	low	oxic	432	274	0.0%	82.5%	17.5%
USAWB-07	Central California Foothills and Coastal Mountains	0.0300	Nov 29 2006	0.017	low	oxic	305	199	0.0%	0.0%	100.0%
USAWB-08	Central California Foothills and Coastal Mountains	0.0300	Dec 11 2006	0.014	low	oxic	361	177	4.7%	0.0%	95.3%
USAWB-10	Central California Foothills and Coastal Mountains	0.0300	Dec 12 2006	0.022	low	oxic	347	305	0.0%	0.0%	100.0%
USAWB-11	Central California Foothills and Coastal Mountains	0.0300	Dec 13 2006	0.038	moderate	oxic	381	185	0.0%	0.0%	100.0%
USAWB-14	Central California Foothills and Coastal Mountains	0.0300	Dec 14 2006	0.028	low	oxic	432	323	14.1%	0.0%	85.9%
USAWB-16	Central California Foothills and Coastal Mountains	0.0300	Jan 8 2007	0.019	low	oxic	316	294	0.0%	0.0%	100.0%
USAWB-18	Central California Foothills and Coastal Mountains	0.0300	Jan 11 2007	0.035	moderate	oxic	319	163	0.0%	0.0%	100.0%
USAWC-01	Central California Foothills and Coastal Mountains	0.0300	Jan 29 2007	0.018	low	oxic	329	242	1.4%	0.0%	98.6%

Online resource (suppplementary table) 2. Selected attributes of GAMA-PBP (https://ca.water.usgs.gov/gama/) status wells sampled for orthophosphate concentration-page 99.

GAMA-PBP ID	Agricultural land use in 1982[9] (percent)	Natural land use in 1982[9] (percent)	Urban land use in 1982[9] (percent)	Agricultural land use in 1992[9] (percent)	Natural land use in 1992[9] (percent)	Urban land use in 1992[9] (percent)	Agricultural land use in 2002[9] (percent)	Natural land use in 2002[9] (percent)	Urban land use in 2002[9] (percent)	Agricultural land use in 2012[9] (percent)	Natural land use in 2012[9] (percent)	Urban land use in 2012[9] (percent)	Age Classification[10]	Septic Tanks[11]	Aridity[12]
SDTEM-06[2]	55.3%	1.8%	42.9%	29.2%	0.5%	70.3%	22.4%	0.5%	77.2%	4.6%	0.0%	95.4%	Modern	2.61	0.308
SDTEM-10	1.4%	97.7%	0.9%	1.4%	97.7%	0.9%	2.7%	96.3%	0.9%	2.7%	96.3%	0.9%	Modern	3.01	0.337
SDTEM-12	57.5%	39.7%	2.7%	57.5%	0.5%	42.0%	58.4%	0.5%	41.1%	58.4%	0.5%	41.1%	Modern	12.67	0.270
SDTEM-13	23.0%	39.9%	37.1%	9.4%	15.0%	75.6%	0.0%	6.6%	93.4%	0.0%	4.2%	95.8%	Premodern	22.86	0.286
SDTEMFP-01	29.5%	5.0%	65.5%	10.9%	9.1%	80.0%	7.7%	9.1%	83.2%	3.2%	2.7%	94.1%	Mixed	9.89	0.297
SDTEMFP-03	25.9%	68.5%	5.6%	19.4%	75.0%	5.6%	15.7%	74.5%	9.7%	15.7%	74.5%	9.7%	Mixed	2.95	0.276
SDWARN-04	4.1%	94.9%	0.9%	4.1%	94.9%	0.9%	4.1%	94.9%	0.9%	4.1%	94.9%	0.9%	Premodern	0.66	0.421
SDWARN-05	0.0%	99.1%	0.9%	0.0%	99.1%	0.9%	0.0%	99.1%	0.9%	0.0%	99.1%	0.9%	Premodern	0.66	0.372
SDWARN-06	0.0%	99.1%	0.9%	0.0%	99.1%	0.9%	0.0%	98.2%	1.8%	0.0%	98.2%	1.8%	Premodern	0.66	0.380
ULASF-08	0.0%	0.9%	99.1%	0.0%	0.9%	99.1%	0.0%	0.9%	99.1%	0.0%	0.9%	99.1%	Mixed	0.00	0.319
ULASF-09	0.0%	0.0%	100.0%	0.0%	0.0%	100.0%	0.0%	0.0%	100.0%	0.0%	0.0%	100.0%	Modern	1.21	0.397
ULASF-10	0.0%	0.0%	100.0%	0.0%	0.0%	100.0%	0.0%	0.0%	100.0%	0.0%	0.0%	100.0%	Mixed	0.79	0.307
ULASF-12	0.0%	0.0%	100.0%	0.0%	0.0%	100.0%	0.0%	0.0%	100.0%	0.0%	0.0%	100.0%	ModernOrMixed	1.19	0.319
ULASG-05	0.0%	0.0%	100.0%	0.0%	0.0%	100.0%	0.0%	0.0%	100.0%	0.0%	0.0%	100.0%	PremodernOrMixed	1.80	0.272
ULASG-06	5.6%	0.0%	94.4%	0.0%	0.0%	100.0%	0.0%	0.0%	100.0%	0.0%	0.0%	100.0%	Modern	0.05	0.264
ULASG-08	0.0%	0.0%	100.0%	0.0%	0.0%	100.0%	0.0%	0.0%	100.0%	0.0%	0.0%	100.0%	Modern	169.23	0.349
ULASG-11	0.0%	0.0%	100.0%	0.0%	0.0%	100.0%	0.0%	0.0%	100.0%	0.0%	0.0%	100.0%	ModernOrMixed	22.90	0.390
ULASG-14	0.0%	0.0%	100.0%	0.0%	0.0%	100.0%	0.0%	0.0%	100.0%	0.0%	0.0%	100.0%	Modern	0.11	0.329
ULASG-15	0.0%	26.0%	74.0%	0.0%	26.0%	74.0%	0.0%	26.0%	74.0%	0.0%	26.0%	74.0%	Modern	0.00	0.380
ULASG-16	0.0%	0.0%	100.0%	0.0%	0.0%	100.0%	0.0%	0.0%	100.0%	0.0%	0.0%	100.0%	Modern	12.43	0.341
USAWB-02	0.0%	36.4%	63.6%	0.0%	35.0%	65.0%	0.0%	30.0%	70.0%	0.0%	25.9%	74.1%	Modern	1.70	0.464
USAWB-04	0.0%	77.4%	22.6%	0.0%	77.0%	23.0%	0.0%	77.0%	23.0%	0.0%	77.0%	23.0%	Modern	4.39	0.354
USAWB-07	0.0%	0.0%	100.0%	0.0%	0.0%	100.0%	0.0%	0.0%	100.0%	0.0%	0.0%	100.0%	Mixed	1.36	0.231
USAWB-08	1.9%	0.0%	98.1%	0.0%	0.0%	100.0%	0.0%	0.0%	100.0%	0.0%	0.0%	100.0%	Modern	11.96	0.289
USAWB-10	0.0%	0.0%	100.0%	0.0%	0.0%	100.0%	0.0%	0.0%	100.0%	0.0%	0.0%	100.0%	Modern	6.66	0.271
USAWB-11	0.0%	0.0%	100.0%	0.0%	0.0%	100.0%	0.0%	0.0%	100.0%	0.0%	0.0%	100.0%	Modern	1.64	0.300
USAWB-14	14.1%	0.0%	85.9%	0.0%	0.0%	100.0%	0.0%	0.0%	100.0%	0.0%	0.0%	100.0%	Mixed	17.58	0.371
USAWB-16	0.0%	0.0%	100.0%	0.0%	0.0%	100.0%	0.0%	0.0%	100.0%	0.0%	0.0%	100.0%	Premodern	35.97	0.245
USAWB-18	0.0%	0.0%	100.0%	0.0%	0.0%	100.0%	0.0%	0.0%	100.0%	0.0%	0.0%	100.0%	Mixed	44.28	0.265
USAWC-01	0.0%	0.0%	100.0%	0.0%	0.0%	100.0%	0.0%	0.0%	100.0%	0.0%	0.0%	100.0%	Premodern	74.53	0.285

Online resource (suppplementary table) 2. Selected attributes of GAMA-PBP (https://ca.water.usgs.gov/gama/) status wells sampled for orthophosphate concentration-page 100.

GAMA-PBP ID	USGS Station ID[1]	GAMA-PBP study unit	GAMA-PBP study area[3]	Hydrogeologic zone
USAWC-02	340500117280001	Upper Santa Ana Watershed	Cucamonga and Chino subbasins	Southern California
USAWC-04	340303117434701	Upper Santa Ana Watershed	Cucamonga and Chino subbasins	Southern California
USAWC-08	340103117312601	Upper Santa Ana Watershed	Cucamonga and Chino subbasins	Southern California
USAWC-11	340025117301101	Upper Santa Ana Watershed	Cucamonga and Chino subbasins	Southern California
USAWC-12[2]	335800117350001	Upper Santa Ana Watershed	Cucamonga and Chino subbasins	Southern California
USAWC-14	340336117374201	Upper Santa Ana Watershed	Cucamonga and Chino subbasins	Southern California
USAWC-17	340406117351701	Upper Santa Ana Watershed	Cucamonga and Chino subbasins	Southern California
USAWC-20	340615117283201	Upper Santa Ana Watershed	Cucamonga and Chino subbasins	Southern California
USAWC-21	340830117355101	Upper Santa Ana Watershed	Cucamonga and Chino subbasins	Southern California
USAWC-23	335924117412201	Upper Santa Ana Watershed	Cucamonga and Chino subbasins	Southern California
USAWC-24	340200117360001	Upper Santa Ana Watershed	Cucamonga and Chino subbasins	Southern California
USAWC-25	340000117330001	Upper Santa Ana Watershed	Cucamonga and Chino subbasins	Southern California
USAWE-01	333753117174301	Upper Santa Ana Watershed	Elsinore	Southern California
USAWE-02	333844117190801	Upper Santa Ana Watershed	Elsinore	Southern California
USAWE-03	334000117230001	Upper Santa Ana Watershed	Elsinore	Southern California
USAWE-04	334459117283201	Upper Santa Ana Watershed	Elsinore	Southern California
USAWR-03	335900117250001	Upper Santa Ana Watershed	Riverside-Arlington and Temescal subbasins	Southern California
USAWR-05	340306117203001	Upper Santa Ana Watershed	Riverside-Arlington and Temescal subbasins	Southern California
USAWR-06	335500117260001	Upper Santa Ana Watershed	Riverside-Arlington and Temescal subbasins	Southern California
USAWR-07	335300117280001	Upper Santa Ana Watershed	Riverside-Arlington and Temescal subbasins	Southern California
USAWR-08	340033117204001	Upper Santa Ana Watershed	Riverside-Arlington and Temescal subbasins	Southern California
USAWR-09	335810117224701	Upper Santa Ana Watershed	Riverside-Arlington and Temescal subbasins	Southern California
USAWR-10	335227117345501	Upper Santa Ana Watershed	Riverside-Arlington and Temescal subbasins	Southern California
USAWR-11	340444117205401	Upper Santa Ana Watershed	Riverside-Arlington and Temescal subbasins	Southern California
USAWR-12	335224117351101	Upper Santa Ana Watershed	Riverside-Arlington and Temescal subbasins	Southern California
USAWS-02	334948117072401	Upper Santa Ana Watershed	San Jacinto basin	Southern California
USAWS-05	334047117093601	Upper Santa Ana Watershed	San Jacinto basin	Southern California
USAWS-06	334705116583201	Upper Santa Ana Watershed	San Jacinto basin	Southern California
USAWS-08	335646117143201	Upper Santa Ana Watershed	San Jacinto basin	Southern California
USAWS-12	335053117135801	Upper Santa Ana Watershed	San Jacinto basin	Southern California

Status and trends of orthophosphate concentrations in groundwater used for public supply in California *Environmental Monitoring and Assessment, Robert Kent, Tyler D. Johnson, and Michael R. Rosen, U.S. Geological Survey California Water Science Center-rhkent@usgs.gov*

Online resource (suppplementary table) 2. Selected attributes of GAMA-PBP (https://ca.water.usgs.gov/gama/) status wells sampled for orthophosphate concentration-page 101.

GAMA-PBP ID	USEPA Level III Ecoregions[4]	Level III Ecoregion Reference Concentration[4]	Status Sample Date	Status Sample Orthophosphate Concentration (mg/L as P)	Relative Concentration Category[5]	Redox state[6]	Elevation of LSD (meters above NAVD 88)[7]	Well depth (meters below LSD)[8]	Agricultural land use in 1974[9] (percent)	Natural land use in 1974[9] (percent)	Urban land use in 1974[9] (percent)
USAWC-02	Central California Foothills and Coastal Mountains	0.0300	Jan 29 2007	0.039	moderate	oxic	358	265	5.2%	0.0%	94.8%
USAWC-04	Central California Foothills and Coastal Mountains	0.0300	Jan 30 2007	0.020	low	oxic	262	171	0.0%	0.0%	100.0%
USAWC-08	Central California Foothills and Coastal Mountains	0.0300	Jan 31 2007	0.016	low	oxic	240	113	9.6%	5.5%	84.9%
USAWC-11	Central California Foothills and Coastal Mountains	0.0300	Feb 1 2007	0.046	moderate	oxic	228	107	22.3%	2.3%	75.5%
USAWC-12[2]	Central California Foothills and Coastal Mountains	0.0300	Feb 1 2007	0.047	moderate	oxic	193	na	86.6%	8.3%	5.1%
USAWC-14	Central California Foothills and Coastal Mountains	0.0300	Feb 12 2007	0.034	moderate	oxic	293	313	0.0%	0.0%	100.0%
USAWC-17	Central California Foothills and Coastal Mountains	0.0300	Feb 13 2007	0.037	moderate	oxic	300	274	15.0%	0.0%	85.0%
USAWC-20	Central California Foothills and Coastal Mountains	0.0300	Feb 14 2007	0.037	moderate	oxic	380	280	0.5%	0.0%	99.5%
USAWC-21	Central California Foothills and Coastal Mountains	0.0300	Feb 14 2007	0.043	moderate	oxic	498	338	44.7%	0.5%	54.8%
USAWC-23	Central California Foothills and Coastal Mountains	0.0300	Feb 15 2007	0.015	low	oxic	193	360	62.3%	0.0%	37.7%
USAWC-24	Central California Foothills and Coastal Mountains	0.0300	Feb 15 2007	0.060	moderate	oxic	257	91	16.4%	0.9%	82.6%
USAWC-25	Central California Foothills and Coastal Mountains	0.0300	Mar 28 2007	0.033	moderate	oxic	236	133	13.2%	15.0%	71.8%
USAWE-01	Central California Foothills and Coastal Mountains	0.0300	Dec 4 2006	0.012	low	oxic	386	436	6.8%	47.3%	45.9%
USAWE-02	Central California Foothills and Coastal Mountains	0.0300	Dec 5 2006	0.027	low	anoxic	380	524	0.0%	94.5%	5.5%
USAWE-03	Central California Foothills and Coastal Mountains	0.0300	Dec 6 2006	0.049	moderate	oxic	400	299	49.3%	1.8%	48.8%
USAWE-04	Central California Foothills and Coastal Mountains	0.0300	Dec 7 2006	0.026	low	oxic	383	226	14.2%	71.6%	14.2%
USAWR-03	Central California Foothills and Coastal Mountains	0.0300	Nov 30 2006	0.030	low	oxic	232	56	21.9%	7.3%	70.8%
USAWR-05	Central California Foothills and Coastal Mountains	0.0300	Dec 12 2006	0.062	moderate	oxic	272	59	1.4%	5.2%	93.4%
USAWR-06	Central California Foothills and Coastal Mountains	0.0300	Jan 9 2007	0.036	moderate	oxic	248	58	0.0%	0.0%	100.0%
USAWR-07	Central California Foothills and Coastal Mountains	0.0300	Jan 9 2007	0.045	moderate	oxic	217	52	19.7%	0.0%	80.3%
USAWR-08	Central California Foothills and Coastal Mountains	0.0300	Jan 10 2007	0.017	low	anoxic	274	123	0.0%	0.0%	100.0%
USAWR-09	Central California Foothills and Coastal Mountains	0.0300	Jan 10 2007	0.028	low	oxic	250	105	0.0%	0.0%	100.0%
USAWR-10	Central California Foothills and Coastal Mountains	0.0300	Jan 10 2007	0.057	moderate	oxic	196	67	12.8%	0.0%	87.2%
USAWR-11	Central California Foothills and Coastal Mountains	0.0300	Jan 11 2007	0.025	low	oxic	333	196	0.5%	0.0%	99.5%
USAWR-12	Central California Foothills and Coastal Mountains	0.0300	Jan 29 2007	0.020	low	oxic	223	157	0.0%	0.0%	100.0%
USAWS-02	Central California Foothills and Coastal Mountains	0.0300	Jan 22 2007	0.035	moderate	oxic	449	158	41.6%	12.7%	45.7%
USAWS-05	Central California Foothills and Coastal Mountains	0.0300	Jan 23 2007	0.059	moderate	oxic	438	191	56.7%	8.4%	34.9%
USAWS-06	Central California Foothills and Coastal Mountains	0.0300	Jan 23 2007	0.511	high	anoxic	467	198	46.1%	0.0%	53.9%
USAWS-08	Central California Foothills and Coastal Mountains	0.0300	Jan 24 2007	0.075	moderate	oxic	504	130	6.4%	0.0%	93.6%
USAWS-12	Central California Foothills and Coastal Mountains	0.0300	Jan 25 2007	0.029	low	oxic	446	232	16.9%	76.3%	6.8%

Status and trends of orthophosphate concentrations in groundwater used for public supply in California *Environmental Monitoring and Assessment, Robert Kent, Tyler D. Johnson, and Michael R. Rosen, U.S. Geological Survey California Water Science Center-rhkent@usgs.gov*

Online resource (suppplementary table) 2. Selected attributes of GAMA-PBP (https://ca.water.usgs.gov/gama/) status wells sampled for orthophosphate concentration-page 102.

GAMA-PBP ID	Agricultural land use in 1982[9] (percent)	Natural land use in 1982[9] (percent)	Urban land use in 1982[9] (percent)	Agricultural land use in 1992[9] (percent)	Natural land use in 1992[9] (percent)	Urban land use in 1992[9] (percent)	Agricultural land use in 2002[9] (percent)	Natural land use in 2002[9] (percent)	Urban land use in 2002[9] (percent)	Agricultural land use in 2012[9] (percent)	Natural land use in 2012[9] (percent)	Urban land use in 2012[9] (percent)	Age Classification[10]	Septic Tanks[11]	Aridity[12]
USAWC-02	0.9%	0.0%	99.1%	0.0%	0.0%	100.0%	0.0%	0.0%	100.0%	0.0%	0.0%	100.0%	Mixed	238.53	0.303
USAWC-04	0.0%	0.0%	100.0%	0.0%	0.0%	100.0%	0.0%	0.0%	100.0%	0.0%	0.0%	100.0%	Mixed	2.44	0.317
USAWC-08	7.8%	5.5%	86.8%	5.9%	5.5%	88.6%	6.4%	5.0%	88.6%	0.0%	0.0%	100.0%	Mixed	1.86	0.273
USAWC-11	18.2%	2.3%	79.5%	8.2%	2.3%	89.5%	12.7%	1.8%	85.5%	1.4%	0.5%	98.2%	Modern	10.53	0.245
USAWC-12[2]	85.3%	5.1%	9.7%	77.0%	3.2%	19.8%	74.2%	1.4%	24.4%	0.0%	0.0%	100.0%	Modern	8.23	0.272
USAWC-14	0.0%	0.0%	100.0%	0.0%	0.0%	100.0%	0.0%	0.0%	100.0%	0.0%	0.0%	100.0%	Mixed	18.37	0.311
USAWC-17	7.7%	0.0%	92.3%	0.0%	0.0%	100.0%	0.0%	0.0%	100.0%	0.0%	0.0%	100.0%	Modern	5.37	0.295
USAWC-20	0.5%	0.0%	99.5%	0.5%	0.0%	99.5%	0.0%	0.0%	100.0%	0.0%	0.0%	100.0%	Mixed	179.03	0.331
USAWC-21	23.5%	0.5%	76.0%	0.0%	0.0%	100.0%	0.0%	0.0%	100.0%	0.0%	0.0%	100.0%	Mixed	68.43	0.375
USAWC-23	50.0%	0.0%	50.0%	44.5%	0.0%	55.5%	35.0%	0.0%	65.0%	18.2%	0.0%	81.8%	Premodern	0.00	0.294
USAWC-24	12.3%	0.9%	86.8%	9.6%	0.9%	89.5%	0.0%	0.9%	99.1%	0.0%	0.9%	99.1%	Modern	2.48	0.302
USAWC-25	9.1%	14.5%	76.4%	7.3%	13.6%	79.1%	5.5%	11.8%	82.7%	5.5%	1.4%	93.2%	Mixed	27.47	0.288
USAWE-01	10.9%	44.1%	45.0%	0.0%	42.3%	57.7%	0.0%	42.3%	57.7%	0.0%	0.0%	100.0%	Mixed	27.74	0.196
USAWE-02	0.0%	89.9%	10.1%	0.0%	84.4%	15.6%	0.0%	78.9%	21.1%	0.0%	3.7%	96.3%	ModernOrMixed	7.30	0.199
USAWE-03	36.4%	1.4%	62.2%	0.0%	0.0%	100.0%	0.0%	0.0%	100.0%	0.0%	0.0%	100.0%	Mixed	70.85	0.209
USAWE-04	9.2%	53.7%	37.2%	5.5%	42.7%	51.8%	0.5%	24.8%	74.8%	0.0%	9.6%	90.4%	Modern	1.84	0.227
USAWR-03	26.5%	3.7%	69.9%	3.2%	0.0%	96.8%	2.7%	0.0%	97.3%	2.7%	0.0%	97.3%	Modern	10.47	0.183
USAWR-05	1.4%	4.7%	93.9%	0.9%	4.7%	94.4%	0.5%	4.7%	94.8%	0.5%	4.7%	94.8%	Modern	5.89	0.221
USAWR-06	0.0%	0.0%	100.0%	0.0%	0.0%	100.0%	0.0%	0.0%	100.0%	0.0%	0.0%	100.0%	Modern	2.02	0.189
USAWR-07	4.7%	0.0%	95.3%	0.0%	0.0%	100.0%	0.0%	0.0%	100.0%	0.0%	0.0%	100.0%	Modern	14.19	0.214
USAWR-08	0.0%	0.0%	100.0%	0.0%	0.0%	100.0%	0.0%	0.0%	100.0%	0.0%	0.0%	100.0%	Modern	128.39	0.191
USAWR-09	0.0%	0.0%	100.0%	0.0%	0.0%	100.0%	0.0%	0.0%	100.0%	0.0%	0.0%	100.0%	Modern	0.00	0.182
USAWR-10	4.1%	0.0%	95.9%	0.0%	0.0%	100.0%	0.0%	0.0%	100.0%	0.0%	0.0%	100.0%	Modern	0.00	0.239
USAWR-11	0.5%	0.0%	99.5%	0.0%	0.0%	100.0%	0.0%	0.0%	100.0%	0.0%	0.0%	100.0%	Mixed	87.27	0.269
USAWR-12	0.0%	0.0%	100.0%	0.0%	0.0%	100.0%	0.0%	0.0%	100.0%	0.0%	0.0%	100.0%	Mixed	7.12	0.248
USAWS-02	51.1%	6.8%	42.1%	31.2%	4.5%	64.3%	32.1%	2.3%	65.6%	32.1%	2.3%	65.6%	Mixed	46.53	0.211
USAWS-05	56.7%	8.4%	34.9%	0.9%	7.4%	91.6%	0.9%	7.4%	91.6%	0.0%	5.6%	94.4%	Mixed	10.64	0.210
USAWS-06	47.0%	0.0%	53.0%	12.9%	0.0%	87.1%	13.4%	0.0%	86.6%	8.3%	0.0%	91.7%	Premodern	8.95	0.230
USAWS-08	11.9%	0.0%	88.1%	0.0%	0.0%	100.0%	0.0%	0.0%	100.0%	0.0%	0.0%	100.0%	Modern	25.69	0.212
USAWS-12	63.9%	24.2%	11.9%	13.2%	66.7%	20.1%	37.4%	34.2%	28.3%	37.4%	28.3%	34.2%	ModernOrMixed	17.98	0.187

Online resource (suppplementary table) 2. Selected attributes of GAMA-PBP (https://ca.water.usgs.gov/gama/) status wells sampled for orthophosphate concentration-page 103.

GAMA-PBP ID	USGS Station ID[1]	GAMA-PBP study unit	GAMA-PBP study area[3]	Hydrogeologic zone
USAWS-14	334621116564601	Upper Santa Ana Watershed	San Jacinto basin	Southern California
USAWS-15	334407116592901	Upper Santa Ana Watershed	San Jacinto basin	Southern California
USAWS-16[2]	335000117030001	Upper Santa Ana Watershed	San Jacinto basin	Southern California
USAWS-18	334503116580501	Upper Santa Ana Watershed	San Jacinto basin	Southern California
USAWS-20	334344116584001	Upper Santa Ana Watershed	San Jacinto basin	Southern California
USAWS-21	335000117000001	Upper Santa Ana Watershed	San Jacinto basin	Southern California
USAWY-04[2]	340137117053501	Upper Santa Ana Watershed	Yucaipa and San Timoteo subbasins	Southern California
USAWY-05	340105117031601	Upper Santa Ana Watershed	Yucaipa and San Timoteo subbasins	Southern California
USAWY-06	335911117025501	Upper Santa Ana Watershed	Yucaipa and San Timoteo subbasins	Southern California
SCRV-03	340912119040901	Santa Clara River Valley	Santa Clara River Valley basins	Southern California
SCRV-04[2]	342700119110001	Santa Clara River Valley	Santa Clara River Valley basins	Southern California
SCRV-06	341204119103001	Santa Clara River Valley	Santa Clara River Valley basins	Southern California
SCRV-07	342500119170001	Santa Clara River Valley	Santa Clara River Valley basins	Southern California
SCRV-09	341700119080001	Santa Clara River Valley	Santa Clara River Valley basins	Southern California
SCRV-10	340932119130501	Santa Clara River Valley	Santa Clara River Valley basins	Southern California
SCRV-12	340932119111101	Santa Clara River Valley	Santa Clara River Valley basins	Southern California
SCRV-16	342123119024201	Santa Clara River Valley	Santa Clara River Valley basins	Southern California
SCRV-19	342200118520001	Santa Clara River Valley	Santa Clara River Valley basins	Southern California
SCRV-27	341400119120001	Santa Clara River Valley	Santa Clara River Valley basins	Southern California
SCRV-28	342243118594301	Santa Clara River Valley	Santa Clara River Valley basins	Southern California
SCRV-33	341217119020201	Santa Clara River Valley	Santa Clara River Valley basins	Southern California
SCRV-35	341600118470002	Santa Clara River Valley	Santa Clara River Valley basins	Southern California
SCRV-37	340730119074701	Santa Clara River Valley	Santa Clara River Valley basins	Southern California
SCRV-39	341615119124201	Santa Clara River Valley	Santa Clara River Valley basins	Southern California
SCRV-42	342403118472601	Santa Clara River Valley	Santa Clara River Valley basins	Southern California
HR-PR-01	330427116481301	Santa Cruz, San Gabriel, and Peninsular Ranges Hard Rock Aquifers	Hard Rock Peninsular Ranges	Southern California
HR-PR-02[2]	333200116430001	Santa Cruz, San Gabriel, and Peninsular Ranges Hard Rock Aquifers	Hard Rock Peninsular Ranges	Southern California
HR-PR-04[2]	331300116450001	Santa Cruz, San Gabriel, and Peninsular Ranges Hard Rock Aquifers	Hard Rock Peninsular Ranges	Southern California
HR-PR-06	324359116292601	Santa Cruz, San Gabriel, and Peninsular Ranges Hard Rock Aquifers	Hard Rock Peninsular Ranges	Southern California
HR-PR-08[2]	333300117280001	Santa Cruz, San Gabriel, and Peninsular Ranges Hard Rock Aquifers	Hard Rock Peninsular Ranges	Southern California

Online resource (suppplementary table) 2. Selected attributes of GAMA-PBP (https://ca.water.usgs.gov/gama/) status wells sampled for orthophosphate concentration-page 104.

GAMA-PBP ID	USEPA Level III Ecoregions[4]	Level III Ecoregion Reference Concentration[4]	Status Sample Date	Status Sample Orthophosphate Concentration (mg/L as P)	Relative Concentration Category[5]	Redox state[6]	Elevation of LSD (meters above NAVD 88)[7]	Well depth (meters below LSD)[8]	Agricultural land use in 1974[9] (percent)	Natural land use in 1974[9] (percent)	Urban land use in 1974[9] (percent)
USAWS-14	Central California Foothills and Coastal Mountains	0.0300	Feb 5 2007	0.048	moderate	anoxic	486	472	43.5%	0.0%	56.5%
USAWS-15	Central California Foothills and Coastal Mountains	0.0300	Feb 6 2007	0.013	low	oxic	479	100	18.8%	0.0%	81.2%
USAWS-16[2]	Central California Foothills and Coastal Mountains	0.0300	Feb 6 2007	0.010	low	oxic	442	na	68.3%	30.3%	1.4%
USAWS-18	Central California Foothills and Coastal Mountains	0.0300	Feb 7 2007	0.011	low	oxic	486	218	0.0%	0.0%	100.0%
USAWS-20	Central California Foothills and Coastal Mountains	0.0300	Feb 8 2007	0.035	moderate	oxic	478	212	19.6%	0.0%	80.4%
USAWS-21	Central California Foothills and Coastal Mountains	0.0300	Feb 8 2007	0.060	moderate	anoxic	451	274	65.3%	25.8%	8.9%
USAWY-04[2]	Central California Foothills and Coastal Mountains	0.0300	Dec 13 2006	0.013	low	na	633	521	0.0%	0.0%	100.0%
USAWY-05	Central California Foothills and Coastal Mountains	0.0300	Jan 8 2007	0.026	low	oxic	745	180	0.0%	0.0%	100.0%
USAWY-06	Central California Foothills and Coastal Mountains	0.0300	Jan 9 2007	0.024	low	oxic	745	241	0.5%	12.2%	87.3%
SCRV-03	Central California Foothills and Coastal Mountains	0.0300	Apr 2 2007	0.047	moderate	anoxic	7	312	90.8%	4.1%	5.1%
SCRV-04[2]	Southern California Mountains	0.0109	Apr 3 2007	0.013	moderate	oxic	316	na	30.5%	15.9%	53.6%
SCRV-06	Central California Foothills and Coastal Mountains	0.0300	Apr 4 2007	0.048	moderate	anoxic	17	67	0.0%	0.0%	100.0%
SCRV-07	Southern California Mountains	0.0109	Apr 4 2007	0.010	low	oxic	180	74	6.0%	2.3%	91.7%
SCRV-09	Central California Foothills and Coastal Mountains	0.0300	Apr 5 2007	0.046	moderate	anoxic	58	263	86.7%	1.8%	11.5%
SCRV-10	Central California Foothills and Coastal Mountains	0.0300	Apr 5 2007	0.031	moderate	anoxic	2	233	0.0%	8.8%	91.2%
SCRV-12	Central California Foothills and Coastal Mountains	0.0300	Apr 9 2007	0.034	moderate	anoxic	6	366	0.0%	0.0%	100.0%
SCRV-16	Central California Foothills and Coastal Mountains	0.0300	Apr 10 2007	0.102	high	anoxic	86	213	7.2%	21.7%	71.0%
SCRV-19	Central California Foothills and Coastal Mountains	0.0300	Apr 11 2007	0.052	moderate	oxic	144	33	78.1%	21.5%	0.5%
SCRV-27	Central California Foothills and Coastal Mountains	0.0300	Apr 16 2007	0.035	moderate	anoxic	19	266	38.7%	7.4%	53.9%
SCRV-28	Central California Foothills and Coastal Mountains	0.0300	Apr 17 2007	0.023	low	oxic	136	102	80.6%	10.6%	8.8%
SCRV-33	Central California Foothills and Coastal Mountains	0.0300	Apr 18 2007	0.044	moderate	oxic	37	154	74.3%	0.0%	25.7%
SCRV-35	Central California Foothills and Coastal Mountains	0.0300	Apr 19 2007	0.027	low	oxic	234	91	0.0%	0.0%	100.0%
SCRV-37	Central California Foothills and Coastal Mountains	0.0300	May 8 2007	0.040	moderate	anoxic	3	165	17.5%	59.0%	23.5%
SCRV-39	Central California Foothills and Coastal Mountains	0.0300	May 14 2007	0.067	moderate	anoxic	75	363	15.4%	0.0%	84.6%
SCRV-42	Central California Foothills and Coastal Mountains	0.0300	Jun 6 2007	0.071	moderate	oxic	195	87	43.1%	38.0%	19.0%
HR-PR-01	Central California Foothills and Coastal Mountains	0.0300	Aug 22 2011	0.174	high	anoxic	634	14	0.0%	91.8%	8.2%
HR-PR-02[2]	Central California Foothills and Coastal Mountains	0.0300	Aug 23 2011	0.040	moderate	oxic	1,184	68	0.0%	94.5%	5.5%
HR-PR-04[2]	Southern California Mountains	0.0109	Aug 24 2011	0.020	moderate	anoxic	871	85	0.0%	98.2%	1.8%
HR-PR-06	Central California Foothills and Coastal Mountains	0.0300	Aug 25 2011	0.009	low	anoxic	951	152	0.0%	99.1%	0.9%
HR-PR-08[2]	Central California Foothills and Coastal Mountains	0.0300	Aug 30 2011	0.015	low	oxic	696	171	6.8%	87.7%	5.5%

Online resource (suppplementary table) 2. Selected attributes of GAMA-PBP (https://ca.water.usgs.gov/gama/) status wells sampled for orthophosphate concentration-page 105.

GAMA-PBP ID	Agricultural land use in 1982[9] (percent)	Natural land use in 1982[9] (percent)	Urban land use in 1982[9] (percent)	Agricultural land use in 1992[9] (percent)	Natural land use in 1992[9] (percent)	Urban land use in 1992[9] (percent)	Agricultural land use in 2002[9] (percent)	Natural land use in 2002[9] (percent)	Urban land use in 2002[9] (percent)	Agricultural land use in 2012[9] (percent)	Natural land use in 2012[9] (percent)	Urban land use in 2012[9] (percent)	Age Classification[10]	Septic Tanks[11]	Aridity[12]
USAWS-14	15.0%	0.0%	85.0%	7.9%	0.0%	92.1%	1.9%	0.0%	98.1%	0.0%	0.0%	100.0%	Mixed	14.51	0.230
USAWS-15	2.3%	0.0%	97.7%	0.0%	0.0%	100.0%	0.0%	0.0%	100.0%	0.0%	0.0%	100.0%	Premodern	2.24	0.221
USAWS-16[2]	69.3%	28.0%	2.8%	67.4%	28.4%	4.1%	63.3%	25.7%	11.0%	63.3%	21.1%	15.6%	Mixed	6.58	0.228
USAWS-18	0.0%	0.0%	100.0%	0.0%	0.0%	100.0%	0.0%	0.0%	100.0%	0.0%	0.0%	100.0%	Premodern	11.87	0.224
USAWS-20	0.5%	0.0%	99.5%	0.0%	0.0%	100.0%	0.0%	0.0%	100.0%	0.0%	0.0%	100.0%	Mixed	3.27	0.222
USAWS-21	64.8%	24.4%	10.8%	62.9%	23.0%	14.1%	60.1%	17.8%	22.1%	60.1%	1.4%	38.5%	Mixed	1.81	0.228
USAWY-04[2]	0.0%	0.0%	100.0%	0.0%	0.0%	100.0%	0.0%	0.0%	100.0%	0.0%	0.0%	100.0%	Premodern	194.85	0.269
USAWY-05	0.0%	0.0%	100.0%	0.0%	0.0%	100.0%	0.0%	0.0%	100.0%	0.0%	0.0%	100.0%	Mixed	15.87	0.329
USAWY-06	0.5%	12.2%	87.3%	0.5%	12.2%	87.3%	0.5%	12.2%	87.3%	0.5%	12.2%	87.3%	Modern	23.84	0.312
SCRV-03	90.8%	4.1%	5.1%	88.5%	3.7%	7.8%	87.6%	3.7%	8.8%	87.6%	3.7%	8.8%	Premodern	1.19	0.260
SCRV-04[2]	30.5%	15.9%	53.6%	30.5%	15.9%	53.6%	24.5%	17.3%	58.2%	40.0%	11.4%	48.6%	ModernOrMixed	40.65	0.441
SCRV-06	0.0%	0.0%	100.0%	0.0%	0.0%	100.0%	0.0%	0.0%	100.0%	0.0%	0.0%	100.0%	ModernOrMixed	12.94	0.284
SCRV-07	13.9%	2.3%	83.8%	8.3%	2.3%	89.4%	7.9%	1.9%	90.3%	7.9%	1.9%	90.3%	Modern	16.99	0.358
SCRV-09	80.7%	0.9%	18.3%	80.7%	0.9%	18.3%	78.4%	0.9%	20.6%	78.4%	0.5%	21.1%	PremodernOrMixed	1.98	0.324
SCRV-10	0.0%	8.8%	91.2%	0.0%	8.8%	91.2%	0.0%	8.8%	91.2%	0.0%	8.8%	91.2%	Premodern	0.12	0.262
SCRV-12	0.0%	0.0%	100.0%	0.0%	0.0%	100.0%	0.0%	0.0%	100.0%	0.0%	0.0%	100.0%	Mixed	0.00	0.266
SCRV-16	5.9%	21.7%	72.4%	4.5%	21.3%	74.2%	4.5%	21.3%	74.2%	2.7%	22.2%	75.1%	ModernOrMixed	3.80	0.308
SCRV-19	78.1%	21.5%	0.5%	78.1%	21.5%	0.5%	78.1%	21.5%	0.5%	78.1%	21.5%	0.5%	ModernOrMixed	1.12	0.327
SCRV-27	30.9%	5.5%	63.6%	29.0%	5.5%	65.4%	28.6%	5.5%	65.9%	12.0%	1.8%	86.2%	ModernOrMixed	0.00	0.281
SCRV-28	83.3%	7.9%	8.8%	82.9%	7.4%	9.7%	81.9%	6.5%	11.6%	81.9%	6.5%	11.6%	ModernOrMixed	5.37	0.321
SCRV-33	64.7%	0.0%	35.3%	61.5%	0.0%	38.5%	60.6%	0.0%	39.4%	60.6%	0.0%	39.4%	ModernOrMixed	1.22	0.272
SCRV-35	0.0%	0.0%	100.0%	0.0%	0.0%	100.0%	0.0%	0.0%	100.0%	0.0%	0.0%	100.0%	Mixed	18.27	0.285
SCRV-37	17.5%	59.0%	23.5%	17.5%	59.0%	23.5%	17.5%	59.0%	23.5%	17.5%	10.1%	72.4%	PremodernOrMixed	1.90	0.254
SCRV-39	3.7%	0.0%	96.3%	0.5%	0.0%	99.5%	0.5%	0.0%	99.5%	0.5%	0.0%	99.5%	PremodernOrMixed	8.32	0.310
SCRV-42	44.0%	38.0%	18.1%	44.0%	38.0%	18.1%	43.5%	38.0%	18.5%	43.5%	35.6%	20.8%	ModernOrMixed	3.37	0.311
HR-PR-01	0.0%	91.3%	8.7%	0.0%	90.4%	9.6%	0.0%	90.4%	9.6%	0.0%	90.0%	10.0%	ModernOrMixed	5.95	0.381
HR-PR-02[2]	0.0%	94.5%	5.5%	0.0%	94.5%	5.5%	0.0%	94.5%	5.5%	0.0%	94.5%	5.5%	na	5.06	0.282
HR-PR-04[2]	0.0%	98.2%	1.8%	0.0%	98.2%	1.8%	0.0%	98.2%	1.8%	0.0%	98.2%	1.8%	na	0.60	0.523
HR-PR-06	0.0%	99.1%	0.9%	0.0%	99.1%	0.9%	0.0%	98.2%	1.8%	0.0%	98.2%	1.8%	ModernOrMixed	1.93	0.348
HR-PR-08[2]	6.8%	87.7%	5.5%	6.8%	87.2%	5.9%	6.8%	86.8%	6.4%	6.8%	86.8%	6.4%	na	0.42	0.373

Online resource (suppplementary table) 2. Selected attributes of GAMA-PBP (https://ca.water.usgs.gov/gama/) status wells sampled for orthophosphate concentration-page 106.

GAMA-PBP ID	USGS Station ID[1]	GAMA-PBP study unit	GAMA-PBP study area[3]	Hydrogeologic zone
HR-PR-09[2]	325700116350001	Santa Cruz, San Gabriel, and Peninsular Ranges Hard Rock Aquifers	Hard Rock Peninsular Ranges	Southern California
HR-PR-10[2]	330200116370001	Santa Cruz, San Gabriel, and Peninsular Ranges Hard Rock Aquifers	Hard Rock Peninsular Ranges	Southern California
HR-PR-11[2]	330300116540001	Santa Cruz, San Gabriel, and Peninsular Ranges Hard Rock Aquifers	Hard Rock Peninsular Ranges	Southern California
HR-PR-12[2]	332335117111501	Santa Cruz, San Gabriel, and Peninsular Ranges Hard Rock Aquifers	Hard Rock Peninsular Ranges	Southern California
HR-PR-13[2]	333400116590001	Santa Cruz, San Gabriel, and Peninsular Ranges Hard Rock Aquifers	Hard Rock Peninsular Ranges	Southern California
HR-PR-14[2]	324300116400001	Santa Cruz, San Gabriel, and Peninsular Ranges Hard Rock Aquifers	Hard Rock Peninsular Ranges	Southern California
HR-PR-15[2]	331900116510001	Santa Cruz, San Gabriel, and Peninsular Ranges Hard Rock Aquifers	Hard Rock Peninsular Ranges	Southern California
HR-PR-16[2]	323905116512001	Santa Cruz, San Gabriel, and Peninsular Ranges Hard Rock Aquifers	Hard Rock Peninsular Ranges	Southern California
HR-PR-17[2]	323800116290001	Santa Cruz, San Gabriel, and Peninsular Ranges Hard Rock Aquifers	Hard Rock Peninsular Ranges	Southern California
HR-PR-18	330957116594501	Santa Cruz, San Gabriel, and Peninsular Ranges Hard Rock Aquifers	Hard Rock Peninsular Ranges	Southern California
HR-PR-19[2]	324600116260001	Santa Cruz, San Gabriel, and Peninsular Ranges Hard Rock Aquifers	Hard Rock Peninsular Ranges	Southern California
HR-PR-20[2]	333600117300001	Santa Cruz, San Gabriel, and Peninsular Ranges Hard Rock Aquifers	Hard Rock Peninsular Ranges	Southern California
HR-PR-21[2]	324300116480001	Santa Cruz, San Gabriel, and Peninsular Ranges Hard Rock Aquifers	Hard Rock Peninsular Ranges	Southern California
HR-PR-22[2]	325100116330001	Santa Cruz, San Gabriel, and Peninsular Ranges Hard Rock Aquifers	Hard Rock Peninsular Ranges	Southern California
HR-PR-23[2]	325400116370001	Santa Cruz, San Gabriel, and Peninsular Ranges Hard Rock Aquifers	Hard Rock Peninsular Ranges	Southern California
HR-PR-24[2]	332700117170001	Santa Cruz, San Gabriel, and Peninsular Ranges Hard Rock Aquifers	Hard Rock Peninsular Ranges	Southern California
HR-PR-25[2]	333400117180001	Santa Cruz, San Gabriel, and Peninsular Ranges Hard Rock Aquifers	Hard Rock Peninsular Ranges	Southern California
HR-PR-26[2]	330400117090001	Santa Cruz, San Gabriel, and Peninsular Ranges Hard Rock Aquifers	Hard Rock Peninsular Ranges	Southern California
HR-PR-27	331122116380801	Santa Cruz, San Gabriel, and Peninsular Ranges Hard Rock Aquifers	Hard Rock Peninsular Ranges	Southern California
HR-PR-28[2]	333800116480001	Santa Cruz, San Gabriel, and Peninsular Ranges Hard Rock Aquifers	Hard Rock Peninsular Ranges	Southern California
HR-PR-29[2]	333600116260001	Santa Cruz, San Gabriel, and Peninsular Ranges Hard Rock Aquifers	Hard Rock Peninsular Ranges	Southern California
HR-PR-30[2]	334200116380001	Santa Cruz, San Gabriel, and Peninsular Ranges Hard Rock Aquifers	Hard Rock Peninsular Ranges	Southern California
HR-PR-31[2]	334500116420001	Santa Cruz, San Gabriel, and Peninsular Ranges Hard Rock Aquifers	Hard Rock Peninsular Ranges	Southern California
HR-PR-32[2]	333500116280001	Santa Cruz, San Gabriel, and Peninsular Ranges Hard Rock Aquifers	Hard Rock Peninsular Ranges	Southern California
HR-PR-33[2]	324000116170001	Santa Cruz, San Gabriel, and Peninsular Ranges Hard Rock Aquifers	Hard Rock Peninsular Ranges	Southern California
HR-PR-34[2]	324100116150001	Santa Cruz, San Gabriel, and Peninsular Ranges Hard Rock Aquifers	Hard Rock Peninsular Ranges	Southern California
HR-PR-35[2]	335200116470001	Santa Cruz, San Gabriel, and Peninsular Ranges Hard Rock Aquifers	Hard Rock Peninsular Ranges	Southern California
HR-PR-36[2]	334600116440002	Santa Cruz, San Gabriel, and Peninsular Ranges Hard Rock Aquifers	Hard Rock Peninsular Ranges	Southern California
HR-SG-01[2]	343300118280001	Santa Cruz, San Gabriel, and Peninsular Ranges Hard Rock Aquifers	Hard Rock San Gabriel	Southern California
HR-SG-02[2]	343800118300001	Santa Cruz, San Gabriel, and Peninsular Ranges Hard Rock Aquifers	Hard Rock San Gabriel	Southern California

Online resource (suppplementary table) 2. Selected attributes of GAMA-PBP (https://ca.water.usgs.gov/gama/) status wells sampled for orthophosphate concentration-page 107.

GAMA-PBP ID	USEPA Level III Ecoregions[4]	Level III Ecoregion Reference Concentration[4]	Status Sample Date	Status Sample Orthophosphate Concentration (mg/L as P)	Relative Concentration Category[5]	Redox state[6]	Elevation of LSD (meters above NAVD 88)[7]	Well depth (meters below LSD)[8]	Agricultural land use in 1974[9] (percent)	Natural land use in 1974[9] (percent)	Urban land use in 1974[9] (percent)
HR-PR-09[2]	Central California Foothills and Coastal Mountains	0.0300	Aug 31 2011	0.008	low	oxic	1,467	183	0.0%	95.9%	4.1%
HR-PR-10[2]	Southern California Mountains	0.0109	Sep 13 2011	0.010	low	oxic	1,252	152	0.0%	96.3%	3.7%
HR-PR-11[2]	Central California Foothills and Coastal Mountains	0.0300	Sep 13 2011	0.017	low	anoxic	470	298	0.0%	99.5%	0.5%
HR-PR-12[2]	Central California Foothills and Coastal Mountains	0.0300	Sep 14 2011	0.042	moderate	oxic	306	28	14.7%	65.1%	20.2%
HR-PR-13[2]	Central California Foothills and Coastal Mountains	0.0300	Sep 14 2011	0.104	high	anoxic	534	18	19.5%	74.1%	6.4%
HR-PR-14[2]	Central California Foothills and Coastal Mountains	0.0300	Sep 15 2011	0.027	low	oxic	503	107	0.0%	93.6%	6.4%
HR-PR-15[2]	Southern California Mountains	0.0109	Sep 20 2011	0.019	moderate	oxic	1,505	na	0.0%	99.5%	0.5%
HR-PR-16[2]	Central California Foothills and Coastal Mountains	0.0300	Sep 21 2011	0.007	low	anoxic	201	152	0.0%	97.3%	2.7%
HR-PR-17[2]	Central California Foothills and Coastal Mountains	0.0300	Sep 21 2011	0.017	low	oxic	849	104	0.0%	89.4%	10.6%
HR-PR-18	Central California Foothills and Coastal Mountains	0.0300	Sep 22 2011	0.012	low	anoxic	463	139	0.0%	94.9%	5.1%
HR-PR-19[2]	Central California Foothills and Coastal Mountains	0.0300	Oct 3 2011	0.017	low	oxic	1,258	61	0.0%	98.6%	1.4%
HR-PR-20[2]	Central California Foothills and Coastal Mountains	0.0300	Oct 4 2011	0.020	low	oxic	284	32	0.0%	100.0%	0.0%
HR-PR-21[2]	Central California Foothills and Coastal Mountains	0.0300	Oct 17 2011	0.014	low	oxic	494	101	0.0%	96.8%	3.2%
HR-PR-22[2]	Central California Foothills and Coastal Mountains	0.0300	Oct 31 2011	0.009	low	oxic	1,230	174	0.0%	96.8%	3.2%
HR-PR-23[2]	Central California Foothills and Coastal Mountains	0.0300	Oct 31 2011	0.014	low	anoxic	970	214	0.0%	99.1%	0.9%
HR-PR-24[2]	Central California Foothills and Coastal Mountains	0.0300	Nov 1 2011	0.146	high	oxic	161	66	5.0%	93.6%	1.4%
HR-PR-25[2]	Central California Foothills and Coastal Mountains	0.0300	Nov 1 2011	0.020	low	oxic	682	85	0.0%	95.9%	4.1%
HR-PR-26[2]	Central California Foothills and Coastal Mountains	0.0300	Nov 2 2011	0.019	low	anoxic	130	93	0.0%	12.3%	87.7%
HR-PR-27	Southern California Mountains	0.0109	Nov 3 2011	0.035	moderate	oxic	1,145	57	0.0%	100.0%	0.0%
HR-PR-28[2]	Central California Foothills and Coastal Mountains	0.0300	Nov 14 2011	0.014	low	oxic	874	127	0.0%	99.5%	0.5%
HR-PR-29[2]	Southern California Mountains	0.0109	Nov 15 2011	0.004	low	anoxic	1,237	427	0.0%	94.1%	5.9%
HR-PR-30[2]	Southern California Mountains	0.0109	Nov 16 2011	0.018	moderate	oxic	1,576	108	0.0%	98.6%	1.4%
HR-PR-31[2]	Southern California Mountains	0.0109	Nov 16 2011	0.012	moderate	oxic	1,736	197	0.0%	0.0%	100.0%
HR-PR-32[2]	Southern California Mountains	0.0109	Nov 17 2011	0.008	low	oxic	1,174	94	0.0%	99.5%	0.5%
HR-PR-33[2]	Central California Foothills and Coastal Mountains	0.0300	Nov 28 2011	0.068	moderate	oxic	1,086	na	0.0%	79.1%	20.9%
HR-PR-34[2]	Central California Foothills and Coastal Mountains	0.0300	Nov 28 2011	0.140	high	oxic	1,059	23	0.5%	99.1%	0.5%
HR-PR-35[2]	Southern California Mountains	0.0109	Nov 29 2011	0.021	moderate	oxic	1,061	305	5.5%	91.2%	3.2%
HR-PR-36[2]	Southern California Mountains	0.0109	Nov 29 2011	0.004	low	oxic	1,766	152	0.0%	98.2%	1.8%
HR-SG-01[2]	Southern California Mountains	0.0109	May 16 2011	0.027	moderate	oxic	557	na	1.8%	95.0%	3.2%
HR-SG-02[2]	Southern California Mountains	0.0109	May 16 2011	0.027	moderate	oxic	828	na	0.0%	95.0%	5.0%

Status and trends of orthophosphate concentrations in groundwater used for public supply in California *Environmental Monitoring and Assessment, Robert Kent, Tyler D. Johnson, and Michael R. Rosen, U.S. Geological Survey California Water Science Center-rhkent@usgs.gov*

Online resource (suppplementary table) 2. Selected attributes of GAMA-PBP (https://ca.water.usgs.gov/gama/) status wells sampled for orthophosphate concentration-page 108.

GAMA-PBP ID	Agricultural land use in 1982[9] (percent)	Natural land use in 1982[9] (percent)	Urban land use in 1982[9] (percent)	Agricultural land use in 1992[9] (percent)	Natural land use in 1992[9] (percent)	Urban land use in 1992[9] (percent)	Agricultural land use in 2002[9] (percent)	Natural land use in 2002[9] (percent)	Urban land use in 2002[9] (percent)	Agricultural land use in 2012[9] (percent)	Natural land use in 2012[9] (percent)	Urban land use in 2012[9] (percent)	Age Classification[10]	Septic Tanks[11]	Aridity[12]
HR-PR-09[2]	0.0%	95.9%	4.1%	0.0%	95.4%	4.6%	5.0%	90.0%	5.0%	5.0%	89.5%	5.5%	na	0.37	0.715
HR-PR-10[2]	0.0%	96.3%	3.7%	0.0%	96.3%	3.7%	0.0%	96.3%	3.7%	0.0%	96.3%	3.7%	na	11.24	0.593
HR-PR-11[2]	0.0%	99.1%	0.9%	0.0%	98.6%	1.4%	0.0%	98.6%	1.4%	0.0%	98.6%	1.4%	na	7.22	0.310
HR-PR-12[2]	17.0%	50.9%	32.1%	9.6%	6.9%	83.5%	5.5%	6.9%	87.6%	5.5%	6.9%	87.6%	na	17.42	0.314
HR-PR-13[2]	22.7%	70.9%	6.4%	16.8%	75.5%	7.7%	16.8%	73.6%	9.5%	16.8%	73.2%	10.0%	na	2.72	0.274
HR-PR-14[2]	0.0%	93.2%	6.8%	0.0%	92.3%	7.7%	0.0%	91.8%	8.2%	0.0%	91.8%	8.2%	na	2.73	0.345
HR-PR-15[2]	0.0%	99.5%	0.5%	0.0%	99.5%	0.5%	0.0%	99.5%	0.5%	0.0%	99.5%	0.5%	na	0.66	0.605
HR-PR-16[2]	0.0%	96.8%	3.2%	0.0%	96.8%	3.2%	0.0%	96.8%	3.2%	0.0%	96.8%	3.2%	na	2.08	0.275
HR-PR-17[2]	0.9%	88.5%	10.6%	0.0%	89.0%	11.0%	0.0%	89.0%	11.0%	0.0%	89.0%	11.0%	na	1.78	0.302
HR-PR-18	0.0%	93.0%	7.0%	0.0%	92.6%	7.4%	0.0%	92.1%	7.9%	0.0%	92.1%	7.9%	ModernOrMixed	2.97	0.359
HR-PR-19[2]	0.0%	98.6%	1.4%	0.0%	98.6%	1.4%	0.0%	98.6%	1.4%	0.0%	98.6%	1.4%	na	0.94	0.469
HR-PR-20[2]	0.0%	100.0%	0.0%	0.0%	100.0%	0.0%	0.0%	100.0%	0.0%	0.0%	100.0%	0.0%	na	0.00	0.341
HR-PR-21[2]	0.0%	96.3%	3.7%	0.0%	95.9%	4.1%	0.0%	95.9%	4.1%	0.0%	95.9%	4.1%	na	2.95	0.353
HR-PR-22[2]	0.0%	96.8%	3.2%	0.0%	94.0%	6.0%	0.0%	94.0%	6.0%	0.0%	94.0%	6.0%	na	4.58	0.529
HR-PR-23[2]	0.0%	99.1%	0.9%	0.0%	99.1%	0.9%	0.0%	98.1%	1.9%	0.0%	97.7%	2.3%	na	5.61	0.448
HR-PR-24[2]	7.3%	91.4%	1.4%	5.0%	93.6%	1.4%	32.3%	65.5%	2.3%	32.3%	65.0%	2.7%	na	1.91	0.257
HR-PR-25[2]	0.0%	95.9%	4.1%	0.0%	94.9%	5.1%	0.0%	93.5%	6.5%	0.0%	93.1%	6.9%	na	5.05	0.263
HR-PR-26[2]	0.0%	12.3%	87.7%	0.0%	12.3%	87.7%	0.0%	7.7%	92.3%	0.0%	0.0%	100.0%	na	14.23	0.278
HR-PR-27	0.0%	100.0%	0.0%	0.0%	100.0%	0.0%	0.0%	100.0%	0.0%	0.0%	100.0%	0.0%	ModernOrMixed	0.58	0.495
HR-PR-28[2]	0.0%	99.5%	0.5%	0.0%	98.2%	1.8%	0.0%	97.3%	2.7%	0.0%	97.3%	2.7%	na	1.41	0.307
HR-PR-29[2]	0.0%	94.1%	5.9%	0.0%	94.1%	5.9%	0.0%	93.2%	6.8%	0.0%	93.2%	6.8%	na	3.93	0.245
HR-PR-30[2]	0.0%	98.6%	1.4%	0.0%	98.6%	1.4%	0.0%	98.1%	1.9%	0.0%	98.1%	1.9%	na	5.92	0.477
HR-PR-31[2]	0.0%	0.0%	100.0%	0.0%	0.0%	100.0%	0.0%	0.0%	100.0%	0.0%	0.0%	100.0%	na	89.27	0.555
HR-PR-32[2]	0.0%	99.5%	0.5%	0.0%	99.5%	0.5%	0.0%	99.5%	0.5%	0.0%	99.5%	0.5%	na	3.93	0.259
HR-PR-33[2]	0.0%	79.1%	20.9%	0.0%	79.1%	20.9%	0.0%	78.6%	21.4%	0.0%	78.6%	21.4%	na	0.94	0.333
HR-PR-34[2]	9.2%	90.4%	0.5%	0.0%	99.5%	0.5%	0.0%	98.6%	1.4%	0.0%	98.2%	1.8%	na	0.94	0.310
HR-PR-35[2]	5.5%	90.8%	3.7%	5.1%	91.2%	3.7%	5.5%	90.8%	3.7%	5.5%	90.8%	3.7%	na	2.05	0.357
HR-PR-36[2]	0.0%	98.2%	1.8%	0.0%	98.2%	1.8%	0.0%	98.2%	1.8%	0.0%	98.2%	1.8%	na	9.71	0.504
HR-SG-01[2]	1.8%	95.0%	3.2%	1.8%	94.1%	4.1%	1.8%	94.1%	4.1%	1.8%	93.6%	4.5%	na	0.54	0.318
HR-SG-02[2]	0.0%	95.0%	5.0%	0.0%	95.0%	5.0%	0.0%	95.0%	5.0%	0.0%	95.0%	5.0%	na	1.85	0.389

Online resource (suppplementary table) 2. Selected attributes of GAMA-PBP (https://ca.water.usgs.gov/gama/) status wells sampled for orthophosphate concentration-page 109.

GAMA-PBP ID	USGS Station ID[1]	GAMA-PBP study unit	GAMA-PBP study area[3]	Hydrogeologic zone
HR-SG-03[2]	342600118170001	Santa Cruz, San Gabriel, and Peninsular Ranges Hard Rock Aquifers	Hard Rock San Gabriel	Southern California
HR-SG-04[2]	343000118210001	Santa Cruz, San Gabriel, and Peninsular Ranges Hard Rock Aquifers	Hard Rock San Gabriel	Southern California
HR-SG-05[2]	344000118270001	Santa Cruz, San Gabriel, and Peninsular Ranges Hard Rock Aquifers	Hard Rock San Gabriel	Southern California
HR-SG-06[2]	341400118130001	Santa Cruz, San Gabriel, and Peninsular Ranges Hard Rock Aquifers	Hard Rock San Gabriel	Southern California
HR-SG-07[2]	341600118090001	Santa Cruz, San Gabriel, and Peninsular Ranges Hard Rock Aquifers	Hard Rock San Gabriel	Southern California
HR-SG-08[2]	342600118190001	Santa Cruz, San Gabriel, and Peninsular Ranges Hard Rock Aquifers	Hard Rock San Gabriel	Southern California
HR-SG-09[2]	342800118280001	Santa Cruz, San Gabriel, and Peninsular Ranges Hard Rock Aquifers	Hard Rock San Gabriel	Southern California
HR-SG-10[2]	344400118350001	Santa Cruz, San Gabriel, and Peninsular Ranges Hard Rock Aquifers	Hard Rock San Gabriel	Southern California
HR-SG-11[2]	344200118330001	Santa Cruz, San Gabriel, and Peninsular Ranges Hard Rock Aquifers	Hard Rock San Gabriel	Southern California
HR-SG-12[2]	341400118030001	Santa Cruz, San Gabriel, and Peninsular Ranges Hard Rock Aquifers	Hard Rock San Gabriel	Southern California
HR-SG-13[2]	342300117430001	Santa Cruz, San Gabriel, and Peninsular Ranges Hard Rock Aquifers	Hard Rock San Gabriel	Southern California
HR-SG-14[2]	342200117430001	Santa Cruz, San Gabriel, and Peninsular Ranges Hard Rock Aquifers	Hard Rock San Gabriel	Southern California
HR-SG-15[2]	341900117490001	Santa Cruz, San Gabriel, and Peninsular Ranges Hard Rock Aquifers	Hard Rock San Gabriel	Southern California
HR-SG-16[2]	341100118010001	Santa Cruz, San Gabriel, and Peninsular Ranges Hard Rock Aquifers	Hard Rock San Gabriel	Southern California
HR-SG-17[2]	342000117580001	Santa Cruz, San Gabriel, and Peninsular Ranges Hard Rock Aquifers	Hard Rock San Gabriel	Southern California
HR-SG-18[2]	341900118070001	Santa Cruz, San Gabriel, and Peninsular Ranges Hard Rock Aquifers	Hard Rock San Gabriel	Southern California
HR-SG-19[2]	342300118040001	Santa Cruz, San Gabriel, and Peninsular Ranges Hard Rock Aquifers	Hard Rock San Gabriel	Southern California
HR-SG-20[2]	342400117490001	Santa Cruz, San Gabriel, and Peninsular Ranges Hard Rock Aquifers	Hard Rock San Gabriel	Southern California
HR-SG-21[2]	342200118100001	Santa Cruz, San Gabriel, and Peninsular Ranges Hard Rock Aquifers	Hard Rock San Gabriel	Southern California
HR-SG-22[2]	343000118270001	Santa Cruz, San Gabriel, and Peninsular Ranges Hard Rock Aquifers	Hard Rock San Gabriel	Southern California
HR-SG-23[2]	341300117290001	Santa Cruz, San Gabriel, and Peninsular Ranges Hard Rock Aquifers	Hard Rock San Gabriel	Southern California
HR-SG-24[2]	341900118190002	Santa Cruz, San Gabriel, and Peninsular Ranges Hard Rock Aquifers	Hard Rock San Gabriel	Southern California
HR-SG-25[2]	341300117250001	Santa Cruz, San Gabriel, and Peninsular Ranges Hard Rock Aquifers	Hard Rock San Gabriel	Southern California
HR-SG-26[2]	341400117490001	Santa Cruz, San Gabriel, and Peninsular Ranges Hard Rock Aquifers	Hard Rock San Gabriel	Southern California
HR-SG-27[2]	342000117590001	Santa Cruz, San Gabriel, and Peninsular Ranges Hard Rock Aquifers	Hard Rock San Gabriel	Southern California
HR-SG-28[2]	341000117340001	Santa Cruz, San Gabriel, and Peninsular Ranges Hard Rock Aquifers	Hard Rock San Gabriel	Southern California
HR-SG-29[2]	341600117360001	Santa Cruz, San Gabriel, and Peninsular Ranges Hard Rock Aquifers	Hard Rock San Gabriel	Southern California
HR-SG-30[2]	341600117370001	Santa Cruz, San Gabriel, and Peninsular Ranges Hard Rock Aquifers	Hard Rock San Gabriel	Southern California
HR-SG-31[2]	343600118260001	Santa Cruz, San Gabriel, and Peninsular Ranges Hard Rock Aquifers	Hard Rock San Gabriel	Southern California
HR-SG-32[2]	341800118010001	Santa Cruz, San Gabriel, and Peninsular Ranges Hard Rock Aquifers	Hard Rock San Gabriel	Southern California
HR-SG-33[2]	342900118190001	Santa Cruz, San Gabriel, and Peninsular Ranges Hard Rock Aquifers	Hard Rock San Gabriel	Southern California

Status and trends of orthophosphate concentrations in groundwater used for public supply in California *Environmental Monitoring and Assessment, Robert Kent, Tyler D. Johnson, and Michael R. Rosen, U.S. Geological Survey California Water Science Center-rhkent@usgs.gov*

Online resource (suppplementary table) 2. Selected attributes of GAMA-PBP (https://ca.water.usgs.gov/gama/) status wells sampled for orthophosphate concentration-page 110.

GAMA-PBP ID	USEPA Level III Ecoregions[4]	Level III Ecoregion Reference Concentration[4]	Status Sample Date	Status Sample Orthophosphate Concentration (mg/L as P)	Relative Concentration Category[5]	Redox state[6]	Elevation of LSD (meters above NAVD 88)[7]	Well depth (meters below LSD)[8]	Agricultural land use in 1974[9] (percent)	Natural land use in 1974[9] (percent)	Urban land use in 1974[9] (percent)
HR-SG-03[2]	Southern California Mountains	0.0109	May 17 2011	0.058	moderate	oxic	657	na	3.7%	94.5%	1.8%
HR-SG-04[2]	Southern California Mountains	0.0109	May 18 2011	0.041	moderate	oxic	721	27	0.0%	69.5%	30.5%
HR-SG-05[2]	Mojave Basin and Range	0.0100	May 19 2011	0.019	moderate	oxic	1,012	245	8.7%	82.2%	9.1%
HR-SG-06[2]	Southern California Mountains	0.0109	May 23 2011	0.005	low	oxic	859	0	0.0%	16.1%	83.9%
HR-SG-07[2]	Southern California Mountains	0.0109	May 23 2011	0.004	low	anoxic	1,025	0	0.0%	98.6%	1.4%
HR-SG-08[2]	Southern California Mountains	0.0109	May 24 2011	0.115	high	anoxic	609	244	0.0%	100.0%	0.0%
HR-SG-09[2]	Southern California Mountains	0.0109	May 25 2011	0.034	moderate	oxic	449	183	19.2%	32.9%	47.9%
HR-SG-10[2]	Southern California Mountains	0.0109	May 25 2011	0.010	low	oxic	1,047	na	5.9%	82.7%	11.4%
HR-SG-11[2]	Southern California Mountains	0.0109	May 26 2011	0.006	low	oxic	1,256	183	6.4%	91.3%	2.3%
HR-SG-12[2]	Southern California Mountains	0.0109	Jul 11 2011	0.014	moderate	oxic	1,560	0	0.0%	100.0%	0.0%
HR-SG-13[2]	Southern California Mountains	0.0109	Jul 12 2011	0.012	moderate	oxic	1,879	112	0.0%	100.0%	0.0%
HR-SG-14[2]	Southern California Mountains	0.0109	Jul 12 2011	0.013	moderate	anoxic	2,222	81	0.0%	97.3%	2.7%
HR-SG-15[2]	Southern California Mountains	0.0109	Jul 13 2011	0.006	low	oxic	1,720	76	0.0%	95.9%	4.1%
HR-SG-16[2]	Southern California Mountains	0.0109	Jul 13 2011	0.011	low	oxic	827	107	0.0%	99.5%	0.5%
HR-SG-17[2]	Southern California Mountains	0.0109	Jul 14 2011	0.019	moderate	oxic	1,891	0	0.0%	97.3%	2.7%
HR-SG-18[2]	Southern California Mountains	0.0109	Jul 18 2011	0.028	moderate	oxic	1,017	34	0.0%	99.5%	0.5%
HR-SG-19[2]	Southern California Mountains	0.0109	Jul 18 2011	0.016	moderate	oxic	1,440	0	0.0%	99.5%	0.5%
HR-SG-20[2]	Southern California Mountains	0.0109	Jul 19 2011	0.009	low	oxic	1,301	na	0.0%	100.0%	0.0%
HR-SG-21[2]	Southern California Mountains	0.0109	Jul 20 2011	0.023	moderate	oxic	1,343	0	0.0%	100.0%	0.0%
HR-SG-22[2]	Southern California Mountains	0.0109	Jul 21 2011	0.045	moderate	anoxic	506	26	0.5%	97.3%	2.3%
HR-SG-23[2]	Southern California Mountains	0.0109	Jul 25 2011	0.015	moderate	oxic	876	na	1.8%	89.0%	9.2%
HR-SG-24[2]	Southern California Mountains	0.0109	Jul 26 2011	0.012	moderate	oxic	722	na	0.0%	100.0%	0.0%
HR-SG-25[2]	Southern California Mountains	0.0109	Jul 27 2011	0.044	moderate	oxic	711	0	0.0%	93.6%	6.4%
HR-SG-26[2]	Southern California Mountains	0.0109	Jul 28 2011	0.012	moderate	oxic	456	na	0.0%	96.3%	3.7%
HR-SG-27[2]	Southern California Mountains	0.0109	Aug 1 2011	0.038	moderate	oxic	1,843	244	0.0%	97.7%	2.3%
HR-SG-28[2]	Central California Foothills and Coastal Mountains	0.0300	Aug 2 2011	0.008	low	oxic	871	0	0.0%	98.6%	1.4%
HR-SG-29[2]	Southern California Mountains	0.0109	Aug 3 2011	0.004	low	oxic	2,246	0	0.0%	85.7%	14.3%
HR-SG-30[2]	Southern California Mountains	0.0109	Aug 3 2011	0.004	low	oxic	2,353	0	0.0%	93.6%	6.4%
HR-SG-31[2]	Southern California Mountains	0.0109	Aug 17 2011	0.015	moderate	oxic	799	18	0.0%	98.6%	1.4%
HR-SG-32[2]	Southern California Mountains	0.0109	Aug 18 2011	0.008	low	anoxic	1,501	27	0.0%	100.0%	0.0%
HR-SG-33[2]	Southern California Mountains	0.0109	Jan 31 2012	0.091	moderate	oxic	707	57	0.9%	54.5%	44.5%

Online resource (suppplementary table) 2. Selected attributes of GAMA-PBP (https://ca.water.usgs.gov/gama/) status wells sampled for orthophosphate concentration-page 111.

GAMA-PBP ID	Agricultural land use in 1982[9] (percent)	Natural land use in 1982[9] (percent)	Urban land use in 1982[9] (percent)	Agricultural land use in 1992[9] (percent)	Natural land use in 1992[9] (percent)	Urban land use in 1992[9] (percent)	Agricultural land use in 2002[9] (percent)	Natural land use in 2002[9] (percent)	Urban land use in 2002[9] (percent)	Agricultural land use in 2012[9] (percent)	Natural land use in 2012[9] (percent)	Urban land use in 2012[9] (percent)	Age Classification[10]	Septic Tanks[11]	Aridity[12]
HR-SG-03[2]	3.7%	94.0%	2.3%	3.7%	94.0%	2.3%	3.7%	94.0%	2.3%	3.7%	94.0%	2.3%	na	3.85	0.285
HR-SG-04[2]	0.0%	69.5%	30.5%	0.0%	69.5%	30.5%	0.0%	69.5%	30.5%	0.0%	67.6%	32.4%	na	5.81	0.341
HR-SG-05[2]	8.7%	82.2%	9.1%	8.2%	82.6%	9.1%	8.2%	82.6%	9.1%	8.2%	82.6%	9.1%	na	0.59	0.354
HR-SG-06[2]	0.0%	16.1%	83.9%	0.0%	16.1%	83.9%	0.0%	16.1%	83.9%	0.0%	16.1%	83.9%	na	0.02	0.471
HR-SG-07[2]	0.0%	98.6%	1.4%	0.0%	98.6%	1.4%	0.0%	98.6%	1.4%	0.0%	98.6%	1.4%	na	0.19	0.527
HR-SG-08[2]	0.0%	99.5%	0.5%	0.0%	99.1%	0.9%	0.0%	96.3%	3.7%	0.0%	96.3%	3.7%	na	3.59	0.294
HR-SG-09[2]	19.2%	32.9%	47.9%	16.9%	32.4%	50.7%	16.9%	32.4%	50.7%	16.9%	31.1%	52.1%	na	3.10	0.297
HR-SG-10[2]	5.9%	82.7%	11.4%	5.0%	83.6%	11.4%	5.0%	83.6%	11.4%	5.0%	83.6%	11.4%	na	0.59	0.326
HR-SG-11[2]	6.4%	91.3%	2.3%	0.5%	97.3%	2.3%	0.5%	97.3%	2.3%	0.5%	97.3%	2.3%	na	0.59	0.488
HR-SG-12[2]	0.0%	100.0%	0.0%	0.0%	100.0%	0.0%	0.0%	100.0%	0.0%	0.0%	100.0%	0.0%	na	0.32	0.796
HR-SG-13[2]	0.0%	100.0%	0.0%	0.0%	100.0%	0.0%	0.0%	100.0%	0.0%	0.0%	100.0%	0.0%	na	0.87	0.428
HR-SG-14[2]	0.0%	96.8%	3.2%	0.0%	95.9%	4.1%	0.0%	95.4%	4.6%	0.0%	95.4%	4.6%	na	0.57	0.470
HR-SG-15[2]	0.0%	95.9%	4.1%	0.0%	95.9%	4.1%	0.0%	95.9%	4.1%	0.0%	95.9%	4.1%	na	0.14	0.714
HR-SG-16[2]	0.0%	99.5%	0.5%	0.0%	99.5%	0.5%	0.0%	99.5%	0.5%	0.0%	99.5%	0.5%	na	0.32	0.601
HR-SG-17[2]	0.0%	96.4%	3.6%	0.0%	95.5%	4.5%	0.0%	95.0%	5.0%	0.0%	95.0%	5.0%	na	0.37	0.823
HR-SG-18[2]	0.0%	99.5%	0.5%	0.0%	99.5%	0.5%	0.0%	99.5%	0.5%	0.0%	99.5%	0.5%	na	0.16	0.478
HR-SG-19[2]	0.0%	99.5%	0.5%	0.0%	99.5%	0.5%	0.0%	99.5%	0.5%	0.0%	99.5%	0.5%	na	1.18	0.480
HR-SG-20[2]	0.0%	100.0%	0.0%	0.0%	100.0%	0.0%	0.0%	100.0%	0.0%	0.0%	100.0%	0.0%	na	0.87	0.238
HR-SG-21[2]	0.0%	100.0%	0.0%	0.0%	100.0%	0.0%	0.0%	100.0%	0.0%	0.0%	100.0%	0.0%	na	1.18	0.399
HR-SG-22[2]	0.5%	97.3%	2.3%	0.5%	97.3%	2.3%	0.5%	97.3%	2.3%	0.5%	96.8%	2.7%	na	1.31	0.349
HR-SG-23[2]	1.8%	89.0%	9.2%	1.8%	89.0%	9.2%	1.8%	89.0%	9.2%	1.8%	89.0%	9.2%	na	0.31	0.766
HR-SG-24[2]	0.0%	100.0%	0.0%	0.0%	100.0%	0.0%	0.0%	100.0%	0.0%	0.0%	100.0%	0.0%	na	0.18	0.391
HR-SG-25[2]	0.0%	93.2%	6.8%	0.0%	93.2%	6.8%	0.0%	93.2%	6.8%	0.0%	92.7%	7.3%	na	0.96	0.600
HR-SG-26[2]	0.0%	96.3%	3.7%	0.0%	96.3%	3.7%	0.0%	96.3%	3.7%	0.0%	96.3%	3.7%	na	0.14	0.498
HR-SG-27[2]	0.0%	97.7%	2.3%	0.0%	97.7%	2.3%	0.0%	97.7%	2.3%	0.0%	97.7%	2.3%	na	0.81	0.713
HR-SG-28[2]	0.0%	95.9%	4.1%	0.0%	95.5%	4.5%	0.0%	95.5%	4.5%	0.0%	95.5%	4.5%	na	0.38	0.585
HR-SG-29[2]	0.0%	85.7%	14.3%	0.0%	83.9%	16.1%	0.0%	83.9%	16.1%	0.0%	83.9%	16.1%	na	0.38	0.860
HR-SG-30[2]	0.0%	93.6%	6.4%	0.0%	92.7%	7.3%	0.0%	92.7%	7.3%	0.0%	92.7%	7.3%	na	0.38	0.860
HR-SG-31[2]	0.0%	98.6%	1.4%	0.0%	98.6%	1.4%	0.0%	98.2%	1.8%	0.0%	98.2%	1.8%	na	2.48	0.383
HR-SG-32[2]	0.0%	100.0%	0.0%	0.0%	100.0%	0.0%	0.0%	100.0%	0.0%	0.0%	100.0%	0.0%	na	0.14	0.775
HR-SG-33[2]	0.9%	53.6%	45.5%	0.5%	52.7%	46.8%	0.5%	52.3%	47.3%	0.5%	51.4%	48.2%	na	11.06	0.266

Footnotes:

[1]Groundwater chemistry data are available through the USGS National Water Information System (NWIS) database at https://waterdata.usgs.gov/nwis. From there select "Water Quality", then "Field/Lab samples", and use these "Site Numbers" as "Site Identifier."

[2]Well not included in Principle Component Analysis (PCA) because data value for at least one parameter used in PCA was not available for this well.

[3]Descriptions and information for the 87 GAMA-PBP study areas can be found in Supporting Information table S1 of Belitz et al, 2015.

Belitz, K., Fram, M. S., & Johnson T. D. (2015). Metrics for assessing the quality of groundwater used for public supply, CA, USA: Equivalent population and area. *Environmental Science and Technology* , *49* (14), 8330-8338. doi: 10.1021/acs.est.5b00265

[4]USEPA Level III ecoregions and corresponding phosphorus reference concentrations are described in the following three publications:

U.S. Environmental Protection Agency. (2000a), Ambient water quality criteria recommendations, Information supporting the development of state and tribal nutrient criteria, Rivers and streams in nutrient ecoregion III: Washington, D.C., EPA 822-B-00-016, Office of Water, Office of Science and Technology, Health and Ecological Criteria Division [variously paged].

U.S. Environmental Protection Agency. (2000b), Ambient water quality criteria recommendations, Information supporting the development of state and tribal nutrient criteria, Rivers and streams in nutrient ecoregion II: Washington, D.C., EPA 822-B-00-015, Office of Water, Office of Science and Technology, Health and Ecological Criteria Division [variously paged].

U.S. Environmental Protection Agency. (2001), Ambient water quality criteria recommendations, Information supporting the development of state and tribal nutrient criteria, Rivers and streams in nutrient ecoregion I: Washington, D.C., EPA 822-B-01-012, Office of Water, Office of Science and Technology, Health and Ecological Criteria Division [variously paged].

[5]Relative Concentration Categories determined as follows: Orthophosphate concentrations that are less than the USEPA-determined level III ecoregion reference concentration are considered low. Concentrations between the ecor[illegible]

[6]Redox state of groundwater is considered anoxic if measured dissolved oxygen is less than 0.5 mg/L.

[7]Land-surface datum (LSD) is a datum that is approximately at land surface at each well. The elevation of the LSD is given in meters above the North American Vertical Datum 1988.

[8]Well depth given as distance from the elevation of the land-surface datum (LSD) given in the previous column. Springs are assigned a well depth of zero. Unknown well depths are assigned "na", not applicable.

[9]Land use data were represented as percentages of the broad categories, agricultural, natural, and urban in discrete years spanning five decades; 1974, 1982, 1992, 2002, and 2012 (Falcone, 2015).

Falcone, J. A. (2015). U.S. conterminous wall-to-wall anthropogenic land use trends (NWALT), 1974–2012: U.S. Geological Survey Data Series 948, 33 p. plus appendixes 3–6 as separate files, http://dx.doi.org/10.3133/ds948.

[10]Age classifications for groundwater in each well are based principally on activities of tritium (Plummer et al., 1993) and carbon-14 (Clark and Fritz, 1997). The age classifications are presented as categories: modern, modern or mi[illegible]

Plummer, L. N., Michel, R. L., Thurman, E. M., & Glynn, P. D. (1993). Environmental tracers for age-dating young groundwater, in Alley, W. M., eds. Regional Groundwater Quality (pp. 255-294). New York: Van Nostrand Reinhold.

Clark, I. D., & Fritz, P. (1997). Environmental Isotopes in Hydrogeology. Boca Raton & New York: Lewis Publishers.

[11]Septic tank density was determined from the 1990 Census of Population and Housing (U.S. Department of Commerce, 1992), and expressed as tanks/km2.

U.S. Department of Commerce. (1992), 1990 Census of population and housing, summary tape file 3A: U. S. Census Gureau, CD-ROM, http://www.census.gov/mp/www/cat/decennial_census_1990/1990_census_of_population_and_housing_summary_tape_file_3a.html.

[12]Aridity index is calculated as the average annual precipitation (PRISM Climate Group, 2012) divided by the average annual evapotranspiration (Flint and Flint, 2007), and values can range from 0.05 (hyper-arid) to greater than 1.0[illegible]

PRISM Climate Group, (2012). United States average annual precipitation, maximum and minimum temperature, 1971-2000: Oregon State University, PRISM website, accessed November 14, 2018 at http://prism.oregonstate.edu/
