# Supplementary material for: Status and trends of orthophosphate concentrations in groundwater used for public supply in California
Source: Environ Monit Assess. 2020 Jul 29;192(8):550. doi: 10.1007/s10661-020-08504-x (PMC7391407; doi:10.1007/s10661-020-08504-x)
Supplement: Supplementary file 3 — (PDF 473 kb) [file 10661_2020_8504_MOESM3_ESM.pdf]

Status and trends of orthophosphate concentrations in groundwater used for public supply in California *Environmental Monitoring and Assessment, Robert Kent, Tyler D. Johnson, and Michael R. Rosen, U.S. Geological Survey California Water Science Center-rhkent@usgs.gov*

Online resource (suppplementary table) 3. Selected attributes of GAMA-PBP (https://ca.water.usgs.gov/gama/) trend wells evaluated for step trends in orthophosphate concentration-page 1.

GAMA-PBP ID	USGS Station ID[1]	GAMA-PBP project study unit	GAMA-PBP study area[3]	Hydrogeologic Zone
COS-08	381700121190001	Northern San Joaquin Basin	Cosumnes subbasin	Central Valley
ESJ-01	374900121160001	Northern San Joaquin Basin	Eastern San Joaquin subbasin	Central Valley
ESJ-06	380606121164501	Northern San Joaquin Basin	Eastern San Joaquin subbasin	Central Valley
NSJ-QPC-04	381956121053401	Northern San Joaquin Basin	Northern San Joaquin Valley QPc area	Central Valley
TRCY-03	374100121260001	Northern San Joaquin Basin	Tracy subbasin	Central Valley
NAM-02	383703121280701	Southern Sacramento Valley	North American subbasin	Central Valley
NAM-05	384224121261401	Southern Sacramento Valley	North American subbasin	Central Valley
SAM-02	382501121222201	Southern Sacramento Valley	South American subbasin	Central Valley
SAM-07	383514121184001	Southern Sacramento Valley	South American subbasin	Central Valley
SAM-10	383046121270201	Southern Sacramento Valley	South American subbasin	Central Valley
SOL-01	380935121421601	Southern Sacramento Valley	Solano subbasin	Central Valley
SOL-03	382100121560001	Southern Sacramento Valley	Solano subbasin	Central Valley
SOL-06	381426121304001	Southern Sacramento Valley	Solano subbasin	Central Valley
SOL-08	382400121560001	Southern Sacramento Valley	Solano subbasin	Central Valley
SSV-QPC-06	384100121210001	Southern Sacramento Valley	Southern Sacramento Valley QPc area	Central Valley
SSV-QPC-07	384413121163801	Southern Sacramento Valley	Southern Sacramento Valley QPc area	Central Valley
SSV-QPC-09	383300121120001	Southern Sacramento Valley	Southern Sacramento Valley QPc area	Central Valley
SUI-03	381200121520001	Southern Sacramento Valley	Suisun subbasin	Central Valley
YOL-01	384113121455501	Southern Sacramento Valley	Yolo subbasin	Central Valley
YOL-03	383155121452001	Southern Sacramento Valley	Yolo subbasin	Central Valley
YOL-04	383121121575301	Southern Sacramento Valley	Yolo subbasin	Central Valley
YOL-06	384000121520001	Southern Sacramento Valley	Yolo subbasin	Central Valley
YOL-14	383308121322801	Southern Sacramento Valley	Yolo subbasin	Central Valley
KING-04	363600120020001	Southeast San Joaquin Valley	Kings subbasin	Central Valley
KING-11	363200119220001	Southeast San Joaquin Valley	Kings subbasin	Central Valley
KING-13	363500119280001	Southeast San Joaquin Valley	Kings subbasin	Central Valley
KING-15	364355119484601	Southeast San Joaquin Valley	Kings subbasin	Central Valley
KING-17	364156119475201	Southeast San Joaquin Valley	Kings subbasin	Central Valley
KING-20	364359119293601	Southeast San Joaquin Valley	Kings subbasin	Central Valley
KING-24	364418119415101	Southeast San Joaquin Valley	Kings subbasin	Central Valley
KING-30	363900119260001	Southeast San Joaquin Valley	Kings subbasin	Central Valley

Online resource (suppplementary table) 3. Selected attributes of GAMA-PBP (https://ca.water.usgs.gov/gama/) trend wells evaluated for step trends in orthophosphate concentration-page 2.

GAMA-PBP ID	Initial Sample Date	Initital Sample Orthophos-phate Concentra-tion (mg/L as P)	Orthophos-phate reporting level for initial sample (mg/L as P)	Triennial Trend Sample Date	Triennial Sample Orthophos-phate Concentra-tion (mg/L as P)	Orthophos-phate reporting level for triennial sample (mg/L as P)	Decadal Trend Sample Date	Decadal Sample Orthophos-phate Concentra-tion (mg/L as P)	Orthophos-phate reporting level for decadal sample (mg/L as P)	Performed Evaluation 1 (comparison between initial and triennial results)	Performed Evaluation 2 (comparison between initial and decadal results)	Performed Evaluation 3 (comparison between triennial and decadal results)
COS-08	1/3/2005[4]	na	na	4/3/2008	0.162	0.006	1/13/2014	0.159	0.004	no	no	yes
ESJ-01	1/24/2005[4]	na	na	4/2/2008	0.034	0.006	2/10/2014	0.039	0.004	no	no	yes
ESJ-06	1/10/2005[4]	na	na	4/2/2008	0.039	0.006	1/14/2014	0.040	0.004	no	no	yes
NSJ-QPC-04	1/24/2005[4]	na	na	4/1/2008	0.286	0.006	1/16/2014	0.293	0.004	no	no	yes
TRCY-03	1/6/2005	0.016	0.006	3/31/2008	0.021	0.006	2/11/2014	0.022	0.004	yes	yes	yes
NAM-02	3/29/2005	0.032	0.006	none	na	na	5/15/2014	0.141	0.004	no	yes[5]	no
NAM-05	4/7/2005	0.025	0.006	none	na	na	3/12/2014	0.056	0.004	no	yes[5]	no
SAM-02	3/15/2005	0.064	0.006	none	na	na	3/31/2014	0.090	0.004	no	yes[5]	no
SAM-07	4/5/2005	0.025	0.006	none	na	na	3/12/2014	0.030	0.004	no	yes[5]	no
SAM-10	4/21/2005[4]	na	na	4/8/2008	0.040	0.006	3/11/2014	0.043	0.004	no	no	yes
SOL-01	3/16/2005	0.123	0.006	none	na	na	3/31/2014	0.130	0.004	no	yes[5]	no
SOL-03	3/23/2005	0.023	0.006	none	na	na	4/15/2014	0.039	0.004	no	yes[5]	no
SOL-06	3/30/2005	0.225	0.012	none	na	na	4/22/2014	0.250	0.004	no	yes[5]	no
SOL-08	5/10/2005[4]	na	na	4/8/2008	0.025	0.006	4/2/2014	0.031	0.004	no	no	yes
SSV-QPC-06	3/22/2005	0.097	0.006	none	na	na	3/11/2014	0.087	0.004	no	yes	no
SSV-QPC-07	4/4/2005	0.084	0.006	4/10/2008	0.101	0.006	3/10/2014	0.103	0.004	yes	yes	yes
SSV-QPC-09	4/7/2005	0.118	0.006	none	na	na	4/14/2014	0.132	0.004	no	yes[5]	no
SUI-03	5/12/2005[4]	na	na	4/9/2008	0.041	0.006	4/3/2014	0.048	0.004	no	no	yes
YOL-01	4/11/2005[4]	na	na	4/7/2008	0.040	0.006	3/13/2014	0.040	0.004	no	no	yes
YOL-03	4/19/2005	0.052	0.006	none	na	na	4/2/2014	0.047	0.004	no	yes	no
YOL-04	4/26/2005	0.045	0.006	none	na	na	4/1/2014	0.060	0.004	no	yes	no
YOL-06	4/27/2005	0.169	0.006	none	na	na	4/1/2014	0.168	0.004	no	yes[5]	no
YOL-14	5/25/2005	0.027	0.006	4/9/2008	0.121	0.006	none	na	na	yes	no	no
KING-04	10/17/2005	0.015	0.006	none	na	na	10/19/2015	0.047	0.004	no	yes	no
KING-11	10/20/2005	0.014	0.006	11/5/2008	0.026	0.008	none	na	na	yes	no	no
KING-13	10/20/2005	0.020	0.006	11/5/2008	0.035	0.008	12/2/2015	0.038	0.004	yes	yes	yes
KING-15	10/25/2005	0.035	0.006	none	na	na	12/2/2015	0.067	0.004	no	yes	no
KING-17	10/26/2005	0.014	0.006	11/4/2008	0.030	0.008	10/21/2015	0.031	0.004	yes	yes	yes
KING-20	10/27/2005	0.078	0.006	none	na	na	10/20/2015	0.078	0.004	no	yes	no
KING-24	11/5/2005[4]	na	na	11/3/2008	0.041	0.008	10/21/2015	0.041	0.004	no	no	yes
KING-30	11/3/2005	0.024	0.006	none	na	na	10/20/2015	0.035	0.004	no	yes	no

Online resource (suppplementary table) 3. Selected attributes of GAMA-PBP (https://ca.water.usgs.gov/gama/) trend wells evaluated for step trends in orthophosphate concentration-page 3.

GAMA-PBP ID	Elevation of LSD (meters above NAVD 88)[6]	Well depth (meters below LSD)	Agricultural land use in 1974[9] (percent)	Natural land use in 1974[9] (percent)	Urban land use in 1974[9] (percent)	Agricultural land use in 1982[9] (percent)	Natural land use in 1982[9] (percent)	Urban land use in 1982[9] (percent)	Agricultural land use in 1992[9] (percent)	Natural land use in 1992[9] (percent)	Urban land use in 1992[9] (percent)
COS-08	14	189	68.6%	7.3%	24.1%	68.2%	0.5%	31.4%	52.3%	0.5%	47.3%
ESJ-01	9	89	30.6%	33.3%	36.1%	24.7%	33.3%	42.0%	20.5%	33.3%	46.1%
ESJ-06	13	132	66.4%	0.9%	32.7%	55.0%	0.5%	44.5%	52.3%	0.0%	47.7%
NSJ-QPC-04	85	171	0.0%	100.0%	0.0%	0.0%	100.0%	0.0%	0.0%	100.0%	0.0%
TRCY-03	68	295	11.0%	49.8%	39.3%	17.8%	35.2%	47.0%	9.1%	40.2%	50.7%
NAM-02	8	123	0.0%	0.0%	100.0%	0.0%	0.0%	100.0%	0.0%	0.0%	100.0%
NAM-05	22	171	8.3%	1.8%	89.9%	7.8%	0.9%	91.2%	0.5%	1.8%	97.7%
SAM-02	15	168	3.2%	0.0%	96.8%	0.5%	0.0%	99.5%	0.0%	0.0%	100.0%
SAM-07	20	101	0.0%	0.0%	100.0%	0.0%	0.0%	100.0%	0.0%	0.0%	100.0%
SAM-10	10	91	0.0%	0.0%	100.0%	0.0%	0.0%	100.0%	0.0%	0.0%	100.0%
SOL-01	35	262	62.3%	0.5%	37.3%	52.7%	0.5%	46.8%	50.9%	0.9%	48.2%
SOL-03	35	308	70.4%	0.0%	29.6%	62.9%	0.0%	37.1%	0.0%	0.0%	100.0%
SOL-06	2	80	58.9%	10.0%	31.1%	58.9%	10.0%	31.1%	58.9%	9.6%	31.5%
SOL-08	35	584	19.2%	17.4%	63.5%	9.1%	17.4%	73.5%	5.9%	17.4%	76.7%
SSV-QPC-06	40	177	14.6%	0.0%	85.4%	1.4%	0.0%	98.6%	0.0%	0.0%	100.0%
SSV-QPC-07	52	99	8.4%	0.0%	91.6%	0.9%	0.0%	99.1%	0.0%	0.0%	100.0%
SSV-QPC-09	5	97	0.0%	80.3%	19.7%	0.0%	80.3%	19.7%	0.0%	80.3%	19.7%
SUI-03	29	74	0.0%	91.4%	8.6%	0.0%	91.4%	8.6%	0.0%	91.4%	8.6%
YOL-01	19	154	0.0%	0.5%	99.5%	0.0%	0.5%	99.5%	0.0%	0.5%	99.5%
YOL-03	18	476	31.1%	4.1%	64.8%	32.0%	4.1%	63.9%	31.5%	4.1%	64.4%
YOL-04	34	89	49.5%	8.6%	41.8%	42.7%	9.1%	48.2%	39.1%	9.5%	51.4%
YOL-06	45	130	38.6%	16.4%	45.0%	38.6%	15.9%	45.5%	33.6%	20.5%	45.9%
YOL-14	4	443	31.3%	10.1%	58.5%	21.7%	10.1%	68.2%	12.4%	18.0%	69.6%
KING-04	66	164	72.6%	25.1%	2.3%	84.5%	12.8%	2.7%	78.5%	15.5%	5.9%
KING-11	112	177	7.3%	0.0%	92.7%	6.8%	0.0%	93.2%	6.8%	0.0%	93.2%
KING-13	103	138	34.9%	34.9%	30.2%	34.9%	34.9%	30.2%	33.5%	34.9%	31.6%
KING-15	93	144	0.0%	0.0%	100.0%	0.0%	0.0%	100.0%	0.0%	0.0%	100.0%
KING-17	92	213	21.0%	0.0%	79.0%	17.8%	0.0%	82.2%	17.4%	0.0%	82.6%
KING-20	129	41	72.4%	14.3%	13.4%	72.8%	13.4%	13.8%	72.4%	13.4%	14.3%
KING-24	106	77	5.0%	0.0%	95.0%	1.4%	0.0%	98.6%	0.0%	0.0%	100.0%
KING-30	126	161	81.4%	15.0%	3.6%	87.7%	8.2%	4.1%	85.0%	10.9%	4.1%

Online resource (suppplementary table) 3. Selected attributes of GAMA-PBP (https://ca.water.usgs.gov/gama/) trend wells evaluated for step trends in orthophosphate concentration-page 4.

GAMA-PBP ID	Agricultural land use in 2002[9] (percent)	Natural land use in 2002[9] (percent)	Urban land use in 2002[9] (percent)	Agricultural land use in 2012[9] (percent)	Natural land use in 2012[9] (percent)	Urban land use in 2012[9] (percent)	Age Classification[8]	Septic Tanks[10]	Aridity[11]
COS-08	52.3%	0.5%	47.3%	52.3%	0.5%	47.3%	PremodernOrMixed	20.25	0.353
ESJ-01	41.6%	11.9%	46.6%	40.2%	9.6%	50.2%	ModernOrMixed	14.25	0.261
ESJ-06	51.8%	0.0%	48.2%	51.8%	0.0%	48.2%	ModernOrMixed	4.11	0.352
NSJ-QPC-04	0.0%	99.5%	0.5%	0.0%	99.5%	0.5%	ModernOrMixed	1.75	0.404
TRCY-03	8.7%	33.8%	57.5%	7.3%	25.6%	67.1%	Premodern	0.56	0.192
NAM-02	0.0%	0.0%	100.0%	0.0%	0.0%	100.0%	ModernOrMixed	3.09	0.396
NAM-05	0.0%	0.0%	100.0%	0.0%	0.0%	100.0%	PremodernOrMixed	69.88	0.437
SAM-02	0.0%	0.0%	100.0%	0.0%	0.0%	100.0%	ModernOrMixed	1.38	0.372
SAM-07	0.0%	0.0%	100.0%	0.0%	0.0%	100.0%	ModernOrMixed	2.64	0.398
SAM-10	0.0%	0.0%	100.0%	0.0%	0.0%	100.0%	ModernOrMixed	0.95	0.370
SOL-01	50.9%	0.9%	48.2%	50.9%	0.9%	48.2%	PremodernOrMixed	0.36	0.346
SOL-03	0.0%	0.0%	100.0%	0.0%	0.0%	100.0%	PremodernOrMixed	0.19	0.473
SOL-06	58.9%	9.6%	31.5%	58.9%	9.1%	32.0%	ModernOrMixed	4.99	0.346
SOL-08	13.2%	16.4%	70.3%	11.0%	16.4%	72.6%	PremodernOrMixed	6.20	0.481
SSV-QPC-06	0.0%	0.0%	100.0%	0.0%	0.0%	100.0%	Modern	0.00	0.453
SSV-QPC-07	0.0%	0.0%	100.0%	0.0%	0.0%	100.0%	Mixed	3.19	0.469
SSV-QPC-09	0.0%	80.3%	19.7%	0.0%	60.6%	39.4%	PremodernOrMixed	0.38	0.433
SUI-03	0.0%	91.4%	8.6%	0.0%	91.4%	8.6%	PremodernOrMixed	0.37	0.387
YOL-01	0.0%	0.5%	99.5%	0.0%	0.0%	100.0%	ModernOrMixed	11.46	0.404
YOL-03	34.7%	3.7%	61.6%	26.5%	0.9%	72.6%	Premodern	0.54	0.381
YOL-04	39.5%	9.1%	51.4%	39.5%	6.8%	53.6%	Modern	3.54	0.459
YOL-06	33.6%	20.5%	45.9%	26.4%	18.2%	55.5%	ModernOrMixed	4.47	0.414
YOL-14	12.0%	16.1%	71.9%	9.7%	16.1%	74.2%	Premodern	40.34	0.368
KING-04	90.9%	1.4%	7.8%	90.9%	1.4%	7.8%	Mixed	1.25	0.159
KING-11	6.4%	0.0%	93.6%	0.0%	0.0%	100.0%	PremodernOrMixed	4.33	0.211
KING-13	53.8%	18.4%	27.8%	53.8%	8.5%	37.7%	PremodernOrMixed	3.00	0.210
KING-15	0.0%	0.0%	100.0%	0.0%	0.0%	100.0%	ModernOrMixed	15.81	0.208
KING-17	17.4%	0.0%	82.6%	16.4%	0.0%	83.6%	Premodern	15.63	0.206
KING-20	72.8%	12.9%	14.3%	72.8%	12.9%	14.3%	Modern	7.31	0.219
KING-24	0.0%	0.0%	100.0%	0.0%	0.0%	100.0%	ModernOrMixed	37.30	0.209
KING-30	86.4%	8.6%	5.0%	86.4%	8.6%	5.0%	PremodernOrMixed	4.75	0.215

Online resource (suppplementary table) 3. Selected attributes of GAMA-PBP (https://ca.water.usgs.gov/gama/) trend wells evaluated for step trends in orthophosphate concentration-page 5.

GAMA-PBP ID	USGS Station ID[1]	GAMA-PBP project study unit	GAMA-PBP study area[3]	Hydrogeologic Zone
KWH-06	361224119060001	Southeast San Joaquin Valley	Kaweah subbasin	Central Valley
KWH-10	360850119032201	Southeast San Joaquin Valley	Kaweah subbasin	Central Valley
KWH-11	360945119200001	Southeast San Joaquin Valley	Kaweah subbasin	Central Valley
KWH-12	361228119202101	Southeast San Joaquin Valley	Kaweah subbasin	Central Valley
TLR-03	361801119380001	Southeast San Joaquin Valley	Tulare Lake subbasin	Central Valley
TLR-04	362128119390001	Southeast San Joaquin Valley	Tulare Lake subbasin	Central Valley
TULE-01	355259119160701	Southeast San Joaquin Valley	Tule subbasin	Central Valley
TULE-03	360631119190001	Southeast San Joaquin Valley	Tule subbasin	Central Valley
TULE-07	360331119180001	Southeast San Joaquin Valley	Tule subbasin	Central Valley
TULE-10	360118119010001	Southeast San Joaquin Valley	Tule subbasin	Central Valley
KERN-02	351200118490001	Kern County Subbasin	Kern County subbasin	Central Valley
KERN-20	351300119150001	Kern County Subbasin	Kern County subbasin	Central Valley
KERN-21	352800119260001	Kern County Subbasin	Kern County subbasin	Central Valley
KERN-29	354400119100001	Kern County Subbasin	Kern County subbasin	Central Valley
KERN-34	351800119180001	Kern County Subbasin	Kern County subbasin	Central Valley
KERN-42	351800119040001	Kern County Subbasin	Kern County subbasin	Central Valley
KERN-43	350300118580001	Kern County Subbasin	Kern County subbasin	Central Valley
KERN-45	352800119170001	Kern County Subbasin	Kern County subbasin	Central Valley
KERN-47	352600119120001	Kern County Subbasin	Kern County subbasin	Central Valley
CE-QPC-01	374526120475501	Central Eastside San Joaquin Basin	Central-Eastside San Joaquin Valley QPc area	Central Valley
CE-QPC-02	373828120444001	Central Eastside San Joaquin Basin	Central-Eastside San Joaquin Valley QPc area	Central Valley
MER-10	371829120300801	Central Eastside San Joaquin Basin	Merced subbasin	Central Valley
MER-11	371300120150001	Central Eastside San Joaquin Basin	Merced subbasin	Central Valley
MER-12	371100120370001	Central Eastside San Joaquin Basin	Merced subbasin	Central Valley
MER-14	372100120340001	Central Eastside San Joaquin Basin	Merced subbasin	Central Valley
MOD-02	374109121000101	Central Eastside San Joaquin Basin	Modesto subbasin	Central Valley
TRLK-02	372900120490001	Central Eastside San Joaquin Basin	Turlock subbasin	Central Valley
TRLK-03	373500120510001	Central Eastside San Joaquin Basin	Turlock subbasin	Central Valley
TRLK-05	373000120500001	Central Eastside San Joaquin Basin	Turlock subbasin	Central Valley
TRLK-11	373100120470001	Central Eastside San Joaquin Basin	Turlock subbasin	Central Valley
ESAC-01	390058121260001	Middle Sacramento Valley	Eastern Sacramento Valley subbasins	Central Valley

Online resource (suppplementary table) 3. Selected attributes of GAMA-PBP (https://ca.water.usgs.gov/gama/) trend wells evaluated for step trends in orthophosphate concentration-page 6.

GAMA-PBP ID	Initial Sample Date	Initital Sample Orthophos-phate Concentra-tion (mg/L as P)	Orthophos-phate reporting level for initial sample (mg/L as P)	Triennial Trend Sample Date	Triennial Sample Orthophos-phate Concentra-tion (mg/L as P)	Orthophos-phate reporting level for triennial sample (mg/L as P)	Decadal Trend Sample Date	Decadal Sample Orthophos-phate Concentra-tion (mg/L as P)	Orthophos-phate reporting level for decadal sample (mg/L as P)	Performed Evaluation 1 (comparison between initial and triennial results)	Performed Evaluation 2 (comparison between initial and decadal results)	Performed Evaluation 3 (comparison between triennial and decadal results)
KWH-06	11/15/2005	0.007	0.006	none	na	na	10/28/2015	0.017	0.004	no	yes	no
KWH-10	11/17/2005[4]	na	na	11/5/2008	0.021	0.008	10/26/2015	0.022	0.004	no	no	yes
KWH-11	11/17/2005	0.042	0.006	none	na	na	10/28/2015	0.060	0.004	no	yes	no
KWH-12	11/28/2005	not detected	0.006	11/6/2008	0.007	0.008	10/29/2015	not detected	0.004	yes	yes	yes
TLR-03	11/29/2005[4]	na	na	11/4/2008	0.048	0.008	11/30/2015	0.055	0.004	no	no	yes
TLR-04	12/1/2005	0.021	0.006	none	na	na	10/27/2015	0.030	0.004	no	yes	no
TULE-01	11/29/2005	0.006	0.006	none	na	na	12/1/2015	0.013	0.004	no	yes	no
TULE-03	11/30/2005	0.020	0.006	none	na	na	12/1/2015	0.030	0.004	no	yes	no
TULE-07	12/6/2005	0.005	0.006	none	na	na	11/30/2015	0.012	0.004	no	yes	no
TULE-10	12/7/2005[4]	na	na	11/3/2008	0.020	0.008	10/26/2015	0.016	0.004	no	no	yes
KERN-02	1/10/2006[4]	na	na	2/11/2010	0.034	0.008	1/13/2016	0.022	0.004	no	no	yes
KERN-20	2/13/2006[4]	na	na	2/10/2010	0.053	0.008	12/3/2015	0.073	0.004	no	no	yes
KERN-21	2/13/2006[4]	na	na	3/16/2010	0.055	0.008	1/11/2016	0.008	0.004	no	no	yes
KERN-29	2/28/2006[4]	na	na	2/10/2010	0.031	0.008	12/3/2015	0.022	0.004	no	no	yes
KERN-34	1/10/2006	0.006	0.006	none	na	na	1/13/2016	0.007	0.004	no	yes	no
KERN-42	2/1/2006	0.012	0.006	none	na	na	1/12/2016	0.017	0.004	no	yes	no
KERN-43	2/2/2006	0.004	0.006	none	na	na	4/19/2016	0.011	0.004	no	yes	no
KERN-45	2/8/2006	0.009	0.006	none	na	na	1/11/2016	0.008	0.004	no	yes	no
KERN-47	3/2/2006	not detected	0.006	none	na	na	1/14/2016	0.032	0.004	no	yes	no
CE-QPC-01	3/20/2006	0.022	0.006	none	na	na	2/8/2016	0.032	0.004	no	yes	no
CE-QPC-02	3/22/2006[4]	na	na	1/26/2010	0.079	0.008	3/1/2016	0.057	0.004	no	no	yes
MER-10	4/11/2006	0.023	0.006	none	na	na	2/11/2016	0.036	0.004	no	yes	no
MER-11	4/12/2006	0.030	0.006	1/28/2010	0.048	0.008	3/2/2016	0.037	0.004	yes	yes	yes
MER-12	4/13/2006	0.029	0.006	none	na	na	3/3/2016	0.050	0.004	no	yes	no
MER-14	4/17/2006[4]	na	na	1/27/2010	0.051	0.008	3/1/2016	0.029	0.004	no	no	yes
MOD-02	3/14/2006	0.021	0.006	none	na	na	2/10/2016	0.037	0.004	no	yes	no
TRLK-02	3/16/2006	0.011	0.006	none	na	na	2/9/2016	0.025	0.004	no	yes	no
TRLK-03	3/21/2006	0.046	0.006	1/28/2010	0.073	0.008	3/3/2016	0.064	0.004	yes	yes	yes
TRLK-05	3/22/2006	0.009	0.006	1/25/2010	0.034	0.008	2/8/2016	0.026	0.004	yes	yes	yes
TRLK-11	3/28/2006	0.011	0.006	none	na	na	2/29/2016	0.025	0.004	no	yes	no
ESAC-01	6/29/2006[4]	na	na	8/11/2010	0.103	0.008	10/4/2016	0.106	0.004	no	no	yes

Online resource (suppplementary table) 3. Selected attributes of GAMA-PBP (https://ca.water.usgs.gov/gama/) trend wells evaluated for step trends in orthophosphate concentration-page 7.

GAMA-PBP ID	Elevation of LSD (meters above NAVD 88)[6]	Well depth (meters below LSD)	Agricultural land use in 1974[9] (percent)	Natural land use in 1974[9] (percent)	Urban land use in 1974[9] (percent)	Agricultural land use in 1982[9] (percent)	Natural land use in 1982[9] (percent)	Urban land use in 1982[9] (percent)	Agricultural land use in 1992[9] (percent)	Natural land use in 1992[9] (percent)	Urban land use in 1992[9] (percent)
KWH-06	122	190	18.7%	2.8%	78.5%	16.4%	0.9%	82.7%	15.0%	0.9%	84.1%
KWH-10	135	106	18.3%	1.4%	80.4%	18.7%	1.4%	79.9%	18.7%	1.4%	79.9%
KWH-11	89	230	50.5%	4.6%	45.0%	51.4%	3.2%	45.4%	50.5%	3.2%	46.3%
KWH-12	95	133	0.0%	0.0%	100.0%	0.0%	0.0%	100.0%	0.0%	0.0%	100.0%
TLR-03	81	466	64.8%	2.7%	32.4%	67.1%	2.7%	30.1%	66.7%	2.7%	30.6%
TLR-04	83	433	69.5%	1.4%	29.1%	64.1%	1.4%	34.5%	23.2%	0.0%	76.8%
TULE-01	94	262	11.4%	1.4%	87.3%	11.8%	0.9%	87.3%	11.4%	0.9%	87.7%
TULE-03	87	92	60.9%	29.1%	10.0%	72.7%	16.4%	10.9%	60.9%	26.4%	12.7%
TULE-07	91	197	23.6%	0.0%	76.4%	24.1%	0.0%	75.9%	24.1%	0.0%	75.9%
TULE-10	132	317	94.4%	0.9%	4.6%	96.3%	0.9%	2.8%	95.8%	1.4%	2.8%
KERN-02	150	230	49.5%	1.4%	49.1%	50.0%	0.9%	49.1%	49.5%	0.9%	49.5%
KERN-20	97	328	6.9%	82.9%	10.1%	6.9%	82.9%	10.1%	6.9%	82.9%	10.1%
KERN-21	88	149	94.5%	5.0%	0.5%	98.6%	0.9%	0.5%	98.6%	0.9%	0.5%
KERN-29	132	164	52.1%	47.9%	0.0%	52.1%	47.9%	0.0%	52.1%	47.9%	0.0%
KERN-34	101	251	0.9%	99.1%	0.0%	0.9%	99.1%	0.0%	0.9%	99.1%	0.0%
KERN-42	118	236	24.5%	5.9%	69.5%	25.9%	3.6%	70.5%	0.9%	0.0%	99.1%
KERN-43	175	289	72.1%	2.3%	25.6%	69.4%	2.3%	28.3%	68.9%	2.3%	28.8%
KERN-45	108	197	93.6%	3.2%	3.2%	96.4%	0.9%	2.7%	96.4%	0.9%	2.7%
KERN-47	113	266	84.0%	5.9%	10.0%	84.5%	5.5%	10.0%	83.6%	5.5%	11.0%
CE-QPC-01	75	92	49.3%	28.2%	22.5%	54.5%	24.4%	21.1%	51.6%	23.9%	24.4%
CE-QPC-02	57	98	41.4%	0.9%	57.7%	41.4%	0.9%	57.7%	41.4%	0.0%	58.6%
MER-10	55	96	12.3%	0.0%	87.7%	7.3%	0.0%	92.7%	5.5%	0.0%	94.5%
MER-11	84	207	55.0%	1.8%	43.2%	41.8%	1.4%	56.8%	40.9%	0.0%	59.1%
MER-12	36	69	56.2%	43.3%	0.5%	58.1%	41.5%	0.5%	62.2%	37.3%	0.5%
MER-14	56	241	13.2%	3.7%	83.1%	12.3%	3.2%	84.5%	13.2%	2.3%	84.5%
MOD-02	30	130	0.0%	0.0%	100.0%	0.0%	0.0%	100.0%	0.0%	0.0%	100.0%
TRLK-02	35	89	77.6%	0.0%	22.4%	77.6%	0.0%	22.4%	77.6%	0.0%	22.4%
TRLK-03	42	163	80.3%	6.9%	12.8%	81.2%	6.0%	12.8%	81.2%	6.0%	12.8%
TRLK-05	35	155	0.0%	0.0%	100.0%	0.0%	0.0%	100.0%	0.0%	0.0%	100.0%
TRLK-11	7	135	47.0%	0.0%	53.0%	47.0%	0.0%	53.0%	47.0%	0.0%	53.0%
ESAC-01	25	91	60.7%	2.7%	36.5%	56.6%	2.7%	40.6%	54.3%	2.7%	42.9%

Online resource (suppplementary table) 3. Selected attributes of GAMA-PBP (https://ca.water.usgs.gov/gama/) trend wells evaluated for step trends in orthophosphate concentration-page 8.

GAMA-PBP ID	Agricultural land use in 2002[9] (percent)	Natural land use in 2002[9] (percent)	Urban land use in 2002[9] (percent)	Agricultural land use in 2012[9] (percent)	Natural land use in 2012[9] (percent)	Urban land use in 2012[9] (percent)	Age Classification[8]	Septic Tanks[10]	Aridity[11]
KWH-06	13.6%	0.5%	86.0%	13.6%	0.0%	86.4%	ModernOrMixed	8.72	0.199
KWH-10	20.1%	0.0%	79.9%	20.1%	0.0%	79.9%	ModernOrMixed	28.06	0.201
KWH-11	49.5%	3.2%	47.2%	46.8%	0.0%	53.2%	ModernOrMixed	9.53	0.164
KWH-12	0.0%	0.0%	100.0%	0.0%	0.0%	100.0%	Mixed	1.30	0.170
TLR-03	66.2%	2.7%	31.1%	66.2%	2.7%	31.1%	ModernOrMixed	13.29	0.152
TLR-04	9.5%	0.0%	90.5%	6.8%	0.0%	93.2%	Premodern	4.21	0.153
TULE-01	11.4%	0.9%	87.7%	5.9%	0.0%	94.1%	Mixed	3.06	0.137
TULE-03	63.6%	23.2%	13.2%	63.6%	23.2%	13.2%	ModernOrMixed	1.97	0.155
TULE-07	24.1%	0.0%	75.9%	24.1%	0.0%	75.9%	ModernOrMixed	0.15	0.152
TULE-10	95.4%	1.4%	3.2%	95.4%	1.4%	3.2%	ModernOrMixed	5.38	0.188
KERN-02	49.5%	0.9%	49.5%	49.5%	0.0%	50.5%	ModernOrMixed	5.76	0.119
KERN-20	6.5%	82.9%	10.6%	6.5%	82.9%	10.6%	ModernOrMixed	0.32	0.112
KERN-21	99.5%	0.0%	0.5%	99.5%	0.0%	0.5%	ModernOrMixed	1.61	0.116
KERN-29	52.1%	47.9%	0.0%	52.1%	47.9%	0.0%	ModernOrMixed	0.60	0.141
KERN-34	2.8%	97.2%	0.0%	24.1%	75.9%	0.0%	Modern	0.74	0.114
KERN-42	0.0%	0.0%	100.0%	0.0%	0.0%	100.0%	Premodern	0.00	0.111
KERN-43	68.5%	1.8%	29.7%	68.5%	0.0%	31.5%	PremodernOrMixed	0.39	0.121
KERN-45	95.9%	0.9%	3.2%	95.9%	0.9%	3.2%	Mixed	11.46	0.120
KERN-47	84.5%	4.6%	11.0%	84.5%	4.1%	11.4%	ModernOrMixed	2.31	0.118
CE-QPC-01	51.6%	23.9%	24.4%	51.6%	23.9%	24.4%	Modern	15.97	0.291
CE-QPC-02	33.6%	0.0%	66.4%	18.2%	0.0%	81.8%	ModernOrMixed	7.52	0.269
MER-10	0.0%	0.0%	100.0%	0.0%	0.0%	100.0%	Modern	5.04	0.238
MER-11	40.5%	0.0%	59.5%	38.6%	0.5%	60.9%	Modern	9.79	0.249
MER-12	65.0%	34.6%	0.5%	65.0%	34.1%	0.9%	Modern	0.62	0.229
MER-14	13.2%	2.3%	84.5%	12.3%	2.7%	84.9%	ModernOrMixed	2.96	0.227
MOD-02	0.0%	0.0%	100.0%	0.0%	0.0%	100.0%	Modern	7.04	0.257
TRLK-02	77.6%	0.0%	22.4%	77.6%	0.0%	22.4%	Mixed	39.46	0.245
TRLK-03	81.2%	6.0%	12.8%	58.7%	2.8%	38.5%	Premodern	15.92	0.261
TRLK-05	0.0%	0.0%	100.0%	0.0%	0.0%	100.0%	Mixed	20.87	0.246
TRLK-11	47.0%	0.0%	53.0%	13.5%	0.0%	86.5%	Mixed	13.20	0.252
ESAC-01	53.0%	2.7%	44.3%	42.0%	0.5%	57.5%	ModernOrMixed	6.48	0.423

Online resource (suppplementary table) 3. Selected attributes of GAMA-PBP (https://ca.water.usgs.gov/gama/) trend wells evaluated for step trends in orthophosphate concentration-page 9.

GAMA-PBP ID	USGS Station ID[1]	GAMA-PBP project study unit	GAMA-PBP study area[3]	Hydrogeologic Zone
ESAC-18	394300121480001	Middle Sacramento Valley	Eastern Sacramento Valley subbasins	Central Valley
ESAC-19	390800121450001	Middle Sacramento Valley	Eastern Sacramento Valley subbasins	Central Valley
ESAC-27	390900121350001	Middle Sacramento Valley	Eastern Sacramento Valley subbasins	Central Valley
ESAC-28	392200121413201	Middle Sacramento Valley	Eastern Sacramento Valley subbasins	Central Valley
ESAC-31	392000121560001	Middle Sacramento Valley	Eastern Sacramento Valley subbasins	Central Valley
ESAC-34	392910121451101	Middle Sacramento Valley	Eastern Sacramento Valley subbasins	Central Valley
WSAC-03	395500122100001	Middle Sacramento Valley	Western Sacramento Valley subbasins	Central Valley
WSAC-06	394600122210001	Middle Sacramento Valley	Western Sacramento Valley subbasins	Central Valley
WSAC-08	394435122110801	Middle Sacramento Valley	Western Sacramento Valley subbasins	Central Valley
WSAC-17	385123121470801	Middle Sacramento Valley	Western Sacramento Valley subbasins	Central Valley
WSAC-18	390900122030001	Middle Sacramento Valley	Western Sacramento Valley subbasins	Central Valley
WSAC-19	385543121552101	Middle Sacramento Valley	Western Sacramento Valley subbasins	Central Valley
WSAC-22	390000122080001	Middle Sacramento Valley	Western Sacramento Valley subbasins	Central Valley
WSAC-32	392800122010001	Middle Sacramento Valley	Western Sacramento Valley subbasins	Central Valley
NSAC-09	400900122130001	Northern Sacramento Valley	Northern Sacramento Valley subbasins	Central Valley
NSAC-12	400400122090001	Northern Sacramento Valley	Northern Sacramento Valley subbasins	Central Valley
NSAC-16	400118122054501	Northern Sacramento Valley	Northern Sacramento Valley subbasins	Central Valley
NSAC-19	400900122210001	Northern Sacramento Valley	Northern Sacramento Valley subbasins	Central Valley
RED-01	402000122130001	Northern Sacramento Valley	Redding area basin	Central Valley
RED-04	403000122180001	Northern Sacramento Valley	Redding area basin	Central Valley
RED-05	402300122170001	Northern Sacramento Valley	Redding area basin	Central Valley
RED-12	403300122140001	Northern Sacramento Valley	Redding area basin	Central Valley
RED-19	400900122084301	Northern Sacramento Valley	Redding area basin	Central Valley
MADCHOW-03	365747120034901	Madera-Chowchilla	Madera and Chowchilla subbasins	Central Valley
MADCHOW-04	365042119493001	Madera-Chowchilla	Madera and Chowchilla subbasins	Central Valley
MADCHOW-05	370300119590001	Madera-Chowchilla	Madera and Chowchilla subbasins	Central Valley
MADCHOW-10	370700120150001	Madera-Chowchilla	Madera and Chowchilla subbasins	Central Valley
MADCHOW-12	370700120290001 [2]	Madera-Chowchilla	Madera and Chowchilla subbasins	Central Valley
MADCHOW-24	370056120200601	Madera-Chowchilla	Madera and Chowchilla subbasins	Central Valley
MADCHOW-28	370100120210001	Madera-Chowchilla	Madera and Chowchilla subbasins	Central Valley
MADCHOW-29	365045120151801	Madera-Chowchilla	Madera and Chowchilla subbasins	Central Valley

Online resource (suppplementary table) 3. Selected attributes of GAMA-PBP (https://ca.water.usgs.gov/gama/) trend wells evaluated for step trends in orthophosphate concentration-page 10.

GAMA-PBP ID	Initial Sample Date	Initital Sample Orthophos-phate Concentra-tion (mg/L as P)	Orthophos-phate reporting level for initial sample (mg/L as P)	Triennial Trend Sample Date	Triennial Sample Orthophos-phate Concentra-tion (mg/L as P)	Orthophos-phate reporting level for triennial sample (mg/L as P)	Decadal Trend Sample Date	Decadal Sample Orthophos-phate Concentra-tion (mg/L as P)	Orthophos-phate reporting level for decadal sample (mg/L as P)	Performed Evaluation 1 (comparison between initial and triennial results)	Performed Evaluation 2 (comparison between initial and decadal results)	Performed Evaluation 3 (comparison between triennial and decadal results)
ESAC-18	7/20/2006	0.086	0.006	none	na	na	9/20/2016	0.083	0.004	no	yes	no
ESAC-19	7/20/2006	0.090	0.006	8/10/2010	0.096	0.008	9/22/2016	0.092	0.004	yes	yes	yes
ESAC-27	8/2/2006	0.100	0.006	none	na	na	10/4/2016	0.101	0.004	no	yes	no
ESAC-28	8/3/2006	0.194	0.006	none	na	na	10/5/2016	0.193	0.004	no	yes	no
ESAC-31	8/7/2006	0.101	0.006	none	na	na	10/5/2016	0.098	0.004	no	yes	no
ESAC-34	8/17/2006	0.108	0.006	8/12/2010	0.112	0.008	9/22/2016	0.104	0.004	yes	yes	yes
WSAC-03	7/11/2006	0.036	0.006	8/10/2010	0.043	0.008	9/19/2016	0.038	0.004	yes[5]	yes[5]	yes[5]
WSAC-06	7/12/2006	0.028	0.006	none	na	na	9/19/2016	0.028	0.004	no	yes[5]	no
WSAC-08	7/18/2006	0.031	0.006	8/12/2010	0.033	0.008	9/20/2016	0.030	0.004	yes	yes	yes
WSAC-17	8/1/2006	0.257	0.006	8/9/2010	0.226	0.008	10/6/2016	0.238	0.004	yes	yes	yes
WSAC-18	8/1/2006	0.070	0.006	none	na	na	10/3/2016	0.069	0.004	no	yes	no
WSAC-19	8/1/2006[4]	na	na	8/11/2010	0.100	0.008	10/3/2016	0.159	0.004	no	no	yes
WSAC-22	8/8/2006	0.047	0.006	none	na	na	9/21/2016	0.039	0.004	no	yes	no
WSAC-32	8/21/2006[4]	na	na	8/9/2010	0.062	0.008	9/21/2016	0.062	0.004	no	no	yes
NSAC-09	11/6/2007	0.052	0.006	1/11/2011	0.058	0.004	10/25/2017	0.051	0.004	yes	yes	yes
NSAC-12	11/27/2007	0.057	0.006	none	na	na	1/17/2018	0.063	0.004	no	yes	no
NSAC-16	12/6/2007	0.139	0.006	1/12/2011	0.148	0.004	10/26/2017	0.142	0.004	yes	yes	yes
NSAC-19	1/8/2008	0.035	0.006	none	na	na	10/25/2017	0.038	0.004	no	yes	no
RED-01	10/1/2007	0.128	0.006	1/11/2011	0.133	0.004	10/23/2017	0.128	0.004	yes	yes	yes
RED-04	10/3/2007	0.044	0.006	none	na	na	10/24/2017	0.042	0.004	no	yes	no
RED-05	10/3/2007	0.184	0.006	none	na	na	1/17/2018	0.178	0.004	no	yes	no
RED-12	11/8/2007	0.156	0.006	1/10/2011	0.154	0.004	1/16/2018	0.156	0.004	yes	yes	yes
RED-19	12/11/2007	0.056	0.006	none	na	na	10/24/2017	0.081	0.004	no	yes	no
MADCHOW-03	4/15/2008	0.058	0.006	3/15/2011	0.063	0.004	6/5/2018	0.059	0.004	yes	yes	yes
MADCHOW-04	4/16/2008	0.094	0.006	none	na	na	6/6/2018	0.087	0.004	no	yes	no
MADCHOW-05	4/16/2008	0.033	0.006	3/15/2011	0.037	0.004	6/5/2018	0.033	0.004	yes[5]	yes[5]	yes
MADCHOW-10	4/24/2008	0.038	0.006	none	na	na	6/7/2018	0.042	0.004	no	yes	no
MADCHOW-12	4/28/2008	0.017	0.006	none	na	na	6/6/2018	0.021	0.004	no	yes	no
MADCHOW-24	5/13/2008	0.024	0.006	3/16/2011	0.043	0.004	none	na	na	yes	no	no
MADCHOW-28	5/19/2008	0.053	0.006	3/16/2011	0.045	0.004	none	na	na	yes	no	no
MADCHOW-29	5/20/2008	0.041	0.006	none	na	na	6/4/2018	0.037	0.004	no	yes	no

Online resource (suppplementary table) 3. Selected attributes of GAMA-PBP (https://ca.water.usgs.gov/gama/) trend wells evaluated for step trends in orthophosphate concentration-page 11.

GAMA-PBP ID	Elevation of LSD (meters above NAVD 88)[6]	Well depth (meters below LSD)	Agricultural land use in 1974[9] (percent)	Natural land use in 1974[9] (percent)	Urban land use in 1974[9] (percent)	Agricultural land use in 1982[9] (percent)	Natural land use in 1982[9] (percent)	Urban land use in 1982[9] (percent)	Agricultural land use in 1992[9] (percent)	Natural land use in 1992[9] (percent)	Urban land use in 1992[9] (percent)
ESAC-18	72	184	23.2%	0.0%	76.8%	23.2%	0.0%	76.8%	0.0%	0.0%	100.0%
ESAC-19	15	87	84.1%	0.0%	15.9%	83.6%	0.0%	16.4%	83.6%	0.0%	16.4%
ESAC-27	21	44	0.5%	19.7%	79.8%	1.4%	18.8%	79.8%	1.4%	18.8%	79.8%
ESAC-28	30	118	4.6%	0.0%	95.4%	4.2%	0.0%	95.8%	2.3%	0.0%	97.7%
ESAC-31	20	77	99.1%	0.9%	0.0%	99.5%	0.5%	0.0%	99.5%	0.5%	0.0%
ESAC-34	33	20	96.2%	2.8%	0.9%	96.2%	2.8%	0.9%	96.2%	2.8%	0.9%
WSAC-03	90	na	5.0%	3.2%	91.8%	5.0%	3.2%	91.8%	2.3%	3.2%	94.5%
WSAC-06	159	na	0.0%	100.0%	0.0%	0.0%	100.0%	0.0%	0.0%	100.0%	0.0%
WSAC-08	81	59	1.8%	0.0%	98.2%	1.8%	0.0%	98.2%	1.8%	0.0%	98.2%
WSAC-17	11	85	79.1%	15.5%	5.5%	79.1%	15.5%	5.5%	77.7%	16.8%	5.5%
WSAC-18	28	132	3.2%	4.5%	92.3%	4.1%	4.5%	91.4%	3.6%	4.1%	92.3%
WSAC-19	12	119	100.0%	0.0%	0.0%	100.0%	0.0%	0.0%	100.0%	0.0%	0.0%
WSAC-22	116	285	61.2%	38.8%	0.0%	61.6%	38.4%	0.0%	61.6%	38.4%	0.0%
WSAC-32	29	59	95.0%	0.5%	4.6%	95.0%	0.5%	4.6%	94.5%	0.5%	5.0%
NSAC-09	104	167	0.0%	12.0%	88.0%	0.0%	12.0%	88.0%	0.0%	12.0%	88.0%
NSAC-12	82	79	49.8%	29.7%	20.5%	48.4%	29.7%	21.9%	48.4%	29.7%	21.9%
NSAC-16	73	101	39.4%	0.0%	60.6%	36.7%	0.0%	63.3%	32.6%	0.0%	67.4%
NSAC-19	142	111	23.4%	75.2%	1.4%	23.4%	75.2%	1.4%	17.4%	81.2%	1.4%
RED-01	232	137	0.0%	98.6%	1.4%	0.0%	98.6%	1.4%	0.0%	98.6%	1.4%
RED-04	161	130	5.0%	0.5%	94.5%	5.0%	0.5%	94.5%	2.7%	0.5%	96.8%
RED-05	157	161	1.4%	1.4%	97.2%	0.5%	0.0%	99.5%	0.5%	0.0%	99.5%
RED-12	150	118	20.5%	7.8%	71.7%	10.5%	8.2%	81.3%	10.5%	8.2%	81.3%
RED-19	138	98	14.0%	85.1%	0.9%	14.0%	85.1%	0.9%	14.0%	85.1%	0.9%
MADCHOW-03	90	177	0.0%	0.0%	100.0%	0.0%	0.0%	100.0%	0.0%	0.0%	100.0%
MADCHOW-04	111	157	17.3%	5.0%	77.7%	17.3%	4.5%	78.2%	1.4%	0.9%	97.7%
MADCHOW-05	117	115	57.3%	32.7%	10.0%	60.5%	29.5%	10.0%	60.0%	30.0%	10.0%
MADCHOW-10	77	272	0.0%	0.0%	100.0%	0.0%	0.0%	100.0%	0.0%	0.0%	100.0%
MADCHOW-12	47	98	84.4%	3.2%	12.4%	83.9%	3.2%	12.8%	83.9%	3.2%	12.8%
MADCHOW-24	55	96	97.7%	1.8%	0.5%	97.7%	1.8%	0.5%	97.7%	1.8%	0.5%
MADCHOW-28	56	71	98.6%	0.0%	1.4%	98.6%	0.0%	1.4%	98.6%	0.0%	1.4%
MADCHOW-29	58	112	72.4%	25.8%	1.8%	76.0%	19.8%	4.1%	71.0%	23.0%	6.0%

Online resource (suppplementary table) 3. Selected attributes of GAMA-PBP (https://ca.water.usgs.gov/gama/) trend wells evaluated for step trends in orthophosphate concentration-page 12.

GAMA-PBP ID	Agricultural land use in 2002[9] (percent)	Natural land use in 2002[9] (percent)	Urban land use in 2002[9] (percent)	Agricultural land use in 2012[9] (percent)	Natural land use in 2012[9] (percent)	Urban land use in 2012[9] (percent)	Age Classification[8]	Septic Tanks[10]	Aridity[11]
ESAC-18	0.0%	0.0%	100.0%	0.0%	0.0%	100.0%	Premodern	108.17	0.556
ESAC-19	82.7%	0.0%	17.3%	82.7%	0.0%	17.3%	Premodern	23.04	0.424
ESAC-27	1.4%	18.8%	79.8%	1.4%	18.8%	79.8%	Modern	10.01	0.438
ESAC-28	2.3%	0.0%	97.7%	2.3%	0.0%	97.7%	Modern	16.87	0.454
ESAC-31	100.0%	0.0%	0.0%	100.0%	0.0%	0.0%	Mixed	0.55	0.401
ESAC-34	95.8%	2.8%	1.4%	95.8%	2.8%	1.4%	Modern	0.26	0.490
WSAC-03	2.3%	3.2%	94.5%	0.0%	2.7%	97.3%	Modern	1.83	0.479
WSAC-06	0.0%	100.0%	0.0%	0.0%	100.0%	0.0%	Modern	0.25	0.456
WSAC-08	1.8%	0.0%	98.2%	1.8%	0.0%	98.2%	Mixed	35.50	0.452
WSAC-17	79.5%	15.0%	5.5%	79.5%	15.0%	5.5%	Mixed	0.42	0.385
WSAC-18	3.6%	4.1%	92.3%	3.6%	0.0%	96.4%	Mixed	1.08	0.342
WSAC-19	99.5%	0.0%	0.5%	99.5%	0.0%	0.5%	PremodernOrMixed	1.41	0.388
WSAC-22	63.0%	37.0%	0.0%	63.0%	37.0%	0.0%	Mixed	0.69	0.388
WSAC-32	94.1%	0.5%	5.5%	94.1%	0.0%	5.9%	ModernOrMixed	0.70	0.415
NSAC-09	0.0%	12.0%	88.0%	0.0%	12.0%	88.0%	Mixed	5.71	0.552
NSAC-12	49.3%	28.8%	21.9%	52.5%	28.3%	19.2%	Premodern	19.89	0.501
NSAC-16	31.2%	0.0%	68.8%	31.2%	0.0%	68.8%	Mixed	52.10	0.499
NSAC-19	16.5%	82.1%	1.4%	16.5%	82.1%	1.4%	Mixed	1.54	0.573
RED-01	0.0%	97.7%	2.3%	0.0%	97.2%	2.8%	Premodern	2.39	0.646
RED-04	6.8%	0.0%	93.2%	6.8%	0.0%	93.2%	Modern	23.56	0.769
RED-05	0.0%	0.0%	100.0%	0.0%	0.0%	100.0%	Premodern	7.64	0.655
RED-12	23.7%	7.3%	68.9%	23.3%	7.3%	69.4%	Mixed	17.29	0.765
RED-19	19.1%	79.5%	1.4%	19.1%	79.5%	1.4%	Modern	1.46	0.615
MADCHOW-03	0.0%	0.0%	100.0%	0.0%	0.0%	100.0%	Mixed	4.05	0.218
MADCHOW-04	1.4%	0.9%	97.7%	1.4%	0.9%	97.7%	Modern	13.78	0.214
MADCHOW-05	60.0%	30.0%	10.0%	57.7%	31.8%	10.5%	Mixed	1.73	0.227
MADCHOW-10	0.0%	0.0%	100.0%	0.0%	0.0%	100.0%	Premodern	1.87	0.230
MADCHOW-12	83.9%	3.2%	12.8%	83.9%	3.2%	12.8%	Mixed	1.06	0.224
MADCHOW-24	97.3%	1.8%	0.9%	97.3%	1.8%	0.9%	Mixed	2.79	0.198
MADCHOW-28	98.6%	0.0%	1.4%	98.6%	0.0%	1.4%	Modern	2.79	0.204
MADCHOW-29	69.1%	22.1%	8.8%	65.4%	22.1%	12.4%	Modern	0.38	0.168

Online resource (suppplementary table) 3. Selected attributes of GAMA-PBP (https://ca.water.usgs.gov/gama/) trend wells evaluated for step trends in orthophosphate concentration-page 13.

GAMA-PBP ID	USGS Station ID[1]	GAMA-PBP project study unit	GAMA-PBP study area[3]	Hydrogeologic Zone
DM-12	373600121170001	Western San Joaquin Valley	Delta-Mendota subbasin	Central Valley
DM-19	365000120400001	Western San Joaquin Valley	Delta-Mendota subbasin	Central Valley
DM-26	371000121010001	Western San Joaquin Valley	Delta-Mendota subbasin	Central Valley
WS-07	362600120120001	Western San Joaquin Valley	Westside subbasin	Central Valley
SDALLV-01	332005117004101	San Diego Drainages hydrogeologic province	San Diego alluvial basin	Southern California
SDALLV-02	333147117401901	San Diego Drainages hydrogeologic province	San Diego alluvial basin	Southern California
SDALLV-03	323925117044001	San Diego Drainages hydrogeologic province	San Diego alluvial basin	Southern California
SDALLV-06	331334117202102	San Diego Drainages hydrogeologic province	San Diego alluvial basin	Southern California
SDALLV-09	324111117052601	San Diego Drainages hydrogeologic province	San Diego alluvial basin	Southern California
SDHDRK-04	332011116534301	San Diego Drainages hydrogeologic province	San Diego hard rock	Southern California
SDHDRK-05	330447117065301	San Diego Drainages hydrogeologic province	San Diego hard rock	Southern California
SDHDRK-07	330659116362101	San Diego Drainages hydrogeologic province	San Diego hard rock	Southern California
SDHDRK-09	323657116354401	San Diego Drainages hydrogeologic province	San Diego hard rock	Southern California
SDTEM-10	333010117003101	San Diego Drainages hydrogeologic province	Temecula Valley	Southern California
SDTEMFP-01	332845117064801	San Diego Drainages hydrogeologic province	Temecula Valley	Southern California
SDTEMFP-03	332922117025301	San Diego Drainages hydrogeologic province	Temecula Valley	Southern California
SDWARN-04	331508116422901	San Diego Drainages hydrogeologic province	Warner Valley	Southern California
SDWARN-06	331533116395601	San Diego Drainages hydrogeologic province	Warner Valley	Southern California
ULASF-08	340900118170001	San Fernando-San Gabriel	San Fernando Valley	Southern California
ULASF-09	341100118130001	San Fernando-San Gabriel	San Fernando Valley	Southern California
ULASF-10	341000118220001	San Fernando-San Gabriel	San Fernando Valley	Southern California
ULASG-01	340400117530001	San Fernando-San Gabriel	San Gabriel Valley	Southern California
ULASG-06	340148118030901	San Fernando-San Gabriel	San Gabriel Valley	Southern California
ULASG-08	340835118055401	San Fernando-San Gabriel	San Gabriel Valley	Southern California
ULASG-11	341100118092001	San Fernando-San Gabriel	San Gabriel Valley	Southern California
ULASG-15	340900117550001	San Fernando-San Gabriel	San Gabriel Valley	Southern California
ULASG-17	340300117480001	San Fernando-San Gabriel	San Gabriel Valley	Southern California
CLABCB-12	335938118110501	Coastal Los Angeles Basin	Central Basin	Southern California
CLABCB-13	335643118105801	Coastal Los Angeles Basin	Central Basin	Southern California
CLABCB-14	335302118072201	Coastal Los Angeles Basin	Central Basin	Southern California
CLABCB-17	335712118054901	Coastal Los Angeles Basin	Central Basin	Southern California

Online resource (suppplementary table) 3. Selected attributes of GAMA-PBP (https://ca.water.usgs.gov/gama/) trend wells evaluated for step trends in orthophosphate concentration-page 14.

GAMA-PBP ID	Initial Sample Date	Initital Sample Orthophos-phate Concentra-tion (mg/L as P)	Orthophos-phate reporting level for initial sample (mg/L as P)	Triennial Trend Sample Date	Triennial Sample Orthophos-phate Concentra-tion (mg/L as P)	Orthophos-phate reporting level for triennial sample (mg/L as P)	Decadal Trend Sample Date	Decadal Sample Orthophos-phate Concentra-tion (mg/L as P)	Orthophos-phate reporting level for decadal sample (mg/L as P)	Performed Evaluation 1 (comparison between initial and triennial results)	Performed Evaluation 2 (comparison between initial and decadal results)	Performed Evaluation 3 (comparison between triennial and decadal results)
DM-12	3/11/2010	0.027	0.008	4/2/2013	0.023	0.004	pending	na	na	yes	no	no
DM-19	4/14/2010	0.022	0.008	4/2/2013	0.017	0.004	pending	na	na	yes	no	no
DM-26	6/17/2010	0.029	0.008	4/3/2013	0.025	0.004	pending	na	na	yes	no	no
WS-07	6/10/2010	0.028	0.008	4/3/2013	0.020	0.004	pending	na	na	yes	no	no
SDALLV-01	6/30/2004	0.021	0.006	none	na	na	5/1/2014	0.031	0.004	no	yes	no
SDALLV-02	7/1/2004	0.016	0.006	none	na	na	4/30/2014	0.021	0.004	no	yes	no
SDALLV-03	7/12/2004	0.022	0.006	none	na	na	5/2/2014	0.025	0.004	no	yes	no
SDALLV-06	7/13/2004	0.062	0.006	none	na	na	8/12/2014	0.321	0.004	no	yes	no
SDALLV-09	7/14/2004	0.008	0.006	none	na	na	5/2/2014	0.015	0.004	no	yes	no
SDHDRK-04	7/19/2004	0.003	0.006	none	na	na	5/15/2014	0.010	0.004	no	yes	no
SDHDRK-05	7/20/2004	0.026	0.006	none	na	na	4/30/2014	0.061	0.004	no	yes	no
SDHDRK-07	7/22/2004	0.031	0.006	none	na	na	4/21/2014	0.030	0.004	no	yes	no
SDHDRK-09	7/27/2004[4]	na	na	9/11/2007	0.056	0.006	4/29/2014	0.045	0.004	no	no	yes
SDTEM-10	7/26/2004	0.037	0.006	9/17/2007	0.046	0.006	4/23/2014	0.027	0.004	yes	yes	yes
SDTEMFP-01	5/19/2004	0.015	0.006	none	na	na	8/12/2014	0.017	0.004	no	yes	no
SDTEMFP-03	6/14/2004	0.013	0.006	9/19/2007	0.013	0.006	none	na	na	yes	no	no
SDWARN-04	6/24/2004	0.048	0.006	none	na	na	4/21/2014	0.055	0.004	no	yes	no
SDWARN-06	6/29/2004	0.049	0.006	9/11/2007[4]	na	na	4/22/2014	0.083	0.004	no	yes	no
ULASF-08	6/6/2005	0.015	0.006	none	na	na	8/11/2015	0.094	0.004	no	yes	no
ULASF-09	6/7/2005	0.024	0.006	6/16/2008	0.036	0.006	8/11/2015	0.044	0.004	yes	yes	yes
ULASF-10	6/8/2005	0.022	0.006	6/16/2008	0.021	0.006	none	na	na	yes	no	no
ULASG-01	6/7/2005[4]	na	na	6/16/2008	0.066	0.006	8/10/2015	0.065	0.004	no	no	yes
ULASG-06	6/14/2005	0.008	0.006	none	na	na	8/13/2015	0.014	0.004	no	yes	no
ULASG-08	6/15/2005	0.018	0.006	6/17/2008	0.028	0.006	none	na	na	yes	no	no
ULASG-11	6/16/2005	0.020	0.006	none	na	na	8/12/2015	0.032	0.004	no	yes	no
ULASG-15	6/23/2005	0.003	0.006	6/17/2008	0.009	0.006	8/12/2015	0.009	0.004	yes	yes	yes
ULASG-17	7/11/2005[4]	na	na	6/17/2008	0.028	0.006	8/10/2015	0.032	0.004	no	no	yes
CLABCB-12	8/29/2006[4]	na	na	8/25/2010	0.036	0.008	7/20/2016	0.032	0.004	no	no	yes
CLABCB-13	8/29/2006[4]	na	na	8/23/2010	0.045	0.008	7/18/2016	0.045	0.004	no	no	yes
CLABCB-14	8/29/2006[4]	na	na	8/23/2010	0.020	0.008	7/18/2016	0.020	0.004	no	no	yes
CLABCB-17	9/14/2006	0.025	0.006	8/25/2010	0.030	0.008	7/20/2016	0.026	0.004	yes	yes	yes

Online resource (suppplementary table) 3. Selected attributes of GAMA-PBP (https://ca.water.usgs.gov/gama/) trend wells evaluated for step trends in orthophosphate concentration-page 15.

GAMA-PBP ID	Elevation of LSD (meters above NAVD 88)[6]	Well depth (meters below LSD)	Agricultural land use in 1974[9] (percent)	Natural land use in 1974[9] (percent)	Urban land use in 1974[9] (percent)	Agricultural land use in 1982[9] (percent)	Natural land use in 1982[9] (percent)	Urban land use in 1982[9] (percent)	Agricultural land use in 1992[9] (percent)	Natural land use in 1992[9] (percent)	Urban land use in 1992[9] (percent)
DM-12	43	115	98.2%	0.9%	0.9%	98.2%	0.9%	0.9%	98.2%	0.9%	0.9%
DM-19	75	115	98.6%	0.5%	0.9%	98.6%	0.5%	0.9%	98.6%	0.0%	1.4%
DM-26	47	92	95.4%	0.5%	4.1%	95.4%	0.5%	4.1%	95.4%	0.5%	4.1%
WS-07	78	335	99.1%	0.9%	0.0%	99.1%	0.9%	0.0%	98.1%	0.9%	0.9%
SDALLV-01	230	66	63.3%	34.0%	2.8%	63.3%	34.0%	2.8%	63.3%	33.5%	3.3%
SDALLV-02	69	43	9.1%	0.0%	90.9%	8.7%	0.0%	91.3%	0.5%	0.0%	99.5%
SDALLV-03	7	199	0.0%	0.0%	100.0%	0.0%	0.0%	100.0%	0.0%	0.0%	100.0%
SDALLV-06	13	66	2.7%	0.0%	97.3%	0.5%	0.0%	99.5%	0.0%	0.0%	100.0%
SDALLV-09	31	266	0.0%	0.0%	100.0%	0.0%	0.0%	100.0%	0.0%	0.0%	100.0%
SDHDRK-04	1561	103	0.0%	100.0%	0.0%	0.0%	100.0%	0.0%	0.0%	100.0%	0.0%
SDHDRK-05	112	148	0.0%	0.0%	100.0%	0.0%	0.0%	100.0%	0.0%	0.0%	100.0%
SDHDRK-07	1278	131	0.0%	99.5%	0.5%	1.4%	97.3%	1.4%	0.0%	96.3%	3.7%
SDHDRK-09	788	131	0.0%	97.7%	2.3%	0.0%	96.3%	3.7%	0.0%	89.8%	10.2%
SDTEM-10	417	82	1.4%	97.7%	0.9%	1.4%	97.7%	0.9%	1.4%	97.7%	0.9%
SDTEMFP-01	347	820	11.4%	64.5%	24.1%	29.5%	5.0%	65.5%	10.9%	9.1%	80.0%
SDTEMFP-03	386	284	19.9%	74.5%	5.6%	25.9%	68.5%	5.6%	19.4%	75.0%	5.6%
SDWARN-04	907	144	4.1%	94.9%	0.9%	4.1%	94.9%	0.9%	4.1%	94.9%	0.9%
SDWARN-06	971	240	0.0%	99.1%	0.9%	0.0%	99.1%	0.9%	0.0%	99.1%	0.9%
ULASF-08	153	131	0.0%	0.9%	99.1%	0.0%	0.9%	99.1%	0.0%	0.9%	99.1%
ULASF-09	333	60	0.0%	0.0%	100.0%	0.0%	0.0%	100.0%	0.0%	0.0%	100.0%
ULASF-10	217	305	0.0%	0.0%	100.0%	0.0%	0.0%	100.0%	0.0%	0.0%	100.0%
ULASG-01	152	266	0.0%	0.0%	100.0%	0.0%	0.0%	100.0%	0.0%	0.0%	100.0%
ULASG-06	66	234	5.6%	0.0%	94.4%	5.6%	0.0%	94.4%	0.0%	0.0%	100.0%
ULASG-08	236	131	0.0%	0.0%	100.0%	0.0%	0.0%	100.0%	0.0%	0.0%	100.0%
ULASG-11	378	161	0.0%	0.0%	100.0%	0.0%	0.0%	100.0%	0.0%	0.0%	100.0%
ULASG-15	224	na	0.0%	26.0%	74.0%	0.0%	26.0%	74.0%	0.0%	26.0%	74.0%
ULASG-17	236	61	32.2%	0.0%	67.8%	9.3%	0.0%	90.7%	2.3%	0.0%	97.7%
CLABCB-12	52	388	0.0%	0.0%	100.0%	0.0%	0.0%	100.0%	0.0%	0.0%	100.0%
CLABCB-13	35	245	0.0%	0.0%	100.0%	0.0%	0.0%	100.0%	0.0%	0.0%	100.0%
CLABCB-14	23	164	0.0%	0.0%	100.0%	0.0%	0.0%	100.0%	0.0%	0.0%	100.0%
CLABCB-17	46	206	0.0%	0.0%	100.0%	0.0%	0.0%	100.0%	0.0%	0.0%	100.0%

Online resource (suppplementary table) 3. Selected attributes of GAMA-PBP (https://ca.water.usgs.gov/gama/) trend wells evaluated for step trends in orthophosphate concentration-page 16.

GAMA-PBP ID	Agricultural land use in 2002[9] (percent)	Natural land use in 2002[9] (percent)	Urban land use in 2002[9] (percent)	Agricultural land use in 2012[9] (percent)	Natural land use in 2012[9] (percent)	Urban land use in 2012[9] (percent)	Age Classification[8]	Septic Tanks[10]	Aridity[11]
DM-12	98.2%	0.5%	1.4%	98.2%	0.5%	1.4%	Mixed	1.79	0.208
DM-19	98.6%	0.0%	1.4%	98.6%	0.0%	1.4%	Premodern	0.34	0.167
DM-26	95.0%	0.5%	4.6%	95.0%	0.0%	5.0%	Modern	1.53	0.204
WS-07	98.1%	0.9%	0.9%	98.1%	0.9%	0.9%	Premodern	0.31	0.137
SDALLV-01	67.4%	29.3%	3.3%	67.4%	29.3%	3.3%	Modern	3.51	0.305
SDALLV-02	0.0%	0.0%	100.0%	0.0%	0.0%	100.0%	Modern	9.70	0.250
SDALLV-03	0.0%	0.0%	100.0%	0.0%	0.0%	100.0%	Premodern	34.04	0.190
SDALLV-06	0.0%	0.0%	100.0%	0.0%	0.0%	100.0%	Modern	0.05	0.228
SDALLV-09	0.0%	0.0%	100.0%	0.0%	0.0%	100.0%	Premodern	0.05	0.195
SDHDRK-04	0.0%	100.0%	0.0%	0.0%	100.0%	0.0%	Modern	0.66	0.540
SDHDRK-05	0.0%	0.0%	100.0%	0.0%	0.0%	100.0%	Modern	65.63	0.277
SDHDRK-07	0.0%	96.3%	3.7%	0.0%	96.3%	3.7%	Mixed	1.01	0.577
SDHDRK-09	0.0%	84.7%	15.3%	0.0%	84.7%	15.3%	ModernOrMixed	1.70	0.348
SDTEM-10	2.7%	96.3%	0.9%	2.7%	96.3%	0.9%	Modern	3.01	0.337
SDTEMFP-01	7.7%	9.1%	83.2%	3.2%	2.7%	94.1%	Mixed	9.89	0.297
SDTEMFP-03	15.7%	74.5%	9.7%	15.7%	74.5%	9.7%	Mixed	2.95	0.276
SDWARN-04	4.1%	94.9%	0.9%	4.1%	94.9%	0.9%	Premodern	0.66	0.421
SDWARN-06	0.0%	98.2%	1.8%	0.0%	98.2%	1.8%	Premodern	0.66	0.380
ULASF-08	0.0%	0.9%	99.1%	0.0%	0.9%	99.1%	Mixed	0.00	0.319
ULASF-09	0.0%	0.0%	100.0%	0.0%	0.0%	100.0%	Modern	1.21	0.397
ULASF-10	0.0%	0.0%	100.0%	0.0%	0.0%	100.0%	Mixed	0.79	0.307
ULASG-01	0.0%	0.0%	100.0%	0.0%	0.0%	100.0%	ModernOrMixed	4.14	0.319
ULASG-06	0.0%	0.0%	100.0%	0.0%	0.0%	100.0%	Modern	0.05	0.264
ULASG-08	0.0%	0.0%	100.0%	0.0%	0.0%	100.0%	Modern	169.23	0.349
ULASG-11	0.0%	0.0%	100.0%	0.0%	0.0%	100.0%	ModernOrMixed	22.90	0.390
ULASG-15	0.0%	26.0%	74.0%	0.0%	26.0%	74.0%	Modern	0.00	0.380
ULASG-17	2.3%	0.0%	97.7%	0.5%	0.0%	99.5%	ModernOrMixed	0.00	0.304
CLABCB-12	0.0%	0.0%	100.0%	0.0%	0.0%	100.0%	ModernOrMixed	48.99	0.263
CLABCB-13	0.0%	0.0%	100.0%	0.0%	0.0%	100.0%	ModernOrMixed	14.34	0.266
CLABCB-14	0.0%	0.0%	100.0%	0.0%	0.0%	100.0%	PremodernOrMixed	8.20	0.252
CLABCB-17	0.0%	0.0%	100.0%	0.0%	0.0%	100.0%	ModernOrMixed	0.40	0.263

Online resource (suppplementary table) 3. Selected attributes of GAMA-PBP (https://ca.water.usgs.gov/gama/) trend wells evaluated for step trends in orthophosphate concentration-page 17.

GAMA-PBP ID	USGS Station ID[1]	GAMA-PBP project study unit	GAMA-PBP study area[3]	Hydrogeologic Zone
CLABDA-02	340152118273601	Coastal Los Angeles Basin	Santa Monica basin	Southern California
CLABOC-01	334631117504101	Coastal Los Angeles Basin	Orange County Coastal Plain	Southern California
CLABOC-09	334327117511501	Coastal Los Angeles Basin	Orange County Coastal Plain	Southern California
CLABOC-13	335145117494101	Coastal Los Angeles Basin	Orange County Coastal Plain	Southern California
CLABOC-14	334552118020201	Coastal Los Angeles Basin	Orange County Coastal Plain	Southern California
CLABWB-03	335314118223801	Coastal Los Angeles Basin	West Coast basin	Southern California
CLABWB-04	335800118230001	Coastal Los Angeles Basin	West Coast basin	Southern California
USAWB-01	340332117164201	Upper Santa Ana Watershed	Bunker Hill and Rialto-Colton subbasins	Southern California
USAWB-02	341018117253201	Upper Santa Ana Watershed	Bunker Hill and Rialto-Colton subbasins	Southern California
USAWB-04	340904117221001	Upper Santa Ana Watershed	Bunker Hill and Rialto-Colton subbasins	Southern California
USAWB-11	340858117152002	Upper Santa Ana Watershed	Bunker Hill and Rialto-Colton subbasins	Southern California
USAWB-12	340627117101401	Upper Santa Ana Watershed	Bunker Hill and Rialto-Colton subbasins	Southern California
USAWB-14	340700117240001	Upper Santa Ana Watershed	Bunker Hill and Rialto-Colton subbasins	Southern California
USAWC-01	340300117270001	Upper Santa Ana Watershed	Cucamonga and Chino subbasins	Southern California
USAWC-02	340500117280001	Upper Santa Ana Watershed	Cucamonga and Chino subbasins	Southern California
USAWC-08	340103117312601	Upper Santa Ana Watershed	Cucamonga and Chino subbasins	Southern California
USAWC-10	335800117390001	Upper Santa Ana Watershed	Cucamonga and Chino subbasins	Southern California
USAWC-21	340830117355101	Upper Santa Ana Watershed	Cucamonga and Chino subbasins	Southern California
USAWC-23	335924117412201	Upper Santa Ana Watershed	Cucamonga and Chino subbasins	Southern California
USAWE-02	333844117190801	Upper Santa Ana Watershed	Elsinore	Southern California
USAWR-08	340033117204001	Upper Santa Ana Watershed	Riverside-Arlington and Temescal subbasins	Southern California
USAWR-12	335224117351101	Upper Santa Ana Watershed	Riverside-Arlington and Temescal subbasins	Southern California
USAWS-01	335544117165201	Upper Santa Ana Watershed	San Jacinto basin	Southern California
USAWS-08	335646117143201	Upper Santa Ana Watershed	San Jacinto basin	Southern California
USAWS-12	335053117135801	Upper Santa Ana Watershed	San Jacinto basin	Southern California
USAWS-14	334621116564601	Upper Santa Ana Watershed	San Jacinto basin	Southern California
USAWY-05	340105117031601	Upper Santa Ana Watershed	Yucaipa and San Timoteo subbasins	Southern California
USAWY-06	335911117025501	Upper Santa Ana Watershed	Yucaipa and San Timoteo subbasins	Southern California
USAWY-07	340054117002901	Upper Santa Ana Watershed	Yucaipa and San Timoteo subbasins	Southern California
SCRV-06	341204119103001	Santa Clara River Valley	Santa Clara River Valley basins	Southern California
SCRV-08	341300119040001	Santa Clara River Valley	Santa Clara River Valley basins	Southern California

Online resource (suppplementary table) 3. Selected attributes of GAMA-PBP (https://ca.water.usgs.gov/gama/) trend wells evaluated for step trends in orthophosphate concentration-page 18.

GAMA-PBP ID	Initial Sample Date	Initital Sample Orthophos-phate Concentra-tion (mg/L as P)	Orthophos-phate reporting level for initial sample (mg/L as P)	Triennial Trend Sample Date	Triennial Sample Orthophos-phate Concentra-tion (mg/L as P)	Orthophos-phate reporting level for triennial sample (mg/L as P)	Decadal Trend Sample Date	Decadal Sample Orthophos-phate Concentra-tion (mg/L as P)	Orthophos-phate reporting level for decadal sample (mg/L as P)	Performed Evaluation 1 (comparison between initial and triennial results)	Performed Evaluation 2 (comparison between initial and decadal results)	Performed Evaluation 3 (comparison between triennial and decadal results)
CLABDA-02	8/8/2006	0.110	0.006	8/24/2010	0.109	0.008	7/19/2016	0.105	0.004	yes	yes	yes
CLABOC-01	6/5/2006	0.023	0.006	6/4/2009	0.034	0.008	7/27/2016	0.047	0.004	yes	yes	yes
CLABOC-09	8/24/2006[4]	na	na	6/3/2009	0.039	0.008	8/18/2016	0.028	0.004	no	no	yes
CLABOC-13	8/28/2006[4]	na	na	8/26/2010	0.130	0.008	7/21/2016	0.142	0.004	no	no	yes
CLABOC-14	8/31/2006[4]	na	na	8/26/2010	0.028	0.008	7/28/2016	0.023	0.004	no	no	yes
CLABWB-03	8/30/2006[4]	na	na	8/24/2010	0.049	0.008	7/15/2016	0.127	0.004	no	no	yes
CLABWB-04	9/12/2006	0.160	0.006	none	na	na	7/26/2016	0.162	0.004	no	yes	no
USAWB-01	11/27/2006[4]	na	na	4/20/2009	0.020	0.008	1/31/2017	0.018	0.004	no	no	yes
USAWB-02	11/27/2006	0.012	0.006	none	na	na	1/31/2017	0.015	0.004	no	yes	no
USAWB-04	11/28/2006	0.018	0.006	5/6/2009	0.023	0.008	none	na	na	yes	no	no
USAWB-11	12/13/2006	0.038	0.006	none	0.040	0.008	2/1/2017	0.042	0.004	no	yes	no
USAWB-12	12/13/2006[4]	na	na	4/22/2009	0.021	0.008	2/1/2017	0.018	0.004	no	no	yes
USAWB-14	12/14/2006	0.028	0.006	4/14/2009	0.035	0.008	1/24/2017	0.028	0.004	yes	yes	yes
USAWC-01	1/29/2007	0.018	0.006	none	na	na	1/24/2017	0.017	0.004	no	yes	no
USAWC-02	1/29/2007	0.039	0.006	4/30/2009	0.045	0.008	none	na	na	yes	no	no
USAWC-08	1/31/2007	0.016	0.006	4/28/2009	0.019	0.008	1/25/2017	0.014	0.004	yes	yes	yes
USAWC-10	2/1/2007[4]	na	na	4/27/2009	0.015	0.008	1/26/2017	0.013	0.004	no	no	yes
USAWC-21	2/14/2007	0.043	0.006	4/13/2009	0.048	0.008	1/23/2017	0.043	0.004	yes	yes	yes
USAWC-23	2/15/2007	0.015	0.006	4/16/2009	0.019	0.008	1/23/2017	0.017	0.004	yes	yes	yes
USAWE-02	12/5/2006	0.027	0.006	none	na	na	2/13/2017	0.035	0.004	no	yes	no
USAWR-08	1/10/2007	0.017	0.006	4/21/2009	0.020	0.008	2/2/2017	0.020	0.004	yes	yes	yes
USAWR-12	1/29/2007	0.020	0.006	4/29/2009	0.021	0.008	1/26/2017	0.017	0.004	yes	yes	yes
USAWS-01	1/22/2007[4]	na	na	5/7/2009	0.088	0.008	2/15/2017	0.081	0.004	no	no	yes
USAWS-08	1/24/2007	0.075	0.006	5/7/2009	0.074	0.008	none	na	na	yes	no	no
USAWS-12	1/25/2007	0.029	0.006	none	na	na	2/14/2017	0.033	0.004	no	yes	no
USAWS-14	2/5/2007	0.048	0.006	none	na	na	2/14/2017	0.038	0.004	no	yes	no
USAWY-05	1/8/2007	0.026	0.006	none	na	na	1/30/2017	0.021	0.004	no	yes	no
USAWY-06	1/9/2007	0.024	0.006	5/5/2009	0.026	0.008	none	na	na	yes	no	no
USAWY-07	1/11/2007[4]	na	na	5/4/2009	0.052	0.008	1/30/2017	0.051	0.004	no	no	yes
SCRV-06	4/4/2007	0.048	0.006	4/26/2011	0.062	0.004	none	na	na	yes	no	no
SCRV-08	4/4/2007[4]	na	na	4/26/2011	0.040	0.004	6/28/2017	0.022	0.004	no	no	yes

Online resource (supplementary table) 3. Selected attributes of GAMA-PBP (https://ca.water.usgs.gov/gama/) trend wells evaluated for step trends in orthophosphate concentration-page 19.

GAMA-PBP ID	Elevation of LSD (meters above NAVD 88)[6]	Well depth (meters below LSD)	Agricultural land use in 1974[9] (percent)	Natural land use in 1974[9] (percent)	Urban land use in 1974[9] (percent)	Agricultural land use in 1982[9] (percent)	Natural land use in 1982[9] (percent)	Urban land use in 1982[9] (percent)	Agricultural land use in 1992[9] (percent)	Natural land use in 1992[9] (percent)	Urban land use in 1992[9] (percent)
CLABDA-02	52	180	0.0%	0.0%	100.0%	0.0%	0.0%	100.0%	0.0%	0.0%	100.0%
CLABOC-01	66	427	10.5%	0.0%	89.5%	2.7%	0.0%	97.3%	0.0%	0.0%	100.0%
CLABOC-09	26	378	3.8%	0.0%	96.2%	0.9%	0.0%	99.1%	0.0%	0.0%	100.0%
CLABOC-13	83	138	36.2%	0.5%	63.3%	13.6%	0.5%	86.0%	0.0%	0.5%	99.5%
CLABOC-14	8	289	0.0%	0.0%	100.0%	0.0%	0.0%	100.0%	0.0%	0.0%	100.0%
CLABWB-03	30	146	0.0%	0.0%	100.0%	0.0%	0.0%	100.0%	0.0%	0.0%	100.0%
CLABWB-04	25	203	0.0%	0.0%	100.0%	0.0%	0.0%	100.0%	0.0%	0.0%	100.0%
USAWB-01	354	350	0.0%	0.0%	100.0%	0.0%	0.0%	100.0%	0.0%	0.0%	100.0%
USAWB-02	609	73	0.0%	77.3%	22.7%	0.0%	36.4%	63.6%	0.0%	35.0%	65.0%
USAWB-04	466	295	0.0%	82.5%	17.5%	0.0%	77.4%	22.6%	0.0%	77.0%	23.0%
USAWB-11	410	199	0.0%	0.0%	100.0%	0.0%	0.0%	100.0%	0.0%	0.0%	100.0%
USAWB-12	437	312	3.6%	0.0%	96.4%	1.8%	0.0%	98.2%	0.0%	0.0%	100.0%
USAWB-14	465	348	14.1%	0.0%	85.9%	14.1%	0.0%	85.9%	0.0%	0.0%	100.0%
USAWC-01	354	261	1.4%	0.0%	98.6%	0.0%	0.0%	100.0%	0.0%	0.0%	100.0%
USAWC-02	385	285	5.2%	0.0%	94.8%	0.9%	0.0%	99.1%	0.0%	0.0%	100.0%
USAWC-08	257	121	9.6%	5.5%	84.9%	7.8%	5.5%	86.8%	5.9%	5.5%	88.6%
USAWC-10	205	171	20.5%	0.0%	79.5%	20.5%	0.0%	79.5%	20.0%	0.0%	80.0%
USAWC-21	536	364	44.7%	0.5%	54.8%	23.5%	0.5%	76.0%	0.0%	0.0%	100.0%
USAWC-23	208	387	62.3%	0.0%	37.7%	50.0%	0.0%	50.0%	44.5%	0.0%	55.5%
USAWE-02	409	564	0.0%	94.5%	5.5%	0.0%	89.9%	10.1%	0.0%	84.4%	15.6%
USAWR-08	295	132	0.0%	0.0%	100.0%	0.0%	0.0%	100.0%	0.0%	0.0%	100.0%
USAWR-12	240	169	0.0%	0.0%	100.0%	0.0%	0.0%	100.0%	0.0%	0.0%	100.0%
USAWS-01	508	74	0.0%	0.0%	100.0%	0.5%	0.0%	99.5%	0.0%	0.0%	100.0%
USAWS-08	543	140	6.4%	0.0%	93.6%	11.9%	0.0%	88.1%	0.0%	0.0%	100.0%
USAWS-12	480	249	16.9%	76.3%	6.8%	63.9%	24.2%	11.9%	13.2%	66.7%	20.1%
USAWS-14	523	509	43.5%	0.0%	56.5%	15.0%	0.0%	85.0%	7.9%	0.0%	92.1%
USAWY-05	802	194	0.0%	0.0%	100.0%	0.0%	0.0%	100.0%	0.0%	0.0%	100.0%
USAWY-06	802	259	0.5%	12.2%	87.3%	0.5%	12.2%	87.3%	0.5%	12.2%	87.3%
USAWY-07	945	103	8.9%	22.1%	69.0%	8.0%	21.1%	70.9%	2.3%	15.0%	82.6%
SCRV-06	19	72	0.0%	0.0%	100.0%	0.0%	0.0%	100.0%	0.0%	0.0%	100.0%
SCRV-08	31	299	47.7%	0.0%	52.3%	50.9%	0.0%	49.1%	50.5%	0.0%	49.5%

Online resource (suppplementary table) 3. Selected attributes of GAMA-PBP (https://ca.water.usgs.gov/gama/) trend wells evaluated for step trends in orthophosphate concentration-page 20.

GAMA-PBP ID	Agricultural land use in 2002[9] (percent)	Natural land use in 2002[9] (percent)	Urban land use in 2002[9] (percent)	Agricultural land use in 2012[9] (percent)	Natural land use in 2012[9] (percent)	Urban land use in 2012[9] (percent)	Age Classification[8]	Septic Tanks[10]	Aridity[11]
CLABDA-02	0.0%	0.0%	100.0%	0.0%	0.0%	100.0%	ModernOrMixed	4.46	0.278
CLABOC-01	0.0%	0.0%	100.0%	0.0%	0.0%	100.0%	ModernOrMixed	9.97	0.248
CLABOC-09	0.0%	0.0%	100.0%	0.0%	0.0%	100.0%	ModernOrMixed	0.00	0.237
CLABOC-13	0.0%	0.5%	99.5%	0.0%	0.5%	99.5%	ModernOrMixed	2.11	0.248
CLABOC-14	0.0%	0.0%	100.0%	0.0%	0.0%	100.0%	PremodernOrMixed	0.00	0.215
CLABWB-03	0.0%	0.0%	100.0%	0.0%	0.0%	100.0%	ModernOrMixed	0.00	0.247
CLABWB-04	0.0%	0.0%	100.0%	0.0%	0.0%	100.0%	Premodern	0.00	0.259
USAWB-01	0.0%	0.0%	100.0%	0.0%	0.0%	100.0%	PremodernOrMixed	6.05	0.224
USAWB-02	0.0%	30.0%	70.0%	0.0%	25.9%	74.1%	Modern	1.70	0.464
USAWB-04	0.0%	77.0%	23.0%	0.0%	77.0%	23.0%	Modern	4.39	0.354
USAWB-11	0.0%	0.0%	100.0%	0.0%	0.0%	100.0%	Modern	1.64	0.300
USAWB-12	0.0%	0.0%	100.0%	0.0%	0.0%	100.0%	ModernOrMixed	19.27	0.252
USAWB-14	0.0%	0.0%	100.0%	0.0%	0.0%	100.0%	Mixed	17.58	0.371
USAWC-01	0.0%	0.0%	100.0%	0.0%	0.0%	100.0%	Premodern	74.53	0.285
USAWC-02	0.0%	0.0%	100.0%	0.0%	0.0%	100.0%	Mixed	238.53	0.303
USAWC-08	6.4%	5.0%	88.6%	0.0%	0.0%	100.0%	Mixed	1.86	0.273
USAWC-10	19.5%	0.0%	80.5%	19.5%	0.0%	80.5%	ModernOrMixed	4.24	0.287
USAWC-21	0.0%	0.0%	100.0%	0.0%	0.0%	100.0%	Mixed	68.43	0.375
USAWC-23	35.0%	0.0%	65.0%	18.2%	0.0%	81.8%	Premodern	0.00	0.294
USAWE-02	0.0%	78.9%	21.1%	0.0%	3.7%	96.3%	ModernOrMixed	7.30	0.199
USAWR-08	0.0%	0.0%	100.0%	0.0%	0.0%	100.0%	Modern	128.39	0.191
USAWR-12	0.0%	0.0%	100.0%	0.0%	0.0%	100.0%	Mixed	7.12	0.248
USAWS-01	0.0%	0.0%	100.0%	0.0%	0.0%	100.0%	ModernOrMixed	3.36	0.202
USAWS-08	0.0%	0.0%	100.0%	0.0%	0.0%	100.0%	Modern	25.69	0.212
USAWS-12	37.4%	34.2%	28.3%	37.4%	28.3%	34.2%	ModernOrMixed	17.98	0.187
USAWS-14	1.9%	0.0%	98.1%	0.0%	0.0%	100.0%	Mixed	14.51	0.230
USAWY-05	0.0%	0.0%	100.0%	0.0%	0.0%	100.0%	Mixed	15.87	0.329
USAWY-06	0.5%	12.2%	87.3%	0.5%	12.2%	87.3%	Modern	23.84	0.312
USAWY-07	0.9%	14.6%	84.5%	0.9%	4.7%	94.4%	ModernOrMixed	14.97	0.399
SCRV-06	0.0%	0.0%	100.0%	0.0%	0.0%	100.0%	ModernOrMixed	12.94	0.284
SCRV-08	32.3%	0.0%	67.7%	25.9%	0.0%	74.1%	PremodernOrMixed	21.14	0.272

Online resource (suppplementary table) 3. Selected attributes of GAMA-PBP (https://ca.water.usgs.gov/gama/) trend wells evaluated for step trends in orthophosphate concentration-page 21.

GAMA-PBP ID	USGS Station ID[1]	GAMA-PBP project study unit	GAMA-PBP study area[3]	Hydrogeologic Zone
SCRV-12	340932119111101	Santa Clara River Valley	Santa Clara River Valley basins	Southern California
SCRV-16	342123119024201	Santa Clara River Valley	Santa Clara River Valley basins	Southern California
SCRV-17	342100119180001	Santa Clara River Valley	Santa Clara River Valley basins	Southern California
SCRV-18	341700119090001	Santa Clara River Valley	Santa Clara River Valley basins	Southern California
SCRV-19	342200118520001	Santa Clara River Valley	Santa Clara River Valley basins	Southern California
SCRV-32	341600118470001	Santa Clara River Valley	Santa Clara River Valley basins	Southern California
OIW-05	353601117483802	Owens and Indian Wells Valleys	Indian Wells Valley	Desert
OIW-07	353740117414301	Owens and Indian Wells Valleys	Indian Wells Valley	Desert
OIW-12	354711117464001	Owens and Indian Wells Valleys	Indian Wells Valley	Desert
OIWU-01	353900117460001	Owens and Indian Wells Valleys	Indian Wells Valley	Desert
OV-06	365600118140001	Owens and Indian Wells Valleys	Owens Valley	Desert
OV-11	371900118320001	Owens and Indian Wells Valleys	Owens Valley	Desert
OV-12	363500118110001	Owens and Indian Wells Valleys	Owens Valley	Desert
OV-16	370500118150001	Owens and Indian Wells Valleys	Owens Valley	Desert
OV-21	364300118080001	Owens and Indian Wells Valleys	Owens Valley	Desert
OV-22	364800118110001	Owens and Indian Wells Valleys	Owens Valley	Desert
OV-24	370900118160001	Owens and Indian Wells Valleys	Owens Valley	Desert
OV-29	374900118280001	Owens and Indian Wells Valleys	Owens Valley	Desert
OV-36	361400117580001	Owens and Indian Wells Valleys	Owens Valley	Desert
COA-01	335532116471701	Coachella Valley	Coachella Valley	Desert
COA-04	334400116160001	Coachella Valley	Coachella Valley	Desert
COA-12	333300116020001	Coachella Valley	Coachella Valley	Desert
COA-14	334551116242101	Coachella Valley	Coachella Valley	Desert
COA-15	335500116320001	Coachella Valley	Coachella Valley	Desert
COA-16	334700116100001	Coachella Valley	Coachella Valley	Desert
COLOR-03	324400114380001	Colorado River	Colorado River Valleys	Desert
COLOR-06	333646114360901	Colorado River	Colorado River Valleys	Desert
COLOR-10	333130114394001	Colorado River	Colorado River Valleys	Desert
COLOR-14	324452114443101	Colorado River	Colorado River Valleys	Desert
COLOR-17	344300114310001	Colorado River	Colorado River Valleys	Desert
ANT-07	345006118125701	Antelope Valley	Antelope Valley	Desert

Online resource (suppplementary table) 3. Selected attributes of GAMA-PBP (https://ca.water.usgs.gov/gama/) trend wells evaluated for step trends in orthophosphate concentration-page 22.

GAMA-PBP ID	Initial Sample Date	Initital Sample Orthophos-phate Concentra-tion (mg/L as P)	Orthophos-phate reporting level for initial sample (mg/L as P)	Triennial Trend Sample Date	Triennial Sample Orthophos-phate Concentra-tion (mg/L as P)	Orthophos-phate reporting level for triennial sample (mg/L as P)	Decadal Trend Sample Date	Decadal Sample Orthophos-phate Concentra-tion (mg/L as P)	Orthophos-phate reporting level for decadal sample (mg/L as P)	Performed Evaluation 1 (comparison between initial and triennial results)	Performed Evaluation 2 (comparison between initial and decadal results)	Performed Evaluation 3 (comparison between triennial and decadal results)
SCRV-12	4/9/2007	0.034	0.006	none	na	na	8/10/2017	0.039	0.004	no	yes	no
SCRV-16	4/10/2007	0.102	0.006	none	na	na	6/28/2017	0.089	0.004	no	yes	no
SCRV-17	4/11/2007[4]	na	na	4/27/2011	0.047	0.004	6/27/2017	0.039	0.004	no	no	yes
SCRV-18	4/11/2007[4]	na	na	4/27/2011	0.055	0.004	6/27/2017	0.950	0.004	no	no	yes
SCRV-19	4/11/2007	0.052	0.006	none	na	na	7/11/2017	0.057	0.004	no	yes	no
SCRV-32	4/18/2007[4]	na	na	4/25/2011	0.053	0.004	7/12/2017	0.033	0.004	no	no	yes
OIW-05	10/18/2006	0.025	0.006	10/27/2010	0.047	0.004	12/14/2016	0.028	0.004	yes	yes	yes
OIW-07	10/19/2006	0.018	0.006	10/27/2010	0.036	0.004	none	na	na	yes	no	no
OIW-12	12/7/2006	0.029	0.006	none	na	na	12/15/2016	0.035	0.004	no	yes	no
OIWU-01	10/17/2006	0.011	0.006	none	na	na	12/14/2016	0.011	0.004	no	yes	no
OV-06	9/12/2006	0.082	0.006	none	na	na	12/6/2016	0.080	0.004	no	yes	no
OV-11	9/14/2006	0.013	0.006	none	na	na	12/7/2016	0.013	0.004	no	yes	no
OV-12	9/14/2006	0.031	0.006	none	na	na	12/13/2016	0.036	0.004	no	yes	no
OV-16	9/20/2006	0.075	0.006	none	na	na	12/13/2016	0.068	0.004	no	yes	no
OV-21	10/3/2006	0.050	0.006	10/26/2010	0.061	0.004	12/8/2016	0.058	0.004	yes	yes	yes
OV-22	10/3/2006	0.020	0.006	none	na	na	12/8/2016	0.026	0.004	no	yes	no
OV-24	10/4/2006	0.022	0.006	10/28/2010	0.028	0.004	12/7/2016	0.023	0.004	yes	yes	yes
OV-29	10/5/2006	0.017	0.006	10/28/2010	0.032	0.004	12/6/2016	0.028	0.004	yes	yes	yes
OV-36	10/25/2006	0.054	0.006	10/26/2010	0.057	0.004	none	na	na	yes[5]	no	no
COA-01	2/27/2007	0.019	0.006	none	na	na	2/21/2017	0.018	0.004	no	yes	no
COA-04	3/1/2007	0.011	0.006	none	na	na	2/21/2017	0.012	0.004	no	yes	no
COA-12	3/8/2007	0.020	0.006	1/5/2011	0.021	0.004	2/22/2017	0.023	0.004	yes	yes	yes
COA-14	3/12/2007	0.012	0.006	1/4/2011	0.020	0.004	none	na	na	yes	no	no
COA-15	3/14/2007	0.018	0.006	1/6/2011	0.025	0.004	none	na	na	yes	no	no
COA-16	3/15/2007	0.006	0.006	1/5/2011	0.012	0.004	2/22/2017	0.006	0.004	yes	yes	yes
COLOR-03	10/2/2007	0.054	0.006	1/4/2011	0.060	0.004	10/12/2017	0.059	0.004	yes	yes	yes
COLOR-06	10/24/2007	0.016	0.006	1/3/2011	0.020	0.004	10/11/2017	0.018	0.004	yes	yes	yes
COLOR-10	11/6/2007	0.016	0.006	none	na	na	10/11/2017	0.019	0.004	no	yes	no
COLOR-14	11/28/2007	0.010	0.006	none	na	na	10/12/2017	0.010	0.004	no	yes[5]	no
COLOR-17	12/11/2007	0.007	0.006	1/3/2011	0.013	0.004	10/10/2017	0.007	0.004	yes	yes	yes
ANT-07	1/29/2008	0.014	0.006	2/15/2012	0.016	0.004	none	na	na	yes	no	no

Online resource (suppplementary table) 3. Selected attributes of GAMA-PBP (https://ca.water.usgs.gov/gama/) trend wells evaluated for step trends in orthophosphate concentration-page 23.

GAMA-PBP ID	Elevation of LSD (meters above NAVD 88)[6]	Well depth (meters below LSD)	Agricultural land use in 1974[9] (percent)	Natural land use in 1974[9] (percent)	Urban land use in 1974[9] (percent)	Agricultural land use in 1982[9] (percent)	Natural land use in 1982[9] (percent)	Urban land use in 1982[9] (percent)	Agricultural land use in 1992[9] (percent)	Natural land use in 1992[9] (percent)	Urban land use in 1992[9] (percent)
SCRV-12	7	394	0.0%	0.0%	100.0%	0.0%	0.0%	100.0%	0.0%	0.0%	100.0%
SCRV-16	93	230	7.2%	21.7%	71.0%	5.9%	21.7%	72.4%	4.5%	21.3%	74.2%
SCRV-17	83	20	0.5%	74.0%	25.6%	0.5%	74.0%	25.6%	0.5%	72.1%	27.4%
SCRV-18	53	138	16.0%	0.9%	83.1%	10.5%	0.5%	89.0%	9.6%	0.5%	90.0%
SCRV-19	155	35	78.1%	21.5%	0.5%	78.1%	21.5%	0.5%	78.1%	21.5%	0.5%
SCRV-32	238	98	0.0%	0.0%	100.0%	0.0%	0.0%	100.0%	0.0%	0.0%	100.0%
OIW-05	840	335	0.0%	100.0%	0.0%	0.0%	100.0%	0.0%	0.0%	100.0%	0.0%
OIW-07	766	203	0.0%	52.5%	47.5%	0.0%	47.0%	53.0%	0.0%	46.1%	53.9%
OIW-12	740	66	0.0%	100.0%	0.0%	0.0%	100.0%	0.0%	0.0%	100.0%	0.0%
OIWU-01	792	400	0.0%	90.3%	9.7%	0.0%	54.2%	45.8%	0.0%	54.2%	45.8%
OV-06	1262	64	0.0%	86.4%	13.6%	0.0%	86.4%	13.6%	0.0%	86.4%	13.6%
OV-11	1917	53	7.9%	92.1%	0.0%	9.3%	90.7%	0.0%	9.3%	90.7%	0.0%
OV-12	1960	41	0.0%	99.5%	0.5%	0.0%	99.5%	0.5%	0.0%	99.5%	0.5%
OV-16	1274	61	0.0%	100.0%	0.0%	0.0%	100.0%	0.0%	0.0%	100.0%	0.0%
OV-21	1265	84	0.0%	89.5%	10.5%	0.0%	89.5%	10.5%	0.0%	89.0%	11.0%
OV-22	1283	213	22.3%	35.8%	41.9%	22.3%	34.4%	43.3%	22.3%	34.4%	43.3%
OV-24	1298	100	1.4%	47.0%	51.6%	1.4%	44.7%	53.9%	0.0%	44.7%	55.3%
OV-29	1765	66	0.0%	78.8%	21.2%	0.0%	78.8%	21.2%	0.0%	78.8%	21.2%
OV-36	1217	na	48.2%	49.1%	2.8%	48.2%	49.1%	2.8%	48.2%	49.1%	2.8%
COA-01	654	394	0.5%	97.3%	2.3%	0.5%	97.3%	2.3%	0.5%	97.3%	2.3%
COA-04	26	351	0.0%	2.8%	97.2%	0.0%	0.9%	99.1%	0.0%	0.5%	99.5%
COA-12	-57	172	62.6%	29.7%	7.8%	80.8%	11.4%	7.8%	62.6%	29.7%	7.8%
COA-14	76	269	13.7%	0.0%	86.3%	2.7%	0.0%	97.3%	0.5%	0.0%	99.5%
COA-15	286	131	0.0%	32.4%	67.6%	0.0%	32.4%	67.6%	0.0%	32.4%	67.6%
COA-16	156	213	0.0%	98.6%	1.4%	0.0%	98.6%	1.4%	0.0%	98.6%	1.4%
COLOR-03	43	168	57.8%	1.8%	40.4%	57.8%	1.8%	40.4%	56.4%	1.8%	41.7%
COLOR-06	88	166	0.0%	0.0%	100.0%	0.0%	0.0%	100.0%	0.0%	0.0%	100.0%
COLOR-10	80	328	60.8%	16.1%	23.0%	69.6%	7.4%	23.0%	61.3%	15.7%	23.0%
COLOR-14	83	na	0.0%	86.7%	13.3%	0.0%	86.7%	13.3%	0.0%	86.7%	13.3%
COLOR-17	175	69	0.0%	96.4%	3.6%	0.0%	95.9%	4.1%	0.0%	95.9%	4.1%
ANT-07	774	173	0.0%	98.2%	1.8%	0.0%	98.2%	1.8%	0.0%	98.2%	1.8%

Status and trends of orthophosphate concentrations in groundwater used for public supply in California *Environmental Monitoring and Assessment, Robert Kent, Tyler D. Johnson, and Michael R. Rosen, U.S. Geological Survey California Water Science Center-rhkent@usgs.gov*

Online resource (suppplementary table) 3. Selected attributes of GAMA-PBP (https://ca.water.usgs.gov/gama/) trend wells evaluated for step trends in orthophosphate concentration-page 24.

GAMA-PBP ID	Agricultural land use in 2002[9] (percent)	Natural land use in 2002[9] (percent)	Urban land use in 2002[9] (percent)	Agricultural land use in 2012[9] (percent)	Natural land use in 2012[9] (percent)	Urban land use in 2012[9] (percent)	Age Classification[8]	Septic Tanks[10]	Aridity[11]
SCRV-12	0.0%	0.0%	100.0%	0.0%	0.0%	100.0%	Mixed	0.00	0.266
SCRV-16	4.5%	21.3%	74.2%	2.7%	22.2%	75.1%	ModernOrMixed	3.80	0.308
SCRV-17	0.5%	71.2%	28.3%	0.5%	70.8%	28.8%	ModernOrMixed	9.86	0.363
SCRV-18	8.2%	0.5%	91.3%	1.4%	0.9%	97.7%	PremodernOrMixed	25.92	0.308
SCRV-19	78.1%	21.5%	0.5%	78.1%	21.5%	0.5%	ModernOrMixed	1.12	0.327
SCRV-32	0.0%	0.0%	100.0%	0.0%	0.0%	100.0%	ModernOrMixed	0.11	0.280
OIW-05	0.0%	100.0%	0.0%	0.0%	100.0%	0.0%	Premodern	0.46	0.081
OIW-07	0.0%	42.0%	58.0%	0.0%	41.6%	58.4%	Premodern	11.51	0.079
OIW-12	0.0%	100.0%	0.0%	0.0%	100.0%	0.0%	Premodern	0.06	0.076
OIWU-01	0.0%	54.2%	45.8%	0.0%	54.2%	45.8%	Premodern	16.82	0.077
OV-06	0.0%	86.4%	13.6%	0.0%	86.4%	13.6%	Premodern	0.08	0.128
OV-11	13.6%	86.4%	0.0%	13.6%	86.4%	0.0%	Mixed	0.32	0.237
OV-12	0.0%	99.5%	0.5%	0.0%	99.5%	0.5%	Modern	0.35	0.205
OV-16	0.0%	100.0%	0.0%	0.0%	100.0%	0.0%	Mixed	0.19	0.112
OV-21	0.0%	89.0%	11.0%	0.0%	89.0%	11.0%	ModernOrMixed	0.26	0.125
OV-22	22.3%	34.4%	43.3%	22.3%	34.4%	43.3%	Modern	0.16	0.126
OV-24	0.0%	44.3%	55.7%	0.0%	44.3%	55.7%	Modern	13.62	0.108
OV-29	6.8%	71.6%	21.6%	6.8%	71.6%	21.6%	PremodernOrMixed	0.11	0.136
OV-36	48.2%	49.1%	2.8%	45.4%	51.8%	2.8%	Mixed	0.06	0.127
COA-01	0.5%	97.3%	2.3%	0.5%	97.3%	2.3%	Modern	13.45	0.266
COA-04	0.0%	0.5%	99.5%	0.0%	0.0%	100.0%	Premodern	108.13	0.059
COA-12	62.6%	29.7%	7.8%	62.6%	29.2%	8.2%	Premodern	1.41	0.055
COA-14	0.0%	0.0%	100.0%	0.0%	0.0%	100.0%	Modern	7.88	0.089
COA-15	0.0%	32.4%	67.6%	0.0%	32.0%	68.0%	Premodern	19.93	0.123
COA-16	0.0%	98.6%	1.4%	0.0%	98.6%	1.4%	Premodern	0.68	0.067
COLOR-03	57.8%	1.4%	40.8%	57.8%	0.9%	41.3%	Mixed	8.25	0.063
COLOR-06	0.0%	0.0%	100.0%	0.0%	0.0%	100.0%	Mixed	18.52	0.067
COLOR-10	61.3%	15.7%	23.0%	61.3%	15.7%	23.0%	Modern	0.71	0.068
COLOR-14	0.0%	86.7%	13.3%	0.0%	86.7%	13.3%	Premodern	0.05	0.057
COLOR-17	0.0%	95.9%	4.1%	0.0%	95.9%	4.1%	Premodern	0.39	0.083
ANT-07	0.0%	98.2%	1.8%	0.0%	5.5%	94.5%	Premodern	2.64	0.123

Online resource (suppplementary table) 3. Selected attributes of GAMA-PBP (https://ca.water.usgs.gov/gama/) trend wells evaluated for step trends in orthophosphate concentration-page 25.

GAMA-PBP ID	USGS Station ID[1]	GAMA-PBP project study unit	GAMA-PBP study area[3]	Hydrogeologic Zone
ANT-09	344000118250001 [2]	Antelope Valley	Antelope Valley	Desert
ANT-12	343953118041901	Antelope Valley	Antelope Valley	Desert
ANT-15	344700118360001	Antelope Valley	Antelope Valley	Desert
ANT-20	343122118024401	Antelope Valley	Antelope Valley	Desert
ANT-23	342643117505201	Antelope Valley	Antelope Valley	Desert
ANT-24	343002117393301	Antelope Valley	Antelope Valley	Desert
ANT-26	343222117552901	Antelope Valley	Antelope Valley	Desert
ANT-33	350048117442001	Antelope Valley	Antelope Valley	Desert
ANT-42	344600118160001	Antelope Valley	Antelope Valley	Desert
ANT-46	343932118144001	Antelope Valley	Antelope Valley	Desert
MOJO-01	343052117153501	Mojave	Mojave River Valleys	Desert
MOJO-09	344434117200401	Mojave	Mojave River Valleys	Desert
MOJO-11	343051117223401	Mojave	Mojave River Valleys	Desert
MOJO-16	342438117181401	Mojave	Mojave River Valleys	Desert
MOJO-18	342600117140001	Mojave	Mojave River Valleys	Desert
MOJO-34	342000117160001	Mojave	Mojave River Valleys	Desert
MOJO-42	345127116473201	Mojave	Mojave River Valleys	Desert
MOJO-48	343448117344001	Mojave	Mojave River Valleys	Desert
MOJO-49	345551116520801	Mojave	Mojave River Valleys	Desert
BV-03	332057116240101	Borrego Valley, Central Desert, and Low-Use Basins of the Mojave and Sonoran Deserts	Borrego Valley	Desert
BV-05	331333116205401	Borrego Valley, Central Desert, and Low-Use Basins of the Mojave and Sonoran Deserts	Borrego Valley	Desert
CD-02	341343116263601	Borrego Valley, Central Desert, and Low-Use Basins of the Mojave and Sonoran Deserts	Central Desert basins	Desert
CD-05	340742116053201	Borrego Valley, Central Desert, and Low-Use Basins of the Mojave and Sonoran Deserts	Central Desert basins	Desert
CD-07	340831116172201	Borrego Valley, Central Desert, and Low-Use Basins of the Mojave and Sonoran Deserts	Central Desert basins	Desert
LUB-05	342400116540001	Borrego Valley, Central Desert, and Low-Use Basins of the Mojave and Sonoran Deserts	Low-use basins of the Mojave and Sonoran Deserts	Desert
LUB-07	351125116374901	Borrego Valley, Central Desert, and Low-Use Basins of the Mojave and Sonoran Deserts	Low-use basins of the Mojave and Sonoran Deserts	Desert
LUB-11	352350117451601	Borrego Valley, Central Desert, and Low-Use Basins of the Mojave and Sonoran Deserts	Low-use basins of the Mojave and Sonoran Deserts	Desert
LUB-16	324222115130201	Borrego Valley, Central Desert, and Low-Use Basins of the Mojave and Sonoran Deserts	Low-use basins of the Mojave and Sonoran Deserts	Desert
LUB-18	323600116110001	Borrego Valley, Central Desert, and Low-Use Basins of the Mojave and Sonoran Deserts	Low-use basins of the Mojave and Sonoran Deserts	Desert
LUB-23	334712115485601	Borrego Valley, Central Desert, and Low-Use Basins of the Mojave and Sonoran Deserts	Low-use basins of the Mojave and Sonoran Deserts	Desert
CGOLD-02	371200119400001	Central Sierra	Coarse Gold watershed	Mountain

Online resource (suppplementary table) 3. Selected attributes of GAMA-PBP (https://ca.water.usgs.gov/gama/) trend wells evaluated for step trends in orthophosphate concentration-page 26.

GAMA-PBP ID	Initial Sample Date	Initital Sample Orthophos-phate Concentra-tion (mg/L as P)	Orthophos-phate reporting level for initial sample (mg/L as P)	Triennial Trend Sample Date	Triennial Sample Orthophos-phate Concentra-tion (mg/L as P)	Orthophos-phate reporting level for triennial sample (mg/L as P)	Decadal Trend Sample Date	Decadal Sample Orthophos-phate Concentra-tion (mg/L as P)	Orthophos-phate reporting level for decadal sample (mg/L as P)	Performed Evaluation 1 (comparison between initial and triennial results)	Performed Evaluation 2 (comparison between initial and decadal results)	Performed Evaluation 3 (comparison between triennial and decadal results)
ANT-09	1/30/2008	0.074	0.006	none	na	na	4/18/2018	0.144	0.004	no	yes	no
ANT-12	1/30/2008	0.011	0.006	none	na	na	4/16/2018	0.026	0.004	no	yes	no
ANT-15	1/31/2008	0.032	0.006	none	na	na	5/2/2018	0.048	0.004	no	yes	no
ANT-20	2/5/2008[4]	na	na	2/14/2012	0.087	0.004	4/30/2018	0.093	0.004	no	no	yes
ANT-23	2/6/2008[4]	na	na	2/13/2012	0.026	0.004	5/30/2018	0.028	0.004	no	no	yes
ANT-24	2/6/2008[4]	na	na	2/16/2012	0.015	0.004	3/22/2018	0.013	0.004	no	no	yes
ANT-26	2/7/2008	0.015	0.006	none	na	na	5/1/2018	0.023	0.004	no	yes	no
ANT-33	2/13/2008[4]	na	na	2/15/2012	0.034	0.004	4/17/2018	0.047	0.004	no	no	yes
ANT-42	3/3/2008[4]	na	na	2/14/2012	0.016	0.004	4/18/2018	0.025	0.004	no	no	yes
ANT-46	3/6/2008	0.017	0.006	none	na	na	5/3/2018	0.066	0.004	no	yes	no
MOJO-01	2/4/2008[4]	na	na	3/8/2011	0.013	0.004	3/6/2018	0.007	0.004	no	no	yes
MOJO-09	2/7/2008[4]	na	na	3/8/2011	0.021	0.004	3/7/2018	0.008	0.004	no	no	yes
MOJO-11	2/7/2008[4]	na	na	3/9/2011	0.012	0.004	3/7/2018	0.007	0.004	no	no	yes
MOJO-16	2/13/2008	0.008	0.006	3/10/2011	0.010	0.004	3/8/2018	0.009	0.004	yes	yes	yes
MOJO-18	2/14/2008	0.036	0.006	none	na	na	3/6/2018	0.038	0.004	no	yes[5]	no
MOJO-34	3/17/2008[4]	na	na	3/10/2011	0.045	0.004	3/5/2018	0.034	0.004	no	no	yes[5]
MOJO-42	3/26/2008	0.016	0.006	none	na	na	3/20/2018	0.019	0.004	no	yes	no
MOJO-48	4/1/2008[4]	na	na	3/9/2011	0.022	0.004	3/21/2018	0.020	0.004	no	no	yes
MOJO-49	4/1/2008	0.015	0.006	3/7/2011	0.034	0.004	3/19/2018	0.020	0.004	yes	yes	yes
BV-03	10/20/2009	0.019	0.008	none	na	na	11/8/2018	0.017	0.004	no	yes[5]	no
BV-05	10/29/2009[4]	na	na	10/18/2012	0.009	0.004	12/12/2018	0.012	0.004	no	no	yes[5]
CD-02	12/15/2008[4]	na	na	10/16/2012	0.022	0.004	10/23/2018	0.025	0.004	no	no	yes[5]
CD-05	12/17/2008[4]	na	na	10/16/2012	0.018	0.004	10/23/2018	0.022	0.004	no	no	yes[5]
CD-07	10/5/2009	0.027	0.008	none	na	na	10/24/2018	0.177	0.004	no	yes[5]	no
LUB-05	12/4/2008	0.012	0.008	10/17/2012	0.013	0.004	10/24/2018	0.018	0.004	yes	yes[5]	yes[5]
LUB-07	12/9/2008	0.011	0.008	10/17/2012	0.010	0.004	10/22/2018	0.014	0.004	yes	yes[5]	yes[5]
LUB-11	12/11/2008	0.017	0.008	10/15/2012	0.019	0.004	10/22/2018	0.023	0.004	yes	yes[5]	yes[5]
LUB-16	10/28/2009	0.009	0.008	none	na	na	11/6/2018	0.008	0.004	no	yes[5]	no
LUB-18	11/2/2009	na	na	none	na	na	11/6/2018	0.132	0.004	no	yes[5]	no
LUB-23	12/7/2009	0.010	0.008	none	na	na	11/7/2018	0.013	0.004	no	yes[5]	no
CGOLD-02	5/9/2006	0.006	0.006	none	na	na	5/17/2016	0.013	0.004	no	yes	no

Online resource (suppplementary table) 3. Selected attributes of GAMA-PBP (https://ca.water.usgs.gov/gama/) trend wells evaluated for step trends in orthophosphate concentration-page 27.

GAMA-PBP ID	Elevation of LSD (meters above NAVD 88)[6]	Well depth (meters below LSD)	Agricultural land use in 1974[9] (percent)	Natural land use in 1974[9] (percent)	Urban land use in 1974[9] (percent)	Agricultural land use in 1982[9] (percent)	Natural land use in 1982[9] (percent)	Urban land use in 1982[9] (percent)	Agricultural land use in 1992[9] (percent)	Natural land use in 1992[9] (percent)	Urban land use in 1992[9] (percent)
ANT-09	1080	70	18.8%	77.5%	3.7%	18.8%	77.5%	3.7%	17.4%	16.1%	66.5%
ANT-12	801	394	70.8%	17.4%	11.9%	66.7%	16.4%	16.9%	63.5%	16.9%	19.6%
ANT-15	977	223	34.1%	65.5%	0.5%	34.1%	65.5%	0.5%	28.2%	71.4%	0.5%
ANT-20	1004	115	0.0%	96.8%	3.2%	0.0%	96.8%	3.2%	0.0%	95.9%	4.1%
ANT-23	1225	49	17.8%	82.2%	0.0%	17.8%	82.2%	0.0%	17.8%	82.2%	0.0%
ANT-24	1111	261	0.0%	97.2%	2.8%	0.0%	95.4%	4.6%	0.0%	94.0%	6.0%
ANT-26	926	105	0.0%	97.2%	2.8%	0.0%	97.2%	2.8%	0.0%	97.2%	2.8%
ANT-33	772	174	0.0%	99.1%	0.9%	0.0%	99.1%	0.9%	0.0%	99.1%	0.9%
ANT-42	799	218	95.9%	1.8%	2.3%	95.9%	1.8%	2.3%	95.9%	1.8%	2.3%
ANT-46	799	156	72.6%	0.9%	26.5%	72.6%	0.9%	26.5%	47.0%	0.0%	53.0%
MOJO-01	915	129	0.0%	14.7%	85.3%	0.0%	14.7%	85.3%	0.0%	11.0%	89.0%
MOJO-09	798	139	0.5%	81.3%	18.2%	0.5%	79.0%	20.6%	0.5%	78.0%	21.5%
MOJO-11	1002	207	0.0%	40.6%	59.4%	0.0%	40.6%	59.4%	0.0%	40.6%	59.4%
MOJO-16	1068	328	0.0%	0.0%	100.0%	0.0%	0.0%	100.0%	0.0%	0.0%	100.0%
MOJO-18	938	151	0.0%	53.9%	46.1%	0.0%	53.9%	46.1%	0.0%	39.7%	60.3%
MOJO-34	1007	na	0.0%	100.0%	0.0%	0.0%	100.0%	0.0%	0.0%	100.0%	0.0%
MOJO-42	630	127	0.0%	29.0%	71.0%	0.0%	29.0%	71.0%	0.0%	29.0%	71.0%
MOJO-48	977	171	12.7%	86.4%	0.9%	12.7%	86.4%	0.9%	12.7%	86.4%	0.9%
MOJO-49	658	131	0.0%	99.5%	0.5%	0.0%	99.5%	0.5%	0.0%	99.5%	0.5%
BV-03	305	115	0.0%	100.0%	0.0%	0.0%	100.0%	0.0%	0.0%	100.0%	0.0%
BV-05	175	190	0.0%	96.4%	3.6%	0.0%	96.4%	3.6%	0.0%	96.4%	3.6%
CD-02	1139	117	0.0%	72.6%	27.4%	0.0%	72.6%	27.4%	0.0%	71.2%	28.8%
CD-05	709	141	0.0%	1.4%	98.6%	0.0%	1.4%	98.6%	0.0%	1.4%	98.6%
CD-07	862	243	0.0%	98.6%	1.4%	0.0%	98.6%	1.4%	0.0%	89.0%	11.0%
LUB-05	1058	161	0.0%	94.0%	6.0%	0.0%	94.0%	6.0%	0.0%	93.5%	6.5%
LUB-07	716	217	0.0%	100.0%	0.0%	0.0%	100.0%	0.0%	0.0%	100.0%	0.0%
LUB-11	751	197	0.0%	100.0%	0.0%	0.0%	100.0%	0.0%	0.0%	100.0%	0.0%
LUB-16	29	34	0.0%	100.0%	0.0%	0.0%	100.0%	0.0%	0.0%	100.0%	0.0%
LUB-18	935	12	0.0%	76.5%	23.5%	0.0%	76.5%	23.5%	0.0%	76.5%	23.5%
LUB-23	977	132	0.0%	100.0%	0.0%	0.0%	100.0%	0.0%	0.0%	100.0%	0.0%
CGOLD-02	729	246	0.0%	84.9%	15.1%	0.0%	84.9%	15.1%	0.0%	84.9%	15.1%

Online resource (suppplementary table) 3. Selected attributes of GAMA-PBP (https://ca.water.usgs.gov/gama/) trend wells evaluated for step trends in orthophosphate concentration-page 28.

GAMA-PBP ID	Agricultural land use in 2002[9] (percent)	Natural land use in 2002[9] (percent)	Urban land use in 2002[9] (percent)	Agricultural land use in 2012[9] (percent)	Natural land use in 2012[9] (percent)	Urban land use in 2012[9] (percent)	Age Classification[8]	Septic Tanks[10]	Aridity[11]
ANT-09	17.0%	16.1%	67.0%	17.0%	16.1%	67.0%	Modern	2.48	0.379
ANT-12	61.6%	16.4%	21.9%	37.0%	7.3%	55.7%	Premodern	4.24	0.113
ANT-15	28.2%	71.4%	0.5%	66.8%	32.7%	0.5%	Mixed	0.59	0.194
ANT-20	0.0%	95.0%	5.0%	0.0%	94.0%	6.0%	ModernOrMixed	6.60	0.163
ANT-23	17.8%	82.2%	0.0%	17.8%	82.2%	0.0%	ModernOrMixed	5.54	0.198
ANT-24	0.0%	91.7%	8.3%	0.0%	91.7%	8.3%	PremodernOrMixed	6.02	0.134
ANT-26	0.0%	96.3%	3.7%	0.0%	95.8%	4.2%	Premodern	7.24	0.118
ANT-33	0.0%	99.1%	0.9%	0.0%	98.6%	1.4%	PremodernOrMixed	0.49	0.089
ANT-42	95.9%	1.8%	2.3%	93.2%	1.8%	5.0%	PremodernOrMixed	4.39	0.140
ANT-46	43.4%	0.0%	56.6%	42.0%	0.0%	58.0%	Mixed	3.12	0.149
MOJO-01	0.0%	11.0%	89.0%	0.0%	1.4%	98.6%	PremodernOrMixed	57.46	0.094
MOJO-09	0.5%	77.1%	22.4%	0.5%	50.0%	49.5%	ModernOrMixed	0.38	0.077
MOJO-11	0.0%	40.6%	59.4%	0.0%	0.0%	100.0%	PremodernOrMixed	12.38	0.115
MOJO-16	0.0%	0.0%	100.0%	0.0%	0.0%	100.0%	Premodern	99.30	0.174
MOJO-18	0.0%	39.7%	60.3%	0.0%	0.0%	100.0%	Modern	20.40	0.109
MOJO-34	0.0%	100.0%	0.0%	0.0%	100.0%	0.0%	ModernOrMixed	3.06	0.187
MOJO-42	0.0%	29.0%	71.0%	0.0%	29.0%	71.0%	Premodern	3.82	0.065
MOJO-48	12.7%	86.4%	0.9%	12.7%	86.4%	0.9%	PremodernOrMixed	1.45	0.099
MOJO-49	0.0%	99.5%	0.5%	0.0%	99.5%	0.5%	PremodernOrMixed	1.88	0.072
BV-03	0.0%	100.0%	0.0%	0.0%	100.0%	0.0%	Modern	0.47	0.107
BV-05	0.0%	0.9%	99.1%	0.0%	0.9%	99.1%	Premodern	2.58	0.082
CD-02	0.0%	71.2%	28.8%	0.0%	70.8%	29.2%	Premodern	1.51	0.140
CD-05	0.0%	1.4%	98.6%	0.0%	1.4%	98.6%	Premodern	73.21	0.089
CD-07	0.0%	89.0%	11.0%	0.0%	89.0%	11.0%	Premodern	9.36	0.129
LUB-05	0.0%	93.5%	6.5%	0.0%	93.5%	6.5%	Premodern	7.55	0.120
LUB-07	0.0%	100.0%	0.0%	0.0%	100.0%	0.0%	Premodern	0.06	0.075
LUB-11	0.0%	100.0%	0.0%	0.0%	100.0%	0.0%	Premodern	0.41	0.106
LUB-16	0.0%	100.0%	0.0%	0.0%	100.0%	0.0%	Modern	0.05	0.057
LUB-18	0.0%	76.5%	23.5%	0.0%	76.5%	23.5%	Modern	4.56	0.262
LUB-23	0.0%	100.0%	0.0%	0.0%	100.0%	0.0%	Premodern	0.05	0.100
CGOLD-02	0.0%	84.9%	15.1%	0.0%	84.9%	15.1%	Mixed	4.97	0.540

Online resource (suppplementary table) 3. Selected attributes of GAMA-PBP (https://ca.water.usgs.gov/gama/) trend wells evaluated for step trends in orthophosphate concentration-page 29.

GAMA-PBP ID	USGS Station ID[1]	GAMA-PBP project study unit	GAMA-PBP study area[3]	Hydrogeologic Zone
CGOLD-07	372700119380001	Central Sierra	Coarse Gold watershed	Mountain
CGOLD-13	371200119460001	Central Sierra	Coarse Gold watershed	Mountain
CGOLD-16	372500119360001	Central Sierra	Coarse Gold watershed	Mountain
CWISH-01	371600119320001	Central Sierra	Wishon watershed	Mountain
CWISH-04	371900119340001	Central Sierra	Wishon watershed	Mountain
SOSA-06	351500118370001	Southern Sierra	Southern Sierra Nevada	Mountain
SOSA-10	354100118130001	Southern Sierra	Southern Sierra Nevada	Mountain
SOSA-15	354300118330001	Southern Sierra	Southern Sierra Nevada	Mountain
SOSA-20	353500118280001	Southern Sierra	Southern Sierra Nevada	Mountain
SOSA-22	355300118220001	Southern Sierra	Southern Sierra Nevada	Mountain
SOSA-31	353900118170001	Southern Sierra	Southern Sierra Nevada	Mountain
SOSA-32	350600118270002	Southern Sierra	Southern Sierra Nevada	Mountain
TMART-03	392200120040001	Tahoe-Martis	Martis Valley	Mountain
TMART-06	391900120100001	Tahoe-Martis	Martis Valley	Mountain
TMART-14	391700120070001	Tahoe-Martis	Martis Valley	Mountain
TROCK-02	385700120060001	Tahoe-Martis	Hard Rock Tahoe-Martis	Mountain
TROCK-05	391000120120001	Tahoe-Martis	Hard Rock Tahoe-Martis	Mountain
TROCK-06	391552120045101	Tahoe-Martis	Hard Rock Tahoe-Martis	Mountain
TTAHO-01	390600120090001	Tahoe-Martis	Tahoe Valley basins	Mountain
TTAHO-06	385500120000001	Tahoe-Martis	Tahoe Valley basins	Mountain
TTAHO-07	385200120000001	Tahoe-Martis	Tahoe Valley basins	Mountain
TTAHO-10	385238120015101	Tahoe-Martis	Tahoe Valley basins	Mountain
SIERRA-G-08	370600119190001	Sierra Nevada	Sierra Nevada Regional	Mountain
SIERRA-G-10	381900119450001	Sierra Nevada	Sierra Nevada Regional	Mountain
SIERRA-G-12	375100119560001	Sierra Nevada	Sierra Nevada Regional	Mountain
SIERRA-G-13	383900120360001	Sierra Nevada	Sierra Nevada Regional	Mountain
SIERRA-G-14	384800120050001	Sierra Nevada	Sierra Nevada Regional	Mountain
SIERRA-G-15	385300121150001	Sierra Nevada	Sierra Nevada Regional	Mountain
SIERRA-G-16	363500118140001	Sierra Nevada	Sierra Nevada Regional	Mountain
SIERRA-G-17	371100118330001	Sierra Nevada	Sierra Nevada Regional	Mountain
SIERRA-G-18	372700118440001	Sierra Nevada	Sierra Nevada Regional	Mountain

Online resource (suppplementary table) 3. Selected attributes of GAMA-PBP (https://ca.water.usgs.gov/gama/) trend wells evaluated for step trends in orthophosphate concentration-page 30.

GAMA-PBP ID	Initial Sample Date	Initital Sample Orthophos-phate Concentra-tion (mg/L as P)	Orthophos-phate reporting level for initial sample (mg/L as P)	Triennial Trend Sample Date	Triennial Sample Orthophos-phate Concentra-tion (mg/L as P)	Orthophos-phate reporting level for triennial sample (mg/L as P)	Decadal Trend Sample Date	Decadal Sample Orthophos-phate Concentra-tion (mg/L as P)	Orthophos-phate reporting level for decadal sample (mg/L as P)	Performed Evaluation 1 (comparison between initial and triennial results)	Performed Evaluation 2 (comparison between initial and decadal results)	Performed Evaluation 3 (comparison between triennial and decadal results)
CGOLD-07	5/16/2006	not detected	0.006	none	na	na	5/4/2016	0.008	0.004	no	yes	no
CGOLD-13	5/23/2006	not detected	0.006	6/14/2010	0.018	0.008	5/5/2016	0.011	0.004	yes	yes	yes
CGOLD-16	5/24/2006	0.044	0.006	5/25/2010	0.044	0.008	5/3/2016	0.049	0.004	yes	yes	yes
CWISH-01	5/8/2006	0.008	0.006	none	na	na	5/4/2016	0.021	0.004	no	yes	no
CWISH-04	5/16/2006	0.023	0.006	6/14/2010	0.041	0.008	5/3/2016	0.019	0.004	yes	yes	yes
SOSA-06	6/7/2006[4]	na	na	6/23/2008	0.014	0.006	5/16/2016	0.013	0.004	no	no	yes
SOSA-10	5/23/2006	0.054	0.006	none	na	na	5/18/2016	0.033	0.004	no	yes	no
SOSA-15	6/12/2006	0.025	0.006	7/8/2008	0.030	0.006	5/18/2016	0.014	0.004	yes[5]	yes[5]	yes[5]
SOSA-20	6/14/2006[4]	na	na	6/25/2008	0.016	0.006	5/17/2016	0.015	0.004	no	no	yes
SOSA-22	6/15/2006	0.068	0.006	none	na	na	5/19/2016	0.076	0.004	no	yes[5]	no
SOSA-31	6/29/2006[4]	na	na	6/25/2008	0.046	0.006	5/17/2016	0.046	0.004	no	no	yes
SOSA-32	6/29/2006[4]	na	na	6/24/2008	0.024	0.006	5/16/2016	0.024	0.004	no	no	yes
TMART-03	6/26/2007	0.140	0.006	8/27/2012	0.153	0.004	8/29/2017	0.177	0.004	yes	yes	yes
TMART-06	6/28/2007	0.043	0.006	8/27/2012	0.036	0.004	8/29/2017	0.032	0.004	yes	yes	yes
TMART-14	9/12/2007	0.050	0.006	none	na	na	8/30/2017	0.066	0.004	no	yes	no
TROCK-02	7/11/2007	0.006	0.006	8/28/2012	0.006	0.004	9/19/2017	not detected	0.004	yes	yes	yes
TROCK-05	7/17/2007	0.038	0.006	8/28/2012	0.037	0.004	8/31/2017	0.039	0.004	yes	yes	yes
TROCK-06	7/18/2007	0.029	0.006	none	na	na	8/30/2017	0.032	0.004	no	yes	no
TTAHO-01	7/11/2007	0.105	0.006	none	na	na	9/21/2017	0.085	0.004	no	yes	no
TTAHO-06	8/2/2007	0.016	0.006	8/29/2012	0.013	0.004	9/20/2017	0.025	0.004	yes	yes	yes
TTAHO-07	8/13/2007	0.153	0.006	none	na	na	9/20/2017	0.149	0.004	no	yes	no
TTAHO-10	8/16/2007	0.035	0.006	8/29/2012	0.032	0.004	9/20/2017	0.034	0.004	yes	yes[5]	yes[5]
SIERRA-G-08	7/23/2008	0.031	0.006	10/3/2012	0.026	0.004	9/10/2018	0.031	0.004	yes	yes	yes
SIERRA-G-10	7/28/2008	0.013	0.006	10/3/2012	0.008	0.004	8/29/2018	0.008	0.004	yes	yes	yes
SIERRA-G-12	8/14/2008	0.036	0.006	10/4/2012	0.032	0.004	8/29/2018	0.036	0.004	yes	yes	yes
SIERRA-G-13	8/21/2008	0.078	0.006	10/2/2012	0.084	0.004	8/27/2018	0.085	0.004	yes	yes	yes
SIERRA-G-14	8/26/2008	0.024	0.006	8/30/2012	0.022	0.004	8/28/2018	0.025	0.004	yes	yes	yes
SIERRA-G-15	9/8/2008	0.005	0.006	10/1/2012	0.007	0.004	8/13/2018	0.007	0.004	yes[5]	yes	yes[5]
SIERRA-G-16	9/22/2008	not detected	0.006	9/17/2012	not detected	0.004	9/12/2018	not detected	0.004	yes	yes	yes
SIERRA-G-17	9/23/2008	0.019	0.006	9/18/2012	0.020	0.004	9/12/2018	0.021	0.004	yes	yes	yes
SIERRA-G-18	9/24/2008	0.008	0.006	9/19/2012	0.007	0.004	9/11/2018	0.007	0.004	yes	yes	yes

Online resource (suppplementary table) 3. Selected attributes of GAMA-PBP (https://ca.water.usgs.gov/gama/) trend wells evaluated for step trends in orthophosphate concentration-page 31.

GAMA-PBP ID	Elevation of LSD (meters above NAVD 88)[6]	Well depth (meters below LSD)	Agricultural land use in 1974[9] (percent)	Natural land use in 1974[9] (percent)	Urban land use in 1974[9] (percent)	Agricultural land use in 1982[9] (percent)	Natural land use in 1982[9] (percent)	Urban land use in 1982[9] (percent)	Agricultural land use in 1992[9] (percent)	Natural land use in 1992[9] (percent)	Urban land use in 1992[9] (percent)
CGOLD-07	1444	152	0.0%	100.0%	0.0%	0.0%	100.0%	0.0%	0.0%	100.0%	0.0%
CGOLD-13	359	99	0.0%	97.2%	2.8%	0.0%	97.2%	2.8%	0.0%	18.1%	81.9%
CGOLD-16	1576	164	0.0%	100.0%	0.0%	0.0%	100.0%	0.0%	0.0%	100.0%	0.0%
CWISH-01	1101	199	0.0%	93.6%	6.4%	0.0%	93.6%	6.4%	0.0%	93.6%	6.4%
CWISH-04	1116	230	0.0%	91.8%	8.2%	0.0%	85.5%	14.5%	0.0%	80.5%	19.5%
SOSA-06	1066	197	0.0%	100.0%	0.0%	0.0%	100.0%	0.0%	0.0%	100.0%	0.0%
SOSA-10	893	57	0.0%	92.5%	7.5%	0.0%	92.5%	7.5%	0.0%	92.5%	7.5%
SOSA-15	1957	na	0.0%	34.1%	65.9%	0.0%	33.2%	66.8%	0.0%	33.2%	66.8%
SOSA-20	973	54	0.0%	3.7%	96.3%	0.0%	0.5%	99.5%	0.0%	0.5%	99.5%
SOSA-22	2364	na	0.0%	100.0%	0.0%	0.0%	100.0%	0.0%	0.0%	100.0%	0.0%
SOSA-31	870	39	45.5%	49.3%	5.2%	46.5%	26.8%	26.8%	45.5%	26.8%	27.7%
SOSA-32	1372	131	10.0%	75.5%	14.5%	9.5%	75.5%	15.0%	9.5%	70.5%	20.0%
TMART-03	1836	126	0.0%	95.9%	4.1%	0.0%	95.0%	5.0%	0.0%	95.0%	5.0%
TMART-06	1929	295	0.0%	29.5%	70.5%	0.0%	21.8%	78.2%	0.0%	21.8%	78.2%
TMART-14	1916	262	0.0%	97.7%	2.3%	0.0%	97.7%	2.3%	0.0%	62.5%	37.5%
TROCK-02	2160	0	0.0%	96.3%	3.7%	0.0%	96.3%	3.7%	0.0%	95.9%	4.1%
TROCK-05	2217	0	0.0%	99.1%	0.9%	0.0%	48.8%	51.2%	0.0%	48.8%	51.2%
TROCK-06	2072	72	0.0%	43.6%	56.4%	0.0%	42.7%	57.3%	0.0%	42.7%	57.3%
TTAHO-01	2045	16	0.0%	86.4%	13.6%	0.0%	86.4%	13.6%	0.0%	86.0%	14.0%
TTAHO-06	2045	162	0.0%	24.3%	75.7%	0.0%	24.3%	75.7%	0.0%	21.5%	78.5%
TTAHO-07	2070	87	0.0%	16.8%	83.2%	0.0%	16.4%	83.6%	0.0%	16.4%	83.6%
TTAHO-10	2073	82	0.0%	64.8%	35.2%	0.0%	64.3%	35.7%	0.0%	53.1%	46.9%
SIERRA-G-08	1839	39	0.0%	61.8%	38.2%	0.0%	44.5%	55.5%	0.0%	40.0%	60.0%
SIERRA-G-10	2050	34	0.0%	91.8%	8.2%	0.0%	91.8%	8.2%	0.0%	90.9%	9.1%
SIERRA-G-12	1270	221	0.0%	100.0%	0.0%	0.0%	100.0%	0.0%	0.0%	100.0%	0.0%
SIERRA-G-13	906	131	0.0%	100.0%	0.0%	0.0%	100.0%	0.0%	0.0%	100.0%	0.0%
SIERRA-G-14	2216	131	0.0%	100.0%	0.0%	0.0%	100.0%	0.0%	0.0%	100.0%	0.0%
SIERRA-G-15	66	157	8.2%	81.3%	10.5%	8.2%	77.6%	14.2%	8.2%	77.6%	14.2%
SIERRA-G-16	2820	0	0.0%	100.0%	0.0%	0.0%	100.0%	0.0%	0.0%	100.0%	0.0%
SIERRA-G-17	3215	0	0.0%	100.0%	0.0%	0.0%	100.0%	0.0%	0.0%	100.0%	0.0%
SIERRA-G-18	3317	0	0.0%	100.0%	0.0%	0.0%	100.0%	0.0%	0.0%	100.0%	0.0%

Online resource (suppplementary table) 3. Selected attributes of GAMA-PBP (https://ca.water.usgs.gov/gama/) trend wells evaluated for step trends in orthophosphate concentration-page 32.

GAMA-PBP ID	Agricultural land use in 2002[9] (percent)	Natural land use in 2002[9] (percent)	Urban land use in 2002[9] (percent)	Agricultural land use in 2012[9] (percent)	Natural land use in 2012[9] (percent)	Urban land use in 2012[9] (percent)	Age Classification[8]	Septic Tanks[10]	Aridity[11]
CGOLD-07	0.0%	100.0%	0.0%	0.0%	100.0%	0.0%	Premodern	3.99	0.924
CGOLD-13	0.0%	18.1%	81.9%	0.0%	18.1%	81.9%	Modern	9.96	0.375
CGOLD-16	0.0%	100.0%	0.0%	0.0%	100.0%	0.0%	ModernOrMixed	4.06	0.872
CWISH-01	0.0%	93.1%	6.9%	0.0%	93.1%	6.9%	Mixed	7.46	0.731
CWISH-04	0.0%	79.1%	20.9%	0.0%	79.1%	20.9%	Modern	9.28	0.757
SOSA-06	0.0%	100.0%	0.0%	0.0%	100.0%	0.0%	PremodernOrMixed	4.02	0.279
SOSA-10	0.0%	92.5%	7.5%	0.0%	92.5%	7.5%	Mixed	0.66	0.223
SOSA-15	0.0%	33.2%	66.8%	0.0%	32.7%	67.3%	Modern	1.11	0.761
SOSA-20	0.0%	0.5%	99.5%	0.0%	0.5%	99.5%	ModernOrMixed	28.68	0.236
SOSA-22	0.0%	100.0%	0.0%	0.0%	100.0%	0.0%	Modern	0.02	0.657
SOSA-31	45.5%	26.8%	27.7%	45.5%	26.8%	27.7%	ModernOrMixed	0.50	0.202
SOSA-32	9.5%	70.5%	20.0%	9.5%	59.1%	31.4%	ModernOrMixed	1.99	0.247
TMART-03	0.0%	95.0%	5.0%	0.0%	95.0%	5.0%	Premodern	1.34	0.587
TMART-06	0.0%	21.8%	78.2%	0.0%	21.8%	78.2%	Modern	64.35	0.761
TMART-14	0.0%	59.3%	40.7%	0.0%	57.4%	42.6%	Premodern	5.27	0.783
TROCK-02	0.0%	95.4%	4.6%	0.0%	95.4%	4.6%	Modern	0.00	1.128
TROCK-05	0.0%	48.8%	51.2%	0.0%	48.8%	51.2%	Modern	1.20	1.343
TROCK-06	0.0%	42.7%	57.3%	0.0%	42.7%	57.3%	Modern	0.00	0.824
TTAHO-01	0.0%	86.0%	14.0%	0.0%	86.0%	14.0%	Modern	0.00	0.815
TTAHO-06	0.0%	21.5%	78.5%	0.0%	21.5%	78.5%	Mixed	3.07	0.635
TTAHO-07	0.0%	16.4%	83.6%	0.0%	16.4%	83.6%	Modern	0.34	0.803
TTAHO-10	0.0%	53.1%	46.9%	0.0%	53.1%	46.9%	Premodern	0.00	0.891
SIERRA-G-08	0.0%	40.0%	60.0%	0.0%	40.0%	60.0%	Modern	8.39	0.784
SIERRA-G-10	0.0%	90.9%	9.1%	0.0%	90.9%	9.1%	Modern	0.03	1.731
SIERRA-G-12	0.0%	100.0%	0.0%	0.0%	100.0%	0.0%	Modern	0.21	0.765
SIERRA-G-13	0.0%	100.0%	0.0%	0.0%	100.0%	0.0%	Modern	0.88	0.996
SIERRA-G-14	0.0%	100.0%	0.0%	0.0%	100.0%	0.0%	Modern	7.41	1.286
SIERRA-G-15	8.2%	77.6%	14.2%	7.8%	33.3%	58.9%	Mixed	3.64	0.470
SIERRA-G-16	0.0%	100.0%	0.0%	0.0%	100.0%	0.0%	Mixed	0.35	0.550
SIERRA-G-17	0.0%	100.0%	0.0%	0.0%	100.0%	0.0%	Modern	0.54	0.663
SIERRA-G-18	0.0%	100.0%	0.0%	0.0%	100.0%	0.0%	Mixed	0.32	0.752

Online resource (suppplementary table) 3. Selected attributes of GAMA-PBP (https://ca.water.usgs.gov/gama/) trend wells evaluated for step trends in orthophosphate concentration-page 33.

GAMA-PBP ID	USGS Station ID[1]	GAMA-PBP project study unit	GAMA-PBP study area[3]	Hydrogeologic Zone
SIERRA-M-03	401000120560001	Sierra Nevada	Sierra Nevada Regional	Mountain
SIERRA-M-04	394100120400001	Sierra Nevada	Sierra Nevada Regional	Mountain
SIERRA-M-06	393100121110001	Sierra Nevada	Sierra Nevada Regional	Mountain
SIERRA-S-02	385131120021601	Sierra Nevada	Sierra Nevada Regional	Mountain
SIERRA-S-03	394800120300001	Sierra Nevada	Sierra Nevada Regional	Mountain
SIERRA-V-02	394800121350001	Sierra Nevada	Sierra Nevada Regional	Mountain
SIERRA-V-03	373700119050001	Sierra Nevada	Sierra Nevada Regional	Mountain
BEAR-G07	341539117130401	Bear Valley and Selected Hard Rock Areas	Hard Rock Lake Arrowhead	Mountain
BEAR-G12	341500117040001	Bear Valley and Selected Hard Rock Areas	Hard Rock Lake Arrowhead	Mountain
BEAR-S05	341400116510001	Bear Valley and Selected Hard Rock Areas	Bear Valley	Mountain
BEAR-S12	341559116495101	Bear Valley and Selected Hard Rock Areas	Bear Valley	Mountain
CAMP-ES-05	394500121360001	Cascade Range and Modoc Plateau	Eastside Sacramento Valley	Mountain
CAMP-ES-09	395200121460001	Cascade Range and Modoc Plateau	Eastside Sacramento Valley	Mountain
CAMP-HL-02	402100120380001	Cascade Range and Modoc Plateau	Honey Lake Valley	Mountain
CAMP-HL-10	400800120080002	Cascade Range and Modoc Plateau	Honey Lake Valley	Mountain
CAMP-LU-04	413200120100001	Cascade Range and Modoc Plateau	Low-use basins of the Cascade Range and Modoc Plateau	Mountain
CAMP-LU-15	401700121140001	Cascade Range and Modoc Plateau	Low-use basins of the Cascade Range and Modoc Plateau	Mountain
CAMP-QV-04	415200121220001	Cascade Range and Modoc Plateau	Quaternary volcanic areas	Mountain
CAMP-QV-12	403100121560001	Cascade Range and Modoc Plateau	Quaternary volcanic areas	Mountain
CAMP-SH-01	414100122380001	Cascade Range and Modoc Plateau	Shasta Valley and Shasta Volcanic area	Mountain
CAMP-SH-07	411800122120001	Cascade Range and Modoc Plateau	Shasta Valley and Shasta Volcanic area	Mountain
CAMP-TV-07	410800120160001	Cascade Range and Modoc Plateau	Tertiary volcanic area	Mountain
CAMP-TV-15	415500122270001	Cascade Range and Modoc Plateau	Tertiary volcanic area	Mountain
KLAM-01	415100123530001	Klamath Mountains	Klamath Mountains	Mountain
KLAM-11	412200123270001	Klamath Mountains	Klamath Mountains	Mountain
KLAM-20	405100122450001	Klamath Mountains	Klamath Mountains	Mountain
KLAM-30	410800122180001	Klamath Mountains	Klamath Mountains	Mountain
NSFVOL-14	381639122150801	North San Francisco Bay hydrologic provinces	Sonoma Volcanic Highlands	Coastal
NSFVOL-18	383038122271301	North San Francisco Bay hydrologic provinces	Sonoma Volcanic Highlands	Coastal
NSFVOL-20	381906122274901	North San Francisco Bay hydrologic provinces	Sonoma Volcanic Highlands	Coastal
NSFVP-10	383034122590701	North San Francisco Bay hydrologic provinces	North San Francisco Bay Valley and Plain	Coastal

Online resource (suppplementary table) 3. Selected attributes of GAMA-PBP (https://ca.water.usgs.gov/gama/) trend wells evaluated for step trends in orthophosphate concentration-page 34.

GAMA-PBP ID	Initial Sample Date	Initital Sample Orthophos-phate Concentra-tion (mg/L as P)	Orthophos-phate reporting level for initial sample (mg/L as P)	Triennial Trend Sample Date	Triennial Sample Orthophos-phate Concentra-tion (mg/L as P)	Orthophos-phate reporting level for triennial sample (mg/L as P)	Decadal Trend Sample Date	Decadal Sample Orthophos-phate Concentra-tion (mg/L as P)	Orthophos-phate reporting level for decadal sample (mg/L as P)	Performed Evaluation 1 (comparison between initial and triennial results)	Performed Evaluation 2 (comparison between initial and decadal results)	Performed Evaluation 3 (comparison between triennial and decadal results)
SIERRA-M-03	9/10/2008	0.010	0.006	9/11/2012	0.012	0.004	8/15/2018	0.013	0.004	yes	yes	yes
SIERRA-M-04	9/17/2008	0.005	0.006	9/12/2012	0.006	0.004	8/16/2018	not detected	0.004	yes	yes	yes
SIERRA-M-06	10/8/2008	0.006	0.008	9/13/2012	0.005	0.004	8/14/2018	0.006	0.004	yes	yes	yes
SIERRA-S-02	8/19/2008	0.031	0.006	8/30/2012	0.027	0.004	8/28/2018	0.029	0.004	yes	yes	yes
SIERRA-S-03	10/20/2008	0.049	0.008	9/12/2012	0.065	0.004	8/15/2018	0.049	0.004	yes	yes	yes
SIERRA-V-02	10/7/2008	0.079	0.008	9/10/2012	0.081	0.004	8/14/2018	0.087	0.004	yes	yes	yes
SIERRA-V-03	10/21/2008	0.090	0.008	9/20/2012	0.099	0.004	8/30/2018	0.091	0.004	yes[5]	yes[5]	yes
BEAR-G07	5/10/2010	0.016	0.008	6/5/2013	0.013	0.004	pending	na	na	yes	no	no
BEAR-G12	5/19/2010	0.072	0.008	6/5/2013	0.075	0.004	pending	na	na	yes	no	no
BEAR-S05	4/28/2010	0.013	0.008	6/6/2013	0.009	0.004	pending	na	na	yes	no	no
BEAR-S12	5/5/2010	0.060	0.008	6/6/2013	0.056	0.004	pending	na	na	yes	no	no
CAMP-ES-05	9/20/2010	0.097	0.008	8/1/2013	0.083	0.004	pending	na	na	yes	no	no
CAMP-ES-09	10/4/2010	0.116	0.004	8/1/2013	0.106	0.004	pending	na	na	yes	no	no
CAMP-HL-02	8/11/2010	0.032	0.008	8/5/2013	0.036	0.004	pending	na	na	yes	no	no
CAMP-HL-10	8/23/2010	0.086	0.008	8/5/2013	0.083	0.004	pending	na	na	yes	no	no
CAMP-LU-04	7/21/2010	0.048	0.008	8/6/2013	0.033	0.004	pending	na	na	yes	no	no
CAMP-LU-15	8/30/2010	0.032	0.008	7/30/2013	0.025	0.004	pending	na	na	yes	no	no
CAMP-QV-04	8/3/2010	0.194	0.008	8/8/2013	0.182	0.004	pending	na	na	yes	no	no
CAMP-QV-12	9/14/2010	0.050	0.008	7/31/2013	0.040	0.004	pending	na	na	yes	no	no
CAMP-SH-01	7/12/2010	0.033	0.008	7/29/2013	0.025	0.004	pending	na	na	yes	no	no
CAMP-SH-07	7/15/2010	0.037	0.008	7/30/2013	0.027	0.004	pending	na	na	yes	no	no
CAMP-TV-07	8/26/2010	0.038	0.008	8/7/2013	0.029	0.004	pending	na	na	yes	no	no
CAMP-TV-15	10/13/2010	0.011	0.004	7/29/2013	0.006	0.004	pending	na	na	yes	no	no
KLAM-01	10/18/2010	0.006	0.004	10/29/2013	not detected	0.004	pending	na	na	yes	no	no
KLAM-11	11/2/2010	0.016	0.004	10/30/2013	0.010	0.004	pending	na	na	yes	no	no
KLAM-20	11/17/2010	0.026	0.004	10/30/2013	0.018	0.004	pending	na	na	yes	no	no
KLAM-30	12/2/2010	0.025	0.004	10/31/2013	0.004	0.004	pending	na	na	yes	no	no
NSFVOL-14	10/7/2004	0.181	0.007	8/21/2007	0.164	0.006	11/19/2014	0.225	0.004	yes[5]	yes	yes[5]
NSFVOL-18	10/20/2004[4]	na	na	8/28/2007	0.207	0.006	11/5/2014	0.214	0.004	no	no	yes
NSFVOL-20	11/4/2004	0.061	0.006	none	na	na	11/20/2014	0.097	0.004	no	yes	no
NSFVP-10	9/13/2004	0.019	0.006	none	na	na	9/17/2014	0.032	0.004	no	yes[5]	no

Online resource (suppplementary table) 3. Selected attributes of GAMA-PBP (https://ca.water.usgs.gov/gama/) trend wells evaluated for step trends in orthophosphate concentration-page 35.

GAMA-PBP ID	Elevation of LSD (meters above NAVD 88)[6]	Well depth (meters below LSD)	Agricultural land use in 1974[9] (percent)	Natural land use in 1974[9] (percent)	Urban land use in 1974[9] (percent)	Agricultural land use in 1982[9] (percent)	Natural land use in 1982[9] (percent)	Urban land use in 1982[9] (percent)	Agricultural land use in 1992[9] (percent)	Natural land use in 1992[9] (percent)	Urban land use in 1992[9] (percent)
SIERRA-M-03	1281	0	0.0%	100.0%	0.0%	0.0%	100.0%	0.0%	0.0%	100.0%	0.0%
SIERRA-M-04	2108	0	0.0%	100.0%	0.0%	0.0%	100.0%	0.0%	0.0%	100.0%	0.0%
SIERRA-M-06	1103	62	0.0%	90.1%	9.9%	0.0%	90.1%	9.9%	0.0%	90.1%	9.9%
SIERRA-S-02	2068	108	0.0%	36.7%	63.3%	0.0%	34.4%	65.6%	0.0%	34.4%	65.6%
SIERRA-S-03	1590	171	1.4%	55.0%	43.6%	0.0%	55.0%	45.0%	0.0%	12.4%	87.6%
SIERRA-V-02	776	230	0.0%	8.2%	91.8%	0.0%	0.9%	99.1%	0.0%	0.0%	100.0%
SIERRA-V-03	2511	72	0.0%	99.5%	0.5%	0.0%	99.5%	0.5%	0.0%	99.5%	0.5%
BEAR-G07	1687	164	0.0%	6.4%	93.6%	0.0%	6.4%	93.6%	0.0%	6.4%	93.6%
BEAR-G12	2006	0	0.0%	100.0%	0.0%	0.0%	100.0%	0.0%	0.0%	99.5%	0.5%
BEAR-S05	2269	233	0.0%	0.0%	100.0%	0.0%	0.0%	100.0%	0.0%	0.0%	100.0%
BEAR-S12	2204	57	0.0%	21.8%	78.2%	0.0%	21.8%	78.2%	0.0%	21.8%	78.2%
CAMP-ES-05	635	180	0.0%	0.0%	100.0%	0.0%	0.0%	100.0%	0.0%	0.0%	100.0%
CAMP-ES-09	500	240	0.0%	99.1%	0.9%	0.0%	99.1%	0.9%	0.0%	99.1%	0.9%
CAMP-HL-02	1425	79	0.9%	96.3%	2.7%	0.9%	96.3%	2.7%	0.9%	94.1%	5.0%
CAMP-HL-10	1353	179	1.9%	75.5%	22.7%	1.4%	75.5%	23.1%	1.4%	75.5%	23.1%
CAMP-LU-04	1531	116	21.8%	28.6%	49.5%	21.8%	28.6%	49.5%	21.8%	28.6%	49.5%
CAMP-LU-15	1486	122	0.0%	6.4%	93.6%	0.0%	6.4%	93.6%	0.0%	6.4%	93.6%
CAMP-QV-04	1342	107	0.0%	99.1%	0.9%	4.1%	95.0%	0.9%	16.4%	82.6%	0.9%
CAMP-QV-12	849	76	0.0%	100.0%	0.0%	0.0%	100.0%	0.0%	0.0%	100.0%	0.0%
CAMP-SH-01	900	39	0.0%	31.2%	68.8%	0.0%	28.1%	71.9%	0.0%	28.1%	71.9%
CAMP-SH-07	1465	0	0.0%	100.0%	0.0%	0.0%	100.0%	0.0%	0.0%	100.0%	0.0%
CAMP-TV-07	2054	75	0.0%	100.0%	0.0%	0.0%	100.0%	0.0%	0.0%	100.0%	0.0%
CAMP-TV-15	746	90	0.0%	99.5%	0.5%	0.0%	99.5%	0.5%	0.0%	99.5%	0.5%
KLAM-01	237	0	0.0%	91.7%	8.3%	0.0%	91.2%	8.8%	0.0%	91.2%	8.8%
KLAM-11	212	29	0.0%	99.1%	0.9%	0.0%	99.1%	0.9%	0.0%	99.1%	0.9%
KLAM-20	809	0	0.0%	98.2%	1.8%	0.0%	98.2%	1.8%	0.0%	98.2%	1.8%
KLAM-30	659	39	0.0%	88.0%	12.0%	0.0%	88.0%	12.0%	0.0%	88.0%	12.0%
NSFVOL-14	46	137	9.1%	10.5%	80.4%	7.3%	10.5%	82.2%	7.8%	9.6%	82.6%
NSFVOL-18	67	220	34.1%	20.5%	45.5%	32.3%	6.8%	60.9%	36.4%	5.5%	58.2%
NSFVOL-20	99	82	0.0%	57.4%	42.6%	0.0%	56.9%	43.1%	0.0%	56.9%	43.1%
NSFVP-10	12	32	0.0%	10.1%	89.9%	0.0%	10.1%	89.9%	0.0%	10.1%	89.9%

Online resource (suppplementary table) 3. Selected attributes of GAMA-PBP (https://ca.water.usgs.gov/gama/) trend wells evaluated for step trends in orthophosphate concentration-page 36.

GAMA-PBP ID	Agricultural land use in 2002[9] (percent)	Natural land use in 2002[9] (percent)	Urban land use in 2002[9] (percent)	Agricultural land use in 2012[9] (percent)	Natural land use in 2012[9] (percent)	Urban land use in 2012[9] (percent)	Age Classification[8]	Septic Tanks[10]	Aridity[11]
SIERRA-M-03	0.0%	100.0%	0.0%	0.0%	100.0%	0.0%	ModernOrMixed	1.52	0.978
SIERRA-M-04	0.0%	100.0%	0.0%	0.0%	100.0%	0.0%	Mixed	5.42	1.598
SIERRA-M-06	0.0%	90.1%	9.9%	0.0%	89.7%	10.3%	Modern	1.95	1.563
SIERRA-S-02	0.0%	33.9%	66.1%	0.0%	33.9%	66.1%	Modern	0.00	0.886
SIERRA-S-03	0.0%	12.4%	87.6%	0.0%	12.4%	87.6%	Modern	1.55	0.507
SIERRA-V-02	0.0%	0.0%	100.0%	0.0%	0.0%	100.0%	Modern	117.37	1.343
SIERRA-V-03	0.0%	99.5%	0.5%	0.0%	99.5%	0.5%	Mixed	0.00	0.601
BEAR-G07	0.0%	6.4%	93.6%	0.0%	6.4%	93.6%	Mixed	4.55	0.637
BEAR-G12	0.0%	98.2%	1.8%	0.0%	94.5%	5.5%	ModernOrMixed	0.97	0.582
BEAR-S05	0.0%	0.0%	100.0%	0.0%	0.0%	100.0%	Premodern	0.47	0.452
BEAR-S12	0.0%	21.8%	78.2%	0.0%	19.1%	80.9%	Premodern	1.27	0.461
CAMP-ES-05	0.0%	0.0%	100.0%	0.0%	0.0%	100.0%	Modern	255.86	1.251
CAMP-ES-09	0.0%	99.1%	0.9%	0.0%	99.1%	0.9%	Mixed	1.65	0.994
CAMP-HL-02	0.9%	94.1%	5.0%	0.9%	94.1%	5.0%	Mixed	2.22	0.375
CAMP-HL-10	2.3%	74.5%	23.1%	2.3%	74.5%	23.1%	Mixed	0.00	0.217
CAMP-LU-04	21.8%	28.6%	49.5%	21.8%	28.6%	49.5%	Modern	0.43	0.324
CAMP-LU-15	0.0%	6.4%	93.6%	0.0%	6.4%	93.6%	Modern	1.95	0.797
CAMP-QV-04	16.4%	82.6%	0.9%	16.4%	82.6%	0.9%	Mixed	0.12	0.313
CAMP-QV-12	0.0%	100.0%	0.0%	0.0%	100.0%	0.0%	Modern	1.53	0.845
CAMP-SH-01	0.0%	28.1%	71.9%	0.0%	28.1%	71.9%	Mixed	6.28	0.481
CAMP-SH-07	0.0%	100.0%	0.0%	0.0%	100.0%	0.0%	Modern	1.93	1.324
CAMP-TV-07	0.0%	100.0%	0.0%	0.0%	100.0%	0.0%	Premodern	0.04	0.626
CAMP-TV-15	0.0%	99.5%	0.5%	0.0%	99.5%	0.5%	Mixed	0.53	0.434
KLAM-01	0.0%	91.2%	8.8%	0.0%	91.2%	8.8%	Modern	0.27	2.433
KLAM-11	0.0%	99.1%	0.9%	0.0%	99.1%	0.9%	Modern	0.16	1.490
KLAM-20	0.0%	98.2%	1.8%	0.0%	98.2%	1.8%	Modern	0.34	0.919
KLAM-30	0.0%	88.0%	12.0%	0.0%	88.0%	12.0%	Mixed	0.47	1.594
NSFVOL-14	6.8%	9.6%	83.6%	6.8%	9.6%	83.6%	Modern	2.58	0.611
NSFVOL-18	36.8%	5.5%	57.7%	38.2%	3.6%	58.2%	ModernOrMixed	12.26	0.729
NSFVOL-20	0.0%	56.9%	43.1%	0.0%	56.9%	43.1%	Mixed	6.42	0.672
NSFVP-10	0.0%	10.1%	89.9%	0.0%	10.1%	89.9%	Modern	13.75	1.085

Online resource (suppplementary table) 3. Selected attributes of GAMA-PBP (https://ca.water.usgs.gov/gama/) trend wells evaluated for step trends in orthophosphate concentration-page 37.

GAMA-PBP ID	USGS Station ID[1]	GAMA-PBP project study unit	GAMA-PBP study area[3]	Hydrogeologic Zone
NSFVP-19	383630122512601	North San Francisco Bay hydrologic provinces	North San Francisco Bay Valley and Plain	Coastal
NSFVP-26	383916122473501	North San Francisco Bay hydrologic provinces	North San Francisco Bay Valley and Plain	Coastal
NSFVP-29	384238122541201	North San Francisco Bay hydrologic provinces	North San Francisco Bay Valley and Plain	Coastal
NSFVP-32	382553122232501	North San Francisco Bay hydrologic provinces	North San Francisco Bay Valley and Plain	Coastal
NSFVP-34	382307122311301	North San Francisco Bay hydrologic provinces	North San Francisco Bay Valley and Plain	Coastal
NSFVP-36	381153122185701	North San Francisco Bay hydrologic provinces	North San Francisco Bay Valley and Plain	Coastal
NSFVP-37	381808122293801	North San Francisco Bay hydrologic provinces	North San Francisco Bay Valley and Plain	Coastal
NSFVP-38	381544122263801	North San Francisco Bay hydrologic provinces	North San Francisco Bay Valley and Plain	Coastal
NSFVP-39	383148122292901	North San Francisco Bay hydrologic provinces	North San Francisco Bay Valley and Plain	Coastal
NSFVP-41	381932122172601	North San Francisco Bay hydrologic provinces	North San Francisco Bay Valley and Plain	Coastal
NSFVP-45	382109122201001	North San Francisco Bay hydrologic provinces	North San Francisco Bay Valley and Plain	Coastal
NSFVP-46	382720122245701	North San Francisco Bay hydrologic provinces	North San Francisco Bay Valley and Plain	Coastal
NSFVP-48	381440122191101	North San Francisco Bay hydrologic provinces	North San Francisco Bay Valley and Plain	Coastal
NSFWG-03	382318122511401	North San Francisco Bay hydrologic provinces	Wilson Grove Formation Highlands	Coastal
NSFWGFP-01	382345122490701	North San Francisco Bay hydrologic provinces	Wilson Grove Formation Highlands	Coastal
MSMB-04	365218121490301	Monterey Bay and Salinas Valley basins	Monterey Bay area basins	Coastal
MSMB-09	365500121470001	Monterey Bay and Salinas Valley basins	Monterey Bay area basins	Coastal
MSMB-20	365425121452201	Monterey Bay and Salinas Valley basins	Monterey Bay area basins	Coastal
MSMB-28	363618121381901	Monterey Bay and Salinas Valley basins	Monterey Bay area basins	Coastal
MSMB-37	364100121360001	Monterey Bay and Salinas Valley basins	Monterey Bay area basins	Coastal
MSPR-01	353041120394501	Monterey Bay and Salinas Valley basins	Paso Robles area basin	Coastal
MSPR-03	353800120380001	Monterey Bay and Salinas Valley basins	Paso Robles area basin	Coastal
MSPR-09	354805120453601	Monterey Bay and Salinas Valley basins	Paso Robles area basin	Coastal
MSSC-04	370304122014201	Monterey Bay and Salinas Valley basins	Santa Cruz area basins	Coastal
MSSC-06	365700121580001	Monterey Bay and Salinas Valley basins	Santa Cruz area basins	Coastal
MSSC-08	370150121565301	Monterey Bay and Salinas Valley basins	Santa Cruz area basins	Coastal
MSSV-02	360000120540001	Monterey Bay and Salinas Valley basins	Salinas Valley basins	Coastal
MSSV-03	360600121000001	Monterey Bay and Salinas Valley basins	Salinas Valley basins	Coastal
MSSV-06	361100121070001	Monterey Bay and Salinas Valley basins	Salinas Valley basins	Coastal
MSSV-07	361207121075501	Monterey Bay and Salinas Valley basins	Salinas Valley basins	Coastal
MSSV-11	361900121160001	Monterey Bay and Salinas Valley basins	Salinas Valley basins	Coastal

Online resource (suppplementary table) 3. Selected attributes of GAMA-PBP (https://ca.water.usgs.gov/gama/) trend wells evaluated for step trends in orthophosphate concentration-page 38.

GAMA-PBP ID	Initial Sample Date	Initital Sample Orthophos-phate Concentra-tion (mg/L as P)	Orthophos-phate reporting level for initial sample (mg/L as P)	Triennial Trend Sample Date	Triennial Sample Orthophos-phate Concentra-tion (mg/L as P)	Orthophos-phate reporting level for triennial sample (mg/L as P)	Decadal Trend Sample Date	Decadal Sample Orthophos-phate Concentra-tion (mg/L as P)	Orthophos-phate reporting level for decadal sample (mg/L as P)	Performed Evaluation 1 (comparison between initial and triennial results)	Performed Evaluation 2 (comparison between initial and decadal results)	Performed Evaluation 3 (comparison between triennial and decadal results)
NSFVP-19	9/16/2004	0.021	0.006	none	na	na	9/16/2014	0.029	0.004	no	yes[5]	no
NSFVP-26	9/27/2004	0.090	0.006	none	na	na	9/15/2014	0.171	0.004	no	yes	no
NSFVP-29	9/28/2004	0.010	0.006	8/27/2007	0.019	0.006	9/15/2014	0.014	0.004	yes[5]	yes	yes[5]
NSFVP-32	10/7/2004[4]	na	na	8/20/2007	0.076	0.006	11/4/2014	0.055	0.004	no	no	yes[5]
NSFVP-34	10/18/2004	0.318	0.012	8/22/2007	0.338	0.006	10/20/2014	0.357	0.004	yes[5]	yes	yes[5]
NSFVP-36	10/19/2004[4]	na	na	8/20/2007	0.586	0.006	11/18/2014	0.468	0.004	no	no	yes[5]
NSFVP-37	10/19/2004	0.112	0.006	8/22/2007	0.129	0.006	10/20/2014	0.130	0.004	yes[5]	yes	yes[5]
NSFVP-38	10/20/2004	0.146	0.006	8/22/2007	0.158	0.006	10/21/2014	0.146	0.004	yes[5]	yes	yes[5]
NSFVP-39	10/21/2004[4]	na	na	11/16/2007	0.135	0.006	10/23/2014	0.130	0.004	no	no	yes
NSFVP-41	10/21/2004[4]	na	na	8/20/2007	0.221	0.006	11/6/2014	0.201	0.004	no	no	yes[5]
NSFVP-45	11/2/2004	0.023	0.006	8/21/2007	0.067	0.006	none	na	na	yes[5]	no	no
NSFVP-46	11/3/2004	0.012	0.006	none	na	na	11/4/2014	0.014	0.004	no	yes	no
NSFVP-48	11/4/2004[*]	na	na	11/15/2007	2.087	0.006	11/5/2014	2.113	0.004	no	no	yes
NSFWG-03	9/21/2004[4]	na	na	8/29/2007	0.147	0.006	9/18/2014	0.226	0.004	no	no	yes
NSFWGFP-01	10/5/2004	0.008	0.006	8/29/2007	0.019	0.006	9/17/2014	0.015	0.004	yes[5]	yes	yes[5]
MSMB-04	8/17/2005	0.011	0.006	8/20/2008	0.019	0.006	8/4/2014	0.015	0.004	yes	yes	yes
MSMB-09	8/15/2005	0.079	0.006	none	na	na	8/5/2014	0.096	0.004	no	yes	no
MSMB-20	8/16/2005	0.068	0.006	none	na	na	8/5/2014	0.082	0.004	no	yes	no
MSMB-28	8/3/2005[4]	na	na	8/21/2008	0.039	0.006	8/13/2014	0.039	0.004	no	no	yes
MSMB-37	9/1/2005	0.054	0.006	none	na	na	8/21/2014	0.066	0.004	no	yes	no
MSPR-01	7/19/2005	0.010	0.006	none	na	na	8/11/2014	0.022	0.004	no	yes	no
MSPR-03	7/28/2005[4]	na	na	11/14/2008	0.019	0.008	8/11/2014	0.016	0.004	no	no	yes
MSPR-09	7/18/2005[4]	na	na	11/14/2008	0.021	0.008	9/10/2014	0.151	0.004	no	no	yes
MSSC-04	8/25/2005	0.007	0.006	none	na	na	8/6/2014	0.025	0.004	no	yes	no
MSSC-06	8/24/2005	0.103	0.012	8/18/2008	0.131	0.006	8/4/2014	0.133	0.004	yes	yes	yes
MSSC-08	9/15/2005	0.075	0.006	none	na	na	8/6/2014	0.095	0.004	no	yes	no
MSSV-02	8/4/2005	0.051	0.006	none	na	na	8/12/2014	0.065	0.004	no	yes	no
MSSV-03	9/12/2005	0.106	0.006	none	na	na	9/10/2014	0.056	0.004	no	yes	no
MSSV-06	8/2/2005[4]	na	na	11/13/2008	0.066	0.008	8/18/2014	0.070	0.004	no	no	yes
MSSV-07	8/2/2005	0.040	0.006	none	na	na	8/18/2014	0.364	0.004	no	yes	no
MSSV-11	7/25/2005	0.013	0.006	none	na	na	9/8/2014	0.021	0.004	no	yes	no

Online resource (suppplementary table) 3. Selected attributes of GAMA-PBP (https://ca.water.usgs.gov/gama/) trend wells evaluated for step trends in orthophosphate concentration-page 39.

GAMA-PBP ID	Elevation of LSD (meters above NAVD 88)[6]	Well depth (meters below LSD)	Agricultural land use in 1974[9] (percent)	Natural land use in 1974[9] (percent)	Urban land use in 1974[9] (percent)	Agricultural land use in 1982[9] (percent)	Natural land use in 1982[9] (percent)	Urban land use in 1982[9] (percent)	Agricultural land use in 1992[9] (percent)	Natural land use in 1992[9] (percent)	Urban land use in 1992[9] (percent)
NSFVP-19	28	33	4.7%	27.4%	67.9%	0.5%	13.2%	86.3%	0.0%	8.5%	91.5%
NSFVP-26	63	197	77.2%	11.9%	11.0%	68.9%	11.0%	20.1%	58.4%	10.5%	31.1%
NSFVP-29	69	39	58.6%	6.4%	35.0%	59.1%	5.5%	35.5%	59.1%	5.5%	35.5%
NSFVP-32	42	131	71.4%	28.6%	0.0%	77.9%	22.1%	0.0%	85.0%	15.0%	0.0%
NSFVP-34	106	85	35.0%	35.5%	29.5%	35.0%	30.9%	34.1%	26.8%	36.8%	36.4%
NSFVP-36	3	100	0.0%	97.7%	2.3%	0.0%	97.7%	2.3%	0.0%	97.7%	2.3%
NSFVP-37	40	118	0.9%	0.9%	98.2%	1.8%	0.5%	97.7%	0.0%	0.5%	99.5%
NSFVP-38	11	253	30.0%	11.8%	58.2%	28.6%	11.4%	60.0%	27.3%	11.4%	61.4%
NSFVP-39	81	151	17.5%	54.3%	28.3%	17.9%	53.8%	28.3%	20.6%	51.1%	28.3%
NSFVP-41	16	77	16.4%	8.7%	74.9%	13.7%	8.7%	77.6%	15.5%	5.9%	78.5%
NSFVP-45	34	na	76.4%	10.0%	13.6%	75.5%	9.5%	15.0%	75.5%	9.1%	15.5%
NSFVP-46	50	59	81.9%	7.2%	10.9%	81.0%	7.2%	11.8%	81.4%	6.3%	12.2%
NSFVP-48	22	66	79.5%	15.0%	5.5%	79.5%	15.0%	5.5%	80.0%	14.1%	5.9%
NSFWG-03	90	181	5.9%	1.4%	92.7%	5.9%	1.4%	92.7%	5.9%	1.4%	92.7%
NSFWGFP-01	27	173	9.4%	10.3%	80.3%	9.9%	9.9%	80.3%	9.9%	9.9%	80.3%
MSMB-04	3	262	40.7%	43.1%	16.2%	39.4%	27.3%	33.3%	37.5%	27.3%	35.2%
MSMB-09	34	153	11.4%	0.0%	88.6%	3.2%	0.0%	96.8%	0.0%	0.0%	100.0%
MSMB-20	9	58	0.9%	0.0%	99.1%	0.0%	0.0%	100.0%	0.0%	0.0%	100.0%
MSMB-28	20	161	20.4%	58.4%	21.3%	17.6%	43.4%	38.9%	13.6%	43.4%	43.0%
MSMB-37	38	266	15.8%	1.4%	82.8%	14.9%	1.4%	83.7%	7.7%	0.0%	92.3%
MSPR-01	267	164	44.8%	49.3%	5.9%	45.7%	48.4%	5.9%	45.7%	26.7%	27.6%
MSPR-03	247	223	6.4%	90.4%	3.2%	27.4%	47.9%	24.7%	7.8%	63.5%	28.8%
MSPR-09	191	165	0.0%	92.3%	7.7%	0.0%	92.3%	7.7%	0.0%	92.3%	7.7%
MSSC-04	152	118	0.0%	0.0%	100.0%	0.0%	0.0%	100.0%	0.0%	0.0%	100.0%
MSSC-06	14	75	0.0%	0.0%	100.0%	0.0%	0.0%	100.0%	0.0%	0.0%	100.0%
MSSC-08	73	78	0.5%	56.2%	43.4%	0.5%	0.0%	99.5%	0.5%	0.0%	99.5%
MSSV-02	155	43	57.3%	39.1%	3.6%	57.3%	39.1%	3.6%	56.4%	40.0%	3.6%
MSSV-03	119	46	93.6%	6.4%	0.0%	93.6%	6.4%	0.0%	93.6%	6.4%	0.0%
MSSV-06	99	72	45.7%	54.3%	0.0%	45.7%	54.3%	0.0%	45.7%	54.3%	0.0%
MSSV-07	99	70	5.0%	32.7%	62.3%	5.0%	32.3%	62.7%	5.0%	29.1%	65.9%
MSSV-11	106	290	90.9%	0.5%	8.6%	90.9%	0.5%	8.6%	90.9%	0.5%	8.6%

Online resource (suppplementary table) 3. Selected attributes of GAMA-PBP (https://ca.water.usgs.gov/gama/) trend wells evaluated for step trends in orthophosphate concentration-page 40.

GAMA-PBP ID	Agricultural land use in 2002[9] (percent)	Natural land use in 2002[9] (percent)	Urban land use in 2002[9] (percent)	Agricultural land use in 2012[9] (percent)	Natural land use in 2012[9] (percent)	Urban land use in 2012[9] (percent)	Age Classification[8]	Septic Tanks[10]	Aridity[11]
NSFVP-19	0.0%	8.5%	91.5%	0.0%	8.5%	91.5%	Modern	19.62	0.853
NSFVP-26	48.9%	10.0%	41.1%	48.9%	6.8%	44.3%	Premodern	4.27	0.846
NSFVP-29	59.1%	5.5%	35.5%	59.5%	5.0%	35.5%	Modern	5.71	0.873
NSFVP-32	84.0%	15.0%	0.9%	84.0%	15.0%	0.9%	ModernOrMixed	9.62	0.699
NSFVP-34	30.0%	25.5%	44.5%	30.0%	25.5%	44.5%	Premodern	11.79	0.778
NSFVP-36	0.0%	97.7%	2.3%	0.0%	97.7%	2.3%	PremodernOrMixed	3.01	0.483
NSFVP-37	0.0%	0.5%	99.5%	0.0%	0.5%	99.5%	Modern	62.54	0.604
NSFVP-38	26.8%	11.4%	61.8%	26.8%	8.2%	65.0%	Premodern	20.98	0.564
NSFVP-39	43.0%	29.1%	27.8%	43.0%	29.1%	27.8%	ModernOrMixed	16.57	0.743
NSFVP-41	15.5%	5.9%	78.5%	15.5%	5.9%	78.5%	ModernOrMixed	13.35	0.552
NSFVP-45	75.5%	9.1%	15.5%	75.5%	9.1%	15.5%	Mixed	7.81	0.585
NSFVP-46	81.0%	6.3%	12.7%	81.0%	6.3%	12.7%	ModernOrMixed	8.74	0.703
NSFVP-48	80.5%	13.6%	5.9%	80.5%	13.6%	5.9%	PremodernOrMixed	3.33	0.499
NSFWG-03	5.9%	1.4%	92.7%	5.9%	1.4%	92.7%	PremodernOrMixed	40.86	0.897
NSFWGFP-01	9.9%	9.9%	80.3%	9.9%	9.4%	80.8%	Mixed	33.67	0.829
MSMB-04	40.3%	25.9%	33.8%	40.3%	25.9%	33.8%	Mixed	6.27	0.425
MSMB-09	0.0%	0.0%	100.0%	0.0%	0.0%	100.0%	Mixed	6.61	0.481
MSMB-20	0.0%	0.0%	100.0%	0.0%	0.0%	100.0%	Mixed	0.22	0.475
MSMB-28	11.3%	43.4%	45.2%	12.2%	41.2%	46.6%	ModernOrMixed	5.70	0.334
MSMB-37	3.6%	0.0%	96.4%	0.0%	0.0%	100.0%	Premodern	4.79	0.287
MSPR-01	45.2%	25.8%	29.0%	31.7%	24.4%	43.9%	Modern	44.32	0.347
MSPR-03	7.8%	63.0%	29.2%	7.8%	62.6%	29.7%	PremodernOrMixed	6.50	0.268
MSPR-09	0.0%	92.3%	7.7%	0.0%	92.3%	7.7%	PremodernOrMixed	0.16	0.223
MSSC-04	0.0%	0.0%	100.0%	0.0%	0.0%	100.0%	Mixed	11.09	0.837
MSSC-06	0.0%	0.0%	100.0%	0.0%	0.0%	100.0%	Mixed	6.84	0.569
MSSC-08	0.5%	0.0%	99.5%	0.5%	0.0%	99.5%	Premodern	24.92	0.678
MSSV-02	59.5%	36.4%	4.1%	59.5%	36.4%	4.1%	Modern	0.50	0.233
MSSV-03	93.6%	6.4%	0.0%	93.6%	6.4%	0.0%	ModernOrMixed	0.13	0.229
MSSV-06	47.0%	53.0%	0.0%	47.0%	53.0%	0.0%	ModernOrMixed	0.33	0.237
MSSV-07	7.3%	20.9%	71.8%	7.3%	14.5%	78.2%	Modern	0.06	0.236
MSSV-11	91.4%	0.0%	8.6%	91.4%	0.0%	8.6%	Modern	5.10	0.238

Online resource (suppplementary table) 3. Selected attributes of GAMA-PBP (https://ca.water.usgs.gov/gama/) trend wells evaluated for step trends in orthophosphate concentration-page 41.

GAMA-PBP ID	USGS Station ID[1]	GAMA-PBP project study unit	GAMA-PBP study area[3]	Hydrogeologic Zone
SF-01	374500122300001	San Francisco Bay	South San Francisco Bay basins	Coastal
SF-05	373700122240001	San Francisco Bay	South San Francisco Bay basins	Coastal
SF-08	372750122112201	San Francisco Bay	South San Francisco Bay basins	Coastal
SF-10	372347122040301	San Francisco Bay	South San Francisco Bay basins	Coastal
SF-14	372141121591101	San Francisco Bay	South San Francisco Bay basins	Coastal
SF-20	371658121573802	San Francisco Bay	South San Francisco Bay basins	Coastal
SF-21	371837121574710	San Francisco Bay	South San Francisco Bay basins	Coastal
SF-28	371801121501401	San Francisco Bay	South San Francisco Bay basins	Coastal
SF-33	371000121390001	San Francisco Bay	South San Francisco Bay basins	Coastal
SF-35	373200122010001	San Francisco Bay	South San Francisco Bay basins	Coastal
SF-38	373700122050001	San Francisco Bay	South San Francisco Bay basins	Coastal
SF-42	374504122112201	San Francisco Bay	South San Francisco Bay basins	Coastal
SCRC-B08	343600120110001	South Coast Range	South Coast Coastal alluvial basins	Coastal
SCRC-B10	344428120161201 [2]	South Coast Range	South Coast Coastal alluvial basins	Coastal
SCRC-B11	343939120265302	South Coast Range	South Coast Coastal alluvial basins	Coastal
SCRC-B16	345600120250001	South Coast Range	South Coast Coastal alluvial basins	Coastal
SCRC-B18	345545120220101	South Coast Range	South Coast Coastal alluvial basins	Coastal
SCRC-B23	350200120320001	South Coast Range	South Coast Coastal alluvial basins	Coastal
SCRC-B24	345200120290001	South Coast Range	South Coast Coastal alluvial basins	Coastal
SCRC-H08	343900120040001	South Coast Range	South Coast Coastal uplands	Coastal
SCRC-H10	344600120080001	South Coast Range	South Coast Coastal uplands	Coastal
SCRC-H11	351100120330001	South Coast Range	South Coast Coastal uplands	Coastal
SCRC-H13	344700120190001	South Coast Range	South Coast Coastal uplands	Coastal
CUY-02	345603119411901	South Coast Interior Basins	Cuyama Valley	Coastal
CUY-06	345300119310001	South Coast Interior Basins	Cuyama Valley	Coastal
CUY-10	345100119100001	South Coast Interior Basins	Cuyama Valley	Coastal
GIL-02	370000121330001	South Coast Interior Basins	Gilroy-Hollister Valley	Coastal
GIL-12	365200121330001	South Coast Interior Basins	Gilroy-Hollister Valley	Coastal
GIL-09	370300121350001	South Coast Interior Basins	Gilroy-Hollister Valley	Coastal
GIL-01	370700121370001	South Coast Interior Basins	Gilroy-Hollister Valley	Coastal
LIV-03	374000121520001	South Coast Interior Basins	Livermore Valley	Coastal

Online resource (suppplementary table) 3. Selected attributes of GAMA-PBP (https://ca.water.usgs.gov/gama/) trend wells evaluated for step trends in orthophosphate concentration-page 42.

GAMA-PBP ID	Initial Sample Date	Initital Sample Orthophos-phate Concentra-tion (mg/L as P)	Orthophos-phate reporting level for initial sample (mg/L as P)	Triennial Trend Sample Date	Triennial Sample Orthophos-phate Concentra-tion (mg/L as P)	Orthophos-phate reporting level for triennial sample (mg/L as P)	Decadal Trend Sample Date	Decadal Sample Orthophos-phate Concentra-tion (mg/L as P)	Orthophos-phate reporting level for decadal sample (mg/L as P)	Performed Evaluation 1 (comparison between initial and triennial results)	Performed Evaluation 2 (comparison between initial and decadal results)	Performed Evaluation 3 (comparison between triennial and decadal results)
SF-01	6/20/2007	0.105	0.006	none	na	na	6/8/2017	0.105	0.004	no	yes[5]	no
SF-05	5/23/2007	0.103	0.006	3/23/2011	0.098	0.004	6/6/2017	0.103	0.004	yes[5]	yes[5]	yes
SF-08	5/21/2007	0.109	0.006	none	na	na	6/7/2017	0.082	0.004	no	yes[5]	no
SF-10	5/3/2007	0.050	0.006	3/22/2011	0.049	0.004	none	na	na	yes[5]	no	no
SF-14	4/25/2007	0.052	0.006	3/21/2011	0.051	0.004	6/15/2017	0.052	0.004	yes[5]	yes[5]	yes
SF-20	5/2/2007	0.026	0.006	3/21/2011	0.025	0.004	none	na	na	yes[5]	no	no
SF-21	5/2/2007	0.025	0.006	none	na	na	6/13/2017	0.027	0.004	no	yes[5]	no
SF-28	4/23/2007	0.045	0.006	none	na	na	6/13/2017	0.045	0.004	no	yes	no
SF-33	4/30/2007	0.116	0.006	none	na	na	6/14/2017	0.071	0.004	no	yes[5]	no
SF-35	6/12/2007	0.095	0.006	none	na	na	6/14/2017	0.105	0.004	no	yes	no
SF-38	6/21/2007	0.117	0.006	none	na	na	6/7/2017	0.128	0.004	no	yes[5]	no
SF-42	6/19/2007	0.027	0.006	3/22/2011	0.044	0.004	none	na	na	yes[5]	no	no
SCRC-B08	6/4/2008	0.068	0.006	none	na	na	7/12/2018	0.044	0.004	no	yes	no
SCRC-B10	6/11/2008	0.800	0.006	none	na	na	6/27/2018	0.645	0.004	no	yes	no
SCRC-B11	6/12/2008	0.296	0.006	none	na	na	7/10/2018	0.076	0.004	no	yes	no
SCRC-B16	6/19/2008	0.019	0.006	none	na	na	7/11/2018	0.023	0.004	no	yes[5]	no
SCRC-B18	6/23/2008[4]	na	na	6/6/2012	0.036	0.004	6/26/2018	0.037	0.004	no	no	yes
SCRC-B23	7/7/2008	0.042	0.006	6/7/2012	0.030	0.004	6/25/2018	0.030	0.004	yes	yes	yes
SCRC-B24	7/7/2008[4]	na	na	6/6/2012	0.033	0.004	6/26/2018	0.035	0.004	no	no	yes
SCRC-H08	6/10/2008	0.025	0.006	6/5/2012	0.028	0.004	7/10/2018	0.036	0.004	yes	yes	yes
SCRC-H10	6/24/2008	0.021	0.006	none	na	na	6/28/2018	0.024	0.004	no	yes	no
SCRC-H11	6/26/2008	0.028	0.006	6/7/2012	0.020	0.004	6/25/2018	0.022	0.004	yes	yes	yes
SCRC-H13	6/23/2008	0.083	0.006	none	na	na	6/27/2018	0.080	0.004	no	yes	no
CUY-02	9/15/2008	0.018	0.006	9/20/2012	0.022	0.004	7/31/2018	0.021	0.004	yes	yes	yes
CUY-06	9/17/2008	0.010	0.006	9/20/2012	0.009	0.004	7/31/2018	0.017	0.004	yes	yes	yes
CUY-10	9/23/2008	0.049	0.006	none	na	na	8/1/2018	0.086	0.004	no	yes	no
GIL-02	8/12/2008	0.037	0.006	9/18/2012	0.031	0.004	7/25/2018	0.035	0.004	yes	yes	yes
GIL-12	8/19/2008	0.064	0.006	9/19/2012	0.049	0.004	7/26/2018	0.054	0.004	yes	yes	yes
GIL-09	8/18/2008	0.036	0.006	none	na	na	7/24/2018	0.037	0.004	no	yes[5]	no
GIL-01	8/11/2008	0.039	0.006	none	na	na	7/25/2018	0.025	0.004	no	yes	no
LIV-03	8/25/2008	0.031	0.006	9/18/2012	0.031	0.004	7/23/2018	0.050	0.004	yes	yes	yes

Online resource (suppplementary table) 3. Selected attributes of GAMA-PBP (https://ca.water.usgs.gov/gama/) trend wells evaluated for step trends in orthophosphate concentration-page 43.

GAMA-PBP ID	Elevation of LSD (meters above NAVD 88)[6]	Well depth (meters below LSD)	Agricultural land use in 1974[9] (percent)	Natural land use in 1974[9] (percent)	Urban land use in 1974[9] (percent)	Agricultural land use in 1982[9] (percent)	Natural land use in 1982[9] (percent)	Urban land use in 1982[9] (percent)	Agricultural land use in 1992[9] (percent)	Natural land use in 1992[9] (percent)	Urban land use in 1992[9] (percent)
SF-01	8	na	0.0%	12.2%	87.8%	0.0%	12.2%	87.8%	0.0%	12.2%	87.8%
SF-05	17	157	0.0%	0.0%	100.0%	0.0%	0.0%	100.0%	0.0%	0.0%	100.0%
SF-08	16	90	0.0%	0.0%	100.0%	0.0%	0.0%	100.0%	0.0%	0.0%	100.0%
SF-10	24	223	0.0%	0.0%	100.0%	0.0%	0.0%	100.0%	0.0%	0.0%	100.0%
SF-14	21	173	0.0%	0.0%	100.0%	0.0%	0.0%	100.0%	0.0%	0.0%	100.0%
SF-20	67	276	0.0%	0.0%	100.0%	0.0%	0.0%	100.0%	0.0%	0.0%	100.0%
SF-21	52	267	0.0%	0.0%	100.0%	0.0%	0.0%	100.0%	0.0%	0.0%	100.0%
SF-28	40	170	8.2%	0.5%	91.3%	8.2%	0.5%	91.3%	0.0%	0.5%	99.5%
SF-33	125	120	19.1%	7.7%	73.2%	15.5%	7.3%	77.3%	14.1%	6.8%	79.1%
SF-35	11	81	0.0%	0.0%	100.0%	0.0%	0.0%	100.0%	0.0%	0.0%	100.0%
SF-38	4	176	1.4%	1.4%	97.3%	0.0%	1.4%	98.6%	0.0%	1.4%	98.6%
SF-42	4	162	0.0%	0.0%	100.0%	0.0%	0.0%	100.0%	0.0%	0.0%	100.0%
SCRC-B08	116	367	7.0%	0.0%	93.0%	1.9%	0.0%	98.1%	0.0%	0.0%	100.0%
SCRC-B10	194	161	40.0%	9.5%	50.5%	33.2%	9.1%	57.7%	31.8%	8.6%	59.5%
SCRC-B11	33	22	22.7%	0.0%	77.3%	7.3%	0.0%	92.7%	3.6%	0.0%	96.4%
SCRC-B16	75	121	35.6%	0.0%	64.4%	21.5%	0.0%	78.5%	5.5%	0.0%	94.5%
SCRC-B18	99	69	98.2%	0.9%	0.9%	98.2%	0.9%	0.9%	98.2%	0.9%	0.9%
SCRC-B23	86	203	15.5%	82.6%	1.9%	20.7%	77.5%	1.9%	16.4%	80.8%	2.8%
SCRC-B24	93	197	0.0%	93.6%	6.4%	0.0%	93.6%	6.4%	0.0%	93.6%	6.4%
SCRC-H08	314	302	0.0%	97.3%	2.7%	0.0%	97.3%	2.7%	0.0%	96.8%	3.2%
SCRC-H10	434	197	1.4%	97.3%	1.4%	1.4%	97.3%	1.4%	1.4%	97.3%	1.4%
SCRC-H11	106	85	1.4%	96.8%	1.8%	21.3%	76.9%	1.8%	21.3%	54.8%	24.0%
SCRC-H13	274	322	0.0%	99.5%	0.5%	0.0%	99.5%	0.5%	0.0%	99.5%	0.5%
CUY-02	708	259	0.0%	100.0%	0.0%	0.0%	100.0%	0.0%	0.0%	100.0%	0.0%
CUY-06	837	262	90.1%	9.9%	0.0%	98.6%	1.4%	0.0%	98.6%	1.4%	0.0%
CUY-10	1772	98	3.2%	42.3%	54.5%	3.2%	32.3%	64.5%	3.2%	26.4%	70.5%
GIL-02	63	154	72.5%	0.0%	27.5%	70.2%	0.0%	29.8%	66.5%	0.0%	33.5%
GIL-12	51	230	40.6%	32.0%	27.4%	40.6%	31.5%	27.9%	40.6%	30.6%	28.8%
GIL-09	79	na	51.2%	0.9%	47.9%	47.5%	0.9%	51.6%	39.2%	0.9%	59.9%
GIL-01	116	159	35.5%	1.4%	63.1%	22.6%	0.9%	76.5%	15.7%	0.9%	83.4%
LIV-03	114	213	2.8%	0.0%	97.2%	0.5%	0.0%	99.5%	0.0%	0.0%	100.0%

Online resource (suppplementary table) 3. Selected attributes of GAMA-PBP (https://ca.water.usgs.gov/gama/) trend wells evaluated for step trends in orthophosphate concentration-page 44.

GAMA-PBP ID	Agricultural land use in 2002[9] (percent)	Natural land use in 2002[9] (percent)	Urban land use in 2002[9] (percent)	Agricultural land use in 2012[9] (percent)	Natural land use in 2012[9] (percent)	Urban land use in 2012[9] (percent)	Age Classification[8]	Septic Tanks[10]	Aridity[11]
SF-01	0.0%	12.2%	87.8%	0.0%	10.8%	89.2%	Mixed	0.00	0.481
SF-05	0.0%	0.0%	100.0%	0.0%	0.0%	100.0%	Premodern	0.00	0.496
SF-08	0.0%	0.0%	100.0%	0.0%	0.0%	100.0%	Mixed	0.00	0.367
SF-10	0.0%	0.0%	100.0%	0.0%	0.0%	100.0%	Mixed	22.44	0.310
SF-14	0.0%	0.0%	100.0%	0.0%	0.0%	100.0%	Mixed	4.76	0.321
SF-20	0.0%	0.0%	100.0%	0.0%	0.0%	100.0%	Modern	34.22	0.378
SF-21	0.0%	0.0%	100.0%	0.0%	0.0%	100.0%	Modern	3.21	0.347
SF-28	0.0%	0.5%	99.5%	0.0%	0.5%	99.5%	Modern	0.95	0.334
SF-33	11.8%	6.8%	81.4%	11.8%	5.5%	82.7%	Mixed	11.06	0.433
SF-35	0.0%	0.0%	100.0%	0.0%	0.0%	100.0%	Mixed	0.00	0.340
SF-38	0.0%	1.4%	98.6%	0.0%	1.4%	98.6%	Premodern	0.36	0.374
SF-42	0.0%	0.0%	100.0%	0.0%	0.0%	100.0%	Premodern	0.00	0.450
SCRC-B08	0.0%	0.0%	100.0%	0.0%	0.0%	100.0%	Premodern	9.90	0.319
SCRC-B10	31.4%	8.2%	60.5%	31.4%	7.3%	61.4%	Mixed	0.44	0.322
SCRC-B11	2.3%	0.0%	97.7%	2.3%	0.0%	97.7%	Modern	1.43	0.303
SCRC-B16	5.5%	0.0%	94.5%	5.5%	0.0%	94.5%	Modern	0.43	0.294
SCRC-B18	98.2%	0.0%	1.8%	98.2%	0.0%	1.8%	ModernOrMixed	4.24	0.303
SCRC-B23	16.0%	79.3%	4.7%	13.6%	80.3%	6.1%	Premodern	5.67	0.334
SCRC-B24	0.0%	93.6%	6.4%	0.0%	93.6%	6.4%	ModernOrMixed	0.63	0.294
SCRC-H08	0.0%	95.4%	4.6%	0.0%	95.4%	4.6%	Premodern	1.39	0.383
SCRC-H10	1.4%	97.3%	1.4%	1.4%	97.3%	1.4%	Premodern	0.49	0.421
SCRC-H11	21.3%	53.8%	24.9%	22.6%	53.8%	23.5%	Mixed	7.35	0.370
SCRC-H13	25.8%	73.7%	0.5%	38.7%	60.8%	0.5%	Premodern	0.63	0.353
CUY-02	0.0%	100.0%	0.0%	0.0%	100.0%	0.0%	Premodern	0.08	0.157
CUY-06	93.9%	6.1%	0.0%	93.9%	6.1%	0.0%	Mixed	0.08	0.158
CUY-10	7.7%	21.8%	70.5%	7.7%	21.4%	70.9%	ModernOrMixed	4.84	0.315
GIL-02	65.6%	0.0%	34.4%	46.8%	0.0%	53.2%	Mixed	6.84	0.413
GIL-12	38.4%	29.2%	32.4%	38.4%	27.4%	34.2%	Premodern	7.48	0.426
GIL-09	30.9%	0.9%	68.2%	30.9%	0.0%	69.1%	Modern	18.47	0.412
GIL-01	7.4%	0.5%	92.2%	7.4%	0.5%	92.2%	Mixed	17.20	0.413
LIV-03	0.0%	0.0%	100.0%	0.0%	0.0%	100.0%	Mixed	1.32	0.361

Online resource (suppplementary table) 3. Selected attributes of GAMA-PBP (https://ca.water.usgs.gov/gama/) trend wells evaluated for step trends in orthophosphate concentration-page 45.

GAMA-PBP ID	USGS Station ID[1]	GAMA-PBP project study unit	GAMA-PBP study area[3]	Hydrogeologic Zone
LIVU-03	374100121520001	South Coast Interior Basins	Livermore Valley	Coastal
NOCO-CO-02	385852123420501	Northern Coast Ranges	North Coast coastal basins	Coastal
NOCO-CO-03	404436124115701	Northern Coast Ranges	North Coast coastal basins	Coastal
NOCO-CO-08	405411124052401	Northern Coast Ranges	North Coast coastal basins	Coastal
NOCO-CO-18	403614124104201	Northern Coast Ranges	North Coast coastal basins	Coastal
NOCO-CO-23	415522124084301	Northern Coast Ranges	North Coast coastal basins	Coastal
NOCO-IN-10	390528123103802	Northern Coast Ranges	North Coast inland basins	Coastal
NOCO-IN-11	391943123070001	Northern Coast Ranges	North Coast inland basins	Coastal
NOCO-IN-27	390309122413901	Northern Coast Ranges	North Coast inland basins	Coastal
SB-07	342647119451701	Santa Barbara	Santa Barbara area basins	Coastal
SB-12	342623119373201	Santa Barbara	Santa Barbara area basins	Coastal

Online resource (suppplementary table) 3. Selected attributes of GAMA-PBP (https://ca.water.usgs.gov/gama/) trend wells evaluated for step trends in orthophosphate concentration-page 46.

GAMA-PBP ID	Initial Sample Date	Initital Sample Orthophos-phate Concentra-tion (mg/L as P)	Orthophos-phate reporting level for initial sample (mg/L as P)	Triennial Trend Sample Date	Triennial Sample Orthophos-phate Concentra-tion (mg/L as P)	Orthophos-phate reporting level for triennial sample (mg/L as P)	Decadal Trend Sample Date	Decadal Sample Orthophos-phate Concentra-tion (mg/L as P)	Orthophos-phate reporting level for decadal sample (mg/L as P)	Performed Evaluation 1 (comparison between initial and triennial results)	Performed Evaluation 2 (comparison between initial and decadal results)	Performed Evaluation 3 (comparison between triennial and decadal results)
LIVU-03	8/27/2008	0.072	0.006	none	na	na	7/24/2018	0.065	0.004	no	yes	no
NOCO-CO-02	7/30/2009	0.074	0.008	5/10/2012	0.068	0.004	pending	na	na	yes[5]	no	no
NOCO-CO-03	8/3/2009	0.056	0.008	5/9/2012	0.051	0.004	pending	na	na	yes[5]	no	no
NOCO-CO-08	8/11/2009	0.027	0.008	5/8/2012	0.028	0.004	pending	na	na	yes[5]	no	no
NOCO-CO-18	8/31/2009	0.006	0.008	5/9/2012	0.219	0.004	pending	na	na	yes[5]	no	no
NOCO-CO-23	9/15/2009	0.013	0.008	5/8/2012	0.012	0.004	pending	na	na	yes[5]	no	no
NOCO-IN-10	6/18/2009	0.012	0.008	5/2/2012	0.009	0.004	pending	na	na	yes[5]	no	no
NOCO-IN-11	6/22/2009	0.187	0.008	5/2/2012	0.095	0.004	pending	na	na	yes	no	no
NOCO-IN-27	7/27/2009	0.304	0.008	5/1/2012	0.803	0.004	pending	na	na	yes[5]	no	no
SB-07	1/26/2011	0.063	0.004	12/18/2013	0.057	0.004	pending	na	na	yes	no	no
SB-12	2/7/2011	0.025	0.004	12/18/2013	0.025	0.004	pending	na	na	yes	no	no

Online resource (suppplementary table) 3. Selected attributes of GAMA-PBP (https://ca.water.usgs.gov/gama/) trend wells evaluated for step trends in orthophosphate concentration-page 47.

GAMA-PBP ID	Elevation of LSD (meters above NAVD 88)[6]	Well depth (meters below LSD)	Agricultural land use in 1974[9] (percent)	Natural land use in 1974[9] (percent)	Urban land use in 1974[9] (percent)	Agricultural land use in 1982[9] (percent)	Natural land use in 1982[9] (percent)	Urban land use in 1982[9] (percent)	Agricultural land use in 1992[9] (percent)	Natural land use in 1992[9] (percent)	Urban land use in 1992[9] (percent)
LIVU-03	111	244	26.5%	0.0%	73.5%	5.9%	0.0%	94.1%	0.0%	0.0%	100.0%
NOCO-CO-02	16	18	0.0%	99.5%	0.5%	0.0%	99.5%	0.5%	0.0%	99.5%	0.5%
NOCO-CO-03	12	125	32.9%	2.7%	64.4%	33.3%	2.7%	63.9%	31.5%	2.7%	65.8%
NOCO-CO-08	11	71	38.2%	0.0%	61.8%	37.3%	0.0%	62.7%	35.9%	0.0%	64.1%
NOCO-CO-18	13	21	4.6%	64.4%	31.1%	4.6%	64.4%	31.1%	1.8%	67.1%	31.1%
NOCO-CO-23	13	15	54.3%	5.9%	39.7%	54.3%	5.9%	39.7%	54.3%	5.9%	39.7%
NOCO-IN-10	181	33	85.0%	12.7%	2.3%	85.0%	12.7%	2.3%	84.5%	12.7%	2.7%
NOCO-IN-11	314	33	86.7%	1.4%	11.9%	86.2%	1.4%	12.4%	86.2%	1.4%	12.4%
NOCO-IN-27	583	51	39.3%	60.7%	0.0%	39.3%	60.7%	0.0%	39.3%	60.7%	0.0%
SB-07	63	140	0.0%	0.0%	100.0%	0.0%	0.0%	100.0%	0.0%	0.0%	100.0%
SB-12	88	92	0.0%	0.0%	100.0%	0.0%	0.0%	100.0%	0.0%	0.0%	100.0%

Online resource (suppplementary table) 3. Selected attributes of GAMA-PBP (https://ca.water.usgs.gov/gama/) trend wells evaluated for step trends in orthophosphate concentration-page 48.

GAMA-PBP ID	Natural land use in 2002[9] (percent)	Urban land use in 2002[9] (percent)	Agricultural land use in 2012[9] (percent)	Natural land use in 2012[9] (percent)	Urban land use in 2012[9] (percent)	Age Classification[8]	Septic Tanks[10]	Aridity[11]
LIVU-03	0.0%	100.0%	0.0%	0.0%	100.0%	Mixed	0.70	0.370
NOCO-CO-02	99.5%	0.5%	0.0%	99.5%	0.5%	Modern	0.36	0.855
NOCO-CO-03	2.7%	65.8%	31.5%	2.7%	65.8%	Premodern	7.60	1.193
NOCO-CO-08	0.0%	65.5%	34.5%	0.0%	65.5%	Mixed	31.08	1.290
NOCO-CO-18	67.1%	31.1%	1.8%	67.1%	31.1%	Mixed	9.55	1.260
NOCO-CO-23	5.9%	39.7%	54.3%	5.9%	39.7%	Modern	24.90	1.996
NOCO-IN-10	9.5%	3.6%	86.8%	9.5%	3.6%	Modern	4.33	0.797
NOCO-IN-11	0.9%	12.4%	86.7%	0.9%	12.4%	Mixed	14.67	0.920
NOCO-IN-27	60.7%	0.0%	39.3%	60.7%	0.0%	Mixed	1.31	0.706
SB-07	0.0%	100.0%	0.0%	0.0%	100.0%	Mixed	27.96	0.339
SB-12	0.0%	100.0%	0.0%	0.0%	100.0%	Modern	10.02	0.362

Footnotes:

[1]Groundwater chemistry data are available through the USGS National Water Information System (NWIS) database at https://waterdata.usgs.gov/nwis. From there select "Water Quality", then "Field/Lab samples", and use these "Site Numbers" as "Site Identifier."

[2]Three trend samples were collected from replacement wells constructed near the original wells. Chemistry data for these wells can be retrieved by the USGS Station IDs of the replacement wells. For MADCHOW-12, the replacement well Station ID is 370800120291701; for ANT-09, the replacement well Station ID is 344000118250002; for SCRC-B10, the replacement well Station ID is 344428120161202.

[3]Descriptions and information for the 87 GAMA-PBP study areas can be found in Supporting Information table S1 of Belitz et al, 2015.

Belitz, K., Fram, M. S., & Johnson T. D. (2015). Metrics for assessing the quality of groundwater used for public supply, CA, USA: Equivalent population and area *Environmental Science and Technology*, *49* (14), 8330-8338. doi: 10.1021/acs.est.5b00265

[4]Sample collected on this date was not analyzed for orthophosphate. For this reason, the corresponding entries for orthophosphate concentration and reporting level in the following two columns are "na", nc applicable.

[5]Well not included in Principle Component Analysis (PCA) for this evaluation interval because data value for at least one parameter used in PCA was not available for the well during this interval.

[6]LSD is a datum plane that is approximately at land surface at each well. The altitude of the LSD is described in meters above the NAVD 88.

[7]Well depth given as distance from the elevation of the land-surface datum (LSD) given in the previous column. Springs are assigned a well depth of zero. Unknown well depths are assigned "na", not applicable.

[8]Land use data were represented as percentages of the broad categories, agricultural, natural, and urban in discrete years spanning five decades; 1974, 1982, 1992, 2002, and 2012 (Falcone, 2015).

Falcone, J. A. (2015). U.S. conterminous wall-to-wall anthropogenic land use trends (NWALT), 1974–2012: U.S. Geological Survey Data Series 948, 33 p. plus appendixes 3–6 as separate files, http://dx.doi.org/10.3133/ds948.

[9]Age classifications for groundwater in each well are based principally on activities of tritium (Plummer et al., 1993) and carbon-14 (Clark and Fritz, 1997). The age classifications are presented as categories: modern, modern or mixed, premodern or mixed, and premodern. For principal component analyses these 5 categories were assigned values 1 through 5 in the order listed above representing a youngest-to-oldest gradient scale.

Plummer, L. N., Michel, R. L., Thurman, E. M., & Glynn, P. D. (1993). Environmental tracers for age-dating young groundwater, in Alley, W. M., eds. Regional Groundwater Quality (pp. 25 294). New York: Van Nostrand Reinhold.

Clark, I. D., & Fritz, P. (1997). Environmental Isotopes in Hydrogeology. Boca Raton & New York: Lewis Publishers.

[10]Septic tank density was determined from the 1990 Census of Population and Housing (U.S. Department of Commerce, 1992), and expressed as tanks/km2

U.S. Department of Commerce. (1992), 1990 Census of population and housing, summary tape file 3A: U. S. Census Gureau, CD-ROM, http://www.census.gov/mp/www/cat/decennial_census_1990/1990_census_of_population_and_housing_summary_tape_file_3a.html.

[11]Aridity index is calculated as the average annual precipitation (PRISM Climate Group, 2012) divided by the average annual evapotranspiration (Flint and Flint, 2007), and values can range from 0.05 (hyper-arid) to greater than 1.00 (wet).

PRISM Climate Group, (2012). United States average annual precipitation, maximum and minimum temperature, 1971-2000: Oregon State University, PRISM website, accessed November 14, 2018 at http://prism.oregonstate.edu/
