# Supplementary material for: Status and trends of orthophosphate concentrations in groundwater used for public supply in California
Source: Environ Monit Assess. 2020 Jul 29;192(8):550. doi: 10.1007/s10661-020-08504-x (PMC7391407; doi:10.1007/s10661-020-08504-x)
Supplement: Supplementary file 4 — (PDF 286 kb) [file 10661_2020_8504_MOESM4_ESM.pdf]

Status and trends of orthophosphate concentrations in groundwater used for public supply in California *Environmental Monitoring and Assessment, Robert Kent, Tyler D. Johnson, and Michael R. Rosen, U.S. Geological Survey California Water Science Center-rhkent@usgs.gov*

Online resource (suppplementary table) 4. Selected attributes of wells evaluated for time-series trends in orthophosphate concentrations-page 1.

USGS Station ID[1]	GAMA-PBP ID	Well location relative to GAMA-PBP project study unit	Hydrogeologic zone	Kendall's tau τ (correlation coefficient)[3]	p-value[4] (values ≤ 0.05 are bolded)	Sen slope estimate (rate of change in mg/L/yr as P)[5]	Trend test outcome[4]
344910119270501		South Coast Interior Basins	Coastal	0.473	**0.039**	2.34E-03	Increase
345603119411901	CUY-02	South Coast Interior Basins	Coastal	0.357	0.266	5.88E-04	no trend
343828120293201		South Coast Range	Coastal	-0.111	0.755	-1.46E-03	no trend
343833120030901	SCRC-B09	South Coast Range	Coastal	0.343	0.071	7.73E-04	no trend
343914120270301		South Coast Range	Coastal	0.595	**0.001**	9.27E-03	Increase
343920120262001		South Coast Range	Coastal	0.352	0.075	7.16E-03	no trend
343926120201001		South Coast Range	Coastal	0.181	0.373	1.41E-03	no trend
343926120293001		South Coast Range	Coastal	0.085	0.649	1.60E-03	no trend
343938120264002		South Coast Range	Coastal	0.162	0.387	2.26E-03	no trend
343948120300301	SCRC-U02	South Coast Range	Coastal	-0.137	0.449	-4.45E-03	no trend
343948120320901		South Coast Range	Coastal	-0.447	**0.019**	-1.17E-02	decrease
343948120321301	SCRC-B21	South Coast Range	Coastal	0.279	0.128	5.98E-03	no trend
344008120320901		South Coast Range	Coastal	0.191	0.303	2.50E-03	no trend
344009120320403		South Coast Range	Coastal	-0.176	0.325	-2.95E-03	no trend
344041120341101		South Coast Range	Coastal	-0.074	0.711	-2.50E-03	no trend
344043120322407		South Coast Range	Coastal	0.516	**0.003**	6.06E-03	Increase
344045120324601		South Coast Range	Coastal	0.310	0.295	4.32E-03	no trend
344110120351201		South Coast Range	Coastal	-0.037	0.938	-3.87E-03	no trend
344112120351001		South Coast Range	Coastal	0.013	0.970	2.51E-03	no trend
344442120173201	SCRC-B07	South Coast Range	Coastal	-0.143	0.488	-2.61E-03	no trend
345325120184201	SCRC-B19	South Coast Range	Coastal	-0.017	0.964	-6.32E-05	no trend
345340120261801		South Coast Range	Coastal	0.450	**0.013**	7.19E-04	Increase
345441120291301		South Coast Range	Coastal	0.428	**0.022**	1.00E-03	Increase
345459120232301		South Coast Range	Coastal	0.213	0.297	3.71E-04	no trend
345535120204401		South Coast Range	Coastal	0.200	0.300	9.65E-04	no trend
345552120220001		South Coast Range	Coastal	0.000	1.000	1.32E-05	no trend
345650120255901		South Coast Range	Coastal	-0.644	**0.012**	-9.80E-04	decrease
345712120321701	SCRC-B22	South Coast Range	Coastal	0.426	**0.019**	9.65E-04	Increase
345808120271401	SCRC-B25	South Coast Range	Coastal	0.221	0.232	5.56E-04	no trend
345823120383901		South Coast Range	Coastal	0.522	**0.003**	1.14E-03	Increase
345823120383903		South Coast Range	Coastal	0.553	**0.002**	1.02E-03	Increase

Online resource (suppplementary table) 4. Selected attributes of wells evaluated for time-series trends in orthophosphate concentrations-page 2.

USGS Station ID[1]	First sample date of time series	Last sample date of time series	Time series length (years)	Well depth (meters below LSD)[6]	Agricultural land use in 1974[9] (percent)	Natural land use in 1974[9] (percent)	Urban land use in 1974[9] (percent)	Agricultural land use in 1982[9] (percent)	Natural land use in 1982[9] (percent)	Urban land use in 1982[9] (percent)
344910119270501	7/18/2000	7/12/2011	11.0	71	73.2%	26.8%	0.0%	73.2%	26.8%	0.0%
345603119411901	9/15/2008	7/31/2018	9.9	241	0.0%	100.0%	0.0%	0.0%	100.0%	0.0%
343828120293201	11/21/2001	8/13/2009	7.7	27	92.7%	4.1%	3.2%	91.8%	4.1%	4.1%
343833120030901	7/17/2000	7/16/2018	18.0	106	92.3%	2.7%	5.0%	92.3%	0.5%	7.3%
343914120270301	8/16/2000	8/10/2017	17.0	52	5.0%	0.0%	95.0%	0.9%	0.0%	99.1%
343920120262001	8/14/2000	8/14/2014	14.0	38	6.8%	24.2%	68.9%	6.8%	24.2%	68.9%
343926120201001	7/29/2003	7/16/2018	15.0	224	97.2%	2.8%	0.0%	97.2%	2.8%	0.0%
343926120293001	8/16/2000	8/8/2017	17.0	49	93.9%	3.8%	2.3%	93.0%	2.3%	4.7%
343938120264002	8/16/2000	8/10/2017	17.0	53	8.8%	7.9%	83.3%	7.9%	7.9%	84.3%
343948120300301	8/18/2000	8/7/2017	17.0	53	97.2%	0.5%	2.3%	97.2%	0.5%	2.3%
343948120320901	8/20/2002	8/9/2017	15.0	58	95.9%	2.3%	1.8%	96.3%	1.8%	1.8%
343948120321301	11/21/2001	8/7/2017	15.7	58	95.5%	2.3%	2.3%	95.9%	1.8%	2.3%
344008120320901	8/18/2000	8/9/2017	17.0	58	98.6%	0.9%	0.5%	98.6%	0.9%	0.5%
344009120320403	8/17/2000	8/7/2017	17.0	25	98.6%	0.9%	0.5%	98.6%	0.9%	0.5%
344041120341101	8/16/2000	8/7/2017	17.0	55	0.5%	99.1%	0.5%	0.5%	99.1%	0.5%
344043120322407	8/18/2000	8/8/2017	17.0	53	74.4%	25.1%	0.5%	74.4%	25.1%	0.5%
344045120324601	8/23/2001	8/10/2016	15.0	22	67.3%	32.3%	0.5%	67.3%	32.3%	0.5%
344110120351201	8/15/2000	8/25/2010	10.0	27	0.0%	96.2%	3.8%	0.0%	95.3%	4.7%
344112120351001	8/15/2000	8/7/2017	17.0	38	0.0%	95.9%	4.1%	0.0%	94.5%	5.5%
344442120173201	7/15/2004	7/17/2018	14.0	152	95.9%	1.8%	2.3%	95.9%	1.8%	2.3%
345325120184201	7/25/2001	7/12/2017	16.0	156	91.2%	3.7%	5.1%	91.2%	3.7%	5.1%
345340120261801	9/20/2002	7/16/2018	15.8	101	34.6%	0.0%	65.4%	32.2%	0.0%	67.8%
345441120291301	7/20/2000	7/10/2017	17.0	34	90.0%	9.1%	0.9%	90.0%	9.1%	0.9%
345459120232301	10/4/2002	7/11/2016	13.8	202	70.6%	17.8%	11.7%	70.6%	17.8%	11.7%
345535120204401	7/17/2003	7/16/2018	15.0	78	82.2%	16.9%	0.9%	87.2%	11.9%	0.9%
345552120220001	7/19/2000	7/16/2018	18.0	53	97.7%	0.9%	1.4%	97.7%	0.9%	1.4%
345650120255901	7/10/2008	7/18/2018	10.0	81	0.0%	0.0%	100.0%	0.0%	0.0%	100.0%
345712120321701	7/23/2001	7/10/2017	16.0	107	100.0%	0.0%	0.0%	100.0%	0.0%	0.0%
345808120271401	9/27/2002	7/17/2018	15.8	122	44.2%	0.5%	55.3%	43.8%	0.5%	55.8%
345823120383901	12/13/2000	11/15/2017	16.9	205	0.0%	97.7%	2.3%	0.0%	97.7%	2.3%
345823120383903	12/13/2000	11/15/2017	16.9	135	0.0%	97.7%	2.3%	0.0%	97.7%	2.3%

Online resource (supplementary table) 4. Selected attributes of wells evaluated for time-series trends in orthophosphate concentrations-page 3.

USGS Station ID[1]	Agricultural land use in 1992[9] (percent)	Natural land use in 1992[9] (percent)	Urban land use in 1992[9] (percent)	Agricultural land use in 2002[9] (percent)	Natural land use in 2002[9] (percent)	Urban land use in 2002[9] (percent)	Agricultural land use in 2012[9] (percent)	Natural land use in 2012[9] (percent)	Urban land use in 2012[9] (percent)	Septic Tanks[8]	Aridity[9]
344910119270501	73.2%	26.8%	0.0%	75.0%	25.0%	0.0%	75.0%	25.0%	0.0%	0.08	0.171
345603119411901	0.0%	100.0%	0.0%	0.0%	100.0%	0.0%	0.0%	100.0%	0.0%	0.08	0.157
343828120293201	90.4%	4.1%	5.5%	90.4%	0.5%	9.1%	90.4%	0.5%	9.1%	0.57	0.302
343833120030901	91.8%	0.5%	7.7%	94.1%	0.0%	5.9%	94.1%	0.0%	5.9%	15.31	0.369
343914120270301	0.5%	0.0%	99.5%	0.0%	0.0%	100.0%	0.0%	0.0%	100.0%	0.00	0.303
343920120262001	6.8%	24.2%	68.9%	6.8%	24.2%	68.9%	6.8%	21.0%	72.1%	2.32	0.304
343926120201001	97.2%	2.8%	0.0%	97.7%	2.3%	0.0%	97.7%	2.3%	0.0%	1.13	0.338
343926120293001	91.5%	1.4%	7.0%	88.7%	0.0%	11.3%	88.7%	0.0%	11.3%	0.57	0.290
343938120264002	6.5%	7.9%	85.6%	6.5%	7.9%	85.6%	6.5%	7.9%	85.6%	2.29	0.303
343948120300301	96.3%	0.5%	3.3%	96.7%	0.0%	3.3%	96.7%	0.0%	3.3%	0.57	0.289
343948120320901	94.5%	1.8%	3.7%	94.5%	0.5%	5.1%	94.5%	0.5%	5.1%	0.57	0.281
343948120321301	94.1%	1.8%	4.1%	94.5%	0.5%	5.0%	94.5%	0.5%	5.0%	0.57	0.283
344008120320901	98.6%	0.9%	0.5%	96.8%	0.5%	2.8%	96.8%	0.0%	3.2%	0.57	0.281
344009120320403	98.6%	0.9%	0.5%	96.7%	0.5%	2.8%	96.7%	0.0%	3.3%	0.57	0.283
344041120341101	0.5%	99.1%	0.5%	19.2%	78.9%	1.9%	19.2%	78.9%	1.9%	0.09	0.304
344043120322407	74.4%	24.7%	0.9%	78.1%	20.1%	1.8%	78.1%	20.1%	1.8%	0.41	0.290
344045120324601	67.3%	32.3%	0.5%	72.7%	25.0%	2.3%	72.7%	25.0%	2.3%	0.30	0.285
344110120351201	0.0%	92.0%	8.0%	0.0%	88.7%	11.3%	0.0%	88.7%	11.3%	0.09	0.278
344112120351001	0.0%	90.3%	9.7%	0.0%	86.6%	13.4%	0.0%	86.6%	13.4%	0.09	0.278
344442120173201	95.9%	1.8%	2.3%	96.3%	0.9%	2.7%	96.3%	0.9%	2.7%	0.93	0.343
345325120184201	91.2%	3.7%	5.1%	93.1%	1.8%	5.1%	93.1%	1.8%	5.1%	0.73	0.313
345340120261801	31.3%	0.0%	68.7%	31.3%	0.0%	68.7%	31.3%	0.0%	68.7%	0.49	0.288
345441120291301	90.0%	9.1%	0.9%	97.3%	0.9%	1.8%	97.3%	0.9%	1.8%	0.90	0.282
345459120232301	70.6%	17.8%	11.7%	71.5%	16.8%	11.7%	73.4%	15.0%	11.7%	4.24	0.301
345535120204401	87.2%	11.9%	0.9%	89.0%	9.6%	1.4%	89.0%	9.6%	1.4%	4.24	0.307
345552120220001	97.7%	0.9%	1.4%	97.3%	0.0%	2.7%	97.3%	0.0%	2.7%	4.24	0.303
345650120255901	0.0%	0.0%	100.0%	0.0%	0.0%	100.0%	0.0%	0.0%	100.0%	0.00	0.295
345712120321701	100.0%	0.0%	0.0%	100.0%	0.0%	0.0%	100.0%	0.0%	0.0%	0.61	0.303
345808120271401	43.8%	0.5%	55.8%	43.8%	0.5%	55.8%	43.8%	0.0%	56.2%	1.48	0.295
345823120383901	0.0%	97.7%	2.3%	0.0%	97.7%	2.3%	0.0%	97.3%	2.7%	0.55	0.322
345823120383903	0.0%	97.7%	2.3%	0.0%	97.7%	2.3%	0.0%	97.3%	2.7%	0.55	0.322

Online resource (suppplementary table) 4. Selected attributes of wells evaluated for time-series trends in orthophosphate concentrations-page 4.

USGS Station ID[1]	GAMA-PBP ID	Well location relative to GAMA-PBP project study unit	Hydrogeologic zone	Kendall's tau τ (correlation coefficient)[3]	p-value[4] (values ≤ 0.05 are bolded)	Sen slope estimate (rate of change in mg/L/yr as P)[5]	Trend test outcome[4]
345823120383904		South Coast Range	Coastal	0.375	**0.034**	9.63E-04	Increase
345921120381601	SCRC-B39	South Coast Range	Coastal	0.607	**0.001**	7.01E-04	Increase
345921120381602		South Coast Range	Coastal	0.743	**<0.001**	1.18E-03	Increase
345921120381603		South Coast Range	Coastal	0.551	**0.002**	7.70E-04	Increase
345921120381604		South Coast Range	Coastal	0.428	**0.015**	7.47E-04	Increase
342452119405504		Santa Barbara area basins	Coastal	0.520	**0.002**	4.22E-03	Increase
342452119405505		Santa Barbara area basins	Coastal	-0.392	**0.021**	-1.60E-03	decrease
342455119405602		Santa Barbara area basins	Coastal	0.645	**<0.001**	1.57E-03	Increase
342455119405603		Santa Barbara area basins	Coastal	0.446	**0.009**	9.20E-04	Increase
342455119405604		Santa Barbara area basins	Coastal	0.576	**0.011**	2.78E-03	Increase
342455119412401		Santa Barbara area basins	Coastal	0.235	0.172	7.77E-04	no trend
342456119425201		Santa Barbara area basins	Coastal	0.275	0.189	7.47E-03	no trend
342501119410501		Santa Barbara area basins	Coastal	0.618	**<0.001**	1.41E-03	Increase
342506119412201		Santa Barbara area basins	Coastal	0.340	**0.046**	8.37E-04	Increase
342506119412203		Santa Barbara area basins	Coastal	0.602	**<0.001**	2.79E-03	Increase
342508119413702	SBU-02	Santa Barbara area basins	Coastal	0.359	**0.041**	6.41E-04	Increase
342508119413703	SBU-03	Santa Barbara area basins	Coastal	-0.509	**0.003**	-3.32E-03	decrease
342508119413704	SBU-04	Santa Barbara area basins	Coastal	0.216	0.208	3.27E-04	no trend
342508119413705	SBU-05	Santa Barbara area basins	Coastal	0.123	0.484	2.16E-04	no trend
342630119442301		Santa Barbara area basins	Coastal	0.386	**0.028**	8.11E-04	Increase
342630119442302		Santa Barbara area basins	Coastal	0.411	**0.016**	1.24E-03	Increase
372205120381701		Central Eastside San Joaquin Basin	Central Valley	0.500	0.076	1.10E-03	no trend
372746120443601		Central Eastside San Joaquin Basin	Central Valley	0.429	0.174	1.54E-03	no trend
373239120473001		Central Eastside San Joaquin Basin	Central Valley	0.389	0.175	1.93E-03	no trend
374148120581601		Central Eastside San Joaquin Basin	Central Valley	0.500	0.108	2.00E-03	no trend
360302119202101	S4-TUSK-TLE06[2]	Southeast San Joaquin Valley	Central Valley	0.500	0.108	7.14E-04	no trend
363107119372201		Southeast San Joaquin Valley	Central Valley	0.556	**0.048**	2.02E-03	Increase
363418119384201		Southeast San Joaquin Valley	Central Valley	0.389	0.175	8.48E-04	no trend
363645119420901		Southeast San Joaquin Valley	Central Valley	0.618	**0.046**	1.57E-03	Increase
363806119345301		Southeast San Joaquin Valley	Central Valley	-0.278	0.348	-4.46E-04	no trend
363928119401701		Southeast San Joaquin Valley	Central Valley	-0.833	**0.002**	-3.36E-03	decrease

Online resource (suppplementary table) 4. Selected attributes of wells evaluated for time-series trends in orthophosphate concentrations-page 5.

USGS Station ID[1]	First sample date of time series	Last sample date of time series	Time series length (years)	Well depth (meters below LSD)[6]	Agricultural land use in 1974[9] (percent)	Natural land use in 1974[9] (percent)	Urban land use in 1974[9] (percent)	Agricultural land use in 1982[9] (percent)	Natural land use in 1982[9] (percent)	Urban land use in 1982[9] (percent)
345823120383904	12/13/2000	11/15/2017	16.9	115	0.0%	97.7%	2.3%	0.0%	97.7%	2.3%
345921120381601	11/29/2000	11/14/2017	17.0	187	0.0%	100.0%	0.0%	0.0%	100.0%	0.0%
345921120381602	11/28/2000	11/14/2017	17.0	151	0.0%	100.0%	0.0%	0.0%	100.0%	0.0%
345921120381603	11/28/2000	11/14/2017	17.0	69	0.0%	100.0%	0.0%	0.0%	100.0%	0.0%
345921120381604	11/28/2000	11/14/2017	17.0	41	0.0%	100.0%	0.0%	0.0%	100.0%	0.0%
342452119405504	6/15/2000	6/14/2018	18.0	131	0.0%	37.3%	62.7%	0.0%	37.3%	62.7%
342452119405505	6/15/2000	6/14/2018	18.0	59	0.0%	37.3%	62.7%	0.0%	37.3%	62.7%
342455119405602	6/15/2000	6/12/2018	18.0	116	0.0%	26.8%	73.2%	0.0%	26.8%	73.2%
342455119405603	6/15/2000	6/12/2018	18.0	168	0.0%	25.7%	74.3%	0.0%	25.7%	74.3%
342455119405604	6/22/2000	6/12/2018	18.0	244	0.0%	25.7%	74.3%	0.0%	25.7%	74.3%
342455119412401	6/21/2000	6/13/2018	18.0	61	0.0%	0.0%	100.0%	0.0%	0.0%	100.0%
342456119425201	6/14/2000	6/20/2013	13.0	46	0.0%	0.0%	100.0%	0.0%	0.0%	100.0%
342501119410501	6/21/2000	6/14/2018	18.0	232	0.0%	3.2%	96.8%	0.0%	3.2%	96.8%
342506119412201	6/21/2000	7/18/2018	18.1	85	0.0%	0.0%	100.0%	0.0%	0.0%	100.0%
342506119412203	6/21/2000	6/12/2018	18.0	226	0.0%	0.0%	100.0%	0.0%	0.0%	100.0%
342508119413702	6/13/2000	6/11/2018	18.0	238	0.0%	0.0%	100.0%	0.0%	0.0%	100.0%
342508119413703	6/13/2000	6/11/2018	18.0	204	0.0%	0.0%	100.0%	0.0%	0.0%	100.0%
342508119413704	6/13/2000	6/11/2018	18.0	134	0.0%	0.0%	100.0%	0.0%	0.0%	100.0%
342508119413705	6/13/2000	6/11/2018	18.0	67	0.0%	0.0%	100.0%	0.0%	0.0%	100.0%
342630119442301	6/20/2000	6/13/2018	18.0	207	0.0%	0.0%	100.0%	0.0%	0.0%	100.0%
342630119442302	6/20/2000	6/13/2018	18.0	85	0.0%	0.0%	100.0%	0.0%	0.0%	100.0%
372205120381701	10/5/2001	7/17/2014	12.8	70	98.1%	0.0%	1.9%	98.1%	0.0%	1.9%
372746120443601	10/4/2001	7/16/2014	12.8	29	98.6%	0.0%	1.4%	98.6%	0.0%	1.4%
373239120473001	10/3/2001	7/15/2014	12.8	59	95.4%	0.9%	3.7%	95.4%	0.9%	3.7%
374148120581601	8/20/2002	8/4/2015	13.0	34	18.3%	0.0%	81.7%	5.5%	0.0%	94.5%
360302119202101	8/14/2002	3/23/2015	12.6	94	99.5%	0.0%	0.5%	99.5%	0.0%	0.5%
363107119372201	7/18/2000	7/17/2013	13.0	39	95.4%	0.9%	3.7%	96.3%	0.0%	3.7%
363418119384201	7/18/2000	7/17/2013	13.0	37	97.3%	1.4%	1.4%	97.7%	0.9%	1.4%
363645119420901	7/19/2000	7/18/2013	13.0	37	96.8%	2.3%	0.9%	97.2%	1.8%	0.9%
363806119345301	7/17/2000	7/22/2013	13.0	35	98.2%	1.8%	0.0%	100.0%	0.0%	0.0%
363928119401701	7/19/2000	7/23/2013	13.0	46	97.7%	1.4%	0.9%	98.2%	0.9%	0.9%

Online resource (suppplementary table) 4. Selected attributes of wells evaluated for time-series trends in orthophosphate concentrations-page 6.

USGS Station ID[1]	Agricultural land use in 1992[9] (percent)	Natural land use in 1992[9] (percent)	Urban land use in 1992[9] (percent)	Agricultural land use in 2002[9] (percent)	Natural land use in 2002[9] (percent)	Urban land use in 2002[9] (percent)	Agricultural land use in 2012[9] (percent)	Natural land use in 2012[9] (percent)	Urban land use in 2012[9] (percent)	Septic Tanks[8]	Aridity[9]
345823120383904	0.0%	97.7%	2.3%	0.0%	97.7%	2.3%	0.0%	97.3%	2.7%	0.55	0.322
345921120381601	0.0%	100.0%	0.0%	0.0%	100.0%	0.0%	0.0%	100.0%	0.0%	0.84	0.323
345921120381602	0.0%	100.0%	0.0%	0.0%	100.0%	0.0%	0.0%	100.0%	0.0%	0.81	0.323
345921120381603	0.0%	100.0%	0.0%	0.0%	100.0%	0.0%	0.0%	100.0%	0.0%	0.81	0.323
345921120381604	0.0%	100.0%	0.0%	0.0%	100.0%	0.0%	0.0%	100.0%	0.0%	0.81	0.323
342452119405504	0.0%	37.3%	62.7%	0.0%	37.3%	62.7%	0.0%	37.3%	62.7%	2.41	0.333
342452119405505	0.0%	37.3%	62.7%	0.0%	37.3%	62.7%	0.0%	37.3%	62.7%	2.41	0.333
342455119405602	0.0%	26.8%	73.2%	0.0%	26.8%	73.2%	0.0%	26.8%	73.2%	2.53	0.333
342455119405603	0.0%	25.7%	74.3%	0.0%	25.7%	74.3%	0.0%	25.7%	74.3%	2.53	0.333
342455119405604	0.0%	25.7%	74.3%	0.0%	25.7%	74.3%	0.0%	25.7%	74.3%	2.53	0.333
342455119412401	0.0%	0.0%	100.0%	0.0%	0.0%	100.0%	0.0%	0.0%	100.0%	0.35	0.333
342456119425201	0.0%	0.0%	100.0%	0.0%	0.0%	100.0%	0.0%	0.0%	100.0%	3.09	0.348
342501119410501	0.0%	3.2%	96.8%	0.0%	3.2%	96.8%	0.0%	3.2%	96.8%	1.95	0.333
342506119412201	0.0%	0.0%	100.0%	0.0%	0.0%	100.0%	0.0%	0.0%	100.0%	0.84	0.333
342506119412203	0.0%	0.0%	100.0%	0.0%	0.0%	100.0%	0.0%	0.0%	100.0%	0.84	0.333
342508119413702	0.0%	0.0%	100.0%	0.0%	0.0%	100.0%	0.0%	0.0%	100.0%	0.08	0.333
342508119413703	0.0%	0.0%	100.0%	0.0%	0.0%	100.0%	0.0%	0.0%	100.0%	0.08	0.333
342508119413704	0.0%	0.0%	100.0%	0.0%	0.0%	100.0%	0.0%	0.0%	100.0%	0.08	0.333
342508119413705	0.0%	0.0%	100.0%	0.0%	0.0%	100.0%	0.0%	0.0%	100.0%	0.08	0.333
342630119442301	0.0%	0.0%	100.0%	0.0%	0.0%	100.0%	0.0%	0.0%	100.0%	12.75	0.335
342630119442302	0.0%	0.0%	100.0%	0.0%	0.0%	100.0%	0.0%	0.0%	100.0%	12.75	0.335
372205120381701	98.1%	0.0%	1.9%	97.7%	0.0%	2.3%	97.7%	0.0%	2.3%	10.59	0.232
372746120443601	98.6%	0.0%	1.4%	98.2%	0.0%	1.8%	98.2%	0.0%	1.8%	5.91	0.250
373239120473001	95.4%	0.9%	3.7%	95.4%	0.9%	3.7%	95.4%	0.5%	4.1%	8.92	0.263
374148120581601	0.0%	0.0%	100.0%	0.0%	0.0%	100.0%	0.0%	0.0%	100.0%	3.07	0.259
360302119202101	99.5%	0.0%	0.5%	99.1%	0.0%	0.9%	99.1%	0.0%	0.9%	0.90	0.150
363107119372201	96.3%	0.0%	3.7%	95.9%	0.0%	4.1%	95.9%	0.0%	4.1%	5.72	0.185
363418119384201	97.3%	0.9%	1.8%	98.2%	0.0%	1.8%	98.2%	0.0%	1.8%	11.56	0.200
363645119420901	97.2%	1.8%	0.9%	98.2%	0.9%	0.9%	98.2%	0.0%	1.8%	10.06	0.202
363806119345301	100.0%	0.0%	0.0%	100.0%	0.0%	0.0%	100.0%	0.0%	0.0%	5.54	0.215
363928119401701	98.2%	0.9%	0.9%	99.1%	0.0%	0.9%	99.1%	0.0%	0.9%	6.60	0.210

Online resource (suppplementary table) 4. Selected attributes of wells evaluated for time-series trends in orthophosphate concentrations-page 7.

USGS Station ID[1]	GAMA-PBP ID	Well location relative to GAMA-PBP project study unit	Hydrogeologic zone	Kendall's tau τ (correlation coefficient)[3]	p-value[4] (values ≤ 0.05 are bolded)	Sen slope estimate (rate of change in mg/L/yr as P)[5]	Trend test outcome[4]
374448121130701		Northern San Joaquin Basin	Central Valley	0.327	0.319	1.24E-03	no trend
384145121184101		Southern Sacramento Valley	Central Valley	0.764	**0.013**	2.70E-03	Increase
324117115552001		Borrego Valley, Central Desert, and Low-Use Basins of the Mojave and Sonoran Deserts	Desert	0.512	**0.004**	4.78E-04	Increase
324323115580001		Borrego Valley, Central Desert, and Low-Use Basins of the Mojave and Sonoran Deserts	Desert	0.512	**0.003**	9.33E-04	Increase
324407115590901		Borrego Valley, Central Desert, and Low-Use Basins of the Mojave and Sonoran Deserts	Desert	0.652	**0.003**	1.13E-03	Increase
324415116000501		Borrego Valley, Central Desert, and Low-Use Basins of the Mojave and Sonoran Deserts	Desert	0.350	0.142	9.87E-04	no trend
324416115594101		Borrego Valley, Central Desert, and Low-Use Basins of the Mojave and Sonoran Deserts	Desert	0.512	**0.004**	7.66E-04	Increase
324424116012301		Borrego Valley, Central Desert, and Low-Use Basins of the Mojave and Sonoran Deserts	Desert	0.497	**0.003**	7.82E-04	Increase
324428115581601		Borrego Valley, Central Desert, and Low-Use Basins of the Mojave and Sonoran Deserts	Desert	0.574	**0.001**	8.03E-04	Increase
324439115593401		Borrego Valley, Central Desert, and Low-Use Basins of the Mojave and Sonoran Deserts	Desert	0.532	**0.002**	5.11E-04	Increase
324558115595201		Borrego Valley, Central Desert, and Low-Use Basins of the Mojave and Sonoran Deserts	Desert	0.331	0.059	3.79E-04	no trend
324608115593501		Borrego Valley, Central Desert, and Low-Use Basins of the Mojave and Sonoran Deserts	Desert	0.418	**0.015**	5.52E-04	Increase
340718116263701		Borrego Valley, Central Desert, and Low-Use Basins of the Mojave and Sonoran Deserts	Desert	0.927	**<0.001**	3.33E-03	Increase
340724116264801		Borrego Valley, Central Desert, and Low-Use Basins of the Mojave and Sonoran Deserts	Desert	0.214	0.536	1.16E-03	no trend
340727116263801		Borrego Valley, Central Desert, and Low-Use Basins of the Mojave and Sonoran Deserts	Desert	-0.167	0.602	-4.19E-04	no trend
340729116264702		Borrego Valley, Central Desert, and Low-Use Basins of the Mojave and Sonoran Deserts	Desert	-0.929	**0.002**	-1.52E-03	decrease
340734116264301		Borrego Valley, Central Desert, and Low-Use Basins of the Mojave and Sonoran Deserts	Desert	0.200	0.436	1.19E-03	no trend
340736116244601		Borrego Valley, Central Desert, and Low-Use Basins of the Mojave and Sonoran Deserts	Desert	-0.236	0.350	-4.11E-04	no trend
340737116250801		Borrego Valley, Central Desert, and Low-Use Basins of the Mojave and Sonoran Deserts	Desert	-0.422	0.107	-1.58E-03	no trend
340737116250802		Borrego Valley, Central Desert, and Low-Use Basins of the Mojave and Sonoran Deserts	Desert	-0.467	0.074	-1.20E-02	no trend
340737116250803		Borrego Valley, Central Desert, and Low-Use Basins of the Mojave and Sonoran Deserts	Desert	-0.422	0.107	-6.82E-04	no trend
340746116244201		Borrego Valley, Central Desert, and Low-Use Basins of the Mojave and Sonoran Deserts	Desert	-0.689	**0.007**	-2.00E-03	decrease
340746116244202		Borrego Valley, Central Desert, and Low-Use Basins of the Mojave and Sonoran Deserts	Desert	-0.600	**0.020**	-1.35E-03	decrease
340746116244203		Borrego Valley, Central Desert, and Low-Use Basins of the Mojave and Sonoran Deserts	Desert	-0.111	0.721	-3.19E-04	no trend
340746116244204		Borrego Valley, Central Desert, and Low-Use Basins of the Mojave and Sonoran Deserts	Desert	-0.600	**0.020**	-2.73E-03	decrease
341556116233401		Borrego Valley, Central Desert, and Low-Use Basins of the Mojave and Sonoran Deserts	Desert	0.231	0.300	3.23E-04	no trend
342518116505401		Borrego Valley, Central Desert, and Low-Use Basins of the Mojave and Sonoran Deserts	Desert	-0.500	0.076	-1.96E-03	no trend
345703116350801	LUB-10	Borrego Valley, Central Desert, and Low-Use Basins of the Mojave and Sonoran Deserts	Desert	0.495	**0.016**	1.81E-03	Increase
352350117451601	LUB-11	Borrego Valley, Central Desert, and Low-Use Basins of the Mojave and Sonoran Deserts	Desert	0.444	0.118	7.49E-04	no trend
342610116564102		Borrego Valley, Central Desert, and Low-Use Basins of the Mojave and Sonoran Deserts	Desert	0.242	0.304	3.50E-04	no trend
350053117115301		Borrego Valley, Central Desert, and Low-Use Basins of the Mojave and Sonoran Deserts	Desert	0.156	0.592	1.10E-04	no trend

Online resource (suppplementary table) 4. Selected attributes of wells evaluated for time-series trends in orthophosphate concentrations-page 8.

USGS Station ID[1]	First sample date of time series	Last sample date of time series	Time series length (years)	Well depth (meters below LSD)[6]	Agricultural land use in 1974[9] (percent)	Natural land use in 1974[9] (percent)	Urban land use in 1974[9] (percent)	Agricultural land use in 1982[9] (percent)	Natural land use in 1982[9] (percent)	Urban land use in 1982[9] (percent)
374448121130701	10/16/2001	7/7/2014	12.7	53	98.6%	0.0%	1.4%	98.6%	0.0%	1.4%
384145121184101	6/13/2002	9/4/2012	10.2	60	12.1%	0.0%	87.9%	6.5%	0.0%	93.5%
324117115552001	3/28/2000	3/27/2018	18.0	106	0.0%	95.4%	4.6%	0.0%	94.4%	5.6%
324323115580001	3/29/2000	3/27/2018	18.0	34	0.0%	55.8%	44.2%	0.0%	55.3%	44.7%
324407115590901	5/30/2000	4/2/2014	13.8	125	0.0%	82.1%	17.9%	0.0%	81.7%	18.3%
324415116000501	3/28/2000	4/25/2011	11.1	91	0.0%	93.1%	6.9%	0.0%	92.7%	7.3%
324416115594101	3/30/2000	3/28/2018	18.0	92	0.0%	54.0%	46.0%	0.0%	53.5%	46.5%
324424116012301	3/28/2000	3/26/2018	18.0	125	0.0%	100.0%	0.0%	0.0%	100.0%	0.0%
324428115581601	3/29/2000	3/27/2018	18.0	23	0.0%	86.3%	13.7%	0.0%	86.3%	13.7%
324439115593401	3/29/2000	3/26/2018	18.0	113	0.0%	89.1%	10.9%	0.0%	89.1%	10.9%
324558115595201	3/27/2000	3/26/2018	18.0	45	0.0%	100.0%	0.0%	0.0%	100.0%	0.0%
324608115593501	3/27/2000	3/26/2018	18.0	39	0.0%	100.0%	0.0%	0.0%	100.0%	0.0%
340718116263701	6/15/2005	5/11/2018	12.9	106	0.0%	18.3%	81.7%	0.0%	18.3%	81.7%
340724116264801	4/11/2007	5/7/2018	11.1	231	0.0%	22.1%	77.9%	0.0%	22.1%	77.9%
340727116263801	8/27/2001	5/7/2018	16.7	302	0.0%	31.1%	68.9%	0.0%	31.1%	68.9%
340729116264702	7/10/2001	8/20/2014	13.1	195	0.0%	32.7%	67.3%	0.0%	32.7%	67.3%
340734116264301	6/16/2005	5/8/2018	12.9	122	0.0%	48.6%	51.4%	0.0%	48.6%	51.4%
340736116244601	5/9/2006	5/18/2017	11.0	244	0.0%	18.2%	81.8%	0.0%	15.5%	84.5%
340737116250801	8/29/2001	5/10/2018	16.7	174	0.0%	26.8%	73.2%	0.0%	26.8%	73.2%
340737116250802	8/29/2001	5/10/2018	16.7	119	0.0%	26.8%	73.2%	0.0%	26.8%	73.2%
340737116250803	8/29/2001	5/10/2018	16.7	91	0.0%	26.8%	73.2%	0.0%	26.8%	73.2%
340746116244201	8/28/2001	5/9/2018	16.7	174	0.0%	28.4%	71.6%	0.0%	26.0%	74.0%
340746116244202	8/27/2001	5/9/2018	16.7	122	0.0%	28.4%	71.6%	0.0%	26.0%	74.0%
340746116244203	8/28/2001	5/9/2018	16.7	93	0.0%	28.4%	71.6%	0.0%	26.0%	74.0%
340746116244204	8/28/2001	5/9/2018	16.7	70	0.0%	28.4%	71.6%	0.0%	26.0%	74.0%
341556116233401	4/20/2001	5/19/2017	16.1	94	0.0%	81.3%	18.7%	0.0%	81.3%	18.7%
342518116505401	7/1/2009	5/21/2018	8.9	196	0.0%	100.0%	0.0%	0.0%	100.0%	0.0%
345703116350801	4/18/2001	5/8/2017	16.1	91	0.0%	100.0%	0.0%	0.0%	100.0%	0.0%
352350117451601	7/10/2001	10/23/2018	17.3	183	0.0%	100.0%	0.0%	0.0%	100.0%	0.0%
342610116564102	4/19/2001	5/17/2017	16.1	61	0.0%	90.7%	9.3%	0.0%	90.7%	9.3%
350053117115301	10/23/2006	5/12/2017	10.6	61	0.0%	100.0%	0.0%	0.0%	100.0%	0.0%

Online resource (suppplementary table) 4. Selected attributes of wells evaluated for time-series trends in orthophosphate concentrations-page 9.

USGS Station ID[1]	Agricultural land use in 1992[9] (percent)	Natural land use in 1992[9] (percent)	Urban land use in 1992[9] (percent)	Agricultural land use in 2002[9] (percent)	Natural land use in 2002[9] (percent)	Urban land use in 2002[9] (percent)	Agricultural land use in 2012[9] (percent)	Natural land use in 2012[9] (percent)	Urban land use in 2012[9] (percent)	Septic Tanks[8]	Aridity[9]
374448121130701	98.6%	0.0%	1.4%	98.6%	0.0%	1.4%	98.6%	0.0%	1.4%	8.80	0.255
384145121184101	0.0%	0.0%	100.0%	0.0%	0.0%	100.0%	0.0%	0.0%	100.0%	5.68	0.467
324117115552001	0.0%	94.4%	5.6%	0.0%	94.4%	5.6%	0.0%	94.4%	5.6%	0.12	0.059
324323115580001	0.0%	52.5%	47.5%	0.0%	39.6%	60.4%	0.0%	23.5%	76.5%	0.12	0.056
324407115590901	0.0%	81.7%	18.3%	0.0%	81.2%	18.8%	0.0%	81.2%	18.8%	8.76	0.061
324415116000501	0.0%	92.7%	7.3%	0.0%	91.7%	8.3%	0.0%	91.7%	8.3%	8.76	0.062
324416115594101	0.0%	53.5%	46.5%	0.0%	53.1%	46.9%	0.0%	52.6%	47.4%	8.76	0.062
324424116012301	0.0%	100.0%	0.0%	0.0%	100.0%	0.0%	0.0%	100.0%	0.0%	8.76	0.067
324428115581601	0.0%	86.3%	13.7%	0.0%	85.8%	14.2%	0.0%	85.8%	14.2%	8.75	0.056
324439115593401	0.0%	89.1%	10.9%	0.0%	89.1%	10.9%	0.0%	89.1%	10.9%	8.76	0.060
324558115595201	0.0%	100.0%	0.0%	0.0%	100.0%	0.0%	0.0%	100.0%	0.0%	0.01	0.061
324608115593501	0.0%	100.0%	0.0%	0.0%	100.0%	0.0%	0.0%	100.0%	0.0%	0.01	0.060
340718116263701	0.0%	18.3%	81.7%	0.0%	18.3%	81.7%	0.0%	6.6%	93.4%	124.26	0.162
340724116264801	0.0%	22.1%	77.9%	0.0%	22.1%	77.9%	0.0%	12.9%	87.1%	144.51	0.165
340727116263801	0.0%	29.2%	70.8%	0.0%	29.2%	70.8%	0.0%	15.1%	84.9%	137.93	0.165
340729116264702	0.0%	29.5%	70.5%	0.0%	29.5%	70.5%	0.0%	16.8%	83.2%	131.82	0.165
340734116264301	0.0%	28.2%	71.8%	0.0%	28.2%	71.8%	0.0%	15.0%	85.0%	103.16	0.162
340736116244601	0.0%	5.9%	94.1%	0.0%	5.9%	94.1%	0.0%	2.3%	97.7%	86.05	0.156
340737116250801	0.0%	3.2%	96.8%	0.0%	3.2%	96.8%	0.0%	2.7%	97.3%	98.67	0.156
340737116250802	0.0%	3.2%	96.8%	0.0%	3.2%	96.8%	0.0%	2.7%	97.3%	98.67	0.156
340737116250803	0.0%	3.2%	96.8%	0.0%	3.2%	96.8%	0.0%	2.7%	97.3%	98.67	0.156
340746116244201	0.0%	1.9%	98.1%	0.0%	1.9%	98.1%	0.0%	0.9%	99.1%	47.49	0.152
340746116244202	0.0%	1.9%	98.1%	0.0%	1.9%	98.1%	0.0%	0.9%	99.1%	47.49	0.152
340746116244203	0.0%	1.9%	98.1%	0.0%	1.9%	98.1%	0.0%	0.9%	99.1%	47.49	0.152
340746116244204	0.0%	1.9%	98.1%	0.0%	1.9%	98.1%	0.0%	0.9%	99.1%	47.49	0.152
341556116233401	0.0%	14.6%	85.4%	0.0%	14.6%	85.4%	0.0%	14.6%	85.4%	2.53	0.118
342518116505401	0.0%	100.0%	0.0%	0.0%	100.0%	0.0%	0.0%	100.0%	0.0%	1.45	0.102
345703116350801	0.0%	100.0%	0.0%	0.0%	100.0%	0.0%	0.0%	100.0%	0.0%	0.30	0.064
352350117451601	0.0%	100.0%	0.0%	0.0%	100.0%	0.0%	0.0%	100.0%	0.0%	0.41	0.106
342610116564102	0.0%	90.7%	9.3%	0.0%	90.7%	9.3%	0.0%	90.3%	9.7%	7.55	0.102
350053117115301	0.0%	100.0%	0.0%	0.0%	100.0%	0.0%	8.1%	91.9%	0.0%	0.04	0.083

Online resource (suppplementary table) 4. Selected attributes of wells evaluated for time-series trends in orthophosphate concentrations-page 10.

USGS Station ID[1]	GAMA-PBP ID	Well location relative to GAMA-PBP project study unit	Hydrogeologic zone	Kendall's tau τ (correlation coefficient)[3]	p-value[4] (values ≤ 0.05 are bolded)	Sen slope estimate (rate of change in mg/L/yr as P)[5]	Trend test outcome[4]
335231116345401		Coachella Valley	Desert	0.420	**0.018**	6.55E-04	Increase
335304116353001		Coachella Valley	Desert	0.363	0.072	5.06E-04	no trend
335318116363301		Coachella Valley	Desert	0.525	**0.003**	6.87E-04	Increase
335339116345301		Coachella Valley	Desert	0.364	**0.040**	4.76E-04	Increase
335339116345302		Coachella Valley	Desert	0.426	**0.017**	5.06E-04	Increase
335339116345303		Coachella Valley	Desert	-0.029	0.902	-4.12E-05	no trend
335348116352701		Coachella Valley	Desert	0.300	0.094	3.94E-04	no trend
335348116352702		Coachella Valley	Desert	0.159	0.417	1.49E-04	no trend
335348116352703		Coachella Valley	Desert	0.459	**0.009**	5.11E-04	Increase
335532116471701	COA-01	Coachella Valley	Desert	0.000	1.000	4.54E-05	no trend
342300117160301	MOJO-05	Mojave Valley	Desert	0.271	0.222	2.71E-04	no trend
342301117205001		Mojave Valley	Desert	0.067	0.858	4.76E-05	no trend
342450117151201		Mojave Valley	Desert	0.152	0.537	5.93E-05	no trend
342639117194001		Mojave Valley	Desert	0.295	0.213	4.90E-04	no trend
342726117082401		Mojave Valley	Desert	-0.200	0.474	-4.32E-04	no trend
342743117211701		Mojave Valley	Desert	0.260	0.271	1.31E-04	no trend
342918117153201		Mojave Valley	Desert	0.506	0.057	7.92E-04	no trend
342931117201501		Mojave Valley	Desert	0.406	0.138	5.62E-04	no trend
342953117173101		Mojave Valley	Desert	0.321	0.169	1.19E-04	no trend
343030117300901	MOJOU-05	Mojave Valley	Desert	0.212	0.373	6.47E-04	no trend
343030117300902		Mojave Valley	Desert	-0.382	0.120	-7.64E-04	no trend
343038117341701	MOJO-08	Mojave Valley	Desert	0.379	0.085	4.68E-04	no trend
343341117101601		Mojave Valley	Desert	0.294	0.242	6.16E-04	no trend
344927116394101		Mojave Valley	Desert	0.390	**0.048**	9.88E-04	Increase
345153117080701		Mojave Valley	Desert	0.045	0.928	3.57E-05	no trend
345249116483401		Mojave Valley	Desert	0.673	**0.005**	1.30E-03	Increase
345330116425501		Mojave Valley	Desert	0.026	0.951	1.49E-05	no trend
345446116485101		Mojave Valley	Desert	0.473	0.135	4.40E-04	no trend
345600116523901		Mojave Valley	Desert	0.590	**0.006**	2.41E-03	Increase
344230117200501		Mojave Valley	Desert	0.360	0.178	6.17E-04	no trend
373818118513301		Owens and Indian Wells Valleys	Desert	0.657	**<0.001**	1.91E-03	Increase

Online resource (suppplementary table) 4. Selected attributes of wells evaluated for time-series trends in orthophosphate concentrations-page 11.

USGS Station ID[1]	First sample date of time series	Last sample date of time series	Time series length (years)	Well depth (meters below LSD)[6]	Agricultural land use in 1974[9] (percent)	Natural land use in 1974[9] (percent)	Urban land use in 1974[9] (percent)	Agricultural land use in 1982[9] (percent)	Natural land use in 1982[9] (percent)	Urban land use in 1982[9] (percent)
335231116345401	11/14/2000	3/19/2018	17.4	151	0.0%	77.1%	22.9%	0.0%	76.1%	23.9%
335304116353001	11/14/2000	3/20/2018	17.4	175	0.0%	77.1%	22.9%	0.0%	77.1%	22.9%
335318116363301	11/13/2000	3/20/2018	17.4	221	0.0%	14.3%	85.7%	0.0%	14.3%	85.7%
335339116345301	11/15/2000	3/20/2018	17.4	172	0.0%	100.0%	0.0%	0.0%	100.0%	0.0%
335339116345302	11/15/2000	3/20/2018	17.4	211	0.0%	100.0%	0.0%	0.0%	100.0%	0.0%
335339116345303	11/15/2000	3/20/2018	17.4	264	0.0%	100.0%	0.0%	0.0%	100.0%	0.0%
335348116352701	11/16/2000	3/21/2018	17.4	188	0.0%	100.0%	0.0%	0.0%	100.0%	0.0%
335348116352702	11/16/2000	3/21/2018	17.4	263	0.0%	100.0%	0.0%	0.0%	100.0%	0.0%
335348116352703	11/16/2000	3/21/2018	17.4	344	0.0%	100.0%	0.0%	0.0%	100.0%	0.0%
335532116471701	9/10/2003	2/21/2017	13.5	366	0.5%	97.3%	2.3%	0.5%	97.3%	2.3%
342300117160301	4/15/2001	5/16/2017	16.1	213	0.0%	75.7%	24.3%	0.0%	46.3%	53.7%
342301117205001	4/17/2001	5/25/2017	16.1	309	0.0%	1.8%	98.2%	0.0%	1.8%	98.2%
342450117151201	4/19/2001	5/11/2017	16.1	184	0.0%	0.0%	100.0%	0.0%	0.0%	100.0%
342639117194001	4/20/2000	5/15/2017	17.1	274	0.0%	0.0%	100.0%	0.0%	0.0%	100.0%
342726117082401	5/21/2008	5/19/2017	9.0	182	0.0%	97.7%	2.3%	0.0%	97.7%	2.3%
342743117211701	4/11/2000	5/17/2016	16.1	246	0.0%	64.1%	35.9%	0.0%	63.6%	36.4%
342918117153201	4/18/2001	5/16/2017	16.1	122	0.0%	0.9%	99.1%	0.0%	0.9%	99.1%
342931117201501	4/20/2000	5/11/2017	17.1	198	0.0%	0.0%	100.0%	0.0%	0.0%	100.0%
342953117173101	4/20/2000	5/11/2017	17.1	158	0.0%	0.0%	100.0%	0.0%	0.0%	100.0%
343030117300901	6/4/2002	5/23/2018	16.0	228	0.0%	93.6%	6.4%	0.0%	92.7%	7.3%
343030117300902	6/4/2002	5/23/2018	16.0	172	0.0%	93.6%	6.4%	0.0%	92.7%	7.3%
343038117341701	4/18/2001	3/21/2018	16.9	201	0.0%	91.8%	8.2%	0.0%	87.2%	12.8%
343341117101601	5/15/2003	5/16/2016	13.0	93	0.0%	0.0%	100.0%	0.0%	0.0%	100.0%
344927116394101	4/13/2000	2/27/2018	17.9	66	0.0%	83.3%	16.7%	0.0%	83.3%	16.7%
345153117080701	5/12/2004	5/12/2015	11.0	65	0.0%	94.5%	5.5%	0.0%	94.1%	5.9%
345249116483401	4/13/2000	5/13/2015	15.1	110	41.7%	58.3%	0.0%	41.7%	58.3%	0.0%
345330116425501	10/30/2002	5/10/2017	14.5	61	0.0%	97.7%	2.3%	0.0%	97.7%	2.3%
345446116485101	4/12/2000	11/8/2013	13.6	73	0.0%	75.7%	24.3%	0.0%	75.7%	24.3%
345600116523901	4/12/2000	5/9/2017	17.1	69	0.0%	94.1%	5.9%	0.0%	93.6%	6.4%
344230117200501	4/11/2000	5/15/2014	14.1	61	3.2%	91.3%	5.5%	3.2%	91.3%	5.5%
373818118513301	1/20/2000	1/31/2018	18.0	0	0.0%	83.2%	16.8%	0.0%	83.2%	16.8%

Online resource (suppplementary table) 4. Selected attributes of wells evaluated for time-series trends in orthophosphate concentrations-page 12.

USGS Station ID[1]	Agricultural land use in 1992[9] (percent)	Natural land use in 1992[9] (percent)	Urban land use in 1992[9] (percent)	Agricultural land use in 2002[9] (percent)	Natural land use in 2002[9] (percent)	Urban land use in 2002[9] (percent)	Agricultural land use in 2012[9] (percent)	Natural land use in 2012[9] (percent)	Urban land use in 2012[9] (percent)	Septic Tanks[8]	Aridity[9]
335231116345401	0.0%	76.1%	23.9%	0.0%	75.7%	24.3%	0.0%	75.7%	24.3%	9.31	0.150
335304116353001	0.0%	77.1%	22.9%	0.0%	77.1%	22.9%	0.0%	77.1%	22.9%	11.98	0.140
335318116363301	0.0%	14.3%	85.7%	0.0%	14.3%	85.7%	0.0%	14.3%	85.7%	5.08	0.150
335339116345301	0.0%	100.0%	0.0%	0.0%	100.0%	0.0%	0.0%	100.0%	0.0%	13.67	0.133
335339116345302	0.0%	100.0%	0.0%	0.0%	100.0%	0.0%	0.0%	100.0%	0.0%	13.67	0.133
335339116345303	0.0%	100.0%	0.0%	0.0%	100.0%	0.0%	0.0%	100.0%	0.0%	13.67	0.133
335348116352701	0.0%	100.0%	0.0%	0.0%	100.0%	0.0%	0.0%	100.0%	0.0%	13.52	0.140
335348116352702	0.0%	100.0%	0.0%	0.0%	100.0%	0.0%	0.0%	100.0%	0.0%	13.52	0.140
335348116352703	0.0%	100.0%	0.0%	0.0%	100.0%	0.0%	0.0%	100.0%	0.0%	13.52	0.140
335532116471701	0.5%	97.3%	2.3%	0.5%	97.3%	2.3%	0.5%	97.3%	2.3%	13.45	0.266
342300117160301	0.0%	45.9%	54.1%	0.0%	45.0%	55.0%	0.0%	45.0%	55.0%	48.36	0.155
342301117205001	0.0%	0.9%	99.1%	0.0%	0.9%	99.1%	0.0%	0.9%	99.1%	33.36	0.220
342450117151201	0.0%	0.0%	100.0%	0.0%	0.0%	100.0%	0.0%	0.0%	100.0%	148.27	0.129
342639117194001	0.0%	0.0%	100.0%	0.0%	0.0%	100.0%	0.0%	0.0%	100.0%	146.13	0.159
342726117082401	0.0%	97.7%	2.3%	0.0%	97.7%	2.3%	0.0%	97.7%	2.3%	7.59	0.116
342743117211701	0.0%	63.1%	36.9%	0.0%	61.8%	38.2%	0.0%	0.0%	100.0%	41.21	0.144
342918117153201	0.0%	0.9%	99.1%	0.0%	0.9%	99.1%	0.0%	0.9%	99.1%	0.00	0.099
342931117201501	0.0%	0.0%	100.0%	0.0%	0.0%	100.0%	0.0%	0.0%	100.0%	152.37	0.126
342953117173101	0.0%	0.0%	100.0%	0.0%	0.0%	100.0%	0.0%	0.0%	100.0%	29.12	0.107
343030117300901	0.0%	76.7%	23.3%	0.0%	76.3%	23.7%	0.0%	76.3%	23.7%	4.26	0.130
343030117300902	0.0%	76.7%	23.3%	0.0%	76.3%	23.7%	0.0%	76.3%	23.7%	4.23	0.130
343038117341701	0.0%	84.0%	16.0%	0.0%	79.5%	20.5%	0.0%	79.0%	21.0%	1.45	0.132
343341117101601	0.0%	0.0%	100.0%	0.0%	0.0%	100.0%	0.0%	0.0%	100.0%	1.79	0.097
344927116394101	0.0%	83.3%	16.7%	0.0%	83.3%	16.7%	0.0%	83.3%	16.7%	0.30	0.070
345153117080701	0.0%	90.9%	9.1%	0.0%	90.4%	9.6%	0.0%	90.4%	9.6%	12.70	0.088
345249116483401	41.7%	58.3%	0.0%	41.7%	58.3%	0.0%	40.8%	59.2%	0.0%	3.82	0.065
345330116425501	0.0%	97.7%	2.3%	0.0%	97.2%	2.8%	0.0%	97.2%	2.8%	3.82	0.063
345446116485101	0.0%	75.7%	24.3%	0.0%	75.7%	24.3%	0.0%	75.7%	24.3%	1.88	0.073
345600116523901	0.0%	90.9%	9.1%	0.0%	89.5%	10.5%	0.0%	89.5%	10.5%	1.88	0.074
344230117200501	2.3%	92.2%	5.5%	2.3%	91.8%	5.9%	6.8%	87.2%	5.9%	1.11	0.077
373818118513301	0.0%	82.7%	17.3%	0.0%	82.7%	17.3%	0.0%	82.7%	17.3%	0.33	0.397

Online resource (suppplementary table) 4. Selected attributes of wells evaluated for time-series trends in orthophosphate concentrations-page 13.

USGS Station ID[1]	GAMA-PBP ID	Well location relative to GAMA-PBP project study unit	Hydrogeologic zone	Kendall's tau τ (correlation coefficient)[3]	p-value[4] (values ≤ 0.05 are bolded)	Sen slope estimate (rate of change in mg/L/yr as P)[5]	Trend test outcome[4]
373822118514401		Owens and Indian Wells Valleys	Desert	0.699	**<0.001**	1.85E-03	Increase
373829118505801		Owens and Indian Wells Valleys	Desert	0.692	**<0.001**	2.23E-03	Increase
334631117504101	CLABOC-01	Coastal Los Angeles Basins	Southern California	0.643	**0.035**	1.47E-03	Increase
335826116580501		Upper Santa Ana Watershed	Southern California	0.127	0.640	5.05E-04	no trend
340105117031601	USAWY-05	Upper Santa Ana Watershed	Southern California	0.405	0.127	9.42E-04	no trend
340406117351701	USAWC-17	Upper Santa Ana Watershed	Southern California	0.704	**0.012**	1.99E-03	Increase
340425117164401		Upper Santa Ana Watershed	Southern California	0.458	0.134	7.39E-04	no trend
340437117170301	USAWB-07	Upper Santa Ana Watershed	Southern California	0.429	0.174	9.23E-04	no trend
340441117153501		Upper Santa Ana Watershed	Southern California	0.423	0.142	1.30E-03	no trend
340447117152701		Upper Santa Ana Watershed	Southern California	0.479	0.093	1.43E-03	no trend
340508117150401		Upper Santa Ana Watershed	Southern California	0.500	0.108	6.09E-04	no trend
340544117103701		Upper Santa Ana Watershed	Southern California	0.141	0.675	2.55E-04	no trend
340617117163601	USAWB-16	Upper Santa Ana Watershed	Southern California	-0.113	0.799	-7.56E-05	no trend
340627117101401	USAWB-12	Upper Santa Ana Watershed	Southern California	0.565	**0.013**	1.37E-03	Increase
340717117194601	USAWB-08	Upper Santa Ana Watershed	Southern California	0.429	0.174	1.06E-03	no trend
340846117160501		Upper Santa Ana Watershed	Southern California	0.278	0.348	6.21E-04	no trend
340858117152002	USAWB-11	Upper Santa Ana Watershed	Southern California	0.378	0.152	6.98E-04	no trend

Online resource (suppplementary table) 4. Selected attributes of wells evaluated for time-series trends in orthophosphate concentrations-page 14.

USGS Station ID[1]	First sample date of time series	Last sample date of time series	Time series length (years)	Well depth (meters below LSD)[6]	Agricultural land use in 1974[9] (percent)	Natural land use in 1974[9] (percent)	Urban land use in 1974[9] (percent)	Agricultural land use in 1982[9] (percent)	Natural land use in 1982[9] (percent)	Urban land use in 1982[9] (percent)
373822118514401	1/20/2000	1/31/2018	18.0	0	0.0%	88.9%	11.1%	0.0%	88.9%	11.1%
373829118505801	1/20/2000	1/31/2018	18.0	0	0.0%	95.8%	4.2%	0.0%	95.8%	4.2%
334631117504101	5/28/2003	7/27/2016	13.2	396	10.5%	0.0%	89.5%	2.7%	0.0%	97.3%
335826116580501	6/10/2003	6/26/2017	14.1	348	3.6%	6.4%	90.0%	26.8%	4.1%	69.1%
340105117031601	5/4/2000	1/30/2017	16.8	180	0.0%	0.0%	100.0%	0.0%	0.0%	100.0%
340406117351701	4/4/2000	5/16/2012	12.1	274	15.0%	0.0%	85.0%	7.7%	0.0%	92.3%
340425117164401	5/22/2000	5/29/2008	8.0	337	0.0%	0.0%	100.0%	0.0%	0.0%	100.0%
340437117170301	5/17/2000	5/15/2012	12.0	199	0.0%	0.0%	100.0%	0.0%	0.0%	100.0%
340441117153501	5/23/2000	3/11/2009	8.8	99	19.0%	0.0%	81.0%	8.3%	0.0%	91.7%
340447117152701	5/23/2000	5/28/2008	8.0	87	25.6%	0.0%	74.4%	11.0%	0.0%	89.0%
340508117150401	5/24/2000	3/11/2009	8.8	126	0.0%	0.0%	100.0%	0.0%	0.0%	100.0%
340544117103701	5/25/2000	5/29/2008	8.0	152	0.0%	9.5%	90.5%	0.0%	10.0%	90.0%
340617117163601	5/24/2000	1/8/2007	6.6	294	0.0%	0.0%	100.0%	0.0%	0.0%	100.0%
340627117101401	10/25/2000	2/1/2017	16.3	290	3.6%	0.0%	96.4%	1.8%	0.0%	98.2%
340717117194601	4/5/2000	10/10/2012	12.5	177	4.7%	0.0%	95.3%	1.9%	0.0%	98.1%
340846117160501	5/22/2000	5/27/2008	8.0	210	0.0%	0.0%	100.0%	0.0%	0.0%	100.0%
340858117152002	5/24/2000	2/1/2017	16.7	185	0.0%	0.0%	100.0%	0.0%	0.0%	100.0%

Online resource (suppplementary table) 4. Selected attributes of wells evaluated for time-series trends in orthophosphate concentrations-page 15.

USGS Station ID[1]	Agricultural land use in 1992[9] (percent)	Natural land use in 1992[9] (percent)	Urban land use in 1992[9] (percent)	Agricultural land use in 2002[9] (percent)	Natural land use in 2002[9] (percent)	Urban land use in 2002[9] (percent)	Agricultural land use in 2012[9] (percent)	Natural land use in 2012[9] (percent)	Urban land use in 2012[9] (percent)	Septic Tanks[8]	Aridity[9]
373822118514401	0.0%	88.5%	11.5%	0.0%	88.5%	11.5%	0.0%	88.5%	11.5%	0.33	0.404
373829118505801	0.0%	94.9%	5.1%	0.0%	94.9%	5.1%	0.0%	94.9%	5.1%	0.33	0.396
334631117504101	0.0%	0.0%	100.0%	0.0%	0.0%	100.0%	0.0%	0.0%	100.0%	9.97	0.248
335826116580501	3.6%	6.4%	90.0%	3.6%	6.4%	90.0%	3.6%	4.5%	91.8%	65.03	0.372
340105117031601	0.0%	0.0%	100.0%	0.0%	0.0%	100.0%	0.0%	0.0%	100.0%	15.87	0.329
340406117351701	0.0%	0.0%	100.0%	0.0%	0.0%	100.0%	0.0%	0.0%	100.0%	5.37	0.295
340425117164401	0.0%	0.0%	100.0%	0.0%	0.0%	100.0%	0.0%	0.0%	100.0%	9.10	0.231
340437117170301	0.0%	0.0%	100.0%	0.0%	0.0%	100.0%	0.0%	0.0%	100.0%	1.36	0.231
340441117153501	0.0%	0.0%	100.0%	0.0%	0.0%	100.0%	0.0%	0.0%	100.0%	79.92	0.236
340447117152701	0.0%	0.0%	100.0%	0.0%	0.0%	100.0%	0.0%	0.0%	100.0%	92.00	0.236
340508117150401	0.0%	0.0%	100.0%	0.0%	0.0%	100.0%	0.0%	0.0%	100.0%	84.38	0.237
340544117103701	0.0%	10.5%	89.5%	0.0%	10.5%	89.5%	0.0%	10.5%	89.5%	10.95	0.245
340617117163601	0.0%	0.0%	100.0%	0.0%	0.0%	100.0%	0.0%	0.0%	100.0%	35.97	0.245
340627117101401	0.0%	0.0%	100.0%	0.0%	0.0%	100.0%	0.0%	0.0%	100.0%	19.27	0.252
340717117194601	0.0%	0.0%	100.0%	0.0%	0.0%	100.0%	0.0%	0.0%	100.0%	11.96	0.289
340846117160501	0.0%	0.0%	100.0%	0.0%	0.0%	100.0%	0.0%	0.0%	100.0%	8.06	0.296
340858117152002	0.0%	0.0%	100.0%	0.0%	0.0%	100.0%	0.0%	0.0%	100.0%	1.64	0.300

Footnotes:

[1]Groundwater chemistry data are available through the USGS National Water Information System (NWIS) database at https://waterdata.usgs.gov/nwis. From there select "Water Quality", then "Field/Lab samples", and use these "Site Numbers" as "Site Identifier."

[2]The well S4-TUSK-TLE06 was sampled for the GAMA-PBP Tulare study unit Shallow Aquifer Assessment (Fram, 2017). However, the depth of this well is comparable to those of wells sampled for the GAMA-PBP Southeast San Joaquin Valley study unit Public Supply Aquifer Assessment (Burton, 2012), which is located in approximately the same geographic area of California.

Fram, M. S., (2017). *Groundwater quality in the shallow aquifers of the Tulare, Kaweah, and Tule Groundwater Basins and adjacent highlands areas, Southern San Joaquin Valley, California.* U.S. Geological Survey Fact Sheet 2017-3001, 4 p. U.S. Geological Survey, Sacramento CA.
Burton, C. A., (2012). *Groundwater quality in the Southeast San Joaquin Valley, California.* U.S. Geological Survey Fact Sheet 2011-3151, 4 p. U.S. Geological Survey, Sacramento CA. https://pubs.usgs.gov/fs/2011/3151/

[3]The nonparametric Mann-Kendall trend test (Mann,1945; Helsel and Hirsch, 2002) was used to test for the significance of a Kendall's τ correlation of orthophosphate concentration and time

Mann, H. B. (1945). Nonparametric tests against trend. *Econometrica*, *13(3)* , 245-59. https://www.jstor.org/stable/pdf/1907187.pdf
Helsel, D. R., & Hirsch, R. M. (2002). Statistical methods in water resources. U.S. Geological Survey Techniques of Water-Resources Investigations, bk.4:chap.A3, U.S. Geological Survey, Reston VA. http://water.usgs.gov/pubs/twri/twri4a3/.

[4]Time-series trends were considered detected at attained significance levels [p]≤0.05.

[5]The Sen slope estimator was calculated to estimate trend magnitude (Sen 1968; Hirsch et al. 1991), or rate of change in orthophosphate concentrations in milligrams per liter as phoshporus per year.

Sen, P. K. (1968). Estimates of the regression coefficient based on Kendall's Tau. *Journal of the American Statistical Association, 63*:1379-89. https://www.pacificclimate.org/~wernera/zyp/Sen%201968%20JASA.pdf
Hirsch, R. M., Alexander, R. B., & Smith, R. A. (1991). Selection of methods for the detection and estimation of trends in water quality. *Water Resources Research 27* (5), 803-13.

[6]Well depth given as distance from the elevation of the land-surface datum (LSD) given in the previous column. Springs are assigned a well depth of zero. Unknown well depths are assigned "na", not applicable.

[7]Land use data were represented as percentages of the broad categories, agricultural, natural, and urban in discrete years spanning five decades; 1974, 1982, 1992, 2002, and 2012 (Falcone, 2015).

Falcone, J. A. (2015). U.S. conterminous wall-to-wall anthropogenic land use trends (NWALT), 1974–2012: U.S. Geological Survey Data Series 948, 33 p. plus appendixes 3–6 as separate files, http://dx.doi.org/10.3133/ds948.

[8]Septic tank density was determined from the 1990 Census of Population and Housing (U.S. Department of Commerce, 1992), and expressed as tanks/km2.

U.S. Department of Commerce. (1992), 1990 Census of population and housing, summary tape file 3A: U. S. Census Gureau, CD-ROM, http://www.census.gov/mp/www/cat/decennial_census_1990/1990_census_of_population_and_housing_summary_tape_file_3a.html.

[9]Aridity index is calculated as the average annual precipitation (PRISM Climate Group, 2012) divided by the average annual evapotranspiration (Flint and Flint, 2007), and values can range from 0.05 (hyper-arid) to greater than 1.00 (wet).

PRISM Climate Group, (2012). United States average annual precipitation, maximum and minimum temperature, 1971-2000: Oregon State University, PRISM website, accessed November 14, 2018 at http://prism.oregonstate.edu/
