# Supplementary material for: Status and trends of orthophosphate concentrations in groundwater used for public supply in California
Source: Environ Monit Assess. 2020 Jul 29;192(8):550. doi: 10.1007/s10661-020-08504-x (PMC7391407; doi:10.1007/s10661-020-08504-x)
Supplement: Supplementary file 5 — (PDF 231 kb) [file 10661_2020_8504_MOESM5_ESM.pdf]

Status and trends of orthophosphate concentrations in groundwater used for public supply in California Environmental Monitoring and Assessment, Robert Kent, Tyler D. Johnson, and Michael R. Rosen, U.S. Geological Survey California Water Science Center-rhkent@usgs.gov

Online resource (suppplementary table) 5. Tables of Eigen values, percents of variance and first 3 principle component loading factors for 19 variables -page 1.

Table 1. Eigen values and percent of variance for principle component analysis of static data.

Principle component number	Eigen Value	Percent of Variance	Cumulative percent of variance
1	3.29	17.34	17.34
2	2.37	12.49	29.83
3	2.21	11.62	41.45
4	1.37	7.22	48.67
5	1.27	6.69	55.36
6	1.12	5.88	61.24
7	1.02	5.37	66.61
8	0.96	5.05	71.66
9	0.84	4.42	76.08
10	0.77	4.05	80.14
11	0.70	3.69	83.83
12	0.60	3.14	86.97
13	0.58	3.04	90.00
14	0.53	2.79	92.79
15	0.48	2.52	95.31
16	0.42	2.21	97.52
17	0.36	1.89	99.41
18	0.11	0.59	100.00
19	0.00	0.00	100.00

Table 2. First 3 principle component loading factors for Static analysis data. Loading values greater than ±0.4 are bolded.

	Loadings		
Parameter	PC1	PC2	PC3
Orthophosphate	0.069	0.004	0.148
pH	0.161	**0.469**	-0.032
Dissolved Oxygen	-0.261	-0.183	-0.142
As	0.108	0.171	0.219
Mg	0.391	-0.350	0.023
B	0.236	0.050	0.243
Total Dissolved Solids	**0.458**	-0.177	0.100
HCO_3	0.341	-0.126	0.052
NO_3 as N	0.180	-0.223	-0.233
U	0.008	-0.023	0.071
F	0.124	0.286	0.156
Mn	0.163	-0.178	0.093
Well Depth	0.127	0.340	-0.220
Aridity	-0.348	-0.207	0.117
Septic tank density	-0.026	0.053	-0.352
Age rank	0.160	**0.435**	0.089
2002 Agriculture land use	0.238	-0.153	0.039
2002 Natural land use	-0.241	0.041	**0.508**
2002 Urban land use	0.055	0.079	**-0.539**

Table 3. Eigen values and percent of variance for principle component analysis of E1 step-trend data.

Principle component number	Eigen Value	Percent of Variance	Cumulative percent of variance
1	3.92	20.63	20.63
2	2.16	11.36	31.99
3	1.89	9.95	41.94
4	1.53	8.03	49.97
5	1.32	6.92	56.89
6	1.18	6.21	63.10
7	1.03	5.40	68.50
8	0.98	5.17	73.67
9	0.90	4.73	78.40
10	0.73	3.86	82.27
11	0.70	3.68	85.94
12	0.66	3.48	89.43
13	0.47	2.47	91.90
14	0.44	2.31	94.21
15	0.42	2.24	96.44
16	0.29	1.51	97.95
17	0.22	1.14	99.09
18	0.17	0.88	99.97
19	0.01	0.03	100.00

Table 4. First 3 principle component loading factors for E1 step-trend analysis data. Loading values greater than ±0.4 are bolded.

	Loadings		
Parameter	PC1	PC2	PC3
Slope Orthophosphate	0.264	-0.116	0.178
Slope pH	0.163	0.192	0.391
Slope Total Dissolved Solids	0.308	0.014	-0.363
Slope Dissolved Oxygen	-0.057	0.331	0.104
Slope Mg	**0.438**	0.085	-0.030
Slope As	0.176	-0.047	-0.114
Slope B	0.239	0.005	0.044
Slope Mn	0.341	0.056	0.338
Slope NO_3as N	0.117	0.016	-0.377
Slope Alkalinity	0.385	0.030	0.042
Slope U	0.125	0.233	-0.331
Slope F	-0.046	0.057	0.190
Slope SO_4	0.326	0.095	-0.364
Slope Agricultural land use	-0.192	0.187	-0.192
Slope Natural land use	0.011	**0.552**	0.111
Slope Urban land use	0.095	**-0.627**	0.005
Well Depth	0.226	-0.130	0.242
Septic Tank Density	0.034	-0.040	0.023
Water Age Rank	0.158	0.090	0.110

Online resource (suppplementary table) 5. Tables of Eigen values, percents of variance and first 3 principle component loading factors for 19 variables-page 5.

Table 5. Eigen values and percent of variance for principle component analysis of E2 step-trend data.

Principle component number	Eigen Value	Percent of Variance	Cumulative percent of variance
1	4.81	25.31	25.31
2	2.14	11.27	36.58
3	1.63	8.59	45.17
4	1.50	7.90	53.07
5	1.09	5.73	58.80
6	1.06	5.56	64.36
7	1.02	5.36	69.72
8	0.96	5.04	74.76
9	0.86	4.53	79.29
10	0.84	4.42	83.71
11	0.73	3.83	87.54
12	0.67	3.50	91.04
13	0.58	3.06	94.09
14	0.50	2.64	96.74
15	0.25	1.29	98.03
16	0.20	1.05	99.07
17	0.10	0.54	99.61
18	0.07	0.35	99.96
19	0.01	0.04	100.00

Online resource (suppplementary table) 5. Tables of Eigen values, percents of variance and first 3 principle component loading factors for 19 variables-page 6.

Table 6. First 3 principle component loading factors for E2 step-trend analysis data. Loading values greater than ±0.4 are bolded.

	Loadings		
Parameter	PC1	PC2	PC3
Slope Agricultural land use	0.008	-0.352	**-0.446**
Slope Natural land use	0.002	**-0.444**	**0.471**
Slope Urban land use	-0.006	**0.643**	-0.065
Well Depth	-0.025	0.322	**0.423**
Septic Tank density	-0.008	0.075	0.229
Water Age Rank	-0.003	0.086	**0.408**
Slope Orthophosphate	-0.056	0.064	-0.188
Slope pH	-0.070	-0.128	0.201
Slope Total Dissolved Solids	**0.428**	0.031	0.013
Slope Dissolved Oxygen	0.145	-0.288	0.168
Slope As	-0.034	0.094	0.082
Slope Mg	0.415	0.020	0.027
Slope B	0.159	0.025	0.160
Slope Mn	**0.412**	0.039	-0.022
Slope NO_3-N	**0.396**	-0.035	0.022
Slope HCO_3	0.161	0.169	-0.055
Slope U	0.193	-0.011	-0.080
Slope F	-0.111	-0.063	0.174
Slope SO_4	**0.433**	-0.009	-0.001

Table 7. Eigen values and percent of variance for principle component analysis of E3 step-trend data.

Principle component number	Eigen Value	Percent of Variance	Cumulative percent of variance
1	3.18	16.72	16.72
2	2.11	11.12	27.84
3	1.86	9.79	37.62
4	1.55	8.16	45.78
5	1.42	7.45	53.24
6	1.36	7.14	60.38
7	1.30	6.82	67.20
8	0.95	5.01	72.21
9	0.87	4.58	76.80
10	0.83	4.39	81.19
11	0.76	3.98	85.17
12	0.60	3.14	88.30
13	0.55	2.89	91.19
14	0.49	2.59	93.78
15	0.43	2.26	96.04
16	0.33	1.74	97.78
17	0.24	1.28	99.06
18	0.17	0.90	99.96
19	0.01	0.05	100.00

Table 8. First 3 principle component loading factors for E3 step-trend analysis data. Loading values greater than ±0.4 are bolded.

	Loadings		
Parameter	PC1	PC2	PC3
Slope Agricultural land use	0.056	-0.248	-0.119
Slope Natural land use	-0.080	**-0.496**	0.153
Slope Urban land use	0.031	**0.615**	-0.048
Well Depth	0.005	0.002	0.208
Septic Tank density	-0.123	0.099	0.007
Water Age Rank	0.015	-0.077	**0.472**
Slope Orthophosphate	-0.173	0.138	0.363
Slope pH	-0.027	-0.133	-0.332
Slope Total Dissolved Solids	**0.490**	-0.005	-0.097
Slope Dissolved Oxygen	0.040	-0.190	0.271
Slope As	-0.001	0.108	0.031
Slope Mg	0.400	-0.048	0.261
Slope B	0.316	0.108	-0.088
Slope Mn	0.038	0.387	0.321
Slope NO_3-N	0.277	-0.188	0.243
Slope HCO_3	**0.418**	0.006	0.145
Slope U	0.231	0.018	-0.251
Slope F	-0.008	-0.116	-0.150
Slope SO_4	0.367	0.036	-0.153

Table 9. Eigen values and percent of variance for principle component analysis of time-series trend data.

Principle component number	Eigen Value	Percent of Variance	Cumulative percent of variance
1	3.33	23.82	23.82
2	1.74	12.44	36.26
3	1.46	10.39	46.65
4	1.34	9.57	56.22
5	1.12	7.98	64.20
6	1.04	7.42	71.63
7	0.91	6.49	78.11
8	0.89	6.36	84.47
9	0.87	6.18	90.65
10	0.65	4.67	95.33
11	0.37	2.66	97.99
12	0.26	1.88	99.87
13	0.02	0.13	100
14	0.00	0.00	100

Table 10. First 3 principle component loading factors for time-series trend analysis data. Loading values greater than ±0.4 are bolded.

	Loadings		
Parameter	PC1	PC2	PC3
Orthophosphate slope	0.049	-0.112	0.164
Well Depth	-0.029	0.206	-0.197
pH slope	0.006	0.018	0.043
Total Dissolved Solids (TDS) slope	**-0.439**	0.055	-0.043
Alkalinity Slope	-0.070	-0.223	0.232
Mg slope	**-0.513**	0.024	0.000
B slope	-0.350	0.053	-0.006
Mn slope	**-0.418**	-0.020	0.089
NO_3 as N slope	-0.045	-0.230	-0.222
F slope	-0.046	0.111	0.087
SO_4 slope	**-0.482**	-0.003	-0.008
Change in Agriculture land use	-0.017	-0.263	**-0.731**
Change in Natural land use	-0.018	**-0.515**	**0.499**
Change in Urban land use	0.031	**0.699**	0.170
